# Interactions Increase Forager Availability and Activity in Harvester Ants

**DOI:** 10.1371/journal.pone.0141971

**Published:** 2015-11-05

**Authors:** Evlyn Pless, Jovel Queirolo, Noa Pinter-Wollman, Sam Crow, Kelsey Allen, Maya B. Mathur, Deborah M. Gordon

**Affiliations:** 1 Department of Biology, Stanford University, Stanford, California, United States of America; 2 BioCircuits Institute, University of California, San Diego, La Jolla, California, United States of America; 3 Department of Computer Science and Engineering, University of Washington, Seattle, Washington, United States of America; 4 Department of Brain and Cognitive Sciences, Massachusetts Institute of Technology, Cambridge, Massachusetts, United States of America; 5 Quantitative Sciences Unit, Stanford University, Stanford, California, United States of America; Universidade de São paulo, BRAZIL

## Abstract

Social insect colonies use interactions among workers to regulate collective behavior. Harvester ant foragers interact in a chamber just inside the nest entrance, here called the 'entrance chamber'. Previous studies of the activation of foragers in red harvester ants show that an outgoing forager inside the nest experiences an increase in brief antennal contacts before it leaves the nest to forage. Here we compare the interaction rate experienced by foragers that left the nest and ants that did not. We found that ants in the entrance chamber that leave the nest to forage experienced more interactions than ants that descend to the deeper nest without foraging. Additionally, we found that the availability of foragers in the entrance chamber is associated with the rate of forager return. An increase in the rate of forager return leads to an increase in the rate at which ants descend to the deeper nest, which then stimulates more ants to ascend into the entrance chamber. Thus a higher rate of forager return leads to more available foragers in the entrance chamber. The highest density of interactions occurs near the nest entrance and the entrances of the tunnels from the entrance chamber to the deeper nest. Local interactions with returning foragers regulate both the activation of waiting foragers and the number of foragers available to be activated.

## Introduction

A fundamental question in the study of animal behavior and other networks is how simple individual behaviors add up to complex collective behaviors [1]. Distributed networks, including those found in natural populations, are regulated using feedback based on local interactions [2,3]. Social insect colonies offer a compelling example of collective behavior, as they operate without any central control [4].

In social insect colonies, individual workers use local interactions to perform and regulate collective behavior [4,5], such as nest construction [6,7], nest relocation [8,9,10], and foraging [11,12,13,14,15]. Many studies suggest that wasps, honeybees, and ants all use interaction rate to activate foraging [14,15, 16, 17]. In wasps, foragers may be activated by the biting of incoming foragers and other nestmates, and foragers are bitten more frequently than other wasps [14]. In honeybees, forager arrival and interactions inside the hive [15], such as the waggle dance [18] and vibration signals [19], are associated with an increase in outgoing foraging [15,19], while another 'stop signal’ may counter the waggle dance [18].

Local encounters also play an important role for the organization of foraging in ants, for example to respond to the intensity of crowding [20,21,22]. Ants appear to use the rate of antennal contact as an indication of local density [23,24,25] and adjust recruitment and trail networks accordingly [20,21,22]. For example, *Lasius niger* ants downregulate the production of recruitment signals [22] and bifurcate their trail during crowding to maintain a high rate of food return [20].

It appears that interactions also regulate the availability of foragers. For example, interactions among honeybees affect hormonal factors that determine the onset of foraging and therefore the number of bees available to forage [26]. Depleting a colony of the older foragers induces an early onset of foraging in younger bees [26]. Removal experiments in ants also suggest that the availability of foragers depends on interactions, because the removal of foragers leads other ants to switch tasks and become foragers [27, 28].

Red harvester ants (*Pogonomyrmex barbatus*) use interaction rates to decide which task to perform, including whether and when to forage [12,13,29]. Interactions between harvester ants are tactile and chemical and take the form of brief antennal contacts [30,31]. During an antennal contact, one ant assesses the cuticular hydrocarbon profile of the other, which reveals an ant’s task and whether it is carrying food [30,31,32]. Foraging poses trade-offs for seed-eating ants in the desert. Ants must spend water to gain water because foraging in the desert heat can lead to desiccation, and they obtain some water by metabolizing the fats in the seeds they collect [33,34]. In addition, colonies compete with their neighbors for foraging area [35,36]. How well a colony manages these trade-offs influences its reproductive success [37].

**Fig 1:**
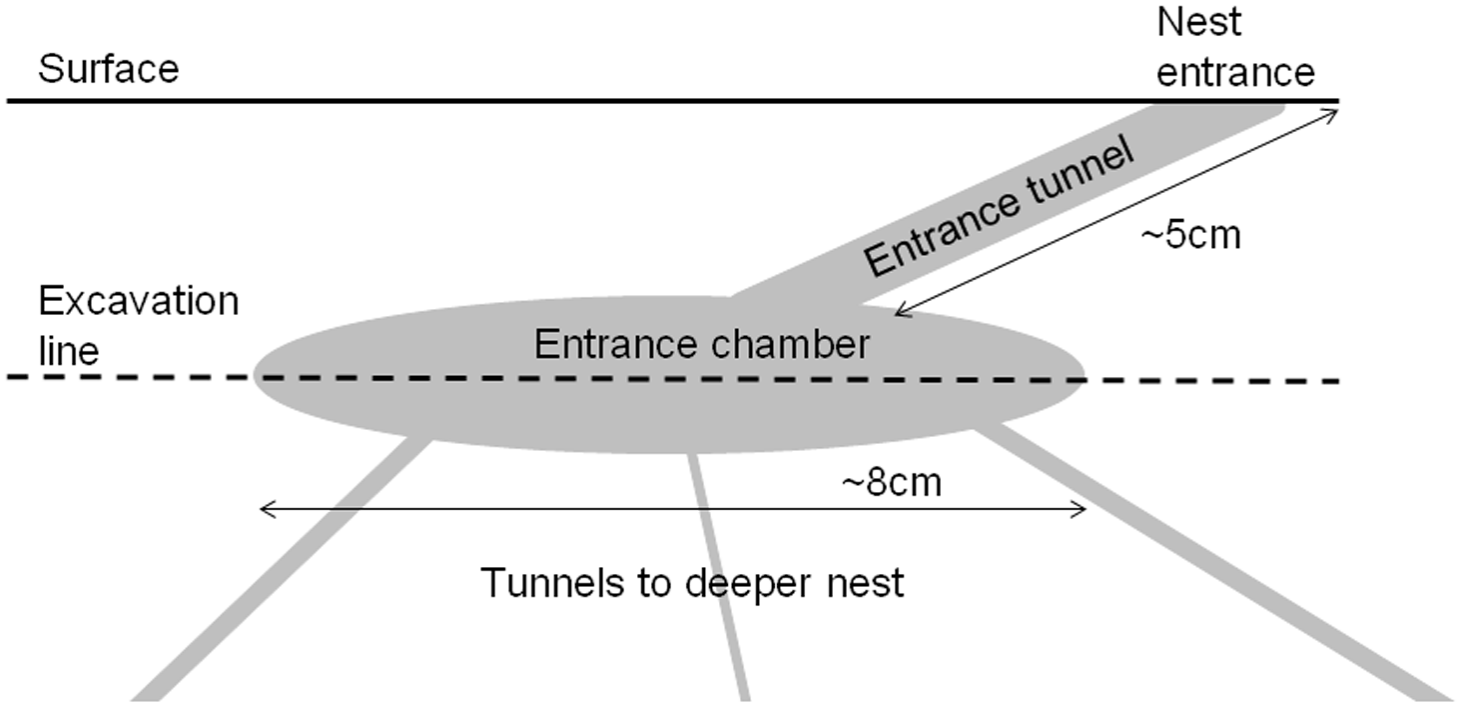
Diagram of the entrance chamber. A schematic vertical cross section of a typical *P*. *barbatus* ant nest. The solid line indicates the ground surface, and the dashed line indicates where a horizontal section was made to observe activity inside the nest. Interactions occur in the entrance chamber, connected to the outside by the entrance tunnel, with tunnels leading to the deeper nest.

In harvester ants, returning foragers come into the nest entrance through a tunnel and into a chamber just inside the entrance that we call the 'entrance chamber' (Fig 1). In previous work we referred to the 'vestibule' as the area just inside the nest entrance [13], but in this study we excavated more deeply to observe ants in the full chamber at the end of the entrance tunnel. Foragers deposit the food they collected in this chamber or carry it down tunnels leading from the entrance chamber to the deeper nest. The sizes and shapes of tunnels and chambers vary among colonies, and these differences probably influence the rate and location of interactions. Nest structure affects ant movement [38], which affects the rate of interaction [39,40]. In turn, rate of interaction can influence traffic flow [41,42].

Here we consider three questions about the regulation of foraging through interaction rate in harvester ants:

### Question 1: Do outgoing foragers experience a different interaction rate from other ants?

Studies on harvester ants have shown that interactions with successful returning foragers stimulate foraging activity [12,43]. The chemical odors of both a returning forager and the seed it is carrying are required in combination to stimulate foraging [32]. A previous study showed that outgoing foragers experience a substantial increase in interactions 3–8 seconds before they choose to leave the nest [13]. However, this study examined the interaction rates only of the foragers that left the nest. Here we test whether ants that left the nest to forage experienced a higher interaction rate than ants that did not leave the nest to forage and instead descended from the entrance chamber to the deeper nest without foraging.

### Question 2: What regulates the number of outgoing foragers available in the entrance chamber?

Previous work on harvester ants shows that while foragers inside the nest respond to interactions within seconds, the pool of ants available to become foragers is regulated on the order of minutes [13]. Inhibiting forager return for more than four minutes leads to a decrease in the number of foragers inside the nest entrance that are available to forage (S1 Movie) [13].

Here we examine how the rate of returning foragers regulates the number of ants in the entrance chamber available to forage. We hypothesized that the descent into the deeper nest of returning foragers, or other ants transporting food within the nest, influences the rate at which ants ascend from the deeper nest to the entrance chamber to become available to forage. We manipulated the rate of forager return to determine the effect on the rate at which ants ascend from the deeper nest, and return to the deeper nest, thus regulating the numbers in the entrance chamber available to forage.

### Question 3: What determines the spatial distribution of interactions?

Previous work shows that an ant’s interaction frequency depends on its path shape and location [35,38,39,40,41,42,44,45]. By influencing interactions, the shape and size of the entrance chamber, which varies among colonies, probably affects the regulation of foraging in harvester ants. Previous studies show that most interactions occur at the entrances of the tunnels from the entrance chamber to the deeper nest [13]. We asked how manipulating the rate of incoming foragers would change the spatial distribution and frequency of interactions in the nest.

## Materials and Methods

Data were collected over the course of one week in August 2012 and two weeks in August 2013 at the site of a long-term study in Rodeo, New Mexico [37]. Prof. Gordon’s long-term study site is owned by Stanford University, and no permission was required to work on the site. This study did not involve any endangered or protected species.

Nests of *P*. *barbatus* have an entrance approximately 2cm in diameter, which leads to one or more entrance tunnels approximately 5cm long. The entrance tunnels lead into an entrance chamber approximately 8cm wide, and from this chamber, tunnels descend into the deeper nest (Fig 1).

**Fig 2:**
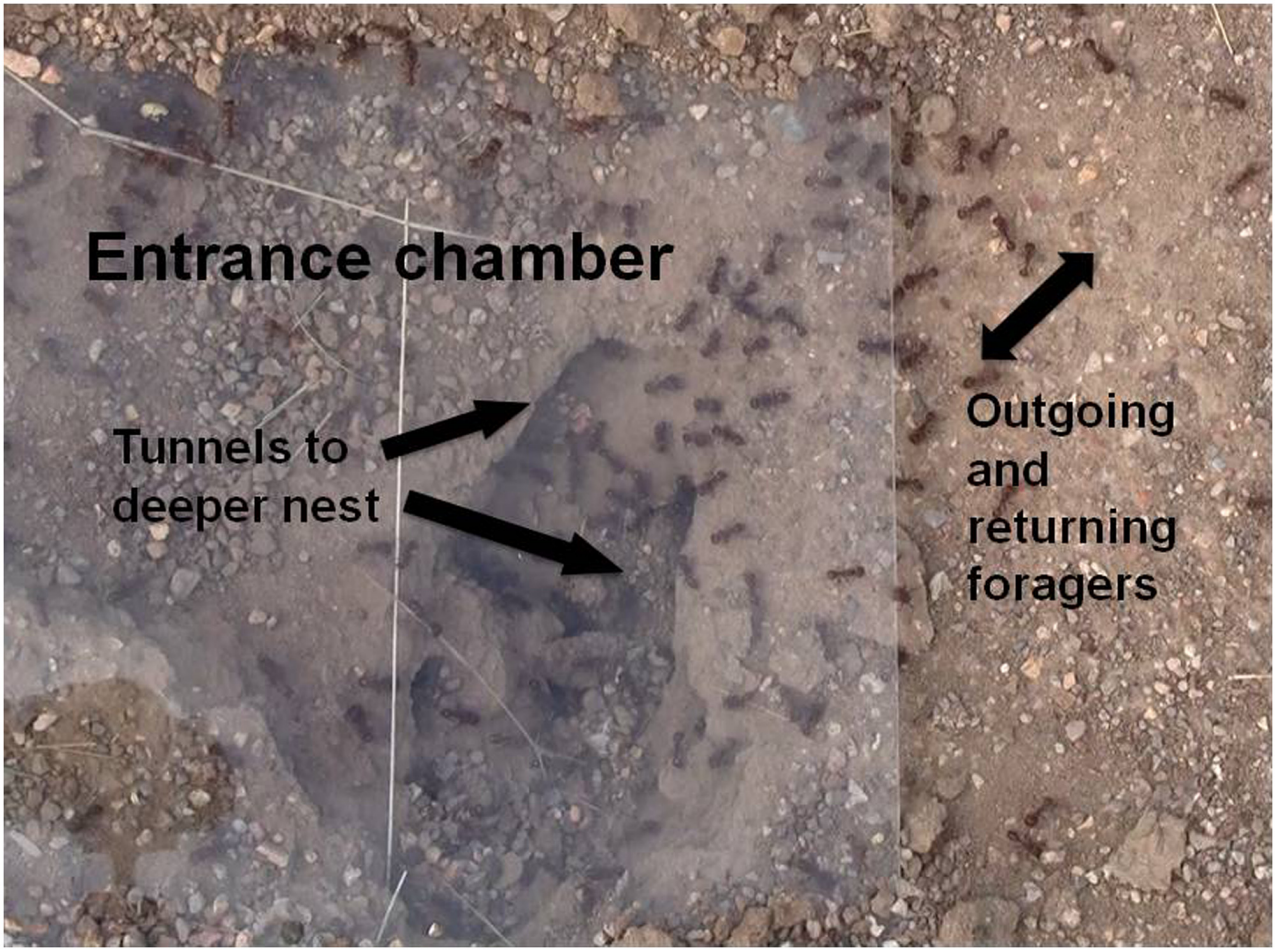
Labeled entrance chamber. A photograph of the entrance chamber of colony N_4 covered with the window used during filming. The tunnels to the deeper nest are labeled, as is the trail of returning and outgoing foragers.

To observe interactions inside the entrance chamber, we excavated the soil above the entrance chamber, as in Pinter-Wollman *et al*. [13]. We positioned a rectangular piece of plywood (23cm x 28cm) such that one of the short edges of the wood was directly over the nest entrance. The rest of the wood was positioned over the entrance tunnel and entrance chamber. We drew an outline around the wood, removed the wood, and dug out approximately 4cm of soil from the rectangular area for all films made in 2012 and approximately 9cm of soil for films made in 2013. The area exposed in 2012 corresponds to the area inside the nest entrance described as the 'vestibule' in Pinter-Wollman *et al*. [13]. The area we exposed in the 2013 observations described here included more of the entrance chamber than in 2012. Here we refer to both as the 'entrance chamber'. We covered the area with the wood overnight so that the ants would acclimate to their new nest ceiling. Each morning between 6am and 11am, we removed the wood and covered the excavated area with a rectangular glass sheet (23cm x 28cm) for filming (Fig 2). Previous studies show that the incoming light does not disturb the ants [13].

In 2012 we filmed three colonies for three consecutive days, and in 2013 we filmed six colonies over a period of 10 days (S1 Table). We converted the films into JPEGs (30 frames/second) using Adobe Premiere Elements 7.0 in 2012 and Java software that we developed in 2013. We observed the films of ants in the entrance chamber, which included all area covered by the glass sheet and all area immediately around the glass window which was depressed at the same level as the rest of the ant chamber.

When observing the films for each of our analyses, we used the following definitions for categorizing ant activities:

1. Outgoing foragers: ants that leave the entrance chamber and go outside not carrying anything
2. Returning foragers: ants that come from outside of the nest and enter the nest (Observations were made at a time when most ants outside the nest were foragers, but it is possible that some of the ants considered to be returning foragers were ants of another task that did not carry a food item.)
3. Descending ants: ants that were first observed in the entrance chamber and go down a tunnel into the deeper nest
4. Ascending ants: ants that were in the deeper nest, come up a tunnel, and emerge into the entrance chamber
5. Nest maintenance workers: ants carrying dirt or debris inside the nest

To test whether outgoing foragers experience a different interaction rate from other ants (Question 1), we made 3 sets of measurements from the films made in 2012. First, we determined what percentage of the ants that ascended from the deeper nest into the entrance chamber (‘ascending ants’) later performed each of the following: outgoing foraging, descending into the deeper nest, or nest maintenance. The focal ants for this analysis were chosen by selecting the first ant to emerge from the deeper nest at five-second intervals. Second, we observed outgoing foragers and recorded the time and location of all their interactions, in the form of brief antennal contacts, before leaving the entrance chamber. Third, we recorded the time and locations of the interactions of descending ants. The first clearly visible foraging and descending ants from each video were chosen as the focal ants for the second and third analyses.

**Fig 3:**
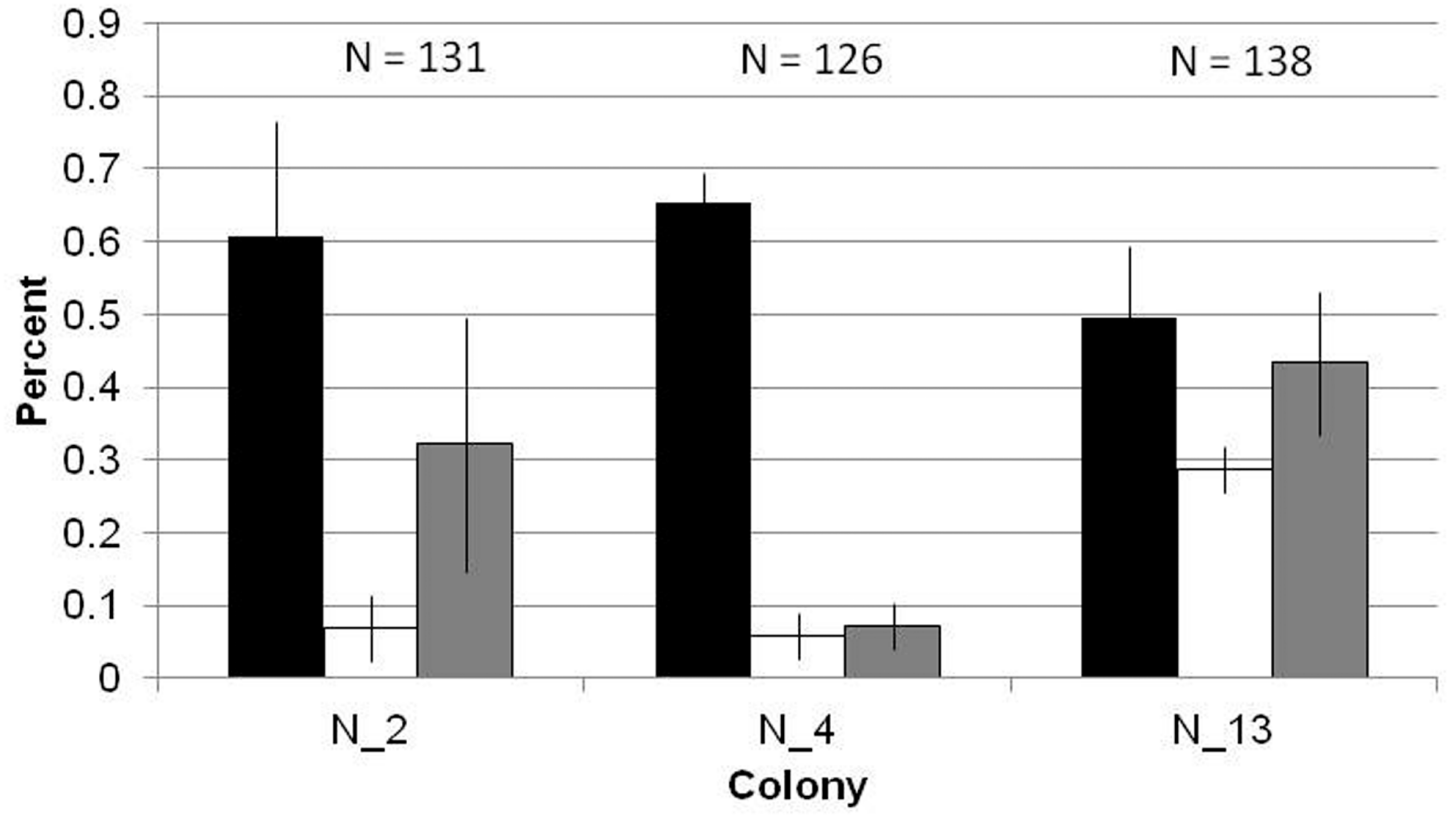
Comparison of activity distribution by colony. Each bar shows the mean proportion of ascending ants that foraged (black), did nest maintenance (white), or ascended into the nest entrance but then descended back into the deeper nest without leaving the nest to forage (grey), over the course of three days. Error bars show standard errors of the mean.

To determine the sample size of outgoing foragers, we considered the possibility that if the proportion of foragers were extremely different in the three colonies observed, a given sample size would not be equally representative in all three colonies. We found that the proportions foraging in the three colonies were not significantly different (ANOVA, F_2,6_ = 3.854; p = 0.08) (Fig 3) (S1 Dataset). Therefore, we decided to measure interaction rates in the same number of foragers and descending ants in all three colonies, rather than choose different sample sizes for each colony.

To determine what percentage of the ants that ascended from the deeper nest into the entrance chamber were outgoing foragers, descending ants, or nest maintenance workers within our 5–7 minutes of film, we observed randomly selected ants in each of the nine films (three per colony for three colonies taken over the course of three days, S1 Table). Every five seconds for the first four minutes of film, the first ant to ascend from the entrance tunnel was tracked until it left the nest, performed nest maintenance by moving dirt or debris around the nest entrance, or descended to the deeper nest. Ants that were lost from view due to glare or an obstruction, and ants that did not perform any of the indicated behavior until the end of the film were excluded from analysis. Out of 147 possible focal ants per colony (pooled over the three days), 16 were excluded from the analysis of N_2, 21 from the analysis of N_4, and 9 from the analysis of N_13 (S1 Dataset).

To record the interactions of outgoing foraging and descending ants, we analyzed the JPEGs from the films using a custom written MATLAB script as in Pinter-Wollman *et al*. [13] (code provided in S1 Appendix). We observed ten outgoing foragers and four or five descending ants per film, the first foraging and descending ants in the film segment that were clearly visible, from when the ant entered the entrance chamber from the deeper nest to when she either left the entrance chamber or descended to the deeper nest. (Five descending ants were selected for each film except for the film of N_2 on August 15th, which showed only four descending ants.) We recorded the time each ant entered and left the entrance chamber, and the time and location of each interaction of the focal ant. Interactions were recorded when the focal ant made antennal contact with the head or body of another ant, and the location of the interaction was defined as the point in between the head of the first ant and the place of contact on the second ant. Our analysis did not examine interactions with other outgoing foragers, because previous work showed that only successful returning foragers stimulated foraging [43]. An outgoing forager was identified as an ant that moved directly toward the outside of the nest and immediately left the nest as soon as it reached the edge of the entrance chamber.

We used a linear multilevel model with normal errors, fit via REML using R’s lme4 package, to determine if the rate of interactions and time spent in the entrance chamber differed between outgoing foragers and descending ants [46]. To account for colony and day effects, ant activity was a fixed effect with colony and day as random effects with normally distributed error terms.

To investigate what regulates the number of outgoing foragers available in the entrance chamber (Question 2), we filmed the entrance chamber in the morning when the ants were foraging and relatively undisturbed for about 6 minutes in 2013 (S1 Table). To test how changes in the rate of forager return influence the rate at which ants ascend into and descend from the entrance chamber, we manipulated forager return rate. Returning foragers were removed for 3–5 minutes (depending on the amount of time needed to collect most of the returning foragers) and kept in a plastic box (and returned to the nest after observations were completed), as in Gordon *et al*. [12]. We observed and filmed behavior inside the nest during and after forager removals. We performed removal experiments in 13 out of the 16 trials (S1 Table).

We counted from the films the numbers of returning and outgoing foragers at the nest entrance and the number of ascending and descending ants at all tunnel entrances. We used MATLAB to test for cross-correlations (p < 0.01) among:

1. rates of returning foragers and ascending ants
2. rates of returning foragers and descending ants
3. rates of ascending ants and descending ants

We tested for cross-correlations in each of the three combinations of variables listed above, to determine whether the two variables tended to be correlated in time, and if so, with what lag. For example, if an ant in the entrance chamber often descended to the deeper nest 10 seconds after a returning forager came in, then there would be a positive correlation at a lag of 10 seconds in the rates of returning foragers and descending ants. To normalize each time series for differences in numbers of ants, we subtracted from each point the moving average of each time series using a time window that was half the duration of each film (S2 Table). The timescale of the smoothing window was minutes (median 7 minutes), whereas observed lags were on the order of seconds (median ~15 seconds), and thus the results were probably not affected by this normalization procedure.

To identify significant cross-correlations, indicating a time dependence for two rates, we calculated the likelihood of a correlation for each possible lag time, relative to the null hypothesis of no time dependence. We report lag times that were statistically significant at p < 0.01. In addition, we ran 1000 simulations in which the time of each event (ant returning, ascending, or descending) was randomly assigned from a uniform distribution covering the average length of the videos. We then compared the proportion of significant cross correlations in the simulated data with that proportion in the observed data. A smaller proportion of significant events in the simulated data compared with the observed indicates that the observed correlations were not found by chance. We next investigated the spatial distribution of interactions (Question 3). Using Java software we developed, we split the films into JPEG frames (at 30 frames per second) and marked the location in the entrance chamber of each interaction of the ants we tracked. The locations of interactions were recorded in two instantaneous images, one at the start of the removal of returning foragers, and the other immediately after forager removals ended 3–5 minutes later.

To illustrate the spatial pattern of interactions, we used a 2D Gaussian kernel density estimator in R package MASS to produce 13 pairs of utilization distribution maps for the 13 trials with forager removals [46]. These maps show the spatial density of interactions in the entrance chamber at the start and at the end of the forager removals.

## Results

### 1. Interaction rates of outgoing foragers

The mean (±SD) time outgoing foragers were tracked was 7.51 (±7.43) seconds (range 0.401 to 36.6) and the mean time descending ants were tracked was 16.0 (±10.8) seconds (range 2.91 to 47.6). Although sampling times varied widely, the distribution was similar across each colony (S1 Fig).

**Fig 4:**
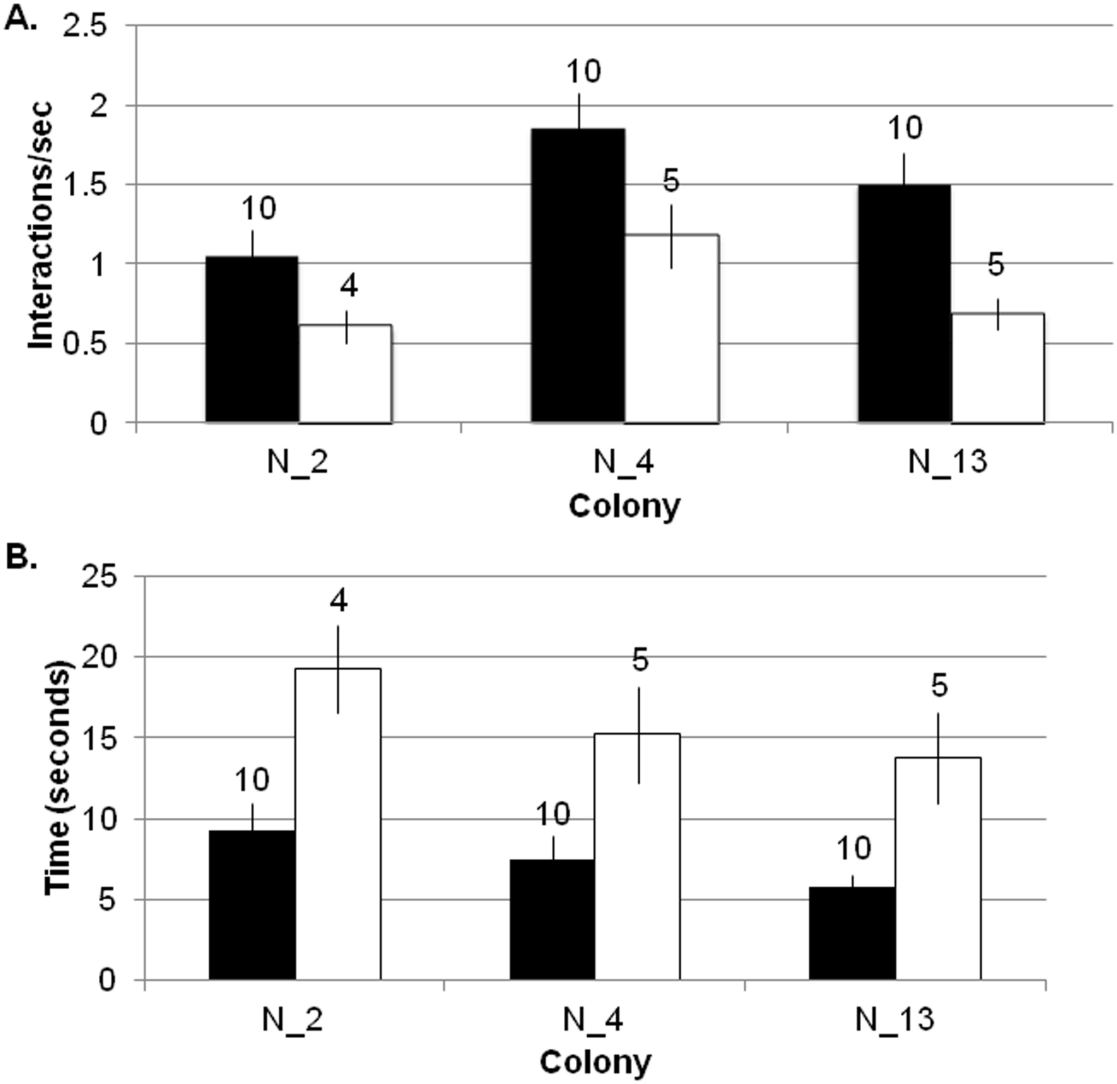
Comparison of interaction rate and time in entrance chamber in outgoing foragers and descending ants. A) Each bar shows the mean rate of brief antennal contacts, in interactions per second, of ants in the entrance chamber. B) Each bar shows the mean time in seconds that ants spent in the entrance chamber. Black: ants that subsequently left the nest to forage (outgoing foragers); White: ants that descended from the entrance chamber to the deeper nest (descending ants). Error bars show standard errors of the mean.

The mean (±SD) interaction rate for outgoing foragers was 1.47 (±1.09) interactions per second, while the mean interaction rate for descending ants was 0.83 (±0.587) interactions per second (S2 Dataset). Taking into account differences in colony- and day-specific interaction rates, the interaction rates of foragers were significantly higher than those of descending ants (linear multilevel model, b = 0.64; 95% CI: 0.33, 0.95; p < 0.001). (The coefficient (b) represents the model-based estimate for the increase in interaction rate between foragers and descending ants.) Foragers also spent a shorter time in the entrance chamber than ants that eventually descended to the deeper nest (linear multilevel model with normal errors, b = -8.55; 95% CI: 11.65, -5.44; p<0.001). For illustrative purposes, interaction rates and time in the entrance chamber by colony (pooling across all three days) are shown in Fig 4.

The three colonies differed in the interaction rates of outgoing foragers (ANOVA, F_2,27_ = 8.42; p = 0.0004) and descending ants (ANOVA, F_2,11_ = 4.83; p = 0.0002), and all differed in pairwise tests except colonies N_2 and N_13 (N_2 vs N_13 Tukey multiple comparison of means, p = 0.622 for outgoing foragers, p = 0.92 for descending). The three days also differed in the interaction rates of outgoing foragers (ANOVA, F_2,27_ = 5.64; p = 0.005) and descending ants (ANOVA, F_2,11_ = 12.21; p <0.0001), due to a difference between August 13 and August 14 (Tukey multiple comparisons of means, p = 0.0033 for outgoing foragers, p < 0.0001 for descending ants).

Within the 5–7 minutes of each of our films, the proportion of ascending ants that left the nest ranged from 0.31–0.89, (mean (±SD) 0.59 (±0.18)) (Fig 3) (S1 Dataset). The proportion of ascending ants that performed nest maintenance, carrying soil or debris out of or around the nest, ranged from 0.00–0.083 (mean (±SD) 0.067 (±0.048)), and the proportion that returned to the deeper nest ranged from 0.056–0.65 (mean (±SD) 0.34 (±0.19)) (Fig 3). In all colonies and days, significantly more ascending ants left the nest (Student’s t-test, p<0.0001) or returned to the deeper nest (Student’s t-test, p = 0.0036) than did nest maintenance. In seven of the nine colonies and days, more ants left the nest than returned to the deeper nest without foraging.

### 2. Availability of foragers in entrance chamber

**Fig 5:**
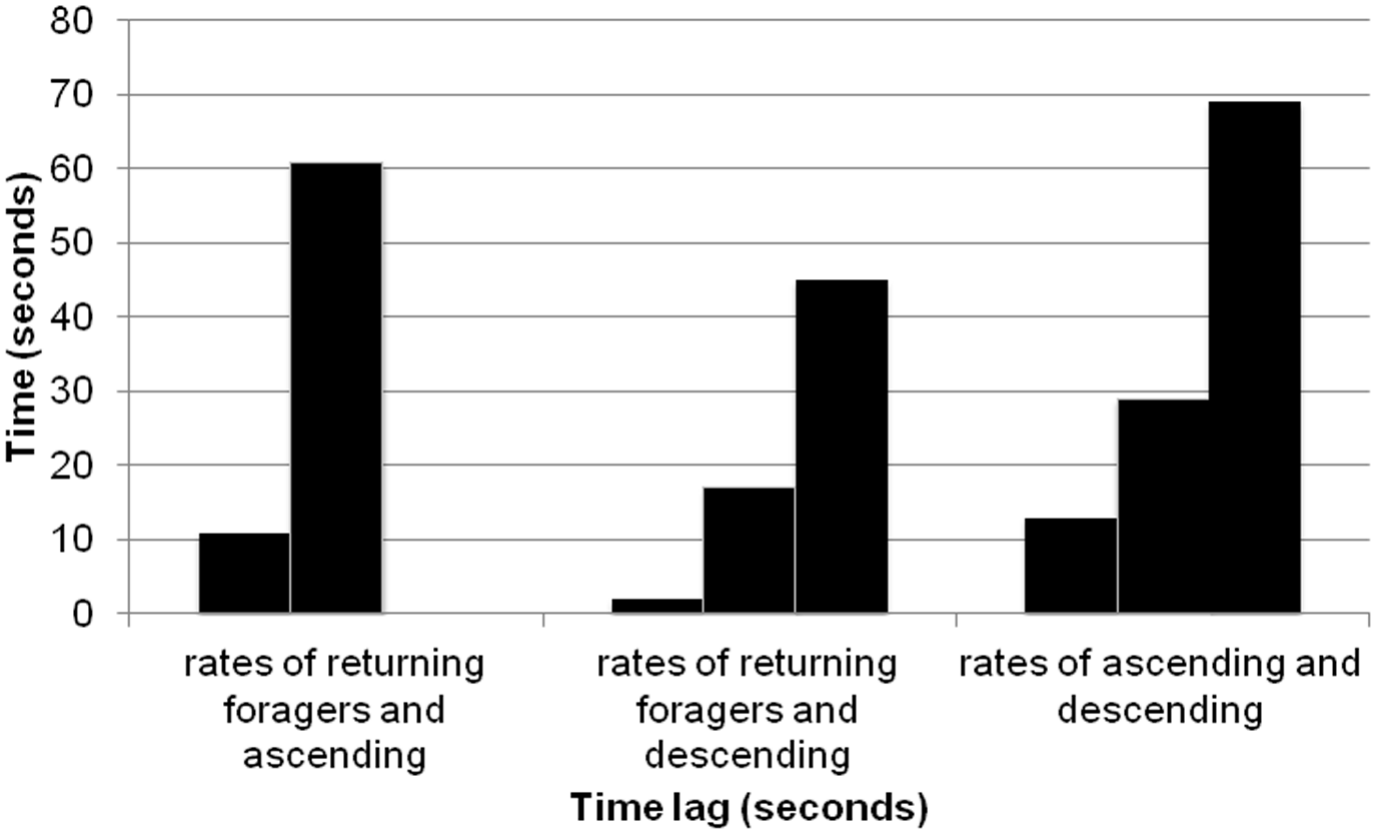
Comparison of significant cross-correlations in lag times for a single colony. All lag times are from colony 367 on August 20, 2013, the only trial that showed significant lag times for all three cross-correlations. Each bar shows the lag of a significant cross-correlation between 1) rate of forager return and rate at which ants ascended to the entrance chamber from the deeper nest, 2) rate of forager return and rate at which ants descended to the deeper nest, and 3) rate at which ants ascended to the entrance chamber and rate at which ants descended to the deeper nest.

There was evidence of time-dependence among the rates at which ants returned to the nest, ascended from the deeper nest to the entrance chamber, and descended from the entrance chamber to the deeper nest (S3 Dataset). We found significant cross-correlations between the rates of returning foragers and ascending ants, returning foragers and descending ants, and ascending ants and descending ants in some of the trials, when removals were performed, for each of the 3 colonies. (Table 1; Cross-correlation, p < 0.01). The cross-correlation between rates of returning foragers and ascending ants was significant in one out of three trials for colony 367 and one out of two trials for colony 229 (2/5 trials) (Table 1). The cross-correlation between rates of returning foragers and descending ants was significant in one out of three trials for colony N_5, two out of three trials for colony 367, one out of one trial for colony 25, and one out of one trials for colony 242 (5/7 trials) (Table 1). The cross-correlation between rates of ascending ants and descending ants was significant in two out of three trials for colony N_5, one out of three trials for colony 367, two out of three trials for colony 868, and one out of two trials for colony 229 (9/11 trials) (Table 1). The simulations in which ascending, descending, and returning ant times were chosen from a uniform distribution produced only 76/1000 significant cross correlations, a smaller proportion than any of the observed cross correlations (0.076 for the randomization simulations compared with 0.4, 0.71, and 0.82 for the observed data). There was one removal trial that produced statistically significant lag times for all three cross-correlations: colony 367 on August 20, 2013. There were two statistically significant lag times found between the returning foragers and ascending ants, three for returning foragers and descending ants, and three for ascending ants and descending ants. Fig 5 shows all the statistically significant cross correlations from this trial.

**Table 1:** Lag times found after removals were performed. The lag values in seconds between returning foragers and ascending ants, returning foragers and descending ants, and ascending ants and descending ants. Mean and standard deviation across all colonies and days also shown. For cross-correlations in which more than one lag time was detected, all significant lags are reported. A “—” indicates that no significant lags were found.

Colony name	Date filmed	Lag times between returning foragers and ascending (seconds)	Lag times between returning foragers and descending ants (seconds)	Lag times between ascending and descending ants (seconds)
N_5	8-17-2013	-	13	-
N_5	8-18-2013	-	-	5
N_5	8-20-2013	-	-	30, 68
367	8-18-2013	-	6, 26	-
367	8-20-2013	11, 61	2, 17, 45	13, 29, 69
367	8-21-2013	-	-	-
868	8-19-2013	-	-	43
868	8-20-2013	-	-	4
868	8-21-2013	-	-	-
25	8-24-2013	-	22	-
229	8-24-2013	-	-	-
229	8-26-2013	58	-	62, 68
242	8-25-2013	-	13	-
Mean		43.33	18	39.1
Standard Deviation		28.04	13.42	26.59

**Fig 6:**
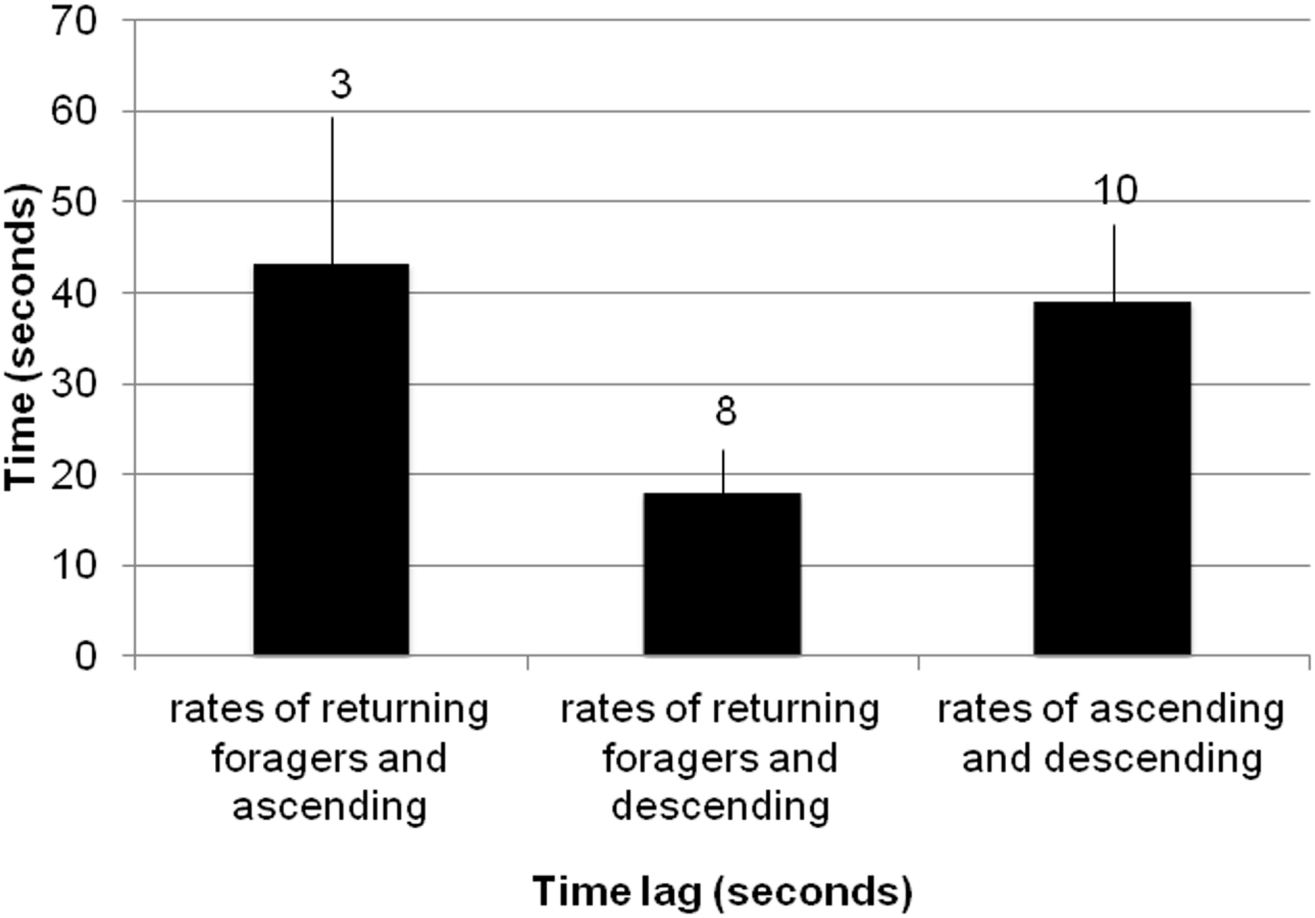
Comparison of time lags between ant activities. Each bar shows the duration in seconds of the lag between two rates, with data pooled across six colonies and eight days: 1) rate of forager return and rate at which ants ascended to the entrance chamber from the deeper nest, 2) rate of forager return and rate at which ants descended to the deeper nest from the entrance chamber, and 3) rate at which ants ascended to the entrance chamber from the deeper nest and rate at which ants descended to the deeper nest from the entrance chamber. The figure shows lag values only for the cross-correlations that were statistically significant. Error bars show standard errors of the mean. Numbers above bars signify sample sizes.

The rate at which ants descended into the deeper nest depended significantly on the rate of returning foragers. Of the three relationships considered, the shortest lag time was between foragers returning and ants descending into the deeper nest. The mean (±SD) lag, 18.00 (±13.42) seconds, between returning foragers and descending ants was significantly shorter than the lag between ascending ants and descending ants, 39.10 (±26.59) seconds (Student’s t-test, p = 0.048) (Fig 6). It was also significantly shorter than the lag between returning foragers and ascending ants, 43.33 (±28.04) seconds (Student’s t-test, p = 0.032) (Fig 6). Some colonies showed significant lag times between more than one of the tested relationships, while other colonies had only one (Table 1).

### 3. Spatial distribution of interactions

**Fig 7:**
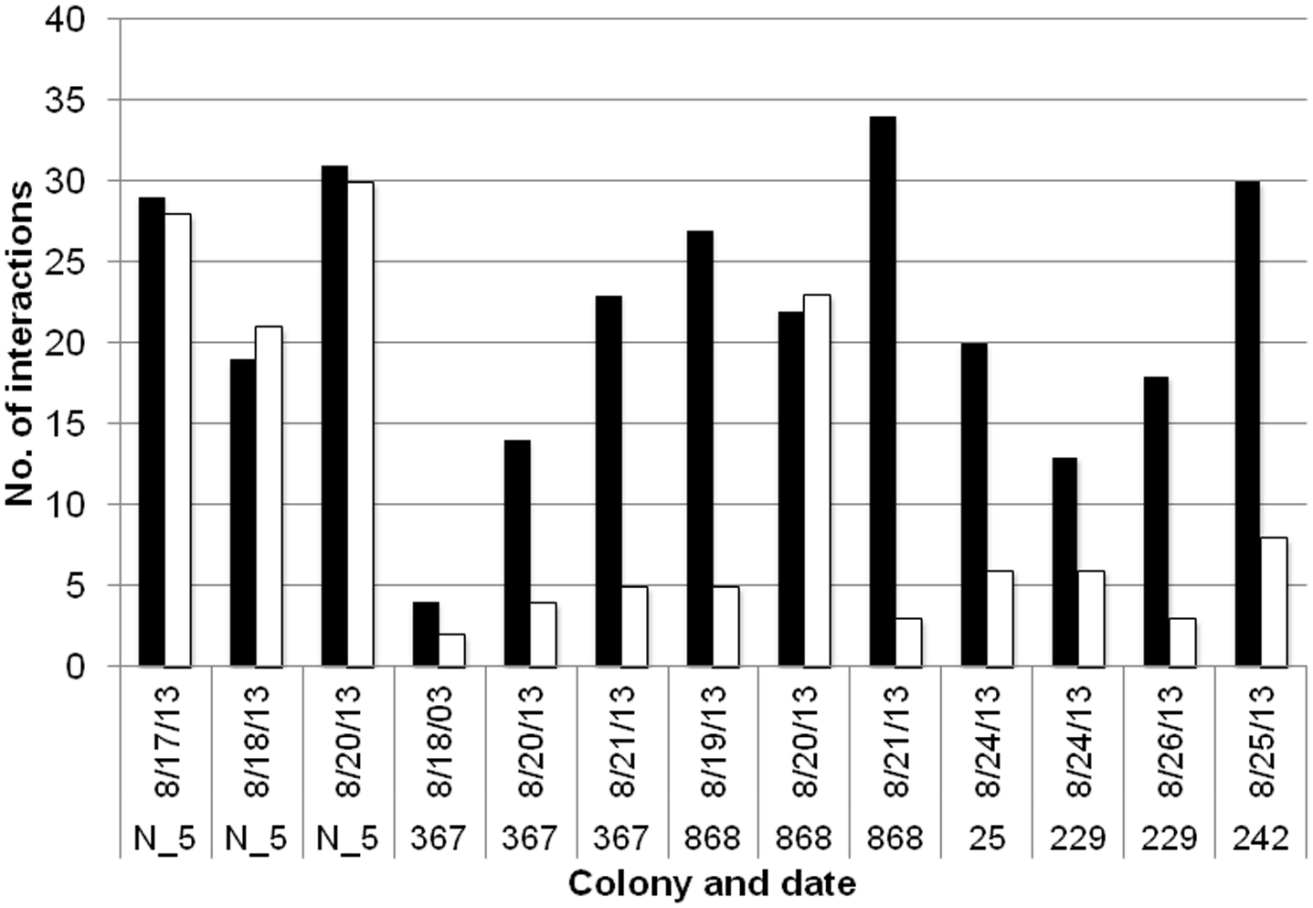
Comparison of number of interactions in the entrance chamber before and after removals. Black: the number of interactions observed from one film frame at the start of forager removals; White: the number of interactions observed from one film frame 3–5 minutes after removal of returning foragers was completed.

**Fig 8:**
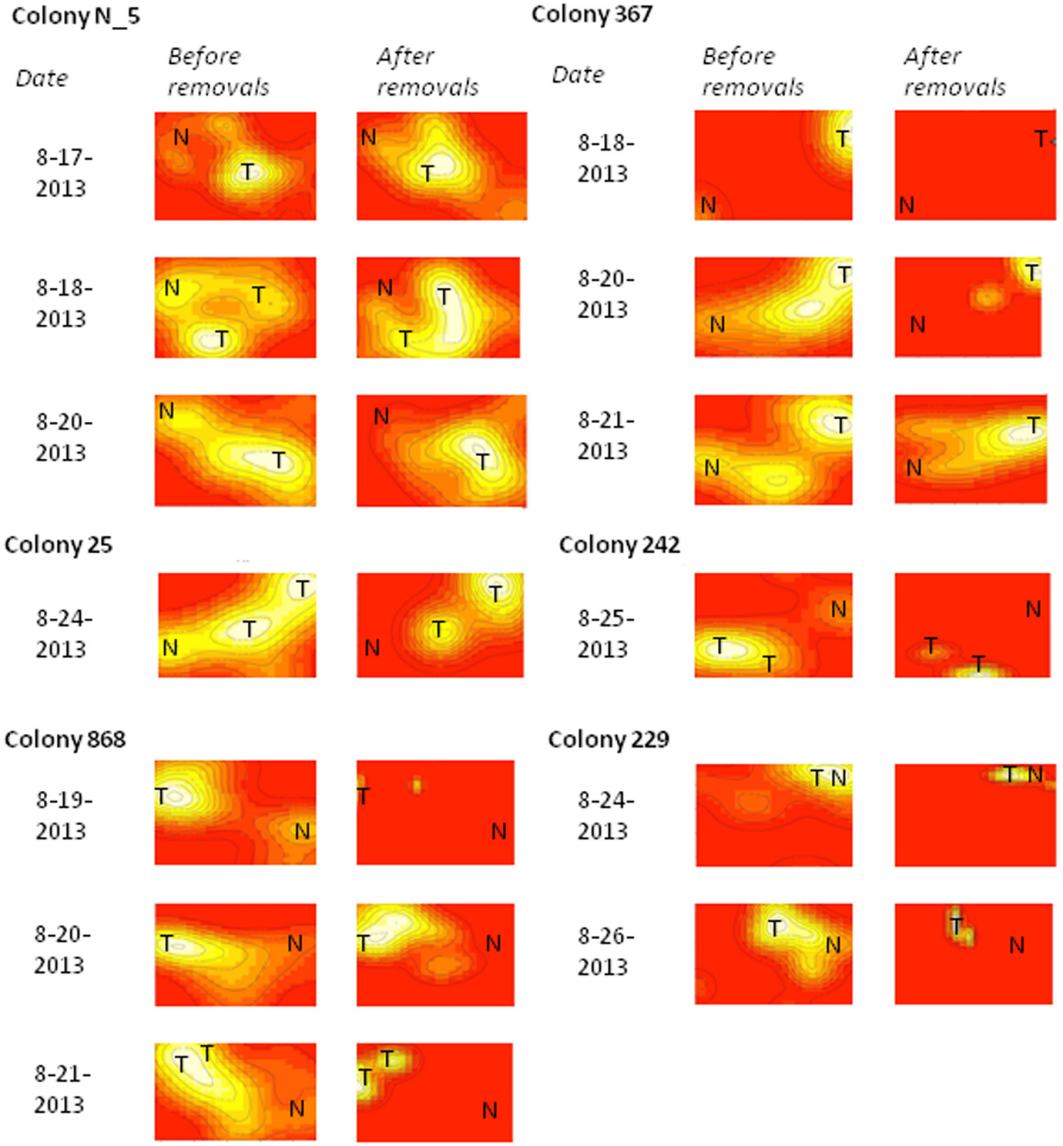
Spatial distribution of interactions. Each interaction utilization distribution map shows the location of interactions, brief antennal contacts between ants, that were observed from instantaneous images. The left figure of each pair shows interactions at the very beginning of an experiment that decreased the rate of incoming foragers, and the right figure shows interactions 3–5 minutes after the experiment was complete. White shows the areas of highest interaction density for each pair of heat maps, yellow shows areas with some interactions, and red shows areas with no interactions. The tunnel entrance/exit is indicated with a T and the nest entrance/exit is indicated with an N. The scales for size and color are relative to each pair of maps.

Several minutes after the rate of returning foragers was experimentally reduced, there were significantly fewer ant interactions in the entrance chamber (Paired t-test, p = 0.003) (Figs 7 and 8) (S4 Dataset, S1 Movie). Considering each of the thirteen films individually, eight films showed significantly fewer interactions after removals than before removals: colony 367 on 8-20-13 and 8-21-13, colony 868 on 8-19-13 and 8-21-13, colony 25 on 8-24-13, colony 229 on 8-24-13 and 8-26-13, and colony 242 on 8-25-13 (Binomial test, p < 0.05) (Fig 7).

The interaction utilization distribution maps show that the highest number of interactions occurs at the entrances to the tunnels to the deeper nest, both before and after removals (Fig 8) (S4 Dataset).

## Discussion

Ants in the entrance chamber that leave the nest to forage have experienced more interactions, in the form of brief antennal contacts, than ants that descend to the deeper nest without foraging. The availability of foragers in the entrance chamber is regulated by another process that determines the flow of ants into and out of the entrance chamber from the deeper nest. This movement of ants in and out of the entrance chamber from the deeper nest, which determines the availability of foragers, is associated with the rate of forager return. The more frequently that returning foragers come in, the more frequently ants descend from the entrance chamber to the deeper nest, and in turn, the more frequently foragers ascend to the entrance chamber from the deeper nest. In this way, higher rates of forager return lead to the presence of more available foragers, as previously observed [13]. Interactions occur at highest density in the nest entrance and the entrances of the tunnels leading to the deeper nest.

Our result supports previous work showing that ants in the entrance chamber determine whether to forage based on the rate of interactions with returning, successful foragers [11,12,32] and showed that the rate of forager return is correlated with the rate of interactions inside the nest [13]. Here we show directly that the ants that left the entrance chamber to forage had experienced a significantly higher rate of interactions (mean 1.47 interactions per sec) than ants that descended from the entrance chamber to the deeper nest without foraging (mean 0.83 interactions per sec). Ants that left the nest to forage also spent significantly less time in the entrance chamber (mean 7.51 sec) than those that descended to the deeper nest (mean 16.03 sec).

Our results show that during the period of foraging activity, the rate of forager return helps to regulate the rate at which foragers become available to forage. Most of the ants in the entrance chamber are foragers: of the ants that came from the deeper nest into the entrance chamber, most left the nest to forage. Almost half the ants that did not forage went back down into the deeper nest (Fig 2).

The regulation of the number of ants in the entrance chamber allows the colony to adjust forager availability quickly in response to changing conditions. When food availability is high, foragers find food quickly, leading to a high rate of forager return [47]. The number of ants in the entrance chamber depends on the rate of forager return [13]. The rate of forager return is linked with a short lag (mean 18 seconds) to the rate at which ants descend to the deeper nest (Table 1). The returning foragers sometimes deposit their food in the entrance chamber for other ants to carry to the lower nest, but in the exposed entrance chambers that we filmed, more often returning foragers descended still carrying the food items they brought back (more discussion below). The rate at which foragers ascend is linked with a significantly longer lag (mean 38.25 seconds) to the rate at which ants descend (Table 1). When the rate of forager return slows, indicating low food availability, the number of available foragers in the entrance chamber also slows [13]. The number of interactions in the entrance chamber nest decreases with low forager return (Figs 7 and 8), further decreasing the probability ants will leave the nest to forage (Fig 4A).

The process that regulates the numbers in the entrance chamber is very rapid; most ants in the entrance chamber either forage or descend in less than 20 seconds. The rapid turnover of ants entering and leaving the entrance chamber may help to limit the number of waiting ants in the entrance chamber [13], who could slow the rate of interactions with incoming, successful foragers.

The different lags between rates of forager return, ascending ants, and descending ants may be due to different processes. For example, the lag times between ascending ants and descending ants were approximately factors of one another in two cases (30 and 68 seconds for colony N_5 on 8-20-13; and 13, 29, and 69 seconds for colony 367 on 8-20-13). This suggests a periodicity in the behavior of ascending into and descending from the entrance chamber. It is interesting to note that the lag between returning foragers and ascending ants tends to be larger than both the lags between returning foragers and descending ants and between ascending and descending ants. This is what we would expect if the same ants return from foraging, descend to the deeper nest, drop off their food, and then ascend back to the entrance chamber to be available to leave on the next foraging trip. More work is needed to determine how often returning foragers carry their food into the deeper nest, or instead deposit it in the entrance chamber for other ants to transport down to the seed chambers in the deeper nest.

A number of factors influence the spatial distribution and frequency of interactions. Harvester ant colonies vary consistently from year to year in foraging activity [12,37]. These variations may arise from colony-specific differences in how interaction rates affect the foraging decisions of ants. The three colonies filmed in 2013 differed in interaction rates, and interaction rates differed between two of the days (August 14^th^ and August 15^th^). Colony differences in interaction rate could be related to variation in nest structure [38,41,42]. Nests vary in the size and shape of the entrance chamber and in the number of tunnels leading from the chamber to the deeper nest (Fig 8). This variation may lead to differences among colonies in the timing of interactions and in the relation between the rate of forager return and of the flow of ants into the entrance chamber. In another species of harvester ant, the rate of recruitment to food increased with the number of tunnels leading from the entrance chamber to the deeper nest [48]. Our heat maps of interaction rate (Fig 8) show that interactions in the entrance chamber tend to occur at the exit to the surface and at the entrances to tunnels leading down to the deeper nest.

## Conclusions

Individual workers in social insect colonies respond to rates of interaction with other workers. Collectively, this allows a colony to regulate its behavior and respond to changing conditions. Here we show that interactions regulate both the activation of foragers and the availability of foragers to be activated. Variation among harvester ant colonies in the regulation of foraging is associated with variation in reproductive success [37]. Investigating the allocation of effort in ant colonies contributes to a fundamental question in biology: how local interactions produce the collective behavior of the whole system.

## Supporting Information

S1 Appendix Matlab script for recording information about ant interactions. This is a Matlab function that opens up a stack of JPEG images and allows a user to move through the images and ‘click’ anywhere on any image to record x-y coordinates of events in the frame. The output is a csv file with 6 columns: xy coordinates of the click, time = frame/image number, ant ID, ant type, and activity—both of which are pre-defined by the user, see below. Copyright (C)2015 Noa Pinter-Wollman.
(PDF)

S1 Dataset 2012 Ant Activity Data. We observed ants in two minutes of each of the nine films to determine what percentage of the ants that ascended from the deeper nest into the entrance chamber were later outgoing foragers, descending ants, or nest maintenance workers. Every five seconds, the first ant to ascend from the entrance tunnel was tracked until it left the nest, performed nest maintenance by moving dirt or debris around the nest entrance, or descended to the deeper nest. This dataset shows the activity type of each of the tracked ants.
(PDF)

S2 Dataset 2012 Ant Interactions Data. We analyzed the JPEGs from the films using a custom written MATLAB script as in Pinter-Wollman *et al*. [13] (code provided in S1 Appendix). We observed ten outgoing foragers and four or five descending ants per film, the first foraging and descending ants in the video segment that were clearly visible. This dataset shows the time each ant entered and left the entrance chamber, and the time and location of each interaction of the focal ants.
(PDF)

S3 Dataset 2013 Correlation Data. We observed and filmed behavior inside the nest during and after forager removals. This dataset shows our counts made from the films of the numbers of returning and outgoing foragers at the nest entrance and the number of ascending and descending ants at all tunnel entrances.
(ZIP)

S4 Dataset 2013 Heat Maps Data. After splitting the 2013 videos into JPEG frames, we marked the location in the entrance chamber of each interaction of the ants we tracked. This dataset shows the locations of interactions from two instantaneous images, one at the start of the removal of returning foragers, and the other immediately after forager removals ended 3–5 minutes later.
(PDF)

S1 Fig Sampling time distribution histograms. These histograms show the distribution of times that foraging and descending ants filmed in 2012 remained in the entrance chamber.
(TIF)

S1 Movie Interactions over time for a single colony. The attached clip shows the location of interactions, in the form of brief antennal contacts between ants, in colony 367 on August 20, 2013. Removal of returning foragers began at 00:37 and ended at 05:41. There is an observable difference between 00:37 seconds and 2:37. By 2:37, as the rate of forager return decreases, the ants are no longer as spread out in the entrance chamber, and interactions occur only at the interaction hotspots (Fig 8), the entrance to tunnels to the deeper nest and the exit from the chamber to outside the nest. The circular metal device is a thermometer and humidity sensor. Some of the ants were painted previously for a different experiment.
(MP4)

S1 Table Red harvester ant colonies filmed in August 2012 and August 2013. (DOCX)

S2 Table Lengths of films taken in 2013 in colonies where removal experiments were performed. (DOCX)
