# Supplementary material for: Interactions Increase Forager Availability and Activity in Harvester Ants
Source: PLoS One. 2015 Nov 5;10(11):e0141971. doi: 10.1371/journal.pone.0141971 (PMC4635008; doi:10.1371/journal.pone.0141971)
Supplement: S1 Appendix — This is a Matlab function that opens up a stack of JPEG images and allows a user to move through the images and ‘click’ anywhere on any image to record x-y coordinates of events in the frame. The output is a csv file with 6 columns: xy coordinates of the click, time = frame/image number, ant ID, ant type, and activity—both of which are pre-defined by the user, see below. Copyright (C)2015 Noa Pinter-Wollman. (PDF) [file pone.0141971.s001.pdf]

```
% This is a Matlab function that opens up a stack of JPEG images and allows a
user to move through the images and 'click' anywhere on any image to record
x-y coordinates of events in the frame. The output is a csv file with 6
columns: xy coordinates of the click, time = frame/image number, ant ID, ant
type, and activity – both of which are pre-defined by the user, see below.
```

Copyright (C)2015 Noa Pinter-Wollman

This program is free software: you can redistribute it and/or modify
it under the terms of the GNU General Public License as published by
the Free Software Foundation, either version 3 of the License, or
(at your option) any later version.

This program is distributed in the hope that it will be useful,
but WITHOUT ANY WARRANTY; without even the implied warranty of
MERCHANTABILITY or FITNESS FOR A PARTICULAR PURPOSE. See the
GNU General Public License for more details.
See <http://www.gnu.org/licenses/> for a copy of the GNU General Public
License

```
function ClickGUInobuttons
%%%%%%%%%%%%%%%%%%%%%%%%%%%%%%%%%%%%%%%%%%%%%%%%%%%%%%%%%%%%%%%%%%%%%%
% do not try to create more ants if the csv output file is open!

% the output of the program will be in indexes to this cell array
% what matters is the order:
% define ant types to code (e.g. foragers (F), nest maintenance (NM) etc…
must use these exact types as defined here to initiate an ant
AntTypes={'F','NM'}; % these are the possible ant types to input – you can
add as many as you'd like

% the following are the types of clicks (Ant activities):
% interaction with standing ant(left mouse click), interaction with returning
ant (right mouse click), interaction with leaving ant (type the letter 'l'
when the mouse is at the location of the click), exit nest (type letter 'w'),
etc…
SpecialClicks={char(1),char(3),'l','w'}; %you can add as many as you'd like

%%%%%%%%%%%%%%%%%%%%%%%%%%%%%%%%%%%%%%%%%%%%%%%%%%%%%%%%%%%%%%%%%%%%%%

%% FUNCTION KEYs – when the GUI is open, these keys will initiate various
operations:
CFG.change='a'; % change ant id and type (type the letter 'a')
CFG.forward=char(29); % right arrow moves frame forward
CFG.back=char(28); %left arrow moves frame back
CFG.jump='j'; % jump to frame # (type letter 'j')
CFG.quit='q'; % quit program and save csv file (type letter 'q')
CFG.undo='u'; % undo last click (type letter 'u')

%%%%%%%%%% OUTPUT FORMAT %%%%%%%%%%%%%%%%%%%%%%%%%%%%
% csv file saved in foder with images, named 'interactions - 'colonyID' '
```

```
% column order: ant id, x, y, frame from file name, click type, ant type
%%%%%%%%%%%%%%%%%%%%%%%%%%%%%%%%%%%%%%%%%%%%%%%%%%%%%%%%%%%%%%%%%%%%%%%%%

H={'ant id', 'x', 'y', 'frame from file name', 'click type', 'ant type'};
%header for csv file
%% get path info
% using the GUI
pth = uigetdir(pwd,'browse to folder with jpgs to analyze') ;

ext='jpg';
lst=dir(fullfile(pth,['*.' ext]));

N=length(lst);

%% parse T from file names - get the frame number from the file name of the
jpeg - might need to adjust this portion based on your naming convention
T=zeros(N,1);
for i=1:N
    [bla, filename]=fileparts(lst(i).name);
    [colonyname,frameAsStr]=strtok(filename,'_');
    frameAsStr=frameAsStr(2:end);
    T(i)=str2double(frameAsStr);
end

currentAntID=nan;
currentFrame=1;
currentAntType=nan;
img=[];
x=[];
y=[];
b=[];

quit=false;

clr=hsv(10000);
clr=clr(randperm(10000),:);

% read existing click from that folder
if exist(fullfile(pth,['interactions-' colonyname '.csv']),'file')
    IM=importdata(fullfile(pth,['interactions-' colonyname '.csv']),',',1);
    M=IM.data;
else
    M=[];
end

%%
figure(1)
clf
hImg=subplot('position',[0 0.1 1 0.9]);
% these are the play/rewind buttons. can adjust size and location here
set(1,'name','')
readImg;
showImg;

while ~quit
```

```
    try % to get click from user
        [x,y,b]=ginput(1);

        % parse the click
        parseClick;

        % show image
        showImg;
    catch e
        button = questdlg(e.message,'An error has
occured:','Continue','Quit','Continue');
        if strcmp(button,'Quit')
            quit=true;
        end
    end
end

saveOutput; % save on exit
delete(1); % close figure

%% Nested functions

    function parseClick % this is what the function keys do
        switch char(b)
            case CFG.change
                changeAnt;
            case CFG.forward
                currentFrame=min(N,currentFrame+1);
                readImg;
            case CFG.back
                currentFrame=max(1,currentFrame-1);
                readImg;
            case CFG.quit
                quit=true;
            case CFG.undo
                M=M(1:end-1,:);
            case CFG.jump
                msg=sprintf('Jump to frame number: (1 - %d)',N);
                str=inputdlg(msg);
                % if user canceled (and str is empty) return without doing
                % anything
                if isempty(str)
                    return % exit parseClick subfunction to main function
                end
                oldfrm=currentFrame;
                currentFrame=str2double(str);
                if currentFrame>N || currentFrame<1
                    uiwait(warndlg('out of bound frame - ignoring'));
                    currentFrame=oldfrm;
                end
            otherwise
                currentClick=find(ismember(SpecialClicks,char(b)));
                if isempty(currentClick)
                    uiwait(warndlg('Illigal key pressed - nothing will be
recorded!'));
                else
```

```
                    if ~isnan(currentAntID)
                        M=[M; [currentAntID x y T(currentFrame) currentClick
currentAntType]];
                    end
                end
        end
    end

    function changeAnt
        prompt={'Change ant to:','Type:'};
        dlg_title='Enter ant number and type:';
        if ~isempty(M)
            nxtant=max(M(:,1))+1;
        else
            nxtant=1;
        end
        defAns={num2str(nxtant),''};
        str = inputdlg(prompt,dlg_title,1,defAns);
        % if user cancels return (stays with current ant and type
        if isempty(str),
            return, % exit from function changeAnt into outer function
        end
        currentAntID=str2double(str{1});
        currentAntType=find(ismember(AntTypes,str{2}));
        if isempty(currentAntType)
            uiwait(warndlg('WRONG ANT TYPE - chose change ant again and enter
valid ant type!'))
        end
        showImg;
        saveOutput
    end

    function readImg
         img=imread(fullfile(pth,lst(currentFrame).name));
    end

    function saveOutput
        try
            if ~isempty(M)
                % csvwrite(fullfile(pth,['interactions-' colonyname
'.csv']),M);
                WriteCSVwithHeader(M,H,fullfile(pth,['interactions-'
colonyname '.csv']))
            end
        catch %#ok<CTCH>
            uiwait(warndlg('Could not save  - maybe the file is still open?
Check and retry'))
        end
    end

    function showImg
        %%
        figure(1)
        axes(hImg); %#ok<MAXES>
        cla
        imshow(img,'Border','Tight')
```

```
        if isnan(currentAntID)
            set(1,'name',sprintf('Frame: %d NO ANT
SELECTED!!!',currentFrame))
        else
            set(1,'name',sprintf('Frame: %d, Current ant id: %d, type:
%s',currentFrame,currentAntID,AntTypes{currentAntType}));
        end
%set markers and colors to display each click
        mrkr=['o','.','d','+','x','v'];
        clr2=['c','y','m','k'];
        if ~isempty(M)
            McrntFrame=M(M(:,4)==T(currentFrame),:);
            hold on
            antsInCurrentFrame=unique(McrntFrame(:,1));
            for ant_i=1:length(antsInCurrentFrame)
                ix=find(McrntFrame(:,1)==antsInCurrentFrame(ant_i));
                if ~isempty(ix)

plot(McrntFrame(ix,2),McrntFrame(ix,3),mrkr(McrntFrame(ix,5)),'color',clr2(Mc
rntFrame(ix,6)))% clr(antsInCurrentFrame(ant_i),:)
                    for p=1:length(ix)

text(McrntFrame(ix(p),2)+10,McrntFrame(ix(p),3),num2str(antsInCurrentFrame(an
t_i)),'color',clr(antsInCurrentFrame(ant_i),:), 'FontSize', 7)
%clr(antsInCurrentFrame(ant_i),:)
                    end
                end
            end
        end

        drawnow;
    end


end % main function

function WriteCSVwithHeader(M,H,filename)
% M - matrix OR cell array
% H - Header - cell array
% filename - filename

fid=fopen(filename,'w');
if fid<=0
    error('Problem opening file - please check filename');
end


%% write header
Cn=length(H);
for i=1:Cn-1
    fprintf(fid,'%s,',H{i});
end
fprintf(fid,'%s\n',H{end});

%% write M (either number of text)
```

```
if iscell(M)
    prt='%s';
else
    prt='%f';
    M=num2cell(M);
end

for i=1:size(M,1)
    for j=1:size(M,2)-1
        fprintf(fid,[prt ','],M{i,j});
    end
    fprintf(fid,[prt '\n'],M{i,end});
end

%% close file
fclose(fid);
end
```
