# Supplementary material for: Interactions Increase Forager Availability and Activity in Harvester Ants
Source: PLoS One. 2015 Nov 5;10(11):e0141971. doi: 10.1371/journal.pone.0141971 (PMC4635008; doi:10.1371/journal.pone.0141971)
Supplement: S1 Dataset — We observed ants in two minutes of each of the nine films to determine what percentage of the ants that ascended from the deeper nest into the entrance chamber were later outgoing foragers, descending ants, or nest maintenance workers. Every five seconds, the first ant to ascend from the entrance tunnel was tracked until it left the nest, performed nest maintenance by moving dirt or debris around the nest entrance, or descended to the deeper nest. This dataset shows the activity type of each of the tracked ants. (PDF) [file pone.0141971.s002.pdf]

2012 Ant Activity Data
Researcher
Evlyn Pless

Ant type key

A blank in the "Ant Type" column means no ant entered the entrance chamber within 5 seconds of that video time
1 = outgoing forager
2 = ant leaves range of the camera but does not leave entrance chamber (in some cases, we did not film the entire entrance chamber)
3 = nest maintanenence worker
4 = ant does not leave entrance chamber (or briefly leaves and returns without foraging)
5 = descending ant

Ant number	Colony	Date of video	Video time	Ant type
101	N2	8/13/12	:30	2
102	N2	8/13/12	:35	1
103	N2	8/13/12	:40	1
104	N2	8/13/12	:45	1
105	N2	8/13/12	:50	2
106	N2	8/13/12	:55	5
107	N2	8/13/12	1:00	1
108	N2	8/13/12	1:05	1
109	N2	8/13/12	1:10	2
110	N2	8/13/12	1:15	1
111	N2	8/13/12	1:20	1
112	N2	8/13/12	1:25	1
113	N2	8/13/12	1:30	2
114	N2	8/13/12	1:35	1
115	N2	8/13/12	1:40	1
116	N2	8/13/12	1:45	1
117	N2	8/13/12	1:50	2
118	N2	8/13/12	1:55	1
119	N2	8/13/12	2:00	1
120	N2	8/13/12	2:05	1
121	N2	8/13/12	2:10	2
122	N2	8/13/12	2:15	2
123	N2	8/13/12	2:20	1
124	N2	8/13/12	2:25	5
125	N2	8/13/12	2:30	1

126	N2	8/13/12	2:35	1
127	N2	8/13/12	2:40	1
128	N2	8/13/12	2:45	2
129	N2	8/13/12	2:50	1
130	N2	8/13/12	2:55	2
131	N2	8/13/12	3:00	1
132	N2	8/13/12	3:05	2
133	N2	8/13/12	3:10	1
134	N2	8/13/12	3:15	1
135	N2	8/13/12	3:20	1
136	N2	8/13/12	3:25	2
137	N2	8/13/12	3:30	3
138	N2	8/13/12	3:35	1
139	N2	8/13/12	3:40	1
140	N2	8/13/12	3:45	2
141	N2	8/13/12	3:50	1
142	N2	8/13/12	3:55	1
143	N2	8/13/12	4:00	1
144	N2	8/13/12	4:05	3
145	N2	8/13/12	4:10	1
146	N2	8/13/12	4:15	1
147	N2	8/13/12	4:20	2
148	N2	8/13/12	4:25	1
149	N2	8/13/12	4:30	1
101	N2	8/14/12	:50	5
102	N2	8/14/12	:55	1
103	N2	8/14/12	1:00	
104	N2	8/14/12	1:05	
105	N2	8/14/12	1:10	
106	N2	8/14/12	1:15	
107	N2	8/14/12	1:20	
108	N2	8/14/12	1:25	
109	N2	8/14/12	1:30	5
110	N2	8/14/12	1:35	
111	N2	8/14/12	1:40	
112	N2	8/14/12	1:45	
113	N2	8/14/12	1:50	
114	N2	8/14/12	1:55	
115	N2	8/14/12	2:00	
116	N2	8/14/12	2:05	
117	N2	8/14/12	2:10	

118	N2	8/14/12	2:15	
119	N2	8/14/12	2:20	
120	N2	8/14/12	2:25	5
121	N2	8/14/12	2:30	
122	N2	8/14/12	2:35	5
123	N2	8/14/12	2:40	5
124	N2	8/14/12	2:45	5
125	N2	8/14/12	2:50	
126	N2	8/14/12	2:55	
127	N2	8/14/12	3:00	5
128	N2	8/14/12	3:05	5
129	N2	8/14/12	3:10	
130	N2	8/14/12	3:15	
131	N2	8/14/12	3:20	
132	N2	8/14/12	3:25	
133	N2	8/14/12	3:30	5
134	N2	8/14/12	3:35	1
135	N2	8/14/12	3:40	1
136	N2	8/14/12	3:45	
137	N2	8/14/12	3:50	5
138	N2	8/14/12	3:55	5
139	N2	8/14/12	4:00	
140	N2	8/14/12	4:05	
141	N2	8/14/12	4:10	5
142	N2	8/14/12	4:15	1
143	N2	8/14/12	4:20	1
144	N2	8/14/12	4:25	1
145	N2	8/14/12	4:30	
146	N2	8/14/12	4:35	
147	N2	8/14/12	4:40	5
148	N2	8/14/12	4:45	1
149	N2	8/14/12	4:50	
101	N2	8/15/12	:35	1
102	N2	8/15/12	:40	1
103	N2	8/15/12	:45	1
104	N2	8/15/12	1:00	1
105	N2	8/15/12	1:05	1
106	N2	8/15/12	1:10	3
107	N2	8/15/12	1:15	1
108	N2	8/15/12	1:20	1
109	N2	8/15/12	1:25	5

110	N2	8/15/12	1:30	1
111	N2	8/15/12	1:35	5
112	N2	8/15/12	1:40	5
113	N2	8/15/12	1:45	1
114	N2	8/15/12	1:50	1
115	N2	8/15/12	1:55	1
116	N2	8/15/12	2:00	5
117	N2	8/15/12	2:05	3
118	N2	8/15/12	2:10	5
119	N2	8/15/12	2:15	5
120	N2	8/15/12	2:20	1
121	N2	8/15/12	2:25	1
122	N2	8/15/12	2:30	3
123	N2	8/15/12	2:35	1
124	N2	8/15/12	2:40	1
125	N2	8/15/12	2:45	1
126	N2	8/15/12	2:50	1
127	N2	8/15/12	2:55	5
128	N2	8/15/12	3:00	1
129	N2	8/15/12	3:05	5
130	N2	8/15/12	3:10	5
131	N2	8/15/12	3:15	1
132	N2	8/15/12	3:20	1
133	N2	8/15/12	3:25	5
134	N2	8/15/12	3:30	1
135	N2	8/15/12	3:35	5
136	N2	8/15/12	3:40	1
137	N2	8/15/12	3:45	5
138	N2	8/15/12	3:50	1
139	N2	8/15/12	3:55	4
140	N2	8/15/12	4:00	1
141	N2	8/15/12	4:05	3
142	N2	8/15/12	4:10	3
143	N2	8/15/12	4:15	3
144	N2	8/15/12	4:20	1
145	N2	8/15/12	4:25	3
146	N2	8/15/12	4:30	1
147	N2	8/15/12	4:35	1
148	N2	8/15/12	4:40	4
149	N2	8/15/12	4:45	4

101 N4 8/13/12 :35 1

102	N4	8/13/12 :40		3
103	N4	8/13/12 :45		5
104	N4	8/13/12	1:00	1
105	N4	8/13/12	1:05	1
106	N4	8/13/12	1:10	5
107	N4	8/13/12	1:15	5
108	N4	8/13/12	1:20	2
109	N4	8/13/12	1:25	1
110	N4	8/13/12	1:30	1
111	N4	8/13/12	1:35	3
112	N4	8/13/12	1:40	1
113	N4	8/13/12	1:45	1
114	N4	8/13/12	1:50	1
115	N4	8/13/12	1:55	5
116	N4	8/13/12	2:00	2
117	N4	8/13/12	2:05	5
118	N4	8/13/12	2:10	5
119	N4	8/13/12	2:15	1
120	N4	8/13/12	2:20	5
121	N4	8/13/12	2:25	1
122	N4	8/13/12	2:30	2
123	N4	8/13/12	2:35	5
124	N4	8/13/12	2:40	1
125	N4	8/13/12	2:45	3
126	N4	8/13/12	2:50	5
127	N4	8/13/12	2:55	4
128	N4	8/13/12	3:00	1
129	N4	8/13/12	3:05	1
130	N4	8/13/12	3:10	1
131	N4	8/13/12	3:15	1
132	N4	8/13/12	3:20	1
133	N4	8/13/12	3:25	1
134	N4	8/13/12	3:30	1
135	N4	8/13/12	3:35	1
136	N4	8/13/12	3:40	1
137	N4	8/13/12	3:45	3
138	N4	8/13/12	3:50	5
139	N4	8/13/12	3:55	1
140	N4	8/13/12	4:00	5
141	N4	8/13/12	4:05	1
142	N4	8/13/12	4:10	1
143	N4	8/13/12	4:15	2

144	N4	8/13/12		4:20	2
145	N4	8/13/12		4:25	2
146	N4	8/13/12		4:30	2
147	N4	8/13/12		4:35	1
148	N4	8/13/12	:50		1
149	N4	8/13/12	:55		3
101	N4	8/14/13	:35		1
102	N4	8/14/13	:40		2
103	N4	8/14/13	:45		5
	N4	8/14/13	:50		1
	N4	8/14/13	:55		5
104	N4	8/14/13		1:00	5
105	N4	8/14/13		1:05	2
106	N4	8/14/13		1:10	1
107	N4	8/14/13		1:15	2
108	N4	8/14/13		1:20	5
109	N4	8/14/13		1:25	1
110	N4	8/14/13		1:30	1
111	N4	8/14/13		1:35	1
112	N4	8/14/13		1:40	1
113	N4	8/14/13		1:45	1
114	N4	8/14/13		1:50	2
115	N4	8/14/13		1:55	1
116	N4	8/14/13		2:00	5
117	N4	8/14/13		2:05	3
118	N4	8/14/13		2:10	
119	N4	8/14/13		2:15	
120	N4	8/14/13		2:20	
121	N4	8/14/13		2:25	
122	N4	8/14/13		2:30	
123	N4	8/14/13		2:35	
124	N4	8/14/13		2:40	
125	N4	8/14/13		2:45	
126	N4	8/14/13		2:50	
127	N4	8/14/13		2:55	
128	N4	8/14/13		3:00	1
129	N4	8/14/13		3:05	
130	N4	8/14/13		3:10	2
131	N4	8/14/13		3:15	2
132	N4	8/14/13		3:20	5
133	N4	8/14/13		3:25	1

134	N4	8/14/13	3:30	1
135	N4	8/14/13	3:35	5
136	N4	8/14/13	3:40	2
137	N4	8/14/13	3:45	2
138	N4	8/14/13	3:50	5
139	N4	8/14/13	3:55	5
140	N4	8/14/13	4:00	5
141	N4	8/14/13	4:05	1
142	N4	8/14/13	4:10	2
143	N4	8/14/13	4:15	1
144	N4	8/14/13	4:20	2
145	N4	8/14/13	4:25	1
146	N4	8/14/13	4:30	1
147	N4	8/14/13	4:35	1

101	N4	8/15/12 :35		1
102	N4	8/15/12 :40		1
103	N4	8/15/12 :45		5
104	N4	8/15/12 :50		1
105	N4	8/15/12 :55		1
106	N4	8/15/12	1:00	5
107	N4	8/15/12	1:05	1
108	N4	8/15/12	1:10	1
109	N4	8/15/12	1:15	1
110	N4	8/15/12	1:20	1
111	N4	8/15/12	1:25	5
112	N4	8/15/12	1:30	1
113	N4	8/15/12	1:35	1
114	N4	8/15/12	1:40	1
115	N4	8/15/12	1:45	5
116	N4	8/15/12	1:50	1
117	N4	8/15/12	1:55	5
118	N4	8/15/12	2:00	1
119	N4	8/15/12	2:05	1
120	N4	8/15/12	2:10	5
121	N4	8/15/12	2:15	1
122	N4	8/15/12	2:20	1
123	N4	8/15/12	2:25	1
124	N4	8/15/12	2:30	1
125	N4	8/15/12	2:35	1
126	N4	8/15/12	2:40	1
127	N4	8/15/12	2:45	1

128	N4		8/15/12	2:50	1
129	N4		8/15/12	2:55	5
130	N4		8/15/12	3:00	5
131	N4		8/15/12	3:05	1
132	N4		8/15/12	3:10	5
133	N4		8/15/12	3:15	1
134	N4		8/15/12	3:20	1
135	N4		8/15/12	3:25	1
136	N4		8/15/12	3:30	1
137	N4		8/15/12	3:35	1
138	N4		8/15/12	3:40	1
139	N4		8/15/12	3:45	1
140	N4		8/15/12	3:50	5
141	N4		8/15/12	3:55	1
142	N4		8/15/12	4:00	1
143	N4		8/15/12	4:05	3
144	N4		8/15/12	4:10	1
145	N4		8/15/12	4:15	1
146	N4		8/15/12	4:20	1
147	N4		8/15/12	4:25	5
148	N4		8/15/12	4:30	5
149	N4		8/15/12	4:35	1
101		N13	8/13/12	:30	5
102		N13	8/13/12	:35	5
103		N13	8/13/12	:40	1
104		N13	8/13/12	:45	2
105		N13	8/13/12	:50	2
106		N13	8/13/12	:55	2
107		N13	8/13/12	1:00	1
108		N13	8/13/12	1:05	1
109		N13	8/13/12	1:10	1
110		N13	8/13/12	1:15	1
111		N13	8/13/12	1:20	1
112		N13	8/13/12	1:25	5
113		N13	8/13/12	1:30	3
114		N13	8/13/12	1:35	5
115		N13	8/13/12	1:40	2
116		N13	8/13/12	1:45	5
117		N13	8/13/12	1:50	1
118		N13	8/13/12	1:55	2
119		N13	8/13/12	2:00	5

120	N13	8/13/12	2:05	5
121	N13	8/13/12	2:10	2
122	N13	8/13/12	2:15	5
123	N13	8/13/12	2:20	5
124	N13	8/13/12	2:25	5
125	N13	8/13/12	2:30	5
126	N13	8/13/12	2:35	5
127	N13	8/13/12	2:40	5
128	N13	8/13/12	2:45	5
129	N13	8/13/12	2:50	5
130	N13	8/13/12	2:55	5
131	N13	8/13/12	3:00	5
132	N13	8/13/12	3:05	1
133	N13	8/13/12	3:10	5
134	N13	8/13/12	3:15	5
135	N13	8/13/12	3:20	1
136	N13	8/13/12	3:25	3
137	N13	8/13/12	3:30	3
138	N13	8/13/12	3:35	5
139	N13	8/13/12	3:40	5
140	N13	8/13/12	3:45	1
141	N13	8/13/12	3:50	1
142	N13	8/13/12	3:55	5
143	N13	8/13/12	4:00	2
144	N13	8/13/12	4:05	1
145	N13	8/13/12	4:10	5
146	N13	8/13/12	4:15	5
147	N13	8/13/12	4:20	5
148	N13	8/13/12	4:25	1
149	N13	8/13/12	4:30	5
101	N13	8/14/12	:30	1
103	N13	8/14/12	:40	1
106	N13	8/14/12	:55	1
108	N13	8/14/12	1:05	1
109	N13	8/14/12	1:10	1
110	N13	8/14/12	1:15	1
111	N13	8/14/12	1:25	1
112	N13	8/14/12	1:30	1
113	N13	8/14/12	1:40	1
114	N13	8/14/12	1:45	1
115	N13	8/14/12	1:50	1

116	N13	8/14/12	2:00	1
117	N13	8/14/12	2:05	1
118	N13	8/14/12	2:10	1
119	N13	8/14/12	2:15	1
120	N13	8/14/12	2:30	1
121	N13	8/14/12	2:35	1
122	N13	8/14/12	2:45	1
123	N13	8/14/12	2:50	1
124	N13	8/14/12	2:55	1
125	N13	8/14/12	3:05	1
126	N13	8/14/12	3:10	1
127	N13	8/14/12	3:25	1
128	N13	8/14/12	3:35	1
129	N13	8/14/12	3:40	1
130	N13	8/14/12	3:45	1
131	N13	8/14/12	3:50	1
132	N13	8/14/12	3:55	1
133	N13	8/14/12	4:00	1
134	N13	8/14/12	4:10	1
135	N13	8/14/12	4:15	1
149	N13	8/14/12	4:30	1
150	N13	8/14/12	2:25	3
151	N13	8/14/12	3:30	3
152	N13	8/14/12	4:20	3
102	N13	8/14/12	:35	5
104	N13	8/14/12	:45	5
105	N13	8/14/12	:50	5
107	N13	8/14/12	1:00	5
108	N13	8/14/12	1:20	5
109	N13	8/14/12	1:35	5
110	N13	8/14/12	1:55	5
111	N13	8/14/12	2:20	5
112	N13	8/14/12	2:40	5
113	N13	8/14/12	3:00	5
114	N13	8/14/12	3:15	5
115	N13	8/14/12	3:20	5
116	N13	8/14/12	4:05	5
117	N13	8/14/12	4:25	5
101	N13	8/15/12	:30	1
102	N13	8/15/12	:35	5
103	N13	8/15/12	:40	1

104	N13	8/15/12	:45	5
105	N13	8/15/12	:50	1
106	N13	8/15/12	:55	1
107	N13	8/15/12	1:00	1
108	N13	8/15/12	1:05	1
109	N13	8/15/12	1:10	5
110	N13	8/15/12	1:15	5
111	N13	8/15/12	1:20	
112	N13	8/15/12	1:25	1
113	N13	8/15/12	1:30	5
114	N13	8/15/12	1:35	
115	N13	8/15/12	1:40	5
116	N13	8/15/12	1:45	1
117	N13	8/15/12	1:50	5
118	N13	8/15/12	1:55	1
119	N13	8/15/12	2:00	1
120	N13	8/15/12	2:05	1
121	N13	8/15/12	2:10	5
122	N13	8/15/12	2:15	1
123	N13	8/15/12	2:20	1
124	N13	8/15/12	2:25	4
125	N13	8/15/12	2:30	1
126	N13	8/15/12	2:35	5
127	N13	8/15/12	2:40	1
128	N13	8/15/12	2:45	5
129	N13	8/15/12	2:50	5
130	N13	8/15/12	2:55	5
131	N13	8/15/12	3:00	5
132	N13	8/15/12	3:05	1
133	N13	8/15/12	3:10	1
134	N13	8/15/12	3:15	5
135	N13	8/15/12	3:20	1
136	N13	8/15/12	3:25	5
137	N13	8/15/12	3:30	1
138	N13	8/15/12	3:35	1
139	N13	8/15/12	3:40	1
140	N13	8/15/12	3:45	3
141	N13	8/15/12	3:50	5
142	N13	8/15/12	3:55	5
143	N13	8/15/12	4:00	1
144	N13	8/15/12	4:05	1
145	N13	8/15/12	4:10	1

146	N13	8/15/12	4:15	5
147	N13	8/15/12	4:20	3
148	N13	8/15/12	4:25	3
149	N13	8/15/12	4:30	5
