# Supplementary material for: Interactions Increase Forager Availability and Activity in Harvester Ants
Source: PLoS One. 2015 Nov 5;10(11):e0141971. doi: 10.1371/journal.pone.0141971 (PMC4635008; doi:10.1371/journal.pone.0141971)
Supplement: S2 Dataset — We analyzed the JPEGs from the films using a custom written MATLAB script as in Pinter-Wollman et al. [13] (code provided in S1 Appendix). We observed ten outgoing foragers and four or five descending ants per film, the first foraging and descending ants in the video segment that were clearly visible. This dataset shows the time each ant entered and left the entrance chamber, and the time and location of each interaction of the focal ants. (PDF) [file pone.0141971.s003.pdf]

2012 Ant Interactions Data

Notes: The first and last entry for each ant marks where and when the ant left the entrance chamber and entered the entrance chamber respectively. All the other entries record information on each of the interactions the focal ant experienced while in the entrance chamber. There are line spaces between each video. "x", "y" are spatial coordinate values.
Ant type 1 = Outgoing foragers
Ant type 2 = Descending ants

Ant id = labels for each ant within each video (not indicative of chronological order)

Each video was divided into frames (30 frames/sec)

Researcher Evie Pless

**colony**	**date**	**ant id**	**x**	**y**	**frame from file name**	**ant type**
N2	8/13/12	1	373.307175	291.674888	2303	1
N2	8/13/12	1	437.020179	336.446188	2269	1
N2	8/13/12	1	454.23991	360.553812	2249	1
N2	8/13/12	1	443.908072	377.773543	2242	1
N2	8/13/12	1	478.347534	381.217489	2204	1
N2	8/13/12	1	464.571749	398.43722	2195	1
N2	8/13/12	1	481.79148	425.988789	2135	1
N2	8/13/12	1	480.069507	389.827354	2129	1
N2	8/13/12	1	500.733184	345.056054	2102	1
N2	8/13/12	1	552.392377	357.109865	2085	1
N2	8/13/12	1	598.88565	360.553812	2005	1
N2	8/13/12	1	11.692825	43.710762	1977	1
N2	8/13/12	2	352.894366	344.443662	4283	1
N2	8/13/12	2	332.612676	335.992958	4278	1
N2	8/13/12	2	325.852113	437.401408	4186	1
N2	8/13/12	2	314.021127	483.035211	4138	1
N2	8/13/12	2	302.190141	559.091549	4119	1
N2	8/13/12	2	415.429577	369.795775	3962	1
N2	8/13/12	2	510.077465	393.457746	3880	1
N2	8/13/12	2	525.288732	368.105634	3864	1
N2	8/13/12	2	623.316901	356.274648	3837	1
N2	8/13/12	2	626.697183	310.640845	3798	1
N2	8/13/12	2	594.584507	325.852113	3780	1
N2	8/13/12	2	575.992958	314.021127	3776	1
N2	8/13/12	3	822.753521	286.978873	2327	1
N2	8/13/12	3	765.288732	413.739437	2164	1

N2	8/13/12	3	817.683099	439.091549	2093	1
N2	8/13/12	3	814.302817	484.725352	2058	1
N2	8/13/12	3	844.725352	483.035211	2042	1
N2	8/13/12	3	907.260563	493.176056	2016	1
N2	8/13/12	3	910.640845	479.65493	1977	1
N2	8/13/12	3	922.471831	479.65493	1976	1
N2	8/13/12	3	895.429577	724.725352	1876	1
N2	8/13/12	3	587.823944	557.401408	1761	1
N2	8/13/12	3	543.880282	511.767606	1697	1
N2	8/13/12	3	525.288732	476.274648	1588	1
N2	8/13/12	3	596.274648	400.21831	1481	1
N2	8/13/12	3	577.683099	361.34507	1476	1
N2	8/13/12	3	560.78169	379.93662	1474	1
N2	8/13/12	3	603.035211	351.204225	1437	1
N2	8/13/12	3	570.922535	317.401408	1420	1
N2	8/13/12	3	574.302817	312.330986	1418	1
N2	8/13/12	4	374.814721	295.626904	5358	1
N2	8/13/12	4	528.317259	385.170051	5229	1
N2	8/13/12	4	606.895939	359.586294	5206	1
N2	8/13/12	4	574.002538	368.72335	5179	1
N2	8/13/12	4	597.758883	344.967005	5175	1
N2	8/13/12	4	586.794416	319.383249	5167	1
N2	8/13/12	4	542.936548	365.068528	5144	1
N2	8/13/12	4	517.352792	354.104061	5126	1
N2	8/13/12	4	504.560914	334.002538	5029	1
N2	8/13/12	4	510.043147	344.967005	4984	1
N2	8/13/12	4	517.352792	335.829949	4892	1
N2	8/13/12	4	539.281726	313.901015	4864	1
N2	8/13/12	5	321.819797	323.038071	7713	1
N2	8/13/12	5	351.058376	370.550761	7698	1
N2	8/13/12	5	287.098985	390.652284	7679	1
N2	8/13/12	5	318.164975	460.093909	7546	1
N2	8/13/12	5	524.662437	584.357868	7263	1
N2	8/13/12	5	493.596447	405.271574	7161	1
N2	8/13/12	5	475.322335	359.586294	7152	1
N2	8/13/12	5	471.667513	381.515228	7128	1
N2	8/13/12	5	502.733503	348.621827	7078	1
N2	8/13/12	5	564.865482	306.591371	7051	1
N2	8/13/12	6	361.091133	299.317734	1205	1
N2	8/13/12	6	440.894089	405.721675	1129	1
N2	8/13/12	6	478.135468	393.307882	1113	1
N2	8/13/12	6	474.58867	363.160099	1106	1

N2	8/13/12	6	510.05665	348.972906	1087	1
N2	8/13/12	6	510.05665	361.3867	1083	1
N2	8/13/12	6	621.780788	336.559113	1052	1
N2	8/13/12	6	593.406404	331.238916	1044	1
N2	8/13/12	6	602.273399	290.450739	1020	1
N2	8/13/12	6	589.859606	285.130542	1016	1
N2	8/13/12	7	589.859606	237.248768	8182	1
N2	8/13/12	7	586.312808	258.529557	8171	1
N2	8/13/12	7	616.460591	242.568966	8065	1
N2	8/13/12	7	598.726601	364.933498	8030	1
N2	8/13/12	7	543.751232	354.293103	8014	1
N2	8/13/12	7	559.711823	306.41133	7995	1
N2	8/13/12	7	549.071429	306.41133	7993	1
N2	8/13/12	8	784.813725	261.732493	9220	1
N2	8/13/12	8	1071.64846	708.119048	8945	1
N2	8/13/12	8	1023.2451	763.693277	8852	1
N2	8/13/12	8	811.704482	858.707283	8654	1
N2	8/13/12	8	542.796919	598.763305	8451	1
N2	8/13/12	8	521.284314	539.603641	8426	1
N2	8/13/12	8	428.063025	498.371148	8365	1
N2	8/13/12	8	458.539216	349.57563	8272	1
N2	8/13/12	8	562.516807	310.135854	8126	1
N2	8/13/12	9	329.463585	363.917367	7604	1
N2	8/13/12	9	340.219888	353.161064	7601	1
N2	8/13/12	9	324.085434	462.516807	7494	1
N2	8/13/12	9	625.261905	453.553221	7369	1
N2	8/13/12	9	671.872549	469.687675	7348	1
N2	8/13/12	9	727.446779	500.163866	7336	1
N2	8/13/12	9	630.640056	521.676471	7308	1
N2	8/13/12	9	524.869748	579.043417	7262	1
N2	8/13/12	9	548.17507	306.55042	7171	1
N2	8/13/12	10	350.97619	326.270308	3645	1
N2	8/13/12	10	517.69888	546.77451	3521	1
N2	8/13/12	10	533.833333	589.79972	3504	1
N2	8/13/12	10	679.043417	849.743697	3422	1
N2	8/13/12	10	689.79972	591.592437	3333	1
N2	8/13/12	10	682.628852	562.908964	3306	1
N2	8/13/12	10	673.665266	484.029412	3248	1
N2	8/13/12	10	761.508403	396.186275	3176	1
N2	8/13/12	10	709.519608	387.222689	3120	1
N2	8/13/12	10	684.421569	324.477591	3104	1
N2	8/13/12	10	637.810924	335.233894	3083	1

N2	8/13/12	10	584.029412	290.415966	3048	1
N2	8/13/12	200	557.208861	291.386076	1668	2
N2	8/13/12	200	555.689873	291.386076	1623	2
N2	8/13/12	200	549.613924	317.208861	1611	2
N2	8/13/12	200	546.575949	338.474684	1594	2
N2	8/13/12	200	461.512658	344.550633	1575	2
N2	8/13/12	200	464.550633	321.765823	1566	2
N2	8/13/12	200	467.588608	308.094937	1564	2
N2	8/13/12	200	472.14557	314.170886	1561	2
N2	8/13/12	201	508.601266	270.120253	3759	2
N2	8/13/12	201	511.639241	273.158228	3727	2
N2	8/13/12	201	481.259494	295.943038	3660	2
N2	8/13/12	201	473.664557	291.386076	3585	2
N2	8/13/12	201	441.765823	305.056962	3567	2
N2	8/13/12	201	450.879747	311.132911	3521	2
N2	8/13/12	201	412.905063	279.234177	3287	2
N2	8/13/12	201	444.803797	309.613924	3244	2
N2	8/13/12	201	466.06962	286.829114	3230	2
N2	8/13/12	201	470.626582	321.765823	3214	2
N2	8/13/12	201	469.107595	311.132911	3194	2
N2	8/13/12	202	546.575949	297.462025	6625	2
N2	8/13/12	202	554.170886	294.424051	6600	2
N2	8/13/12	202	557.208861	289.867089	6596	2
N2	8/13/12	202	545.056962	259.487342	6576	2
N2	8/13/12	202	517.71519	292.905063	6558	2
N2	8/13/12	202	487.335443	308.094937	6487	2
N2	8/13/12	202	475.183544	289.867089	6484	2
N2	8/13/12	202	450.879747	308.094937	6453	2
N2	8/13/12	202	447.841772	286.829114	6451	2
N2	8/13/12	202	438.727848	280.753165	6437	2
N2	8/13/12	202	438.727848	298.981013	6420	2
N2	8/13/12	202	450.879747	317.208861	6417	2
N2	8/13/12	202	426.575949	318.727848	6393	2
N2	8/13/12	202	411.386076	315.689873	6373	2
N2	8/13/12	202	388.601266	327.841772	6352	2
N2	8/13/12	202	387.082278	347.588608	6334	2
N2	8/13/12	202	426.575949	391.639241	6282	2
N2	8/13/12	202	437.208861	379.487342	6278	2
N2	8/13/12	202	441.765823	347.588608	6219	2
N2	8/13/12	202	397.71519	303.537975	6141	2
N2	8/13/12	202	461.512658	315.689873	6086	2

N2	8/13/12	202	470.626582	305.056962	6064	2
N2	8/13/12	203	572.398734	274.677215	8440	2
N2	8/13/12	203	668.094937	280.753165	8344	2
N2	8/13/12	203	662.018987	405.310127	8289	2
N2	8/13/12	203	624.044304	339.993671	8216	2
N2	8/13/12	203	625.563291	361.259494	8207	2
N2	8/13/12	203	611.892405	364.297468	8196	2
N2	8/13/12	203	567.841772	379.487342	8162	2
N2	8/13/12	203	561.765823	285.310127	8113	2
N2	8/13/12	204	555.689873	282.272152	8601	2
N2	8/13/12	204	558.727848	288.348101	8514	2
N2	8/13/12	204	511.639241	265.563291	8384	2
N2	8/13/12	204	513.158228	273.158228	8362	2
N2	8/13/12	204	534.424051	273.158228	8341	2
N2	8/13/12	204	624.044304	192.651899	8044	2
N2	8/13/12	204	1459.48734	604.297468	7958	2
N2	8/13/12	204	630.120253	239.740506	7949	2
N2	8/13/12	204	631.639241	264.044304	7802	2
N2	8/13/12	204	583.031646	297.462025	7746	2
N2	8/13/12	204	593.664557	274.677215	7736	2
N2	8/13/12	204	567.841772	276.196203	7693	2
N2	8/14/12	1	599.861702	251.351064	1777	1
N2	8/14/12	1	599.861702	284.542553	1480	1
N2	8/14/12	1	621.56383	307.521277	1459	1
N2	8/14/12	1	639.43617	310.074468	1297	1
N2	8/14/12	2	747.946809	241.138298	1918	1
N2	8/14/12	2	760.712766	278.159574	1904	1
N2	8/14/12	2	730.074468	303.691489	1837	1
N2	8/14/12	2	717.308511	298.585106	1830	1
N2	8/14/12	3	593.478723	276.882979	2912	1
N2	8/14/12	3	638.159574	324.117021	2865	1
N2	8/14/12	3	653.478723	322.840426	2857	1
N2	8/14/12	3	656.031915	319.010638	2849	1
N2	8/14/12	4	592.202128	306.244681	4831	1
N2	8/14/12	4	647.095745	347.095745	4780	1
N2	8/14/12	4	661.138298	333.053191	4776	1
N2	8/14/12	4	641.989362	311.351064	4775	1
N2	8/14/12	4	656.031915	316.457447	4768	1
N2	8/14/12	5	786.244681	325.393617	5091	1
N2	8/14/12	5	763.265957	289.648936	5074	1
N2	8/14/12	5	761.989362	352.202128	5025	1

N2	8/14/12	5	718.585106	324.117021	4976	1
N2	8/14/12	5	712.202128	303.691489	4879	1
N2	8/14/12	5	726.244681	315.180851	4784	1
N2	8/14/12	5	661.138298	331.776596	4684	1
N2	8/14/12	5	673.904255	325.393617	4678	1
N2	8/14/12	6	574.329787	350.925532	6794	1
N2	8/14/12	6	675.180851	377.734043	6692	1
N2	8/14/12	6	593.478723	339.43617	6763	1
N2	8/14/12	6	659.861702	334.329787	6640	1
N2	8/14/12	6	653.478723	324.117021	6635	1
N2	8/14/12	7	467.095745	313.904255	7374	1
N2	8/14/12	7	487.521277	335.606383	7365	1
N2	8/14/12	7	644.542553	320.287234	7076	1
N2	8/14/12	7	658.585106	317.734043	7071	1
N2	8/14/12	8	537.308511	347.095745	7517	1
N2	8/14/12	8	544.968085	335.606383	7511	1
N2	8/14/12	8	610.074468	412.202128	7460	1
N2	8/14/12	8	639.43617	367.521277	7382	1
N2	8/14/12	8	672.62766	329.223404	7274	1
N2	8/14/12	9	593.478723	316.457447	7881	1
N2	8/14/12	9	611.351064	326.670213	7871	1
N2	8/14/12	9	594.755319	370.074468	7848	1
N2	8/14/12	9	588.37234	377.734043	7838	1
N2	8/14/12	9	664.968085	329.223404	7797	1
N2	8/14/12	9	654.755319	319.010638	7792	1
N2	8/14/12	10	599.861702	285.819149	8743	1
N2	8/14/12	10	606.244681	319.010638	8674	1
N2	8/14/12	10	644.542553	336.882979	8574	1
N2	8/14/12	10	641.989362	311.351064	8529	1
N2	8/14/12	10	656.031915	322.840426	8514	1
N2	8/14/12	101	710.626582	303.537975	2337	2
N2	8/14/12	101	712.14557	300.5	2333	2
N2	8/14/12	101	713.664557	297.462025	2328	2
N2	8/14/12	101	739.487342	309.613924	2185	2
N2	8/14/12	101	698.474684	352.14557	2151	2
N2	8/14/12	101	728.85443	317.208861	2123	2
N2	8/14/12	101	724.297468	298.981013	2068	2
N2	8/14/12	101	689.360759	327.841772	2004	2
N2	8/14/12	101	721.259494	288.348101	1864	2
N2	8/14/12	101	709.107595	305.056962	1629	2
N2	8/14/12	102	715.183544	295.943038	3400	2

N2	8/14/12	102	706.06962	295.943038	3379	2
N2	8/14/12	102	730.373418	298.981013	3189	2
N2	8/14/12	102	719.514925	301.126866	3049	2
N2	8/14/12	102	703.395522	326.91791	2931	2
N2	8/14/12	102	707.588608	303.537975	2863	2
N2	8/14/12	102	736.449367	298.981013	2752	2
N2	8/14/12	102	724.297468	294.424051	2683	2
N2	8/14/12	103	715.183544	292.905063	3924	2
N2	8/14/12	103	724.297468	280.753165	3875	2
N2	8/14/12	103	710.626582	294.424051	3870	2
N2	8/14/12	103	552.651899	332.398734	3744	2
N2	8/14/12	103	584.550633	339.993671	3717	2
N2	8/14/12	103	634.677215	350.626582	3675	2
N2	8/14/12	103	633.158228	338.474684	3663	2
N2	8/14/12	103	646.829114	361.259494	3635	2
N2	8/14/12	103	660.5	346.06962	3590	2
N2	8/14/12	103	642.272152	330.879747	3583	2
N2	8/14/12	103	668.094937	327.841772	3515	2
N2	8/14/12	103	663.537975	326.322785	3495	2
N2	8/14/12	104	719.740506	291.386076	5360	2
N2	8/14/12	104	713.664557	309.613924	5335	2
N2	8/14/12	104	728.85443	305.056962	5316	2
N2	8/14/12	104	721.259494	323.28481	5312	2
N2	8/14/12	104	675.689873	370.373418	5238	2
N2	8/14/12	104	672.651899	330.879747	5208	2
N2	8/14/12	104	677.208861	329.360759	5082	2
N2	8/14/12	104	734.93038	311.132911	4991	2
N2	8/14/12	104	718.221519	314.170886	4987	2
N2	8/14/12	104	722.778481	294.424051	4974	2
N2	8/14/12	105	731.892405	286.829114	6588	2
N2	8/14/12	105	722.778481	289.867089	6545	2
N2	8/14/12	105	734.93038	274.677215	6521	2
N2	8/14/12	105	716.702532	302.018987	6405	2
N2	8/14/12	105	710.626582	303.537975	6404	2
N2	8/15/12	1	1360.71277	335.606383	1367	1
N2	8/15/12	1	1323.69149	347.095745	1354	1
N2	8/15/12	1	1310.92553	339.43617	1346	1
N2	8/15/12	1	1314.75532	336.882979	1335	1
N2	8/15/12	1	1318.58511	317.734043	1328	1
N2	8/15/12	1	1328.79787	335.606383	1327	1
N2	8/15/12	2	1335.18085	364.968085	2932	1

N2	8/15/12	2	1305.81915	356.031915	2920	1
N2	8/15/12	2	1295.60638	345.819149	2916	1
N2	8/15/12	2	1317.30851	335.606383	2903	1
N2	8/15/12	2	1322.41489	331.776596	2897	1
N2	8/15/12	3	1307.09575	366.244681	3238	1
N2	8/15/12	3	1296.88298	362.414894	3227	1
N2	8/15/12	3	1312.20213	345.819149	3222	1
N2	8/15/12	3	1304.54255	325.393617	3219	1
N2	8/15/12	3	1316.03192	331.776596	3215	1
N2	8/15/12	4	1377.30851	398.159574	4284	1
N2	8/15/12	4	1333.90426	395.606383	4226	1
N2	8/15/12	4	1321.1383	345.819149	4215	1
N2	8/15/12	4	1322.41489	331.776596	4209	1
N2	8/15/12	4	1331.35106	322.840426	4201	1
N2	8/15/12	5	1281.56383	576.882979	5960	1
N2	8/15/12	5	1268.79787	597.308511	5955	1
N2	8/15/12	5	1253.47872	584.542553	5943	1
N2	8/15/12	5	1208.79787	539.861702	5861	1
N2	8/15/12	5	1231.7766	539.861702	5853	1
N2	8/15/12	5	1249.64894	528.37234	5841	1
N2	8/15/12	5	1259.8617	468.37234	5811	1
N2	8/15/12	5	1266.24468	436.457447	5799	1
N2	8/15/12	5	1263.69149	414.755319	5779	1
N2	8/15/12	5	1277.73404	399.43617	5764	1
N2	8/15/12	5	1287.94681	372.62766	5750	1
N2	8/15/12	5	1332.62766	363.691489	5737	1
N2	8/15/12	5	1316.03192	348.37234	5731	1
N2	8/15/12	5	1331.35106	329.223404	5717	1
N2	8/15/12	6	1355.60638	419.861702	5306	1
N2	8/15/12	6	1361.98936	407.095745	5296	1
N2	8/15/12	6	1295.60638	398.159574	5261	1
N2	8/15/12	6	1286.67021	372.62766	5242	1
N2	8/15/12	6	1301.98936	363.691489	5240	1
N2	8/15/12	6	1299.43617	324.117021	5217	1
N2	8/15/12	6	1324.96809	329.223404	5212	1
N2	8/15/12	6	1314.75532	321.56383	5211	1
N2	8/15/12	7	1333.90426	382.840426	7048	1
N2	8/15/12	7	1336.45745	364.968085	7008	1
N2	8/15/12	7	1310.92553	331.776596	6990	1
N2	8/15/12	7	1322.41489	324.117021	6981	1
N2	8/15/12	7	1328.79787	326.670213	6979	1
N2	8/15/12	8	1273.90426	196.457447	5546	1

N2	8/15/12	8	1261.1383	200.287234	5538	1
N2	8/15/12	8	1281.56383	211.776596	5522	1
N2	8/15/12	8	1262.41489	215.606383	5512	1
N2	8/15/12	8	1284.11702	234.755319	5489	1
N2	8/15/12	8	1266.24468	244.968085	5481	1
N2	8/15/12	8	1279.01064	261.56383	5476	1
N2	8/15/12	8	1584.11702	108.37234	5464	1
N2	8/15/12	8	1284.11702	264.117021	5467	1
N2	8/15/12	9	1321.1383	359.861702	7544	1
N2	8/15/12	9	1319.8617	324.117021	7527	1
N2	8/15/12	10	1653.05319	416.031915	8882	1
N2	8/15/12	10	1312.20213	371.351064	8800	1
N2	8/15/12	10	1294.32979	371.351064	8796	1
N2	8/15/12	10	1318.58511	334.329787	8779	1
N2	8/15/12	10	1322.41489	329.223404	8773	1
N2	8/15/12					
N2	8/15/12	130	1304.13636	326.911483	6670	2
N2	8/15/12	130	1257.62919	338.9689	6611	2
N2	8/15/12	130	1264.51914	382.0311	6589	2
N2	8/15/12	130	1255.9067	382.0311	6582	2
N2	8/15/12	130	1224.90191	447.485646	6555	2
N2	8/15/12	130	1145.66746	447.485646	6515	2
N2	8/15/12	130	1159.44737	450.930622	6496	2
N2	8/15/12	130	1183.5622	404.423445	6465	2
N2	8/15/12	130	1212.8445	394.088517	6420	2
N2	8/15/12	130	1219.73445	421.648325	6404	2
N2	8/15/12	130	1261.07416	430.260766	6368	2
N2	8/15/12	130	1273.13158	399.255981	6323	2
N2	8/15/12	130	1304.13636	399.255981	5869	2
N2	8/15/12	130	1307.58134	383.753589	5851	2
N2	8/15/12	130	1292.07895	380.308612	5838	2
N2	8/15/12	130	1281.74402	387.198565	5824	2
N2	8/15/12	130	1317.91627	356.19378	5779	2
N2	8/15/12	137	1305.85885	328.633971	7555	2
N2	8/15/12	137	1262.79665	345.858852	7543	2
N2	8/15/12	137	1285.189	356.19378	7527	2
N2	8/15/12	137	1285.189	380.308612	7471	2
N2	8/15/12	137	1335.14115	354.471292	7445	2
N2	8/15/12	137	1438.49043	531.88756	7324	2
N2	8/15/12	137	1385.0933	426.815789	7282	2
N2	8/15/12	137	1324.80622	428.538278	7265	2
N2	8/15/12	137	1323.08373	507.772727	7224	2

N2	8/15/12	137	1314.47129	521.552632	7205	2
N2	8/15/12	137	1309.30383	481.935407	7115	2
N2	8/15/12	137	1274.85407	487.102871	7087	2
N2	8/15/12	137	1254.18421	395.811005	6935	2
N2	8/15/12	137	1278.29904	351.026316	6909	2
N2	8/15/12	137	1276.57656	282.126794	6894	2
N2	8/15/12	119	1307.58134	335.523923	4450	2
N2	8/15/12	119	1271.40909	399.255981	4367	2
N2	8/15/12	119	1262.79665	404.423445	4333	2
N2	8/15/12	119	1235.23684	394.088517	4293	2
N2	8/15/12	119	1202.50957	385.476077	4223	2
N2	8/15/12	119	1190.45215	344.136364	4199	2
N2	8/15/12	119	1238.68182	333.801435	4130	2
N2	8/15/12	119	1300.69139	338.9689	4067	2
N2	8/15/12	119	1293.80144	356.19378	4058	2
N2	8/15/12	133	1274.85407	275.236842	7417	2
N2	8/15/12	133	1205.95455	316.576555	7358	2
N2	8/15/12	133	1147.38995	369.973684	7305	2
N2	8/15/12	133	1157.72488	375.141148	7300	2
N2	8/15/12	133	1112.94019	399.255981	7281	2
N2	8/15/12	133	1214.56699	459.543062	7173	2
N2	8/15/12	133	1290.35646	500.882775	7079	2
N2	8/15/12	133	1211.12201	550.834928	7028	2
N2	8/15/12	133	1226.6244	559.447368	6995	2
N2	8/15/12	133	1174.94976	688.633971	6807	2
N2	8/15/12	133	1230.06938	550.834928	6630	2
N2	8/15/12	133	1204.23206	543.944976	6356	2
N2	8/15/12	133	1187.00718	471.600478	6322	2
N2	8/15/12	133	1321.36124	402.700957	6235	2
N2	8/15/12	133	1302.41388	340.691388	6194	2
N2	8/15/12	133	1319.63876	340.691388	6183	2
N4	8/13/12	1	1336.45745	390.5	1308	1
N4	8/13/12	1	1279.01064	481.138298	1292	1
N4	8/13/12	1	1268.79787	520.712766	1284	1
N4	8/13/12	1	1248.37234	533.478723	1276	1
N4	8/13/12	1	1276.45745	538.585106	1272	1
N4	8/13/12	1	1291.7766	661.138298	1249	1
N4	8/13/12	1	1243.26596	753.053191	1225	1
N4	8/13/12	2	1346.67021	722.414894	2168	1
N4	8/13/12	2	1303.26596	724.968085	2154	1
N4	8/13/12	2	1299.43617	753.053191	2144	1

N4	8/13/12	2	1303.26596	783.691489	2131	1
N4	8/13/12	3	1346.67021	722.414894	3157	1
N4	8/13/12	3	1342.84043	713.478723	3156	1
N4	8/13/12	3	1321.1383	732.62766	3150	1
N4	8/13/12	3	1321.1383	739.010638	3144	1
N4	8/13/12	3	1296.88298	755.606383	3135	1
N4	8/13/12	3	1289.2234	773.478723	3127	1
N4	8/13/12	4	1345.39362	643.265957	4572	1
N4	8/13/12	4	1350.5	641.989362	4561	1
N4	8/13/12	4	1327.52128	639.43617	4558	1
N4	8/13/12	4	1317.30851	661.138298	4551	1
N4	8/13/12	4	1327.52128	673.904255	4537	1
N4	8/13/12	4	1331.35106	698.159574	4526	1
N4	8/13/12	4	1332.62766	691.776596	4516	1
N4	8/13/12	4	1307.09575	726.244681	4507	1
N4	8/13/12	4	1304.54255	733.904255	4493	1
N4	8/13/12	4	1281.56383	781.138298	4481	1
N4	8/13/12	5	1335.18085	336.882979	5310	1
N4	8/13/12	5	1323.69149	315.180851	5307	1
N4	8/13/12	5	1261.1383	386.670213	5294	1
N4	8/13/12	5	1203.69149	407.095745	5285	1
N4	8/13/12	5	1178.15957	424.968085	5279	1
N4	8/13/12	5	1125.81915	439.010638	5260	1
N4	8/13/12	5	1130.92553	509.223404	5241	1
N4	8/13/12	5	1226.67021	530.925532	5219	1
N4	8/13/12	5	1267.52128	556.457447	5212	1
N4	8/13/12	5	1263.69149	592.202128	5207	1
N4	8/13/12	5	1331.35106	574.329787	5197	1
N4	8/13/12	5	1342.84043	589.648936	5190	1
N4	8/13/12	5	1323.69149	599.861702	5187	1
N4	8/13/12	5	1323.69149	627.946809	5184	1
N4	8/13/12	5	1322.41489	625.393617	5177	1
N4	8/13/12	5	1324.96809	654.755319	5170	1
N4	8/13/12	5	1308.37234	666.244681	5157	1
N4	8/13/12	5	1319.8617	676.457447	5153	1
N4	8/13/12	5	1296.88298	685.393617	5141	1
N4	8/13/12	5	1316.03192	680.287234	5130	1
N4	8/13/12	5	1312.20213	723.691489	5105	1
N4	8/13/12	5	1291.7766	776.031915	5078	1
N4	8/13/12	6	1351.7766	736.457447	5225	1
N4	8/13/12	6	1365.81915	724.968085	5224	1
N4	8/13/12	6	1339.01064	718.585106	5220	1

N4	8/13/12	6	1328.79787	754.329787	5206	1
N4	8/13/12	6	1314.75532	749.223404	5196	1
N4	8/13/12	6	1312.20213	758.159574	5185	1
N4	8/13/12	6	1318.58511	761.989362	5175	1
N4	8/13/12	6	1326.24468	767.095745	5174	1
N4	8/13/12	6	1318.58511	776.031915	5172	1
N4	8/13/12	7	1345.39362	675.180851	6933	1
N4	8/13/12	7	1350.5	664.968085	6932	1
N4	8/13/12	7	1328.79787	677.734043	6924	1
N4	8/13/12	7	1317.30851	684.117021	6914	1
N4	8/13/12	7	1293.05319	693.053191	6901	1
N4	8/13/12	7	1300.71277	712.202128	6884	1
N4	8/13/12	7	1304.54255	755.606383	6873	1
N4	8/13/12	7	1280.28723	772.202128	6860	1
N4	8/13/12	8	1342.84043	647.095745	7396	1
N4	8/13/12	8	1337.73404	634.329787	7386	1
N4	8/13/12	8	1324.96809	663.691489	7382	1
N4	8/13/12	8	1327.52128	677.734043	7379	1
N4	8/13/12	8	1336.45745	684.117021	7378	1
N4	8/13/12	8	1275.18085	745.393617	7362	1
N4	8/13/12	8	1261.1383	754.329787	7360	1
N4	8/13/12	8	1268.79787	768.37234	7358	1
N4	8/13/12	9	1346.67021	733.904255	7931	1
N4	8/13/12	9	1337.73404	765.819149	7913	1
N4	8/13/12	9	1323.69149	777.308511	7905	1
N4	8/13/12	10	1353.05319	737.734043	9298	1
N4	8/13/12	10	1359.43617	728.797872	9296	1
N4	8/13/12	10	1383.69149	745.393617	9304	1
N4	8/13/12	10	1373.47872	758.159574	9287	1
N4	8/13/12	10	1369.64894	724.968085	9273	1
N4	8/13/12	10	1340.28723	710.925532	9258	1
N4	8/13/12	10	1354.32979	698.159574	9252	1
N4	8/13/12	10	1328.79787	714.755319	9244	1
N4	8/13/12	10	1344.11702	732.62766	9240	1
N4	8/13/12	10	1319.8617	745.393617	9239	1
N4	8/13/12	10	1281.56383	776.031915	9228	1
N4	8/13/12					
N4	8/13/12	130	1325.40674	774.474175	8943	2
N4	8/13/12	130	1401.33214	734.187231	8966	2
N4	8/13/12	130	1409.07963	738.835725	8987	2
N4	8/13/12	130	1398.23314	726.439742	8988	2
N4	8/13/12	130	1404.43113	738.835725	8998	2

N4	8/13/12	130	1385.83716	704.746772	9056	2
N4	8/13/12	130	1340.90172	683.053802	9075	2
N4	8/13/12	130	1319.20875	577.687948	9113	2
N4	8/13/12	130	1323.85725	552.895983	9170	2
N4	8/13/12	130	1317.65925	557.544476	9157	2
N4	8/13/12	130	1339.35222	579.237446	9137	2
N4	8/13/12	131	1260.5	710.626582	4179	2
N4	8/13/12	131	1274.17089	649.867089	4193	2
N4	8/13/12	131	1260.5	631.639241	4195	2
N4	8/13/12	131	1289.36076	613.411392	4213	2
N4	8/13/12	131	1287.84177	602.778481	4234	2
N4	8/13/12	131	1287.84177	610.373418	4262	2
N4	8/13/12	131	1266.57595	583.031646	4279	2
N4	8/13/12	131	1251.38608	589.107595	4318	2
N4	8/13/12	131	1246.82911	555.689873	4361	2
N4	8/13/12	131	1237.71519	578.474684	4372	2
N4	8/13/12	132	1318.22152	569.360759	5620	2
N4	8/13/12	132	1295.43671	595.183544	5625	2
N4	8/13/12	132	1258.98101	587.588608	5634	2
N4	8/13/12	132	1237.71519	567.841772	5650	2
N4	8/13/12	132	1260.5	672.651899	5680	2
N4	8/13/12	132	1272.6519	668.094937	5684	2
N4	8/13/12	132	1245.31013	709.107595	5696	2
N4	8/13/12	132	1255.94304	757.71519	5707	2
N4	8/13/12	133	1342.52532	573.917722	784	2
N4	8/13/12	133	1348.60127	575.436709	773	2
N4	8/13/12	133	1339.48734	631.639241	751	2
N4	8/13/12	133	1351.63924	596.702532	759	2
N4	8/13/12	133	1344.0443	651.386076	742	2
N4	8/13/12	133	1345.56329	692.398734	717	2
N4	8/13/12	133	1313.66456	689.360759	692	2
N4	8/13/12	133	1277.20886	690.879747	680	2
N4	8/13/12	133	1272.6519	719.740506	659	2
N4	8/13/12	133	1266.57595	753.158228	655	2
N4	8/13/12	133	1307.58861	681.765823	689	2
N4	8/13/12	133	1322.77848	645.310127	736	2
N4						
N4	8/14/12	1	964.688482	197.044503	1197	1
N4	8/14/12	1	963.013089	240.604712	1183	1
N4	8/14/12	1	946.259162	264.060209	1175	1
N4	8/14/12	1	916.102094	257.358639	1169	1
N4	8/14/12	1	921.128272	284.164921	1165	1

N4	8/14/12	1	875.89267	240.604712	1149	1
N4	8/14/12	1	894.32199	243.955497	1120	1
N4	8/14/12	1	899.348168	300.918848	1096	1
N4	8/14/12	1	880.918848	327.725131	1085	1
N4	8/14/12	1	897.672775	359.557592	1076	1
N4	8/14/12	1	884.269634	362.908377	1074	1
N4	8/14/12	1	902.698953	393.065445	1065	1
N4	8/14/12	1	859.138743	396.41623	1048	1
N4	8/14/12	1	892.646597	446.67801	1020	1
N4	8/14/12	1	909.400524	460.081152	1016	1
N4	8/14/12	2	1041.75655	188.667539	1327	1
N4	8/14/12	2	916.102094	245.63089	1263	1
N4	8/14/12	2	929.505236	264.060209	1250	1
N4	8/14/12	2	884.269634	274.112565	1225	1
N4	8/14/12	2	916.102094	324.374346	1160	1
N4	8/14/12	2	919.45288	307.620419	1158	1
N4	8/14/12	2	904.374346	372.960733	1125	1
N4	8/14/12	2	889.295812	403.117801	1111	1
N4	8/14/12	2	917.777487	409.819372	1099	1
N4	8/14/12	3	756.939791	212.123037	1832	1
N4	8/14/12	3	736.835079	212.123037	1831	1
N4	8/14/12	3	765.316754	243.955497	1822	1
N4	8/14/12	3	834.007853	269.086387	1807	1
N4	8/14/12	3	852.437173	284.164921	1799	1
N4	8/14/12	3	834.007853	383.013089	1778	1
N4	8/14/12	3	890.971204	426.573298	1764	1
N4	8/14/12	3	869.191099	453.379581	1753	1
N4	8/14/12	3	884.269634	488.562827	1741	1
N4	8/14/12	4	798.76087	208.326087	1617	1
N4	8/14/12	4	788.326087	201.369565	1615	1
N4	8/14/12	4	816.152174	203.108696	1612	1
N4	8/14/12	4	823.108696	222.23913	1603	1
N4	8/14/12	4	823.108696	255.282609	1590	1
N4	8/14/12	4	861.369565	239.630435	1580	1
N4	8/14/12	4	843.978261	270.934783	1559	1
N4	8/14/12	4	803.978261	383.978261	1530	1
N4	8/14/12	4	833.543478	377.021739	1524	1
N4	8/14/12	4	828.326087	429.195652	1511	1
N4	8/14/12	4	847.456522	444.847826	1505	1
N4	8/14/12	4	897.891304	450.065217	1495	1
N4	8/14/12	4	913.543478	457.021739	1493	1
N4	8/14/12	5	677.021739	253.543478	2354	1

N4	8/14/12	5	668.326087	246.586957	2343	1
N4	8/14/12	5	701.369565	272.673913	2324	1
N4	8/14/12	5	609.195652	342.23913	2256	1
N4	8/14/12	5	673.543478	343.978261	2228	1
N4	8/14/12	5	673.543478	378.76087	2218	1
N4	8/14/12	5	692.673913	413.543478	2166	1
N4	8/14/12	5	668.326087	443.108696	2148	1
N4	8/14/12	5	727.456522	483.108696	2073	1
N4	8/14/12	5	743.108696	490.065217	2047	1
N4	8/14/12	5	751.804348	444.847826	2018	1
N4	8/14/12	5	824.847826	450.065217	1956	1
N4	8/14/12	5	835.282609	462.23913	1948	1
N4	8/14/12	5	833.543478	448.326087	1937	1
N4	8/14/12	5	861.369565	458.76087	1918	1
N4	8/14/12	5	845.717391	467.456522	1900	1
N4	8/14/12	5	896.152174	457.021739	1835	1
N4	8/14/12	6	690.934783	260.5	2953	1
N4	8/14/12	6	737.891304	251.804348	2934	1
N4	8/14/12	6	850.934783	368.326087	2903	1
N4	8/14/12	6	833.543478	389.195652	2881	1
N4	8/14/12	6	842.23913	420.5	2872	1
N4	8/14/12	6	901.369565	439.630435	2828	1
N4	8/14/12	7	964.1	185	4464	1
N4	8/14/12	7	958.7	240.8	4415	1
N4	8/14/12	7	926.3	217.4	4398	1
N4	8/14/12	7	892.1	253.4	4377	1
N4	8/14/12	7	915.5	273.2	4356	1
N4	8/14/12	7	879.5	302	4293	1
N4	8/14/12	7	890.3	303.8	4222	1
N4	8/14/12	7	854.3	323.6	4135	1
N4	8/14/12	7	874.1	323.6	3994	1
N4	8/14/12	7	879.5	296.6	3972	1
N4	8/14/12	7	866.9	311	3945	1
N4	8/14/12	7	881.3	311	3867	1
N4	8/14/12	7	886.7	271.4	3755	1
N4	8/14/12	7	884.9	293	3693	1
N4	8/14/12	7	884.9	357.8	3590	1
N4	8/14/12	7	888.5	424.4	3562	1
N4	8/14/12	7	908.3	428	3557	1
N4	8/14/12	8	726.5	213.8	2601	1
N4	8/14/12	8	766.1	316.4	2567	1
N4	8/14/12	8	791.3	312.8	2565	1

N4	8/14/12	8	764.3	359.6	2554	1
N4	8/14/12	8	776.9	357.8	2545	1
N4	8/14/12	8	776.9	410	2523	1
N4	8/14/12	8	814.7	428	2483	1
N4	8/14/12	8	751.7	379.4	2437	1
N4	8/14/12	8	726.5	393.8	2429	1
N4	8/14/12	8	757.1	361.4	2418	1
N4	8/14/12	8	832.7	300.2	2360	1
N4	8/14/12	8	852.5	377.6	2302	1
N4	8/14/12	8	848.9	417.2	2271	1
N4	8/14/12	8	877.7	426.2	2224	1
N4	8/14/12	8	886.7	442.4	2204	1
N4	8/14/12	8	847.1	442.4	2195	1
N4	8/14/12	8	857.9	451.4	2182	1
N4	8/14/12	8	892.1	478.4	2169	1
N4	8/14/12	9	782.448718	236.910256	2761	1
N4	8/14/12	9	823.474359	274.653846	2750	1
N4	8/14/12	9	803.782051	300.910256	2744	1
N4	8/14/12	9	877.628205	420.705128	2699	1
N4	8/14/12	9	890.75641	423.987179	2683	1
N4	8/14/12	9	917.012821	433.833333	2644	1
N4	8/14/12	10	644.602564	277.935897	2392	1
N4	8/14/12	10	649.525641	323.884615	2369	1
N4	8/14/12	10	728.294872	330.448718	2338	1
N4	8/14/12	10	728.294872	359.987179	2322	1
N4	8/14/12	10	739.782051	386.24359	2313	1
N4	8/14/12	10	784.089744	387.884615	2291	1
N4	8/14/12	10	830.038462	387.884615	2278	1
N4	8/14/12	10	790.653846	338.653846	2174	1
N4	8/14/12	10	761.115385	351.782051	2166	1
N4	8/14/12	10	759.474359	384.602564	2157	1
N4	8/14/12	10	759.474359	397.730769	2150	1
N4	8/14/12	10	700.397436	392.807692	2113	1
N4	8/14/12	200	697.172158	415.953048	1912	2
N4	8/14/12	200	714.96458	460.434102	1930	2
N4	8/14/12	200	714.96458	471.109555	1949	2
N4	8/14/12	200	786.134267	508.473641	2046	2
N4	8/14/12	200	819.939868	478.226524	2075	2
N4	8/14/12	200	876.875618	485.343493	2115	2
N4	8/14/12	201	906.340708	421.827434	2639	2
N4	8/14/12	201	863.331858	389.969027	2658	2

N4	8/14/12	201	739.084071	362.889381	2716	2
N4	8/14/12	201	755.013274	386.783186	2731	2
N4	8/14/12	201	694.482301	401.119469	2824	2
N4	8/14/12	202	825.10177	485.544248	4267	2
N4	8/14/12	202	818.730088	491.915929	4211	2
N4	8/14/12	202	793.243363	487.137168	4180	2
N4	8/14/12	202	820.323009	474.393805	4043	2
N4	8/14/12	202	831.473451	436.163717	3976	2
N4	8/14/12	202	791.650442	375.632743	3938	2
N4	8/14/12	202	774.128319	421.827434	3912	2
N4	8/14/12	202	810.765487	439.349558	3878	2
N4	8/14/12	202	892.004425	474.393805	3793	2
N4	8/14/12	203	1181.19565	536.586957	9263	2
N4	8/14/12	203	1196.84783	362.847826	9102	2
N4	8/14/12	203	1178.06522	293.978261	9053	2
N4	8/14/12	203	1246.93478	237.630435	8655	2
N4	8/14/12	203	1409.71739	315.891304	8555	2
N4	8/14/12	203	1383.1087	387.891304	8516	2
N4	8/14/12	203	1311.1087	447.369565	8453	2
N4	8/14/12	203	1237.54348	428.586957	8379	2
N4	8/14/12	204	1223.45652	602.326087	8862	2
N4	8/14/12	204	1178.06522	702.5	8817	2
N4	8/14/12	204	1027.80435	788.586957	8659	2
N4	8/14/12	204	836.847826	765.108696	8586	2
N4	8/14/12	204	429.891304	921.630435	8434	2
N4	8/14/12	204	434.586957	844.934783	8355	2
N4	8/14/12	204	401.717391	721.282609	8185	2
N4	8/14/12	204	512.847826	715.021739	8077	2
N4	8/14/12	204	586.413043	686.847826	7906	2
N4	8/14/12	204	1198.41304	527.195652	7576	2
N4						
N4	8/15/12	1	1258.91642	602.435484	1166	1
N4	8/15/12	1	1275.80792	576.159824	1169	1
N4	8/15/12	1	1292.69941	587.420821	1171	1
N4	8/15/12	1	1300.20675	538.623167	1183	1
N4	8/15/12	1	1272.05425	523.608504	1195	1
N4	8/15/12	1	1296.45308	501.08651	1198	1
N4	8/15/12	1	1118.15396	426.013196	1236	1
N4	8/15/12	1	1056.21848	403.491202	1288	1
N4	8/15/12	1	1061.84897	392.230205	1295	1
N4	8/15/12	1	1067.47947	347.186217	1310	1
N4	8/15/12	1	894.81085	302.142229	1394	1

N4	8/15/12	1	106.541056	407.244868	1886	1
N4	8/15/12	2	1303.96041	619.326979	1197	1
N4	8/15/12	2	1332.1129	585.543988	1209	1
N4	8/15/12	2	1392.17155	593.05132	1220	1
N4	8/15/12	2	1425.95455	591.174487	1232	1
N4	8/15/12	2	1442.84604	568.652493	1248	1
N4	8/15/12	2	1517.91936	581.790323	1281	1
N4	8/15/12	3	1471.04546	425.627273	1518	1
N4	8/15/12	3	1465.15455	459.009091	1496	1
N4	8/15/12	3	1445.51818	445.263636	1490	1
N4	8/15/12	3	1410.17273	447.227273	1479	1
N4	8/15/12	3	1410.17273	466.863636	1458	1
N4	8/15/12	3	1398.39091	480.609091	1454	1
N4	8/15/12	3	1394.46364	453.118182	1449	1
N4	8/15/12	3	1370.9	476.681818	1390	1
N4	8/15/12	3	1372.86364	512.027273	1376	1
N4	8/15/12	3	1437.66364	545.409091	1341	1
N4	8/15/12	3	1461.22727	570.936364	1334	1
N4	8/15/12	3	1359.11818	533.627273	1299	1
N4	8/15/12	3	1337.51818	549.336364	1273	1
N4	8/15/12	3	1308.06364	576.827273	1255	1
N4	8/15/12	3	1282.53636	594.5	1248	1
N4	8/15/12	3	1304.13636	623.954545	1238	1
N4	8/15/12	4	1453.13158	439.131579	1795	1
N4	8/15/12	4	1437.97368	433.447368	1792	1
N4	8/15/12	4	1455.02632	490.289474	1773	1
N4	8/15/12	4	1341.34211	569.868421	1743	1
N4	8/15/12	4	1309.13158	600.184211	1727	1
N4	8/15/12	4	1295.86842	585.026316	1721	1
N4	8/15/12	4	1271.23684	611.552632	1712	1
N4	8/15/12	5	1388.19528	316.469142	2262	1
N4	8/15/12	5	1367.82883	303.508679	2255	1
N4	8/15/12	5	1364.12584	340.538573	2234	1
N4	8/15/12	5	1325.24446	351.647541	2220	1
N4	8/15/12	5	1312.28399	296.1027	2203	1
N4	8/15/12	5	1304.87801	309.063163	2117	1
N4	8/15/12	5	1315.98698	342.390068	2105	1
N4	8/15/12	5	1308.581	377.568467	2096	1
N4	8/15/12	5	1114.17406	453.479749	1999	1
N4	8/15/12	5	1247.48168	525.688042	1964	1
N4	8/15/12	5	1262.29364	560.866442	1948	1
N4	8/15/12	5	1297.47204	549.757473	1942	1

N4	8/15/12	5	1299.32353	573.826905	1937	1
N4	8/15/12	5	1264.14513	609.005304	1923	1
N4	8/15/12	6	1384.49229	331.281099	2374	1
N4	8/15/12	6	1380.7893	331.281099	2372	1
N4	8/15/12	6	1362.27435	320.172131	2364	1
N4	8/15/12	6	1354.86837	316.469142	2358	1
N4	8/15/12	6	1306.72951	294.251205	2328	1
N4	8/15/12	6	1336.35342	320.172131	2302	1
N4	8/15/12	6	1375.23481	260.924301	2233	1
N4	8/15/12	6	1330.79894	307.211668	2221	1
N4	8/15/12	6	1293.76905	255.369817	2168	1
N4	8/15/12	6	1303.02652	355.35053	2156	1
N4	8/15/12	6	1295.62054	377.568467	2149	1
N4	8/15/12	6	1290.06606	373.865477	2143	1
N4	8/15/12	6	1254.88766	401.637898	2139	1
N4	8/15/12	6	1214.15477	383.122951	2130	1
N4	8/15/12	6	1188.23385	379.419961	2122	1
N4	8/15/12	6	1206.7488	410.895371	2104	1
N4	8/15/12	6	1190.08534	444.222276	2087	1
N4	8/15/12	6	1140.09499	457.182739	2078	1
N4	8/15/12	6	1277.10559	486.806654	2014	1
N4	8/15/12	6	1278.95709	564.569431	1986	1
N4	8/15/12	6	1243.77869	570.123915	1981	1
N4	8/15/12	7	1269.82317	618.28837	2234	1
N4	8/15/12	7	1291.78694	616.458055	2236	1
N4	8/15/12	7	1288.12631	574.36082	2243	1
N4	8/15/12	7	1304.59914	512.130124	2258	1
N4	8/15/12	7	1354.01764	557.887989	2281	1
N4	8/15/12	7	1355.84795	530.43327	2290	1
N4	8/15/12	7	1344.86606	502.978551	2301	1
N4	8/15/12	7	1412.5877	468.202574	2320	1
N4	8/15/12	8	1421.73928	453.560057	2883	1
N4	8/15/12	8	1394.28456	499.317922	2866	1
N4	8/15/12	8	1326.56292	567.039561	2837	1
N4	8/15/12	8	1322.90229	599.985224	2827	1
N4	8/15/12	8	1339.37512	599.985224	2819	1
N4	8/15/12	8	1275.31411	567.039561	2795	1
N4	8/15/12	8	1299.1082	610.967112	2778	1
N4	8/15/12	8	1300.93851	623.779314	2776	1
N4	8/15/12	9	124.046235	512.130124	4038	1
N4	8/15/12	9	367.478074	596.324595	3733	1
N4	8/15/12	9	493.769781	460.881316	3672	1

N4	8/15/12	9	918.402765	321.777407	3555	1
N4	8/15/12	9	978.803146	343.741182	3541	1
N4	8/15/12	9	991.615348	378.517159	3527	1
N4	8/15/12	9	1053.84604	332.759295	3505	1
N4	8/15/12	9	1121.56768	314.456149	3478	1
N4	8/15/12	9	1176.47712	347.401811	3447	1
N4	8/15/12	9	1191.11964	365.704957	3442	1
N4	8/15/12	9	1225.89562	347.401811	3430	1
N4	8/15/12	9	1288.12631	371.195901	3407	1
N4	8/15/12	9	1297.27788	363.874643	3399	1
N4	8/15/12	9	1238.70782	416.953765	3341	1
N4	8/15/12	9	1229.55624	426.105338	3323	1
N4	8/15/12	9	1264.33222	484.675405	3284	1
N4	8/15/12	9	1238.70782	513.960439	3278	1
N4	8/15/12	9	1269.82317	537.754528	3269	1
N4	8/15/12	9	1255.18065	552.397045	3254	1
N4	8/15/12	9	1249.6897	598.154909	3247	1
N4	8/15/12	10	1449.19399	512.130124	5495	1
N4	8/15/12	10	1407.09676	535.924214	5455	1
N4	8/15/12	10	1337.54481	559.718303	5423	1
N4	8/15/12	10	1310.09009	598.154909	5412	1
N4	8/15/12	10	1304.59914	625.609628	5404	1
N4	8/15/12	103	1281.76582	599.740506	1344	2
N4	8/15/12	103	1278.72785	581.512658	1348	2
N4	8/15/12	103	1298.47468	586.06962	1359	2
N4	8/15/12	103	1269.61392	570.879747	1362	2
N4	8/15/12	103	1310.62658	513.158228	1385	2
N4	8/15/12	103	1286.32279	526.829114	1386	2
N4	8/15/12	103	1310.62658	449.360759	1407	2
N4	8/15/12	103	1233.15823	485.816456	1438	2
N4	8/15/12	103	1193.66456	701.512658	1553	2
N4	8/15/12	103	1210.37342	751.639241	1593	2
N4	8/15/12	103	1201.25949	804.803797	1630	2
N4	8/15/12	103	1207.33544	792.651899	1636	2
N4	8/15/12	103	1236.1962	809.360759	1651	2
N4	8/15/12	103	1213.41139	804.803797	1683	2
N4	8/15/12	103	1249.86709	829.107595	1699	2
N4	8/15/12	103	1318.22152	725.816456	1807	2
N4	8/15/12	103	1333.41139	631.639241	1865	2
N4	8/15/12	103	1315.18354	608.85443	1874	2
N4	8/15/12	103	1296.9557	604.297468	1878	2

N4	8/15/12	103	1312.14557	619.487342	1883	2
N4	8/15/12	115	1243.79114	579.993671	3147	2
N4	8/15/12	115	1225.56329	484.297468	3201	2
N4	8/15/12	115	1301.51266	402.272152	3268	2
N4	8/15/12	115	1316.70253	408.348101	3285	2
N4	8/15/12	115	1309.1076	429.613924	3311	2
N4	8/15/12	115	1303.03165	422.018987	3335	2
N4	8/15/12	115	1292.39873	453.917722	3354	2
N4	8/15/12	115	1310.62658	441.765823	3361	2
N4	8/15/12	115	1313.66456	458.474684	3379	2
N4	8/15/12	115	1309.1076	463.031646	3382	2
N4	8/15/12	115	1315.18354	446.322785	3385	2
N4	8/15/12	115	1281.76582	443.28481	3393	2
N4	8/15/12	115	1278.72785	426.575949	3397	2
N4	8/15/12	115	1283.28481	406.829114	3406	2
N4	8/15/12	115	1313.66456	385.563291	3417	2
N4	8/15/12	115	1319.74051	399.234177	3426	2
N4	8/15/12	115	1312.14557	394.677215	3451	2
N4	8/15/12	115	1301.51266	384.044304	3441	2
N4	8/15/12	120	1271.13291	602.778481	3864	2
N4	8/15/12	120	1289.36076	543.537975	3875	2
N4	8/15/12	120	1313.66456	548.094937	3880	2
N4	8/15/12	120	92.398734	1120.75317	3881	2
N4	8/15/12	120	1306.06962	549.613924	3886	2
N4	8/15/12	120	1322.77848	558.727848	3911	2
N4	8/15/12	120	1353.15823	589.107595	3914	2
N4	8/15/12	120	1347.08228	485.816456	3946	2
N4	8/15/12	120	1359.23418	478.221519	3952	2
N4	8/15/12	120	1374.42405	478.221519	3959	2
N4	8/15/12	120	1375.94304	447.841772	3972	2
N4	8/15/12	120	1360.75317	435.689873	3985	2
N4	8/15/12	120	1378.98101	429.613924	4007	2
N4	8/15/12	120	1430.62658	396.196203	4030	2
N4	8/15/12	120	1435.18354	393.158228	4049	2
N4	8/15/12	120	1427.58861	349.107595	4068	2
N4	8/15/12	120	1448.85443	347.588608	4069	2
N4	8/15/12	120	1391.13291	330.879747	4109	2
N4	8/15/12	120	1400.24684	341.512658	4116	2
N4	8/15/12	120	1397.20886	368.85443	4150	2
N4	8/15/12	120	1363.79114	388.601266	4195	2
N4	8/15/12	120	1350.12025	373.411392	4203	2
N4	8/15/12	120	1327.33544	368.85443	4221	2

N4	8/15/12	120	1310.62658	377.968354	4240	2
N4	8/15/12	120	1315.18354	391.639241	4244	2
N4	8/15/12	120	1325.81646	382.525316	4251	2
N4	8/15/12	132	1258.98101	589.107595	5607	2
N4	8/15/12	132	1249.86709	569.360759	5619	2
N4	8/15/12	132	1289.36076	579.993671	5630	2
N4	8/15/12	132	1260.5	558.727848	5635	2
N4	8/15/12	132	1262.01899	540.5	5643	2
N4	8/15/12	132	1258.98101	519.234177	5649	2
N4	8/15/12	132	1266.57595	493.411392	5670	2
N4	8/15/12	132	1175.43671	560.246835	5711	2
N4	8/15/12	132	1160.24684	669.613924	5736	2
N4	8/15/12	132	1236.1962	722.778481	5768	2
N4	8/15/12	132	1183.03165	791.132911	5784	2
N4	8/15/12	132	1290.87975	757.71519	5902	2
N4	8/15/12	132	1026.57595	748.601266	6092	2
N4	8/15/12	132	1037.20886	713.664557	6104	2
N4	8/15/12	132	1064.55063	642.272152	6123	2
N4	8/15/12	132	1043.28481	602.778481	6149	2
N4	8/15/12	132	1040.24684	592.14557	6164	2
N4	8/15/12	132	1072.14557	551.132911	6184	2
N4	8/15/12	132	1020.5	543.537975	6198	2
N4	8/15/12	132	1102.52532	513.158228	6245	2
N4	8/15/12	132	1143.53798	418.981013	6268	2
N4	8/15/12	132	1204.29747	449.360759	6285	2
N4	8/15/12	132	1246.82911	432.651899	6301	2
N4	8/15/12	132	1289.36076	423.537975	6313	2
N4	8/15/12	132	1275.68987	409.867089	6324	2
N4	8/15/12	132	1301.51266	408.348101	6350	2
N4	8/15/12	132	1315.18354	390.120253	6402	2
N4	8/15/12	132	1324.29747	405.310127	6405	2
N4	8/15/12	132	1315.18354	402.272152	6406	2
N4	8/15/12	140	1251.38608	567.841772	6833	2
N4	8/15/12	140	1258.98101	584.550633	6835	2
N4	8/15/12	140	1262.01899	569.360759	6845	2
N4	8/15/12	140	1266.57595	522.272152	6851	2
N4	8/15/12	140	1262.01899	496.449367	6867	2
N4	8/15/12	140	1248.3481	516.196203	6868	2
N4	8/15/12	140	1158.72785	490.373418	6898	2
N4	8/15/12	140	1110.12025	532.905063	6915	2
N4	8/15/12	140	1094.93038	525.310127	6916	2
N4	8/15/12	140	1041.76582	461.512658	6939	2

N4	8/15/12	140	1085.81646	412.905063	6984	2
N4	8/15/12	140	1063.03165	399.234177	6986	2
N4	8/15/12	140	1142.01899	330.879747	7054	2
N4	8/15/12	140	1134.42405	347.588608	7066	2
N4	8/15/12	140	1125.31013	355.183544	7089	2
N4	8/15/12	140	1152.6519	336.955696	7100	2
N4	8/15/12	140	1152.6519	283.791139	7125	2
N4	8/15/12	140	1170.87975	247.335443	7168	2
N4	8/15/12	140	1227.08228	302.018987	7201	2
N4	8/15/12	140	1266.57595	370.373418	7232	2
N4	8/15/12	140	1280.24684	356.702532	7241	2
N4	8/15/12	140	1281.76582	370.373418	7244	2
N4	8/15/12	140	1322.77848	390.120253	7305	2
N4	8/15/12	140	1310.62658	370.373418	7311	2
N4	8/15/12	140	1322.77848	385.563291	7323	2
N13	8/13/12	1	1100.28723	255.180851	2229	1
N13	8/13/12	1	1076.03192	287.095745	2221	1
N13	8/13/12	1	1026.24468	296.031915	2206	1
N13	8/13/12	1	1018.58511	296.031915	2204	1
N13	8/13/12	1	1072.20213	330.5	2168	1
N13	8/13/12	1	1128.37234	377.734043	2144	1
N13	8/13/12	1	1148.79787	359.861702	2138	1
N13	8/13/12	1	1212.62766	335.606383	2113	1
N13	8/13/12	1	1240.71277	348.37234	2105	1
N13	8/13/12	1	1256.03192	339.43617	2098	1
N13	8/13/12	2	1041.56383	264.117021	1537	1
N13	8/13/12	2	1050.5	299.861702	1511	1
N13	8/13/12	2	1068.37234	304.968085	1474	1
N13	8/13/12	2	1129.64894	363.691489	1451	1
N13	8/13/12	2	1190.92553	322.840426	1430	1
N13	8/13/12	2	1187.09575	326.670213	1405	1
N13	8/13/12	2	1147.52128	349.648936	1392	1
N13	8/13/12	2	1167.94681	361.138298	1375	1
N13	8/13/12	2	1125.81915	386.670213	1352	1
N13	8/13/12	2	1138.58511	446.670213	1317	1
N13	8/13/12	2	1106.67021	477.308511	1300	1
N13	8/13/12	2	1141.1383	515.606383	1281	1
N13	8/13/12	2	1118.15957	504.117021	1293	1
N13	8/13/12	3	719.861702	270.5	4100	1
N13	8/13/12	3	616.457447	341.989362	4063	1
N13	8/13/12	3	682.840426	336.882979	4051	1

N13	8/13/12	3	675.180851	385.393617	4041	1
N13	8/13/12	3	746.670213	358.585106	4021	1
N13	8/13/12	3	753.053191	358.585106	4020	1
N13	8/13/12	3	754.329787	375.180851	4019	1
N13	8/13/12	4	639.43617	276.882979	4291	1
N13	8/13/12	4	1105.39362	469.648936	4126	1
N13	8/13/12	4	1118.15957	470.925532	4127	1
N13	8/13/12	4	1084.96809	447.946809	4140	1
N13	8/13/12	4	1042.84043	375.180851	4164	1
N13	8/13/12	4	1069.64894	352.202128	4170	1
N13	8/13/12	4	1042.84043	325.393617	4175	1
N13	8/13/12	4	1067.09575	312.62766	4181	1
N13	8/13/12	4	944.542553	384.117021	4218	1
N13	8/13/12	4	765.819149	298.585106	4238	1
N13	8/13/12	4	1141.1383	499.010638	4121	1
N13	8/13/12	4	751.776596	347.095745	4248	1
N13	8/13/12	4	1124.54255	509.223404	4124	1
N13	8/13/12	5	719.861702	275.606383	6155	1
N13	8/13/12	5	735.180851	278.159574	6151	1
N13	8/13/12	5	714.755319	276.882979	6144	1
N13	8/13/12	5	694.329787	308.797872	6136	1
N13	8/13/12	5	667.521277	334.329787	6124	1
N13	8/13/12	5	714.755319	345.819149	6103	1
N13	8/13/12	5	727.521277	361.138298	6094	1
N13	8/13/12	5	746.670213	357.308511	6085	1
N13	8/13/12	6	979.010638	261.56383	5229	1
N13	8/13/12	6	987.946809	250.074468	5223	1
N13	8/13/12	6	981.56383	287.095745	5214	1
N13	8/13/12	6	995.606383	293.478723	5204	1
N13	8/13/12	7	1127.09575	253.904255	4867	1
N13	8/13/12	7	1092.62766	279.43617	4856	1
N13	8/13/12	7	1086.24468	307.521277	4852	1
N13	8/13/12	7	1087.52128	308.797872	4839	1
N13	8/13/12	7	1124.54255	325.393617	4825	1
N13	8/13/12	7	1151.35106	362.414894	4815	1
N13	8/13/12	7	1165.39362	358.585106	4805	1
N13	8/13/12	7	1115.60638	375.180851	4793	1
N13	8/13/12	7	1141.1383	400.712766	4788	1
N13	8/13/12	7	1146.24468	506.670213	4762	1
N13	8/13/12	7	1142.41489	518.159574	4751	1
N13	8/13/12	7	1137.30851	528.37234	4749	1
N13	8/13/12	8	716.031915	279.43617	8414	1

N13	8/13/12	8	705.819149	296.031915	8410	1
N13	8/13/12	8	693.053191	274.329787	8411	1
N13	8/13/12	8	680.287234	316.457447	8407	1
N13	8/13/12	8	726.244681	330.5	8404	1
N13	8/13/12	8	718.585106	375.180851	8399	1
N13	8/13/12	8	727.521277	381.56383	8393	1
N13	8/13/12	9	881.989362	269.223404	8102	1
N13	8/13/12	9	911.351064	290.925532	8095	1
N13	8/13/12	9	957.308511	381.56383	8086	1
N13	8/13/12	9	976.457447	364.968085	8084	1
N13	8/13/12	9	1008.37234	380.287234	8081	1
N13	8/13/12	9	1068.37234	379.010638	8075	1
N13	8/13/12	9	1069.64894	326.670213	8060	1
N13	8/13/12	9	1026.24468	325.393617	8055	1
N13	8/13/12	9	1022.41489	303.691489	8052	1
N13	8/13/12	9	1016.03192	306.244681	8051	1
N13	8/13/12	10	1291.7766	244.968085	4308	1
N13	8/13/12	10	1272.62766	236.031915	4295	1
N13	8/13/12	10	1284.11702	246.244681	4287	1
N13	8/13/12	10	1267.52128	239.861702	4253	1
N13	8/13/12	10	1299.43617	248.797872	4252	1
N13	8/13/12	10	1279.01064	276.882979	4244	1
N13	8/13/12	10	1271.35106	297.308511	4230	1
N13	8/13/12	10	1291.7766	302.414894	4212	1
N13	8/13/12	10	1291.7766	319.010638	4210	1
N13	8/13/12	114	1287.84177	315.689873	3034	2
N13	8/13/12	114	1219.48734	292.905063	3005	2
N13	8/13/12	114	1221.00633	279.234177	2994	2
N13	8/13/12	114	1271.13291	274.677215	2934	2
N13	8/13/12	114	1257.46203	247.335443	2911	2
N13	8/13/12	114	1283.28481	259.487342	2884	2
N13	8/13/12	114	1342.52532	238.221519	2830	2
N13	8/13/12	114	1263.53798	250.373418	2784	2
N13	8/13/12	114	1255.94304	254.93038	2777	2
N13	8/13/12	114	1161.76582	286.829114	2736	2
N13	8/13/12	114	1126.82911	312.651899	2711	2
N13	8/13/12	114	1155.68987	312.651899	2709	2
N13	8/13/12	123	1148.09494	519.234177	4318	2
N13	8/13/12	123	1217.96835	605.816456	4293	2
N13	8/13/12	123	1290.87975	709.107595	4259	2
N13	8/13/12	123	1350.12025	737.968354	4249	2

N13	8/13/12	123	1310.62658	617.968354	4192	2
N13	8/13/12	123	1131.38608	573.917722	4169	2
N13	8/13/12	123	1110.12025	619.487342	4158	2
N13	8/13/12	123	1037.20886	631.639241	4145	2
N13	8/13/12	123	1072.14557	566.322785	4128	2
N13	8/13/12	123	1058.47468	356.702532	4076	2
N13	8/13/12	123	999.234177	306.575949	4061	2
N13	8/13/12	124	722.778481	350.626582	4575	2
N13	8/13/12	124	745.563291	336.955696	4558	2
N13	8/13/12	124	760.753165	349.107595	4540	2
N13	8/13/12	124	927.841772	321.765823	4501	2
N13	8/13/12	124	955.183544	291.386076	4486	2
N13	8/13/12	124	999.234177	405.310127	4430	2
N13	8/13/12	124	981.006329	428.094937	4404	2
N13	8/13/12	124	1014.42405	437.208861	4392	2
N13	8/13/12	124	952.14557	453.917722	4368	2
N13	8/13/12	124	943.031646	469.107595	4361	2
N13	8/13/12	124	1038.72785	493.411392	4330	2
N13	8/13/12	124	1015.94304	534.424051	4284	2
N13	8/13/12	124	1037.20886	505.563291	4238	2
N13	8/13/12	124	1128.3481	505.563291	4187	2
N13	8/13/12	124	1131.38608	538.981013	4183	2
N13	8/13/12	133	1161.76582	513.158228	5888	2
N13	8/13/12	133	1031.13291	470.626582	5849	2
N13	8/13/12	133	1031.13291	488.85443	5826	2
N13	8/13/12	133	798.727848	335.436709	5767	2
N13	8/13/12	133	797.208861	339.993671	5761	2
N13	8/13/12	133	777.462025	277.71519	5752	2
N13	8/13/12	133	762.272152	224.550633	5725	2
N13	8/13/12	133	791.132911	215.436709	5698	2
N13	8/13/12	133	777.462025	180.5	5694	2
N13	8/13/12	133	865.563291	156.196203	5678	2
N13	8/13/12	133	879.234177	218.474684	5670	2
N13	8/13/12	133	958.221519	232.14557	5644	2
N13	8/13/12	133	974.93038	215.436709	5641	2
N13	8/13/12	133	994.677215	195.689873	5628	2
N13	8/13/12	133	1006.82911	189.613924	5625	2
N13	8/13/12	133	990.120253	223.031646	5610	2
N13	8/13/12	133	950.626582	223.031646	5587	2
N13	8/13/12	133	944.550633	259.487342	5580	2
N13	8/13/12	133	977.968354	257.968354	5575	2
N13	8/13/12	133	967.335443	295.943038	5573	2

N13	8/13/12	147	712.14557	385.563291	8325	2
N13	8/13/12	147	639.234177	485.816456	8298	2
N13	8/13/12	147	604.297468	520.753165	8265	2
N13	8/13/12	147	584.550633	540.5	8259	2
N13	8/13/12	147	616.449367	549.613924	8253	2
N13	8/13/12	147	520.753165	687.841772	8215	2
N13	8/13/12	147	713.664557	768.348101	8124	2
N13	8/13/12	147	706.06962	721.259494	8078	2
N13	8/13/12	147	722.778481	703.031646	8070	2
N13	8/13/12	147	657.462025	551.132911	7987	2
N13	8/13/12	147	651.386076	423.537975	7944	2
N13	8/13/12	147	636.196203	384.044304	7919	2
N13	8/13/12	147	669.613924	333.917722	7900	2
N13	8/13/12	147	634.677215	333.917722	7887	2
N13	8/13/12	147	608.85443	323.28481	7883	2
N13	8/13/12	147	631.639241	309.613924	7882	2
N13	8/13/12	147	554.170886	262.525316	7867	2
N13	8/13/12	147	693.917722	224.550633	7787	2
N13	8/13/12	147	798.727848	122.778481	7763	2
N13	8/13/12	147	867.082278	207.841772	7736	2
N13	8/13/12	147	870.120253	226.06962	7733	2
N13	8/13/12	147	1000.75317	174.424051	7709	2
N13	8/13/12	147	1079.74051	241.259494	7690	2
N13	8/13/12	147	1087.33544	308.094937	7682	2
N13	8/13/12	147	1138.98101	270.120253	7652	2
N13	8/13/12	147	1120.75317	283.791139	7641	2
N13						
N13	8/14/12	1	464.443662	613.176056	1438	1
N13	8/14/12	1	476.274648	611.485915	1433	1
N13	8/14/12	1	506.697183	521.908451	1404	1
N13	8/14/12	1	513.457746	526.978873	1377	1
N13	8/14/12	1	596.274648	489.795775	1337	1
N13	8/14/12	1	616.556338	457.683099	1322	1
N13	8/14/12	1	638.528169	467.823944	1318	1
N13	8/14/12	1	626.697183	454.302817	1267	1
N13	8/14/12	1	621.626761	476.274648	1240	1
N13	8/14/12	1	641.908451	452.612676	1221	1
N13	8/14/12	2	486.415493	237.964789	1380	1
N13	8/14/12	2	625.007042	357.964789	1202	1
N13	8/14/12	2	665.570423	371.485915	1118	1
N13	8/14/12	2	679.091549	385.007042	1107	1
N13	8/14/12	2	635.147887	398.528169	1080	1

N13	8/14/12	2	648.669014	412.049296	1074	1
N13	8/14/12	3	452.314947	855.660142	2112	1
N13	8/14/12	3	484.770463	826.620996	2085	1
N13	8/14/12	3	604.343416	758.293594	2057	1
N13	8/14/12	3	599.218861	742.919929	2044	1
N13	8/14/12	3	694.877224	713.880783	2014	1
N13	8/14/12	3	682.919929	703.631673	1949	1
N13	8/14/12	3	727.33274	467.902135	1652	1
N13	8/14/12	3	667.546263	389.325623	1604	1
N13	8/14/12	3	655.588968	408.115658	1591	1
N13	8/14/12	3	614.592527	437.154804	1553	1
N13	8/14/12	3	594.094306	433.738434	1526	1
N13	8/14/12	3	592.386121	418.364769	1519	1
N13	8/14/12	3	544.55694	421.781139	1509	1
N13	8/14/12	4	469.396797	546.478648	1370	1
N13	8/14/12	4	489.895018	539.645907	1359	1
N13	8/14/12	4	493.311388	589.183274	1295	1
N13	8/14/12	4	525.766904	601.140569	1276	1
N13	8/14/12	4	508.685053	563.560498	1231	1
N13	8/14/12	4	566.763345	558.435943	1203	1
N13	8/14/12	4	527.475089	507.190391	1185	1
N13	8/14/12	4	590.677936	447.403915	1118	1
N13	8/14/12	4	568.47153	389.325623	1101	1
N13	8/14/12	4	626.549822	391.033808	1050	1
N13	8/14/12	4	621.425267	385.909253	985	1
N13	8/14/12	4	672.670819	428.613879	955	1
N13	8/14/12	5	466.985014	584.968665	5107	1
N13	8/14/12	5	514.069482	550.091281	5099	1
N13	8/14/12	5	543.715259	516.957766	5088	1
N13	8/14/12	5	576.848774	417.557221	5040	1
N13	8/14/12	5	594.287466	445.459128	5017	1
N13	8/14/12	5	550.690736	429.764305	5010	1
N13	8/14/12	6	465.241144	686.113079	3197	1
N13	8/14/12	6	479.192098	668.674387	3195	1
N13	8/14/12	6	498.374659	694.832425	3189	1
N13	8/14/12	6	514.069482	677.393733	3185	1
N13	8/14/12	7	466.985014	612.870572	5564	1
N13	8/14/12	7	514.069482	673.905995	5541	1
N13	8/14/12	7	559.410082	646.004087	5480	1
N13	8/14/12	7	575.104905	661.69891	5445	1
N13	8/14/12	7	650.091281	682.625341	5421	1
N13	8/14/12	7	629.16485	670.418256	5404	1

N13	8/14/12	7	552.434605	720.990463	5286	1
N13	8/14/12	7	543.715259	691.344687	5233	1
N13	8/14/12	8	465.566667	645.46	5636	1
N13	8/14/12	8	607.22	534.526667	5580	1
N13	8/14/12	8	610.633333	527.7	5569	1
N13	8/14/12	8	600.393333	529.406667	5564	1
N13	8/14/12	8	525.3	515.753333	5520	1
N13	8/14/12	8	518.473333	527.7	5517	1
N13	8/14/12	9	474.1	469.673333	6817	1
N13	8/14/12	9	489.46	456.02	6810	1
N13	8/14/12	9	547.486667	408.233333	6787	1
N13	8/14/12	9	550.9	391.166667	6784	1
N13	8/14/12	9	569.673333	420.18	6757	1
N13	8/14/12	9	608.926667	452.606667	6744	1
N13	8/14/12	9	581.62	478.206667	6738	1
N13	8/14/12	9	581.62	505.513333	6725	1
N13	8/14/12	9	590.153333	522.58	6712	1
N13	8/14/12	9	614.046667	544.766667	6705	1
N13	8/14/12	9	610.633333	481.62	6682	1
N13	8/14/12	9	644.766667	452.606667	6674	1
N13	8/14/12	10	465.566667	693.246667	6745	1
N13	8/14/12	10	494.58	676.18	6733	1
N13	8/14/12	10	501.406667	698.366667	6726	1
N13	8/14/12	10	538.953333	715.433333	6710	1
N13	8/14/12	10	545.78	691.54	6659	1
N13	8/14/12	131	651.386076	431.132911	259	2
N13	8/14/12	131	624.044304	426.575949	265	2
N13	8/14/12	131	554.170886	394.677215	316	2
N13	8/14/12	131	534.424051	349.107595	390	2
N13	8/14/12	131	522.272152	396.196203	409	2
N13	8/14/12	131	478.221519	441.765823	525	2
N13	8/14/12	131	505.563291	484.297468	569	2
N13	8/14/12	131	513.158228	472.14557	645	2
N13	8/14/12	134	528.348101	348.348101	1131	2
N13	8/14/12	134	488.85443	321.006329	1218	2
N13	8/14/12	134	497.968354	299.740506	1308	2
N13	8/14/12	134	520.753165	284.550633	1365	2
N13	8/14/12	134	532.905063	325.563291	1420	2
N13	8/14/12	135	528.348101	360.5	1434	2
N13	8/14/12	135	531.386076	369.613924	1453	2
N13	8/14/12	135	523.791139	412.14557	1542	2

N13	8/14/12	135	552.651899	427.335443	1577	2
N13	8/14/12	135	543.537975	439.487342	1581	2
N13	8/14/12	135	551.132911	441.006329	1617	2
N13	8/14/12	135	616.449367	447.082278	1642	2
N13	8/14/12	135	622.525316	415.183544	1660	2
N13	8/14/12	135	642.272152	445.563291	1672	2
N13	8/14/12	135	654.424051	442.525316	1705	2
N13	8/14/12	136	657.462025	439.487342	6038	2
N13	8/14/12	136	631.639241	439.487342	6016	2
N13	8/14/12	136	593.664557	437.968354	5999	2
N13	8/14/12	136	651.386076	444.044304	5983	2
N13	8/14/12	136	619.487342	744.803797	5879	2
N13	8/14/12	136	578.474684	711.386076	5829	2
N13	8/14/12	136	557.208861	722.018987	5784	2
N13	8/14/12	136	511.639241	720.5	5763	2
N13	8/14/12	136	482.778481	759.993671	5736	2
N13	8/14/12	136	361.259494	834.424051	5690	2
N13	8/14/12	136	291.386076	793.411392	5567	2
N13	8/14/12	136	282.272152	741.765823	5537	2
N13	8/14/12	136	385.563291	706.829114	5374	2
N13	8/14/12	136	400.753165	708.348101	5367	2
N13	8/14/12	136	406.829114	685.563291	5347	2
N13	8/14/12	136	377.968354	655.183544	5300	2
N13	8/14/12	136	429.613924	659.740506	5269	2
N13	8/14/12	136	493.411392	644.550633	5213	2
N13	8/14/12	136	545.056962	653.664557	5141	2
N13	8/14/12	136	557.208861	621.765823	5128	2
N13	8/14/12	136	514.677215	548.85443	5099	2
N13	8/14/12	136	551.132911	533.664557	5059	2
N13	8/14/12	136	511.639241	504.803797	5032	2
N13	8/14/12	136	510.120253	418.221519	4820	2
N13	8/14/12	136	494.93038	398.474684	4781	2
N13	8/14/12	136	526.829114	362.018987	4659	2
N13	8/14/12	136	532.905063	345.310127	4615	2
N13	8/14/12	137	546.575949	406.06962	8370	2
N13	8/14/12	137	551.132911	409.107595	8335	2
N13	8/14/12	137	537.462025	363.537975	8312	2
N13	8/14/12	137	545.056962	349.867089	8293	2
N13	8/14/12	137	538.981013	340.753165	8236	2
N13						
N13	8/15/12	1	1248.04502	332.120853	4315	1
N13	8/15/12	1	1235.30569	348.5	4306	1

N13	8/15/12	1	1144.31043	543.229858	4173	1
N13	8/15/12	2	1186.16825	303.00237	4116	1
N13	8/15/12	2	1131.57109	492.272512	3989	1
N13	8/15/12	2	1102.45261	501.372038	3980	1
N13	8/15/12	2	1084.25356	414.016588	3969	1
N13	8/15/12	2	1120.65166	408.556872	3964	1
N13	8/15/12	2	1117.01185	404.917062	3958	1
N13	8/15/12	2	1102.45261	381.258294	3938	1
N13	8/15/12	2	1078.79384	386.718009	3919	1
N13	8/15/12	2	1126.11137	495.912322	3894	1
N13	8/15/12	3	1186.16825	281.163507	4766	1
N13	8/15/12	3	1055.13507	323.021327	4740	1
N13	8/15/12	3	1140.67062	372.158768	4724	1
N13	8/15/12	3	1109.73223	434.035545	4706	1
N13	8/15/12	3	1095.17299	452.234597	4701	1
N13	8/15/12	3	1124.29147	463.154028	4697	1
N13	8/15/12	3	1122.47156	495.912322	4681	1
N13	8/15/12	4	1195.26777	364.879147	6037	1
N13	8/15/12	4	1173.42891	375.798578	6027	1
N13	8/15/12	4	1146.13033	392.177725	6010	1
N13	8/15/12	4	1151.59005	439.495261	5990	1
N13	8/15/12	4	1160.68957	459.514218	5983	1
N13	8/15/12	4	1138.85071	490.452607	5969	1
N13	8/15/12	5	1193.45868	328.070248	5722	1
N13	8/15/12	5	1197.02893	315.57438	5718	1
N13	8/15/12	5	1161.32645	340.566116	5705	1
N13	8/15/12	5	1123.83884	347.706612	5687	1
N13	8/15/12	5	1107.77273	347.706612	5683	1
N13	8/15/12	5	1054.21901	406.615702	5642	1
N13	8/15/12	5	1130.97934	511.938017	5530	1
N13	8/15/12	6	1197.02893	663.673554	4331	1
N13	8/15/12	6	1186.31818	663.673554	4312	1
N13	8/15/12	6	1168.46694	686.880165	4257	1
N13	8/15/12	6	1155.97107	658.318182	4239	1
N13	8/15/12	6	1123.83884	622.615702	4199	1
N13	8/15/12	6	1130.97934	619.045455	4169	1
N13	8/15/12	6	1107.77273	611.904959	4156	1
N13	8/15/12	6	1093.49174	595.838843	4144	1
N13	8/15/12	6	1095.27686	574.417355	4105	1
N13	8/15/12	6	1118.48347	572.632231	4046	1
N13	8/15/12	7	1186.31818	324.5	5206	1
N13	8/15/12	7	1172.03719	320.929752	5198	1

N13	8/15/12	7	1198.81405	349.491736	5182	1
N13	8/15/12	7	1179.17769	395.904959	5156	1
N13	8/15/12	7	1155.97107	453.028926	5142	1
N13	8/15/12	7	1107.77273	485.161157	5117	1
N13	8/15/12	7	1066.71488	549.42562	5092	1
N13	8/15/12	7	1088.13636	577.987603	5083	1
N13	8/15/12	7	1148.83058	619.045455	5041	1
N13	8/15/12	7	1118.48347	669.028926	4997	1
N13	8/15/12	8	1197.98691	244.58377	4362	1
N13	8/15/12	8	1220.60471	301.128272	4332	1
N13	8/15/12	8	1190.44764	314.32199	4326	1
N13	8/15/12	8	1190.44764	361.442408	4312	1
N13	8/15/12	8	1197.98691	429.295812	4299	1
N13	8/15/12	8	1132.01833	527.306283	4214	1
N13	8/15/12	8	1126.36387	593.274869	4182	1
N13	8/15/12	8	1145.21204	625.316754	4128	1
N13	8/15/12	8	1137.67278	649.819372	4122	1
N13	8/15/12	8	1122.59424	661.128272	4106	1
N13	8/15/12	9	1209.86877	471.430233	6359	1
N13	8/15/12	9	1200.89867	496.546512	6353	1
N13	8/15/12	9	1175.78239	491.164452	6347	1
N13	8/15/12	9	1156.04817	519.868771	6341	1
N13	8/15/12	9	1125.54983	600.599668	6315	1
N13	8/15/12	9	1123.75581	634.686047	6287	1
N13	8/15/12	9	1148.87209	654.420266	6278	1
N13	8/15/12	9	1121.96179	663.390365	6269	1
N13	8/15/12	10	1220.63289	229.237542	7839	1
N13	8/15/12	10	1165.01827	231.031561	7823	1
N13	8/15/12	10	1200.89867	256.147841	7807	1
N13	8/15/12	10	1197.31063	288.440199	7793	1
N13	8/15/12	10	1197.31063	313.556478	7772	1
N13	8/15/12	10	1186.54651	329.702658	7738	1
N13	8/15/12	10	1197.31063	410.433555	7711	1
N13	8/15/12	10	1184.75249	440.931894	7692	1
N13	8/15/12	10	1172.19435	464.254153	7673	1
N13	8/15/12	10	1123.75581	609.569767	7614	1
N13	8/15/12	10	1145.28405	598.805648	7582	1
N13	8/15/12	10	1173.98837	634.686047	7572	1
N13	8/15/12	10	1118.37375	656.214286	7557	1
N13	8/15/12	10	1111.19767	675.948505	7552	1
N13	8/15/12	100	1116.23034	660.5	1325	2

N13	8/15/12	100	1139.15169	556.679775	1204	2
N13	8/15/12	100	1129.71348	489.264045	1120	2
N13	8/15/12	100	993.533708	493.308989	1020	2
N13	8/15/12	100	1005.66854	506.792135	1008	2
N13	8/15/12	100	992.185393	504.095506	986	2
N13	8/15/12	100	1027.24157	545.893258	926	2
N13	8/15/12	100	1043.42135	533.758427	885	2
N13	8/15/12	100	1127.01685	454.207865	807	2
N13	8/15/12	100	1145.89326	491.960674	789	2
N13	8/15/12	101	1127.01685	691.511236	2586	2
N13	8/15/12	101	1155.33146	647.016854	2524	2
N13	8/15/12	101	1143.19663	624.095506	2486	2
N13	8/15/12	101	1108.14045	655.106742	2347	2
N13	8/15/12	102	1144.54494	489.264045	8779	2
N13	8/15/12	102	1182.29775	382.747191	8692	2
N13	8/15/12	102	1170.16292	630.837079	8252	2
N13	8/15/12	102	1144.54494	497.353933	8497	2
N13	8/15/12	102	1143.19663	606.567416	8292	2
N13	8/15/12	102	1120.27528	655.106742	8218	2
N13	8/15/12	103	1059.60112	657.803371	2923	2
N13	8/15/12	103	1033.98315	621.398876	2874	2
N13	8/15/12	103	1069.03933	638.926966	2846	2
N13	8/15/12	103	1109.48876	648.365169	2814	2
N13	8/15/12	103	1127.01685	665.893258	2799	2
N13	8/15/12	104	1067.69101	649.713483	6326	2
N13	8/15/12	104	1078.47753	647.016854	6299	2
N13	8/15/12	104	1081.17416	595.780899	6270	2
N13	8/15/12	104	1042.07303	580.949438	6199	2
N13	8/15/12	104	1116.23034	621.398876	6155	2
N13	8/15/12	104	1112.18539	671.286517	6095	2
