# Supplementary material for: Interactions Increase Forager Availability and Activity in Harvester Ants
Source: PLoS One. 2015 Nov 5;10(11):e0141971. doi: 10.1371/journal.pone.0141971 (PMC4635008; doi:10.1371/journal.pone.0141971)
Supplement: S3 Dataset — We observed and filmed behavior inside the nest during and after forager removals. This dataset shows our counts made from the films of the numbers of returning and outgoing foragers at the nest entrance and the number of ascending and descending ants at all tunnel entrances. (ZIP) [file pone.0141971.s004.zip › S3 Dataset/2013 Correlation Data 229 8-24.pdf]

Researcher **Jovel Queirolo**
Colony 229
8/24/13

**Video time (seconds)**	**Event**
2	Descend
6	Ascend
6	Descend
7	Ascend
8	Ascend
9	Descend
10	Ascend
12	Descend
13	Descend
14	Descend
14	Ascend
15	Ascend
17	Ascend
18	Ascend
18	Ascend
19	Ascend
20	Ascend
21	Descend
23	Descend
24	Ascend
24	Descend
24	Ascend
25	Ascend
26	Ascend
26	Ascend
27	Ascend
27	Descend
27	Ascend
28	Ascend
28	Descend
29	Ascend
29	Descend
29	Ascend
30	Descend
30	Descend
30	Descend
31	Ascend

31	Ascend
31	Ascend
32	Ascend
32	Ascend
32	Ascend
33	Ascend
33	Ascend
34	Ascend
34	Ascend
34	Descend
35	Descend
35	Descend
35	Ascend
36	Ascend
36	Descend
37	Descend
38	Descend
38	Descend
39	Descend
39	Descend
39	Descend
40	Descend
40	Descend
41	Ascend
41	Ascend
41	Descend
42	Ascend
43	Descend
43	Descend
44	Ascend
44	Ascend
45	Ascend
45	Ascend
46	Ascend
46	Ascend
46	Descend
47	Descend
47	Ascend
48	Ascend
48	Ascend
48	Ascend
49	Ascend

49	Ascend
50	Ascend
50	Descend
50	Descend
51	Descend
52	Descend
52	Descend
53	Ascend
53	Ascend
54	Ascend
54	Ascend
55	Ascend
56	Ascend
57	Ascend
58	Ascend
58	Descend
59	Descend
59	Descend
59	Descend
60	Descend
60	Descend
61	Descend
61	Descend
61	Ascend
62	Ascend
62	Ascend
63	Ascend
64	Ascend
64	Ascend
65	Ascend
65	Ascend
65	Ascend
65	Ascend
66	Ascend
66	Ascend
67	Descend
67	Ascend
67	Descend
68	Descend
68	Descend
69	Descend
69	Descend

69	Descend
69	Descend
70	Ascend
70	Ascend
70	Ascend
71	Ascend
71	Descend
71	Descend
72	Descend
72	Descend
72	Ascend
73	Ascend
73	Ascend
73	Ascend
74	Ascend
74	Ascend
74	Ascend
75	Ascend
78	Descend
78	Descend
78	Descend
79	Descend
79	Descend
79	Descend
80	Descend
80	Descend
80	Descend
81	Descend
81	Descend
82	Descend
82	Ascend
83	Ascend
83	Ascend
83	Ascend
86	Descend
86	Descend
86	Descend
87	Descend
87	Ascend
87	Ascend
88	Descend
88	Descend

89	Descend
89	Descend
89	Ascend
90	Ascend
90	Descend
90	Descend
91	Descend
92	Descend
92	Descend
93	Descend
94	Ascend
95	Descend
95	Ascend
96	Descend
97	Descend
97	Ascend
97	Ascend
98	Descend
98	Ascend
98	Ascend
99	Descend
99	Descend
99	Ascend
100	Ascend
100	Descend
101	Ascend
101	Ascend
101	Ascend
102	Ascend
102	Descend
102	Descend
103	Ascend
103	Descend
103	Descend
104	Ascend
104	Ascend
105	Ascend
105	Ascend
106	Ascend
106	Ascend
106	Ascend
107	Descend

107	Descend
108	Descend
108	Descend
108	Descend
109	Descend
109	Descend
110	Descend
110	Descend
110	Ascend
110	Ascend
111	Ascend
111	Ascend
111	Ascend
112	Ascend
112	Ascend
112	Descend
112	Descend
113	Ascend
113	Ascend
113	Descend
113	Descend
114	Descend
114	Descend
114	Descend
114	Descend
115	Descend
115	Descend
115	Descend
116	Ascend
116	Ascend
116	Ascend
117	Ascend
117	Descend
117	Descend
118	Ascend
118	Descend
119	Ascend
119	Ascend
120	Descend
120	Descend
120	Ascend
121	Ascend

121	Ascend
122	Ascend
122	Ascend
122	Ascend
123	Ascend
123	Ascend
124	Ascend
124	Ascend
125	Ascend
125	Ascend
125	Ascend
125	Ascend
126	Ascend
126	Ascend
127	Ascend
128	Ascend
129	Ascend
134	Ascend
134	Ascend
135	Descend
135	Descend
136	Descend
136	Descend
137	Descend
137	Descend
138	Descend
138	Descend
139	Descend
140	Descend
141	Descend
141	Descend
143	Ascend
143	Ascend
145	Descend
145	Descend
147	Descend
148	Descend
148	Descend
148	Descend
150	Descend
151	Descend
151	Descend

152	Descend
152	Descend
153	Descend
153	Descend
154	Descend
155	Descend
155	Descend
156	Descend
157	Descend
158	Descend
159	Descend
160	Descend
161	Descend
162	Descend
162	Descend
163	Descend
163	Descend
164	Descend
165	Descend
167	Descend
168	Descend
169	Descend
169	Ascend
169	Ascend
170	Ascend
170	Ascend
170	Ascend
171	Descend
173	Ascend
174	Descend
174	Descend
175	Descend
176	Descend
177	Descend
177	Ascend
177	Ascend
177	Ascend
178	Ascend
178	Ascend
178	Ascend
179	Ascend
179	Ascend

180	Ascend
181	Descend
182	Descend
182	Descend
183	Descend
183	Descend
184	Descend
184	Descend
185	Ascend
185	Ascend
185	Descend
186	Descend
187	Descend
188	Descend
190	Ascend
190	Ascend
191	Ascend
192	Ascend
193	Ascend
193	Ascend
194	Ascend
195	Ascend
196	Descend
196	Descend
197	Descend
198	Ascend
198	Descend
200	Descend
200	Descend
202	Descend
202	Descend
203	Descend
205	Ascend
206	Ascend
206	Ascend
207	Ascend
207	Ascend
207	Ascend
208	Ascend
209	Ascend
210	Ascend
210	Ascend

210	Ascend
211	Ascend
211	Ascend
212	Ascend
212	Ascend
212	Ascend
213	Ascend
213	Ascend
214	Ascend
216	Ascend
216	Descend
217	Descend
217	Descend
218	Ascend
219	Descend
219	Descend
220	Descend
221	Descend
221	Descend
222	Descend
223	Descend
224	Descend
224	Descend
224	Ascend
225	Ascend
225	Ascend
225	Ascend
226	Ascend
226	Ascend
227	Ascend
227	Ascend
228	Descend
229	Descend
229	Descend
230	Descend
230	Descend
231	Descend
231	Ascend
232	Ascend
232	Ascend
232	Ascend
233	Ascend

233	Ascend
234	Ascend
234	Ascend
235	Ascend
236	Ascend
237	Descend
237	Descend
237	Descend
238	Descend
239	Descend
239	Descend
240	Descend
242	Descend
242	Descend
243	Descend
245	Descend
246	Ascend
246	Ascend
247	Ascend
248	Ascend
249	Ascend
249	Ascend
251	Ascend
251	Descend
252	Descend
252	Descend
253	Descend
253	Ascend
253	Ascend
254	Descend
255	Ascend
256	Descend
258	Descend
258	Descend
258	Descend
259	Descend
259	Descend
260	Descend
260	Descend
260	Descend
261	Descend
261	Descend

261	Descend
263	Descend
263	Descend
264	Descend
265	Descend
265	Descend
265	Descend
266	Descend
266	Descend
267	Descend
267	Descend
268	Descend
268	Descend
269	Descend
269	Ascend
269	Ascend
270	Ascend
271	Ascend
271	Ascend
272	Ascend
272	Ascend
272	Descend
272	Descend
273	Descend
273	Descend
273	Descend
274	Descend
274	Descend
274	Descend
275	Descend
275	Descend
276	Descend
276	Descend
276	Descend
277	Descend
277	Descend
280	Ascend
282	Descend
283	Descend
283	Descend
284	Ascend
284	Ascend

285	Descend
286	Descend
286	Ascend
287	Ascend
288	Descend
288	Descend
289	Descend
290	Ascend
290	Ascend
291	Descend
291	Descend
292	Descend
293	Descend
293	Descend
294	Descend
294	Ascend
294	Ascend
295	Descend
295	Ascend
296	Ascend
296	Descend
297	Descend
298	Descend
298	Ascend
298	Ascend
298	Descend
299	Descend
300	Ascend
301	Ascend
301	Ascend
302	Ascend
302	Ascend
303	Ascend
303	Ascend
304	Ascend
305	Descend
305	Descend
306	Descend
306	Descend
307	Ascend
307	Ascend
308	Descend

308	Descend
312	Descend
313	Descend
313	Ascend
313	Ascend
314	Descend
314	Descend
315	Descend
315	Ascend
317	Ascend
319	Descend
320	Descend
320	Ascend
321	Ascend
321	Ascend
322	Ascend
322	Ascend
322	Ascend
323	Ascend
323	Ascend
323	Ascend
324	Ascend
324	Ascend
324	Ascend
328	Descend
328	Descend
329	Descend
330	Descend
330	Descend
331	Ascend
332	Descend
332	Ascend
333	Ascend
334	Ascend
334	Ascend
335	Ascend
335	Ascend
336	Ascend
336	Ascend
336	Ascend
336	Ascend
337	Ascend

337	Ascend
338	Ascend
338	Ascend
339	Ascend
339	Ascend
340	Ascend
340	Ascend
341	Ascend
342	Descend
343	Descend
343	Ascend
344	Ascend
344	Ascend
345	Ascend
346	Ascend
346	Ascend
348	Descend
354	Descend
354	Descend
356	Ascend
356	Ascend
357	Ascend
358	Ascend
359	Descend
363	Ascend
363	Descend
364	Descend
364	Descend
364	Descend
365	Descend
366	Descend
368	Ascend
368	Ascend
369	Ascend
370	Ascend
370	Ascend
370	Descend
371	Descend
372	Descend
373	Descend
373	Descend
373	Ascend

374	Ascend
374	Ascend
374	Ascend
374	Ascend
375	Ascend
375	Ascend
375	Ascend
376	Ascend
376	Ascend
376	Ascend
377	Ascend
377	Ascend
378	Ascend
378	Ascend
378	Ascend
379	Ascend
379	Ascend
380	Ascend
380	Ascend
381	Ascend
382	Ascend
383	Descend
384	Descend
384	Descend
385	Descend
386	Descend
386	Descend
387	Descend
387	Descend
388	Descend
388	Descend
389	Descend
389	Ascend
389	Ascend
390	Ascend
390	Ascend
391	Ascend
391	Ascend
392	Ascend
392	Ascend
392	Descend
393	Descend

393	Descend
393	Descend
394	Descend
395	Descend
395	Descend
396	Descend
396	Descend
397	Descend
397	Descend
397	Descend
398	Descend
398	Descend
399	Descend
399	Descend
399	Ascend
399	Ascend
400	Ascend
400	Ascend
400	Ascend
401	Ascend
401	Ascend
401	Ascend
402	Ascend
402	Ascend
403	Ascend
403	Ascend
403	Ascend
404	Ascend
404	Ascend
404	Ascend
405	Ascend
405	Ascend
405	Ascend
406	Ascend
406	Ascend
407	Ascend
407	Descend
407	Descend
407	Descend
408	Descend
408	Descend
409	Descend

409	Descend
409	Ascend
409	Ascend
410	Ascend
410	Descend
410	Descend
410	Ascend
411	Ascend
411	Descend
411	Ascend
411	Ascend
412	Ascend
412	Ascend
412	Ascend
412	Ascend
412	Ascend
413	Ascend
413	Ascend
413	Ascend
414	Ascend
414	Ascend
414	Ascend
415	Ascend
415	Ascend
415	Ascend
416	Ascend
416	Ascend
416	Ascend
417	Ascend
417	Ascend
417	Ascend
418	Descend
418	Descend
419	Descend
419	Descend
420	Descend
420	Descend
420	Descend
421	Descend
421	Descend
422	Descend
422	Descend

423	Descend
423	Descend
424	Descend
424	Descend
425	Descend
425	Ascend
425	Descend
426	Descend
426	Descend
427	Descend
427	Descend
428	Descend
428	Descend
429	Ascend
429	Descend
429	Ascend
430	Ascend
430	Ascend
430	Ascend
431	Ascend
431	Descend
432	Ascend
432	Ascend
433	Ascend
433	Descend
434	Descend
436	Descend
436	Descend
436	Descend
437	Descend
437	Descend
438	Descend
438	Descend
438	Descend
439	Descend
440	Descend
440	Descend
441	Descend
441	Descend
441	Descend
442	Descend
443	Descend

443	Descend
443	Descend
444	Descend
444	Descend
445	Descend
446	Ascend
446	Ascend
447	Ascend
447	Ascend
447	Ascend
448	Ascend
448	Descend
448	Descend
449	Ascend
449	Ascend
450	Descend
450	Descend
450	Descend
451	Ascend
451	Ascend
451	Descend
452	Descend
452	Descend
453	Descend
453	Descend
453	Descend
454	Descend
454	Descend
454	Descend
454	Descend
455	Descend
455	Descend
455	Descend
456	Descend
456	Descend
456	Descend
457	Descend
458	Descend
458	Descend
458	Descend
459	Descend
459	Descend

460	Descend
460	Descend
460	Descend
461	Descend
461	Descend
462	Descend
462	Descend
463	Descend
464	Descend
464	Descend
465	Descend
467	Descend
468	Descend
469	Descend
471	Descend
471	Ascend
472	Ascend
472	Ascend
473	Ascend
473	Ascend
473	Ascend
474	Ascend
475	Ascend
475	Ascend
475	Descend
476	Descend
476	Descend
477	Descend
478	Descend
478	Descend
478	Descend
479	Descend
479	Descend
480	Descend
481	Descend
482	Descend
483	Descend
484	Descend
485	Descend
486	Descend
487	Descend
488	Descend

488	Descend
489	Descend
489	Descend
490	Descend
490	Descend
491	Descend
492	Descend
493	Descend
494	Descend
495	Descend
496	Ascend
496	Ascend
497	Descend
497	Descend
498	Descend
499	Descend
500	Descend
501	Descend
502	Descend
504	Descend
507	Descend
508	Descend
508	Descend
510	Descend
511	Descend
514	Descend
515	Descend
517	Descend
519	Descend
521	Descend
523	Descend
524	Descend
525	Descend
526	Descend
527	Descend
529	Descend
530	Ascend
530	Ascend
531	Ascend
531	Ascend
531	Ascend
532	Ascend

532	Ascend
532	Descend
533	Descend
533	Ascend
534	Ascend
537	Descend
537	Ascend
539	Descend
541	Descend
543	Descend
548	Ascend
551	Ascend
552	Ascend
553	Ascend
554	Descend
556	Descend
558	Ascend
559	Ascend
561	Ascend
563	Descend
565	Descend
566	Descend
567	Descend
568	Ascend
569	Descend
571	Descend
572	Descend
574	Descend
576	Descend
577	Ascend
579	Descend
580	Descend
588	Descend
590	Descend
594	Ascend
594	Ascend
596	Ascend
599	Ascend
604	Descend
609	Ascend
610	Descend
615	Descend

632	Descend
645	Descend
653	Descend
656	Descend
659	Descend
666	Ascend
679	Descend
681	Descend
689	Descend
699	Ascend
699	Descend
703	Descend
703	Ascend
703	Ascend
704	Descend
705	Descend
706	Descend
706	Descend
707	Descend
710	Descend
717	Descend
718	Ascend
720	Descend
726	Ascend
730	Descend
732	Descend
734	Descend
737	Descend
739	Descend
740	Descend
748	Descend
749	Descend
752	Descend
753	Descend
756	Descend
758	Descend
758	Ascend
760	Descend
760	Ascend
762	Ascend
764	Descend
765	Ascend

768 Descend
769 Ascend
771 Descend
772 Ascend
774 Ascend
775 Descend
776 Descend
777 Descend
777 Ascend
781 Descend
783 Descend
783 Descend
789 Descend
789 Descend
795 Ascend
809 Descend
810 Descend
812 Descend
812 Descend
813 Ascend
814 Descend
817 Ascend
821 Descend
823 Ascend
828 Ascend
830 Ascend
831 Descend
833 Descend
835 Ascend
836 Ascend
837 Ascend
837 Ascend
838 Ascend
838 Ascend
841 Ascend
843 Ascend
846 Descend
848 Descend
848 Ascend
849 Ascend
849 Ascend
852 Descend

855	Descend
862	Descend
863	Descend
870	Descend
874	Descend
878	Descend
880	Descend
881	Ascend
883	Ascend
888	Descend
892	Descend
899	Ascend
904	Descend
909	Descend
910	Ascend
913	Descend
922	Ascend
923	Descend
925	Descend
930	Descend
931	Ascend
932	Ascend
932	Descend
933	Descend
934	Descend
935	Descend
936	Descend
936	Ascend
937	Ascend
937	Ascend
937	Ascend
938	Ascend
938	Ascend
938	Ascend
939	Ascend
939	Ascend
940	Ascend
941	Ascend
941	Ascend
941	Ascend
942	Ascend
943	Ascend

943	Ascend
943	Ascend
944	Ascend
944	Ascend
944	Ascend
944	Ascend
945	Ascend
945	Ascend
945	Ascend
946	Ascend
946	Ascend
946	Ascend
947	Descend
947	Descend
948	Ascend
948	Ascend
948	Ascend
948	Ascend
949	Ascend
949	Ascend
950	Ascend
950	Ascend
950	Ascend
951	Ascend
951	Ascend
951	Ascend
952	Ascend
952	Ascend
953	Ascend
953	Ascend
953	Ascend
954	Ascend
954	Ascend
955	Ascend
955	Ascend
956	Ascend
956	Ascend
956	Ascend
957	Ascend
957	Ascend
957	Ascend
958	Ascend

959	Ascend
959	Ascend
960	Ascend
960	Ascend
961	Ascend
962	Ascend
962	Ascend
962	Ascend
963	Ascend
963	Ascend
963	Ascend
964	Ascend
964	Ascend
964	Ascend
965	Ascend
965	Ascend
965	Ascend
965	Ascend
966	Ascend
966	Ascend
966	Ascend
967	Descend
967	Descend
968	Descend
968	Descend
968	Descend
969	Descend
969	Descend
970	Descend
970	Descend
970	Descend
971	Descend
971	Descend
972	Descend
972	Ascend
973	Ascend
973	Ascend
974	Ascend
974	Descend
975	Descend
976	Descend
976	Descend

976	Descend
976	Ascend
977	Ascend
977	Ascend
977	Ascend
978	Ascend
978	Ascend
978	Ascend
979	Ascend
979	Ascend
980	Ascend
980	Ascend
981	Ascend
981	Descend
981	Descend
982	Ascend
982	Ascend
983	Ascend
983	Ascend
983	Ascend
984	Ascend
984	Ascend
984	Ascend
985	Ascend
985	Descend
986	Descend
987	Descend
987	Descend
988	Ascend
988	Ascend
989	Ascend
989	Ascend
989	Ascend
990	Ascend
990	Ascend
991	Ascend
991	Ascend
991	Ascend
993	Descend
994	Descend
994	Descend
994	Descend

995	Descend
995	Descend
996	Descend
997	Descend
998	Descend
999	Descend
1000	Descend
1001	Descend
1002	Descend
1003	Descend
1003	Descend
1004	Descend
1005	Ascend
1006	Ascend
1006	Ascend
1006	Ascend
1007	Ascend
1007	Ascend
1009	Descend
1009	Descend
1010	Ascend
1010	Ascend
1010	Ascend
1011	Ascend
1011	Ascend
1012	Ascend
1013	Ascend
1013	Ascend
1013	Ascend
1014	Ascend
1014	Ascend
1015	Descend
1016	Descend
1017	Descend
1017	Descend
1017	Descend
1018	Descend
1018	Descend
1018	Descend
1019	Descend
1019	Descend
1020	Ascend

1020	Ascend
1020	Ascend
1021	Ascend
8	AntIn
9	AntOut
11	AntIn
13	AntIn
13	AntOut
14	AntOut
14	AntOut
17	AntIn
17	AntOut
19	AntIn
20	AntIn
21	AntIn
22	AntOut
22	AntOut
24	AntOut
24	AntOut
25	AntIn
26	AntOut
28	AntOut
28	AntOut
29	AntIn
29	AntIn
30	AntIn
31	AntOut
32	AntOut
33	AntIn
34	AntOut
35	AntOut
35	AntOut
36	AntOut
37	AntIn
37	AntOut
38	AntOut
39	AntOut
39	AntOut
39	AntOut
40	AntOut
40	AntOut
42	AntOut

44 AntOut
45 AntIn
46 AntOut
48 AntIn
49 AntOut
50 AntOut
52 AntIn
53 AntOut
57 AntOut
58 AntIn
59 AntIn
60 AntIn
60 AntOut
62 AntOut
63 AntIn
65 AntIn
65 AntIn
65 AntIn
66 AntIn
67 AntIn
68 AntOut
68 AntOut
69 AntOut
69 AntOut
70 AntOut
71 AntOut
72 AntOut
73 AntOut
73 AntOut
74 AntOut
75 AntIn
75 AntIn
76 AntOut
76 AntOut
76 AntOut
77 AntOut
78 AntOut
79 AntOut
79 AntOut
80 AntOut
80 AntIn
81 AntIn

81	AntIn
82	AntIn
82	AntOut
85	AntIn
86	AntIn
86	AntOut
86	AntOut
86	AntOut
87	AntOut
88	AntIn
89	AntOut
89	AntOut
90	AntIn
90	AntIn
91	AntOut
92	AntOut
93	AntOut
93	AntOut
93	AntOut
94	AntIn
95	AntOut
96	AntOut
98	AntIn
98	AntIn
98	AntOut
99	AntOut
100	AntIn
101	AntOut
102	AntIn
103	AntOut
105	AntOut
107	AntIn
107	AntIn
110	AntOut
110	AntOut
110	AntOut
111	AntOut
112	AntIn
112	AntIn
115	AntIn
115	AntOut
115	AntOut

115	AntOut
115	AntOut
116	AntOut
116	AntOut
116	AntOut
118	AntIn
118	AntIn
120	AntOut
120	AntIn
121	AntIn
122	AntOut
123	AntOut
125	AntOut
125	AntIn
126	AntOut
127	AntIn
128	AntOut
129	AntOut
130	AntIn
130	AntIn
132	AntIn
133	AntIn
134	AntOut
135	AntOut
139	AntIn
139	AntOut
139	AntOut
142	AntOut
142	AntOut
144	AntOut
144	AntOut
145	AntOut
146	AntOut
148	AntIn
150	AntIn
150	AntIn
151	AntIn
151	AntIn
153	AntIn
154	AntIn
155	AntIn
156	AntOut

157 AntIn
158 AntOut
159 AntOut
159 AntOut
162 AntIn
166 AntIn
168 AntOut
168 AntOut
169 AntOut
170 AntOut
171 AntIn
172 AntOut
173 AntOut
175 AntIn
178 AntOut
179 AntOut
179 AntOut
180 AntOut
181 AntOut
181 AntOut
182 AntOut
183 AntOut
185 AntIn
185 AntIn
185 AntIn
187 AntOut
187 AntIn
188 AntOut
189 AntIn
191 AntIn
193 AntOut
194 AntOut
194 AntOut
195 AntOut
195 AntOut
196 AntOut
196 AntOut
197 AntOut
199 AntIn
200 AntIn
201 AntIn
202 AntIn

203	AntOut
203	AntOut
203	AntOut
205	AntOut
207	AntIn
211	AntIn
211	AntOut
212	AntOut
213	AntOut
214	AntOut
214	AntOut
215	AntOut
215	AntOut
216	AntIn
217	AntIn
218	AntOut
219	AntIn
220	AntOut
221	AntOut
221	AntIn
222	AntIn
222	AntIn
223	AntOut
223	AntIn
224	AntIn
225	AntIn
227	AntOut
228	AntOut
228	AntOut
228	AntOut
229	AntOut
230	AntOut
230	AntOut
231	AntIn
232	AntIn
233	AntIn
233	AntIn
233	AntOut
234	AntOut
236	AntIn
238	AntIn
238	AntIn

239	AntIn
240	AntIn
240	AntIn
241	AntOut
244	AntIn
245	AntIn
246	AntOut
247	AntOut
247	AntOut
248	AntOut
248	AntIn
249	AntOut
250	AntOut
250	AntOut
252	AntIn
253	AntOut
254	AntOut
254	AntOut
255	AntOut
256	AntOut
256	AntOut
257	AntOut
258	AntIn
259	AntIn
259	AntIn
260	AntIn
261	AntOut
264	AntOut
267	AntIn
268	AntIn
269	AntIn
272	AntIn
273	AntIn
276	AntIn
276	AntOut
277	AntOut
277	AntOut
278	AntOut
279	AntOut
280	AntOut
281	AntIn
284	AntOut

284 AntOut
286 AntIn
287 AntOut
287 AntIn
288 AntOut
288 AntIn
289 AntIn
290 AntOut
291 AntIn
292 AntOut
293 AntOut
294 AntOut
294 AntIn
296 AntOut
297 AntOut
298 AntIn
299 AntOut
300 AntOut
300 AntOut
302 AntIn
304 AntOut
304 AntOut
304 AntOut
305 AntOut
308 AntOut
309 AntIn
309 AntIn
312 AntOut
312 AntIn
314 AntOut
315 AntOut
315 AntOut
317 AntOut
320 AntOut
321 AntIn
322 AntIn
325 AntOut
326 AntIn
326 AntIn
327 AntOut
327 AntOut
328 AntOut

328 AntOut
329 AntOut
330 AntOut
331 AntOut
333 AntIn
334 AntIn
335 AntOut
336 AntOut
336 AntOut
336 AntOut
337 AntOut
337 AntOut
337 AntOut
338 AntOut
339 AntOut
340 AntOut
341 AntOut
342 AntOut
342 AntOut
343 AntIn
343 AntIn
345 AntOut
346 AntOut
347 AntOut
347 AntIn
350 AntIn
351 AntOut
352 AntOut
353 AntIn
353 AntIn
353 AntOut
354 AntOut
355 AntIn
356 AntIn
356 AntIn
358 AntOut
365 AntIn
366 AntIn
366 AntOut
368 AntIn
368 AntOut
370 AntOut

371 AntIn
372 AntIn
372 AntIn
373 AntOut
374 AntOut
375 AntOut
376 AntOut
376 AntIn
377 AntIn
378 AntIn
378 AntOut
378 AntOut
380 AntOut
381 AntOut
382 AntOut
384 AntIn
385 AntIn
385 AntIn
387 AntIn
387 AntOut
388 AntOut
388 AntOut
391 AntOut
393 AntOut
395 AntIn
396 AntIn
396 AntIn
397 AntOut
397 AntOut
397 AntIn
400 AntOut
401 AntOut
401 AntOut
402 AntOut
402 AntIn
403 AntIn
403 AntIn
404 AntOut
406 AntOut
406 AntOut
407 AntOut
407 AntOut

407	AntOut
408	AntOut
408	AntOut
408	AntIn
411	AntIn
411	AntOut
412	AntOut
412	AntOut
412	AntOut
414	AntOut
414	AntIn
415	AntOut
415	AntOut
415	AntOut
416	AntOut
417	AntOut
417	AntOut
417	AntIn
419	AntIn
420	AntOut
424	AntIn
425	AntIn
425	AntOut
426	AntOut
427	AntIn
427	AntOut
428	AntIn
429	AntOut
430	AntOut
430	AntOut
431	AntOut
431	AntOut
434	AntIn
434	AntIn
435	AntIn
436	AntOut
437	AntIn
437	AntIn
437	AntIn
438	AntIn
469	AntIn
473	AntIn

474 AntOut
475 AntIn
476 AntIn
477 AntIn
477 AntIn
480 AntOut
480 AntOut
481 AntOut
481 AntOut
483 AntOut
483 AntIn
484 AntIn
485 AntOut
486 AntIn
486 AntIn
487 AntIn
487 AntIn
488 AntIn
492 AntIn
492 AntIn
493 AntIn
495 AntOut
495 AntOut
497 AntOut
501 AntIn
501 AntIn
502 AntIn
506 AntOut
508 AntOut
509 AntIn
511 AntOut
514 AntIn
515 AntIn
517 AntIn
522 AntIn
523 AntIn
526 AntIn
527 AntIn
529 AntOut
531 AntOut
532 AntIn
533 AntIn

536 AntIn
539 AntIn
544 AntIn
545 AntOut
547 AntOut
548 AntIn
549 AntIn
551 AntOut
552 AntOut
556 AntIn
564 AntOut
565 AntIn
566 AntIn
568 AntIn
569 AntIn
570 AntIn
571 AntIn
573 AntOut
575 AntIn
575 AntIn
576 AntIn
577 AntOut
579 AntOut
579 AntOut
580 AntIn
581 AntIn
581 AntOut
584 AntOut
589 AntOut
589 AntOut
594 AntIn
597 AntOut
602 AntIn
614 AntOut
614 AntIn
618 AntOut
619 AntOut
619 AntIn
620 AntIn
621 AntIn
622 AntOut
631 AntIn

636 AntOut
637 AntIn
638 AntIn
642 AntOut
644 AntIn
646 AntIn
650 AntOut
650 AntIn
654 AntOut
655 AntIn
657 AntOut
657 AntOut
658 AntIn
659 AntIn
659 AntOut
659 AntOut
662 AntIn
662 AntIn
662 AntIn
664 AntOut
665 AntIn
666 AntIn
667 AntIn
668 AntIn
671 AntIn
671 AntOut
673 AntIn
673 AntIn
675 AntIn
676 AntIn
679 AntIn
688 AntIn
692 AntIn
699 AntIn
703 AntOut
704 AntIn
704 AntIn
705 AntIn
706 AntIn
711 AntIn
711 AntIn
712 AntOut

714 AntIn
732 AntOut
732 AntIn
733 AntIn
737 AntIn
746 AntOut
749 AntIn
749 AntIn
750 AntIn
753 AntIn
755 AntOut
756 AntOut
756 AntOut
757 AntIn
758 AntIn
758 AntIn
761 AntIn
768 AntIn
770 AntIn
771 AntIn
776 AntOut
777 AntIn
779 AntOut
780 AntOut
780 AntOut
780 AntOut
782 AntIn
783 AntIn
784 AntIn
788 AntIn
789 AntIn
809 AntIn
809 AntIn
810 AntIn
812 AntIn
814 AntIn
820 AntIn
820 AntIn
823 AntIn
825 AntOut
826 AntIn
831 AntIn

834 AntIn
836 AntIn
838 AntOut
840 AntOut
841 AntOut
846 AntIn
849 AntOut
852 AntOut
852 AntOut
852 AntOut
853 AntOut
853 AntOut
856 AntIn
861 AntIn
863 AntIn
869 AntOut
871 AntIn
872 AntOut
874 AntIn
874 AntIn
879 AntIn
881 AntIn
881 AntIn
886 AntOut
888 AntIn
889 AntIn
898 AntIn
904 AntIn
912 AntIn
914 AntOut
918 AntIn
924 AntIn
927 AntIn
928 AntOut
929 AntIn
931 AntIn
933 AntIn
934 AntIn
936 AntIn
937 AntOut
937 AntOut
939 AntOut

939 AntOut
940 AntOut
941 AntIn
945 AntOut
954 AntOut
955 AntOut
957 AntOut
959 AntOut
960 AntOut
960 AntOut
961 AntOut
961 AntOut
962 AntOut
962 AntOut
963 AntOut
965 AntIn
966 AntOut
966 AntOut
967 AntOut
967 AntOut
968 AntOut
969 AntOut
969 AntOut
970 AntOut
972 AntOut
973 AntOut
975 AntIn
976 AntIn
977 AntIn
978 AntIn
978 AntOut
980 AntOut
981 AntOut
982 AntOut
983 AntIn
984 AntOut
985 AntOut
986 AntOut
986 AntOut
987 AntOut
988 AntOut
989 AntOut

989	AntOut
990	AntOut
990	AntOut
992	AntIn
993	AntIn
994	AntOut
995	AntOut
996	AntIn
996	AntIn
996	AntOut
998	AntIn
999	AntOut
999	AntIn
1001	AntOut
1004	AntIn
1004	AntIn
1008	AntIn
1009	AntOut
1010	AntIn
1011	AntIn
1011	AntOut
1013	AntOut
1014	AntOut
1014	AntOut
1015	AntIn
1016	AntIn
1017	AntIn
1018	AntIn
