# Supplementary material for: Interactions Increase Forager Availability and Activity in Harvester Ants
Source: PLoS One. 2015 Nov 5;10(11):e0141971. doi: 10.1371/journal.pone.0141971 (PMC4635008; doi:10.1371/journal.pone.0141971)
Supplement: S3 Dataset — We observed and filmed behavior inside the nest during and after forager removals. This dataset shows our counts made from the films of the numbers of returning and outgoing foragers at the nest entrance and the number of ascending and descending ants at all tunnel entrances. (ZIP) [file pone.0141971.s004.zip › S3 Dataset/2013 Correlation Data 229 8-25.pdf]

**Researcher**	**Jovel Queirolo**
**Colony 229**	
**8/25/13**	
**Video time (seconds)**	**Event**
12	Descend
23	Descend
33	Descend
36	Descend
48	Descend
53	Descend
54	Descend
64	Descend
72	Descend
76	Descend
79	Ascend
85	Descend
87	Ascend
88	Ascend
91	Ascend
104	Descend
129	Descend
137	Descend
146	Descend
148	Descend
188	Descend
212	Descend
213	Ascend
214	Ascend
218	Descend
221	Descend
235	Descend
240	Ascend
240	Ascend
251	Descend
256	Ascend
259	Descend
261	Descend
261	Descend
262	Ascend
267	Ascend
270	Ascend

276	Ascend
280	Descend
283	Descend
285	Ascend
287	Descend
293	Descend
295	Ascend
303	Ascend
306	Descend
314	Ascend
317	Descend
327	Ascend
335	Ascend
341	Descend
343	Ascend
346	Ascend
348	Ascend
349	Ascend
350	Ascend
358	Descend
362	Descend
363	Descend
364	Ascend
365	Ascend
368	Ascend
370	Ascend
371	Ascend
375	Descend
377	Ascend
378	Ascend
381	Ascend
383	Descend
385	Descend
386	Descend
387	Ascend
387	Ascend
388	Ascend
388	Ascend
389	Ascend
391	Ascend
391	Ascend
392	Ascend

393	Ascend
394	Ascend
398	Ascend
400	Ascend
400	Ascend
402	Ascend
403	Ascend
405	Ascend
408	Ascend
410	Ascend
413	Ascend
415	Ascend
422	Descend
423	Descend
423	Descend
424	Descend
426	Descend
426	Descend
430	Ascend
432	Ascend
433	Ascend
435	Ascend
437	Ascend
438	Ascend
439	Ascend
443	Ascend
445	Descend
452	Ascend
455	Ascend
459	Ascend
462	Descend
463	Ascend
464	Ascend
465	Ascend
466	Ascend
467	Ascend
468	Ascend
471	Ascend
473	Ascend
474	Ascend
477	Ascend
477	Ascend

479	Descend
480	Descend
481	Ascend
482	Descend
484	Ascend
487	Ascend
489	Descend
491	Ascend
491	Ascend
493	Ascend
494	Ascend
496	Ascend
499	Ascend
501	Ascend
503	Ascend
507	Descend
513	Ascend
521	Ascend
525	Descend
527	Descend
527	Descend
529	Descend
530	Ascend
533	Descend
533	Ascend
535	Ascend
536	Descend
537	Descend
538	Ascend
541	Ascend
544	Descend
546	Descend
547	Descend
547	Ascend
548	Ascend
548	Ascend
550	Ascend
550	Ascend
551	Ascend
551	Ascend
553	Descend
553	Descend

554	Descend
555	Ascend
555	Ascend
557	Ascend
558	Ascend
561	Ascend
561	Ascend
562	Ascend
563	Ascend
563	Ascend
564	Ascend
564	Ascend
565	Ascend
568	Ascend
569	Descend
572	Descend
572	Descend
575	Descend
577	Ascend
578	Ascend
578	Ascend
578	Ascend
579	Ascend
579	Ascend
580	Ascend
582	Ascend
583	Ascend
587	Ascend
590	Ascend
591	Descend
592	Descend
593	Ascend
594	Ascend
594	Ascend
595	Ascend
595	Ascend
596	Ascend
597	Ascend
598	Ascend
599	Ascend
600	Ascend
603	Ascend

603	Descend
605	Descend
606	Ascend
607	Descend
610	Descend
611	Ascend
611	Ascend
611	Ascend
612	Ascend
612	Ascend
613	Ascend
613	Ascend
614	Ascend
615	Ascend
615	Ascend
616	Descend
616	Descend
617	Descend
618	Ascend
618	Descend
619	Ascend
620	Descend
621	Descend
622	Ascend
622	Descend
622	Descend
623	Descend
623	Descend
625	Descend
625	Descend
626	Descend
627	Ascend
629	Ascend
629	Ascend
629	Ascend
630	Ascend
630	Ascend
631	Ascend
632	Ascend
633	Ascend
635	Ascend
636	Ascend

636 Ascend
637 Ascend
638 Ascend
640 Ascend
641 Ascend
641 Ascend
642 Ascend
644 Ascend
644 Ascend
645 Ascend
645 Ascend
647 Ascend
649 Ascend
649 Ascend
650 Ascend
651 Ascend
651 Ascend
653 Ascend
654 Ascend
654 Ascend
655 Ascend
655 Ascend
657 Descend
658 Descend
658 Descend
659 Descend
660 Descend
660 Ascend
661 Ascend
661 Ascend
662 Ascend
663 Ascend
665 Descend
665 Descend
668 Descend
668 Ascend
669 Descend
671 Descend
673 Descend
674 Descend
675 Descend
676 Ascend

676	Ascend
677	Ascend
679	Ascend
681	Ascend
681	Ascend
682	Ascend
683	Ascend
683	Ascend
684	Ascend
684	Ascend
684	Ascend
685	Ascend
685	Ascend
685	Ascend
686	Ascend
686	Ascend
686	Ascend
687	Ascend
687	Ascend
689	Ascend
689	Ascend
691	Ascend
692	Ascend
693	Descend
693	Descend
695	Descend
696	Descend
697	Descend
698	Descend
699	Ascend
703	Descend
705	Descend
708	Descend
709	Ascend
710	Ascend
711	Ascend
712	Ascend
712	Ascend
712	Ascend
713	Ascend
713	Ascend
714	Ascend

714	Ascend
716	Ascend
717	Descend
717	Descend
718	Descend
718	Descend
719	Ascend
720	Ascend
721	Descend
722	Ascend
723	Descend
724	Ascend
725	Ascend
727	Descend
728	Descend
729	Descend
731	Descend
731	Ascend
732	Ascend
734	Ascend
734	Ascend
735	Ascend
736	Ascend
737	Ascend
739	Ascend
742	Ascend
751	Ascend
752	Descend
753	Descend
754	Descend
756	Descend
756	Ascend
757	Ascend
757	Ascend
757	Ascend
758	Ascend
758	Ascend
758	Ascend
759	Ascend
760	Ascend
760	Ascend
761	Ascend

762	Ascend
762	Ascend
762	Ascend
763	Ascend
763	Ascend
765	Ascend
765	Ascend
766	Ascend
767	Ascend
767	Ascend
768	Descend
769	Descend
770	Descend
771	Descend
772	Descend
774	Descend
776	Descend
776	Ascend
776	Ascend
778	Ascend
778	Ascend
779	Ascend
780	Ascend
781	Ascend
782	Ascend
783	Ascend
783	Ascend
784	Ascend
784	Ascend
785	Ascend
787	Ascend
787	Ascend
788	Ascend
789	Ascend
789	Ascend
791	Ascend
792	Ascend
793	Ascend
794	Ascend
794	Ascend
796	Ascend
796	Ascend

797	Ascend
799	Ascend
799	Ascend
800	Ascend
800	Ascend
801	Ascend
803	Ascend
803	Descend
804	Descend
805	Descend
806	Descend
806	Descend
807	Descend
807	Descend
808	Descend
809	Descend
811	Descend
813	Ascend
813	Ascend
813	Ascend
814	Ascend
814	Ascend
815	Ascend
816	Ascend
817	Ascend
817	Ascend
818	Ascend
819	Ascend
819	Ascend
819	Descend
820	Descend
820	Ascend
821	Ascend
822	Ascend
822	Ascend
824	Ascend
825	Ascend
827	Ascend
830	Ascend
830	Ascend
832	Ascend
834	Ascend

834 Ascend
835 Ascend
835 Ascend
836 Ascend
838 Ascend
838 Ascend
839 Ascend
846 Ascend
847 Descend
847 Descend
848 Ascend
849 Ascend
849 Ascend
852 Descend
852 Descend
853 Ascend
853 Ascend
854 Ascend
855 Ascend
857 Ascend
858 Ascend
858 Ascend
859 Ascend
860 Ascend
862 Descend
864 Descend
864 Descend
865 Descend
866 Descend
867 Descend
868 Descend
870 Descend
871 Descend
872 Descend
873 Ascend
875 Ascend
876 Ascend
877 Ascend
878 Ascend
879 Ascend
881 Ascend
882 Descend

883	Descend
884	Descend
884	Descend
885	Ascend
886	Ascend
887	Ascend
889	Ascend
889	Ascend
890	Descend
890	Descend
891	Descend
892	Ascend
892	Ascend
893	Ascend
894	Descend
895	Descend
895	Ascend
896	Ascend
896	Descend
896	Descend
897	Ascend
897	Ascend
899	Ascend
899	Ascend
899	Ascend
901	Ascend
901	Descend
902	Descend
903	Descend
903	Descend
904	Ascend
904	Ascend
905	Ascend
908	Descend
910	Ascend
910	Ascend
911	Ascend
911	Descend
912	Descend
913	Descend
913	Ascend
915	Ascend

915	Ascend
916	Ascend
916	Ascend
917	Ascend
917	Ascend
919	Ascend
920	Ascend
922	Ascend
923	Descend
924	Descend
924	Descend
925	Descend
925	Descend
926	Ascend
926	Ascend
927	Ascend
927	Ascend
929	Descend
929	Descend
931	Descend
932	Descend
933	Ascend
933	Ascend
934	Ascend
935	Ascend
935	Ascend
936	Ascend
937	Descend
937	Descend
938	Descend
938	Descend
940	Descend
940	Descend
942	Descend
942	Descend
942	Ascend
943	Ascend
944	Ascend
944	Ascend
945	Ascend
945	Ascend
947	Ascend

948	Descend
948	Descend
949	Descend
949	Descend
949	Descend
950	Descend
950	Descend
950	Descend
951	Descend
952	Descend
952	Descend
954	Descend
956	Descend
958	Ascend
959	Ascend
960	Ascend
962	Ascend
962	Ascend
962	Ascend
963	Ascend
963	Ascend
963	Ascend
964	Ascend
964	Ascend
964	Ascend
965	Ascend
965	Ascend
966	Ascend
967	Ascend
967	Ascend
969	Ascend
969	Ascend
971	Descend
971	Ascend
971	Descend
972	Descend
973	Ascend
973	Ascend
974	Descend
974	Descend
975	Descend
975	Descend

976	Descend
976	Descend
977	Descend
977	Descend
979	Ascend
979	Ascend
979	Ascend
980	Ascend
980	Ascend
980	Descend
981	Descend
981	Descend
982	Descend
982	Descend
983	Ascend
983	Ascend
984	Ascend
984	Ascend
984	Ascend
984	Ascend
985	Ascend
985	Ascend
986	Ascend
986	Ascend
987	Ascend
987	Descend
987	Descend
988	Ascend
988	Ascend
988	Ascend
989	Descend
989	Descend
989	Ascend
990	Ascend
990	Descend
990	Descend
991	Descend
991	Descend
991	Ascend
992	Ascend
992	Descend
992	Descend

992	Descend
993	Descend
993	Descend
993	Ascend
994	Ascend
994	Ascend
995	Descend
995	Descend
995	Ascend
995	Ascend
996	Ascend
996	Ascend
997	Ascend
997	Ascend
997	Ascend
998	Ascend
998	Ascend
998	Ascend
999	Ascend
999	Descend
999	Descend
1000	Descend
1000	Descend
1000	Descend
1001	Ascend
1001	Ascend
1001	Ascend
1002	Descend
1002	Ascend
1002	Descend
1002	Ascend
1002	Ascend
1003	Ascend
1003	Descend
1003	Ascend
1003	Descend
1003	Descend
1004	Descend
1004	Ascend
1005	Descend
1007	Ascend
1008	Ascend

1008	Ascend
1009	Ascend
1009	Ascend
1010	Ascend
1010	Ascend
1011	Ascend
1012	Ascend
1013	Ascend
1013	Ascend
1013	Ascend
1013	Ascend
1014	Ascend
1014	Ascend
1015	Ascend
1016	Ascend
1016	Ascend
1016	Ascend
1016	Ascend
1017	Ascend
1017	Ascend
1018	Ascend
1018	Ascend
1019	Ascend
1020	Ascend
1021	Ascend
1021	Ascend
1021	Ascend
1022	Ascend
1022	Ascend
1022	Ascend
1023	Ascend
1023	Ascend
1024	Ascend
1024	Ascend
1025	Descend
1025	Descend
1025	Descend
1026	Descend
1026	Descend
1027	Ascend
1027	Ascend
1027	Descend

1027	Descend
1028	Ascend
1028	Ascend
1028	Ascend
1028	Ascend
1029	Ascend
1029	Ascend
1030	Ascend
1030	Ascend
1030	Ascend
1031	Ascend
1031	Ascend
1032	Descend
1032	Descend
1032	Descend
1032	Descend
1033	Descend
1034	Ascend
1034	Ascend
1034	Descend
1035	Descend
1035	Descend
1036	Ascend
1036	Ascend
1036	Descend
1037	Descend
1038	Descend
1039	Descend
1040	Ascend
1040	Ascend
1042	Ascend
1043	Descend
1043	Descend
1043	Descend
1044	Descend
1044	Descend
1045	Descend
1046	Ascend
1046	Ascend
1047	Ascend
1047	Ascend
1048	Descend

1048	Descend
1049	Ascend
1049	Ascend
1050	Descend
1050	Descend
1050	Ascend
1051	Ascend
1051	Descend
1051	Descend
1051	Descend
1052	Descend
1053	Descend
1053	Ascend
1054	Ascend
1054	Ascend
1055	Descend
1056	Ascend
1057	Ascend
1057	Ascend
1058	Descend
1058	Descend
1059	Descend
1060	Ascend
1061	Ascend
1061	Ascend
1062	Ascend
1062	Ascend
1063	Ascend
1064	Descend
1065	Descend
1065	Ascend
1065	Ascend
1066	Descend
1066	Ascend
1067	Ascend
1067	Ascend
1068	Ascend
1069	Ascend
1070	Ascend
1071	Descend
1072	Descend
1072	Descend

1075	Descend
1075	Descend
1076	Descend
1077	Descend
1077	Descend
1078	Descend
1079	Descend
1079	Descend
1080	Descend
1080	Descend
1081	Descend
1081	Descend
1082	Descend
1082	Descend
1083	Descend
1084	Descend
1084	Ascend
1084	Ascend
1085	Ascend
1085	Ascend
1086	Ascend
1086	Ascend
1087	Ascend
1088	Ascend
1088	Ascend
1089	Ascend
1089	Ascend
1090	Ascend
1090	Ascend
1091	Ascend
1091	Ascend
1092	Ascend
1092	Ascend
1093	Ascend
1094	Ascend
1095	Descend
1095	Descend
1095	Ascend
1096	Ascend
1097	Ascend
1097	Ascend
1098	Descend

1098	Descend
1099	Descend
1100	Descend
1100	Ascend
1101	Ascend
1101	Ascend
1102	Ascend
1102	Ascend
1102	Ascend
1102	Ascend
1103	Ascend
1103	Ascend
1104	Ascend
1104	Ascend
1105	Ascend
1105	Ascend
1105	Ascend
1106	Ascend
1106	Ascend
1107	Descend
1107	Descend
1107	Ascend
1108	Ascend
1108	Descend
1108	Descend
1109	Ascend
1109	Ascend
1110	Ascend
1110	Descend
1110	Descend
1111	Ascend
1111	Ascend
1112	Descend
1112	Descend
1113	Ascend
1113	Ascend
1114	Ascend
1114	Ascend
1114	Ascend
1115	Ascend
1115	Ascend
1116	Ascend

1116	Ascend
1117	Ascend
1118	Ascend
1118	Ascend
1119	Ascend
1120	Ascend
1120	Descend
1121	Descend
1121	Descend
1122	Descend
1122	Descend
1122	Descend
1123	Descend
1123	Ascend
1123	Ascend
1124	Descend
1124	Descend
1125	Ascend
1125	Ascend
1126	Ascend
1126	Descend
1126	Descend
1126	Ascend
1126	Ascend
1127	Ascend
1127	Ascend
1127	Ascend
1128	Ascend
1128	Ascend
1128	Ascend
1129	Ascend
1129	Ascend
1129	Descend
1129	Descend
1130	Descend
1130	Descend
1130	Ascend
1130	Ascend
1131	Ascend
1131	Ascend
1132	Ascend
1132	Ascend

1132	Ascend
1132	Ascend
1132	Ascend
1133	Ascend
1133	Ascend
1133	Ascend
1134	Ascend
1134	Ascend
1134	Ascend
1134	Ascend
1135	Ascend
1135	Ascend
1136	Ascend
1136	Ascend
1136	Ascend
1136	Ascend
1136	Ascend
1137	Ascend
1137	Ascend
1137	Ascend
1137	Ascend
1138	Ascend
1138	Ascend
1138	Ascend
1138	Ascend
1139	Descend
1139	Descend
1139	Ascend
1139	Ascend
1140	Ascend
1140	Ascend
1140	Ascend
1140	Ascend
1141	Ascend
1141	Ascend
1141	Ascend
1141	Ascend
1142	Ascend
1142	Descend
1142	Descend
1142	Descend
1143	Ascend

1143	Ascend
1143	Ascend
1143	Ascend
1143	Descend
1144	Descend
1144	Ascend
1144	Ascend
1144	Descend
1144	Descend
1145	Ascend
1145	Ascend
1145	Descend
1145	Descend
1145	Ascend
1145	Descend
1146	Descend
1146	Ascend
1146	Descend
1146	Ascend
1146	Descend
1146	Ascend
1147	Ascend
1147	Ascend
1148	Ascend
1148	Ascend
1149	Ascend
1149	Ascend
1149	Ascend
1150	Ascend
1150	Ascend
1150	Descend
1151	Descend
1151	Descend
1151	Ascend
1151	Ascend
1152	Descend
1152	Ascend
1152	Ascend
1153	Descend
1153	Descend
1154	Ascend
1154	Ascend

1154 Ascend
1155 Descend
1156 Descend
1156 Descend
1156 Descend
1156 Descend
1157 Descend
1157 Descend
1157 Descend
1158 Descend
1158 Descend
1159 Descend
1159 Descend
1159 Descend
1160 Descend
1160 Descend
1161 Descend
1161 Ascend
1161 Ascend
1161 Ascend
1162 Ascend
1162 Ascend
1162 Ascend
1163 Descend
1163 Descend
1163 Ascend
1163 Ascend
1164 Ascend
1164 Ascend
1164 Ascend
1164 Ascend
1165 Ascend
1165 Descend
1165 Descend
1165 Descend
1166 Descend
1166 Descend
1166 Descend
1166 Descend
1166 Ascend
1167 Ascend
1167 Ascend

1167	Ascend
1167	Ascend
1167	Descend
1168	Descend
1168	Ascend
1168	Ascend
1168	Ascend
1168	Ascend
1169	Ascend
1169	Ascend
1169	Ascend
1169	Ascend
1170	Ascend
1170	Ascend
1170	Ascend
1170	Descend
1170	Descend
1171	Ascend
1171	Ascend
1171	Ascend
1171	Ascend
1172	Descend
1172	Descend
1172	Descend
1172	Descend
1173	Descend
1173	Descend
1173	Descend
1173	Descend
1174	Descend
1174	Descend
1174	Ascend
1175	Ascend
1175	Ascend
1175	Ascend
1175	Ascend
1175	Ascend
1176	Ascend
1176	Ascend
1176	Ascend
1176	Ascend
1176	Descend

1177	Descend
1177	Descend
1177	Ascend
1177	Ascend
1178	Ascend
1178	Descend
1178	Descend
1178	Descend
1178	Descend
1179	Descend
1179	Descend
1179	Descend
1179	Descend
1180	Ascend
1180	Ascend
1180	Ascend
1180	Ascend
1181	Ascend
1181	Ascend
1181	Ascend
1181	Descend
1181	Descend
1182	Descend
1182	Descend
1182	Descend
1182	Descend
1183	Ascend
1183	Ascend
1183	Descend
1183	Descend
1184	Descend
1184	Descend
1184	Descend
1184	Ascend
1184	Ascend
1185	Ascend
1185	Ascend
1186	Ascend
1186	Ascend
1186	Ascend
1186	Ascend
1187	Descend

1187	Descend
1187	Descend
1187	Descend
1187	Descend
1187	Descend
1188	Descend
1188	Descend
1188	Descend
1188	Descend
1189	Descend
1189	Descend
1189	Descend
1189	Descend
1190	Descend
1190	Descend
1190	Ascend
1190	Ascend
1191	Ascend
1191	Ascend
1191	Ascend
1191	Descend
1192	Descend
1192	Descend
1192	Descend
1192	Descend
1193	Descend
1193	Descend
1193	Descend
1193	Descend
1194	Descend
1194	Descend
1194	Descend
1194	Descend
1194	Descend
1195	Descend
1195	Descend
1195	Descend
1195	Descend
1196	Descend
1196	Descend
1196	Descend
1197	Descend

1197	Descend
1197	Descend
1197	Descend
1198	Descend
1198	Descend
1198	Descend
1199	Descend
1199	Descend
1199	Descend
1200	Descend
1200	Descend
1200	Ascend
1201	Ascend
1201	Ascend
1201	Ascend
1202	Ascend
1202	Ascend
1202	Ascend
1202	Ascend
1203	Ascend
1203	Ascend
1203	Ascend
1204	Ascend
1204	Ascend
1204	Ascend
1204	Ascend
1205	Ascend
1205	Ascend
1205	Ascend
1206	Ascend
1206	Ascend
1206	Ascend
1207	Descend
1207	Descend
1207	Descend
1207	Descend
1207	Descend
1208	Descend
1208	Descend
1208	Descend
1209	Descend
1209	Descend

1209	Descend
1210	Descend
1211	Descend
1211	Descend
1212	Descend
1213	Descend
1214	Descend
1214	Descend
1215	Descend
1216	Descend
1216	Ascend
1216	Ascend
1216	Ascend
1217	Ascend
1217	Ascend
1217	Ascend
1217	Ascend
1218	Ascend
1218	Ascend
1218	Ascend
1218	Ascend
1219	Ascend
1219	Descend
1219	Descend
1219	Descend
1220	Descend
1220	Descend
1220	Descend
1221	Descend
1221	Descend
1221	Descend
1221	Ascend
1222	Ascend
1222	Ascend
1222	Ascend
1222	Ascend
1223	Descend
1223	Descend
1223	Descend
1223	Descend
1224	Descend
1224	Ascend

1224	Ascend
1226	Ascend
1226	Ascend
1226	Descend
1227	Ascend
1228	Ascend
1229	Descend
1230	Descend
1230	Descend
1230	Descend
1231	Descend
1231	Descend
1232	Descend
1232	Ascend
1233	Ascend
1233	Descend
1234	Descend
1234	Descend
1235	Ascend
1235	Ascend
1235	Ascend
1236	Ascend
1236	Ascend
1236	Ascend
1236	Descend
1236	Descend
1237	Descend
1237	Descend
1237	Descend
1237	Descend
1238	Descend
1238	Descend
1238	Descend
1238	Descend
1238	Descend
1239	Descend
1239	Descend
1239	Descend
1239	Descend
1240	Descend
1240	Descend
1240	Descend

1241	Ascend
1241	Ascend
1241	Ascend
1242	Ascend
1242	Ascend
1242	Descend
1242	Descend
1243	Ascend
1243	Ascend
1243	Descend
1243	Descend
1245	Descend
1245	Descend
1245	Ascend
1246	Descend
1246	Descend
1247	Descend
1247	Descend
1247	Descend
1247	Descend
1248	Ascend
1248	Ascend
1248	Ascend
1249	Ascend
1249	Ascend
1249	Ascend
1250	Ascend
1250	Ascend
1250	Ascend
1251	Descend
1251	Descend
1252	Descend
1252	Descend
1253	Descend
1253	Descend
1253	Descend
1254	Descend
1254	Descend
1254	Descend
1255	Descend
1255	Descend
1255	Descend

1256	Descend
1256	Descend
1256	Ascend
1256	Ascend
1257	Ascend
1257	Descend
1257	Descend
1258	Descend
1258	Ascend
1258	Ascend
1259	Ascend
1259	Ascend
1259	Ascend
1260	Ascend
1260	Ascend
1260	Ascend
1261	Ascend
1261	Ascend
1261	Ascend
1261	Ascend
1262	Ascend
1262	Ascend
1262	Ascend
1262	Ascend
1263	Descend
1263	Ascend
1263	Ascend
1264	Ascend
1264	Ascend
1264	Ascend
1264	Ascend
1265	Ascend
1265	Ascend
1265	Ascend
1265	Descend
1266	Descend
1266	Descend
1266	Descend
1268	Descend
1269	Descend
1269	Descend
1269	Descend

1270	Ascend
1270	Ascend
1270	Descend
1270	Descend
1271	Ascend
1271	Ascend
1271	Ascend
1272	Ascend
1272	Descend
1272	Descend
1273	Descend
1273	Descend
1273	Descend
1273	Descend
1274	Descend
1274	Descend
1274	Descend
1275	Descend
1275	Descend
1275	Descend
1276	Descend
1276	Descend
1276	Descend
1277	Descend
1277	Descend
1277	Descend
1277	Descend
1278	Ascend
1278	Ascend
1278	Ascend
1278	Ascend
1279	Ascend
1279	Ascend
1279	Ascend
1279	Ascend
1280	Ascend
1280	Ascend
1280	Ascend
1280	Ascend
1281	Ascend
1281	Descend
1282	Descend

1282	Descend
1282	Descend
1282	Descend
1283	Ascend
1283	Ascend
1284	Ascend
1284	Ascend
1285	Ascend
1285	Ascend
1285	Descend
1286	Descend
1286	Ascend
1286	Ascend
1287	Descend
1287	Descend
1288	Descend
1288	Descend
1289	Descend
1291	Descend
1295	Descend
1296	Ascend
1296	Ascend
1297	Ascend
1297	Ascend
1298	Ascend
1298	Ascend
1299	Ascend
1299	Ascend
1300	Ascend
1300	Ascend
1300	Ascend
1302	Descend
1302	Descend
1302	Descend
1302	Descend
1303	Descend
1303	Descend
1303	Descend
1303	Descend
1304	Descend
1304	Descend
1304	Descend

1304	Descend
1305	Descend
1305	Ascend
1305	Ascend
1306	Ascend
1306	Ascend
1306	Ascend
1306	Ascend
1307	Ascend
1307	Ascend
1308	Ascend
1308	Ascend
1309	Ascend
1309	Ascend
1310	Ascend
1310	Ascend
1311	Ascend
1311	Ascend
1312	Descend
1312	Descend
1312	Descend
1312	Descend
1313	Descend
1313	Descend
1313	Descend
1314	Descend
1314	Descend
1315	Descend
1315	Descend
1316	Descend
1316	Descend
1317	Descend
1317	Ascend
1317	Ascend
1318	Ascend
1319	Ascend
1319	Ascend
1320	Descend
1320	Descend
1320	Descend
1321	Ascend
1321	Ascend

1321	Descend
1321	Descend
1322	Descend
1322	Descend
1322	Descend
1322	Descend
1323	Descend
1323	Descend
1323	Descend
1324	Descend
1324	Descend
1324	Descend
1325	Descend
1325	Descend
1325	Descend
1326	Ascend
1326	Ascend
1326	Ascend
1327	Ascend
1327	Ascend
1327	Ascend
1328	Ascend
1328	Ascend
1328	Ascend
1329	Ascend
1329	Ascend
1329	Ascend
1329	Ascend
1330	Ascend
1330	Ascend
1330	Ascend
1330	Ascend
1331	Ascend
1331	Ascend
1331	Ascend
1331	Ascend
1332	Ascend
1332	Ascend
1332	Ascend
1332	Ascend
1333	Ascend
1333	Ascend

1333	Ascend
1334	Descend
1334	Descend
1334	Descend
1335	Descend
1335	Descend
1335	Descend
1335	Descend
1336	Descend
1336	Descend
1336	Descend
1337	Descend
1337	Descend
1338	Descend
1338	Descend
1338	Descend
1339	Descend
1339	Descend
1339	Descend
1339	Descend
1340	Descend
1340	Descend
1340	Descend
1341	Descend
1341	Descend
1341	Descend
1342	Descend
1342	Descend
1342	Descend
1343	Descend
1343	Descend
1343	Descend
1343	Descend
1344	Descend
1344	Descend
1344	Descend
1345	Descend
1345	Descend
1345	Descend
1346	Descend
1346	Descend
1346	Descend

1347	Descend
1347	Ascend
1347	Ascend
1347	Ascend
1348	Descend
1348	Descend
1349	Descend
1349	Descend
1349	Descend
1349	Descend
1349	Descend
1350	Ascend
1350	Ascend
1350	Ascend
1351	Ascend
1351	Descend
1351	Descend
1352	Descend
1352	Descend
1352	Descend
1352	Descend
1353	Descend
1353	Descend
1353	Descend
1354	Descend
1354	Descend
1354	Descend
1354	Descend
1355	Descend
1355	Descend
1356	Descend
1356	Descend
1356	Descend
1357	Descend
1357	Descend
1358	Ascend
1358	Ascend
1358	Ascend
1358	Ascend
1359	Ascend
1359	Ascend
1359	Descend

1359	Descend
1360	Descend
1360	Ascend
1360	Ascend
1361	Ascend
1361	Ascend
1361	Ascend
1362	Ascend
1362	Ascend
1362	Ascend
1363	Descend
1363	Descend
1363	Descend
1364	Descend
1364	Ascend
1365	Ascend
1365	Descend
1365	Descend
1366	Descend
1366	Descend
1366	Descend
1367	Descend
1367	Ascend
1367	Descend
1367	Descend
1368	Descend
1368	Descend
1369	Descend
1369	Descend
1369	Descend
1370	Descend
1370	Descend
1371	Descend
1372	Descend
1372	Descend
1372	Descend
1373	Descend
1373	Descend
1373	Descend
1374	Ascend
1374	Ascend
1374	Ascend

1374	Ascend
1375	Ascend
1375	Ascend
1376	Ascend
1376	Ascend
1377	Descend
1378	Ascend
1378	Ascend
1378	Ascend
1378	Ascend
1379	Descend
1379	Descend
1379	Descend
1380	Ascend
1380	Ascend
1380	Descend
1380	Descend
1381	Descend
1381	Descend
1381	Ascend
1382	Ascend
1382	Ascend
1382	Descend
1382	Descend
1383	Ascend
1383	Ascend
1383	Ascend
1383	Ascend
1384	Ascend
1384	Ascend
1384	Ascend
1385	Ascend
1385	Ascend
1385	Ascend
1386	Descend
1386	Descend
1387	Descend
1387	Ascend
1387	Ascend
1388	Ascend
1388	Ascend
1388	Ascend

1389	Ascend
1389	Ascend
1389	Ascend
1390	Ascend
1390	Ascend
1391	Descend
1391	Descend
1392	Descend
1392	Descend
1394	Descend
1394	Descend
1395	Descend
1395	Descend
1396	Descend
1396	Descend
1396	Descend
1397	Descend
1397	Descend
1397	Descend
1398	Descend
1398	Descend
1398	Descend
1399	Ascend
1399	Ascend
1399	Ascend
1400	Ascend
1400	Ascend
1400	Descend
1400	Descend
1401	Descend
1401	Descend
1401	Descend
1402	Descend
1402	Descend
1403	Ascend
1403	Ascend
1403	Ascend
1404	Descend
1404	Descend
1405	Descend
1405	Ascend
1405	Ascend

1406	Ascend
1406	Descend
1406	Descend
1407	Descend
1408	Ascend
1408	Ascend
1408	Ascend
1409	Ascend
1409	Ascend
1410	Ascend
1410	Ascend
1410	Ascend
1411	Ascend
1411	Ascend
1412	Ascend
1412	Ascend
1412	Ascend
1413	Ascend
1414	Ascend
1414	Ascend
1415	Ascend
1415	Ascend
1415	Ascend
1416	Ascend
1416	Ascend
1416	Ascend
1417	Ascend
1417	Ascend
1418	Ascend
1418	Ascend
1419	Ascend
1419	Ascend
1419	Ascend
1420	Ascend
1422	Descend
1422	Descend
1422	Descend
1423	Ascend
1423	Ascend
1424	Descend
1424	Descend
1424	Descend

1425	Descend
1425	Ascend
1426	Ascend
1426	Descend
1426	Descend
1427	Descend
1427	Descend
1428	Ascend
1429	Ascend
1429	Ascend
1430	Ascend
1430	Ascend
1431	Ascend
1433	Ascend
1433	Ascend
1434	Ascend
1434	Ascend
1434	Ascend
1435	Ascend
1435	Ascend
1436	Ascend
1436	Ascend
1436	Ascend
1437	Ascend
1437	Ascend
1437	Ascend
1438	Ascend
1438	Ascend
1438	Ascend
1439	Ascend
1439	Descend
1439	Descend
1439	Descend
1440	Descend
1440	Descend
1440	Descend
1442	Descend
1442	Descend
1442	Descend
1442	Descend
1443	Descend
1443	Descend

1443	Ascend
1444	Ascend
1444	Ascend
1444	Ascend
1444	Ascend
1444	Ascend
1445	Ascend
1445	Ascend
1445	Descend
1446	Descend
1446	Descend
1446	Descend
1446	Ascend
1447	Ascend
1447	Descend
1447	Descend
1447	Descend
1448	Ascend
1448	Ascend
1448	Descend
1448	Descend
1449	Descend
1449	Descend
1449	Descend
1449	Descend
1449	Descend
1450	Descend
1450	Descend
1450	Ascend
1450	Ascend
1451	Ascend
1451	Descend
1451	Descend
1451	Ascend
1452	Ascend
1452	Descend
1452	Descend
1453	Descend
1453	Descend
1453	Descend
1454	Descend
1454	Descend

1454	Descend
1455	Descend
1455	Descend
1457	Ascend
1457	Ascend
1458	Ascend
1458	Ascend
1459	Ascend
1459	Ascend
1459	Descend
1460	Descend
1460	Descend
1460	Ascend
1461	Ascend
1461	Descend
1461	Descend
1462	Descend
1462	Descend
1463	Descend
1463	Descend
1463	Descend
1464	Descend
1464	Descend
1464	Descend
1465	Descend
1465	Descend
1465	Descend
1466	Descend
1466	Descend
1467	Descend
1467	Descend
1467	Descend
1468	Descend
1469	Descend
1469	Descend
1469	Descend
1469	Descend
1470	Descend
1470	Descend
1471	Descend
1471	Descend
1471	Descend

1472	Descend
1473	Descend
1473	Descend
1473	Descend
1474	Descend
1474	Descend
1475	Descend
1476	Descend
1476	Descend
1476	Descend
1477	Descend
1477	Ascend
1478	Ascend
1478	Ascend
1478	Ascend
1478	Ascend
1479	Ascend
1479	Ascend
1480	Ascend
1480	Ascend
1480	Ascend
1480	Ascend
1481	Ascend
1481	Ascend
1481	Ascend
1481	Descend
1482	Descend
1482	Ascend
1482	Ascend
1482	Ascend
1483	Ascend
1483	Ascend
1483	Descend
1484	Descend
1484	Ascend
1485	Ascend
1485	Ascend
1486	Ascend
1486	Ascend
1487	Ascend
1487	Ascend
1488	Ascend

1488	Ascend
1488	Ascend
1489	Ascend
1489	Ascend
1490	Ascend
1490	Ascend
1491	Descend
1491	Descend
1491	Descend
1492	Descend
1492	Descend
1493	Ascend
1493	Ascend
1493	Ascend
1494	Ascend
1495	Descend
1495	Descend
1496	Ascend
1496	Ascend
1497	Descend
1497	Descend
1497	Descend
1497	Descend
1498	Ascend
1498	Ascend
1499	Descend
1499	Descend
1500	Ascend
1500	Ascend
1500	Descend
1500	Descend
1501	Ascend
1501	Ascend
1501	Descend
1501	Descend
1502	Ascend
1502	Ascend
1502	Ascend
1503	Ascend
1503	Ascend
1503	Ascend
1503	Ascend

1504	Ascend
1504	Ascend
1504	Ascend
1504	Ascend
1505	Ascend
1505	Ascend
1505	Ascend
1505	Ascend
1506	Ascend
1506	Ascend
1506	Ascend
1507	Ascend
1507	Ascend
1507	Ascend
1507	Ascend
1507	Ascend
1508	Ascend
1508	Ascend
1508	Ascend
1508	Ascend
1509	Ascend
1509	Ascend
1509	Ascend
1509	Ascend
1510	Ascend
1510	Ascend
1510	Ascend
1511	Ascend
1511	Ascend
1511	Ascend
1512	Ascend
1512	Ascend
1512	Ascend
1512	Ascend
1513	Ascend
1513	Descend
1514	Descend
1514	Descend
1514	Descend
1514	Descend
1515	Descend
1515	Ascend

1515	Ascend
1516	Descend
1516	Descend
1516	Descend
1516	Descend
1517	Ascend
1517	Ascend
1521	Descend
1521	Descend
1522	Ascend
1522	Ascend
1522	Ascend
1522	Ascend
1522	Ascend
1523	Ascend
1523	Ascend
1524	Ascend
1525	Ascend
1525	Ascend
1525	Ascend
1526	Descend
1526	Descend
1526	Descend
1526	Descend
1527	Descend
1527	Descend
1527	Descend
1528	Descend
1528	Descend
1528	Descend
1529	Descend
1529	Descend
1530	Descend
1530	Descend
1530	Descend
1531	Descend
1531	Descend
1531	Descend
1532	Descend
1532	Descend
1532	Descend
1532	Descend

1533	Descend
1533	Descend
1533	Descend
1534	Ascend
1534	Ascend
1534	Ascend
1535	Descend
1535	Descend
1535	Ascend
1535	Ascend
1536	Ascend
1536	Ascend
1536	Ascend
1537	Ascend
1537	Descend
1538	Descend
1538	Ascend
1538	Ascend
1539	Descend
1539	Descend
1540	Ascend
1540	Ascend
1540	Ascend
1541	Ascend
1541	Ascend
1542	Ascend
1542	Ascend
1543	Ascend
1543	Ascend
1543	Ascend
1543	Ascend
1544	Ascend
1544	Ascend
1544	Ascend
1544	Ascend
1545	Ascend
1545	Ascend
1545	Ascend
1545	Ascend
1546	Ascend
1546	Descend
1547	Descend

1550 Ascend
1551 Descend
1551 Ascend
1552 Ascend
1553 Ascend
1553 Ascend
1553 Ascend
1553 Ascend
1554 Ascend
1554 Ascend
1555 Ascend
1555 Ascend
1555 Ascend
1555 Ascend
1556 Ascend
1556 Ascend
1556 Descend
1556 Descend
1556 Descend
1557 Descend
1557 Descend
1557 Descend
1558 Ascend
1558 Ascend
1558 Descend
1559 Descend
1559 Ascend
1559 Ascend
1560 Ascend
1560 Ascend
1560 Ascend
1561 Descend
1561 Descend
1561 Ascend
1561 Ascend
1562 Ascend
1562 Ascend
1562 Ascend
1563 Ascend
1563 Ascend
1563 Ascend
1564 Ascend

1564	Ascend
1564	Ascend
1565	Ascend
1566	Descend
1567	Ascend
1567	Descend
1567	Ascend
1567	Ascend
1568	Ascend
1568	Ascend
1568	Ascend
1569	Ascend
1569	Ascend
1569	Ascend
1570	Ascend
1570	Ascend
1570	Ascend
1571	Ascend
1571	Ascend
1571	Ascend
1572	Ascend
1572	Ascend
1573	Ascend
1573	Ascend
1574	Ascend
1574	Ascend
1575	Ascend
1575	Ascend
1576	Descend
1576	Descend
1576	Descend
1577	Descend
1577	Descend
1578	Descend
1578	Ascend
1578	Ascend
1579	Ascend
1579	Ascend
1579	Ascend
1579	Ascend
1580	Ascend
1580	Ascend

1581	Ascend
1581	Ascend
1582	Ascend
1582	Ascend
1583	Descend
1583	Descend
1583	Descend
1584	Descend
1584	Descend
1584	Descend
1585	Descend
1585	Descend
1586	Descend
1586	Descend
1586	Ascend
1586	Ascend
1587	Ascend
1587	Ascend
1588	Ascend
1588	Ascend
1588	Ascend
1588	Ascend
1589	Ascend
1589	Ascend
1589	Ascend
1589	Ascend
1589	Ascend
1590	Ascend
1590	Ascend
1590	Descend
1590	Descend
1591	Descend
1591	Descend
1591	Descend
1592	Descend
1592	Descend
1592	Ascend
1592	Ascend
1593	Ascend
1593	Ascend
1593	Ascend
1593	Ascend

1594	Ascend
1594	Ascend
1594	Ascend
1595	Ascend
1595	Ascend
1595	Ascend
1596	Ascend
1596	Ascend
1596	Ascend
1597	Descend
1597	Descend
1598	Descend
1598	Descend
1598	Descend
1599	Descend
1599	Descend
1599	Descend
1600	Descend
1600	Descend
1600	Descend
1601	Descend
1601	Ascend
1601	Ascend
1602	Ascend
1602	Ascend
1602	Ascend
1602	Ascend
1603	Ascend
1603	Ascend
1604	Ascend
1604	Ascend
1604	Ascend
1604	Ascend
1604	Ascend
1605	Ascend
1605	Ascend
1605	Ascend
1605	Ascend
1605	Ascend
1606	Ascend
1606	Ascend
1606	Ascend

1606	Ascend
1607	Ascend
1607	Ascend
1607	Ascend
1608	Ascend
1608	Ascend
1608	Ascend
1608	Ascend
1609	Ascend
1609	Ascend
1609	Ascend
1609	Ascend
1610	Ascend
1610	Ascend
1610	Ascend
1610	Ascend
1610	Ascend
1611	Ascend
1611	Ascend
1611	Ascend
1612	Ascend
1612	Ascend
1612	Ascend
1613	Ascend
1613	Ascend
1613	Ascend
1613	Ascend
1614	Ascend
1614	Ascend
1614	Ascend
1614	Ascend
1615	Ascend
1615	Ascend
1615	Ascend
1615	Ascend
1616	Ascend
1616	Ascend
1616	Ascend
1616	Ascend
1617	Ascend
1617	Ascend
1617	Ascend

1617	Ascend
1618	Ascend
1618	Ascend
1618	Ascend
1618	Ascend
1619	Ascend
1619	Ascend
1619	Ascend
1619	Ascend
1620	Ascend
1620	Ascend
1620	Ascend
1621	Ascend
1621	Ascend
1621	Ascend
1621	Ascend
1622	Descend
1622	Descend
1622	Descend
1622	Descend
1622	Descend
1623	Descend
1623	Descend
1623	Ascend
1623	Ascend
1623	Ascend
1624	Ascend
1624	Ascend
1624	Ascend
1624	Ascend
1625	Ascend
1625	Ascend
1626	Ascend
1626	Ascend
1626	Ascend
1627	Ascend
1627	Ascend
1627	Ascend
1627	Ascend
1627	Ascend
1628	Ascend
1628	Ascend

1628	Ascend
1629	Ascend
1629	Ascend
1629	Ascend
1629	Descend
1630	Ascend
1630	Ascend
1630	Ascend
1630	Ascend
1631	Ascend
1631	Ascend
1631	Ascend
1631	Ascend
1632	Descend
1632	Descend
1632	Descend
1633	Descend
1633	Descend
1633	Descend
1633	Descend
1634	Descend
1634	Descend
1634	Ascend
1635	Ascend
1635	Ascend
1635	Ascend
1635	Ascend
1636	Ascend
1636	Descend
1636	Descend
1637	Ascend
1637	Descend
1637	Descend
1637	Descend
1638	Descend
1638	Descend
1638	Descend
1638	Descend
1638	Ascend
1639	Ascend
1639	Ascend
1639	Ascend

1639	Ascend
1640	Ascend
1640	Descend
1640	Descend
1640	Descend
1640	Ascend
1641	Ascend
1641	Ascend
1641	Ascend
1641	Ascend
1641	Ascend
1642	Ascend
1642	Ascend
1642	Ascend
1643	Ascend
1643	Descend
1643	Descend
1643	Ascend
1644	Ascend
1644	Ascend
1644	Ascend
1644	Ascend
1645	Ascend
1645	Descend
1645	Ascend
1646	Ascend
1646	Ascend
1646	Ascend
1646	Ascend
1647	Ascend
1647	Ascend
1647	Ascend
1647	Ascend
1648	Ascend
1648	Ascend
1648	Ascend
1648	Ascend
1649	Ascend
1649	Ascend
1649	Descend
1650	Descend
1650	Ascend

1650	Ascend
1650	Ascend
1651	Descend
1651	Descend
1651	Ascend
1651	Ascend
1652	Descend
1652	Descend
1652	Ascend
1652	Ascend
1653	Descend
1653	Descend
1653	Descend
1654	Descend
1654	Ascend
1654	Ascend
1654	Ascend
1655	Ascend
1655	Ascend
1655	Ascend
1655	Ascend
1656	Ascend
1656	Ascend
1657	Ascend
1657	Ascend
1657	Descend
1657	Descend
1658	Ascend
1658	Ascend
1658	Ascend
1658	Ascend
1659	Ascend
1659	Ascend
1659	Ascend
1660	Ascend
1660	Ascend
1660	Ascend
1661	Descend
1661	Descend
1661	Descend
1661	Descend
1662	Ascend

1662	Ascend
1662	Descend
1662	Descend
1663	Descend
1663	Descend
1663	Descend
1663	Descend
1664	Descend
1664	Descend
1664	Descend
1665	Descend
1665	Descend
1665	Descend
1665	Descend
1666	Descend
1666	Descend
1666	Descend
1667	Descend
1667	Ascend
1667	Ascend
1667	Ascend
1668	Descend
1668	Descend
1668	Ascend
1668	Ascend
1669	Descend
1669	Ascend
1669	Ascend
1669	Ascend
1670	Ascend
1670	Descend
1670	Descend
1671	Ascend
1671	Ascend
1671	Ascend
1672	Ascend
1672	Ascend
1673	Ascend
1673	Ascend
1673	Descend
1673	Descend
1674	Ascend

1674	Descend
1674	Descend
1675	Descend
1675	Descend
1675	Descend
1675	Descend
1675	Descend
1676	Descend
1676	Ascend
1676	Ascend
1676	Ascend
1677	Ascend
1677	Descend
1677	Ascend
1677	Ascend
1677	Ascend
1678	Descend
1678	Ascend
1678	Ascend
1679	Ascend
1679	Descend
1679	Ascend
1680	Ascend
1680	Ascend
1680	Descend
1681	Descend
1681	Ascend
1681	Ascend
1681	Ascend
1682	Descend
1682	Descend
1682	Ascend
1682	Ascend
1683	Descend
1683	Descend
1683	Descend
1683	Ascend
1683	Ascend
1684	Ascend
1684	Ascend
1684	Descend
1684	Descend

1684	Ascend
1685	Ascend
1685	Descend
1685	Descend
1685	Descend
1686	Ascend
1686	Ascend
1687	Descend
1687	Descend
1687	Ascend
1688	Ascend
1688	Ascend
1688	Ascend
1688	Ascend
1689	Ascend
1689	Descend
1689	Descend
1689	Ascend
1690	Ascend
1690	Descend
1690	Descend
1690	Descend
1691	Descend
1692	Ascend
1693	Ascend
1693	Ascend
1694	Ascend
1695	Ascend
1695	Ascend
1695	Descend
1696	Descend
1698	Descend
1698	Descend
1699	Ascend
1699	Ascend
1699	Ascend
1699	Ascend
1699	Ascend
1700	Ascend
1700	Ascend
1700	Ascend
1701	Ascend

1701	Ascend
1701	Ascend
1701	Ascend
1702	Ascend
1702	Descend
1702	Descend
1702	Descend
1703	Descend
1703	Descend
1703	Descend
1704	Descend
1704	Descend
1704	Descend
1704	Descend
1705	Ascend
1705	Ascend
1706	Descend
1706	Descend
1706	Descend
1706	Ascend
1707	Descend
1707	Descend
1707	Ascend
1708	Ascend
1708	Ascend
1708	Descend
1709	Descend
1709	Descend
1709	Descend
1709	Descend
1710	Descend
1710	Descend
1710	Descend
1710	Descend
1711	Ascend
1711	Ascend
1711	Ascend
1711	Descend
1712	Descend
1712	Descend
1712	Descend
1712	Descend

1713	Descend
1713	Descend
1714	Ascend
1714	Ascend
1714	Ascend
1715	Ascend
1715	Ascend
1715	Ascend
1716	Descend
1716	Descend
1717	Ascend
1717	Ascend
1717	Ascend
1717	Ascend
1718	Ascend
1718	Ascend
1718	Ascend
1719	Ascend
1719	Ascend
1719	Ascend
1719	Ascend
1719	Ascend
1720	Ascend
1720	Ascend
1720	Descend
1720	Descend
1720	Descend
1721	Descend
1721	Descend
1721	Descend
1721	Descend
1721	Ascend
1722	Ascend
1722	Ascend
1722	Descend
1722	Descend
1722	Descend
1723	Descend
1723	Descend
1723	Descend
1724	Descend
1724	Descend

1724	Descend
1725	Descend
1725	Ascend
1725	Ascend
1726	Descend
1726	Descend
1726	Ascend
1726	Ascend
1727	Descend
1727	Descend
1728	Descend
1728	Descend
1728	Descend
1729	Descend
1729	Ascend
1730	Ascend
1730	Ascend
1730	Ascend
1730	Ascend
1731	Ascend
1731	Ascend
1731	Ascend
1732	Descend
1732	Descend
1733	Descend
1733	Descend
1733	Descend
1733	Descend
1734	Descend
1734	Descend
1735	Descend
1735	Descend
1736	Descend
1736	Ascend
1737	Ascend
1737	Ascend
1738	Ascend
1738	Ascend
1739	Ascend
1739	Ascend
1739	Ascend
1740	Descend

1740	Descend
1741	Descend
1741	Descend
1741	Descend
1742	Descend
1743	Descend
1743	Descend
1743	Descend
1743	Descend
1744	Descend
1744	Descend
1744	Descend
1745	Descend
1745	Descend
1745	Descend
1745	Descend
1746	Descend
1746	Descend
1746	Descend
1747	Descend
1747	Descend
1747	Descend
1748	Ascend
1748	Ascend
1748	Ascend
1748	Ascend
1749	Ascend
1749	Ascend
1749	Ascend
1749	Ascend
1750	Descend
1750	Descend
1750	Descend
1750	Descend
1751	Descend
1751	Descend
1751	Descend
1752	Descend
1752	Ascend
1752	Ascend
1752	Ascend
1753	Ascend

1753	Ascend
1753	Ascend
1753	Ascend
1754	Ascend
1754	Descend
1754	Descend
1755	Descend
1755	Ascend
1755	Ascend
1756	Ascend
1756	Descend
1756	Descend
1756	Descend
1757	Descend
1757	Descend
1757	Descend
1758	Descend
1758	Ascend
1758	Ascend
1759	Descend
1759	Ascend
1759	Ascend
1760	Ascend
1760	Descend
1760	Descend
1761	Descend
1761	Descend
1761	Descend
1762	Descend
1762	Descend
1763	Descend
1763	Descend
1763	Descend
1764	Descend
1764	Descend
1764	Descend
1765	Descend
1765	Descend
1765	Descend
1765	Descend
1766	Descend
1766	Descend

1766	Descend
1767	Ascend
1768	Ascend
1768	Ascend
1769	Descend
1769	Descend
12	AntIn
17	AntIn
19	AntIn
30	AntIn
37	AntIn
37	AntIn
41	AntIn
42	AntOut
48	AntIn
53	AntIn
53	AntIn
65	AntIn
71	AntIn
76	AntIn
81	AntOut
84	AntIn
91	AntOut
91	AntOut
93	AntOut
101	AntOut
104	AntIn
107	AntIn
111	AntOut
115	AntIn
132	AntIn
132	AntOut
136	AntIn
137	AntOut
146	AntIn
149	AntIn
173	AntIn
184	AntIn
203	AntIn
208	AntIn
219	AntIn
219	AntOut

222	AntIn
227	AntOut
232	AntIn
246	AntOut
246	AntOut
248	AntIn
253	AntOut
257	AntIn
261	AntIn
263	AntOut
283	AntIn
286	AntIn
288	AntOut
294	AntOut
297	AntOut
298	AntOut
306	AntOut
317	AntIn
320	AntIn
326	AntIn
329	AntOut
331	AntIn
332	AntOut
332	AntOut
338	AntIn
352	AntOut
355	AntIn
356	AntOut
358	AntIn
360	AntOut
362	AntOut
366	AntOut
368	AntOut
375	AntIn
375	AntOut
376	AntOut
378	AntOut
380	AntOut
381	AntIn
383	AntOut
384	AntIn
385	AntIn

385	AntOut
386	AntOut
389	AntOut
389	AntOut
391	AntIn
393	AntOut
396	AntIn
397	AntOut
402	AntIn
403	AntOut
405	AntOut
406	AntOut
406	AntOut
409	AntIn
409	AntOut
412	AntOut
414	AntIn
417	AntOut
419	AntIn
420	AntOut
422	AntOut
422	AntIn
423	AntOut
424	AntIn
433	AntIn
434	AntIn
435	AntOut
437	AntOut
437	AntOut
438	AntIn
438	AntOut
439	AntOut
439	AntOut
441	AntOut
442	AntOut
443	AntIn
444	AntIn
447	AntOut
447	AntOut
450	AntIn
451	AntOut
451	AntOut

453	AntIn
457	AntIn
458	AntIn
459	AntIn
460	AntIn
461	AntIn
462	AntOut
463	AntIn
464	AntOut
471	AntOut
472	AntIn
473	AntOut
475	AntOut
475	AntOut
478	AntOut
480	AntIn
482	AntIn
482	AntOut
484	AntIn
491	AntOut
493	AntIn
495	AntIn
498	AntIn
499	AntIn
504	AntIn
505	AntIn
507	AntOut
509	AntOut
510	AntOut
512	AntIn
516	AntOut
517	AntOut
518	AntOut
530	AntIn
531	AntOut
537	AntIn
538	AntOut
538	AntOut
540	AntIn
545	AntIn
549	AntOut
549	AntOut

551 AntOut
551 AntIn
553 AntIn
554 AntIn
554 AntOut
556 AntIn
557 AntIn
559 AntOut
560 AntOut
560 AntOut
561 AntOut
562 AntOut
562 AntOut
563 AntOut
563 AntOut
564 AntIn
566 AntOut
567 AntOut
568 AntOut
569 AntIn
571 AntOut
571 AntOut
572 AntIn
573 AntOut
573 AntOut
575 AntIn
576 AntOut
576 AntIn
577 AntOut
580 AntOut
583 AntOut
583 AntOut
584 AntOut
584 AntOut
586 AntOut
587 AntOut
588 AntOut
589 AntIn
589 AntIn
590 AntIn
591 AntIn
591 AntIn

593	AntIn
593	AntIn
593	AntIn
594	AntOut
595	AntOut
595	AntOut
598	AntIn
599	AntIn
601	AntOut
602	AntOut
603	AntIn
604	AntOut
605	AntOut
606	AntIn
606	AntIn
607	AntOut
607	AntOut
607	AntIn
609	AntOut
610	AntOut
610	AntOut
611	AntOut
613	AntOut
613	AntOut
617	AntIn
620	AntIn
621	AntOut
623	AntIn
624	AntOut
625	AntOut
627	AntIn
627	AntIn
630	AntIn
631	AntIn
633	AntOut
635	AntOut
636	AntOut
638	AntIn
638	AntIn
642	AntOut
643	AntOut
647	AntIn

648 AntOut
652 AntIn
653 AntIn
655 AntIn
656 AntOut
659 AntIn
661 AntIn
664 AntIn
670 AntOut
670 AntIn
671 AntOut
674 AntOut
675 AntIn
677 AntOut
680 AntOut
681 AntIn
683 AntOut
685 AntIn
691 AntIn
693 AntOut
693 AntOut
694 AntOut
696 AntOut
696 AntOut
696 AntOut
696 AntOut
698 AntIn
698 AntIn
698 AntIn
703 AntOut
707 AntIn
707 AntIn
707 AntOut
709 AntOut
715 AntIn
715 AntOut
717 AntIn
717 AntOut
718 AntOut
718 AntOut
718 AntIn
719 AntIn

719 AntIn
720 AntOut
721 AntIn
723 AntOut
724 AntIn
731 AntOut
735 AntOut
736 AntIn
738 AntIn
738 AntOut
739 AntIn
744 AntIn
746 AntOut
749 AntOut
750 AntIn
750 AntOut
754 AntIn
756 AntIn
758 AntOut
761 AntOut
761 AntOut
762 AntOut
763 AntOut
763 AntOut
764 AntIn
765 AntOut
765 AntOut
766 AntOut
767 AntOut
768 AntOut
769 AntIn
769 AntIn
771 AntOut
772 AntOut
773 AntOut
773 AntOut
773 AntOut
775 AntOut
777 AntOut
777 AntOut
778 AntOut
778 AntOut

781 AntIn
782 AntIn
782 AntOut
783 AntOut
783 AntOut
784 AntOut
784 AntOut
785 AntOut
786 AntOut
786 AntOut
787 AntOut
788 AntIn
789 AntIn
789 AntOut
790 AntOut
791 AntIn
792 AntOut
794 AntIn
795 AntIn
796 AntOut
797 AntOut
800 AntIn
801 AntIn
802 AntOut
802 AntOut
803 AntOut
803 AntOut
803 AntIn
803 AntIn
804 AntIn
805 AntOut
806 AntOut
808 AntOut
811 AntIn
811 AntIn
811 AntIn
812 AntOut
813 AntOut
814 AntOut
815 AntIn
817 AntIn
819 AntOut

820 AntOut
821 AntOut
823 AntOut
823 AntIn
824 AntIn
825 AntOut
826 AntOut
829 AntOut
830 AntOut
831 AntOut
832 AntOut
832 AntIn
833 AntOut
834 AntOut
835 AntIn
835 AntOut
838 AntIn
839 AntIn
843 AntIn
844 AntIn
845 AntIn
846 AntOut
846 AntOut
846 AntOut
848 AntOut
850 AntOut
850 AntOut
851 AntIn
854 AntOut
855 AntOut
856 AntIn
858 AntOut
858 AntOut
859 AntOut
860 AntOut
860 AntOut
861 AntIn
863 AntIn
865 AntIn
865 AntIn
866 AntOut
867 AntIn

871	AntOut
873	AntIn
875	AntIn
876	AntOut
879	AntIn
879	AntIn
880	AntIn
880	AntIn
881	AntIn
882	AntOut
882	AntIn
884	AntOut
884	AntIn
885	AntOut
885	AntOut
885	AntOut
886	AntOut
888	AntOut
889	AntIn
889	AntIn
889	AntOut
890	AntOut
891	AntOut
891	AntIn
891	AntOut
892	AntOut
892	AntOut
893	AntOut
893	AntOut
899	AntOut
900	AntIn
902	AntIn
902	AntIn
904	AntOut
905	AntOut
906	AntIn
907	AntOut
907	AntOut
908	AntOut
908	AntIn
910	AntIn
911	AntIn

911 AntIn
912 AntOut
912 AntIn
914 AntOut
914 AntOut
915 AntOut
915 AntOut
916 AntIn
917 AntOut
919 AntOut
920 AntOut
920 AntIn
921 AntIn
922 AntOut
923 AntOut
923 AntIn
923 AntIn
925 AntIn
926 AntOut
929 AntIn
930 AntIn
930 AntIn
930 AntIn
931 AntIn
936 AntOut
937 AntOut
937 AntOut
938 AntIn
939 AntOut
942 AntOut
943 AntIn
944 AntOut
944 AntOut
945 AntOut
946 AntOut
947 AntIn
948 AntIn
949 AntIn
951 AntIn
954 AntOut
955 AntIn
956 AntOut

957	AntIn
958	AntOut
959	AntIn
965	AntIn
967	AntOut
968	AntOut
969	AntIn
969	AntOut
971	AntIn
971	AntOut
973	AntOut
974	AntIn
976	AntOut
979	AntIn
980	AntIn
981	AntIn
981	AntIn
983	AntOut
983	AntOut
984	AntOut
985	AntIn
987	AntOut
990	AntOut
991	AntOut
991	AntOut
992	AntOut
993	AntOut
993	AntOut
994	AntOut
995	AntIn
997	AntOut
997	AntOut
998	AntOut
998	AntIn
999	AntOut
1001	AntOut
1001	AntOut
1002	AntOut
1003	AntOut
1003	AntOut
1004	AntIn
1005	AntIn

1005 AntIn
1005 AntOut
1007 AntOut
1007 AntOut
1008 AntOut
1009 AntIn
1010 AntOut
1011 AntOut
1012 AntOut
1012 AntIn
1018 AntOut
1018 AntIn
1019 AntIn
1019 AntOut
1022 AntOut
1023 AntOut
1024 AntOut
1026 AntOut
1026 AntIn
1027 AntIn
1027 AntOut
1029 AntIn
1031 AntOut
1031 AntOut
1033 AntOut
1034 AntIn
1035 AntOut
1036 AntIn
1038 AntIn
1039 AntIn
1040 AntIn
1040 AntOut
1042 AntOut
1042 AntIn
1043 AntOut
1044 AntIn
1045 AntOut
1046 AntOut
1050 AntIn
1051 AntOut
1051 AntIn
1052 AntIn

1053	AntIn
1053	AntIn
1053	AntIn
1054	AntOut
1055	AntOut
1056	AntIn
1059	AntOut
1061	AntIn
1062	AntOut
1063	AntOut
1065	AntIn
1066	AntIn
1067	AntIn
1067	AntOut
1069	AntOut
1070	AntOut
1070	AntOut
1071	AntOut
1071	AntOut
1072	AntOut
1073	AntOut
1073	AntOut
1074	AntIn
1075	AntOut
1075	AntIn
1076	AntIn
1077	AntIn
1077	AntIn
1078	AntOut
1079	AntOut
1079	AntIn
1081	AntOut
1082	AntOut
1082	AntIn
1083	AntOut
1084	AntIn
1085	AntIn
1086	AntIn
1087	AntOut
1087	AntIn
1087	AntIn
1087	AntIn

1088 AntOut
1089 AntIn
1091 AntIn
1091 AntIn
1092 AntIn
1093 AntIn
1093 AntIn
1094 AntIn
1094 AntOut
1096 AntOut
1097 AntIn
1097 AntIn
1099 AntOut
1100 AntOut
1102 AntIn
1102 AntOut
1103 AntIn
1104 AntOut
1106 AntOut
1106 AntOut
1106 AntOut
1107 AntOut
1107 AntIn
1109 AntOut
1110 AntOut
1110 AntIn
1112 AntOut
1113 AntOut
1115 AntOut
1116 AntIn
1117 AntIn
1120 AntOut
1122 AntOut
1123 AntIn
1124 AntIn
1124 AntIn
1126 AntOut
1126 AntIn
1127 AntIn
1127 AntIn
1127 AntIn
1128 AntOut

1129 AntIn
1129 AntIn
1130 AntIn
1130 AntIn
1131 AntOut
1131 AntOut
1132 AntOut
1132 AntOut
1133 AntOut
1134 AntOut
1135 AntIn
1137 AntOut
1138 AntOut
1138 AntOut
1138 AntOut
1139 AntOut
1139 AntOut
1139 AntIn
1140 AntOut
1141 AntOut
1141 AntOut
1141 AntOut
1142 AntOut
1143 AntOut
1144 AntOut
1145 AntOut
1145 AntOut
1145 AntOut
1146 AntIn
1146 AntIn
1148 AntOut
1148 AntOut
1149 AntOut
1152 AntIn
1152 AntOut
1154 AntOut
1154 AntIn
1155 AntOut
1156 AntOut
1158 AntOut
1159 AntOut
1159 AntOut

1160 AntOut
1160 AntIn
1161 AntOut
1162 AntOut
1163 AntIn
1163 AntIn
1163 AntIn
1164 AntOut
1164 AntOut
1165 AntOut
1165 AntOut
1165 AntOut
1167 AntOut
1168 AntOut
1169 AntIn
1169 AntIn
1170 AntOut
1171 AntIn
1172 AntOut
1172 AntOut
1173 AntOut
1173 AntOut
1174 AntOut
1175 AntOut
1176 AntOut
1177 AntIn
1178 AntIn
1178 AntOut
1179 AntOut
1180 AntOut
1181 AntOut
1182 AntIn
1182 AntIn
1183 AntIn
1183 AntIn
1185 AntOut
1185 AntIn
1185 AntIn
1186 AntIn
1188 AntOut
1188 AntIn
1189 AntOut

1189	AntOut
1190	AntOut
1192	AntIn
1193	AntIn
1194	AntOut
1195	AntOut
1196	AntOut
1196	AntIn
1197	AntOut
1199	AntIn
1200	AntOut
1201	AntIn
1202	AntIn
1202	AntOut
1203	AntOut
1204	AntOut
1205	AntIn
1206	AntIn
1206	AntIn
1207	AntOut
1207	AntOut
1208	AntOut
1208	AntOut
1209	AntOut
1209	AntOut
1210	AntOut
1210	AntOut
1210	AntOut
1211	AntOut
1211	AntOut
1212	AntOut
1213	AntOut
1213	AntIn
1214	AntIn
1214	AntOut
1214	AntOut
1215	AntOut
1215	AntOut
1216	AntOut
1216	AntIn
1218	AntIn
1219	AntIn

1219 AntIn
1220 AntIn
1220 AntIn
1220 AntIn
1221 AntOut
1221 AntOut
1222 AntOut
1223 AntOut
1223 AntOut
1224 AntOut
1224 AntOut
1224 AntOut
1225 AntOut
1226 AntOut
1226 AntIn
1227 AntIn
1228 AntOut
1229 AntOut
1229 AntIn
1230 AntOut
1231 AntOut
1232 AntIn
1233 AntIn
1233 AntIn
1234 AntOut
1234 AntOut
1235 AntOut
1235 AntOut
1236 AntIn
1237 AntOut
1237 AntOut
1238 AntOut
1239 AntOut
1239 AntOut
1240 AntIn
1240 AntOut
1243 AntOut
1243 AntOut
1244 AntOut
1244 AntOut
1245 AntOut
1246 AntIn

1247 AntOut
1247 AntOut
1248 AntOut
1248 AntOut
1248 AntOut
1249 AntOut
1251 AntIn
1251 AntIn
1252 AntIn
1254 AntIn
1255 AntIn
1255 AntIn
1256 AntIn
1256 AntIn
1257 AntOut
1258 AntOut
1258 AntOut
1259 AntOut
1259 AntOut
1259 AntOut
1259 AntOut
1259 AntOut
1260 AntOut
1260 AntOut
1262 AntOut
1262 AntOut
1262 AntIn
1264 AntIn
1265 AntOut
1265 AntIn
1266 AntIn
1266 AntIn
1267 AntOut
1268 AntOut
1269 AntIn
1269 AntOut
1272 AntIn
1273 AntOut
1273 AntOut
1274 AntOut
1276 AntIn
1276 AntIn

1277	AntOut
1277	AntOut
1277	AntOut
1278	AntOut
1278	AntOut
1278	AntOut
1279	AntIn
1280	AntOut
1280	AntIn
1280	AntOut
1281	AntOut
1281	AntIn
1282	AntIn
1284	AntOut
1285	AntOut
1285	AntOut
1286	AntOut
1286	AntOut
1286	AntOut
1286	AntOut
1286	AntOut
1287	AntOut
1287	AntIn
1288	AntIn
1288	AntIn
1288	AntIn
1289	AntIn
1289	AntIn
1289	AntIn
1290	AntIn
1290	AntOut
1290	AntOut
1291	AntOut
1291	AntIn
1291	AntOut
1291	AntOut
1292	AntIn
1292	AntIn
1293	AntOut
1294	AntOut
1295	AntOut
1297	AntIn

1298	AntIn
1299	AntIn
1300	AntOut
1300	AntOut
1301	AntOut
1302	AntOut
1302	AntOut
1303	AntIn
1303	AntIn
1303	AntIn
1305	AntIn
1306	AntIn
1306	AntIn
1306	AntIn
1307	AntIn
1307	AntIn
1308	AntOut
1308	AntIn
1310	AntIn
1311	AntOut
1311	AntOut
1312	AntOut
1312	AntOut
1313	AntIn
1313	AntIn
1314	AntOut
1315	AntOut
1315	AntOut
1315	AntOut
1315	AntOut
1315	AntOut
1316	AntOut
1316	AntIn
1317	AntOut
1317	AntOut
1318	AntOut
1319	AntOut
1319	AntIn
1319	AntIn
1320	AntOut
1320	AntOut
1320	AntIn

1321 AntIn
1322 AntIn
1323 AntOut
1323 AntOut
1323 AntIn
1323 AntIn
1324 AntIn
1325 AntOut
1326 AntOut
1326 AntIn
1327 AntIn
1328 AntIn
1328 AntOut
1329 AntOut
1329 AntOut
1329 AntIn
1330 AntIn
1331 AntIn
1332 AntOut
1332 AntOut
1333 AntOut
1333 AntOut
1334 AntIn
1334 AntIn
1336 AntOut
1337 AntIn
1337 AntOut
1338 AntIn
1339 AntIn
1339 AntIn
1339 AntIn
1340 AntIn
1340 AntIn
1341 AntIn
1341 AntOut
1341 AntOut
1342 AntOut
1342 AntOut
1342 AntIn
1344 AntIn
1344 AntOut
1344 AntOut

1345	AntIn
1345	AntIn
1346	AntIn
1347	AntOut
1348	AntOut
1350	AntIn
1351	AntOut
1351	AntIn
1352	AntIn
1353	AntIn
1354	AntOut
1354	AntOut
1355	AntIn
1355	AntOut
1356	AntOut
1356	AntIn
1358	AntOut
1358	AntIn
1359	AntIn
1363	AntOut
1364	AntIn
1364	AntIn
1365	AntOut
1366	AntIn
1366	AntOut
1366	AntIn
1367	AntIn
1369	AntIn
1370	AntIn
1370	AntOut
1370	AntOut
1371	AntOut
1371	AntOut
1373	AntIn
1374	AntIn
1375	AntOut
1376	AntOut
1377	AntOut
1377	AntOut
1378	AntOut
1378	AntIn
1379	AntIn

1379 AntOut
1380 AntIn
1381 AntIn
1383 AntIn
1384 AntIn
1385 AntIn
1386 AntOut
1386 AntOut
1387 AntIn
1387 AntIn
1388 AntOut
1391 AntIn
1392 AntIn
1393 AntIn
1393 AntIn
1395 AntOut
1395 AntIn
1396 AntIn
1397 AntIn
1397 AntIn
1398 AntIn
1399 AntOut
1399 AntIn
1400 AntIn
1401 AntIn
1402 AntOut
1404 AntOut
1404 AntOut
1405 AntOut
1406 AntOut
1406 AntIn
1408 AntIn
1409 AntIn
1410 AntOut
1411 AntIn
1412 AntIn
1413 AntOut
1414 AntOut
1415 AntOut
1416 AntOut
1417 AntOut
1417 AntOut

1418	AntOut
1418	AntOut
1419	AntIn
1419	AntIn
1420	AntIn
1420	AntIn
1421	AntIn
1421	AntIn
1423	AntIn
1424	AntIn
1425	AntIn
1426	AntOut
1426	AntOut
1427	AntOut
1428	AntIn
1429	AntIn
1429	AntIn
1431	AntOut
1432	AntOut
1433	AntIn
1433	AntIn
1435	AntOut
1435	AntIn
1437	AntIn
1437	AntOut
1437	AntOut
1437	AntOut
1437	AntIn
1438	AntIn
1439	AntIn
1439	AntOut
1440	AntIn
1441	AntOut
1441	AntOut
1442	AntIn
1442	AntOut
1443	AntOut
1444	AntIn
1445	AntIn
1446	AntOut
1446	AntOut
1446	AntIn

1448 AntIn
1448 AntOut
1448 AntOut
1450 AntOut
1451 AntOut
1451 AntOut
1451 AntIn
1452 AntOut
1453 AntIn
1453 AntIn
1453 AntIn
1454 AntIn
1454 AntIn
1454 AntIn
1455 AntOut
1455 AntOut
1458 AntIn
1458 AntIn
1459 AntIn
1459 AntIn
1460 AntIn
1460 AntIn
1460 AntIn
1462 AntOut
1463 AntOut
1463 AntOut
1464 AntIn
1464 AntIn
1465 AntIn
1466 AntOut
1467 AntIn
1467 AntIn
1468 AntIn
1469 AntIn
1470 AntIn
1470 AntIn
1471 AntIn
1471 AntIn
1471 AntIn
1473 AntIn
1473 AntIn
1474 AntIn

1474	AntOut
1474	AntOut
1475	AntOut
1477	AntIn
1478	AntOut
1479	AntOut
1480	AntOut
1480	AntOut
1482	AntOut
1482	AntOut
1484	AntOut
1484	AntOut
1484	AntIn
1485	AntIn
1486	AntIn
1488	AntOut
1488	AntOut
1489	AntIn
1489	AntIn
1490	AntIn
1491	AntIn
1492	AntIn
1492	AntIn
1494	AntIn
1495	AntIn
1495	AntIn
1496	AntOut
1496	AntIn
1496	AntIn
1498	AntIn
1498	AntOut
1498	AntOut
1499	AntOut
1501	AntOut
1501	AntOut
1502	AntOut
1503	AntOut
1503	AntOut
1504	AntIn
1506	AntIn
1507	AntIn
1508	AntIn

1508	AntIn
1509	AntOut
1510	AntOut
1510	AntOut
1510	AntOut
1510	AntOut
1510	AntOut
1511	AntIn
1512	AntIn
1512	AntOut
1512	AntOut
1513	AntIn
1513	AntIn
1514	AntOut
1515	AntOut
1515	AntOut
1515	AntOut
1515	AntOut
1517	AntIn
1518	AntIn
1519	AntIn
1520	AntIn
1520	AntOut
1521	AntOut
1521	AntOut
1521	AntIn
1522	AntIn
1523	AntIn
1523	AntOut
1525	AntIn
1525	AntIn
1526	AntIn
1526	AntOut
1527	AntOut
1529	AntIn
1529	AntIn
1530	AntIn
1530	AntOut
1531	AntOut
1532	AntIn
1532	AntOut
1534	AntIn

1535 AntOut
1536 AntIn
1537 AntIn
1537 AntIn
1538 AntIn
1539 AntIn
1540 AntIn
1542 AntOut
1542 AntOut
1543 AntIn
1543 AntIn
1545 AntOut
1546 AntIn
1547 AntIn
1547 AntOut
1548 AntOut
1548 AntOut
1548 AntOut
1548 AntOut
1548 AntOut
1549 AntOut
1549 AntOut
1549 AntOut
1549 AntOut
1549 AntOut
1550 AntOut
1550 AntOut
1551 AntOut
1552 AntIn
1552 AntOut
1552 AntOut
1554 AntIn
1555 AntIn
1556 AntIn
1556 AntIn
1557 AntOut
1558 AntOut
1558 AntOut
1559 AntOut
1559 AntOut
1561 AntIn
1561 AntIn

1561	AntIn
1561	AntIn
1561	AntOut
1562	AntOut
1563	AntOut
1564	AntOut
1565	AntIn
1565	AntIn
1568	AntIn
1569	AntIn
1569	AntOut
1569	AntOut
1572	AntIn
1572	AntIn
1573	AntIn
1574	AntIn
1574	AntIn
1575	AntOut
1577	AntOut
1578	AntOut
1579	AntIn
1579	AntIn
1579	AntIn
1579	AntIn
1579	AntIn
1580	AntOut
1581	AntIn
1581	AntIn
1581	AntOut
1582	AntIn
1583	AntIn
1584	AntIn
1584	AntIn
1585	AntOut
1586	AntOut
1586	AntOut
1587	AntOut
1587	AntIn
1588	AntIn
1589	AntIn
1590	AntOut
1591	AntOut

1591 AntIn
1592 AntOut
1593 AntOut
1593 AntOut
1595 AntOut
1595 AntOut
1595 AntOut
1595 AntOut
1595 AntOut
1596 AntOut
1596 AntIn
1597 AntIn
1597 AntOut
1597 AntOut
1598 AntOut
1598 AntOut
1598 AntOut
1599 AntOut
1599 AntIn
1599 AntIn
1599 AntIn
1600 AntOut
1600 AntOut
1600 AntOut
1600 AntOut
1601 AntOut
1601 AntOut
1601 AntOut
1601 AntOut
1602 AntIn
1602 AntIn
1602 AntOut
1603 AntIn
1604 AntIn
1604 AntIn
1604 AntIn
1604 AntIn
1608 AntOut
1608 AntOut
1609 AntOut
1609 AntOut
1610 AntOut

1610	AntOut
1610	AntOut
1611	AntIn
1611	AntIn
1612	AntOut
1612	AntOut
1613	AntOut
1614	AntOut
1615	AntIn
1616	AntOut
1616	AntOut
1617	AntOut
1617	AntOut
1617	AntOut
1618	AntOut
1618	AntIn
1618	AntIn
1619	AntIn
1619	AntOut
1620	AntOut
1620	AntOut
1620	AntOut
1620	AntOut
1622	AntIn
1623	AntIn
1624	AntIn
1625	AntIn
1626	AntOut
1627	AntOut
1627	AntOut
1627	AntOut
1628	AntOut
1628	AntOut
1628	AntOut
1628	AntOut
1629	AntOut
1629	AntOut
1629	AntOut
1630	AntOut
1630	AntOut
1630	AntOut
1630	AntOut

1631 AntIn
1632 AntIn
1632 AntIn
1633 AntIn
1633 AntIn
1634 AntIn
1635 AntIn
1635 AntIn
1637 AntOut
1637 AntOut
1638 AntOut
1638 AntOut
1639 AntOut
1639 AntOut
1639 AntOut
1639 AntIn
1639 AntIn
1640 AntIn
1640 AntIn
1640 AntIn
1640 AntIn
1641 AntIn
1641 AntOut
1641 AntOut
1642 AntOut
1643 AntOut
1646 AntOut
1647 AntOut
1648 AntIn
1649 AntIn
1650 AntOut
1650 AntOut
1651 AntOut
1651 AntOut
1651 AntOut
1652 AntOut
1652 AntOut
1655 AntOut
1655 AntOut
1656 AntIn
1657 AntIn
1660 AntOut

1661 AntOut
1662 AntOut
1662 AntOut
1663 AntOut
1663 AntOut
1664 AntOut
1664 AntOut
1664 AntOut
1665 AntOut
1665 AntOut
1665 AntOut
1666 AntIn
1667 AntIn
1667 AntIn
1668 AntIn
1668 AntOut
1669 AntOut
1669 AntOut
1669 AntOut
1670 AntIn
1670 AntIn
1671 AntOut
1671 AntOut
1671 AntOut
1672 AntOut
1672 AntOut
1672 AntOut
1672 AntOut
1674 AntIn
1675 AntOut
1675 AntOut
1675 AntOut
1676 AntOut
1676 AntOut
1676 AntOut
1676 AntOut
1677 AntOut
1677 AntOut
1677 AntOut
1679 AntOut
1679 AntIn
1680 AntOut

1682	AntOut
1682	AntOut
1683	AntOut
1683	AntOut
1683	AntIn
1684	AntOut
1684	AntOut
1685	AntOut
1686	AntOut
1687	AntOut
1687	AntOut
1687	AntOut
1688	AntOut
1690	AntIn
1691	AntIn
1691	AntIn
1692	AntIn
1693	AntOut
1694	AntIn
1696	AntOut
1696	AntOut
1697	AntOut
1697	AntIn
1698	AntIn
1698	AntOut
1698	AntIn
1699	AntIn
1699	AntIn
1699	AntIn
1699	AntIn
1700	AntOut
1700	AntOut
1700	AntOut
1701	AntOut
1702	AntIn
1702	AntIn
1702	AntIn
1703	AntIn
1704	AntIn
1704	AntIn
1706	AntOut
1708	AntIn

1708 AntIn
1709 AntIn
1709 AntIn
1709 AntIn
1710 AntIn
1711 AntIn
1711 AntOut
1712 AntIn
1712 AntIn
1713 AntIn
1713 AntOut
1714 AntOut
1714 AntOut
1717 AntIn
1718 AntIn
1718 AntIn
1719 AntIn
1719 AntOut
1719 AntOut
1721 AntOut
1721 AntOut
1722 AntIn
1723 AntIn
1724 AntIn
1725 AntIn
1725 AntIn
1726 AntOut
1728 AntIn
1728 AntIn
1729 AntIn
1729 AntIn
1729 AntIn
1729 AntOut
1729 AntOut
1730 AntOut
1730 AntIn
1731 AntIn
1731 AntIn
1733 AntIn
1733 AntIn
1734 AntIn
1734 AntIn

1734	AntOut
1734	AntOut
1735	AntOut
1735	AntOut
1735	AntOut
1736	AntOut
1736	AntOut
1736	AntOut
1737	AntOut
1737	AntOut
1737	AntOut
1738	AntIn
1738	AntIn
1739	AntIn
1740	AntIn
1740	AntIn
1740	AntIn
1741	AntIn
1741	AntOut
1742	AntOut
1742	AntOut
1742	AntOut
1742	AntOut
1743	AntOut
1743	AntOut
1744	AntOut
1744	AntOut
1744	AntOut
1745	AntIn
1745	AntIn
1745	AntIn
1745	AntOut
1746	AntOut
1747	AntOut
1747	AntOut
1747	AntIn
1748	AntOut
1749	AntIn
1749	AntIn
1751	AntOut
1751	AntOut
1751	AntIn

1752	AntIn
1753	AntOut
1754	AntOut
1754	AntOut
1754	AntOut
1755	AntOut
1756	AntIn
1756	AntIn
1757	AntIn
1758	AntOut
1760	AntOut
1760	AntOut
1761	AntOut
1762	AntIn
1762	AntIn
1762	AntIn
1764	AntOut
1765	AntIn
1765	AntIn
1765	AntOut
1765	AntOut
1766	AntOut
1767	AntOut
1768	AntOut
1768	AntIn
1769	AntIn
1770	AntIn
1770	AntIn
1771	AntIn
