# Supplementary material for: Interactions Increase Forager Availability and Activity in Harvester Ants
Source: PLoS One. 2015 Nov 5;10(11):e0141971. doi: 10.1371/journal.pone.0141971 (PMC4635008; doi:10.1371/journal.pone.0141971)
Supplement: S3 Dataset — We observed and filmed behavior inside the nest during and after forager removals. This dataset shows our counts made from the films of the numbers of returning and outgoing foragers at the nest entrance and the number of ascending and descending ants at all tunnel entrances. (ZIP) [file pone.0141971.s004.zip › S3 Dataset/2013 Correlation Data 229 8-26.pdf]

Researcher	Jovel Queirolo
Colony 229	
8/26/13	
Video time (seconds)	Event
6	Ascend
7	Ascend
7	Ascend
7	Ascend
8	Ascend
10	Ascend
10	Descend
11	Descend
12	Descend
13	Descend
14	Ascend
14	Descend
15	Descend
15	Ascend
15	Ascend
16	Descend
16	Descend
17	Ascend
17	Ascend
17	Descend
18	Descend
18	Descend
18	Descend
19	Descend
19	Descend
19	Descend
20	Descend
20	Ascend
21	Ascend
21	Ascend
21	Ascend
22	Ascend
22	Ascend
23	Ascend
23	Ascend
24	Ascend
24	Ascend

24	Ascend
26	Ascend
26	Ascend
27	Ascend
27	Ascend
27	Ascend
28	Ascend
28	Ascend
29	Ascend
29	Ascend
30	Ascend
31	Descend
31	Descend
31	Ascend
31	Ascend
32	Ascend
32	Ascend
32	Descend
33	Descend
33	Descend
33	Ascend
33	Descend
34	Ascend
35	Ascend
35	Descend
35	Descend
36	Descend
36	Descend
36	Ascend
37	Ascend
38	Ascend
38	Ascend
39	Ascend
39	Ascend
40	Ascend
40	Descend
40	Descend
41	Descend
41	Descend
42	Descend
42	Descend
42	Descend

43	Descend
43	Descend
43	Descend
44	Descend
44	Descend
45	Descend
45	Descend
45	Descend
46	Descend
46	Descend
46	Descend
47	Descend
48	Descend
49	Descend
52	Descend
57	Descend
58	Ascend
58	Ascend
59	Ascend
60	Ascend
60	Ascend
62	Descend
62	Descend
62	Ascend
62	Descend
63	Descend
63	Descend
65	Descend
65	Descend
65	Descend
66	Descend
67	Descend
67	Descend
68	Descend
68	Descend
69	Descend
69	Descend
70	Descend
70	Descend
71	Descend
72	Descend
72	Descend

73	Descend
74	Descend
74	Ascend
74	Ascend
74	Ascend
75	Ascend
75	Ascend
78	Ascend
78	Ascend
80	Descend
80	Ascend
80	Ascend
81	Ascend
81	Ascend
81	Ascend
82	Ascend
82	Ascend
83	Ascend
83	Ascend
84	Descend
84	Descend
84	Descend
85	Descend
85	Ascend
85	Ascend
86	Descend
86	Ascend
87	Descend
87	Ascend
87	Ascend
87	Ascend
88	Ascend
88	Ascend
88	Ascend
89	Ascend
89	Ascend
89	Ascend
90	Ascend
90	Ascend
90	Ascend
91	Ascend
91	Ascend

91	Ascend
91	Descend
92	Descend
92	Descend
92	Descend
93	Descend
93	Descend
93	Descend
94	Descend
94	Descend
95	Descend
95	Descend
95	Ascend
96	Ascend
96	Ascend
96	Ascend
96	Descend
97	Descend
97	Descend
97	Ascend
97	Ascend
98	Ascend
98	Ascend
98	Descend
99	Descend
99	Descend
99	Descend
99	Ascend
100	Ascend
100	Ascend
101	Ascend
101	Descend
101	Descend
101	Descend
102	Descend
103	Ascend
103	Ascend
104	Ascend
104	Descend
104	Ascend
104	Ascend
105	Ascend

105	Ascend
105	Ascend
106	Descend
106	Ascend
106	Ascend
107	Ascend
107	Ascend
108	Ascend
108	Ascend
108	Ascend
108	Ascend
109	Ascend
109	Descend
109	Descend
109	Descend
110	Descend
110	Descend
110	Descend
111	Ascend
111	Ascend
111	Ascend
111	Ascend
112	Ascend
112	Ascend
112	Ascend
113	Ascend
113	Ascend
113	Ascend
114	Ascend
114	Ascend
114	Ascend
115	Ascend
115	Ascend
115	Ascend
116	Ascend
116	Ascend
116	Ascend
117	Ascend
117	Ascend
117	Ascend
118	Ascend
118	Ascend

118	Ascend
119	Ascend
119	Ascend
119	Ascend
120	Ascend
120	Ascend
120	Ascend
120	Ascend
120	Ascend
121	Ascend
121	Ascend
121	Ascend
121	Ascend
121	Ascend
122	Ascend
122	Ascend
122	Ascend
122	Ascend
123	Ascend
123	Ascend
123	Ascend
123	Ascend
123	Ascend
124	Ascend
125	Ascend
125	Ascend
125	Ascend
126	Ascend
126	Ascend
127	Ascend
127	Ascend
127	Ascend
127	Ascend
128	Ascend
128	Descend
128	Descend
129	Ascend
129	Ascend
129	Ascend
130	Ascend
130	Ascend
130	Ascend

131	Ascend
131	Ascend
132	Descend
132	Descend
132	Descend
133	Descend
134	Descend
134	Descend
134	Descend
134	Ascend
134	Ascend
135	Ascend
135	Ascend
136	Ascend
136	Ascend
136	Descend
136	Descend
137	Descend
137	Descend
137	Descend
138	Descend
138	Descend
138	Ascend
138	Descend
139	Ascend
139	Descend
140	Descend
140	Descend
141	Descend
141	Descend
142	Descend
142	Ascend
142	Ascend
142	Ascend
142	Ascend
143	Ascend
143	Ascend
143	Ascend
144	Ascend
144	Ascend
144	Ascend
145	Ascend

145	Ascend
145	Ascend
145	Descend
146	Descend
146	Descend
146	Descend
147	Descend
147	Descend
148	Descend
148	Descend
148	Descend
150	Ascend
150	Ascend
150	Descend
151	Descend
151	Descend
151	Descend
151	Ascend
152	Ascend
152	Descend
152	Descend
152	Ascend
153	Ascend
153	Ascend
153	Ascend
153	Ascend
154	Ascend
154	Ascend
154	Ascend
155	Ascend
155	Ascend
155	Ascend
155	Ascend
156	Ascend
156	Ascend
156	Descend
156	Descend
157	Descend
157	Descend
160	Ascend
160	Ascend
160	Ascend

160	Ascend
161	Ascend
161	Ascend
161	Ascend
161	Ascend
162	Ascend
162	Ascend
163	Descend
163	Descend
164	Ascend
164	Ascend
164	Descend
164	Descend
165	Ascend
165	Ascend
165	Descend
166	Descend
166	Ascend
166	Ascend
167	Descend
167	Ascend
168	Ascend
168	Ascend
169	Ascend
170	Descend
171	Descend
171	Descend
173	Descend
173	Descend
174	Descend
175	Descend
175	Ascend
176	Descend
177	Descend
177	Ascend
177	Ascend
177	Ascend
178	Ascend
178	Ascend
178	Ascend
179	Ascend
179	Ascend

179	Ascend
179	Descend
180	Descend
180	Descend
180	Ascend
180	Ascend
181	Ascend
181	Ascend
181	Descend
182	Descend
182	Descend
183	Descend
183	Descend
184	Descend
184	Descend
184	Descend
185	Descend
185	Descend
185	Descend
186	Descend
186	Ascend
186	Ascend
187	Ascend
187	Ascend
187	Descend
187	Descend
188	Descend
188	Descend
188	Descend
188	Ascend
189	Ascend
189	Ascend
189	Ascend
190	Ascend
190	Ascend
191	Descend
191	Descend
191	Ascend
191	Ascend
192	Ascend
192	Ascend
192	Descend

193	Descend
193	Descend
193	Ascend
193	Ascend
194	Descend
194	Descend
194	Descend
195	Descend
195	Descend
195	Descend
196	Ascend
196	Ascend
196	Ascend
196	Ascend
197	Ascend
197	Ascend
197	Ascend
198	Ascend
198	Ascend
198	Ascend
198	Ascend
199	Ascend
199	Ascend
200	Descend
200	Descend
200	Ascend
200	Ascend
201	Descend
201	Descend
201	Descend
202	Descend
202	Descend
202	Descend
202	Ascend
202	Ascend
203	Descend
203	Descend
203	Descend
203	Descend
204	Ascend
204	Ascend
204	Ascend

204	Descend
205	Ascend
205	Descend
205	Descend
206	Descend
206	Ascend
206	Ascend
206	Ascend
207	Ascend
207	Ascend
207	Ascend
208	Ascend
208	Ascend
208	Ascend
208	Ascend
209	Ascend
209	Ascend
209	Ascend
209	Ascend
209	Ascend
210	Ascend
210	Ascend
210	Ascend
210	Ascend
211	Ascend
211	Ascend
211	Ascend
211	Ascend
212	Ascend
212	Ascend
212	Descend
213	Descend
213	Descend
213	Descend
213	Descend
214	Descend
214	Ascend
214	Ascend
214	Ascend
215	Ascend
215	Ascend
215	Descend

215	Descend
215	Ascend
216	Ascend
216	Ascend
216	Ascend
217	Ascend
217	Ascend
217	Ascend
218	Ascend
218	Ascend
218	Descend
218	Descend
219	Descend
219	Descend
219	Descend
220	Descend
220	Descend
220	Descend
220	Descend
221	Descend
221	Descend
221	Descend
221	Descend
222	Descend
223	Descend
223	Ascend
223	Ascend
223	Ascend
224	Ascend
224	Descend
224	Descend
224	Descend
225	Descend
225	Descend
225	Descend
225	Descend
226	Ascend
226	Ascend
226	Ascend
226	Ascend
227	Ascend
227	Descend

227	Descend
228	Descend
228	Descend
228	Descend
228	Descend
229	Descend
229	Descend
229	Descend
230	Descend
230	Descend
230	Ascend
231	Ascend
231	Ascend
231	Ascend
232	Descend
232	Descend
232	Ascend
232	Ascend
233	Ascend
233	Ascend
234	Ascend
234	Ascend
234	Descend
234	Descend
234	Descend
235	Ascend
235	Ascend
235	Ascend
235	Descend
236	Descend
236	Descend
236	Descend
236	Descend
237	Ascend
237	Ascend
237	Ascend
237	Descend
237	Descend
238	Ascend
238	Ascend
238	Ascend
238	Ascend

238	Ascend
239	Ascend
239	Ascend
239	Ascend
239	Ascend
240	Ascend
240	Ascend
240	Ascend
240	Ascend
241	Descend
241	Descend
241	Ascend
241	Ascend
242	Ascend
242	Ascend
242	Descend
243	Descend
243	Descend
243	Descend
243	Descend
244	Descend
244	Ascend
244	Ascend
244	Ascend
245	Ascend
245	Ascend
245	Ascend
245	Ascend
246	Ascend
246	Ascend
246	Ascend
247	Ascend
247	Ascend
247	Ascend
248	Ascend
248	Ascend
248	Descend
249	Descend
249	Descend
249	Ascend
250	Descend
252	Descend

253	Descend
253	Ascend
253	Ascend
254	Ascend
255	Ascend
256	Ascend
256	Ascend
257	Ascend
258	Ascend
258	Ascend
258	Ascend
259	Ascend
259	Descend
259	Descend
259	Descend
260	Descend
260	Ascend
260	Ascend
261	Ascend
261	Ascend
261	Descend
262	Descend
262	Ascend
262	Ascend
263	Ascend
263	Ascend
264	Ascend
264	Ascend
264	Ascend
265	Ascend
265	Descend
265	Descend
265	Descend
265	Ascend
266	Ascend
266	Ascend
266	Ascend
267	Ascend
267	Ascend
268	Ascend
268	Descend
268	Descend

268	Descend
268	Descend
269	Descend
269	Descend
269	Descend
270	Descend
270	Descend
270	Descend
271	Descend
271	Descend
271	Descend
272	Descend
272	Descend
272	Descend
272	Descend
273	Descend
273	Descend
274	Descend
274	Descend
274	Descend
274	Descend
275	Descend
275	Descend
276	Descend
276	Descend
276	Descend
279	Ascend
279	Ascend
279	Ascend
279	Ascend
280	Ascend
280	Ascend
281	Ascend
281	Ascend
281	Ascend
282	Ascend
282	Ascend
283	Ascend
284	Ascend
285	Ascend
285	Descend
286	Descend

286	Ascend
287	Ascend
287	Descend
287	Descend
288	Descend
288	Descend
289	Descend
289	Descend
289	Descend
290	Descend
290	Descend
290	Descend
291	Descend
291	Descend
292	Descend
292	Descend
292	Descend
293	Descend
293	Descend
293	Descend
294	Descend
294	Descend
295	Descend
295	Descend
295	Descend
296	Descend
296	Descend
296	Descend
296	Descend
297	Descend
298	Descend
298	Descend
299	Descend
299	Descend
300	Descend
300	Descend
300	Descend
301	Descend
301	Descend
301	Descend
302	Descend
302	Descend

302	Descend
304	Descend
304	Descend
305	Descend
305	Descend
305	Descend
306	Descend
306	Descend
306	Descend
307	Descend
307	Descend
308	Descend
308	Descend
308	Descend
309	Descend
309	Descend
309	Descend
309	Descend
310	Descend
310	Descend
310	Descend
310	Descend
311	Descend
311	Descend
311	Descend
312	Descend
312	Descend
313	Descend
313	Descend
313	Descend
314	Descend
314	Descend
314	Descend
315	Descend
315	Descend
315	Descend
315	Descend
316	Descend
316	Descend
316	Descend
317	Descend
317	Descend

317	Descend
317	Descend
318	Descend
318	Descend
319	Descend
319	Descend
319	Descend
319	Descend
320	Descend
320	Descend
321	Descend
321	Descend
322	Descend
322	Descend
322	Descend
322	Descend
323	Descend
323	Descend
323	Descend
324	Descend
324	Descend
324	Descend
325	Descend
325	Descend
326	Descend
326	Descend
326	Descend
327	Ascend
328	Ascend
328	Ascend
328	Ascend
328	Descend
329	Descend
329	Descend
329	Descend
329	Descend
330	Descend
330	Ascend
330	Ascend
330	Ascend
331	Ascend
331	Ascend

331	Ascend
331	Ascend
332	Ascend
332	Ascend
332	Ascend
332	Ascend
333	Ascend
333	Ascend
333	Ascend
333	Ascend
334	Ascend
334	Ascend
334	Ascend
334	Ascend
334	Ascend
335	Ascend
335	Ascend
336	Ascend
337	Ascend
338	Ascend
338	Ascend
339	Ascend
339	Ascend
340	Ascend
340	Ascend
341	Ascend
341	Ascend
342	Ascend
342	Descend
342	Descend
343	Descend
343	Descend
343	Descend
344	Descend
344	Descend
344	Descend
345	Descend
345	Descend
346	Descend
346	Descend
347	Descend
347	Ascend

347	Ascend
348	Ascend
348	Ascend
349	Ascend
349	Ascend
349	Ascend
350	Ascend
350	Ascend
350	Ascend
351	Ascend
351	Descend
351	Descend
352	Ascend
352	Descend
352	Descend
353	Descend
354	Ascend
354	Ascend
354	Ascend
355	Ascend
355	Ascend
355	Ascend
355	Ascend
356	Ascend
356	Ascend
356	Ascend
357	Ascend
357	Ascend
357	Ascend
358	Ascend
358	Ascend
358	Ascend
358	Ascend
358	Ascend
359	Ascend
359	Ascend
360	Ascend
360	Ascend
360	Ascend
360	Ascend
361	Ascend
361	Ascend

362	Ascend
362	Ascend
362	Descend
363	Descend
365	Ascend
367	Ascend
368	Ascend
368	Ascend
368	Ascend
368	Ascend
369	Descend
369	Descend
369	Ascend
369	Ascend
369	Ascend
370	Descend
370	Descend
370	Ascend
370	Ascend
371	Ascend
371	Ascend
372	Descend
372	Descend
372	Ascend
372	Ascend
373	Ascend
373	Ascend
373	Ascend
373	Ascend
374	Ascend
374	Ascend
374	Ascend
374	Ascend
375	Ascend
375	Ascend
375	Ascend
376	Ascend
376	Ascend
376	Ascend
376	Descend
376	Descend
377	Descend

377	Descend
377	Ascend
377	Ascend
378	Ascend
378	Ascend
378	Ascend
378	Ascend
379	Ascend
379	Descend
379	Descend
380	Descend
380	Descend
380	Descend
381	Descend
381	Ascend
381	Ascend
381	Ascend
382	Descend
382	Descend
382	Descend
383	Ascend
383	Ascend
383	Ascend
383	Descend
384	Descend
384	Descend
384	Descend
384	Descend
385	Ascend
385	Ascend
385	Ascend
385	Ascend
386	Ascend
386	Ascend
386	Descend
386	Descend
387	Ascend
387	Descend
387	Descend
387	Descend
387	Descend
388	Descend

388	Descend
388	Ascend
388	Ascend
389	Ascend
389	Ascend
389	Ascend
389	Ascend
390	Ascend
390	Ascend
390	Descend
391	Ascend
391	Ascend
391	Descend
391	Ascend
392	Ascend
392	Descend
392	Descend
392	Descend
393	Descend
393	Descend
393	Descend
394	Descend
395	Descend
395	Descend
395	Descend
396	Descend
396	Descend
397	Descend
397	Ascend
397	Ascend
397	Ascend
398	Ascend
398	Descend
398	Descend
399	Descend
399	Descend
399	Descend
399	Descend
400	Descend
400	Descend
400	Descend
401	Descend

401	Descend
401	Ascend
401	Ascend
402	Ascend
403	Ascend
403	Descend
403	Ascend
403	Descend
404	Descend
404	Descend
404	Descend
404	Descend
404	Ascend
405	Ascend
405	Descend
405	Descend
405	Ascend
406	Ascend
406	Descend
406	Descend
406	Descend
407	Descend
407	Descend
407	Descend
408	Descend
408	Descend
408	Descend
408	Descend
409	Descend
409	Descend
409	Descend
410	Descend
410	Descend
410	Ascend
410	Descend
410	Ascend
411	Ascend
411	Ascend
411	Ascend
412	Ascend
412	Ascend
412	Ascend

413	Ascend
413	Descend
413	Descend
413	Descend
414	Descend
414	Descend
414	Descend
414	Descend
415	Descend
415	Descend
415	Descend
416	Descend
416	Descend
416	Descend
417	Descend
417	Descend
417	Descend
418	Descend
418	Descend
418	Descend
419	Descend
419	Descend
419	Descend
420	Descend
420	Descend
420	Descend
420	Descend
421	Descend
421	Descend
421	Descend
421	Descend
422	Descend
422	Descend
422	Descend
423	Descend
423	Descend
423	Descend
423	Descend
424	Descend
424	Descend
424	Descend
425	Descend

425	Descend
425	Descend
426	Descend
426	Descend
426	Descend
427	Descend
427	Descend
427	Descend
428	Descend
428	Descend
428	Descend
429	Descend
429	Descend
429	Descend
429	Descend
430	Descend
430	Descend
430	Descend
431	Descend
431	Descend
431	Descend
432	Descend
432	Descend
432	Descend
432	Descend
433	Descend
433	Descend
433	Descend
434	Descend
434	Descend
434	Descend
435	Descend
435	Descend
435	Descend
436	Descend
436	Descend
436	Descend
436	Descend
437	Descend
437	Descend
437	Descend
438	Descend

438	Descend
438	Descend
439	Descend
439	Descend
440	Descend
440	Descend
440	Descend
441	Descend
441	Descend
441	Descend
442	Descend
442	Descend
442	Descend
443	Descend
443	Descend
444	Descend
444	Descend
444	Descend
445	Descend
445	Descend
445	Descend
445	Descend
446	Descend
446	Descend
447	Descend
447	Descend
448	Descend
448	Descend
448	Descend
448	Descend
449	Descend
450	Descend
451	Descend
452	Descend
452	Ascend
452	Descend
452	Ascend
452	Descend
453	Descend
453	Descend
453	Descend
454	Descend

454	Descend
455	Descend
455	Descend
456	Descend
456	Descend
457	Descend
457	Descend
458	Descend
458	Descend
458	Descend
459	Descend
459	Descend
460	Descend
460	Descend
461	Descend
461	Descend
461	Descend
462	Descend
462	Descend
463	Descend
464	Descend
464	Descend
465	Descend
465	Descend
465	Descend
466	Descend
466	Descend
467	Descend
467	Descend
468	Descend
468	Descend
469	Descend
469	Descend
470	Descend
470	Descend
471	Descend
471	Descend
474	Descend
474	Ascend
474	Ascend
475	Ascend
475	Ascend

476	Ascend
476	Descend
477	Descend
477	Ascend
477	Descend
478	Ascend
478	Ascend
478	Descend
479	Descend
480	Descend
482	Descend
483	Descend
483	Descend
485	Descend
486	Descend
486	Ascend
486	Ascend
487	Descend
488	Descend
488	Ascend
489	Ascend
489	Descend
491	Descend
492	Descend
492	Ascend
493	Descend
495	Descend
495	Ascend
496	Ascend
496	Descend
497	Descend
498	Descend
500	Descend
500	Descend
501	Descend
501	Descend
503	Descend
505	Descend
507	Ascend
508	Ascend
508	Ascend
512	Ascend

514	Ascend
515	Descend
516	Ascend
516	Descend
517	Descend
517	Descend
518	Descend
520	Descend
521	Descend
523	Descend
524	Ascend
526	Descend
527	Ascend
527	Ascend
528	Ascend
529	Ascend
530	Ascend
530	Ascend
531	Ascend
532	Ascend
533	Ascend
536	Ascend
549	Descend
554	Descend
560	Ascend
568	Descend
569	Ascend
575	Descend
578	Ascend
580	Descend
583	Descend
585	Ascend
588	Descend
588	Ascend
590	Ascend
592	Descend
593	Descend
596	Descend
598	Descend
600	Descend
603	Descend
603	Ascend

604	Descend
605	Descend
609	Descend
628	Descend
630	Descend
635	Descend
636	Ascend
639	Descend
640	Descend
642	Ascend
642	Descend
662	Descend
670	Ascend
672	Descend
673	Ascend
677	Descend
690	Descend
693	Descend
698	Descend
726	Ascend
726	Descend
729	Descend
736	Ascend
738	Descend
740	Ascend
741	Descend
745	Ascend
747	Descend
754	Descend
754	Descend
755	Ascend
760	Descend
766	Descend
774	Ascend
775	Ascend
775	Ascend
776	Ascend
781	Descend
782	Descend
786	Ascend
789	Descend
791	Ascend

793	Ascend
798	Ascend
800	Ascend
800	Ascend
800	Ascend
800	Ascend
801	Ascend
801	Ascend
801	Ascend
802	Ascend
803	Ascend
803	Ascend
805	Ascend
807	Ascend
810	Descend
814	Descend
815	Descend
818	Ascend
820	Descend
821	Ascend
826	Descend
837	Descend
837	Ascend
838	Ascend
839	Ascend
841	Ascend
841	Descend
842	Descend
843	Ascend
843	Descend
844	Ascend
844	Ascend
844	Ascend
845	Ascend
846	Descend
847	Descend
847	Ascend
847	Ascend
847	Ascend
848	Ascend
848	Ascend
849	Ascend

849	Ascend
850	Descend
850	Descend
851	Descend
853	Ascend
854	Ascend
855	Ascend
856	Descend
858	Descend
859	Descend
860	Ascend
860	Ascend
862	Descend
863	Ascend
874	Descend
877	Descend
879	Descend
879	Descend
880	Descend
883	Ascend
884	Descend
886	Ascend
887	Ascend
890	Ascend
895	Descend
897	Descend
898	Ascend
11	AntIn
18	AntIn
25	AntOut
26	AntIn
28	AntIn
30	AntOut
31	AntIn
37	AntIn
39	AntIn
41	AntIn
51	AntIn
53	AntOut
57	AntIn
61	AntIn
62	AntIn

63	AntIn
64	AntIn
65	AntOut
66	AntIn
66	AntIn
67	AntIn
69	AntIn
77	AntOut
78	AntIn
83	AntIn
85	AntIn
85	AntOut
86	AntOut
89	AntOut
91	AntIn
91	AntIn
94	AntIn
96	AntIn
97	AntIn
98	AntOut
98	AntOut
99	AntOut
100	AntOut
100	AntOut
101	AntOut
102	AntOut
105	AntIn
106	AntOut
107	AntIn
108	AntOut
109	AntOut
110	AntIn
112	AntOut
114	AntOut
115	AntIn
116	AntOut
116	AntOut
118	AntOut
119	AntOut
119	AntOut
120	AntOut
120	AntOut

122	AntIn
123	AntOut
124	AntOut
127	AntOut
128	AntOut
129	AntOut
130	AntOut
131	AntOut
132	AntIn
133	AntOut
134	AntIn
135	AntOut
136	AntOut
137	AntOut
137	AntOut
137	AntOut
139	AntOut
140	AntIn
140	AntIn
142	AntOut
143	AntOut
143	AntIn
144	AntIn
145	AntOut
146	AntOut
146	AntOut
149	AntOut
149	AntOut
149	AntIn
151	AntIn
152	AntIn
153	AntIn
154	AntOut
156	AntOut
157	AntIn
158	AntIn
158	AntOut
159	AntOut
161	AntOut
161	AntIn
162	AntIn
165	AntIn

166	AntOut
167	AntOut
168	AntOut
168	AntIn
172	AntIn
173	AntIn
173	AntOut
175	AntIn
175	AntIn
178	AntOut
180	AntOut
180	AntOut
181	AntOut
182	AntIn
183	AntOut
183	AntIn
184	AntIn
187	AntIn
190	AntIn
191	AntOut
191	AntOut
192	AntOut
192	AntIn
195	AntOut
195	AntIn
198	AntIn
199	AntOut
199	AntOut
200	AntIn
203	AntIn
204	AntIn
206	AntIn
206	AntIn
208	AntIn
210	AntIn
211	AntOut
211	AntOut
212	AntOut
213	AntOut
213	AntOut
213	AntOut
215	AntOut

216	AntIn
219	AntOut
219	AntIn
220	AntOut
221	AntIn
222	AntOut
222	AntOut
223	AntOut
223	AntOut
224	AntOut
225	AntIn
226	AntIn
226	AntIn
227	AntIn
228	AntIn
229	AntOut
232	AntOut
233	AntIn
233	AntOut
234	AntOut
234	AntOut
234	AntIn
237	AntOut
240	AntIn
241	AntIn
242	AntOut
246	AntOut
246	AntOut
246	AntOut
247	AntOut
247	AntIn
248	AntOut
249	AntIn
249	AntOut
251	AntIn
253	AntOut
254	AntIn
256	AntOut
256	AntOut
258	AntOut
259	AntIn
260	AntOut

261	AntOut
264	AntOut
264	AntOut
265	AntOut
265	AntOut
266	AntOut
266	AntOut
266	AntOut
267	AntOut
267	AntIn
267	AntIn
270	AntIn
270	AntIn
271	AntIn
271	AntOut
273	AntOut
274	AntIn
275	AntIn
276	AntIn
277	AntOut
277	AntOut
278	AntOut
278	AntOut
279	AntIn
279	AntIn
280	AntIn
280	AntOut
281	AntOut
288	AntIn
289	AntIn
289	AntIn
290	AntIn
290	AntOut
290	AntOut
292	AntIn
292	AntIn
293	AntOut
294	AntIn
294	AntOut
294	AntOut
296	AntOut
296	AntOut

296 AntOut
297 AntOut
297 AntIn
300 AntIn
300 AntIn
300 AntIn
301 AntOut
301 AntIn
302 AntIn
306 AntIn
308 AntOut
309 AntIn
313 AntIn
313 AntOut
316 AntIn
317 AntIn
317 AntIn
318 AntIn
319 AntIn
320 AntIn
320 AntOut
321 AntIn
322 AntOut
323 AntIn
324 AntOut
325 AntOut
325 AntOut
327 AntOut
327 AntIn
328 AntIn
328 AntOut
330 AntIn
330 AntOut
332 AntOut
332 AntIn
334 AntOut
335 AntIn
336 AntOut
337 AntIn
338 AntOut
339 AntOut
340 AntOut

342	AntIn
343	AntIn
344	AntIn
346	AntOut
348	AntOut
350	AntIn
351	AntIn
352	AntIn
356	AntIn
357	AntOut
358	AntIn
359	AntIn
359	AntOut
362	AntIn
363	AntIn
365	AntIn
370	AntOut
370	AntOut
370	AntOut
371	AntOut
371	AntOut
372	AntIn
372	AntIn
372	AntIn
373	AntIn
376	AntIn
376	AntIn
376	AntOut
377	AntOut
378	AntOut
378	AntOut
379	AntOut
379	AntIn
379	AntIn
380	AntIn
381	AntOut
381	AntIn
382	AntIn
382	AntIn
383	AntOut
383	AntOut
386	AntOut

388 AntOut
388 AntIn
389 AntIn
390 AntOut
392 AntOut
392 AntIn
392 AntIn
393 AntOut
395 AntIn
395 AntIn
397 AntOut
399 AntIn
399 AntOut
401 AntIn
403 AntOut
404 AntIn
404 AntOut
405 AntOut
405 AntOut
406 AntIn
406 AntIn
408 AntIn
408 AntOut
409 AntIn
410 AntOut
410 AntOut
411 AntOut
412 AntIn
415 AntIn
416 AntIn
418 AntIn
421 AntIn
422 AntIn
422 AntIn
423 AntIn
423 AntIn
424 AntIn
425 AntIn
426 AntIn
428 AntOut
429 AntIn
431 AntIn

431	AntIn
434	AntIn
434	AntIn
435	AntOut
439	AntIn
441	AntIn
442	AntIn
445	AntIn
446	AntIn
460	AntOut
489	AntOut
495	AntIn
501	AntOut
502	AntOut
503	AntOut
504	AntOut
505	AntOut
507	AntOut
510	AntOut
514	AntOut
515	AntOut
516	AntOut
517	AntIn
517	AntOut
518	AntOut
522	AntOut
540	AntOut
550	AntOut
550	AntOut
551	AntOut
552	AntIn
552	AntIn
556	AntOut
568	AntOut
573	AntOut
582	AntIn
584	AntOut
586	AntIn
587	AntIn
590	AntOut
590	AntOut
590	AntOut

591 AntIn
594 AntIn
595 AntOut
597 AntIn
597 AntIn
597 AntOut
601 AntOut
602 AntIn
607 AntIn
607 AntIn
611 AntOut
614 AntIn
614 AntIn
617 AntOut
617 AntOut
618 AntIn
620 AntIn
621 AntOut
623 AntIn
632 AntIn
635 AntOut
637 AntIn
640 AntOut
642 AntIn
643 AntIn
643 AntIn
647 AntOut
658 AntIn
660 AntIn
667 AntOut
674 AntOut
693 AntIn
696 AntOut
697 AntIn
699 AntIn
711 AntIn
717 AntOut
718 AntIn
725 AntIn
731 AntIn
735 AntIn
737 AntIn

742	AntIn
746	AntOut
747	AntIn
748	AntIn
754	AntIn
755	AntOut
766	AntIn
772	AntOut
782	AntOut
787	AntIn
792	AntIn
803	AntOut
809	AntOut
818	AntIn
819	AntOut
837	AntIn
844	AntIn
845	AntOut
847	AntIn
852	AntIn
857	AntIn
859	AntOut
860	AntIn
873	AntIn
877	AntOut
878	AntIn
879	AntIn
888	AntOut
894	AntIn
896	AntIn
