# Supplementary material for: Interactions Increase Forager Availability and Activity in Harvester Ants
Source: PLoS One. 2015 Nov 5;10(11):e0141971. doi: 10.1371/journal.pone.0141971 (PMC4635008; doi:10.1371/journal.pone.0141971)
Supplement: S3 Dataset — We observed and filmed behavior inside the nest during and after forager removals. This dataset shows our counts made from the films of the numbers of returning and outgoing foragers at the nest entrance and the number of ascending and descending ants at all tunnel entrances. (ZIP) [file pone.0141971.s004.zip › S3 Dataset/2013 Correlation Data 242 8-25.pdf]

Researcher **Jovel Queirolo**
Colony 242

8/25/13

**Video time (seconds)**	**Event**
4	Descend
5	Ascend
7	Descend
8	Ascend
9	Descend
12	Descend
13	Descend
14	Ascend
15	Descend
20	Ascend
22	Descend
31	Ascend
33	Ascend
33	Ascend
33	Ascend
34	Ascend
37	Ascend
39	Ascend
39	Ascend
39	Ascend
40	Ascend
40	Ascend
41	Ascend
41	Ascend
43	Ascend
44	Descend
51	Ascend
52	Descend
53	Descend
53	Descend
54	Descend
54	Descend
55	Descend
55	Descend
56	Ascend
56	Ascend

57	Ascend
57	Ascend
58	Ascend
58	Ascend
58	Ascend
61	Ascend
61	Ascend
62	Ascend
62	Ascend
63	Ascend
63	Ascend
64	Ascend
64	Ascend
65	Descend
65	Descend
67	Descend
67	Descend
68	Descend
68	Descend
69	Descend
69	Descend
70	Descend
70	Descend
70	Ascend
71	Ascend
71	Ascend
72	Ascend
72	Ascend
72	Ascend
73	Ascend
73	Descend
73	Descend
75	Descend
75	Ascend
75	Ascend
75	Ascend
76	Ascend
77	Descend
78	Descend
78	Descend
79	Descend
79	Ascend

80	Descend
82	Ascend
82	Ascend
83	Ascend
83	Descend
84	Descend
85	Descend
88	Descend
89	Descend
89	Descend
90	Ascend
90	Ascend
91	Ascend
91	Ascend
91	Ascend
92	Ascend
92	Ascend
98	Ascend
98	Ascend
98	Ascend
98	Ascend
98	Ascend
99	Ascend
99	Ascend
99	Ascend
100	Ascend
102	Descend
102	Descend
103	Descend
103	Descend
104	Descend
104	Descend
106	Descend
107	Descend
107	Descend
108	Ascend
111	Ascend
111	Ascend
116	Descend
116	Descend
117	Descend
117	Descend

119	Ascend
119	Ascend
120	Ascend
120	Ascend
120	Ascend
121	Ascend
121	Ascend
121	Ascend
122	Ascend
122	Ascend
122	Ascend
123	Ascend
123	Ascend
123	Ascend
123	Ascend
124	Ascend
124	Ascend
125	Ascend
125	Ascend
126	Ascend
127	Ascend
129	Ascend
132	Descend
133	Descend
134	Descend
134	Descend
135	Descend
136	Descend
137	Descend
139	Ascend
139	Ascend
139	Ascend
140	Descend
140	Ascend
140	Descend
140	Ascend
141	Ascend
141	Descend
141	Descend
141	Descend
142	Descend
142	Ascend

142	Ascend
143	Descend
143	Descend
144	Descend
144	Descend
144	Ascend
144	Ascend
145	Ascend
146	Ascend
146	Ascend
147	Descend
147	Descend
148	Descend
149	Descend
150	Descend
150	Ascend
150	Ascend
150	Ascend
151	Ascend
151	Ascend
152	Ascend
153	Ascend
154	Ascend
154	Ascend
155	Ascend
155	Ascend
155	Ascend
156	Descend
157	Descend
157	Descend
158	Descend
158	Descend
159	Descend
160	Descend
161	Descend
162	Descend
163	Descend
164	Descend
164	Descend
166	Ascend
166	Ascend
167	Descend

167	Descend
168	Descend
168	Descend
168	Ascend
169	Ascend
169	Ascend
169	Ascend
170	Ascend
170	Ascend
170	Ascend
171	Ascend
171	Ascend
172	Ascend
172	Ascend
173	Descend
173	Descend
173	Descend
174	Descend
174	Ascend
175	Ascend
175	Ascend
175	Descend
176	Descend
176	Descend
176	Descend
176	Ascend
177	Ascend
177	Ascend
177	Ascend
178	Descend
178	Descend
178	Ascend
178	Ascend
179	Ascend
179	Ascend
179	Ascend
179	Ascend
179	Ascend
180	Ascend
180	Ascend
180	Descend
180	Descend

181	Descend
181	Descend
181	Descend
181	Descend
182	Descend
182	Ascend
182	Ascend
182	Ascend
183	Ascend
183	Ascend
183	Ascend
183	Ascend
184	Ascend
184	Ascend
184	Ascend
185	Descend
185	Descend
185	Ascend
185	Ascend
185	Ascend
186	Ascend
186	Ascend
186	Ascend
186	Ascend
187	Ascend
187	Descend
187	Descend
187	Descend
188	Ascend
188	Ascend
189	Ascend
189	Ascend
189	Descend
189	Descend
190	Ascend
190	Ascend
190	Ascend
190	Ascend
191	Ascend
191	Ascend
192	Descend
192	Descend

192	Ascend
192	Ascend
193	Ascend
193	Descend
193	Descend
193	Ascend
193	Ascend
194	Ascend
194	Ascend
194	Ascend
195	Ascend
195	Ascend
196	Ascend
196	Ascend
197	Ascend
197	Ascend
197	Descend
197	Descend
198	Descend
198	Descend
198	Ascend
198	Ascend
198	Ascend
199	Ascend
199	Ascend
199	Descend
199	Descend
200	Ascend
200	Ascend
201	Descend
201	Descend
202	Ascend
202	Ascend
202	Ascend
203	Descend
203	Descend
203	Descend
203	Descend
203	Descend
204	Descend
204	Descend
204	Descend

204	Ascend
205	Ascend
205	Ascend
205	Descend
205	Descend
206	Descend
206	Descend
206	Descend
206	Ascend
207	Ascend
207	Ascend
207	Ascend
208	Ascend
208	Ascend
208	Ascend
208	Ascend
208	Ascend
208	Ascend
209	Ascend
209	Ascend
209	Ascend
209	Ascend
209	Ascend
209	Ascend
210	Ascend
210	Ascend
210	Ascend
210	Ascend
211	Ascend
211	Ascend
211	Ascend
211	Ascend
211	Ascend
212	Ascend
212	Ascend
212	Ascend
212	Ascend
213	Ascend
213	Ascend
213	Ascend
215	Descend
215	Descend

216	Descend
216	Descend
216	Ascend
216	Ascend
217	Ascend
217	Ascend
217	Ascend
217	Ascend
217	Ascend
218	Ascend
218	Ascend
218	Ascend
218	Ascend
218	Ascend
219	Ascend
219	Ascend
219	Ascend
219	Ascend
220	Ascend
220	Ascend
220	Ascend
220	Ascend
221	Ascend
222	Ascend
222	Ascend
222	Ascend
223	Descend
223	Descend
223	Descend
223	Descend
223	Descend
224	Descend
224	Descend
224	Descend
224	Descend
225	Ascend
225	Ascend
225	Ascend
225	Ascend
225	Ascend
226	Ascend
226	Descend

226	Descend
226	Descend
226	Descend
227	Descend
227	Ascend
227	Ascend
227	Ascend
227	Descend
228	Descend
228	Descend
228	Descend
228	Descend
228	Ascend
229	Ascend
229	Ascend
229	Ascend
229	Descend
229	Descend
230	Descend
230	Descend
230	Descend
230	Ascend
231	Ascend
231	Descend
231	Descend
231	Descend
231	Descend
232	Ascend
232	Ascend
232	Ascend
232	Ascend
233	Ascend
233	Ascend
233	Ascend
233	Ascend
233	Ascend
234	Ascend
234	Ascend
234	Ascend
235	Descend
235	Descend
236	Descend

237	Ascend
237	Ascend
237	Ascend
238	Descend
238	Descend
238	Descend
239	Descend
239	Ascend
239	Ascend
239	Ascend
239	Ascend
240	Ascend
240	Ascend
240	Ascend
240	Ascend
241	Ascend
241	Ascend
242	Ascend
242	Ascend
242	Ascend
243	Ascend
243	Descend
243	Descend
244	Descend
244	Descend
244	Descend
244	Ascend
245	Ascend
245	Ascend
245	Ascend
245	Descend
246	Descend
246	Ascend
246	Ascend
246	Ascend
246	Descend
247	Descend
247	Descend
247	Ascend
247	Ascend
248	Ascend
248	Ascend

248	Ascend
248	Descend
248	Descend
249	Descend
249	Ascend
249	Ascend
249	Ascend
249	Ascend
250	Ascend
250	Ascend
250	Ascend
250	Ascend
251	Ascend
251	Ascend
251	Ascend
252	Ascend
252	Descend
252	Descend
252	Descend
252	Descend
253	Ascend
253	Ascend
253	Ascend
253	Ascend
254	Ascend
254	Ascend
254	Descend
255	Descend
256	Descend
257	Ascend
257	Ascend
257	Descend
257	Descend
258	Ascend
258	Ascend
259	Ascend
259	Ascend
259	Ascend
260	Descend
260	Descend
260	Ascend
261	Ascend

261	Ascend
261	Ascend
261	Ascend
261	Ascend
262	Ascend
262	Ascend
262	Ascend
263	Ascend
263	Descend
263	Descend
264	Descend
264	Descend
264	Ascend
264	Ascend
265	Descend
265	Descend
265	Descend
266	Descend
266	Ascend
266	Ascend
266	Ascend
267	Ascend
267	Ascend
267	Ascend
268	Ascend
268	Ascend
268	Ascend
268	Ascend
269	Ascend
269	Ascend
269	Ascend
270	Ascend
270	Ascend
271	Ascend
271	Ascend
271	Ascend
271	Ascend
272	Ascend
272	Ascend
272	Descend
273	Descend
273	Descend

273	Descend
273	Descend
274	Descend
274	Descend
274	Descend
274	Descend
275	Descend
275	Descend
275	Descend
275	Descend
276	Descend
276	Ascend
276	Ascend
276	Ascend
277	Ascend
277	Ascend
277	Ascend
278	Ascend
278	Ascend
278	Ascend
279	Ascend
279	Ascend
279	Ascend
280	Ascend
280	Descend
280	Descend
280	Descend
281	Descend
281	Ascend
281	Ascend
282	Ascend
282	Ascend
282	Ascend
283	Descend
283	Descend
283	Descend
284	Descend
284	Descend
284	Descend
285	Descend
285	Descend
287	Descend

287	Descend
289	Descend
289	Descend
289	Ascend
289	Ascend
290	Ascend
290	Ascend
290	Ascend
290	Ascend
291	Ascend
291	Ascend
291	Ascend
291	Ascend
292	Ascend
292	Ascend
292	Descend
293	Descend
293	Descend
293	Ascend
293	Ascend
293	Ascend
294	Descend
294	Descend
295	Ascend
295	Ascend
296	Ascend
297	Descend
297	Descend
298	Ascend
298	Ascend
299	Ascend
299	Ascend
300	Descend
300	Descend
301	Descend
301	Descend
301	Ascend
301	Ascend
302	Ascend
303	Ascend
303	Ascend
304	Ascend

305	Ascend
305	Ascend
306	Ascend
306	Ascend
307	Ascend
307	Ascend
308	Descend
308	Descend
309	Descend
309	Ascend
309	Ascend
310	Ascend
310	Descend
311	Descend
311	Ascend
311	Ascend
311	Ascend
312	Ascend
312	Ascend
312	Ascend
313	Ascend
314	Ascend
314	Descend
315	Descend
315	Descend
315	Descend
316	Ascend
316	Ascend
316	Ascend
316	Ascend
317	Ascend
317	Ascend
318	Ascend
318	Descend
319	Descend
319	Descend
319	Descend
320	Descend
320	Ascend
320	Descend
321	Descend
321	Ascend

321 Ascend
321 Ascend
322 Descend
322 Descend
322 Descend
323 Ascend
323 Ascend
323 Descend
323 Descend
324 Descend
324 Descend
324 Ascend
325 Ascend
325 Descend
325 Descend
326 Ascend
326 Ascend
326 Ascend
327 Descend
327 Descend
327 Ascend
327 Ascend
328 Ascend
328 Ascend
328 Descend
329 Descend
329 Ascend
329 Ascend
329 Ascend
330 Ascend
330 Ascend
330 Descend
330 Descend
331 Ascend
331 Ascend
331 Ascend
332 Descend
332 Descend
332 Descend
333 Descend
333 Descend
333 Descend

334 Ascend
334 Ascend
334 Ascend
334 Ascend
335 Ascend
335 Ascend
335 Ascend
335 Ascend
336 Ascend
336 Ascend
336 Ascend
337 Ascend
337 Ascend
337 Ascend
337 Descend
338 Descend
338 Descend
338 Ascend
338 Ascend
339 Ascend
339 Descend
340 Descend
340 Descend
341 Descend
341 Ascend
341 Ascend
342 Ascend
342 Descend
342 Descend
343 Descend
343 Descend
343 Descend
343 Descend
344 Descend
344 Descend
344 Descend
345 Descend
345 Descend
346 Ascend
346 Ascend
346 Ascend
347 Ascend

347	Ascend
347	Ascend
347	Ascend
348	Ascend
348	Descend
348	Descend
349	Descend
349	Descend
349	Descend
350	Ascend
350	Ascend
350	Ascend
351	Ascend
351	Ascend
351	Descend
351	Descend
352	Descend
352	Descend
353	Ascend
353	Ascend
353	Ascend
354	Ascend
354	Ascend
354	Ascend
356	Descend
356	Descend
356	Ascend
356	Ascend
357	Ascend
358	Ascend
358	Ascend
358	Ascend
361	Descend
361	Ascend
361	Descend
361	Ascend
361	Descend
362	Ascend
362	Descend
362	Ascend
363	Descend
363	Ascend

363	Descend
363	Ascend
364	Descend
364	Ascend
364	Descend
365	Ascend
365	Ascend
366	Descend
366	Descend
367	Descend
367	Descend
367	Ascend
367	Ascend
368	Ascend
368	Descend
368	Descend
368	Descend
369	Descend
369	Ascend
369	Ascend
370	Ascend
370	Ascend
371	Descend
371	Descend
371	Descend
372	Descend
372	Descend
372	Descend
372	Descend
374	Descend
374	Descend
374	Descend
374	Descend
375	Ascend
376	Ascend
376	Descend
376	Descend
376	Descend
377	Descend
377	Descend
377	Descend
378	Ascend

378	Ascend
379	Ascend
379	Ascend
380	Ascend
380	Ascend
380	Ascend
380	Ascend
381	Descend
381	Descend
381	Descend
381	Descend
382	Descend
382	Descend
383	Ascend
383	Ascend
383	Ascend
383	Ascend
384	Ascend
384	Ascend
384	Ascend
385	Ascend
385	Ascend
386	Ascend
386	Ascend
386	Ascend
387	Ascend
387	Ascend
387	Ascend
388	Ascend
388	Ascend
389	Descend
389	Descend
389	Descend
389	Ascend
390	Ascend
390	Ascend
390	Ascend
391	Descend
391	Descend
392	Descend
393	Descend
394	Descend

396	Descend
397	Descend
397	Descend
397	Descend
398	Descend
398	Descend
398	Descend
399	Ascend
399	Ascend
400	Ascend
400	Ascend
400	Ascend
400	Ascend
401	Ascend
401	Ascend
401	Ascend
402	Ascend
402	Ascend
402	Descend
402	Descend
403	Descend
403	Descend
404	Descend
406	Ascend
406	Ascend
407	Ascend
407	Ascend
407	Ascend
407	Ascend
408	Ascend
408	Ascend
408	Ascend
408	Descend
409	Descend
409	Ascend
409	Ascend
409	Ascend
410	Descend
410	Descend
410	Ascend
411	Ascend
411	Ascend

411 Ascend
412 Ascend
413 Descend
413 Descend
413 Descend
414 Descend
415 Ascend
415 Ascend
416 Descend
416 Descend
417 Ascend
417 Ascend
419 Ascend
419 Ascend
419 Descend
419 Descend
420 Ascend
420 Ascend
421 Descend
421 Descend
423 Ascend
425 Ascend
425 Ascend
425 Ascend
426 Ascend
426 Descend
427 Descend
427 Descend
428 Ascend
428 Ascend
432 Ascend
432 Ascend
433 Ascend
434 Ascend
434 Ascend
434 Ascend
434 Ascend
435 Ascend
436 Ascend
436 Ascend
436 Ascend
437 Ascend

438	Ascend
439	Ascend
439	Ascend
440	Ascend
440	Ascend
442	Descend
443	Descend
443	Descend
444	Ascend
444	Ascend
444	Ascend
445	Ascend
445	Ascend
445	Ascend
445	Ascend
445	Ascend
445	Ascend
446	Ascend
446	Ascend
446	Ascend
447	Ascend
447	Ascend
447	Ascend
448	Ascend
448	Ascend
448	Ascend
448	Ascend
449	Ascend
449	Descend
450	Descend
450	Descend
451	Descend
451	Ascend
452	Ascend
452	Ascend
452	Ascend
452	Ascend
453	Ascend
453	Ascend
454	Ascend
454	Ascend
454	Ascend

454	Ascend
455	Ascend
456	Ascend
456	Ascend
457	Descend
457	Descend
457	Descend
457	Descend
458	Ascend
458	Ascend
458	Ascend
458	Ascend
459	Ascend
459	Ascend
459	Ascend
459	Ascend
459	Ascend
460	Ascend
460	Ascend
460	Ascend
461	Ascend
461	Ascend
461	Ascend
461	Ascend
462	Ascend
462	Ascend
462	Ascend
462	Ascend
463	Ascend
463	Ascend
463	Ascend
463	Ascend
464	Ascend
464	Ascend
464	Ascend
465	Ascend
465	Ascend
466	Descend
466	Descend
467	Ascend
467	Ascend
469	Descend

470	Descend
472	Descend
472	Descend
473	Ascend
473	Ascend
473	Ascend
473	Ascend
474	Ascend
474	Ascend
476	Descend
476	Descend
477	Ascend
477	Ascend
478	Ascend
478	Ascend
479	Ascend
480	Descend
481	Ascend
481	Ascend
482	Ascend
482	Ascend
482	Ascend
483	Ascend
483	Ascend
485	Ascend
485	Ascend
486	Ascend
486	Ascend
486	Ascend
487	Ascend
487	Ascend
488	Ascend
488	Ascend
489	Ascend
489	Ascend
490	Descend
490	Ascend
490	Ascend
490	Ascend
491	Descend
491	Descend
492	Ascend

492	Descend
492	Descend
493	Ascend
493	Ascend
494	Ascend
495	Ascend
495	Descend
495	Descend
498	Ascend
498	Ascend
499	Ascend
499	Ascend
501	Ascend
501	Ascend
501	Ascend
501	Ascend
503	Ascend
503	Ascend
504	Ascend
505	Ascend
506	Ascend
506	Ascend
506	Ascend
506	Ascend
507	Ascend
507	Ascend
509	Ascend
509	Ascend
509	Ascend
510	Ascend
510	Ascend
511	Ascend
511	Ascend
513	Ascend
513	Ascend
513	Ascend
514	Ascend
514	Ascend
514	Ascend
515	Ascend
515	Ascend
516	Ascend

516	Ascend
516	Ascend
517	Ascend
517	Ascend
518	Ascend
518	Ascend
518	Ascend
519	Ascend
519	Ascend
519	Ascend
520	Ascend
520	Ascend
520	Ascend
521	Ascend
521	Ascend
523	Ascend
523	Ascend
525	Ascend
525	Ascend
525	Ascend
526	Ascend
526	Ascend
526	Ascend
526	Ascend
527	Ascend
527	Ascend
528	Ascend
528	Ascend
529	Ascend
529	Ascend
530	Ascend
531	Ascend
532	Descend
532	Descend
532	Descend
534	Ascend
534	Ascend
534	Ascend
534	Ascend
535	Ascend
536	Ascend
537	Ascend

537	Ascend
539	Descend
539	Descend
540	Descend
540	Descend
541	Descend
542	Descend
542	Descend
544	Descend
544	Descend
544	Descend
546	Descend
546	Descend
547	Descend
548	Descend
548	Descend
548	Descend
549	Descend
549	Descend
550	Descend
550	Descend
550	Descend
551	Descend
551	Descend
552	Descend
552	Descend
552	Descend
553	Descend
553	Descend
554	Descend
554	Descend
554	Descend
555	Descend
555	Descend
555	Descend
556	Descend
556	Descend
556	Descend
557	Descend
557	Descend
557	Descend
558	Descend

558	Descend
558	Descend
559	Descend
559	Descend
560	Descend
560	Descend
560	Descend
560	Descend
561	Descend
561	Descend
561	Descend
562	Descend
563	Descend
564	Descend
565	Descend
565	Descend
566	Descend
567	Descend
568	Descend
568	Descend
570	Descend
570	Descend
571	Descend
571	Descend
572	Descend
572	Descend
574	Descend
574	Descend
576	Descend
576	Descend
577	Descend
577	Descend
579	Ascend
580	Descend
583	Ascend
583	Descend
585	Ascend
585	Descend
589	Ascend
590	Descend
591	Descend
592	Descend

595	Descend
596	Ascend
598	Descend
599	Ascend
600	Descend
601	Ascend
602	Descend
604	Ascend
605	Descend
608	Descend
612	Descend
615	Ascend
615	Descend
620	Ascend
620	Descend
622	Descend
626	Descend
626	Descend
628	Descend
630	Descend
633	Ascend
633	Descend
638	Ascend
639	Descend
647	Descend
651	Descend
654	Ascend
655	Ascend
659	Descend
698	Ascend
700	Ascend
700	Ascend
701	Ascend
701	Ascend
701	Descend
701	Descend
704	Ascend
704	Ascend
704	Ascend
705	Ascend
705	Ascend
705	Ascend

706	Ascend
707	Descend
708	Descend
710	Descend
710	Descend
712	Descend
713	Descend
714	Descend
715	Descend
715	Descend
716	Descend
718	Descend
719	Ascend
719	Ascend
719	Ascend
720	Descend
721	Descend
721	Descend
721	Descend
721	Descend
722	Descend
723	Descend
723	Descend
724	Descend
724	Ascend
725	Ascend
726	Descend
727	Descend
729	Ascend
731	Ascend
731	Ascend
731	Ascend
731	Ascend
732	Ascend
732	Ascend
732	Ascend
732	Descend
732	Descend
733	Ascend
733	Ascend
733	Ascend
734	Descend

734	Descend
734	Ascend
734	Ascend
734	Ascend
735	Ascend
735	Ascend
735	Ascend
736	Descend
736	Descend
736	Ascend
736	Ascend
737	Ascend
737	Ascend
737	Descend
738	Descend
738	Ascend
738	Ascend
738	Ascend
739	Ascend
740	Ascend
740	Ascend
740	Ascend
741	Descend
741	Descend
742	Descend
742	Descend
742	Ascend
743	Descend
743	Descend
744	Ascend
744	Ascend
745	Descend
746	Descend
747	Ascend
747	Ascend
747	Ascend
748	Ascend
748	Ascend
748	Ascend
748	Ascend
749	Ascend
749	Ascend

750	Ascend
750	Ascend
751	Ascend
751	Ascend
752	Ascend
752	Ascend
753	Ascend
754	Ascend
754	Ascend
754	Ascend
755	Ascend
755	Ascend
756	Ascend
756	Ascend
756	Ascend
756	Ascend
757	Ascend
758	Descend
758	Descend
758	Ascend
758	Ascend
759	Ascend
759	Ascend
760	Ascend
760	Ascend
761	Descend
761	Descend
762	Descend
762	Ascend
763	Ascend
763	Ascend
763	Ascend
764	Ascend
764	Ascend
766	Ascend
766	Ascend
767	Ascend
767	Ascend
769	Ascend
769	Ascend
771	Descend
771	Descend

772	Descend
772	Descend
774	Descend
774	Descend
775	Descend
775	Descend
776	Ascend
776	Ascend
777	Ascend
777	Ascend
779	Ascend
779	Ascend
779	Ascend
780	Ascend
781	Ascend
781	Ascend
782	Ascend
782	Ascend
782	Ascend
783	Ascend
783	Ascend
783	Ascend
784	Ascend
784	Ascend
785	Ascend
785	Ascend
785	Ascend
786	Ascend
786	Ascend
786	Ascend
786	Ascend
787	Ascend
787	Ascend
787	Ascend
787	Ascend
787	Ascend
788	Ascend
788	Ascend
788	Ascend
788	Ascend
789	Ascend
789	Ascend

789	Ascend
790	Ascend
790	Ascend
790	Ascend
791	Ascend
791	Ascend
791	Ascend
792	Ascend
792	Ascend
792	Ascend
792	Ascend
793	Ascend
793	Ascend
793	Ascend
793	Ascend
794	Ascend
794	Ascend
796	Ascend
796	Descend
796	Descend
796	Descend
796	Descend
797	Ascend
797	Ascend
797	Ascend
798	Ascend
799	Ascend
799	Descend
799	Descend
799	Descend
799	Descend
800	Descend
800	Descend
800	Ascend
801	Ascend
801	Ascend
801	Ascend
802	Ascend
802	Ascend
803	Ascend
803	Ascend
803	Ascend

804	Ascend
804	Ascend
804	Ascend
805	Ascend
805	Ascend
805	Ascend
805	Ascend
806	Ascend
806	Ascend
806	Ascend
807	Ascend
807	Ascend
807	Ascend
808	Ascend
808	Ascend
810	Ascend
810	Ascend
811	Ascend
812	Ascend
813	Ascend
813	Ascend
814	Ascend
815	Ascend
816	Ascend
817	Ascend
820	Ascend
820	Ascend
822	Descend
822	Descend
822	Descend
822	Descend
823	Descend
823	Descend
823	Ascend
823	Ascend
824	Descend
825	Descend
825	Descend
825	Ascend
825	Ascend
826	Ascend
826	Ascend

826 Ascend
827 Ascend
828 Ascend
828 Ascend
828 Ascend
829 Descend
829 Descend
829 Descend
830 Ascend
830 Ascend
831 Ascend
831 Ascend
832 Ascend
832 Ascend
836 Descend
836 Descend
836 Descend
836 Descend
837 Ascend
837 Ascend
837 Ascend
837 Ascend
837 Ascend
838 Ascend
838 Ascend
838 Ascend
839 Ascend
839 Ascend
840 Ascend
840 Ascend
840 Ascend
840 Ascend
841 Ascend
841 Descend
841 Descend
842 Descend
842 Descend
842 Descend
843 Ascend
843 Ascend
843 Ascend
844 Ascend

844	Ascend
844	Ascend
845	Descend
845	Descend
845	Ascend
845	Ascend
847	Ascend
847	Ascend
847	Ascend
848	Ascend
848	Ascend
848	Ascend
849	Ascend
849	Descend
850	Descend
850	Descend
851	Descend
851	Ascend
852	Descend
852	Descend
852	Descend
852	Descend
852	Descend
853	Descend
853	Descend
853	Descend
854	Descend
854	Descend
855	Ascend
855	Ascend
856	Descend
856	Descend
856	Descend
857	Descend
857	Descend
857	Descend
858	Descend
858	Descend
859	Descend
859	Descend
859	Descend
860	Descend

860	Descend
861	Descend
861	Descend
862	Descend
862	Ascend
863	Ascend
863	Descend
865	Ascend
867	Ascend
869	Ascend
869	Descend
870	Descend
871	Ascend
871	Ascend
872	Ascend
872	Ascend
872	Ascend
873	Ascend
873	Ascend
873	Ascend
875	Ascend
875	Ascend
876	Ascend
877	Descend
877	Descend
878	Descend
878	Descend
879	Descend
879	Descend
880	Descend
880	Descend
880	Descend
881	Descend
881	Descend
882	Descend
882	Descend
882	Descend
883	Ascend
883	Ascend
883	Ascend
884	Ascend
884	Ascend

884 Ascend
885 Ascend
885 Ascend
885 Ascend
885 Ascend
886 Ascend
887 Ascend
888 Ascend
889 Descend
890 Descend
892 Ascend
893 Ascend
894 Descend
895 Descend
896 Ascend
897 Descend
897 Descend
898 Descend
898 Descend
900 Descend
900 Ascend
900 Ascend
903 Ascend
903 Ascend
904 Ascend
904 Ascend
904 Ascend
904 Ascend
905 Ascend
905 Descend
905 Descend
906 Descend
907 Descend
907 Descend
908 Descend
908 Ascend
908 Ascend
909 Descend
909 Ascend
909 Ascend
910 Ascend
910 Ascend

910	Ascend
911	Ascend
912	Ascend
912	Ascend
913	Ascend
913	Descend
913	Ascend
914	Descend
914	Descend
914	Ascend
914	Ascend
915	Ascend
915	Ascend
915	Ascend
915	Ascend
916	Descend
916	Descend
916	Descend
917	Descend
917	Descend
917	Descend
918	Descend
919	Descend
919	Descend
919	Ascend
919	Ascend
920	Ascend
920	Ascend
920	Descend
921	Descend
921	Ascend
922	Ascend
922	Ascend
922	Ascend
922	Ascend
923	Ascend
923	Ascend
924	Ascend
924	Ascend
924	Ascend
925	Ascend
925	Ascend

926	Ascend
926	Ascend
926	Descend
927	Descend
927	Descend
927	Ascend
928	Ascend
928	Ascend
928	Ascend
929	Ascend
930	Ascend
930	Descend
930	Descend
931	Descend
931	Descend
931	Ascend
933	Descend
934	Ascend
936	Descend
937	Ascend
937	Ascend
939	Ascend
939	Ascend
940	Ascend
941	Ascend
942	Ascend
942	Ascend
943	Ascend
944	Ascend
944	Ascend
945	Ascend
945	Ascend
945	Ascend
945	Ascend
946	Ascend
946	Ascend
946	Ascend
946	Ascend
946	Ascend
947	Ascend
947	Ascend
947	Ascend

947	Ascend
948	Ascend
948	Ascend
949	Ascend
949	Ascend
949	Descend
949	Descend
949	Descend
950	Ascend
950	Ascend
951	Ascend
951	Ascend
951	Ascend
951	Ascend
951	Ascend
952	Ascend
952	Ascend
952	Ascend
952	Ascend
952	Ascend
953	Ascend
953	Ascend
953	Ascend
953	Ascend
954	Ascend
954	Ascend
954	Ascend
954	Ascend
955	Ascend
955	Ascend
956	Ascend
956	Ascend
957	Ascend
957	Ascend
958	Ascend
958	Ascend
958	Ascend
958	Ascend
959	Ascend
959	Descend
959	Descend
960	Descend

961	Descend
961	Descend
961	Descend
961	Descend
962	Descend
963	Descend
963	Descend
964	Descend
964	Descend
966	Ascend
966	Ascend
967	Ascend
967	Ascend
967	Ascend
967	Ascend
968	Ascend
968	Ascend
968	Ascend
968	Ascend
969	Ascend
969	Ascend
969	Ascend
970	Ascend
970	Ascend
970	Ascend
970	Ascend
970	Ascend
971	Ascend
971	Ascend
971	Ascend
971	Ascend
971	Ascend
972	Ascend
972	Ascend
972	Ascend
973	Ascend
973	Ascend
973	Ascend
973	Ascend
973	Ascend
973	Ascend
974	Ascend

974 Ascend
974 Ascend
974 Ascend
974 Ascend
975 Ascend
975 Ascend
975 Ascend
975 Ascend
976 Ascend
976 Ascend
976 Ascend
976 Ascend
977 Ascend
977 Ascend
977 Ascend
978 Ascend
978 Ascend
978 Ascend
978 Ascend
979 Descend
979 Descend
979 Ascend
980 Ascend
980 Ascend
980 Ascend
980 Ascend
981 Ascend
981 Ascend
981 Ascend
981 Ascend
981 Ascend
982 Ascend
982 Ascend
982 Ascend
982 Ascend
983 Ascend
983 Ascend
983 Ascend
983 Ascend
983 Ascend
984 Ascend
984 Ascend

984	Ascend
984	Ascend
985	Ascend
985	Ascend
985	Ascend
986	Ascend
986	Ascend
986	Ascend
987	Ascend
988	Ascend
988	Ascend
988	Ascend
988	Ascend
989	Ascend
989	Ascend
989	Ascend
989	Ascend
990	Ascend
990	Ascend
990	Ascend
990	Ascend
991	Descend
991	Descend
991	Descend
991	Descend
992	Ascend
992	Ascend
992	Ascend
993	Ascend
993	Ascend
993	Ascend
993	Ascend
993	Ascend
994	Ascend
994	Ascend
994	Ascend
994	Ascend
995	Ascend
995	Ascend
995	Ascend
995	Ascend
996	Ascend

996	Ascend
996	Ascend
996	Ascend
997	Ascend
997	Ascend
997	Ascend
998	Ascend
998	Ascend
999	Descend
999	Descend
999	Descend
999	Descend
999	Descend
1000	Ascend
1000	Ascend
1000	Ascend
1000	Ascend
1000	Descend
1001	Descend
1001	Descend
1001	Descend
1001	Ascend
1002	Ascend
1002	Ascend
1002	Ascend
1002	Descend
1002	Descend
1003	Descend
1003	Descend
1003	Ascend
1003	Ascend
1004	Ascend
1004	Ascend
1004	Ascend
1004	Ascend
1004	Ascend
1004	Ascend
1005	Ascend
1005	Ascend
1005	Ascend
1006	Ascend
1006	Ascend

1006	Ascend
1007	Ascend
1007	Descend
1007	Descend
1008	Descend
1008	Descend
1008	Ascend
1009	Ascend
1009	Descend
1009	Descend
1009	Descend
1009	Descend
1010	Descend
1010	Descend
1010	Descend
1011	Ascend
1011	Descend
1011	Descend
1012	Descend
1012	Descend
1012	Descend
1013	Ascend
1013	Ascend
1013	Descend
1013	Descend
1014	Ascend
1014	Ascend
1014	Ascend
1014	Ascend
1015	Ascend
1015	Ascend
1016	Ascend
1016	Ascend
1016	Ascend
1016	Ascend
1017	Ascend
1017	Ascend
1017	Ascend
1017	Ascend
1018	Ascend
1018	Ascend
1018	Ascend

1018	Descend
1018	Descend
1019	Descend
1019	Descend
1021	Ascend
1022	Ascend
1022	Ascend
1022	Ascend
1022	Ascend
1023	Ascend
1024	Descend
1024	Descend
1025	Ascend
1025	Ascend
1026	Ascend
1026	Ascend
1026	Ascend
1027	Ascend
1027	Ascend
1027	Descend
1027	Descend
1028	Descend
1028	Descend
1028	Descend
1028	Descend
1028	Descend
1029	Descend
1029	Descend
1029	Descend
1030	Descend
1030	Descend
1031	Descend
1031	Descend
1032	Descend
1032	Descend
1032	Ascend
1032	Ascend
1033	Descend
1033	Descend
1033	Descend
1033	Descend
1034	Descend

1034	Descend
1034	Ascend
1034	Ascend
1035	Ascend
1035	Ascend
1035	Ascend
1036	Ascend
1036	Ascend
1036	Ascend
1036	Ascend
1036	Ascend
1037	Ascend
1037	Ascend
1037	Ascend
1038	Ascend
1038	Ascend
1039	Ascend
1039	Descend
1039	Descend
1039	Descend
1040	Descend
1040	Descend
1040	Descend
1041	Descend
1041	Descend
1042	Descend
1042	Descend
1042	Descend
1043	Descend
1043	Descend
1044	Ascend
1044	Ascend
1046	Descend
1046	Descend
1048	Ascend
1048	Ascend
1048	Ascend
1048	Ascend
1049	Ascend
1049	Ascend
1050	Ascend
1050	Ascend

1050	Ascend
1050	Ascend
1052	Ascend
1053	Descend
1055	Ascend
1055	Ascend
1055	Ascend
1055	Ascend
1056	Ascend
1056	Ascend
1056	Ascend
1056	Ascend
1059	Descend
1059	Descend
1060	Ascend
1060	Ascend
1060	Ascend
1061	Ascend
1061	Ascend
1063	Descend
1063	Descend
1063	Descend
1064	Descend
1065	Ascend
1065	Ascend
1066	Ascend
1066	Ascend
1067	Ascend
1068	Ascend
1068	Ascend
1068	Ascend
1069	Ascend
1069	Ascend
1070	Ascend
1070	Ascend
1071	Ascend
1071	Ascend
1071	Ascend
1072	Ascend
1073	Ascend
1073	Ascend
1073	Ascend

1073	Ascend
1074	Ascend
1074	Ascend
1074	Ascend
1074	Ascend
1075	Ascend
1075	Ascend
1075	Ascend
1075	Ascend
1078	Ascend
1079	Ascend
1081	Ascend
1081	Ascend
1081	Ascend
1083	Ascend
1084	Descend
1084	Descend
1084	Descend
1085	Descend
1085	Ascend
1086	Ascend
1086	Descend
1086	Descend
1087	Descend
1088	Descend
1088	Ascend
1089	Ascend
1089	Descend
1089	Descend
1090	Descend
1090	Descend
1090	Ascend
1090	Ascend
1091	Ascend
1091	Ascend
1092	Ascend
1092	Ascend
1094	Ascend
1094	Ascend
1095	Ascend
1095	Ascend
1095	Ascend

1096	Ascend
1097	Descend
1097	Descend
1097	Descend
1098	Descend
1098	Descend
1099	Descend
1099	Descend
1099	Descend
1099	Descend
1101	Ascend
1101	Ascend
1101	Ascend
1102	Ascend
1102	Ascend
1102	Ascend
1103	Descend
1103	Descend
1103	Descend
1104	Descend
1104	Descend
1104	Ascend
1105	Descend
1105	Descend
1105	Ascend
1106	Ascend
1107	Ascend
1107	Ascend
1108	Ascend
1109	Ascend
1110	Ascend
1111	Ascend
1113	Ascend
1113	Descend
1114	Descend
1115	Descend
1117	Descend
1118	Descend
1118	Ascend
1120	Ascend
1120	Ascend
1122	Descend

1122	Ascend
1123	Ascend
1123	Ascend
1125	Descend
1125	Ascend
1125	Ascend
1126	Descend
1128	Ascend
1128	Ascend
1128	Ascend
1129	Ascend
1129	Descend
1129	Descend
1131	Ascend
1131	Ascend
1132	Ascend
1132	Descend
1133	Descend
1133	Descend
1134	Ascend
1134	Ascend
1135	Ascend
1135	Ascend
1136	Ascend
1136	Ascend
1136	Ascend
1137	Ascend
1137	Ascend
1137	Ascend
1138	Ascend
1138	Ascend
1139	Ascend
1139	Ascend
1139	Ascend
1140	Descend
1140	Descend
1140	Descend
1141	Ascend
1141	Ascend
1141	Descend
1141	Descend
1142	Descend

1142	Descend
1143	Ascend
1143	Ascend
1143	Ascend
1143	Descend
1143	Descend
1144	Ascend
1144	Ascend
1145	Descend
1145	Descend
1146	Ascend
1147	Ascend
1147	Descend
1148	Ascend
1148	Ascend
1149	Descend
1149	Descend
1151	Ascend
1151	Ascend
1151	Descend
1152	Descend
1152	Descend
1152	Descend
1153	Descend
1153	Descend
1153	Descend
1154	Descend
1154	Descend
1155	Ascend
1155	Ascend
1155	Ascend
1156	Ascend
1156	Ascend
1156	Ascend
1157	Ascend
1157	Ascend
1158	Descend
1158	Descend
1158	Descend
1159	Ascend
1159	Ascend
1159	Ascend

1159	Ascend
1160	Ascend
1160	Ascend
1160	Ascend
1161	Ascend
1161	Ascend
1162	Ascend
1162	Ascend
1162	Descend
1163	Descend
1163	Ascend
1163	Ascend
1163	Ascend
1163	Ascend
1164	Ascend
1164	Ascend
1164	Ascend
1164	Ascend
1164	Ascend
1165	Ascend
1165	Ascend
1166	Ascend
1166	Ascend
1167	Ascend
1169	Ascend
1169	Ascend
1170	Ascend
1170	Ascend
1170	Ascend
1170	Ascend
1171	Ascend
1171	Ascend
1171	Ascend
1172	Ascend
1172	Ascend
1172	Ascend
1173	Ascend
1173	Ascend
1173	Ascend
1174	Ascend
1174	Descend
1174	Descend

1175	Descend
1175	Descend
1175	Descend
1175	Descend
1176	Descend
1176	Descend
1176	Descend
1177	Descend
1177	Ascend
1177	Ascend
1178	Ascend
1178	Ascend
1178	Ascend
1178	Ascend
1179	Ascend
1179	Ascend
1179	Ascend
1179	Ascend
1180	Ascend
1180	Descend
1181	Descend
1181	Descend
1181	Descend
1181	Ascend
1182	Ascend
1182	Ascend
1182	Ascend
1183	Ascend
1183	Ascend
1183	Ascend
1183	Ascend
1184	Ascend
1184	Ascend
1184	Ascend
1185	Ascend
1185	Ascend
1185	Ascend
1185	Ascend
1185	Ascend
1185	Ascend
1186	Ascend
1186	Ascend

1186	Ascend
1187	Ascend
1187	Ascend
1187	Ascend
1187	Ascend
1187	Ascend
1188	Descend
1188	Descend
1188	Descend
1188	Descend
1189	Descend
1189	Descend
1189	Descend
1190	Descend
1190	Descend
1190	Descend
1190	Ascend
1190	Ascend
1191	Ascend
1191	Ascend
1191	Ascend
1191	Descend
1192	Descend
1192	Descend
1192	Ascend
1192	Ascend
1192	Descend
1193	Ascend
1193	Ascend
1193	Ascend
1193	Ascend
1193	Ascend
1194	Ascend
1194	Ascend
1194	Descend
1194	Ascend
1195	Descend
1195	Descend
1195	Descend
1195	Descend
1196	Descend
1196	Ascend

1196	Ascend
1196	Ascend
1196	Ascend
1197	Descend
1197	Descend
1197	Ascend
1197	Ascend
1198	Ascend
1198	Ascend
1199	Descend
1199	Descend
9	AntIn
11	AntIn
11	AntIn
12	AntIn
12	AntOut
12	AntOut
13	AntOut
16	AntIn
19	AntIn
20	AntOut
22	AntIn
22	AntIn
24	AntOut
25	AntIn
27	AntOut
28	AntIn
30	AntIn
31	AntIn
31	AntOut
31	AntOut
33	AntOut
34	AntIn
35	AntIn
35	AntOut
35	AntOut
36	AntIn
37	AntIn
37	AntIn
38	AntOut
43	AntOut
44	AntOut

48	AntIn
49	AntIn
50	AntOut
51	AntOut
51	AntOut
51	AntOut
52	AntIn
53	AntIn
53	AntIn
54	AntIn
55	AntOut
55	AntIn
56	AntIn
57	AntIn
57	AntIn
58	AntOut
59	AntOut
59	AntIn
60	AntIn
61	AntOut
62	AntOut
64	AntIn
66	AntOut
66	AntOut
67	AntIn
68	AntIn
68	AntIn
68	AntIn
68	AntIn
69	AntIn
69	AntIn
72	AntOut
73	AntIn
73	AntIn
73	AntOut
75	AntOut
76	AntOut
77	AntIn
77	AntOut
79	AntOut
79	AntOut
80	AntIn

81	AntIn
82	AntOut
83	AntIn
83	AntIn
84	AntOut
84	AntOut
85	AntIn
86	AntOut
86	AntOut
88	AntOut
89	AntIn
91	AntOut
91	AntIn
92	AntIn
92	AntIn
92	AntIn
93	AntIn
94	AntOut
95	AntOut
96	AntOut
97	AntOut
98	AntIn
98	AntIn
99	AntIn
99	AntIn
100	AntIn
101	AntIn
102	AntIn
104	AntIn
106	AntIn
107	AntIn
108	AntOut
108	AntOut
111	AntOut
112	AntIn
113	AntOut
114	AntOut
114	AntOut
115	AntOut
115	AntOut
116	AntOut
117	AntOut

117 AntOut
117 AntIn
118 AntOut
120 AntOut
120 AntOut
122 AntIn
123 AntOut
124 AntOut
124 AntOut
124 AntOut
125 AntIn
126 AntOut
127 AntOut
127 AntOut
128 AntIn
129 AntIn
130 AntOut
131 AntIn
131 AntIn
131 AntIn
134 AntOut
134 AntIn
134 AntIn
135 AntOut
136 AntOut
136 AntOut
138 AntIn
139 AntIn
140 AntIn
141 AntOut
142 AntIn
142 AntIn
142 AntIn
143 AntIn
144 AntOut
147 AntIn
150 AntOut
152 AntOut
152 AntIn
153 AntOut
154 AntOut
154 AntOut

155 AntOut
155 AntIn
155 AntIn
156 AntOut
156 AntOut
156 AntOut
157 AntOut
157 AntOut
158 AntIn
160 AntOut
160 AntIn
163 AntOut
164 AntOut
164 AntOut
165 AntOut
165 AntIn
166 AntOut
167 AntIn
168 AntIn
168 AntIn
169 AntOut
169 AntOut
170 AntOut
173 AntOut
173 AntIn
176 AntIn
176 AntIn
177 AntIn
177 AntOut
177 AntOut
179 AntIn
179 AntIn
179 AntIn
181 AntIn
182 AntOut
183 AntIn
184 AntIn
185 AntIn
185 AntIn
186 AntIn
186 AntOut
187 AntIn

189	AntIn
192	AntIn
192	AntOut
192	AntOut
193	AntOut
193	AntIn
193	AntIn
194	AntOut
196	AntIn
197	AntIn
197	AntOut
198	AntOut
198	AntOut
198	AntOut
199	AntIn
199	AntIn
200	AntOut
200	AntOut
200	AntOut
201	AntOut
201	AntOut
202	AntIn
203	AntIn
203	AntIn
205	AntOut
205	AntIn
205	AntIn
208	AntIn
209	AntOut
210	AntOut
210	AntOut
211	AntIn
211	AntIn
212	AntOut
214	AntIn
216	AntIn
216	AntOut
217	AntIn
218	AntOut
219	AntOut
219	AntOut
219	AntOut

220 AntIn
220 AntOut
220 AntOut
221 AntOut
221 AntOut
222 AntOut
223 AntIn
224 AntOut
225 AntIn
226 AntOut
226 AntOut
227 AntOut
227 AntOut
228 AntIn
230 AntIn
231 AntOut
233 AntIn
233 AntIn
234 AntIn
236 AntOut
237 AntIn
237 AntIn
237 AntIn
238 AntIn
238 AntIn
239 AntIn
239 AntOut
240 AntOut
241 AntOut
241 AntOut
241 AntIn
242 AntOut
243 AntIn
243 AntIn
244 AntIn
244 AntIn
245 AntIn
245 AntOut
246 AntIn
247 AntIn
248 AntOut
248 AntOut

249	AntIn
250	AntIn
250	AntIn
250	AntIn
251	AntIn
251	AntOut
253	AntOut
254	AntOut
254	AntOut
256	AntIn
257	AntOut
257	AntOut
258	AntIn
259	AntIn
260	AntIn
261	AntIn
262	AntIn
262	AntOut
262	AntOut
263	AntOut
265	AntOut
265	AntOut
266	AntIn
266	AntOut
267	AntOut
268	AntOut
268	AntIn
269	AntIn
270	AntIn
270	AntIn
271	AntOut
272	AntOut
272	AntOut
274	AntOut
274	AntOut
275	AntOut
275	AntOut
276	AntIn
277	AntIn
277	AntIn
278	AntIn
279	AntOut

280 AntOut
280 AntIn
280 AntOut
281 AntIn
281 AntIn
281 AntOut
282 AntIn
283 AntIn
283 AntOut
284 AntIn
284 AntIn
286 AntIn
286 AntOut
287 AntOut
287 AntOut
288 AntIn
289 AntOut
293 AntIn
293 AntOut
294 AntOut
294 AntOut
294 AntOut
295 AntOut
296 AntIn
296 AntIn
298 AntIn
298 AntIn
298 AntIn
299 AntIn
301 AntIn
302 AntOut
303 AntOut
307 AntIn
308 AntIn
308 AntOut
308 AntOut
310 AntIn
311 AntOut
312 AntOut
312 AntOut
314 AntIn
316 AntIn

317	AntIn
317	AntIn
317	AntIn
318	AntIn
318	AntIn
319	AntIn
319	AntIn
319	AntOut
322	AntOut
323	AntIn
323	AntIn
327	AntOut
329	AntIn
329	AntIn
330	AntIn
332	AntOut
332	AntOut
333	AntIn
334	AntIn
335	AntIn
335	AntIn
335	AntIn
337	AntIn
338	AntOut
339	AntIn
339	AntOut
340	AntOut
340	AntOut
341	AntOut
341	AntOut
342	AntIn
343	AntIn
343	AntIn
344	AntIn
346	AntOut
347	AntOut
347	AntOut
347	AntOut
348	AntOut
348	AntOut
349	AntOut
351	AntIn

352 AntIn
352 AntOut
353 AntIn
353 AntIn
355 AntIn
355 AntIn
355 AntIn
356 AntOut
356 AntOut
356 AntOut
357 AntOut
358 AntIn
363 AntIn
363 AntIn
363 AntIn
363 AntIn
364 AntIn
365 AntIn
365 AntIn
368 AntIn
369 AntIn
370 AntIn
370 AntIn
374 AntIn
378 AntOut
378 AntIn
382 AntIn
384 AntIn
384 AntIn
386 AntIn
386 AntOut
388 AntOut
388 AntOut
388 AntOut
389 AntOut
389 AntOut
390 AntIn
390 AntOut
391 AntOut
392 AntIn
394 AntOut
397 AntOut

397	AntOut
398	AntOut
399	AntIn
400	AntIn
402	AntIn
403	AntOut
404	AntOut
405	AntOut
405	AntOut
406	AntOut
407	AntOut
408	AntIn
408	AntIn
410	AntOut
410	AntIn
411	AntOut
412	AntIn
414	AntOut
415	AntOut
415	AntIn
416	AntIn
419	AntOut
420	AntOut
421	AntOut
426	AntIn
432	AntIn
433	AntOut
435	AntIn
441	AntIn
442	AntIn
443	AntOut
444	AntOut
445	AntIn
445	AntIn
446	AntOut
447	AntOut
449	AntOut
451	AntIn
452	AntOut
452	AntOut
454	AntIn
459	AntOut

460	AntOut
461	AntOut
464	AntIn
465	AntIn
467	AntOut
467	AntOut
473	AntOut
473	AntOut
474	AntOut
474	AntOut
481	AntOut
483	AntIn
487	AntIn
489	AntIn
490	AntOut
492	AntOut
492	AntIn
492	AntIn
494	AntOut
495	AntOut
495	AntOut
496	AntIn
497	AntIn
498	AntOut
498	AntOut
500	AntIn
501	AntIn
501	AntIn
502	AntOut
504	AntOut
507	AntOut
508	AntOut
509	AntOut
511	AntOut
511	AntIn
511	AntIn
514	AntOut
514	AntIn
515	AntOut
515	AntOut
516	AntIn
518	AntIn

523	AntOut
524	AntOut
524	AntIn
525	AntIn
526	AntOut
526	AntIn
528	AntOut
529	AntIn
533	AntIn
533	AntIn
533	AntIn
534	AntIn
536	AntIn
536	AntIn
540	AntOut
541	AntOut
542	AntIn
542	AntIn
543	AntIn
544	AntIn
545	AntIn
546	AntIn
546	AntIn
546	AntIn
547	AntOut
548	AntIn
549	AntOut
553	AntOut
554	AntOut
561	AntOut
561	AntIn
562	AntIn
565	AntIn
570	AntOut
571	AntIn
572	AntOut
573	AntIn
576	AntIn
578	AntOut
587	AntOut
590	AntOut
590	AntIn

590	AntIn
591	AntIn
592	AntIn
596	AntOut
606	AntIn
608	AntOut
610	AntIn
615	AntOut
615	AntIn
618	AntIn
623	AntIn
628	AntIn
640	AntIn
669	AntIn
669	AntIn
671	AntOut
672	AntIn
681	AntOut
683	AntIn
686	AntIn
687	AntIn
688	AntIn
689	AntIn
697	AntIn
700	AntIn
702	AntIn
702	AntIn
703	AntOut
704	AntOut
705	AntOut
705	AntIn
706	AntIn
710	AntIn
711	AntIn
712	AntIn
715	AntIn
719	AntIn
721	AntIn
721	AntOut
721	AntOut
722	AntIn
724	AntIn

725 AntOut
726 AntOut
727 AntIn
729 AntIn
730 AntIn
731 AntIn
731 AntOut
733 AntIn
735 AntIn
735 AntIn
735 AntIn
736 AntIn
739 AntIn
741 AntIn
741 AntIn
742 AntIn
743 AntIn
744 AntOut
746 AntIn
747 AntOut
748 AntIn
750 AntIn
750 AntIn
752 AntOut
754 AntOut
755 AntOut
755 AntOut
756 AntOut
756 AntIn
760 AntIn
760 AntIn
761 AntIn
763 AntOut
766 AntOut
768 AntOut
769 AntIn
769 AntIn
770 AntIn
772 AntIn
774 AntIn
774 AntIn
775 AntIn

776 AntOut
777 AntIn
778 AntIn
778 AntIn
780 AntIn
781 AntOut
783 AntIn
783 AntIn
784 AntOut
784 AntIn
785 AntIn
790 AntOut
790 AntOut
792 AntIn
794 AntIn
796 AntIn
797 AntOut
797 AntOut
798 AntIn
800 AntIn
802 AntIn
808 AntIn
814 AntIn
816 AntOut
817 AntOut
817 AntIn
821 AntIn
821 AntOut
823 AntOut
824 AntOut
826 AntIn
828 AntIn
829 AntIn
829 AntIn
831 AntIn
832 AntIn
837 AntIn
837 AntIn
839 AntOut
841 AntIn
842 AntOut
844 AntIn

844 AntIn
844 AntIn
845 AntOut
846 AntIn
847 AntIn
848 AntOut
849 AntIn
850 AntIn
850 AntIn
851 AntIn
852 AntIn
853 AntIn
853 AntIn
853 AntIn
855 AntIn
856 AntIn
857 AntIn
860 AntOut
861 AntOut
862 AntIn
864 AntIn
864 AntIn
867 AntIn
867 AntIn
867 AntIn
868 AntOut
872 AntOut
873 AntIn
874 AntIn
878 AntIn
882 AntIn
886 AntIn
886 AntIn
889 AntIn
891 AntIn
892 AntIn
893 AntOut
893 AntIn
894 AntIn
895 AntIn
896 AntIn
897 AntOut

897 AntOut
900 AntIn
901 AntIn
901 AntIn
902 AntOut
904 AntIn
907 AntOut
911 AntOut
916 AntOut
916 AntIn
918 AntIn
919 AntIn
920 AntIn
921 AntIn
924 AntOut
925 AntOut
926 AntOut
928 AntIn
930 AntIn
931 AntIn
932 AntIn
936 AntIn
941 AntIn
945 AntIn
949 AntIn
949 AntIn
950 AntIn
952 AntOut
953 AntIn
955 AntIn
958 AntIn
959 AntOut
959 AntIn
962 AntOut
962 AntIn
964 AntIn
966 AntIn
967 AntOut
967 AntIn
969 AntIn
970 AntIn
970 AntIn

971	AntIn
971	AntIn
972	AntIn
973	AntIn
974	AntOut
976	AntOut
977	AntOut
977	AntIn
978	AntIn
978	AntOut
979	AntIn
981	AntOut
981	AntIn
981	AntIn
982	AntIn
983	AntIn
983	AntOut
990	AntOut
990	AntIn
990	AntIn
991	AntOut
992	AntOut
992	AntOut
993	AntOut
994	AntIn
995	AntIn
996	AntIn
997	AntIn
997	AntIn
997	AntIn
998	AntIn
998	AntIn
999	AntOut
999	AntOut
1001	AntOut
1001	AntOut
1005	AntIn
1006	AntIn
1006	AntIn
1007	AntIn
1007	AntOut
1008	AntIn

1009 AntOut
1009 AntOut
1010 AntOut
1014 AntOut
1015 AntOut
1015 AntIn
1016 AntIn
1017 AntOut
1018 AntOut
1019 AntOut
1022 AntIn
1023 AntIn
1023 AntOut
1025 AntOut
1026 AntOut
1027 AntOut
1028 AntOut
1030 AntIn
1030 AntOut
1031 AntOut
1033 AntIn
1035 AntOut
1035 AntIn
1040 AntOut
1041 AntOut
1042 AntOut
1042 AntIn
1042 AntIn
1043 AntIn
1045 AntIn
1046 AntIn
1048 AntIn
1049 AntIn
1051 AntIn
1052 AntIn
1054 AntOut
1059 AntIn
1059 AntIn
1060 AntIn
1060 AntIn
1061 AntOut
1063 AntOut

1063 AntIn
1068 AntIn
1071 AntIn
1072 AntIn
1074 AntIn
1074 AntOut
1075 AntIn
1077 AntIn
1077 AntIn
1078 AntOut
1082 AntIn
1087 AntIn
1091 AntOut
1091 AntOut
1092 AntOut
1093 AntOut
1094 AntOut
1094 AntIn
1097 AntOut
1102 AntOut
1103 AntOut
1105 AntOut
1106 AntOut
1109 AntIn
1112 AntIn
1112 AntOut
1113 AntOut
1113 AntOut
1116 AntOut
1116 AntIn
1117 AntIn
1118 AntOut
1119 AntIn
1122 AntOut
1123 AntIn
1126 AntOut
1126 AntIn
1130 AntIn
1131 AntOut
1135 AntIn
1136 AntIn
1137 AntIn

1140 AntOut
1143 AntIn
1144 AntIn
1145 AntIn
1145 AntIn
1148 AntIn
1150 AntOut
1152 AntOut
1153 AntIn
1153 AntIn
1154 AntIn
1154 AntIn
1156 AntIn
1159 AntOut
1164 AntOut
1164 AntOut
1165 AntIn
1167 AntIn
1167 AntOut
1168 AntIn
1169 AntOut
1173 AntOut
1173 AntOut
1174 AntOut
1177 AntOut
1178 AntOut
1180 AntOut
1180 AntOut
1183 AntIn
1186 AntIn
1189 AntIn
1189 AntIn
1190 AntIn
1190 AntIn
1192 AntOut
1194 AntOut
1194 AntIn
1197 AntOut
1198 AntOut
1198 AntOut
1201 AntOut
1202 AntOut
