# Supplementary material for: Interactions Increase Forager Availability and Activity in Harvester Ants
Source: PLoS One. 2015 Nov 5;10(11):e0141971. doi: 10.1371/journal.pone.0141971 (PMC4635008; doi:10.1371/journal.pone.0141971)
Supplement: S3 Dataset — We observed and filmed behavior inside the nest during and after forager removals. This dataset shows our counts made from the films of the numbers of returning and outgoing foragers at the nest entrance and the number of ascending and descending ants at all tunnel entrances. (ZIP) [file pone.0141971.s004.zip › S3 Dataset/2013 Correlation Data 25 8-24.pdf]

**Researcher**	**Jovel Queirolo**
**Colony 25**	
**8/24/13**	
**Video time (seconds)**	**Event**
7	Descend
8	Descend
8	Descend
8	Descend
9	Descend
10	Descend
11	Descend
12	Ascend
12	Ascend
14	Ascend
14	Ascend
15	Ascend
16	Descend
16	Descend
18	Descend
20	Ascend
20	Ascend
23	Descend
24	Ascend
26	Ascend
29	Ascend
29	Ascend
29	Ascend
30	Ascend
30	Ascend
30	Ascend
31	Ascend
31	Ascend
32	Ascend
33	Ascend
33	Ascend
34	Ascend
35	Descend
35	Descend
36	Ascend
36	Descend
37	Descend

37	Descend
37	Descend
38	Descend
38	Descend
39	Ascend
40	Ascend
40	Ascend
41	Descend
41	Descend
42	Ascend
42	Ascend
43	Ascend
43	Descend
44	Descend
44	Descend
45	Descend
45	Descend
46	Descend
46	Descend
46	Descend
47	Descend
48	Descend
48	Ascend
48	Ascend
48	Ascend
49	Ascend
49	Ascend
49	Ascend
50	Ascend
50	Descend
50	Descend
51	Ascend
51	Ascend
54	Ascend
54	Ascend
59	Ascend
59	Ascend
60	Ascend
60	Descend
61	Descend
64	Descend
64	Descend

64	Descend
65	Ascend
66	Ascend
66	Ascend
68	Descend
69	Descend
70	Ascend
71	Ascend
73	Ascend
75	Ascend
78	Ascend
79	Descend
81	Descend
81	Ascend
82	Ascend
83	Ascend
88	Ascend
89	Descend
94	Descend
96	Descend
98	Ascend
101	Ascend
103	Descend
103	Descend
105	Descend
105	Descend
105	Ascend
106	Ascend
108	Descend
108	Ascend
108	Ascend
109	Ascend
109	Ascend
110	Ascend
110	Ascend
110	Ascend
110	Ascend
112	Ascend
114	Ascend
114	Ascend
116	Ascend
118	Ascend

118	Ascend
123	Descend
125	Ascend
126	Descend
127	Ascend
129	Ascend
130	Descend
130	Ascend
132	Ascend
132	Ascend
132	Ascend
135	Descend
137	Ascend
138	Ascend
138	Ascend
138	Ascend
138	Ascend
139	Ascend
141	Ascend
141	Ascend
141	Descend
141	Descend
142	Descend
142	Descend
143	Descend
143	Descend
144	Descend
144	Descend
145	Descend
145	Ascend
145	Ascend
147	Descend
147	Descend
148	Descend
148	Descend
148	Descend
149	Descend
149	Descend
150	Descend
150	Descend
152	Descend
153	Descend

154	Ascend
155	Descend
157	Ascend
157	Ascend
160	Descend
160	Descend
160	Descend
161	Descend
163	Descend
164	Descend
164	Descend
167	Ascend
167	Ascend
169	Ascend
169	Ascend
171	Ascend
176	Descend
176	Descend
177	Descend
178	Descend
180	Ascend
180	Ascend
185	Descend
187	Descend
187	Descend
187	Descend
188	Descend
188	Descend
190	Ascend
190	Ascend
191	Descend
191	Descend
191	Descend
191	Descend
195	Descend
196	Descend
196	Ascend
196	Ascend
197	Ascend
198	Ascend
199	Ascend
199	Ascend

199	Ascend
199	Ascend
200	Ascend
200	Ascend
200	Ascend
200	Ascend
201	Ascend
201	Ascend
201	Ascend
202	Ascend
202	Ascend
202	Ascend
202	Ascend
203	Ascend
203	Ascend
205	Ascend
206	Ascend
207	Ascend
207	Ascend
208	Ascend
208	Ascend
209	Ascend
210	Ascend
211	Descend
211	Descend
211	Descend
211	Descend
213	Descend
213	Descend
213	Descend
214	Descend
214	Descend
214	Descend
215	Ascend
215	Ascend
216	Ascend
217	Ascend
218	Ascend
218	Ascend
218	Ascend
219	Ascend
219	Ascend

220	Ascend
220	Ascend
220	Ascend
223	Descend
223	Descend
224	Descend
224	Ascend
224	Ascend
226	Descend
226	Descend
229	Ascend
229	Ascend
229	Ascend
230	Ascend
230	Ascend
233	Ascend
234	Descend
235	Descend
237	Descend
241	Descend
241	Ascend
243	Ascend
243	Ascend
245	Descend
247	Descend
247	Descend
248	Ascend
248	Ascend
249	Ascend
250	Ascend
253	Ascend
255	Ascend
255	Ascend
259	Ascend
263	Descend
263	Descend
267	Ascend
267	Ascend
269	Ascend
272	Descend
276	Ascend
279	Descend

280	Descend
281	Ascend
281	Descend
283	Ascend
283	Ascend
283	Ascend
284	Ascend
285	Ascend
285	Descend
286	Descend
286	Descend
287	Descend
289	Ascend
289	Ascend
292	Descend
293	Descend
294	Ascend
294	Ascend
296	Ascend
296	Ascend
298	Descend
300	Ascend
300	Ascend
301	Ascend
301	Descend
302	Descend
302	Descend
303	Descend
304	Ascend
304	Ascend
306	Ascend
307	Descend
309	Descend
309	Ascend
310	Ascend
311	Ascend
312	Ascend
313	Ascend
314	Ascend
315	Ascend
317	Ascend
317	Ascend

318	Ascend
318	Ascend
319	Ascend
320	Descend
320	Descend
323	Ascend
324	Ascend
324	Ascend
325	Ascend
325	Ascend
329	Ascend
329	Ascend
329	Ascend
330	Ascend
330	Ascend
331	Ascend
332	Ascend
332	Ascend
333	Ascend
333	Ascend
333	Ascend
333	Ascend
333	Ascend
334	Ascend
334	Ascend
334	Ascend
334	Ascend
335	Descend
336	Descend
336	Descend
337	Ascend
337	Ascend
339	Ascend
339	Ascend
340	Descend
341	Descend
343	Descend
347	Ascend
350	Ascend
350	Ascend
350	Ascend
350	Ascend

351	Ascend
351	Descend
351	Descend
352	Descend
352	Descend
353	Descend
354	Ascend
354	Ascend
354	Ascend
355	Ascend
355	Ascend
356	Ascend
356	Ascend
357	Ascend
357	Ascend
357	Ascend
357	Ascend
358	Ascend
358	Ascend
358	Ascend
358	Ascend
358	Ascend
359	Ascend
359	Ascend
359	Ascend
360	Ascend
360	Ascend
360	Ascend
360	Ascend
361	Ascend
361	Ascend
361	Ascend
362	Ascend
362	Ascend
363	Ascend
367	Ascend
367	Ascend
367	Ascend
367	Ascend
368	Ascend
371	Ascend
371	Ascend

371	Ascend
372	Ascend
372	Ascend
372	Ascend
372	Ascend
372	Descend
373	Descend
373	Descend
373	Descend
373	Ascend
374	Ascend
374	Ascend
374	Ascend
375	Descend
375	Descend
375	Ascend
375	Ascend
376	Descend
376	Descend
376	Ascend
376	Ascend
377	Ascend
377	Ascend
378	Ascend
378	Ascend
379	Ascend
379	Ascend
379	Ascend
379	Ascend
381	Ascend
381	Ascend
383	Ascend
383	Ascend
384	Ascend
384	Ascend
384	Ascend
385	Ascend
385	Ascend
386	Ascend
386	Ascend
386	Ascend
387	Ascend

387	Ascend
388	Ascend
388	Ascend
388	Ascend
388	Ascend
389	Ascend
389	Ascend
390	Ascend
390	Ascend
390	Ascend
391	Ascend
391	Ascend
391	Ascend
392	Ascend
392	Ascend
392	Ascend
393	Ascend
393	Ascend
393	Ascend
393	Ascend
394	Ascend
396	Ascend
396	Ascend
397	Ascend
398	Descend
398	Descend
400	Ascend
401	Ascend
401	Ascend
401	Ascend
402	Ascend
402	Ascend
402	Ascend
402	Ascend
403	Ascend
403	Ascend
404	Ascend
404	Ascend
405	Ascend
405	Ascend
406	Ascend
406	Ascend

406	Ascend
406	Ascend
407	Ascend
407	Ascend
407	Ascend
407	Ascend
408	Ascend
409	Ascend
411	Ascend
411	Ascend
412	Ascend
412	Ascend
413	Ascend
414	Descend
414	Descend
415	Descend
415	Ascend
416	Ascend
417	Ascend
418	Ascend
418	Descend
419	Descend
419	Descend
420	Descend
421	Ascend
422	Ascend
422	Ascend
423	Ascend
423	Ascend
423	Ascend
423	Ascend
424	Ascend
424	Ascend
425	Ascend
425	Ascend
426	Descend
426	Descend
427	Descend
427	Ascend
427	Ascend
428	Ascend
428	Ascend

429	Descend
429	Descend
429	Descend
430	Descend
431	Descend
431	Descend
432	Descend
432	Descend
433	Descend
434	Descend
434	Descend
435	Descend
436	Descend
437	Descend
437	Descend
438	Descend
438	Descend
438	Descend
439	Descend
440	Descend
440	Descend
442	Descend
442	Descend
443	Ascend
444	Ascend
445	Ascend
445	Ascend
446	Descend
446	Descend
447	Descend
447	Descend
447	Descend
450	Descend
450	Descend
451	Descend
452	Ascend
452	Ascend
453	Ascend
453	Ascend
453	Ascend
454	Ascend
455	Ascend

455	Ascend
456	Ascend
456	Ascend
456	Descend
456	Descend
457	Descend
457	Descend
459	Descend
459	Descend
460	Descend
460	Descend
461	Descend
461	Ascend
462	Ascend
462	Ascend
462	Ascend
462	Ascend
463	Ascend
463	Ascend
463	Ascend
464	Ascend
465	Ascend
465	Ascend
466	Descend
466	Descend
467	Ascend
467	Ascend
468	Ascend
468	Ascend
470	Ascend
470	Ascend
470	Ascend
470	Ascend
471	Ascend
471	Ascend
471	Ascend
472	Ascend
473	Ascend
473	Ascend
473	Ascend
473	Ascend
473	Ascend

474	Ascend
474	Ascend
474	Ascend
474	Ascend
475	Ascend
475	Ascend
476	Ascend
476	Ascend
476	Ascend
476	Ascend
477	Ascend
477	Ascend
477	Descend
478	Descend
478	Descend
478	Descend
480	Descend
480	Descend
480	Descend
482	Descend
482	Descend
484	Descend
484	Descend
486	Descend
486	Descend
487	Descend
488	Descend
489	Descend
490	Ascend
490	Ascend
492	Ascend
493	Ascend
494	Descend
494	Ascend
496	Descend
496	Descend
497	Descend
497	Ascend
498	Ascend
498	Ascend
499	Ascend
499	Ascend

499	Ascend
499	Ascend
500	Ascend
500	Ascend
500	Ascend
501	Ascend
501	Ascend
501	Ascend
502	Ascend
502	Ascend
502	Ascend
502	Ascend
503	Ascend
503	Ascend
503	Ascend
503	Ascend
504	Ascend
504	Ascend
505	Ascend
505	Ascend
505	Ascend
506	Ascend
506	Ascend
506	Ascend
506	Ascend
507	Ascend
507	Ascend
507	Ascend
507	Ascend
508	Ascend
508	Ascend
509	Ascend
509	Ascend
510	Descend
510	Descend
510	Descend
510	Descend
511	Descend
511	Descend
513	Descend
513	Descend
515	Descend

515	Descend
516	Descend
516	Descend
523	Ascend
523	Ascend
523	Ascend
523	Ascend
523	Ascend
523	Ascend
525	Ascend
525	Ascend
525	Ascend
525	Ascend
526	Ascend
526	Ascend
527	Ascend
528	Ascend
529	Ascend
529	Ascend
529	Ascend
529	Ascend
529	Ascend
530	Ascend
530	Ascend
532	Ascend
533	Ascend
534	Ascend
534	Ascend
535	Descend
535	Descend
535	Descend
535	Descend
536	Descend
537	Descend
538	Descend
539	Descend
539	Descend
541	Descend
541	Descend
545	Descend
545	Descend
551	Descend

553	Ascend
553	Ascend
554	Ascend
554	Ascend
554	Ascend
555	Ascend
555	Ascend
557	Ascend
557	Ascend
558	Ascend
558	Ascend
558	Ascend
558	Ascend
559	Ascend
559	Ascend
560	Descend
560	Descend
560	Ascend
561	Ascend
561	Ascend
561	Ascend
562	Ascend
562	Ascend
562	Ascend
563	Ascend
563	Ascend
564	Ascend
564	Ascend
566	Descend
566	Descend
566	Descend
567	Ascend
567	Ascend
568	Ascend
569	Ascend
569	Ascend
569	Ascend
569	Ascend
571	Descend
571	Descend
573	Ascend
575	Ascend

576	Ascend
577	Ascend
577	Ascend
578	Ascend
578	Ascend
579	Ascend
579	Ascend
579	Ascend
579	Ascend
579	Ascend
580	Ascend
580	Ascend
582	Ascend
582	Ascend
583	Descend
584	Descend
585	Descend
585	Descend
587	Descend
587	Descend
588	Descend
589	Descend
590	Descend
591	Descend
591	Ascend
592	Ascend
592	Ascend
593	Ascend
593	Ascend
595	Ascend
595	Ascend
596	Ascend
596	Ascend
598	Ascend
598	Ascend
599	Ascend
599	Ascend
600	Ascend
600	Ascend
601	Ascend
601	Ascend
602	Ascend

602 Ascend
603 Ascend
604 Ascend
605 Ascend
605 Ascend
606 Ascend
606 Ascend
607 Ascend
607 Ascend
608 Ascend
608 Ascend
610 Ascend
610 Ascend
611 Ascend
612 Ascend
613 Ascend
615 Descend
615 Descend
615 Descend
615 Descend
616 Descend
616 Descend
617 Ascend
618 Ascend
619 Ascend
619 Ascend
620 Descend
620 Descend
620 Ascend
622 Descend
625 Descend
629 Descend
630 Descend
631 Descend
632 Descend
632 Descend
633 Ascend
633 Ascend
633 Ascend
634 Ascend
634 Ascend
634 Ascend

635	Ascend
636	Descend
636	Descend
636	Descend
638	Descend
638	Descend
639	Descend
639	Descend
639	Descend
640	Descend
640	Descend
641	Descend
641	Descend
641	Descend
642	Ascend
642	Ascend
643	Ascend
643	Ascend
644	Descend
644	Descend
645	Descend
645	Descend
647	Descend
647	Descend
647	Descend
647	Descend
649	Descend
649	Descend
651	Descend
657	Descend
660	Descend
663	Descend
667	Descend
672	Descend
683	Descend
689	Descend
701	Ascend
718	Ascend
721	Ascend
724	Descend
731	Descend
751	Descend

752	Ascend
755	Ascend
757	Ascend
761	Ascend
764	Ascend
767	Ascend
770	Ascend
772	Ascend
772	Ascend
778	Descend
780	Ascend
780	Ascend
785	Ascend
786	Ascend
788	Ascend
794	Ascend
799	Descend
802	Ascend
803	Descend
805	Ascend
807	Descend
817	Descend
822	Ascend
824	Ascend
826	Descend
827	Descend
830	Ascend
842	Descend
868	Ascend
872	Ascend
887	Ascend
897	Descend
900	Descend
909	Ascend
909	Descend
911	Ascend
912	Ascend
918	Ascend
922	Descend
941	Descend
942	Descend
945	Ascend

947	Ascend
950	Descend
953	Descend
953	Ascend
955	Ascend
957	Ascend
958	Ascend
961	Descend
961	Descend
962	Descend
967	Descend
968	Ascend
970	Descend
987	Ascend
988	Descend
999	Descend
1000	Ascend
1005	Ascend
1008	Descend
1014	Ascend
1015	Descend
7	AntIn
13	AntIn
15	AntOut
17	AntOut
17	AntOut
19	AntIn
20	AntOut
24	AntIn
26	AntIn
26	AntIn
27	AntOut
28	AntIn
29	AntIn
31	AntOut
31	AntOut
33	AntIn
35	AntIn
35	AntIn
38	AntIn
40	AntOut
40	AntIn

41	AntOut
43	AntIn
45	AntIn
45	AntOut
47	AntIn
50	AntIn
53	AntOut
56	AntOut
58	AntIn
64	AntOut
66	AntOut
67	AntIn
69	AntIn
73	AntOut
76	AntIn
77	AntIn
81	AntIn
81	AntIn
83	AntIn
89	AntIn
91	AntIn
92	AntIn
92	AntIn
93	AntIn
98	AntOut
100	AntIn
109	AntIn
112	AntIn
116	AntOut
125	AntOut
129	AntIn
130	AntIn
131	AntIn
132	AntIn
132	AntIn
135	AntOut
135	AntOut
138	AntIn
141	AntIn
143	AntIn
144	AntIn
145	AntIn

146	AntOut
148	AntIn
153	AntOut
155	AntOut
172	AntIn
173	AntIn
174	AntIn
177	AntIn
186	AntIn
186	AntIn
188	AntIn
189	AntIn
189	AntIn
191	AntOut
192	AntIn
194	AntIn
198	AntIn
201	AntIn
202	AntIn
203	AntOut
209	AntOut
217	AntOut
217	AntOut
218	AntOut
219	AntIn
220	AntIn
222	AntOut
223	AntIn
225	AntOut
225	AntOut
228	AntIn
230	AntIn
234	AntOut
235	AntOut
237	AntOut
237	AntOut
238	AntOut
241	AntOut
241	AntOut
242	AntIn
246	AntIn
247	AntOut

248	AntIn
249	AntIn
250	AntIn
250	AntIn
250	AntOut
254	AntIn
264	AntOut
265	AntOut
266	AntOut
269	AntIn
269	AntIn
275	AntIn
279	AntOut
280	AntIn
295	AntOut
295	AntOut
298	AntIn
302	AntIn
302	AntIn
305	AntOut
306	AntIn
309	AntIn
312	AntIn
312	AntOut
318	AntIn
321	AntOut
322	AntOut
322	AntIn
324	AntIn
324	AntIn
325	AntIn
327	AntOut
327	AntOut
330	AntOut
333	AntIn
334	AntOut
335	AntIn
338	AntIn
339	AntIn
339	AntOut
341	AntOut
341	AntIn

343 AntIn
346 AntIn
349 AntIn
352 AntOut
353 AntIn
359 AntIn
360 AntIn
361 AntOut
368 AntIn
378 AntIn
380 AntIn
381 AntOut
383 AntOut
387 AntOut
389 AntOut
392 AntIn
393 AntIn
395 AntOut
398 AntOut
398 AntOut
399 AntIn
404 AntOut
406 AntIn
408 AntIn
412 AntOut
413 AntOut
414 AntOut
417 AntIn
420 AntIn
420 AntOut
424 AntIn
425 AntIn
428 AntOut
429 AntIn
442 AntIn
447 AntIn
449 AntIn
452 AntIn
455 AntIn
455 AntOut
456 AntIn
457 AntIn

460 AntOut
462 AntIn
468 AntIn
475 AntOut
478 AntOut
478 AntOut
478 AntOut
479 AntOut
484 AntIn
484 AntOut
486 AntIn
510 AntOut
510 AntIn
511 AntIn
512 AntOut
512 AntOut
514 AntIn
514 AntIn
517 AntOut
518 AntIn
529 AntOut
532 AntIn
536 AntOut
542 AntOut
543 AntOut
552 AntOut
554 AntIn
562 AntOut
566 AntIn
567 AntOut
567 AntOut
582 AntOut
583 AntIn
584 AntIn
584 AntIn
587 AntIn
587 AntIn
591 AntIn
592 AntOut
593 AntIn
600 AntIn
601 AntIn

602 AntOut
603 AntOut
606 AntIn
610 AntIn
611 AntOut
614 AntIn
618 AntIn
622 AntIn
623 AntIn
625 AntIn
635 AntOut
648 AntOut
657 AntOut
660 AntOut
661 AntIn
678 AntIn
678 AntIn
689 AntIn
698 AntIn
704 AntIn
704 AntIn
712 AntOut
729 AntIn
737 AntIn
751 AntIn
759 AntIn
761 AntIn
767 AntIn
780 AntIn
791 AntIn
792 AntIn
792 AntOut
797 AntIn
800 AntIn
803 AntOut
805 AntOut
817 AntIn
825 AntIn
826 AntIn
830 AntIn
850 AntIn
855 AntIn

886	AntIn
886	AntIn
889	AntIn
912	AntOut
915	AntOut
916	AntIn
918	AntIn
920	AntIn
924	AntOut
926	AntIn
928	AntIn
939	AntIn
939	AntIn
940	AntIn
944	AntIn
945	AntIn
946	AntIn
958	AntIn
963	AntOut
964	AntIn
969	AntIn
990	AntIn
995	AntOut
1005	AntIn
1010	AntIn
1019	AntIn
