# Supplementary material for: Interactions Increase Forager Availability and Activity in Harvester Ants
Source: PLoS One. 2015 Nov 5;10(11):e0141971. doi: 10.1371/journal.pone.0141971 (PMC4635008; doi:10.1371/journal.pone.0141971)
Supplement: S3 Dataset — We observed and filmed behavior inside the nest during and after forager removals. This dataset shows our counts made from the films of the numbers of returning and outgoing foragers at the nest entrance and the number of ascending and descending ants at all tunnel entrances. (ZIP) [file pone.0141971.s004.zip › S3 Dataset/2013 Correlation Data 367 8-18.pdf]

**Researcher**	**Jovel Queirolo**
**Colony 367**	
**8/18/13**	
**Video time (seconds)**	**Event**
3	Descend
3	Descend
4	Descend
6	Descend
9	Ascend
11	Ascend
12	Ascend
12	Descend
15	Descend
15	Ascend
18	Descend
23	Descend
24	Descend
26	Ascend
27	Descend
28	Ascend
29	Descend
32	Descend
33	Descend
37	Descend
37	Ascend
40	Descend
43	Ascend
44	Descend
44	Descend
47	Ascend
48	Ascend
50	Descend
52	Descend
54	Ascend
58	Descend
59	Descend
59	Ascend
60	Descend
61	Descend
62	Descend
62	Ascend

70	Descend
71	Ascend
72	Descend
73	Ascend
86	Descend
89	Descend
93	Descend
95	Descend
96	Descend
97	Descend
100	Descend
103	Descend
107	Ascend
109	Descend
111	Descend
115	Descend
119	Descend
124	Descend
131	Descend
132	Descend
133	Descend
135	Ascend
138	Descend
142	Descend
147	Ascend
148	Descend
149	Descend
150	Ascend
150	Descend
151	Descend
152	Ascend
153	Descend
155	Ascend
158	Descend
159	Ascend
159	Descend
160	Descend
161	Descend
161	Ascend
162	Ascend
163	Ascend
164	Descend

165	Descend
166	Ascend
167	Ascend
168	Descend
169	Descend
169	Ascend
170	Ascend
170	Ascend
172	Ascend
173	Ascend
174	Ascend
176	Ascend
178	Ascend
179	Descend
180	Descend
180	Descend
180	Descend
181	Descend
182	Descend
183	Descend
187	Ascend
188	Ascend
189	Ascend
190	Descend
192	Ascend
194	Ascend
196	Descend
198	Ascend
203	Ascend
204	Ascend
205	Descend
205	Ascend
207	Ascend
207	Ascend
208	Ascend
209	Ascend
210	Ascend
211	Ascend
212	Descend
212	Ascend
213	Descend
213	Descend

213	Ascend
214	Ascend
216	Ascend
217	Ascend
218	Descend
218	Descend
220	Descend
220	Descend
222	Descend
223	Ascend
224	Descend
225	Ascend
227	Ascend
229	Descend
230	Ascend
232	Ascend
232	Descend
233	Ascend
234	Ascend
235	Ascend
235	Ascend
236	Descend
237	Descend
237	Ascend
237	Ascend
238	Ascend
238	Ascend
239	Ascend
239	Descend
240	Descend
240	Descend
241	Descend
242	Descend
243	Ascend
244	Ascend
244	Ascend
245	Ascend
245	Descend
246	Descend
247	Descend
248	Ascend
249	Ascend

250	Ascend
250	Ascend
251	Ascend
253	Ascend
255	Descend
256	Descend
256	Ascend
257	Ascend
259	Descend
259	Descend
260	Descend
261	Descend
261	Descend
262	Descend
262	Ascend
263	Ascend
263	Descend
264	Descend
264	Descend
265	Descend
266	Descend
267	Descend
267	Descend
268	Descend
269	Ascend
269	Ascend
269	Ascend
270	Ascend
270	Descend
271	Descend
271	Ascend
271	Ascend
272	Descend
272	Descend
273	Ascend
273	Ascend
275	Descend
276	Descend
276	Ascend
276	Ascend
277	Ascend
277	Ascend

278	Ascend
279	Ascend
280	Ascend
280	Descend
281	Descend
282	Ascend
282	Ascend
283	Descend
284	Descend
285	Descend
286	Descend
286	Ascend
286	Ascend
287	Ascend
289	Ascend
290	Ascend
291	Descend
293	Descend
294	Descend
295	Descend
296	Descend
297	Ascend
298	Ascend
301	Ascend
302	Ascend
302	Ascend
303	Descend
304	Descend
307	Descend
308	Descend
310	Descend
312	Ascend
312	Ascend
314	Ascend
314	Ascend
317	Ascend
317	Descend
318	Descend
319	Descend
320	Descend
321	Ascend
321	Ascend

322	Descend
322	Descend
323	Ascend
323	Ascend
324	Ascend
325	Descend
325	Descend
326	Descend
327	Ascend
327	Ascend
328	Ascend
328	Ascend
329	Ascend
330	Ascend
331	Descend
331	Descend
332	Descend
332	Descend
333	Descend
333	Ascend
334	Ascend
334	Descend
334	Descend
335	Ascend
335	Ascend
336	Descend
336	Descend
337	Ascend
337	Ascend
338	Ascend
339	Ascend
339	Ascend
340	Descend
341	Descend
342	Ascend
343	Descend
344	Ascend
345	Ascend
345	Descend
346	Descend
347	Descend
347	Descend

348	Descend
349	Descend
349	Ascend
350	Ascend
351	Ascend
351	Ascend
353	Ascend
353	Descend
355	Descend
355	Ascend
356	Descend
356	Descend
356	Descend
356	Descend
357	Ascend
357	Ascend
358	Descend
358	Descend
358	Descend
359	Descend
359	Ascend
360	Ascend
360	Descend
361	Descend
365	Descend
366	Descend
366	Descend
368	Descend
370	Ascend
370	Ascend
371	Descend
372	Ascend
373	Ascend
373	Descend
374	Descend
374	Descend
377	Descend
378	Ascend
380	Descend
381	Ascend
382	Ascend
384	Ascend

386	Descend
388	Ascend
388	Ascend
390	Ascend
391	Descend
393	Descend
394	Ascend
399	Descend
400	Descend
401	Descend
404	Ascend
406	Ascend
406	Ascend
411	Ascend
414	Ascend
419	Descend
420	Descend
426	Ascend
430	Ascend
432	Ascend
440	Descend
445	Descend
451	Descend
455	Ascend
456	Ascend
457	Ascend
461	Descend
463	Ascend
464	Ascend
465	Descend
468	Descend
476	Descend
478	Descend
480	Descend
488	Descend
494	Descend
499	Ascend
506	Descend
507	Ascend
513	Descend
523	Descend
529	Ascend

539	Descend
546	Descend
548	Descend
552	Descend
554	Descend
556	Descend
585	Ascend
588	Descend
595	Ascend
596	Descend
601	Ascend
611	Descend
615	Descend
619	Descend
620	Ascend
622	Descend
629	Descend
678	Descend
695	Descend
701	Descend
704	Ascend
706	Descend
706	Descend
707	Ascend
709	Descend
710	Descend
711	Ascend
716	Descend
718	Ascend
727	Ascend
728	Ascend
751	Descend
752	Descend
754	Descend
755	Descend
761	Descend
767	Descend
768	Descend
770	Descend
776	Descend
780	Descend
784	Descend

786	Descend
794	Descend
798	Descend
799	Descend
803	Descend
804	Descend
807	Descend
815	Ascend
818	Descend
821	Descend
822	Descend
824	Descend
825	Descend
826	Descend
827	Ascend
832	Descend
833	Descend
836	Ascend
842	Ascend
843	Descend
844	Ascend
884	Descend
885	Descend
886	Ascend
892	Descend
896	Ascend
7	AntIn
11	AntIn
14	AntOut
16	AntIn
17	AntIn
17	AntIn
18	AntOut
19	AntOut
21	AntIn
23	AntOut
26	AntIn
26	AntIn
27	AntIn
28	AntIn
29	AntOut
30	AntIn

31	AntOut
33	AntIn
37	AntIn
38	AntOut
39	AntIn
39	AntIn
45	AntIn
48	AntOut
53	AntIn
53	AntIn
55	AntOut
57	AntOut
59	AntOut
69	AntOut
71	AntIn
72	AntOut
72	AntIn
73	AntIn
74	AntOut
76	AntIn
82	AntIn
82	AntIn
83	AntIn
86	AntIn
87	AntIn
88	AntIn
92	AntIn
92	AntOut
93	AntOut
93	AntOut
94	AntIn
95	AntIn
96	AntIn
98	AntOut
101	AntIn
103	AntIn
103	AntIn
110	AntOut
111	AntIn
113	AntIn
115	AntIn
117	AntOut

117	AntOut
124	AntIn
125	AntIn
125	AntIn
126	AntIn
127	AntIn
129	AntIn
133	AntIn
139	AntIn
145	AntIn
146	AntIn
146	AntIn
147	AntOut
151	AntIn
152	AntIn
152	AntIn
153	AntIn
154	AntIn
154	AntIn
157	AntIn
159	AntIn
160	AntOut
164	AntIn
166	AntOut
167	AntIn
167	AntIn
168	AntOut
171	AntIn
171	AntIn
177	AntIn
178	AntOut
181	AntIn
183	AntIn
186	AntOut
187	AntOut
188	AntIn
192	AntIn
194	AntIn
194	AntIn
197	AntOut
198	AntIn
199	AntIn

199	AntIn
200	AntIn
200	AntOut
202	AntIn
203	AntOut
204	AntIn
204	AntIn
207	AntIn
208	AntOut
211	AntIn
212	AntIn
214	AntIn
220	AntOut
222	AntIn
222	AntIn
225	AntOut
233	AntIn
233	AntIn
234	AntIn
237	AntIn
245	AntIn
253	AntOut
253	AntIn
255	AntIn
255	AntIn
256	AntIn
256	AntIn
257	AntIn
257	AntIn
258	AntIn
260	AntIn
261	AntIn
261	AntOut
262	AntIn
265	AntOut
267	AntOut
273	AntIn
278	AntIn
278	AntIn
279	AntIn
282	AntOut
283	AntOut

286	AntIn
287	AntIn
287	AntIn
288	AntOut
290	AntOut
290	AntIn
291	AntOut
292	AntIn
296	AntOut
297	AntIn
300	AntIn
302	AntOut
302	AntIn
303	AntIn
304	AntOut
304	AntIn
305	AntIn
305	AntIn
306	AntIn
309	AntOut
313	AntOut
314	AntOut
317	AntOut
317	AntOut
318	AntOut
318	AntOut
319	AntIn
320	AntIn
321	AntIn
321	AntIn
322	AntIn
323	AntOut
323	AntIn
324	AntIn
325	AntIn
326	AntIn
327	AntIn
327	AntIn
328	AntOut
330	AntIn
330	AntIn
331	AntIn

332	AntIn
334	AntOut
334	AntIn
336	AntOut
337	AntOut
338	AntIn
340	AntIn
340	AntIn
341	AntIn
341	AntIn
342	AntIn
343	AntOut
350	AntIn
355	AntOut
356	AntOut
360	AntIn
360	AntIn
361	AntIn
361	AntOut
370	AntOut
372	AntIn
373	AntOut
373	AntIn
376	AntOut
379	AntOut
385	AntOut
387	AntIn
394	AntOut
395	AntOut
397	AntOut
403	AntOut
405	AntOut
406	AntOut
412	AntOut
414	AntIn
416	AntIn
420	AntIn
424	AntOut
425	AntIn
450	AntIn
474	AntOut
493	AntOut

496	AntIn
551	AntIn
593	AntIn
610	AntIn
615	AntIn
619	AntIn
621	AntOut
627	AntIn
670	AntIn
672	AntIn
696	AntIn
702	AntIn
706	AntIn
707	AntIn
708	AntIn
742	AntIn
750	AntIn
751	AntIn
751	AntIn
756	AntIn
758	AntIn
763	AntIn
766	AntIn
772	AntIn
774	AntIn
780	AntIn
783	AntOut
791	AntIn
793	AntOut
793	AntIn
794	AntIn
795	AntIn
795	AntIn
796	AntIn
798	AntIn
809	AntIn
812	AntIn
827	AntOut
829	AntIn
843	AntOut
869	AntIn
874	AntIn
