# Supplementary material for: Interactions Increase Forager Availability and Activity in Harvester Ants
Source: PLoS One. 2015 Nov 5;10(11):e0141971. doi: 10.1371/journal.pone.0141971 (PMC4635008; doi:10.1371/journal.pone.0141971)
Supplement: S3 Dataset — We observed and filmed behavior inside the nest during and after forager removals. This dataset shows our counts made from the films of the numbers of returning and outgoing foragers at the nest entrance and the number of ascending and descending ants at all tunnel entrances. (ZIP) [file pone.0141971.s004.zip › S3 Dataset/2013 Correlation Data 367 8-19.pdf]

**Researcher**	**Jovel Queirolo**
**Colony 367**	
**8/19/13**	
**Video time (seconds)**	**Event**
2	Ascend
3	Descend
3	Descend
10	Descend
12	Ascend
13	Ascend
13	Descend
14	Ascend
15	Ascend
15	Ascend
16	Descend
17	Ascend
19	Ascend
21	Ascend
21	Ascend
22	Ascend
22	Descend
23	Descend
23	Ascend
29	Descend
29	Ascend
30	Ascend
32	Descend
33	Descend
35	Ascend
36	Ascend
37	Descend
38	Descend
40	Descend
40	Ascend
41	Descend
43	Ascend
43	Ascend
44	Ascend
46	Descend
47	Descend
48	Descend

50	Descend
51	Ascend
52	Ascend
52	Ascend
53	Ascend
54	Ascend
54	Ascend
55	Descend
56	Ascend
56	Ascend
57	Descend
59	Descend
61	Descend
62	Descend
63	Ascend
63	Ascend
64	Ascend
65	Descend
65	Descend
66	Descend
66	Ascend
70	Descend
72	Ascend
73	Ascend
75	Ascend
75	Descend
77	Descend
78	Ascend
79	Ascend
83	Descend
84	Descend
84	Descend
85	Ascend
86	Ascend
86	Descend
87	Descend
87	Descend
88	Descend
89	Ascend
89	Ascend
91	Ascend
92	Ascend

92	Ascend
92	Ascend
94	Ascend
94	Descend
95	Descend
95	Ascend
96	Descend
96	Ascend
97	Ascend
98	Ascend
98	Ascend
99	Ascend
99	Ascend
100	Ascend
100	Ascend
101	Ascend
101	Descend
102	Descend
102	Descend
104	Ascend
105	Ascend
105	Ascend
106	Descend
106	Descend
107	Ascend
110	Descend
111	Ascend
112	Ascend
113	Descend
115	Descend
116	Ascend
118	Ascend
119	Ascend
123	Ascend
125	Ascend
125	Ascend
128	Descend
129	Ascend
129	Ascend
130	Descend
130	Descend
131	Ascend

131	Descend
133	Descend
133	Ascend
134	Ascend
135	Ascend
135	Ascend
136	Descend
137	Descend
137	Descend
138	Ascend
138	Ascend
140	Ascend
142	Ascend
143	Descend
145	Descend
146	Descend
146	Ascend
148	Descend
150	Descend
151	Ascend
152	Descend
154	Descend
154	Ascend
154	Ascend
155	Ascend
156	Ascend
156	Descend
156	Ascend
157	Ascend
158	Descend
158	Descend
159	Descend
159	Descend
160	Ascend
160	Ascend
161	Ascend
164	Ascend
165	Descend
168	Ascend
168	Ascend
169	Ascend
170	Descend

170	Descend
171	Descend
171	Descend
172	Descend
173	Ascend
174	Ascend
174	Ascend
175	Ascend
176	Ascend
178	Descend
179	Descend
180	Descend
181	Ascend
183	Ascend
185	Ascend
185	Ascend
186	Ascend
186	Descend
187	Descend
188	Ascend
189	Ascend
190	Descend
190	Descend
192	Descend
192	Descend
192	Descend
193	Descend
194	Ascend
197	Descend
200	Ascend
201	Ascend
201	Ascend
202	Ascend
202	Ascend
204	Ascend
207	Descend
208	Descend
211	Descend
212	Descend
212	Ascend
213	Descend
215	Ascend

216	Ascend
217	Ascend
217	Descend
218	Descend
219	Ascend
220	Descend
220	Descend
221	Descend
221	Ascend
222	Ascend
222	Ascend
223	Ascend
223	Ascend
224	Ascend
224	Ascend
224	Ascend
225	Ascend
225	Ascend
226	Ascend
226	Ascend
227	Ascend
228	Ascend
228	Ascend
229	Ascend
230	Descend
231	Descend
232	Descend
232	Ascend
232	Ascend
235	Descend
235	Ascend
237	Ascend
238	Ascend
239	Descend
240	Ascend
240	Ascend
241	Descend
241	Descend
242	Descend
243	Ascend
243	Descend
243	Descend

245	Descend
245	Ascend
249	Descend
251	Ascend
251	Ascend
253	Descend
255	Descend
256	Ascend
258	Ascend
259	Descend
259	Descend
260	Descend
261	Descend
262	Descend
262	Descend
263	Descend
264	Descend
265	Descend
266	Descend
266	Descend
267	Descend
267	Ascend
269	Descend
270	Ascend
270	Ascend
271	Ascend
272	Ascend
272	Ascend
274	Ascend
275	Ascend
275	Ascend
277	Descend
277	Descend
278	Descend
279	Descend
279	Descend
280	Descend
281	Ascend
282	Ascend
282	Descend
283	Descend
283	Descend

284	Ascend
284	Descend
284	Ascend
287	Ascend
287	Ascend
288	Ascend
288	Ascend
289	Ascend
290	Descend
290	Ascend
291	Ascend
292	Ascend
293	Ascend
293	Ascend
294	Ascend
295	Descend
297	Descend
298	Descend
300	Ascend
301	Ascend
301	Ascend
301	Ascend
302	Ascend
304	Ascend
304	Ascend
305	Ascend
305	Ascend
307	Ascend
307	Ascend
308	Ascend
309	Descend
309	Descend
310	Ascend
310	Ascend
311	Ascend
312	Ascend
315	Descend
316	Descend
317	Descend
318	Ascend
321	Descend
322	Ascend

324	Descend
324	Descend
325	Descend
325	Descend
326	Descend
326	Ascend
327	Ascend
327	Ascend
328	Descend
328	Descend
329	Ascend
329	Ascend
330	Ascend
331	Ascend
331	Descend
331	Descend
332	Ascend
332	Ascend
333	Descend
335	Descend
336	Ascend
336	Ascend
337	Ascend
338	Ascend
339	Descend
340	Descend
340	Ascend
341	Descend
342	Descend
342	Ascend
345	Ascend
345	Ascend
346	Ascend
346	Descend
346	Descend
348	Descend
348	Ascend
348	Descend
349	Descend
349	Ascend
350	Ascend
350	Ascend

351	Descend
351	Descend
351	Descend
353	Descend
353	Ascend
353	Ascend
355	Ascend
356	Ascend
357	Ascend
358	Ascend
360	Descend
11	AntIn
12	AntIn
14	AntIn
15	AntOut
17	AntOut
22	AntIn
25	AntOut
26	AntIn
26	AntOut
27	AntOut
29	AntIn
33	AntOut
39	AntOut
40	AntIn
41	AntIn
43	AntIn
43	AntOut
44	AntIn
45	AntOut
46	AntIn
46	AntIn
49	AntIn
52	AntOut
54	AntIn
57	AntIn
58	AntOut
59	AntOut
59	AntIn
61	AntIn
63	AntIn
64	AntIn

66	AntOut
66	AntOut
67	AntIn
68	AntIn
69	AntIn
70	AntOut
71	AntOut
72	AntIn
73	AntIn
74	AntOut
75	AntIn
77	AntOut
78	AntIn
79	AntIn
79	AntIn
80	AntOut
81	AntIn
83	AntIn
87	AntIn
99	AntIn
104	AntIn
107	AntOut
108	AntIn
109	AntIn
110	AntOut
110	AntOut
112	AntIn
113	AntIn
113	AntIn
115	AntIn
121	AntIn
121	AntIn
123	AntIn
125	AntOut
127	AntIn
128	AntOut
128	AntOut
128	AntIn
129	AntOut
131	AntIn
131	AntIn
134	AntIn

141	AntIn
142	AntIn
143	AntIn
143	AntIn
144	AntOut
145	AntIn
146	AntIn
149	AntOut
150	AntIn
151	AntIn
152	AntIn
154	AntIn
154	AntIn
156	AntOut
159	AntOut
161	AntOut
162	AntIn
162	AntIn
162	AntIn
165	AntOut
165	AntIn
166	AntIn
167	AntIn
168	AntIn
173	AntIn
174	AntIn
175	AntIn
176	AntOut
178	AntIn
181	AntIn
181	AntOut
182	AntIn
183	AntIn
183	AntIn
184	AntOut
184	AntOut
186	AntIn
186	AntOut
188	AntOut
191	AntOut
193	AntOut
193	AntIn

194	AntIn
196	AntIn
197	AntIn
198	AntOut
198	AntOut
199	AntIn
201	AntOut
201	AntIn
201	AntIn
203	AntIn
206	AntOut
207	AntIn
207	AntIn
208	AntOut
210	AntOut
212	AntIn
212	AntIn
213	AntIn
213	AntOut
216	AntIn
217	AntOut
217	AntOut
218	AntIn
220	AntOut
221	AntOut
222	AntIn
223	AntIn
224	AntIn
226	AntOut
229	AntOut
229	AntOut
230	AntIn
230	AntIn
231	AntIn
233	AntIn
233	AntOut
237	AntOut
240	AntIn
240	AntIn
242	AntIn
242	AntIn
244	AntOut

245	AntIn
246	AntIn
247	AntIn
248	AntIn
249	AntOut
249	AntOut
250	AntOut
251	AntOut
252	AntOut
252	AntOut
253	AntOut
255	AntIn
256	AntOut
256	AntIn
257	AntIn
257	AntIn
257	AntIn
258	AntIn
259	AntIn
262	AntIn
265	AntOut
267	AntOut
268	AntIn
269	AntIn
270	AntIn
272	AntOut
273	AntIn
274	AntIn
274	AntIn
278	AntIn
278	AntIn
278	AntIn
279	AntIn
280	AntOut
280	AntOut
282	AntOut
288	AntIn
290	AntIn
290	AntOut
290	AntOut
293	AntIn
293	AntIn

296 AntIn
299 AntIn
303 AntIn
304 AntIn
304 AntIn
304 AntIn
305 AntOut
305 AntOut
306 AntOut
308 AntIn
312 AntOut
313 AntIn
313 AntIn
317 AntOut
318 AntOut
318 AntOut
318 AntOut
319 AntOut
321 AntOut
322 AntOut
323 AntIn
324 AntIn
327 AntIn
327 AntIn
328 AntIn
329 AntIn
332 AntIn
333 AntOut
334 AntIn
334 AntOut
335 AntIn
336 AntOut
337 AntIn
337 AntIn
338 AntOut
339 AntIn
340 AntIn
340 AntIn
345 AntIn
346 AntOut
347 AntIn
351 AntIn

351	AntIn
352	AntIn
355	AntIn
355	AntIn
359	AntIn
360	AntIn
362	AntOut
