# Supplementary material for: Interactions Increase Forager Availability and Activity in Harvester Ants
Source: PLoS One. 2015 Nov 5;10(11):e0141971. doi: 10.1371/journal.pone.0141971 (PMC4635008; doi:10.1371/journal.pone.0141971)
Supplement: S3 Dataset — We observed and filmed behavior inside the nest during and after forager removals. This dataset shows our counts made from the films of the numbers of returning and outgoing foragers at the nest entrance and the number of ascending and descending ants at all tunnel entrances. (ZIP) [file pone.0141971.s004.zip › S3 Dataset/2013 Correlation Data 367 8-20.pdf]

Researcher **Jovel Queirolo**
Colony 367
8/20/13

**Video time (seconds)**	**Event**
5	Ascend
5	Ascend
6	Ascend
8	Ascend
9	Descend
10	Descend
11	Descend
12	Ascend
12	Descend
17	Descend
18	Descend
19	Descend
20	Descend
20	Descend
21	Descend
21	Descend
22	Descend
22	Descend
23	Ascend
24	Descend
25	Descend
30	Ascend
31	Descend
32	Ascend
35	Descend
36	Ascend
37	Ascend
38	Ascend
41	Descend
41	Descend
42	Descend
46	Ascend
47	Ascend
47	Descend
47	Descend
48	Ascend
49	Ascend

49	Ascend
50	Descend
50	Descend
50	Descend
51	Descend
51	Descend
51	Descend
52	Ascend
53	Ascend
53	Ascend
54	Ascend
54	Ascend
54	Ascend
54	Ascend
55	Ascend
55	Ascend
56	Ascend
56	Ascend
57	Ascend
57	Descend
58	Descend
58	Descend
60	Descend
60	Descend
60	Ascend
61	Ascend
61	Ascend
61	Descend
62	Descend
62	Descend
63	Descend
64	Descend
64	Descend
65	Descend
66	Ascend
67	Descend
68	Descend
68	Ascend
69	Ascend
70	Descend
72	Ascend
73	Ascend

73	Ascend
74	Descend
75	Descend
76	Ascend
76	Ascend
77	Ascend
77	Ascend
77	Ascend
77	Ascend
78	Ascend
78	Ascend
79	Ascend
79	Ascend
79	Ascend
80	Ascend
80	Ascend
80	Ascend
80	Ascend
81	Ascend
81	Ascend
81	Ascend
82	Ascend
83	Descend
83	Descend
83	Descend
84	Ascend
84	Ascend
84	Ascend
84	Descend
85	Descend
85	Descend
85	Descend
85	Descend
85	Descend
86	Descend
86	Descend
86	Ascend
87	Ascend
87	Ascend
87	Ascend
87	Ascend
87	Ascend

88	Ascend
88	Ascend
89	Descend
89	Descend
89	Descend
89	Descend
90	Descend
90	Descend
90	Descend
91	Descend
91	Descend
91	Descend
91	Ascend
92	Ascend
92	Ascend
93	Descend
93	Descend
93	Ascend
94	Ascend
95	Ascend
95	Ascend
96	Descend
96	Descend
96	Descend
97	Descend
97	Descend
98	Descend
98	Descend
98	Descend
100	Descend
100	Descend
101	Descend
101	Descend
101	Descend
102	Descend
103	Ascend
103	Ascend
103	Ascend
103	Ascend
103	Ascend
104	Ascend
104	Ascend

104	Ascend
104	Ascend
104	Ascend
105	Ascend
105	Ascend
105	Ascend
105	Ascend
106	Ascend
106	Ascend
106	Descend
106	Descend
106	Descend
107	Descend
107	Descend
107	Descend
107	Descend
108	Descend
108	Descend
108	Descend
108	Descend
109	Descend
109	Descend
109	Ascend
109	Ascend
110	Ascend
110	Ascend
110	Ascend
110	Ascend
110	Ascend
111	Ascend
111	Ascend
111	Ascend
111	Ascend
111	Ascend
112	Ascend
112	Ascend
112	Ascend
112	Ascend
113	Descend
113	Descend
113	Descend
114	Descend

114	Descend
114	Ascend
115	Ascend
115	Ascend
115	Descend
115	Descend
115	Descend
116	Descend
116	Descend
116	Descend
117	Descend
117	Descend
117	Descend
117	Descend
119	Ascend
119	Ascend
119	Ascend
121	Descend
123	Descend
124	Descend
124	Descend
124	Descend
125	Descend
125	Ascend
125	Ascend
126	Ascend
127	Ascend
127	Ascend
127	Ascend
127	Ascend
128	Ascend
129	Ascend
129	Ascend
130	Descend
131	Ascend
132	Ascend
132	Ascend
132	Ascend
132	Ascend
133	Ascend
134	Ascend
135	Ascend

136	Ascend
136	Ascend
136	Ascend
137	Ascend
138	Ascend
139	Descend
140	Descend
140	Descend
140	Ascend
141	Ascend
141	Ascend
143	Descend
143	Descend
143	Descend
144	Descend
144	Descend
145	Descend
145	Ascend
146	Ascend
146	Descend
147	Ascend
147	Ascend
148	Ascend
149	Descend
149	Descend
150	Ascend
150	Ascend
151	Ascend
152	Ascend
152	Ascend
152	Descend
153	Descend
153	Descend
153	Descend
154	Descend
154	Descend
154	Ascend
154	Ascend
155	Ascend
155	Descend
155	Descend
155	Descend

156	Descend
156	Descend
157	Descend
158	Descend
160	Descend
160	Descend
160	Ascend
160	Ascend
160	Ascend
161	Ascend
161	Ascend
162	Ascend
162	Ascend
162	Descend
162	Descend
164	Descend
164	Descend
164	Ascend
164	Ascend
165	Ascend
165	Descend
166	Ascend
166	Ascend
166	Descend
167	Descend
167	Ascend
167	Ascend
168	Descend
169	Ascend
169	Ascend
170	Ascend
170	Ascend
171	Ascend
172	Ascend
173	Ascend
174	Ascend
175	Ascend
175	Ascend
176	Ascend
176	Ascend
177	Ascend
177	Ascend

178	Ascend
178	Descend
178	Descend
178	Descend
179	Descend
179	Descend
180	Descend
180	Descend
180	Descend
181	Descend
181	Descend
181	Descend
181	Descend
182	Descend
182	Descend
182	Descend
182	Descend
183	Descend
184	Descend
184	Descend
186	Descend
186	Descend
187	Ascend
187	Ascend
188	Descend
188	Descend
189	Ascend
190	Ascend
190	Ascend
191	Ascend
192	Ascend
192	Ascend
193	Ascend
193	Ascend
194	Ascend
194	Ascend
195	Ascend
195	Ascend
195	Ascend
195	Ascend
196	Ascend
196	Ascend

197	Descend
198	Descend
198	Descend
198	Descend
199	Descend
200	Descend
200	Descend
202	Descend
204	Descend
204	Descend
205	Descend
208	Descend
208	Ascend
209	Descend
209	Ascend
209	Ascend
210	Ascend
210	Ascend
210	Ascend
210	Ascend
211	Ascend
211	Ascend
211	Ascend
212	Ascend
212	Ascend
212	Ascend
213	Ascend
214	Ascend
214	Ascend
215	Ascend
215	Ascend
215	Ascend
216	Ascend
217	Ascend
217	Ascend
217	Ascend
217	Ascend
218	Ascend
218	Descend
218	Descend
218	Descend
219	Descend

219	Descend
220	Descend
220	Descend
221	Ascend
221	Ascend
222	Ascend
222	Ascend
223	Ascend
223	Ascend
223	Ascend
224	Ascend
224	Ascend
224	Ascend
225	Ascend
225	Descend
226	Descend
226	Ascend
226	Ascend
227	Ascend
227	Ascend
227	Ascend
228	Ascend
228	Ascend
229	Ascend
230	Ascend
231	Ascend
231	Descend
232	Descend
233	Descend
233	Descend
233	Descend
234	Descend
235	Descend
235	Descend
236	Descend
236	Descend
236	Ascend
237	Ascend
237	Ascend
237	Ascend
238	Ascend
238	Descend

239	Descend
239	Ascend
239	Ascend
240	Ascend
240	Descend
240	Descend
241	Descend
241	Descend
242	Descend
243	Descend
243	Ascend
243	Descend
243	Descend
244	Descend
244	Descend
244	Descend
244	Descend
245	Descend
245	Descend
245	Ascend
246	Descend
246	Descend
247	Descend
247	Descend
247	Descend
248	Ascend
249	Ascend
249	Ascend
249	Ascend
250	Ascend
250	Ascend
251	Ascend
251	Ascend
251	Ascend
251	Ascend
252	Ascend
252	Ascend
253	Descend
254	Descend
254	Descend
254	Descend
255	Descend

255	Descend
256	Descend
256	Ascend
256	Ascend
257	Ascend
257	Ascend
258	Descend
258	Descend
258	Descend
259	Descend
259	Descend
261	Descend
261	Descend
261	Descend
262	Descend
263	Descend
263	Descend
265	Descend
265	Descend
266	Descend
266	Descend
267	Ascend
267	Ascend
268	Ascend
268	Ascend
269	Ascend
269	Descend
269	Descend
270	Descend
271	Ascend
271	Ascend
271	Ascend
271	Ascend
272	Ascend
272	Ascend
272	Ascend
273	Ascend
273	Ascend
273	Ascend
273	Ascend
274	Ascend
274	Ascend

274	Ascend
275	Ascend
275	Ascend
275	Ascend
276	Ascend
277	Ascend
277	Ascend
277	Descend
277	Descend
278	Ascend
278	Ascend
278	Descend
279	Ascend
279	Ascend
280	Descend
280	Descend
281	Descend
281	Descend
282	Descend
282	Ascend
283	Ascend
283	Ascend
283	Ascend
285	Ascend
285	Ascend
285	Ascend
285	Ascend
286	Ascend
287	Ascend
288	Ascend
288	Ascend
289	Descend
289	Descend
290	Descend
290	Descend
290	Descend
290	Descend
291	Ascend
291	Ascend
291	Ascend
292	Ascend
292	Ascend

292	Ascend
292	Ascend
293	Ascend
294	Ascend
294	Ascend
294	Ascend
294	Ascend
295	Ascend
295	Ascend
295	Ascend
295	Ascend
295	Ascend
296	Ascend
296	Ascend
296	Ascend
296	Ascend
297	Ascend
297	Ascend
297	Ascend
298	Descend
298	Descend
298	Descend
299	Descend
300	Descend
301	Ascend
301	Ascend
302	Ascend
302	Ascend
302	Ascend
303	Ascend
303	Ascend
303	Ascend
303	Ascend
303	Ascend
304	Ascend
304	Ascend
304	Ascend
305	Ascend
305	Ascend
305	Ascend
306	Ascend
306	Ascend

307	Descend
307	Descend
307	Descend
308	Descend
308	Descend
308	Ascend
309	Ascend
309	Ascend
309	Ascend
309	Descend
310	Descend
311	Ascend
311	Descend
311	Descend
311	Descend
312	Descend
312	Descend
313	Descend
313	Descend
313	Descend
314	Descend
314	Descend
314	Descend
315	Descend
315	Descend
315	Descend
315	Descend
317	Descend
317	Descend
319	Descend
319	Descend
319	Ascend
320	Ascend
321	Descend
321	Descend
322	Descend
323	Ascend
323	Ascend
324	Descend
325	Ascend
325	Ascend
325	Ascend

326	Ascend
326	Ascend
327	Descend
327	Descend
327	Descend
329	Descend
329	Descend
329	Descend
329	Descend
329	Descend
330	Descend
331	Descend
331	Descend
331	Descend
332	Descend
332	Ascend
332	Ascend
332	Ascend
332	Ascend
333	Ascend
333	Ascend
333	Ascend
334	Ascend
334	Ascend
334	Descend
335	Descend
336	Ascend
336	Ascend
337	Ascend
337	Ascend
338	Ascend
338	Ascend
339	Ascend
339	Ascend
339	Ascend
340	Ascend
340	Ascend
340	Ascend
341	Ascend
341	Ascend
342	Ascend
342	Ascend

343	Ascend
344	Descend
345	Descend
346	Ascend
347	Descend
349	Ascend
349	Ascend
350	Ascend
352	Ascend
352	Ascend
353	Ascend
353	Ascend
354	Descend
354	Descend
354	Descend
357	Descend
357	Descend
358	Descend
358	Ascend
358	Ascend
362	Ascend
362	Ascend
363	Ascend
363	Ascend
363	Ascend
364	Ascend
364	Ascend
365	Ascend
367	Descend
367	Descend
368	Descend
368	Descend
369	Descend
369	Descend
369	Ascend
369	Ascend
370	Descend
370	Ascend
371	Ascend
372	Ascend
372	Ascend
372	Ascend

373	Descend
373	Descend
374	Descend
374	Descend
374	Descend
374	Descend
374	Descend
375	Descend
375	Descend
375	Descend
375	Descend
376	Descend
376	Descend
377	Descend
377	Descend
378	Descend
378	Ascend
379	Ascend
380	Ascend
380	Ascend
380	Ascend
380	Ascend
381	Ascend
381	Ascend
382	Ascend
382	Ascend
383	Descend
383	Descend
384	Descend
384	Descend
385	Descend
386	Descend
386	Descend
386	Descend
386	Descend
387	Descend
387	Descend
387	Descend
388	Descend
388	Descend
388	Descend
389	Descend

389	Descend
390	Descend
390	Descend
391	Descend
391	Descend
393	Descend
393	Descend
395	Descend
395	Ascend
396	Ascend
396	Ascend
396	Ascend
396	Ascend
397	Ascend
397	Ascend
397	Ascend
397	Ascend
398	Ascend
398	Ascend
398	Ascend
398	Ascend
399	Ascend
399	Ascend
400	Ascend
400	Ascend
400	Ascend
401	Ascend
401	Ascend
402	Descend
402	Descend
402	Descend
402	Descend
403	Descend
403	Descend
404	Ascend
404	Ascend
404	Ascend
405	Descend
405	Descend
405	Descend
405	Descend
406	Descend

406	Descend
407	Descend
408	Descend
408	Descend
409	Descend
409	Descend
414	Ascend
414	Ascend
414	Descend
415	Descend
418	Descend
418	Descend
419	Ascend
422	Descend
423	Descend
423	Descend
423	Descend
424	Descend
424	Ascend
426	Ascend
427	Descend
428	Ascend
429	Ascend
430	Ascend
430	Ascend
430	Ascend
431	Ascend
431	Ascend
431	Ascend
432	Ascend
433	Ascend
433	Ascend
433	Ascend
434	Ascend
435	Ascend
435	Ascend
436	Ascend
436	Ascend
437	Ascend
437	Ascend
438	Ascend
438	Ascend

439	Descend
440	Descend
443	Descend
443	Descend
444	Descend
444	Ascend
445	Descend
447	Descend
448	Descend
450	Descend
450	Ascend
451	Ascend
452	Descend
453	Descend
454	Descend
455	Descend
457	Descend
458	Descend
459	Ascend
459	Ascend
460	Ascend
462	Ascend
463	Ascend
463	Ascend
463	Ascend
464	Ascend
464	Ascend
465	Ascend
465	Ascend
465	Ascend
468	Descend
472	Descend
475	Descend
477	Descend
479	Descend
480	Descend
481	Descend
484	Descend
488	Descend
490	Descend
492	Descend
492	Descend

492	Descend
495	Descend
501	Descend
510	Descend
514	Ascend
517	Ascend
519	Ascend
528	Ascend
529	Descend
532	Descend
536	Descend
539	Descend
564	Descend
592	Descend
596	Ascend
599	Ascend
619	Ascend
641	Descend
649	Descend
655	Descend
656	Ascend
662	Descend
673	Descend
675	Descend
677	Descend
682	Descend
701	Descend
707	Descend
716	Descend
721	Descend
724	Descend
737	Descend
741	Descend
742	Descend
746	Descend
755	Descend
759	Descend
766	Descend
774	Descend
784	Descend
796	Descend
803	Descend

809	Descend
820	Descend
830	Descend
833	Ascend
847	Descend
865	Descend
866	Descend
867	Descend
870	Descend
886	Descend
906	Descend
907	Descend
910	Descend
917	Descend
928	Descend
942	Descend
949	Descend
950	Descend
960	Descend
961	Descend
964	Descend
965	Descend
968	Descend
975	Descend
1004	Descend
1023	Descend
1025	Descend
1032	Descend
1033	Descend
1042	Descend
1044	Descend
1049	Descend
1052	Descend
1069	Descend
1070	Descend
1091	Descend
1103	Descend
1113	Descend
1117	Descend
1130	Descend
1138	Descend
1151	Descend

1176	Descend
1178	Descend
1199	Descend
1242	Descend
1261	Descend
1261	Descend
1267	Descend
1297	Descend
1312	Descend
1313	Descend
1314	Descend
1318	Ascend
1319	Descend
1328	Ascend
1335	Descend
1337	Descend
1340	Descend
1354	Descend
1355	Descend
1355	Ascend
1358	Descend
1361	Descend
1385	Descend
1386	Ascend
1406	Descend
1409	Descend
1414	Descend
1416	Descend
1435	Descend
1444	Descend
1446	Descend
1458	Descend
1471	Descend
1503	Descend
1512	Descend
1561	Descend
1568	Descend
1576	Descend
1591	Descend
1597	Descend
1601	Descend
1624	Descend

1628	Ascend
1644	Descend
1648	Descend
1678	Descend
1688	Descend
1705	Ascend
1728	Descend
1766	Descend
1807	Descend
1810	Descend
1834	Descend
1843	Descend
1872	Descend
1928	Descend
1929	Descend
1955	Descend
1987	Descend
1990	Descend
2012	Descend
2026	Descend
2052	Descend
2073	Descend
2078	Descend
2098	Descend
2132	Descend
2158	Descend
2174	Descend
2215	Descend
2216	Descend
2273	Descend
2275	Descend
2308	Descend
2324	Descend
2353	Descend
2378	Descend
2386	Ascend
2395	Descend
2487	Descend
2592	Descend
2602	Descend
2691	Descend
10	AntIn

10	AntOut
11	AntOut
12	AntIn
13	AntOut
13	AntOut
14	AntIn
14	AntIn
16	AntIn
20	AntIn
20	AntOut
21	AntIn
23	AntOut
24	AntIn
24	AntOut
24	AntOut
25	AntIn
26	AntIn
27	AntOut
28	AntOut
29	AntOut
30	AntIn
32	AntIn
33	AntOut
34	AntIn
36	AntIn
37	AntIn
38	AntIn
38	AntIn
38	AntIn
39	AntIn
39	AntOut
40	AntIn
40	AntIn
41	AntIn
41	AntOut
41	AntOut
42	AntIn
42	AntIn
43	AntOut
44	AntOut
45	AntIn
45	AntIn

46	AntOut
49	AntIn
49	AntIn
51	AntIn
53	AntIn
55	AntIn
55	AntOut
56	AntIn
58	AntIn
58	AntOut
59	AntOut
60	AntOut
62	AntIn
62	AntIn
64	AntIn
66	AntIn
68	AntOut
71	AntOut
72	AntIn
72	AntIn
73	AntIn
73	AntIn
74	AntIn
74	AntOut
75	AntIn
76	AntIn
77	AntIn
77	AntIn
79	AntIn
79	AntOut
80	AntIn
80	AntIn
81	AntIn
83	AntIn
83	AntOut
84	AntIn
84	AntIn
84	AntIn
89	AntOut
91	AntIn
93	AntIn
94	AntIn

94	AntOut
96	AntIn
98	AntOut
99	AntIn
100	AntIn
100	AntIn
101	AntIn
102	AntOut
104	AntIn
106	AntOut
108	AntIn
109	AntIn
110	AntOut
111	AntIn
111	AntOut
111	AntOut
112	AntIn
112	AntIn
113	AntIn
113	AntIn
114	AntOut
115	AntOut
116	AntOut
119	AntOut
120	AntIn
121	AntOut
122	AntOut
122	AntOut
123	AntIn
124	AntIn
125	AntIn
127	AntOut
128	AntOut
129	AntIn
129	AntOut
130	AntIn
130	AntIn
131	AntIn
132	AntIn
133	AntOut
133	AntOut
134	AntOut

136	AntIn
136	AntOut
137	AntIn
139	AntIn
139	AntIn
140	AntOut
141	AntIn
141	AntIn
143	AntOut
144	AntOut
148	AntIn
148	AntIn
149	AntIn
149	AntIn
149	AntIn
150	AntIn
151	AntOut
153	AntIn
154	AntIn
155	AntIn
155	AntIn
156	AntOut
158	AntOut
159	AntIn
160	AntIn
160	AntIn
162	AntIn
163	AntOut
165	AntOut
167	AntIn
168	AntOut
169	AntIn
173	AntIn
174	AntIn
174	AntOut
175	AntIn
176	AntIn
176	AntIn
178	AntIn
179	AntOut
180	AntOut
181	AntIn

182	AntIn
182	AntIn
183	AntOut
183	AntOut
185	AntOut
186	AntOut
188	AntIn
189	AntOut
190	AntIn
190	AntIn
191	AntIn
191	AntIn
192	AntIn
192	AntIn
193	AntIn
194	AntOut
195	AntIn
195	AntIn
195	AntIn
197	AntIn
197	AntIn
198	AntIn
198	AntOut
199	AntOut
200	AntOut
201	AntIn
201	AntIn
202	AntOut
205	AntOut
206	AntIn
206	AntOut
207	AntIn
209	AntIn
209	AntIn
210	AntIn
210	AntIn
211	AntOut
211	AntOut
213	AntIn
213	AntIn
214	AntIn
214	AntOut

214	AntOut
215	AntOut
216	AntIn
216	AntIn
216	AntIn
217	AntIn
217	AntIn
218	AntIn
220	AntIn
220	AntOut
222	AntIn
222	AntIn
224	AntIn
224	AntIn
225	AntIn
226	AntIn
227	AntOut
228	AntIn
229	AntIn
229	AntIn
230	AntIn
231	AntIn
232	AntOut
234	AntIn
234	AntIn
234	AntIn
235	AntIn
236	AntIn
238	AntIn
238	AntOut
238	AntOut
239	AntOut
239	AntOut
239	AntOut
241	AntIn
242	AntIn
245	AntIn
247	AntOut
247	AntOut
248	AntOut
250	AntIn
250	AntIn

251 AntIn
252 AntIn
253 AntIn
253 AntIn
254 AntIn
255 AntOut
255 AntOut
257 AntIn
258 AntIn
258 AntIn
258 AntIn
258 AntIn
260 AntIn
260 AntIn
261 AntIn
262 AntOut
262 AntOut
263 AntOut
264 AntIn
265 AntIn
265 AntIn
266 AntIn
267 AntIn
267 AntIn
268 AntIn
269 AntOut
270 AntIn
271 AntIn
273 AntOut
274 AntIn
275 AntOut
276 AntOut
278 AntIn
279 AntIn
281 AntOut
282 AntOut
283 AntIn
283 AntOut
283 AntOut
285 AntIn
286 AntOut
287 AntIn

288	AntOut
289	AntIn
292	AntIn
292	AntIn
292	AntIn
292	AntOut
296	AntIn
296	AntIn
299	AntIn
300	AntIn
300	AntOut
302	AntIn
302	AntOut
303	AntIn
305	AntOut
307	AntOut
308	AntIn
308	AntIn
311	AntIn
312	AntIn
314	AntIn
315	AntIn
315	AntIn
316	AntIn
317	AntOut
317	AntOut
318	AntIn
320	AntIn
321	AntIn
321	AntIn
321	AntIn
321	AntIn
322	AntIn
325	AntOut
328	AntOut
329	AntIn
329	AntIn
331	AntIn
332	AntOut
336	AntIn
337	AntIn
337	AntOut

339 AntOut
341 AntIn
342 AntIn
343 AntIn
343 AntOut
345 AntIn
346 AntIn
346 AntIn
347 AntIn
349 AntOut
351 AntIn
355 AntOut
355 AntOut
357 AntIn
357 AntIn
358 AntIn
358 AntIn
358 AntIn
359 AntIn
359 AntIn
359 AntIn
365 AntOut
366 AntIn
366 AntIn
367 AntIn
369 AntIn
369 AntIn
370 AntIn
370 AntOut
371 AntIn
372 AntIn
372 AntIn
373 AntIn
373 AntIn
374 AntOut
375 AntIn
375 AntOut
376 AntIn
376 AntIn
378 AntIn
378 AntIn
379 AntIn

385	AntIn
385	AntIn
385	AntIn
386	AntIn
387	AntIn
387	AntIn
390	AntOut
391	AntIn
391	AntIn
393	AntIn
393	AntOut
395	AntIn
399	AntIn
399	AntIn
400	AntIn
403	AntIn
405	AntIn
405	AntIn
406	AntOut
406	AntOut
407	AntIn
409	AntIn
410	AntIn
410	AntIn
410	AntIn
411	AntIn
411	AntOut
412	AntIn
416	AntIn
416	AntIn
417	AntOut
419	AntIn
422	AntIn
422	AntIn
424	AntOut
429	AntIn
429	AntIn
431	AntIn
432	AntIn
434	AntIn
434	AntOut
437	AntIn

438	AntIn
438	AntIn
438	AntIn
441	AntOut
442	AntOut
443	AntOut
444	AntOut
447	AntOut
448	AntIn
450	AntIn
450	AntIn
451	AntIn
451	AntIn
456	AntIn
456	AntIn
457	AntIn
457	AntIn
460	AntIn
460	AntOut
466	AntIn
466	AntIn
467	AntIn
471	AntOut
472	AntIn
475	AntIn
476	AntIn
481	AntIn
481	AntIn
492	AntIn
504	AntIn
527	AntOut
565	AntOut
566	AntIn
568	AntIn
635	AntOut
636	AntIn
652	AntOut
654	AntIn
670	AntIn
672	AntIn
679	AntIn
695	AntIn

715	AntIn
719	AntIn
723	AntIn
724	AntIn
740	AntIn
745	AntIn
751	AntIn
757	AntIn
766	AntIn
772	AntIn
773	AntIn
780	AntIn
792	AntIn
800	AntOut
802	AntIn
810	AntIn
822	AntIn
846	AntIn
864	AntIn
864	AntIn
890	AntIn
905	AntIn
912	AntIn
912	AntIn
929	AntIn
941	AntIn
942	AntIn
953	AntIn
960	AntIn
963	AntIn
970	AntIn
973	AntIn
994	AntIn
1003	AntIn
1024	AntIn
1027	AntIn
1029	AntIn
1042	AntIn
1042	AntIn
1045	AntIn
1053	AntIn
1067	AntIn

1068	AntIn
1090	AntIn
1090	AntIn
1103	AntIn
1114	AntIn
1118	AntIn
1130	AntIn
1136	AntIn
1151	AntIn
1174	AntIn
1179	AntIn
1200	AntIn
1242	AntIn
1261	AntIn
1261	AntIn
1263	AntIn
1297	AntIn
1310	AntIn
1313	AntIn
1318	AntIn
1331	AntIn
1334	AntIn
1336	AntIn
1354	AntIn
1355	AntIn
1358	AntIn
1406	AntIn
1410	AntIn
1410	AntOut
1413	AntIn
1443	AntIn
1445	AntIn
1460	AntIn
1473	AntIn
1503	AntIn
1506	AntOut
1511	AntIn
1562	AntIn
1567	AntIn
1588	AntIn
1595	AntIn
1596	AntIn

1623	AntIn
1645	AntOut
1648	AntIn
1675	AntIn
1684	AntIn
1715	AntOut
1805	AntIn
1810	AntIn
1826	AntIn
1848	AntIn
1864	AntIn
1871	AntIn
1927	AntIn
1953	AntIn
1987	AntIn
1987	AntIn
2008	AntIn
2018	AntIn
2044	AntIn
2071	AntIn
2214	AntIn
2214	AntIn
2273	AntIn
2276	AntIn
2307	AntIn
2319	AntIn
2367	AntIn
2373	AntIn
2394	AntIn
2418	AntOut
2419	AntIn
2473	AntIn
2589	AntIn
2601	AntIn
2687	AntIn
