# Supplementary material for: Interactions Increase Forager Availability and Activity in Harvester Ants
Source: PLoS One. 2015 Nov 5;10(11):e0141971. doi: 10.1371/journal.pone.0141971 (PMC4635008; doi:10.1371/journal.pone.0141971)
Supplement: S3 Dataset — We observed and filmed behavior inside the nest during and after forager removals. This dataset shows our counts made from the films of the numbers of returning and outgoing foragers at the nest entrance and the number of ascending and descending ants at all tunnel entrances. (ZIP) [file pone.0141971.s004.zip › S3 Dataset/2013 Correlation Data 367 8-21.pdf]

**Researcher**	**Jovel Queirolo**
**Colony 367**	
**8/21/13**	
**Video time (seconds)**	**Event**
2	Ascend
2	Ascend
3	Ascend
3	Ascend
4	Ascend
4	Ascend
5	Ascend
5	Ascend
6	Ascend
6	Descend
7	Descend
7	Descend
8	Descend
9	Descend
9	Descend
10	Descend
10	Descend
10	Descend
10	Descend
11	Descend
11	Descend
11	Descend
12	Descend
12	Descend
12	Descend
13	Ascend
13	Ascend
13	Ascend
14	Ascend
14	Ascend
15	Ascend
15	Descend
16	Descend
17	Ascend
17	Ascend
17	Ascend
18	Ascend

18	Ascend
19	Ascend
19	Ascend
21	Ascend
21	Descend
22	Descend
22	Descend
23	Descend
24	Descend
25	Descend
27	Ascend
28	Ascend
28	Ascend
28	Ascend
29	Ascend
30	Ascend
30	Descend
30	Descend
31	Descend
31	Descend
32	Descend
32	Descend
32	Descend
32	Descend
33	Descend
33	Ascend
33	Ascend
34	Ascend
34	Ascend
35	Ascend
35	Descend
35	Descend
37	Descend
37	Descend
37	Descend
38	Descend
38	Descend
38	Descend
39	Descend
40	Ascend
40	Ascend
41	Ascend

41	Ascend
41	Ascend
47	Ascend
47	Ascend
47	Ascend
47	Ascend
48	Ascend
48	Ascend
48	Ascend
48	Ascend
49	Ascend
49	Ascend
50	Ascend
50	Ascend
50	Ascend
51	Ascend
51	Ascend
51	Ascend
52	Ascend
52	Ascend
52	Ascend
53	Descend
53	Descend
53	Descend
53	Ascend
54	Ascend
54	Ascend
54	Ascend
55	Descend
55	Descend
55	Descend
56	Ascend
56	Ascend
56	Ascend
56	Descend
57	Descend
57	Descend
57	Descend
57	Descend
58	Ascend
58	Ascend
58	Ascend

58	Descend
59	Descend
59	Descend
59	Descend
59	Descend
60	Ascend
60	Ascend
60	Descend
60	Descend
61	Descend
62	Descend
62	Descend
62	Descend
62	Descend
63	Descend
63	Descend
63	Ascend
63	Ascend
64	Ascend
64	Ascend
64	Ascend
65	Ascend
65	Descend
65	Ascend
66	Ascend
66	Ascend
66	Ascend
67	Descend
67	Ascend
68	Descend
69	Descend
69	Ascend
70	Ascend
70	Ascend
70	Descend
71	Descend
71	Descend
71	Descend
72	Descend
72	Descend
72	Descend
73	Descend

73	Descend
73	Descend
73	Ascend
74	Ascend
74	Ascend
74	Ascend
74	Ascend
75	Ascend
75	Ascend
75	Ascend
75	Ascend
76	Ascend
76	Ascend
76	Ascend
77	Ascend
77	Ascend
77	Ascend
78	Ascend
78	Ascend
79	Ascend
79	Ascend
79	Ascend
80	Ascend
80	Descend
80	Descend
81	Descend
81	Descend
81	Descend
82	Descend
82	Descend
83	Ascend
84	Ascend
84	Ascend
84	Ascend
84	Ascend
85	Ascend
85	Ascend
85	Descend
86	Descend
86	Descend
86	Descend
87	Descend

87	Descend
88	Descend
88	Ascend
88	Ascend
90	Ascend
90	Ascend
90	Descend
91	Descend
91	Ascend
91	Ascend
92	Ascend
92	Ascend
92	Ascend
92	Ascend
93	Descend
93	Descend
94	Descend
94	Descend
94	Descend
95	Ascend
95	Ascend
96	Descend
97	Descend
97	Ascend
97	Ascend
97	Descend
98	Ascend
98	Descend
99	Ascend
99	Ascend
101	Descend
102	Ascend
103	Ascend
103	Descend
104	Ascend
105	Descend
105	Ascend
105	Ascend
105	Ascend
106	Ascend
106	Ascend
106	Ascend

107	Ascend
107	Ascend
107	Ascend
107	Ascend
108	Ascend
108	Ascend
108	Ascend
108	Ascend
109	Ascend
109	Ascend
109	Ascend
109	Ascend
110	Ascend
111	Ascend
112	Descend
112	Descend
112	Descend
113	Descend
113	Descend
113	Ascend
114	Ascend
114	Ascend
114	Ascend
115	Ascend
115	Ascend
115	Ascend
115	Descend
116	Descend
116	Descend
116	Descend
116	Descend
116	Descend
117	Descend
117	Ascend
117	Ascend
117	Descend
118	Descend
118	Ascend
118	Ascend
118	Ascend
119	Ascend
119	Ascend

119	Ascend
120	Ascend
120	Descend
120	Descend
120	Descend
121	Descend
121	Descend
121	Descend
122	Descend
122	Descend
122	Descend
123	Descend
124	Ascend
124	Ascend
124	Ascend
125	Ascend
125	Ascend
126	Descend
127	Descend
127	Descend
128	Ascend
129	Descend
130	Ascend
130	Ascend
131	Ascend
131	Descend
131	Descend
132	Ascend
132	Ascend
132	Ascend
133	Ascend
133	Ascend
133	Ascend
134	Ascend
134	Ascend
134	Descend
135	Descend
135	Descend
135	Ascend
136	Ascend
137	Descend
137	Ascend

137	Ascend
138	Ascend
140	Descend
140	Ascend
141	Ascend
141	Ascend
142	Ascend
142	Ascend
143	Descend
143	Descend
143	Ascend
144	Ascend
144	Ascend
145	Ascend
145	Ascend
145	Ascend
145	Ascend
146	Ascend
146	Ascend
147	Descend
147	Descend
147	Descend
147	Ascend
148	Ascend
148	Ascend
149	Ascend
149	Descend
149	Descend
150	Ascend
150	Descend
151	Ascend
152	Descend
154	Descend
154	Descend
155	Ascend
155	Ascend
155	Ascend
156	Ascend
156	Ascend
156	Ascend
157	Ascend
158	Ascend

159	Descend
160	Descend
160	Ascend
161	Descend
161	Descend
162	Descend
162	Descend
162	Ascend
162	Descend
163	Descend
163	Descend
163	Descend
164	Descend
164	Descend
165	Descend
165	Ascend
166	Descend
167	Ascend
168	Ascend
168	Descend
168	Descend
169	Descend
169	Descend
169	Descend
170	Descend
170	Descend
171	Descend
171	Descend
171	Ascend
172	Ascend
172	Ascend
172	Ascend
172	Ascend
173	Ascend
173	Ascend
173	Ascend
174	Ascend
174	Ascend
175	Descend
175	Descend
175	Descend
176	Descend

176	Descend
177	Ascend
177	Descend
177	Descend
178	Ascend
178	Ascend
178	Ascend
179	Ascend
179	Ascend
180	Ascend
180	Ascend
181	Ascend
181	Ascend
181	Ascend
181	Descend
182	Descend
183	Descend
183	Ascend
184	Descend
184	Ascend
184	Ascend
184	Descend
185	Descend
186	Descend
186	Descend
187	Descend
188	Descend
188	Descend
189	Descend
189	Descend
190	Ascend
190	Ascend
190	Ascend
191	Descend
191	Descend
192	Descend
192	Descend
192	Descend
193	Descend
193	Descend
194	Descend
194	Ascend

194	Ascend
194	Ascend
196	Descend
196	Descend
196	Descend
197	Descend
198	Descend
198	Ascend
199	Ascend
199	Ascend
199	Ascend
200	Descend
201	Descend
202	Descend
202	Descend
204	Descend
204	Ascend
205	Ascend
206	Descend
207	Descend
207	Ascend
207	Ascend
207	Ascend
208	Ascend
208	Ascend
208	Ascend
208	Ascend
209	Ascend
209	Ascend
210	Ascend
210	Ascend
210	Ascend
211	Ascend
211	Ascend
212	Ascend
212	Ascend
212	Ascend
212	Ascend
213	Ascend
213	Ascend
214	Ascend
214	Descend

214	Descend
214	Descend
215	Descend
215	Descend
215	Descend
215	Descend
216	Descend
216	Descend
216	Ascend
217	Ascend
217	Descend
217	Descend
218	Descend
218	Ascend
218	Ascend
218	Descend
219	Descend
219	Ascend
219	Ascend
219	Ascend
220	Ascend
220	Ascend
220	Ascend
220	Ascend
221	Ascend
221	Ascend
221	Ascend
221	Ascend
222	Ascend
222	Descend
222	Descend
223	Ascend
223	Ascend
223	Ascend
223	Ascend
223	Ascend
224	Ascend
225	Ascend
225	Descend
225	Descend
226	Ascend
226	Ascend

226	Ascend
227	Descend
227	Descend
227	Descend
227	Descend
227	Descend
228	Descend
228	Ascend
228	Ascend
228	Ascend
229	Descend
229	Descend
229	Ascend
229	Ascend
230	Ascend
230	Ascend
230	Ascend
230	Ascend
231	Ascend
231	Ascend
231	Ascend
231	Ascend
231	Descend
232	Descend
232	Descend
232	Descend
233	Descend
233	Ascend
233	Ascend
233	Descend
234	Descend
234	Ascend
234	Ascend
234	Descend
235	Descend
235	Descend
235	Descend
236	Descend
237	Descend
237	Descend
238	Descend
238	Descend

239	Descend
239	Descend
239	Descend
239	Descend
240	Descend
240	Descend
240	Descend
241	Descend
241	Descend
242	Descend
243	Ascend
243	Ascend
244	Ascend
244	Ascend
245	Ascend
245	Ascend
245	Ascend
246	Ascend
248	Descend
248	Descend
249	Descend
249	Descend
249	Descend
250	Ascend
250	Ascend
250	Ascend
250	Ascend
251	Ascend
251	Ascend
251	Ascend
252	Descend
252	Descend
252	Descend
253	Ascend
253	Ascend
254	Descend
255	Descend
257	Descend
257	Descend
259	Descend
259	Descend
261	Descend

261	Descend
261	Descend
263	Descend
263	Descend
263	Descend
263	Descend
264	Descend
264	Descend
264	Descend
265	Descend
265	Descend
266	Descend
266	Descend
267	Descend
267	Descend
268	Ascend
268	Ascend
268	Ascend
268	Ascend
269	Descend
269	Descend
269	Ascend
269	Ascend
270	Ascend
270	Descend
271	Ascend
271	Ascend
271	Ascend
272	Descend
273	Descend
273	Ascend
274	Ascend
274	Ascend
274	Ascend
275	Ascend
275	Ascend
276	Ascend
277	Ascend
277	Ascend
277	Ascend
277	Ascend
278	Ascend

278	Ascend
278	Ascend
278	Ascend
278	Ascend
278	Ascend
279	Ascend
279	Ascend
279	Ascend
279	Ascend
280	Ascend
280	Ascend
281	Ascend
282	Ascend
283	Ascend
284	Ascend
284	Ascend
287	Ascend
288	Descend
288	Descend
289	Descend
290	Descend
290	Descend
291	Descend
291	Descend
291	Descend
292	Descend
292	Descend
292	Descend
293	Descend
293	Descend
293	Descend
294	Descend
294	Descend
294	Ascend
295	Ascend
295	Ascend
295	Ascend
297	Descend
297	Descend
297	Descend
297	Descend
297	Ascend

298	Ascend
298	Ascend
298	Ascend
298	Ascend
299	Ascend
299	Ascend
299	Ascend
299	Ascend
300	Ascend
300	Ascend
300	Ascend
301	Ascend
301	Ascend
301	Ascend
301	Ascend
302	Ascend
302	Ascend
302	Ascend
303	Ascend
306	Ascend
306	Ascend
307	Ascend
307	Ascend
307	Descend
308	Ascend
308	Ascend
310	Ascend
310	Ascend
311	Ascend
311	Ascend
312	Ascend
312	Ascend
312	Descend
312	Descend
313	Descend
313	Descend
315	Descend
315	Descend
316	Descend
316	Descend
316	Descend
316	Descend

318	Ascend
318	Ascend
319	Ascend
319	Ascend
319	Ascend
320	Ascend
321	Ascend
321	Ascend
321	Ascend
321	Ascend
322	Ascend
322	Ascend
322	Ascend
322	Ascend
323	Descend
323	Descend
323	Descend
324	Ascend
324	Ascend
325	Ascend
325	Ascend
326	Ascend
326	Ascend
326	Ascend
326	Ascend
327	Ascend
327	Ascend
327	Ascend
328	Ascend
328	Ascend
329	Descend
330	Descend
330	Descend
330	Descend
331	Descend
331	Descend
331	Descend
332	Ascend
332	Ascend
332	Ascend
333	Ascend
333	Ascend

333	Ascend
334	Descend
334	Descend
334	Descend
334	Descend
335	Descend
335	Ascend
336	Ascend
336	Ascend
336	Ascend
336	Ascend
337	Descend
337	Descend
337	Descend
337	Descend
338	Ascend
338	Ascend
338	Ascend
339	Descend
339	Descend
339	Descend
339	Ascend
340	Ascend
340	Ascend
340	Ascend
340	Ascend
340	Ascend
341	Ascend
341	Ascend
342	Ascend
344	Ascend
344	Ascend
345	Ascend
345	Ascend
346	Descend
347	Ascend
347	Ascend
348	Ascend
348	Ascend
348	Ascend
348	Ascend
349	Ascend

349	Ascend
349	Ascend
349	Ascend
349	Ascend
350	Ascend
350	Ascend
350	Ascend
351	Ascend
351	Ascend
352	Ascend
352	Ascend
353	Ascend
354	Descend
354	Descend
355	Descend
356	Descend
357	Descend
358	Descend
359	Descend
363	Descend
363	Descend
363	Descend
364	Descend
364	Descend
366	Descend
366	Ascend
366	Ascend
366	Ascend
367	Ascend
367	Ascend
367	Ascend
368	Ascend
368	Ascend
368	Ascend
368	Ascend
369	Ascend
369	Ascend
369	Ascend
369	Ascend
370	Ascend
370	Ascend
372	Ascend

372	Ascend
372	Ascend
373	Ascend
373	Ascend
373	Ascend
374	Ascend
374	Ascend
375	Ascend
375	Ascend
375	Ascend
376	Ascend
377	Ascend
377	Ascend
378	Ascend
379	Descend
379	Descend
379	Descend
380	Descend
381	Descend
381	Descend
381	Ascend
381	Ascend
382	Ascend
382	Ascend
382	Ascend
383	Ascend
384	Ascend
384	Ascend
385	Ascend
385	Ascend
386	Ascend
386	Ascend
386	Ascend
386	Ascend
387	Ascend
387	Ascend
388	Ascend
389	Ascend
389	Descend
389	Descend
389	Descend
390	Descend

390	Descend
392	Descend
392	Ascend
392	Ascend
392	Ascend
393	Ascend
393	Ascend
394	Ascend
394	Ascend
394	Ascend
395	Ascend
395	Ascend
395	Descend
396	Descend
396	Descend
397	Descend
397	Descend
398	Descend
398	Descend
399	Descend
399	Descend
399	Descend
400	Descend
402	Descend
403	Descend
403	Ascend
403	Ascend
404	Ascend
405	Descend
405	Descend
405	Descend
406	Ascend
407	Descend
408	Ascend
408	Ascend
409	Ascend
409	Ascend
410	Ascend
410	Ascend
410	Ascend
411	Ascend
411	Ascend

412	Ascend
412	Ascend
412	Ascend
412	Ascend
412	Descend
413	Descend
413	Descend
413	Descend
414	Descend
414	Descend
414	Descend
414	Ascend
415	Ascend
415	Ascend
415	Ascend
415	Ascend
416	Ascend
416	Ascend
416	Descend
417	Descend
417	Descend
418	Descend
418	Ascend
418	Ascend
419	Ascend
419	Descend
419	Descend
420	Descend
420	Descend
420	Descend
421	Descend
421	Descend
421	Descend
421	Descend
422	Descend
422	Descend
422	Descend
422	Descend
423	Descend
423	Descend
423	Descend
423	Descend

424	Descend
424	Descend
424	Descend
424	Descend
425	Descend
426	Descend
426	Descend
426	Descend
428	Ascend
428	Ascend
428	Ascend
430	Ascend
430	Ascend
430	Descend
430	Descend
432	Descend
432	Descend
432	Descend
432	Descend
433	Descend
433	Descend
433	Descend
433	Descend
434	Descend
435	Descend
435	Descend
436	Descend
436	Descend
436	Descend
436	Descend
437	Descend
438	Descend
439	Ascend
440	Ascend
440	Ascend
441	Ascend
441	Ascend
442	Ascend
442	Ascend
442	Ascend
442	Ascend
443	Ascend

443	Ascend
444	Descend
444	Descend
445	Descend
445	Descend
445	Descend
446	Descend
446	Descend
446	Descend
447	Descend
447	Descend
448	Descend
448	Descend
448	Descend
451	Descend
451	Descend
451	Descend
452	Ascend
453	Ascend
453	Descend
454	Ascend
454	Ascend
458	Descend
459	Descend
461	Descend
461	Descend
462	Descend
462	Descend
462	Descend
463	Descend
464	Descend
464	Descend
465	Descend
465	Descend
465	Ascend
466	Ascend
466	Ascend
466	Ascend
466	Ascend
467	Ascend
467	Ascend
468	Ascend

468	Ascend
468	Ascend
468	Ascend
469	Ascend
470	Ascend
471	Ascend
471	Ascend
472	Ascend
472	Ascend
472	Ascend
473	Ascend
473	Ascend
473	Ascend
474	Descend
474	Descend
475	Descend
475	Descend
476	Descend
477	Descend
477	Descend
477	Descend
479	Ascend
481	Descend
482	Descend
482	Descend
482	Descend
484	Descend
487	Descend
489	Descend
489	Ascend
489	Ascend
490	Ascend
490	Ascend
490	Ascend
490	Ascend
491	Ascend
491	Ascend
492	Ascend
492	Ascend
492	Descend
493	Descend
494	Descend

494	Descend
494	Ascend
494	Ascend
495	Descend
495	Descend
495	Descend
496	Descend
497	Descend
498	Descend
499	Ascend
499	Ascend
500	Descend
500	Descend
501	Descend
501	Descend
502	Descend
503	Ascend
503	Ascend
504	Ascend
506	Ascend
506	Descend
507	Descend
508	Descend
508	Ascend
509	Descend
509	Descend
509	Descend
510	Descend
510	Descend
510	Ascend
511	Ascend
511	Ascend
511	Ascend
512	Ascend
512	Ascend
512	Ascend
512	Ascend
513	Ascend
513	Ascend
513	Descend
513	Descend
514	Descend

514	Descend
515	Descend
515	Descend
516	Descend
517	Descend
517	Descend
517	Ascend
518	Descend
518	Descend
519	Descend
520	Descend
520	Descend
521	Ascend
521	Ascend
521	Descend
522	Descend
523	Descend
523	Descend
523	Descend
524	Descend
524	Descend
525	Descend
525	Descend
525	Descend
526	Descend
526	Descend
530	Ascend
530	Ascend
530	Ascend
531	Ascend
531	Ascend
531	Ascend
532	Ascend
532	Ascend
534	Ascend
534	Ascend
535	Ascend
535	Ascend
535	Ascend
536	Ascend
536	Ascend
536	Ascend

537	Ascend
538	Descend
539	Descend
539	Descend
540	Descend
540	Descend
541	Descend
542	Descend
542	Descend
542	Descend
543	Descend
543	Descend
544	Descend
544	Descend
547	Ascend
547	Descend
549	Descend
550	Descend
551	Descend
551	Descend
552	Descend
553	Descend
553	Descend
556	Descend
557	Descend
560	Descend
563	Descend
563	Descend
563	Descend
565	Descend
565	Descend
566	Descend
568	Descend
568	Descend
569	Descend
569	Descend
569	Descend
570	Descend
570	Descend
570	Descend
571	Descend
571	Descend

571	Descend
572	Descend
572	Descend
573	Descend
573	Descend
573	Descend
574	Descend
575	Descend
577	Descend
578	Descend
580	Descend
586	Descend
589	Descend
589	Descend
592	Descend
593	Descend
595	Descend
604	Descend
605	Ascend
606	Descend
609	Descend
612	Ascend
613	Ascend
614	Descend
616	Ascend
622	Descend
629	Descend
632	Descend
639	Descend
642	Descend
648	Descend
663	Descend
664	Descend
664	Descend
664	Descend
664	Descend
665	Descend
665	Descend
665	Descend
666	Descend
666	Ascend
666	Ascend

668	Ascend
669	Descend
669	Descend
670	Descend
670	Descend
671	Descend
672	Descend
672	Descend
673	Descend
674	Ascend
675	Ascend
676	Descend
680	Descend
680	Ascend
680	Ascend
681	Descend
682	Descend
682	Descend
682	Descend
683	Descend
684	Descend
685	Descend
686	Ascend
687	Ascend
689	Descend
690	Descend
691	Descend
691	Descend
692	Descend
692	Ascend
693	Ascend
695	Descend
696	Descend
699	Descend
700	Descend
703	Ascend
705	Descend
707	Descend
710	Ascend
712	Descend
713	Ascend
718	Ascend

719	Descend
724	Ascend
727	Descend
728	Descend
729	Descend
729	Descend
732	Ascend
733	Descend
735	Descend
735	Descend
736	Descend
737	Descend
737	Descend
737	Descend
741	Descend
741	Ascend
744	Ascend
747	Descend
749	Descend
749	Descend
750	Descend
750	Descend
750	Descend
750	Descend
751	Ascend
752	Ascend
753	Descend
754	Descend
754	Descend
754	Descend
755	Descend
755	Descend
756	Descend
756	Descend
756	Descend
757	Descend
758	Ascend
760	Descend
761	Descend
761	Descend
764	Descend
764	Descend

765	Descend
765	Descend
765	Descend
766	Descend
766	Descend
766	Descend
767	Ascend
768	Ascend
769	Descend
770	Ascend
771	Descend
772	Descend
775	Descend
775	Descend
775	Descend
776	Descend
776	Descend
776	Descend
780	Descend
780	Descend
782	Descend
786	Descend
788	Ascend
792	Descend
793	Descend
794	Descend
794	Descend
794	Descend
796	Descend
797	Descend
798	Descend
798	Descend
800	Descend
805	Descend
805	Ascend
806	Descend
808	Descend
808	Descend
811	Descend
811	Descend
812	Ascend
814	Descend

814	Descend
815	Descend
816	Descend
817	Ascend
825	Descend
833	Descend
834	Descend
834	Descend
835	Descend
835	Descend
836	Descend
839	Ascend
841	Descend
842	Descend
843	Descend
843	Descend
845	Descend
851	Descend
855	Descend
856	Descend
859	Descend
860	Ascend
862	Descend
862	Descend
862	Ascend
864	Descend
864	Descend
864	Descend
865	Descend
866	Ascend
869	Ascend
871	Descend
872	Descend
873	Descend
873	Descend
875	Descend
875	Descend
876	Descend
878	Descend
881	Ascend
884	Ascend
886	Descend

889	Descend
891	Ascend
894	Descend
898	Descend
901	Descend
901	Descend
902	Descend
902	Ascend
905	Descend
907	Descend
909	Ascend
913	Descend
914	Descend
915	Descend
916	Ascend
922	Ascend
923	Ascend
924	Descend
924	Descend
925	Descend
925	Descend
925	Descend
928	Descend
930	Descend
930	Ascend
936	Descend
939	Descend
940	Ascend
941	Descend
944	Descend
945	Ascend
947	Descend
949	Descend
953	Ascend
955	Ascend
958	Descend
958	Descend
958	Descend
958	Descend
959	Ascend
959	Ascend
960	Ascend

961	Descend
962	Descend
962	Descend
963	Descend
965	Descend
966	Descend
967	Ascend
968	Ascend
976	Descend
977	Descend
977	Descend
979	Descend
981	Descend
983	Descend
983	Ascend
983	Descend
983	Descend
983	Descend
984	Descend
984	Descend
985	Ascend
985	Ascend
985	Ascend
986	Ascend
986	Ascend
987	Ascend
987	Ascend
988	Ascend
989	Ascend
989	Ascend
989	Ascend
990	Ascend
990	Ascend
991	Ascend
991	Ascend
991	Ascend
992	Ascend
993	Ascend
993	Descend
993	Descend
994	Ascend
995	Ascend

995	Ascend
995	Ascend
996	Ascend
996	Ascend
996	Ascend
997	Ascend
997	Ascend
997	Ascend
999	Ascend
999	Ascend
999	Ascend
1000	Ascend
1000	Ascend
1000	Ascend
1001	Ascend
1001	Ascend
1001	Descend
1001	Descend
1002	Descend
1002	Descend
1002	Ascend
1002	Ascend
1002	Ascend
1003	Ascend
1003	Ascend
1003	Ascend
1003	Ascend
1004	Ascend
1004	Ascend
1004	Ascend
1005	Ascend
1007	Descend
1008	Descend
1008	Descend
1008	Descend
1009	Descend
1011	Ascend
1011	Ascend
1012	Ascend
1013	Ascend
1013	Ascend
1014	Ascend

1014	Ascend
1015	Ascend
1015	Ascend
1015	Ascend
1016	Ascend
1018	Ascend
1019	Descend
1020	Descend
1020	Ascend
1020	Ascend
1020	Ascend
1021	Ascend
1021	Ascend
1021	Ascend
1022	Ascend
1022	Ascend
1023	Ascend
1023	Ascend
1025	Ascend
1025	Ascend
1026	Ascend
1026	Ascend
1027	Ascend
1027	Ascend
1028	Ascend
1029	Ascend
1029	Ascend
1029	Ascend
1030	Ascend
1031	Descend
1032	Descend
1033	Descend
1033	Ascend
1033	Ascend
1034	Descend
1036	Ascend
1036	Ascend
1036	Ascend
1036	Descend
1037	Descend
1037	Descend
1038	Ascend

1038	Ascend
1039	Ascend
1039	Ascend
1040	Ascend
1040	Ascend
1041	Ascend
1041	Ascend
1043	Ascend
1043	Ascend
1044	Ascend
1044	Ascend
1045	Ascend
1045	Ascend
1045	Ascend
1046	Ascend
1047	Ascend
1047	Ascend
1047	Ascend
1048	Ascend
1049	Descend
1049	Descend
1049	Descend
1050	Descend
1050	Ascend
1050	Ascend
1051	Ascend
1051	Ascend
1051	Ascend
1052	Ascend
1052	Ascend
1053	Ascend
1054	Ascend
1054	Ascend
1054	Ascend
1055	Ascend
1056	Ascend
1056	Ascend
1057	Ascend
1057	Ascend
1059	Descend
1060	Descend
1063	Descend

1063	Descend
1064	Descend
1064	Descend
1065	Ascend
1065	Ascend
1066	Ascend
1066	Ascend
1067	Descend
1067	Descend
1067	Descend
1068	Descend
1068	Descend
1068	Ascend
1069	Ascend
1069	Ascend
1070	Descend
1070	Descend
1071	Descend
1071	Descend
1072	Ascend
1072	Ascend
1073	Ascend
1073	Ascend
1073	Ascend
1074	Ascend
1074	Ascend
1075	Ascend
1075	Ascend
1075	Ascend
1076	Ascend
1077	Ascend
1078	Ascend
1078	Ascend
1078	Ascend
1079	Ascend
1079	Ascend
1080	Ascend
1081	Ascend
1083	Descend
1085	Descend
1086	Descend
1086	Descend

1087	Descend
1087	Descend
1088	Ascend
1088	Descend
1089	Descend
1089	Descend
1092	Descend
1092	Descend
1094	Descend
1096	Descend
1096	Descend
1098	Ascend
1098	Ascend
1098	Ascend
1099	Ascend
1099	Ascend
1099	Ascend
1101	Ascend
1101	Ascend
1101	Ascend
1102	Ascend
1103	Ascend
1103	Ascend
1103	Descend
1103	Descend
1104	Ascend
1104	Ascend
1104	Ascend
1104	Ascend
1105	Ascend
1105	Ascend
1107	Ascend
1107	Ascend
1109	Descend
1110	Descend
1110	Descend
1111	Descend
1112	Descend
1113	Descend
1114	Descend
1115	Descend
1116	Descend

1117	Descend
1117	Descend
1117	Descend
1117	Descend
1118	Descend
1118	Descend
1118	Descend
1119	Descend
1119	Descend
1119	Descend
1120	Ascend
1120	Ascend
1120	Ascend
1121	Ascend
1121	Ascend
1121	Ascend
1122	Ascend
1122	Ascend
1122	Ascend
1123	Ascend
1123	Ascend
1123	Ascend
1124	Ascend
1124	Ascend
1124	Ascend
1125	Ascend
1125	Ascend
1126	Ascend
1126	Ascend
1126	Ascend
1126	Ascend
1126	Ascend
1127	Descend
1127	Descend
1127	Descend
1128	Descend
1128	Descend
1129	Descend
1129	Descend
1129	Descend
1130	Descend
1130	Ascend

1130	Ascend
1130	Ascend
1130	Ascend
1130	Ascend
1131	Ascend
1131	Ascend
1132	Ascend
1132	Ascend
1133	Ascend
1133	Ascend
1134	Ascend
1134	Ascend
1136	Ascend
1138	Descend
1139	Descend
1139	Ascend
1139	Descend
1140	Descend
1140	Ascend
1140	Ascend
1141	Ascend
1142	Ascend
1142	Ascend
1143	Ascend
1143	Ascend
1144	Ascend
1144	Ascend
1144	Ascend
1145	Ascend
1145	Ascend
1145	Ascend
1145	Ascend
1147	Ascend
1148	Ascend
1154	Descend
1156	Descend
1156	Descend
1159	Ascend
1164	Descend
1168	Ascend
1169	Descend
1170	Descend

1171	Descend
1172	Ascend
1175	Ascend
1175	Ascend
1175	Ascend
1176	Ascend
1176	Ascend
1177	Ascend
1179	Ascend
1180	Ascend
1180	Ascend
1181	Ascend
1181	Ascend
1181	Ascend
1181	Ascend
1182	Ascend
1182	Ascend
1182	Ascend
1183	Ascend
1183	Ascend
1183	Ascend
1184	Ascend
1184	Descend
1184	Descend
1185	Descend
1185	Descend
1185	Descend
1186	Descend
1186	Descend
1187	Descend
1189	Descend
1190	Descend
1192	Descend
1192	Ascend
1192	Ascend
1193	Ascend
1193	Ascend
1193	Ascend
1193	Ascend
1194	Ascend
1194	Ascend
1194	Ascend

1195	Ascend
1195	Ascend
1195	Ascend
1196	Ascend
1197	Ascend
1197	Ascend
1197	Descend
1198	Descend
1199	Descend
1199	Descend
1199	Descend
1199	Descend
1200	Descend
1200	Descend
1201	Descend
1201	Descend
1201	Descend
1201	Ascend
1201	Ascend
1202	Ascend
1202	Ascend
1202	Ascend
1204	Ascend
1204	Ascend
1205	Ascend
1205	Ascend
1205	Ascend
1205	Ascend
1206	Ascend
1206	Ascend
1206	Ascend
1206	Ascend
1207	Ascend
1207	Descend
1207	Descend
1208	Descend
1208	Descend
1208	Descend
1209	Descend
1209	Descend
1209	Descend
1210	Ascend

1210	Ascend
1210	Ascend
1210	Ascend
1211	Ascend
1211	Ascend
1211	Ascend
1212	Ascend
1212	Ascend
1212	Ascend
1212	Ascend
1213	Descend
1213	Descend
1214	Descend
1214	Ascend
1214	Ascend
1214	Ascend
1215	Ascend
1215	Ascend
1215	Ascend
1216	Ascend
1216	Ascend
1217	Descend
1217	Descend
1217	Descend
1218	Descend
1218	Descend
1219	Ascend
1221	Ascend
1221	Descend
1222	Ascend
1222	Ascend
1222	Ascend
1223	Ascend
1223	Ascend
1223	Ascend
1223	Ascend
1224	Ascend
1224	Descend
1225	Descend
1225	Descend
1226	Descend
1226	Descend

1226	Descend
1226	Descend
1227	Descend
1227	Descend
1228	Descend
1228	Descend
1228	Descend
1229	Descend
1229	Descend
1230	Ascend
1230	Descend
1230	Descend
1231	Ascend
1231	Descend
1231	Descend
1232	Descend
1233	Descend
1234	Descend
1234	Descend
1234	Descend
1235	Descend
1235	Descend
1236	Descend
1236	Descend
1237	Ascend
1237	Ascend
1237	Ascend
1238	Ascend
1238	Ascend
1239	Ascend
1239	Ascend
1243	Descend
1244	Descend
1244	Descend
1245	Ascend
1245	Ascend
1246	Descend
1247	Ascend
1249	Descend
1249	Descend
1249	Descend
1249	Descend

1250	Descend
1250	Descend
1251	Ascend
1251	Ascend
1251	Ascend
1252	Ascend
1252	Ascend
1252	Ascend
1252	Ascend
1253	Ascend
1253	Ascend
1254	Ascend
1254	Descend
1254	Descend
1255	Descend
1255	Descend
1255	Ascend
1256	Ascend
1256	Ascend
1256	Ascend
1256	Ascend
1257	Ascend
1257	Ascend
1257	Ascend
1257	Ascend
1258	Ascend
1258	Ascend
1259	Ascend
1259	Ascend
1259	Ascend
1260	Ascend
8	AntIn
8	AntIn
12	AntIn
13	AntOut
13	AntIn
14	AntOut
15	AntIn
18	AntIn
19	AntOut
20	AntIn
21	AntIn

21 AntIn
22 AntOut
23 AntIn
26 AntIn
26 AntOut
28 AntOut
29 AntIn
30 AntOut
32 AntIn
33 AntOut
33 AntIn
34 AntIn
34 AntIn
34 AntIn
35 AntIn
36 AntOut
37 AntOut
37 AntIn
38 AntIn
39 AntOut
39 AntIn
40 AntIn
41 AntOut
41 AntIn
42 AntIn
42 AntOut
43 AntOut
43 AntOut
43 AntIn
51 AntOut
51 AntIn
52 AntIn
54 AntOut
54 AntIn
55 AntOut
55 AntIn
56 AntOut
56 AntOut
57 AntOut
58 AntOut
59 AntOut
59 AntIn

59 AntIn
59 AntIn
60 AntOut
61 AntIn
61 AntIn
62 AntIn
62 AntIn
62 AntOut
64 AntIn
64 AntIn
65 AntOut
65 AntOut
66 AntIn
67 AntOut
68 AntOut
69 AntOut
69 AntOut
70 AntOut
71 AntIn
71 AntOut
73 AntIn
73 AntIn
74 AntIn
75 AntOut
78 AntIn
79 AntIn
79 AntIn
80 AntIn
80 AntOut
81 AntOut
81 AntOut
82 AntOut
83 AntOut
84 AntIn
85 AntIn
85 AntOut
86 AntIn
87 AntIn
87 AntIn
87 AntIn
88 AntIn
89 AntIn

90	AntIn
90	AntOut
92	AntOut
92	AntOut
93	AntOut
94	AntIn
96	AntIn
96	AntIn
96	AntIn
97	AntIn
100	AntOut
102	AntOut
103	AntIn
104	AntOut
105	AntOut
106	AntOut
106	AntIn
106	AntIn
109	AntOut
110	AntOut
110	AntOut
111	AntOut
111	AntIn
112	AntOut
113	AntIn
113	AntIn
114	AntIn
115	AntIn
118	AntOut
119	AntOut
120	AntIn
121	AntIn
122	AntIn
124	AntOut
126	AntIn
126	AntIn
126	AntIn
127	AntIn
127	AntIn
128	AntIn
128	AntOut
128	AntOut

128	AntOut
129	AntOut
130	AntOut
131	AntOut
131	AntOut
131	AntOut
133	AntOut
133	AntIn
134	AntIn
134	AntIn
135	AntOut
138	AntIn
138	AntIn
139	AntIn
140	AntIn
140	AntIn
142	AntIn
143	AntIn
145	AntIn
146	AntOut
146	AntOut
147	AntIn
149	AntIn
149	AntIn
150	AntOut
153	AntIn
153	AntIn
154	AntOut
154	AntOut
155	AntOut
155	AntOut
156	AntIn
156	AntIn
156	AntIn
158	AntIn
159	AntOut
160	AntIn
161	AntOut
161	AntIn
162	AntIn
163	AntIn
164	AntOut

165	AntOut
165	AntOut
167	AntIn
169	AntOut
170	AntOut
170	AntIn
171	AntIn
172	AntIn
172	AntIn
173	AntIn
173	AntIn
177	AntIn
177	AntOut
177	AntOut
179	AntIn
180	AntOut
183	AntOut
184	AntOut
184	AntOut
186	AntIn
188	AntIn
188	AntIn
190	AntIn
191	AntOut
192	AntIn
194	AntIn
195	AntIn
195	AntIn
196	AntIn
196	AntIn
196	AntOut
199	AntIn
199	AntIn
200	AntOut
202	AntOut
203	AntIn
204	AntOut
206	AntOut
207	AntOut
209	AntIn
210	AntIn
211	AntIn

211 AntOut
212 AntOut
213 AntIn
213 AntIn
213 AntIn
213 AntIn
214 AntIn
214 AntIn
215 AntIn
216 AntIn
217 AntIn
218 AntIn
219 AntOut
220 AntOut
221 AntIn
221 AntIn
222 AntOut
223 AntOut
224 AntIn
224 AntOut
225 AntIn
226 AntIn
226 AntIn
227 AntOut
227 AntIn
230 AntIn
231 AntIn
232 AntIn
232 AntOut
235 AntIn
235 AntIn
237 AntOut
237 AntOut
237 AntOut
238 AntOut
238 AntOut
239 AntOut
240 AntOut
240 AntOut
243 AntIn
244 AntOut
245 AntIn

245	AntIn
246	AntIn
246	AntOut
248	AntIn
249	AntOut
250	AntOut
251	AntIn
251	AntIn
252	AntOut
254	AntOut
255	AntIn
255	AntIn
256	AntIn
256	AntIn
262	AntIn
262	AntIn
265	AntIn
268	AntOut
269	AntIn
270	AntIn
270	AntIn
271	AntIn
274	AntOut
274	AntIn
275	AntIn
277	AntIn
278	AntIn
279	AntIn
279	AntOut
281	AntOut
283	AntIn
283	AntIn
285	AntIn
285	AntIn
285	AntIn
286	AntOut
287	AntIn
287	AntIn
287	AntIn
289	AntIn
290	AntIn
290	AntIn

291	AntOut
291	AntOut
291	AntOut
292	AntOut
293	AntIn
294	AntIn
294	AntIn
296	AntOut
296	AntIn
297	AntIn
298	AntIn
299	AntOut
299	AntOut
301	AntIn
302	AntOut
303	AntOut
303	AntOut
304	AntOut
304	AntIn
306	AntOut
306	AntOut
306	AntOut
307	AntOut
307	AntIn
308	AntIn
308	AntIn
312	AntOut
313	AntOut
313	AntIn
316	AntIn
316	AntIn
317	AntOut
319	AntOut
320	AntOut
321	AntIn
322	AntIn
323	AntIn
324	AntOut
325	AntIn
325	AntOut
325	AntOut
326	AntOut

327	AntIn
327	AntOut
329	AntOut
329	AntIn
330	AntOut
330	AntOut
331	AntIn
332	AntIn
332	AntOut
332	AntOut
335	AntOut
338	AntIn
340	AntOut
340	AntOut
341	AntIn
341	AntOut
342	AntOut
342	AntOut
342	AntOut
343	AntOut
343	AntOut
343	AntOut
344	AntOut
344	AntOut
346	AntOut
347	AntIn
352	AntIn
353	AntOut
353	AntOut
354	AntIn
354	AntOut
355	AntOut
355	AntOut
358	AntOut
359	AntIn
359	AntOut
360	AntIn
360	AntOut
365	AntOut
366	AntOut
367	AntIn
368	AntOut

369	AntOut
370	AntOut
374	AntOut
374	AntOut
375	AntIn
375	AntIn
376	AntOut
377	AntOut
379	AntOut
380	AntOut
380	AntOut
383	AntIn
383	AntIn
384	AntOut
384	AntIn
387	AntOut
391	AntIn
391	AntIn
397	AntIn
397	AntIn
399	AntIn
399	AntIn
399	AntOut
400	AntOut
402	AntIn
402	AntIn
403	AntIn
405	AntOut
405	AntOut
406	AntIn
406	AntIn
407	AntIn
408	AntIn
408	AntIn
409	AntIn
409	AntIn
411	AntOut
412	AntIn
415	AntOut
416	AntIn
418	AntOut
419	AntIn

421	AntIn
421	AntIn
422	AntIn
423	AntOut
426	AntIn
429	AntOut
430	AntOut
430	AntOut
431	AntIn
431	AntOut
431	AntOut
433	AntIn
435	AntOut
436	AntIn
437	AntIn
439	AntIn
439	AntIn
442	AntIn
447	AntOut
449	AntIn
451	AntOut
455	AntIn
456	AntIn
458	AntIn
458	AntIn
461	AntIn
461	AntOut
464	AntIn
465	AntIn
466	AntIn
470	AntIn
472	AntIn
472	AntIn
472	AntIn
473	AntIn
474	AntIn
477	AntIn
478	AntIn
478	AntIn
479	AntIn
480	AntIn
481	AntOut

482	AntOut
483	AntOut
484	AntOut
487	AntIn
490	AntIn
491	AntIn
492	AntIn
493	AntOut
499	AntIn
500	AntOut
501	AntOut
504	AntIn
505	AntIn
506	AntOut
507	AntOut
507	AntOut
507	AntOut
508	AntIn
510	AntIn
510	AntIn
511	AntIn
511	AntIn
512	AntIn
513	AntIn
515	AntIn
515	AntIn
516	AntIn
518	AntIn
519	AntIn
523	AntOut
523	AntOut
524	AntOut
525	AntIn
531	AntIn
531	AntIn
532	AntIn
534	AntIn
536	AntIn
541	AntIn
542	AntIn
542	AntIn
543	AntIn

543	AntIn
545	AntIn
547	AntIn
555	AntIn
556	AntIn
557	AntIn
558	AntIn
560	AntOut
560	AntIn
561	AntIn
561	AntIn
562	AntIn
563	AntOut
563	AntOut
568	AntIn
570	AntIn
580	AntIn
581	AntIn
582	AntIn
583	AntIn
588	AntIn
589	AntIn
589	AntIn
590	AntIn
591	AntOut
598	AntIn
598	AntOut
602	AntIn
605	AntIn
606	AntIn
629	AntIn
632	AntIn
635	AntIn
653	AntIn
655	AntIn
655	AntIn
656	AntIn
657	AntIn
658	AntIn
659	AntIn
659	AntIn
660	AntIn

661 AntIn
662 AntOut
669 AntIn
670 AntIn
678 AntIn
680 AntOut
681 AntIn
681 AntIn
682 AntIn
682 AntIn
683 AntIn
684 AntIn
686 AntIn
687 AntOut
688 AntOut
689 AntIn
695 AntIn
696 AntIn
698 AntIn
705 AntIn
706 AntIn
708 AntOut
711 AntIn
716 AntIn
718 AntIn
718 AntIn
719 AntIn
720 AntIn
723 AntOut
724 AntIn
725 AntIn
725 AntIn
727 AntIn
727 AntIn
728 AntIn
730 AntIn
731 AntIn
734 AntIn
735 AntIn
736 AntIn
740 AntIn
741 AntIn

743	AntIn
743	AntIn
747	AntIn
748	AntIn
748	AntIn
749	AntIn
752	AntIn
755	AntIn
761	AntIn
762	AntIn
763	AntIn
764	AntIn
765	AntIn
767	AntIn
769	AntIn
771	AntOut
771	AntOut
772	AntOut
776	AntIn
777	AntIn
779	AntOut
779	AntOut
780	AntIn
781	AntIn
785	AntOut
788	AntIn
792	AntIn
793	AntIn
796	AntIn
799	AntIn
801	AntIn
802	AntIn
803	AntIn
807	AntIn
811	AntIn
812	AntIn
813	AntOut
814	AntIn
818	AntOut
819	AntIn
819	AntIn
824	AntIn

825	AntOut
827	AntIn
832	AntIn
833	AntOut
834	AntIn
834	AntIn
835	AntIn
840	AntIn
842	AntIn
848	AntIn
853	AntIn
854	AntOut
855	AntIn
861	AntOut
861	AntOut
862	AntIn
863	AntIn
864	AntIn
871	AntIn
872	AntIn
873	AntIn
873	AntIn
874	AntIn
876	AntIn
880	AntOut
880	AntIn
882	AntIn
882	AntIn
890	AntIn
894	AntOut
897	AntOut
898	AntIn
898	AntIn
899	AntIn
900	AntIn
901	AntIn
903	AntIn
905	AntIn
906	AntIn
906	AntOut
910	AntIn
911	AntOut

913	AntIn
915	AntIn
916	AntIn
917	AntIn
919	AntOut
919	AntIn
921	AntIn
922	AntIn
928	AntIn
928	AntIn
938	AntIn
941	AntIn
942	AntIn
943	AntOut
943	AntOut
949	AntIn
953	AntIn
956	AntIn
960	AntOut
964	AntIn
967	AntIn
968	AntIn
968	AntIn
969	AntIn
974	AntIn
978	AntOut
979	AntIn
981	AntIn
981	AntIn
982	AntIn
983	AntIn
983	AntIn
987	AntOut
987	AntIn
992	AntIn
992	AntIn
996	AntOut
997	AntIn
999	AntIn
1000	AntOut
1003	AntIn
1004	AntIn

1006	AntIn
1011	AntIn
1011	AntIn
1013	AntOut
1013	AntOut
1013	AntOut
1019	AntIn
1022	AntIn
1022	AntOut
1024	AntIn
1026	AntIn
1027	AntIn
1033	AntIn
1041	AntOut
1042	AntIn
1044	AntIn
1046	AntOut
1046	AntOut
1048	AntOut
1049	AntIn
1058	AntOut
1059	AntOut
1059	AntIn
1059	AntIn
1061	AntIn
1065	AntOut
1067	AntIn
1068	AntOut
1074	AntOut
1075	AntOut
1078	AntIn
1079	AntIn
1083	AntOut
1084	AntIn
1085	AntOut
1087	AntOut
1093	AntIn
1094	AntIn
1095	AntIn
1098	AntIn
1101	AntIn
1101	AntOut

1103	AntIn
1104	AntIn
1104	AntIn
1106	AntIn
1110	AntIn
1113	AntIn
1114	AntIn
1114	AntIn
1116	AntOut
1116	AntOut
1117	AntIn
1119	AntIn
1122	AntIn
1123	AntIn
1130	AntIn
1131	AntIn
1133	AntIn
1134	AntOut
1135	AntIn
1148	AntOut
1151	AntIn
1152	AntIn
1152	AntOut
1152	AntOut
1155	AntOut
1156	AntOut
1162	AntOut
1164	AntIn
1167	AntIn
1168	AntIn
1168	AntOut
1168	AntOut
1169	AntIn
1179	AntIn
1183	AntIn
1184	AntOut
1186	AntIn
1187	AntOut
1188	AntIn
1192	AntOut
1193	AntOut
1193	AntIn

1198	AntIn
1199	AntOut
1202	AntOut
1205	AntOut
1206	AntIn
1208	AntOut
1210	AntOut
1210	AntOut
1210	AntOut
1211	AntOut
1213	AntOut
1214	AntOut
1218	AntIn
1219	AntIn
1220	AntIn
1221	AntIn
1221	AntOut
1222	AntOut
1223	AntIn
1224	AntOut
1224	AntOut
1228	AntIn
1228	AntOut
1233	AntIn
1234	AntOut
1235	AntIn
1239	AntOut
1239	AntIn
1241	AntIn
1248	AntIn
1251	AntIn
1253	AntIn
1255	AntIn
1258	AntIn
1258	AntIn
1260	AntIn
1260	AntIn
