# Supplementary material for: Interactions Increase Forager Availability and Activity in Harvester Ants
Source: PLoS One. 2015 Nov 5;10(11):e0141971. doi: 10.1371/journal.pone.0141971 (PMC4635008; doi:10.1371/journal.pone.0141971)
Supplement: S3 Dataset — We observed and filmed behavior inside the nest during and after forager removals. This dataset shows our counts made from the films of the numbers of returning and outgoing foragers at the nest entrance and the number of ascending and descending ants at all tunnel entrances. (ZIP) [file pone.0141971.s004.zip › S3 Dataset/2013 Correlation Data 868 8-19.pdf]

**Researcher**	**Jovel Queirolo**
**Colony 868**	
**8/19/13**	
**Video time (seconds)**	**Event**
3	Descend
3	Descend
3	Descend
4	Ascend
4	Ascend
4	Ascend
5	Descend
6	Ascend
6	Ascend
7	Ascend
7	Ascend
13	Ascend
13	Ascend
14	Ascend
14	Ascend
15	Ascend
16	Descend
16	Descend
16	Descend
17	Ascend
17	Ascend
17	Ascend
17	Ascend
18	Ascend
18	Descend
19	Descend
19	Descend
20	Descend
20	Descend
21	Descend
21	Descend
22	Descend
22	Descend
23	Ascend
23	Ascend
24	Ascend
26	Descend

26	Descend
27	Descend
27	Descend
27	Ascend
28	Ascend
29	Descend
29	Descend
30	Descend
30	Descend
30	Descend
31	Descend
31	Descend
32	Ascend
32	Ascend
32	Ascend
33	Ascend
33	Ascend
34	Ascend
34	Ascend
34	Ascend
35	Ascend
35	Descend
35	Descend
36	Ascend
36	Descend
37	Descend
39	Descend
39	Descend
40	Descend
40	Ascend
41	Descend
41	Ascend
41	Descend
41	Descend
44	Ascend
44	Ascend
44	Ascend
46	Descend
46	Descend
46	Ascend
46	Ascend
47	Descend

47	Ascend
47	Ascend
47	Ascend
49	Ascend
49	Ascend
49	Ascend
50	Descend
50	Descend
50	Descend
50	Descend
51	Descend
51	Descend
51	Descend
51	Descend
52	Descend
52	Descend
52	Descend
52	Descend
53	Descend
53	Descend
53	Descend
54	Descend
54	Descend
54	Descend
55	Descend
55	Descend
56	Descend
56	Descend
56	Descend
57	Descend
57	Descend
58	Descend
58	Ascend
59	Ascend
59	Ascend
59	Ascend
60	Ascend
60	Ascend
61	Ascend
61	Ascend
62	Descend
62	Descend

63	Descend
63	Ascend
63	Ascend
64	Ascend
65	Ascend
65	Ascend
66	Ascend
66	Descend
67	Descend
68	Descend
69	Ascend
70	Ascend
70	Ascend
70	Ascend
70	Ascend
71	Ascend
71	Ascend
72	Descend
72	Descend
73	Descend
73	Descend
73	Descend
73	Descend
74	Descend
75	Descend
75	Descend
76	Descend
76	Descend
77	Ascend
77	Ascend
77	Ascend
78	Ascend
78	Descend
78	Descend
79	Descend
80	Descend
80	Ascend
80	Ascend
81	Ascend
81	Ascend
82	Ascend
82	Ascend

82	Ascend
83	Descend
83	Ascend
84	Ascend
84	Descend
84	Descend
85	Ascend
85	Ascend
86	Ascend
86	Descend
86	Descend
87	Ascend
87	Ascend
87	Ascend
88	Ascend
88	Ascend
89	Ascend
89	Ascend
89	Ascend
90	Ascend
90	Ascend
90	Ascend
91	Ascend
91	Ascend
91	Ascend
92	Ascend
92	Ascend
93	Ascend
93	Ascend
93	Ascend
94	Ascend
94	Descend
94	Descend
95	Descend
95	Descend
95	Descend
95	Descend
96	Descend
96	Descend
96	Descend
96	Descend
97	Descend

97	Descend
97	Ascend
98	Ascend
98	Ascend
98	Ascend
98	Ascend
99	Ascend
99	Ascend
99	Ascend
100	Ascend
100	Ascend
100	Ascend
101	Descend
101	Ascend
101	Ascend
102	Ascend
102	Ascend
103	Ascend
103	Ascend
103	Ascend
104	Ascend
104	Ascend
104	Ascend
105	Ascend
105	Ascend
105	Descend
106	Descend
106	Descend
107	Descend
108	Descend
108	Descend
109	Descend
110	Ascend
111	Descend
112	Descend
112	Descend
112	Descend
113	Descend
113	Descend
113	Descend
114	Descend
114	Descend

115	Descend
115	Ascend
115	Ascend
115	Ascend
116	Ascend
116	Ascend
116	Ascend
116	Ascend
117	Ascend
117	Ascend
117	Ascend
117	Ascend
117	Ascend
118	Ascend
118	Ascend
118	Ascend
118	Ascend
119	Ascend
119	Ascend
119	Ascend
120	Ascend
120	Ascend
120	Ascend
120	Ascend
121	Ascend
121	Ascend
121	Ascend
122	Ascend
122	Ascend
122	Descend
122	Descend
123	Descend
123	Descend
123	Descend
123	Descend
124	Ascend
124	Ascend
124	Ascend
124	Ascend
125	Descend
125	Ascend
125	Ascend

125	Descend
125	Descend
126	Ascend
126	Ascend
126	Descend
126	Descend
127	Ascend
127	Ascend
127	Ascend
127	Ascend
128	Ascend
128	Ascend
128	Ascend
129	Ascend
129	Ascend
129	Ascend
130	Ascend
130	Descend
130	Descend
131	Descend
131	Descend
132	Descend
132	Descend
132	Descend
133	Descend
133	Descend
133	Descend
134	Descend
134	Descend
134	Descend
134	Descend
135	Descend
136	Descend
136	Descend
136	Ascend
137	Ascend
137	Ascend
137	Ascend
137	Ascend
138	Ascend
138	Ascend
138	Ascend

138	Ascend
138	Ascend
139	Ascend
139	Ascend
139	Ascend
139	Ascend
139	Ascend
140	Ascend
140	Ascend
140	Ascend
140	Ascend
141	Ascend
141	Ascend
141	Ascend
141	Ascend
141	Ascend
142	Ascend
143	Ascend
144	Ascend
145	Ascend
146	Ascend
147	Ascend
147	Ascend
148	Ascend
148	Ascend
149	Ascend
149	Ascend
150	Ascend
150	Ascend
151	Ascend
151	Ascend
152	Ascend
153	Ascend
153	Ascend
153	Ascend
154	Ascend
154	Ascend
154	Ascend
155	Ascend
155	Ascend
155	Ascend
155	Ascend

156	Ascend
156	Ascend
157	Descend
157	Descend
157	Descend
158	Descend
158	Descend
158	Descend
158	Descend
158	Descend
159	Descend
159	Descend
159	Descend
160	Descend
161	Descend
161	Descend
162	Descend
162	Descend
163	Descend
163	Ascend
164	Ascend
164	Ascend
165	Ascend
165	Ascend
165	Ascend
165	Ascend
166	Ascend
166	Ascend
166	Ascend
167	Ascend
168	Ascend
168	Ascend
169	Ascend
171	Ascend
172	Ascend
172	Descend
173	Descend
173	Descend
173	Descend
173	Descend
174	Descend
174	Ascend

174	Ascend
174	Ascend
175	Ascend
175	Ascend
175	Ascend
176	Ascend
176	Ascend
176	Ascend
176	Ascend
176	Ascend
177	Ascend
177	Ascend
177	Descend
177	Descend
177	Descend
178	Ascend
178	Ascend
178	Ascend
178	Ascend
178	Ascend
179	Ascend
179	Ascend
179	Ascend
179	Ascend
180	Ascend
180	Ascend
180	Ascend
181	Ascend
181	Descend
181	Descend
181	Descend
182	Ascend
182	Ascend
182	Ascend
182	Ascend
182	Ascend
183	Descend
183	Descend
184	Descend
184	Descend
184	Descend
185	Descend

185	Descend
186	Descend
186	Descend
186	Descend
186	Descend
187	Descend
187	Descend
188	Ascend
188	Ascend
189	Ascend
189	Ascend
189	Ascend
190	Ascend
190	Ascend
191	Ascend
191	Ascend
191	Ascend
192	Ascend
192	Ascend
192	Ascend
193	Ascend
193	Descend
193	Descend
194	Descend
194	Descend
194	Descend
195	Descend
195	Descend
196	Descend
196	Descend
197	Descend
197	Descend
198	Descend
198	Descend
198	Ascend
198	Ascend
199	Ascend
199	Ascend
199	Ascend
200	Ascend
200	Ascend
200	Ascend

201	Ascend
201	Ascend
202	Descend
202	Descend
203	Ascend
204	Ascend
204	Ascend
204	Ascend
204	Ascend
204	Ascend
205	Ascend
205	Ascend
205	Descend
206	Descend
206	Descend
206	Descend
207	Descend
207	Descend
208	Descend
208	Descend
209	Descend
209	Descend
210	Descend
211	Ascend
211	Ascend
211	Ascend
212	Ascend
212	Ascend
213	Ascend
213	Ascend
213	Ascend
213	Ascend
214	Ascend
216	Ascend
216	Ascend
217	Ascend
218	Ascend
219	Ascend
220	Ascend
220	Ascend
221	Ascend
221	Ascend

222	Ascend
222	Ascend
223	Ascend
223	Ascend
224	Ascend
224	Ascend
224	Ascend
224	Ascend
225	Descend
225	Descend
225	Descend
225	Descend
226	Descend
226	Descend
228	Descend
230	Ascend
231	Ascend
231	Descend
231	Descend
233	Ascend
233	Ascend
234	Ascend
234	Ascend
234	Ascend
235	Descend
235	Descend
235	Descend
236	Ascend
236	Ascend
237	Ascend
237	Ascend
238	Descend
238	Descend
238	Descend
239	Descend
239	Descend
240	Descend
240	Descend
241	Descend
241	Descend
242	Ascend
242	Ascend

243	Ascend
243	Ascend
243	Ascend
243	Ascend
244	Ascend
244	Descend
244	Descend
244	Descend
245	Descend
245	Descend
245	Descend
245	Descend
246	Descend
246	Descend
246	Descend
247	Descend
248	Descend
248	Descend
248	Descend
249	Descend
249	Ascend
249	Ascend
250	Ascend
250	Ascend
251	Descend
252	Descend
252	Descend
253	Ascend
253	Ascend
253	Ascend
253	Ascend
254	Ascend
254	Ascend
254	Ascend
254	Ascend
255	Ascend
255	Ascend
255	Ascend
256	Ascend
256	Ascend
256	Ascend
256	Ascend

257 Ascend
258 Ascend
259 Ascend
259 Ascend
260 Ascend
260 Ascend
262 Ascend
264 Ascend
265 Descend
265 Descend
266 Ascend
266 Ascend
266 Ascend
267 Ascend
267 Descend
267 Descend
268 Ascend
268 Descend
269 Descend
269 Ascend
270 Ascend
270 Ascend
271 Descend
272 Ascend
273 Ascend
273 Ascend
274 Ascend
274 Ascend
274 Ascend
275 Ascend
275 Ascend
275 Ascend
275 Ascend
277 Ascend
277 Ascend
277 Ascend
277 Ascend
278 Ascend
278 Ascend
278 Ascend
278 Ascend
279 Ascend

279	Descend
279	Descend
280	Descend
280	Descend
280	Ascend
280	Ascend
280	Ascend
281	Ascend
281	Ascend
282	Ascend
282	Ascend
283	Ascend
283	Ascend
283	Ascend
284	Ascend
284	Ascend
284	Ascend
285	Ascend
285	Ascend
285	Ascend
287	Ascend
287	Ascend
287	Ascend
287	Ascend
288	Ascend
288	Ascend
289	Ascend
291	Ascend
291	Ascend
292	Ascend
292	Descend
292	Descend
294	Ascend
295	Ascend
295	Ascend
296	Descend
296	Descend
297	Descend
297	Descend
297	Descend
297	Descend
298	Descend

298	Descend
298	Descend
299	Descend
299	Ascend
299	Ascend
299	Descend
300	Descend
300	Descend
300	Descend
300	Descend
300	Descend
301	Descend
301	Descend
302	Descend
302	Descend
302	Descend
303	Ascend
303	Ascend
303	Ascend
304	Ascend
304	Ascend
305	Descend
305	Descend
305	Ascend
306	Ascend
308	Ascend
308	Ascend
309	Ascend
310	Ascend
310	Ascend
311	Ascend
311	Ascend
311	Ascend
313	Ascend
314	Ascend
315	Descend
316	Ascend
316	Descend
317	Descend
319	Descend
320	Ascend
320	Ascend

321	Ascend
321	Ascend
322	Descend
322	Descend
323	Descend
324	Ascend
326	Ascend
326	Descend
327	Ascend
328	Ascend
328	Descend
328	Descend
329	Ascend
329	Ascend
330	Ascend
331	Descend
331	Descend
332	Descend
333	Descend
333	Ascend
334	Ascend
335	Ascend
335	Ascend
336	Ascend
337	Ascend
339	Ascend
339	Ascend
340	Ascend
341	Ascend
342	Ascend
344	Ascend
344	Ascend
344	Ascend
344	Ascend
345	Ascend
345	Ascend
345	Ascend
345	Ascend
346	Ascend
346	Ascend
346	Ascend
348	Ascend

348	Ascend
349	Ascend
349	Ascend
349	Ascend
350	Ascend
350	Ascend
351	Ascend
353	Ascend
353	Ascend
353	Ascend
353	Ascend
354	Ascend
354	Ascend
355	Ascend
356	Ascend
357	Ascend
358	Ascend
358	Ascend
363	Ascend
364	Ascend
364	Ascend
365	Ascend
367	Ascend
367	Ascend
368	Ascend
369	Ascend
369	Ascend
370	Ascend
370	Ascend
370	Ascend
371	Ascend
371	Ascend
371	Ascend
372	Ascend
372	Ascend
372	Ascend
373	Ascend
373	Ascend
374	Ascend
379	Ascend
383	Ascend
385	Descend

386	Descend
387	Descend
388	Ascend
389	Descend
395	Ascend
395	Ascend
395	Ascend
396	Descend
397	Descend
399	Descend
400	Ascend
400	Ascend
401	Ascend
404	Ascend
405	Ascend
405	Ascend
406	Ascend
408	Ascend
411	Ascend
412	Ascend
415	Ascend
416	Descend
418	Ascend
419	Descend
419	Descend
421	Descend
422	Descend
423	Ascend
424	Ascend
424	Descend
429	Ascend
429	Ascend
430	Ascend
430	Ascend
430	Ascend
431	Ascend
432	Ascend
433	Ascend
433	Ascend
434	Ascend
434	Ascend
435	Ascend

435	Ascend
435	Ascend
436	Ascend
437	Ascend
437	Descend
438	Descend
438	Descend
438	Descend
439	Descend
439	Descend
440	Descend
440	Descend
440	Descend
441	Descend
441	Descend
441	Descend
442	Descend
442	Descend
442	Descend
443	Descend
443	Descend
443	Descend
444	Descend
444	Descend
444	Descend
445	Descend
445	Descend
445	Descend
445	Descend
446	Descend
446	Descend
447	Descend
447	Descend
448	Descend
448	Descend
449	Descend
449	Descend
449	Descend
450	Descend
450	Descend
450	Descend
451	Descend

451	Descend
451	Descend
452	Descend
452	Descend
453	Descend
453	Descend
453	Descend
454	Descend
454	Descend
454	Descend
455	Descend
455	Descend
457	Descend
457	Descend
458	Descend
458	Descend
460	Ascend
463	Descend
463	Descend
464	Descend
464	Descend
466	Descend
466	Descend
467	Descend
468	Descend
470	Descend
470	Descend
472	Descend
472	Descend
473	Descend
473	Descend
474	Descend
474	Descend
475	Descend
476	Descend
476	Descend
476	Descend
477	Descend
477	Descend
477	Descend
478	Descend
478	Descend

481	Descend
481	Descend
482	Descend
482	Descend
484	Descend
485	Ascend
489	Ascend
490	Descend
493	Descend
497	Descend
498	Descend
499	Descend
500	Descend
502	Descend
503	Ascend
503	Descend
505	Ascend
506	Descend
511	Descend
514	Descend
516	Descend
518	Descend
519	Descend
520	Descend
522	Descend
524	Descend
525	Descend
525	Descend
553	Ascend
553	Descend
555	Descend
557	Ascend
559	Descend
560	Ascend
569	Ascend
572	Descend
572	Descend
578	Ascend
585	Descend
586	Descend
601	Descend
602	Ascend

603	Ascend
604	Descend
605	Descend
613	Descend
620	Ascend
622	Descend
624	Descend
634	Ascend
635	Descend
640	Ascend
643	Descend
657	Descend
657	Descend
660	Descend
669	Descend
688	Descend
691	Ascend
693	Descend
696	Descend
697	Ascend
698	Descend
700	Descend
706	Descend
711	Descend
714	Ascend
714	Descend
716	Ascend
729	Descend
730	Descend
733	Ascend
734	Descend
743	Descend
747	Descend
752	Ascend
756	Descend
761	Descend
763	Ascend
765	Descend
766	Descend
772	Descend
779	Descend
810	Descend

814	Descend
818	Descend
822	Ascend
823	Descend
840	Descend
842	Descend
844	Descend
847	Ascend
849	Ascend
851	Descend
877	Descend
894	Ascend
895	Descend
899	Ascend
8	AntIn
16	AntIn
17	AntIn
18	AntIn
21	AntIn
23	AntIn
23	AntIn
24	AntIn
26	AntIn
26	AntOut
26	AntOut
33	AntIn
34	AntIn
35	AntIn
36	AntIn
37	AntIn
38	AntIn
38	AntIn
38	AntOut
42	AntOut
44	AntOut
46	AntOut
50	AntIn
56	AntIn
57	AntOut
57	AntIn
58	AntIn
59	AntIn

61	AntOut
63	AntIn
63	AntOut
63	AntOut
67	AntIn
68	AntOut
68	AntOut
70	AntIn
70	AntIn
73	AntOut
73	AntOut
74	AntIn
74	AntIn
78	AntOut
78	AntOut
78	AntOut
79	AntOut
81	AntIn
81	AntOut
87	AntOut
87	AntOut
88	AntIn
96	AntIn
96	AntIn
99	AntOut
99	AntOut
100	AntOut
101	AntOut
104	AntIn
105	AntIn
106	AntIn
107	AntIn
108	AntOut
109	AntIn
111	AntOut
116	AntOut
119	AntIn
120	AntOut
122	AntOut
122	AntOut
126	AntIn
126	AntIn

127	AntIn
134	AntIn
137	AntIn
137	AntIn
139	AntIn
139	AntIn
139	AntOut
141	AntIn
142	AntIn
144	AntIn
147	AntIn
150	AntIn
150	AntIn
151	AntIn
151	AntOut
152	AntOut
153	AntOut
155	AntIn
156	AntIn
157	AntIn
159	AntOut
164	AntIn
165	AntIn
165	AntOut
165	AntIn
166	AntOut
171	AntIn
171	AntIn
175	AntOut
176	AntIn
179	AntIn
179	AntIn
180	AntOut
180	AntOut
181	AntIn
184	AntIn
185	AntIn
185	AntIn
186	AntIn
187	AntOut
187	AntIn
188	AntIn

188	AntIn
190	AntIn
191	AntIn
192	AntOut
194	AntIn
198	AntIn
198	AntIn
201	AntIn
202	AntOut
204	AntOut
206	AntIn
206	AntIn
208	AntOut
211	AntIn
212	AntIn
212	AntOut
214	AntOut
215	AntOut
218	AntIn
219	AntIn
219	AntOut
220	AntOut
221	AntOut
224	AntOut
227	AntOut
227	AntOut
231	AntIn
233	AntIn
233	AntIn
233	AntIn
234	AntIn
235	AntIn
235	AntOut
236	AntIn
238	AntOut
238	AntOut
239	AntOut
241	AntIn
241	AntIn
242	AntIn
243	AntOut
244	AntIn

245	AntOut
245	AntOut
246	AntIn
248	AntOut
248	AntOut
248	AntIn
248	AntIn
253	AntIn
254	AntIn
254	AntOut
255	AntOut
256	AntOut
257	AntOut
257	AntOut
258	AntOut
260	AntIn
264	AntOut
264	AntIn
265	AntIn
268	AntOut
270	AntIn
270	AntOut
271	AntOut
271	AntOut
272	AntOut
272	AntOut
273	AntOut
273	AntIn
274	AntIn
275	AntIn
280	AntIn
282	AntOut
283	AntIn
283	AntIn
284	AntIn
285	AntOut
286	AntIn
287	AntIn
287	AntIn
287	AntIn
288	AntOut
289	AntIn

290	AntIn
290	AntOut
291	AntIn
292	AntOut
292	AntOut
293	AntOut
295	AntOut
295	AntOut
297	AntIn
299	AntOut
299	AntOut
300	AntOut
300	AntIn
301	AntIn
302	AntIn
302	AntOut
303	AntIn
305	AntOut
305	AntOut
309	AntOut
309	AntIn
310	AntIn
312	AntIn
313	AntIn
313	AntIn
314	AntIn
316	AntIn
316	AntIn
317	AntIn
317	AntOut
319	AntIn
319	AntOut
320	AntOut
322	AntIn
323	AntOut
323	AntIn
326	AntOut
326	AntOut
326	AntOut
326	AntIn
327	AntIn
328	AntOut

329	AntOut
329	AntOut
329	AntOut
330	AntIn
332	AntIn
333	AntIn
334	AntIn
334	AntOut
335	AntOut
335	AntOut
336	AntIn
337	AntIn
340	AntOut
341	AntIn
342	AntIn
343	AntIn
345	AntOut
347	AntOut
347	AntOut
348	AntOut
348	AntOut
351	AntOut
355	AntIn
357	AntIn
357	AntOut
358	AntIn
359	AntIn
359	AntOut
361	AntIn
365	AntOut
366	AntOut
367	AntIn
369	AntIn
369	AntOut
370	AntOut
370	AntOut
370	AntOut
373	AntIn
373	AntIn
374	AntOut
379	AntIn
379	AntOut

380	AntIn
380	AntOut
381	AntOut
381	AntOut
384	AntIn
384	AntIn
385	AntOut
386	AntIn
388	AntOut
389	AntOut
390	AntIn
391	AntIn
392	AntIn
393	AntOut
393	AntOut
394	AntOut
395	AntOut
395	AntOut
396	AntOut
398	AntIn
399	AntIn
399	AntIn
400	AntIn
402	AntIn
403	AntIn
404	AntIn
404	AntIn
405	AntIn
410	AntOut
412	AntIn
412	AntOut
413	AntOut
413	AntOut
414	AntOut
415	AntOut
415	AntIn
416	AntIn
416	AntIn
417	AntIn
417	AntIn
420	AntOut
420	AntOut

421	AntIn
422	AntIn
422	AntIn
424	AntOut
424	AntIn
425	AntIn
426	AntOut
427	AntIn
427	AntIn
431	AntIn
431	AntIn
432	AntIn
433	AntIn
433	AntOut
436	AntOut
437	AntOut
437	AntIn
438	AntIn
440	AntIn
444	AntIn
445	AntIn
449	AntIn
451	AntIn
454	AntOut
455	AntIn
458	AntOut
460	AntOut
467	AntIn
467	AntOut
475	AntOut
488	AntOut
496	AntOut
498	AntOut
498	AntIn
499	AntIn
501	AntIn
511	AntIn
516	AntIn
517	AntIn
518	AntIn
520	AntIn
521	AntIn

559	AntIn
568	AntIn
576	AntIn
585	AntIn
604	AntIn
611	AntIn
621	AntIn
632	AntIn
650	AntIn
654	AntIn
666	AntIn
678	AntIn
679	AntIn
679	AntIn
682	AntOut
690	AntIn
704	AntIn
716	AntIn
716	AntOut
722	AntIn
729	AntIn
741	AntIn
741	AntIn
742	AntIn
743	AntIn
749	AntIn
757	AntIn
767	AntIn
789	AntOut
791	AntOut
793	AntIn
800	AntOut
806	AntIn
808	AntIn
810	AntIn
814	AntIn
817	AntIn
819	AntOut
840	AntIn
844	AntOut
848	AntIn
854	AntOut

861	AntIn
881	AntIn
887	AntIn
888	AntIn
893	AntIn
901	AntIn
902	AntIn
