# Supplementary material for: Interactions Increase Forager Availability and Activity in Harvester Ants
Source: PLoS One. 2015 Nov 5;10(11):e0141971. doi: 10.1371/journal.pone.0141971 (PMC4635008; doi:10.1371/journal.pone.0141971)
Supplement: S3 Dataset — We observed and filmed behavior inside the nest during and after forager removals. This dataset shows our counts made from the films of the numbers of returning and outgoing foragers at the nest entrance and the number of ascending and descending ants at all tunnel entrances. (ZIP) [file pone.0141971.s004.zip › S3 Dataset/2013 Correlation Data 868 8-20.pdf]

**Researcher**	**Jovel Queirolo**
**Colony 868**	
**8/20/13**	
**Video time (seconds)**	**Event**
3	Ascend
4	Descend
4	Ascend
5	Ascend
5	Descend
6	Ascend
6	Ascend
8	Descend
9	Ascend
9	Ascend
10	Descend
10	Descend
11	Descend
12	Ascend
14	Ascend
14	Descend
16	Ascend
17	Ascend
19	Ascend
20	Ascend
21	Ascend
21	Ascend
22	Ascend
22	Descend
23	Descend
23	Descend
24	Descend
24	Ascend
24	Ascend
25	Ascend
26	Ascend
26	Ascend
27	Ascend
28	Ascend
29	Ascend
29	Ascend
30	Ascend

30 Ascend
31 Ascend
31 Descend
32 Descend
33 Descend
34 Ascend
34 Ascend
34 Ascend
35 Ascend
35 Ascend
36 Ascend
37 Ascend
38 Ascend
38 Descend
39 Descend
41 Descend
41 Descend
42 Ascend
42 Ascend
43 Descend
43 Descend
43 Ascend
45 Ascend
47 Ascend
48 Descend
52 Ascend
52 Descend
54 Ascend
55 Ascend
55 Ascend
55 Ascend
57 Descend
58 Ascend
60 Ascend
61 Ascend
62 Ascend
62 Ascend
62 Ascend
63 Ascend
63 Ascend
64 Ascend
65 Ascend

66	Descend
67	Ascend
67	Ascend
67	Ascend
68	Ascend
68	Ascend
69	Descend
70	Descend
70	Descend
71	Ascend
71	Ascend
72	Ascend
72	Ascend
73	Ascend
74	Ascend
75	Ascend
77	Ascend
77	Ascend
79	Ascend
79	Ascend
81	Descend
82	Descend
83	Descend
84	Descend
84	Ascend
84	Ascend
85	Ascend
85	Ascend
85	Ascend
86	Ascend
86	Ascend
86	Ascend
87	Ascend
87	Ascend
87	Ascend
88	Ascend
90	Ascend
90	Ascend
91	Ascend
91	Ascend
92	Descend
92	Descend

92	Descend
93	Descend
94	Ascend
95	Ascend
95	Descend
96	Ascend
96	Ascend
96	Ascend
97	Ascend
97	Ascend
97	Ascend
98	Ascend
99	Ascend
99	Ascend
100	Descend
100	Descend
100	Descend
101	Descend
101	Descend
102	Descend
103	Descend
103	Ascend
104	Ascend
104	Ascend
105	Ascend
106	Ascend
106	Ascend
108	Ascend
109	Descend
111	Ascend
111	Ascend
112	Descend
113	Descend
114	Descend
114	Descend
115	Descend
115	Descend
116	Ascend
116	Ascend
116	Ascend
117	Ascend
118	Descend

119	Descend
119	Ascend
121	Descend
122	Descend
122	Descend
123	Descend
123	Descend
124	Descend
127	Descend
128	Descend
130	Descend
132	Descend
133	Descend
134	Descend
136	Descend
137	Descend
137	Descend
139	Ascend
141	Ascend
142	Ascend
144	Ascend
146	Ascend
147	Descend
149	Ascend
150	Ascend
155	Descend
155	Descend
156	Descend
156	Descend
157	Ascend
159	Descend
160	Descend
161	Descend
162	Descend
164	Descend
165	Ascend
166	Ascend
167	Ascend
168	Ascend
169	Ascend
169	Ascend
170	Ascend

171	Descend
171	Descend
172	Descend
173	Descend
174	Descend
174	Descend
175	Ascend
176	Ascend
176	Ascend
177	Ascend
177	Ascend
177	Ascend
178	Ascend
179	Ascend
181	Ascend
181	Ascend
182	Ascend
184	Ascend
184	Ascend
186	Descend
187	Descend
187	Descend
188	Ascend
188	Ascend
190	Ascend
191	Ascend
194	Ascend
194	Ascend
195	Ascend
196	Descend
196	Descend
196	Descend
199	Ascend
199	Ascend
200	Ascend
200	Ascend
201	Ascend
202	Ascend
203	Ascend
203	Ascend
205	Ascend
205	Descend

206	Descend
206	Descend
207	Ascend
207	Ascend
210	Descend
210	Descend
213	Ascend
215	Descend
216	Descend
217	Ascend
218	Ascend
218	Ascend
219	Ascend
219	Ascend
221	Ascend
222	Descend
222	Descend
223	Descend
223	Descend
224	Descend
224	Descend
225	Descend
226	Descend
227	Ascend
228	Ascend
228	Ascend
229	Ascend
229	Ascend
230	Ascend
231	Ascend
231	Ascend
232	Ascend
232	Ascend
233	Ascend
234	Ascend
235	Ascend
236	Ascend
236	Ascend
238	Ascend
238	Ascend
238	Ascend
239	Ascend

239	Ascend
240	Ascend
240	Ascend
241	Ascend
241	Ascend
242	Ascend
242	Ascend
242	Ascend
243	Ascend
243	Ascend
244	Ascend
244	Ascend
245	Ascend
247	Ascend
248	Ascend
249	Ascend
250	Ascend
250	Ascend
251	Descend
252	Descend
252	Descend
254	Descend
254	Ascend
254	Ascend
255	Ascend
255	Descend
256	Descend
257	Descend
258	Ascend
258	Ascend
259	Ascend
260	Ascend
260	Ascend
261	Ascend
261	Descend
262	Descend
262	Descend
263	Descend
264	Descend
266	Ascend
266	Ascend
267	Ascend

267	Ascend
268	Ascend
268	Ascend
268	Ascend
269	Descend
269	Descend
270	Ascend
270	Ascend
271	Ascend
271	Ascend
272	Ascend
272	Ascend
272	Ascend
273	Ascend
275	Ascend
276	Ascend
276	Ascend
276	Ascend
276	Ascend
277	Ascend
278	Ascend
280	Ascend
281	Ascend
281	Ascend
281	Ascend
282	Ascend
282	Ascend
283	Ascend
283	Ascend
285	Descend
285	Descend
287	Descend
288	Descend
288	Descend
291	Descend
291	Descend
291	Descend
293	Ascend
293	Ascend
295	Ascend
295	Ascend
296	Descend

297	Descend
297	Descend
299	Descend
301	Ascend
302	Descend
302	Descend
302	Descend
303	Descend
303	Descend
303	Descend
304	Descend
304	Descend
306	Descend
306	Descend
308	Descend
308	Descend
308	Ascend
308	Ascend
310	Ascend
310	Ascend
312	Ascend
315	Ascend
315	Ascend
316	Descend
317	Descend
318	Descend
318	Descend
319	Descend
319	Descend
320	Descend
321	Descend
321	Descend
322	Descend
322	Descend
324	Ascend
325	Ascend
326	Ascend
326	Ascend
328	Ascend
328	Ascend
330	Ascend
331	Descend

331	Descend
333	Ascend
335	Descend
335	Descend
336	Descend
336	Descend
337	Descend
337	Ascend
337	Ascend
337	Ascend
338	Ascend
338	Ascend
338	Ascend
339	Ascend
340	Ascend
340	Ascend
342	Descend
342	Descend
342	Descend
343	Descend
344	Descend
345	Descend
346	Ascend
346	Ascend
348	Ascend
348	Ascend
349	Ascend
350	Ascend
350	Ascend
350	Ascend
350	Descend
350	Descend
352	Descend
352	Descend
353	Descend
354	Descend
355	Descend
357	Descend
357	Descend
358	Ascend
359	Ascend
359	Ascend

360	Ascend
368	Descend
368	Descend
368	Descend
369	Descend
369	Descend
369	Descend
371	Descend
372	Ascend
372	Ascend
373	Descend
373	Descend
374	Descend
374	Descend
375	Descend
375	Descend
375	Descend
376	Descend
377	Ascend
377	Ascend
377	Ascend
377	Ascend
378	Ascend
378	Ascend
378	Ascend
378	Ascend
379	Ascend
379	Ascend
380	Ascend
380	Ascend
380	Descend
381	Descend
381	Descend
381	Descend
382	Descend
382	Descend
383	Descend
383	Descend
384	Descend
384	Descend
384	Descend
384	Descend

385	Descend
385	Ascend
385	Ascend
385	Ascend
386	Ascend
386	Ascend
386	Ascend
387	Ascend
388	Ascend
388	Ascend
389	Ascend
389	Ascend
390	Ascend
391	Ascend
391	Ascend
391	Descend
392	Descend
392	Descend
392	Descend
392	Descend
393	Descend
394	Ascend
394	Ascend
397	Descend
397	Descend
398	Descend
398	Descend
398	Descend
398	Descend
398	Descend
399	Ascend
399	Ascend
399	Ascend
399	Ascend
400	Ascend
400	Ascend
400	Ascend
400	Ascend
401	Ascend
402	Ascend
402	Ascend
403	Descend

403	Descend
403	Descend
404	Descend
404	Descend
404	Descend
405	Descend
406	Ascend
406	Ascend
407	Ascend
407	Ascend
407	Ascend
408	Ascend
408	Descend
408	Descend
408	Descend
409	Descend
409	Descend
409	Descend
410	Ascend
410	Ascend
410	Ascend
410	Ascend
411	Ascend
411	Ascend
411	Ascend
412	Ascend
412	Ascend
413	Ascend
413	Ascend
413	Ascend
414	Descend
414	Descend
414	Descend
414	Descend
415	Ascend
415	Ascend
415	Ascend
417	Ascend
420	Ascend
420	Descend
421	Descend
421	Descend

422	Descend
422	Descend
424	Ascend
425	Ascend
426	Ascend
426	Ascend
427	Ascend
427	Ascend
428	Ascend
429	Ascend
430	Ascend
430	Ascend
431	Ascend
431	Ascend
431	Ascend
433	Descend
433	Descend
434	Descend
435	Descend
435	Descend
435	Descend
436	Descend
437	Ascend
437	Ascend
437	Ascend
439	Ascend
439	Ascend
440	Descend
440	Descend
441	Descend
441	Descend
442	Ascend
443	Ascend
444	Ascend
445	Ascend
446	Ascend
446	Ascend
446	Ascend
447	Ascend
448	Descend
448	Descend
448	Descend

449	Descend
450	Descend
451	Descend
451	Descend
452	Descend
453	Ascend
453	Ascend
453	Ascend
454	Ascend
454	Ascend
454	Ascend
456	Ascend
456	Ascend
456	Ascend
457	Ascend
457	Ascend
457	Ascend
459	Ascend
460	Descend
461	Descend
462	Descend
462	Descend
462	Descend
463	Descend
464	Descend
464	Descend
464	Descend
465	Descend
466	Descend
466	Descend
466	Descend
466	Descend
467	Descend
467	Descend
468	Ascend
468	Ascend
468	Ascend
469	Ascend
469	Ascend
470	Ascend
471	Ascend
473	Descend

473	Descend
474	Ascend
474	Ascend
474	Ascend
475	Ascend
476	Ascend
477	Ascend
477	Ascend
479	Descend
479	Descend
479	Descend
479	Descend
481	Descend
481	Descend
482	Descend
482	Descend
484	Ascend
484	Ascend
486	Ascend
487	Ascend
488	Ascend
488	Descend
489	Descend
489	Descend
490	Descend
490	Descend
490	Descend
491	Descend
492	Descend
492	Descend
493	Descend
493	Descend
495	Descend
495	Descend
495	Ascend
496	Ascend
496	Ascend
496	Ascend
497	Descend
498	Descend
498	Descend
499	Ascend

499	Ascend
500	Descend
500	Descend
501	Descend
502	Descend
502	Descend
503	Descend
504	Descend
504	Descend
505	Descend
505	Descend
506	Descend
507	Descend
508	Descend
508	Descend
509	Descend
509	Descend
509	Descend
510	Descend
510	Descend
511	Descend
511	Descend
511	Descend
512	Ascend
512	Ascend
513	Ascend
513	Ascend
513	Ascend
513	Ascend
514	Ascend
514	Ascend
515	Ascend
515	Ascend
515	Ascend
515	Ascend
516	Ascend
517	Ascend
517	Ascend
517	Ascend
518	Ascend
518	Ascend
519	Descend

519	Descend
519	Descend
520	Descend
520	Descend
520	Descend
520	Descend
521	Descend
521	Descend
521	Descend
524	Descend
524	Descend
526	Descend
526	Descend
527	Descend
527	Descend
527	Descend
527	Descend
528	Descend
528	Descend
529	Descend
529	Descend
531	Descend
535	Descend
535	Descend
535	Descend
536	Descend
538	Descend
539	Descend
539	Descend
539	Descend
540	Descend
541	Descend
541	Descend
541	Descend
541	Ascend
542	Ascend
542	Ascend
542	Ascend
543	Ascend
543	Ascend
544	Ascend
544	Ascend

544	Ascend
544	Ascend
545	Ascend
545	Ascend
547	Ascend
547	Ascend
554	Ascend
555	Ascend
555	Ascend
557	Descend
557	Descend
557	Descend
558	Descend
558	Ascend
558	Ascend
559	Descend
559	Descend
561	Descend
561	Descend
563	Descend
563	Descend
564	Ascend
564	Ascend
565	Ascend
565	Ascend
566	Ascend
566	Ascend
567	Descend
567	Descend
568	Descend
568	Descend
569	Descend
569	Descend
569	Descend
570	Descend
574	Descend
576	Ascend
578	Ascend
578	Ascend
579	Descend
579	Descend
580	Descend

580	Descend
580	Descend
581	Descend
582	Descend
583	Descend
583	Descend
585	Descend
588	Ascend
589	Descend
589	Ascend
591	Descend
593	Ascend
594	Descend
595	Ascend
598	Descend
600	Descend
601	Descend
602	Descend
605	Ascend
605	Ascend
606	Ascend
606	Ascend
608	Ascend
608	Descend
609	Descend
611	Descend
612	Descend
612	Descend
614	Descend
614	Descend
621	Descend
622	Descend
624	Descend
626	Descend
626	Descend
627	Descend
628	Descend
628	Descend
630	Descend
632	Descend
633	Descend
633	Descend

634 Ascend
634 Descend
636 Descend
636 Descend
639 Descend
641 Descend
641 Descend
644 Descend
645 Descend
649 Descend
652 Descend
653 Ascend
655 Descend
655 Descend
656 Descend
658 Ascend
659 Ascend
660 Descend
664 Descend
665 Descend
666 Descend
666 Descend
667 Descend
668 Descend
668 Descend
668 Descend
671 Descend
674 Ascend
676 Ascend
676 Ascend
681 Ascend
683 Ascend
683 Descend
686 Descend
687 Descend
689 Descend
690 Descend
693 Descend
694 Ascend
697 Descend
699 Descend
701 Ascend

709	Ascend
710	Ascend
712	Descend
714	Descend
715	Descend
715	Ascend
718	Ascend
718	Ascend
719	Descend
721	Descend
722	Descend
723	Descend
726	Descend
727	Ascend
734	Descend
736	Descend
739	Descend
742	Ascend
744	Descend
745	Ascend
748	Descend
758	Descend
760	Ascend
763	Descend
765	Ascend
767	Descend
769	Descend
770	Ascend
771	Ascend
772	Ascend
774	Descend
775	Descend
778	Descend
781	Descend
785	Descend
787	Descend
788	Descend
790	Descend
791	Descend
793	Descend
794	Ascend
797	Ascend

798	Ascend
799	Ascend
799	Descend
800	Descend
801	Descend
802	Descend
804	Descend
805	Ascend
806	Ascend
806	Descend
807	Descend
808	Ascend
810	Ascend
811	Ascend
812	Ascend
813	Ascend
818	Descend
819	Descend
820	Ascend
821	Ascend
822	Ascend
822	Descend
823	Descend
825	Ascend
825	Ascend
828	Ascend
829	Ascend
830	Descend
831	Ascend
833	Ascend
834	Descend
840	Descend
842	Descend
843	Descend
844	Ascend
845	Descend
849	Descend
850	Ascend
851	Descend
853	Ascend
856	Ascend
856	Ascend

858	Ascend
859	Ascend
860	Ascend
861	Ascend
861	Descend
862	Descend
862	Descend
863	Descend
865	Descend
865	Descend
865	Descend
867	Descend
868	Ascend
871	Ascend
872	Ascend
874	Ascend
876	Descend
876	Descend
877	Ascend
878	Ascend
879	Ascend
884	Ascend
884	Ascend
884	Ascend
885	Ascend
885	Ascend
885	Ascend
885	Ascend
887	Ascend
887	Ascend
888	Descend
888	Descend
888	Descend
889	Descend
890	Ascend
891	Ascend
891	Ascend
891	Ascend
891	Ascend
892	Ascend
892	Ascend
893	Descend

894	Descend
894	Descend
894	Descend
895	Descend
895	Descend
895	Descend
896	Descend
896	Ascend
896	Ascend
896	Ascend
897	Ascend
897	Ascend
898	Ascend
898	Descend
900	Descend
900	Descend
9	AntIn
10	AntIn
10	AntOut
10	AntOut
11	AntOut
12	AntIn
13	AntIn
13	AntIn
15	AntIn
16	AntIn
16	AntOut
16	AntOut
17	AntIn
17	AntOut
17	AntIn
18	AntIn
18	AntIn
19	AntIn
19	AntIn
20	AntOut
21	AntIn
21	AntIn
21	AntOut
22	AntOut
24	AntOut
26	AntOut

27 AntOut
28 AntOut
29 AntOut
30 AntIn
30 AntOut
31 AntIn
31 AntOut
32 AntOut
32 AntIn
33 AntOut
34 AntIn
35 AntIn
36 AntOut
36 AntIn
37 AntOut
37 AntIn
38 AntOut
38 AntOut
39 AntOut
39 AntIn
40 AntIn
41 AntOut
42 AntIn
42 AntIn
42 AntIn
43 AntIn
46 AntOut
47 AntOut
47 AntOut
47 AntOut
48 AntOut
48 AntOut
49 AntOut
49 AntIn
49 AntIn
50 AntIn
50 AntOut
51 AntOut
52 AntIn
53 AntOut
53 AntOut
53 AntIn

55 AntOut
55 AntOut
56 AntIn
57 AntOut
57 AntIn
58 AntOut
59 AntOut
60 AntOut
60 AntOut
61 AntOut
63 AntOut
64 AntOut
64 AntOut
65 AntIn
66 AntIn
67 AntIn
68 AntOut
68 AntOut
68 AntOut
69 AntOut
69 AntOut
70 AntIn
70 AntOut
71 AntIn
72 AntOut
73 AntIn
73 AntIn
73 AntOut
74 AntOut
74 AntIn
75 AntOut
75 AntOut
76 AntOut
76 AntOut
77 AntOut
77 AntIn
77 AntIn
78 AntIn
78 AntIn
79 AntOut
80 AntOut
80 AntOut

80	AntOut
82	AntIn
82	AntIn
82	AntOut
83	AntOut
84	AntIn
84	AntIn
84	AntIn
85	AntOut
86	AntIn
86	AntIn
87	AntIn
88	AntOut
88	AntIn
88	AntIn
88	AntIn
89	AntIn
91	AntIn
92	AntOut
93	AntOut
93	AntOut
94	AntOut
97	AntIn
97	AntIn
99	AntIn
99	AntOut
100	AntIn
101	AntOut
102	AntOut
103	AntOut
104	AntOut
104	AntOut
104	AntOut
104	AntIn
105	AntIn
105	AntOut
106	AntIn
107	AntIn
108	AntIn
108	AntIn
108	AntIn
109	AntIn

109	AntOut
110	AntOut
110	AntOut
111	AntIn
111	AntOut
112	AntOut
112	AntIn
113	AntIn
113	AntOut
114	AntOut
114	AntOut
115	AntIn
115	AntIn
116	AntIn
116	AntOut
117	AntIn
117	AntIn
118	AntIn
119	AntIn
120	AntOut
120	AntOut
121	AntOut
121	AntOut
122	AntIn
122	AntIn
122	AntOut
125	AntOut
128	AntOut
129	AntIn
130	AntOut
130	AntOut
130	AntOut
130	AntOut
131	AntIn
133	AntOut
134	AntOut
134	AntOut
134	AntIn
134	AntIn
137	AntIn
137	AntIn
141	AntIn

143	AntIn
144	AntIn
145	AntOut
146	AntOut
146	AntOut
147	AntIn
147	AntIn
148	AntIn
149	AntIn
149	AntIn
150	AntIn
151	AntIn
151	AntIn
151	AntIn
153	AntIn
153	AntIn
153	AntIn
154	AntOut
154	AntOut
154	AntIn
155	AntOut
156	AntOut
156	AntOut
157	AntOut
157	AntOut
158	AntIn
158	AntIn
158	AntOut
160	AntIn
160	AntOut
163	AntIn
163	AntIn
164	AntIn
164	AntIn
164	AntIn
166	AntIn
166	AntOut
167	AntIn
167	AntOut
168	AntIn
170	AntOut
172	AntIn

172	AntIn
173	AntOut
174	AntIn
175	AntOut
175	AntOut
175	AntIn
177	AntIn
178	AntIn
180	AntOut
180	AntOut
181	AntOut
182	AntIn
182	AntIn
183	AntOut
183	AntIn
183	AntIn
185	AntOut
186	AntOut
187	AntIn
188	AntIn
188	AntIn
188	AntOut
189	AntIn
189	AntIn
190	AntIn
191	AntOut
192	AntIn
192	AntIn
192	AntIn
193	AntIn
193	AntIn
194	AntOut
196	AntIn
196	AntOut
198	AntIn
198	AntIn
198	AntOut
198	AntOut
199	AntOut
200	AntOut
200	AntIn
201	AntIn

201 AntIn
202 AntOut
202 AntIn
205 AntOut
205 AntIn
205 AntIn
207 AntIn
208 AntIn
208 AntOut
209 AntOut
209 AntOut
212 AntOut
212 AntIn
213 AntIn
213 AntOut
216 AntIn
216 AntIn
216 AntOut
217 AntOut
217 AntIn
218 AntIn
218 AntIn
219 AntIn
220 AntIn
220 AntIn
221 AntIn
221 AntOut
223 AntIn
225 AntIn
225 AntOut
226 AntOut
226 AntIn
226 AntIn
226 AntOut
227 AntIn
228 AntIn
228 AntOut
229 AntIn
229 AntOut
229 AntOut
231 AntIn
232 AntOut

232 AntOut
233 AntIn
234 AntOut
234 AntIn
237 AntIn
238 AntIn
238 AntIn
239 AntIn
239 AntIn
240 AntIn
243 AntOut
243 AntIn
243 AntIn
244 AntIn
246 AntIn
246 AntOut
247 AntIn
248 AntOut
248 AntIn
249 AntIn
249 AntIn
251 AntIn
251 AntOut
252 AntOut
252 AntIn
252 AntIn
253 AntOut
253 AntOut
254 AntIn
254 AntIn
255 AntOut
256 AntOut
256 AntOut
259 AntIn
259 AntIn
260 AntIn
260 AntIn
260 AntIn
261 AntIn
262 AntOut
262 AntOut
264 AntIn

264	AntOut
265	AntIn
265	AntIn
265	AntIn
266	AntOut
266	AntOut
267	AntOut
269	AntIn
270	AntOut
270	AntOut
272	AntIn
273	AntIn
273	AntIn
275	AntIn
275	AntIn
276	AntIn
278	AntOut
278	AntOut
278	AntOut
280	AntOut
281	AntIn
284	AntOut
285	AntIn
285	AntIn
286	AntOut
287	AntIn
288	AntOut
289	AntIn
289	AntOut
290	AntIn
291	AntOut
291	AntOut
292	AntOut
292	AntOut
293	AntOut
293	AntOut
293	AntOut
294	AntIn
294	AntIn
295	AntOut
295	AntIn
296	AntIn

297 AntIn
297 AntIn
300 AntIn
301 AntIn
301 AntIn
301 AntIn
301 AntOut
302 AntIn
303 AntOut
304 AntIn
304 AntIn
305 AntIn
307 AntOut
307 AntOut
308 AntIn
309 AntOut
309 AntIn
311 AntIn
312 AntOut
313 AntIn
313 AntIn
314 AntIn
315 AntIn
315 AntIn
315 AntIn
316 AntOut
316 AntOut
316 AntOut
318 AntIn
318 AntOut
321 AntOut
321 AntIn
321 AntIn
322 AntOut
323 AntOut
324 AntOut
325 AntIn
325 AntIn
325 AntIn
326 AntOut
326 AntOut
326 AntOut

326	AntOut
328	AntOut
328	AntIn
330	AntIn
330	AntIn
331	AntOut
331	AntOut
333	AntOut
333	AntOut
333	AntOut
334	AntIn
335	AntIn
335	AntIn
337	AntIn
337	AntIn
337	AntIn
338	AntIn
340	AntIn
342	AntOut
342	AntIn
342	AntIn
343	AntIn
345	AntOut
346	AntOut
346	AntOut
348	AntOut
349	AntIn
351	AntIn
351	AntOut
352	AntIn
353	AntOut
353	AntOut
355	AntOut
356	AntOut
356	AntOut
357	AntOut
358	AntOut
358	AntIn
359	AntIn
360	AntIn
360	AntIn
360	AntIn

369	AntIn
369	AntIn
370	AntIn
370	AntIn
371	AntOut
372	AntIn
372	AntIn
372	AntOut
373	AntOut
373	AntIn
374	AntIn
376	AntIn
376	AntIn
377	AntIn
378	AntIn
378	AntIn
379	AntIn
379	AntIn
380	AntOut
381	AntOut
381	AntOut
382	AntIn
382	AntOut
383	AntOut
384	AntIn
385	AntOut
386	AntOut
386	AntOut
386	AntIn
387	AntIn
388	AntOut
388	AntOut
389	AntOut
390	AntIn
391	AntIn
391	AntIn
394	AntIn
395	AntIn
395	AntOut
397	AntIn
397	AntOut
397	AntIn

398 AntOut
399 AntIn
399 AntIn
400 AntIn
400 AntIn
401 AntIn
401 AntIn
402 AntOut
403 AntOut
405 AntOut
405 AntOut
406 AntOut
406 AntIn
407 AntIn
407 AntOut
408 AntOut
412 AntOut
412 AntOut
412 AntOut
414 AntOut
414 AntIn
415 AntOut
415 AntIn
417 AntOut
417 AntOut
419 AntIn
419 AntIn
420 AntOut
420 AntIn
421 AntIn
423 AntIn
423 AntIn
423 AntIn
425 AntIn
426 AntIn
426 AntOut
427 AntIn
428 AntOut
429 AntIn
429 AntOut
430 AntIn
431 AntIn

433	AntOut
433	AntOut
433	AntIn
434	AntIn
435	AntIn
435	AntIn
436	AntOut
437	AntIn
437	AntOut
438	AntIn
439	AntIn
440	AntOut
440	AntOut
440	AntOut
441	AntIn
441	AntIn
441	AntOut
442	AntIn
443	AntOut
444	AntOut
444	AntOut
444	AntIn
445	AntIn
447	AntOut
448	AntIn
450	AntOut
451	AntIn
452	AntIn
452	AntIn
454	AntOut
455	AntIn
456	AntIn
456	AntOut
456	AntIn
457	AntOut
458	AntOut
458	AntOut
459	AntIn
460	AntIn
461	AntIn
461	AntIn
462	AntIn

463 AntOut
465 AntIn
466 AntOut
466 AntIn
469 AntIn
469 AntIn
470 AntOut
472 AntIn
472 AntIn
473 AntIn
473 AntIn
477 AntIn
477 AntIn
478 AntIn
478 AntOut
479 AntOut
482 AntIn
482 AntIn
485 AntIn
486 AntIn
486 AntIn
490 AntOut
491 AntIn
492 AntIn
493 AntIn
493 AntIn
494 AntIn
496 AntIn
496 AntIn
497 AntIn
498 AntIn
498 AntIn
499 AntIn
501 AntIn
504 AntIn
505 AntIn
505 AntOut
506 AntIn
506 AntIn
508 AntOut
510 AntOut
511 AntIn

511 AntIn
516 AntOut
517 AntOut
517 AntOut
517 AntOut
518 AntOut
520 AntIn
521 AntOut
523 AntIn
525 AntIn
525 AntIn
526 AntOut
528 AntIn
529 AntIn
529 AntIn
529 AntIn
530 AntIn
530 AntIn
530 AntIn
531 AntOut
533 AntIn
534 AntOut
535 AntIn
535 AntIn
541 AntIn
543 AntIn
545 AntIn
547 AntIn
553 AntIn
553 AntIn
554 AntIn
554 AntIn
557 AntIn
558 AntIn
558 AntIn
560 AntIn
562 AntOut
564 AntIn
564 AntIn
565 AntOut
568 AntIn
569 AntOut

570 AntIn
571 AntIn
571 AntIn
574 AntIn
574 AntIn
575 AntIn
576 AntIn
576 AntIn
578 AntIn
584 AntIn
585 AntIn
587 AntIn
590 AntOut
591 AntIn
592 AntIn
596 AntIn
597 AntIn
597 AntIn
598 AntIn
598 AntIn
599 AntIn
599 AntIn
600 AntIn
603 AntIn
604 AntIn
605 AntIn
607 AntOut
610 AntIn
611 AntIn
612 AntIn
615 AntOut
618 AntIn
618 AntOut
621 AntOut
623 AntIn
624 AntIn
629 AntIn
629 AntIn
630 AntIn
631 AntIn
632 AntIn
632 AntIn

634 AntOut
638 AntIn
639 AntIn
644 AntIn
644 AntOut
649 AntIn
649 AntIn
652 AntIn
653 AntIn
654 AntIn
654 AntIn
660 AntIn
663 AntIn
665 AntIn
669 AntOut
671 AntIn
679 AntIn
679 AntIn
682 AntIn
686 AntOut
689 AntIn
690 AntOut
690 AntOut
690 AntOut
697 AntIn
698 AntIn
699 AntOut
699 AntOut
700 AntOut
705 AntIn
705 AntIn
707 AntIn
708 AntIn
710 AntIn
713 AntOut
714 AntOut
715 AntOut
718 AntOut
719 AntOut
719 AntIn
726 AntIn
728 AntOut

728	AntOut
729	AntOut
730	AntIn
733	AntOut
733	AntIn
734	AntIn
738	AntIn
740	AntOut
743	AntOut
744	AntOut
745	AntIn
746	AntIn
749	AntOut
751	AntOut
754	AntIn
763	AntIn
766	AntIn
767	AntIn
768	AntIn
771	AntOut
771	AntIn
772	AntIn
773	AntIn
777	AntOut
777	AntIn
779	AntOut
780	AntIn
781	AntOut
782	AntIn
783	AntOut
787	AntIn
788	AntIn
791	AntIn
792	AntIn
794	AntOut
795	AntIn
796	AntOut
797	AntIn
801	AntIn
802	AntOut
802	AntIn
806	AntOut

807	AntIn
811	AntOut
813	AntIn
818	AntIn
819	AntOut
823	AntIn
824	AntIn
824	AntIn
825	AntOut
826	AntOut
836	AntIn
837	AntOut
841	AntIn
842	AntIn
844	AntIn
846	AntIn
848	AntIn
849	AntOut
849	AntIn
851	AntIn
853	AntOut
862	AntOut
865	AntOut
867	AntOut
870	AntIn
871	AntIn
875	AntIn
877	AntOut
878	AntIn
883	AntOut
884	AntOut
887	AntIn
888	AntIn
889	AntOut
889	AntIn
890	AntIn
890	AntIn
893	AntIn
894	AntIn
895	AntOut
896	AntOut
897	AntOut

898	AntIn
900	AntIn
901	AntOut
901	AntOut
902	AntIn
