# Supplementary material for: Interactions Increase Forager Availability and Activity in Harvester Ants
Source: PLoS One. 2015 Nov 5;10(11):e0141971. doi: 10.1371/journal.pone.0141971 (PMC4635008; doi:10.1371/journal.pone.0141971)
Supplement: S3 Dataset — We observed and filmed behavior inside the nest during and after forager removals. This dataset shows our counts made from the films of the numbers of returning and outgoing foragers at the nest entrance and the number of ascending and descending ants at all tunnel entrances. (ZIP) [file pone.0141971.s004.zip › S3 Dataset/2013 Correlation Data 868 8-21.pdf]

**Researcher**	**Jovel Queirolo**
**Colony 868**	
**8/21/13**	
**Video time (seconds)**	**Event**
8	Ascend
9	Ascend
9	Ascend
9	Ascend
9	Ascend
10	Descend
11	Descend
11	Descend
11	Descend
11	Descend
13	Ascend
13	Ascend
13	Ascend
13	Ascend
14	Ascend
14	Ascend
14	Ascend
15	Ascend
15	Ascend
16	Ascend
17	Descend
17	Descend
18	Ascend
18	Ascend
18	Ascend
18	Ascend
18	Ascend
19	Ascend
19	Ascend
19	Ascend
20	Ascend
20	Ascend
22	Descend
22	Descend
24	Ascend
24	Ascend
25	Ascend

25	Ascend
25	Ascend
25	Ascend
26	Ascend
26	Ascend
26	Ascend
26	Ascend
27	Ascend
27	Ascend
27	Ascend
28	Ascend
28	Ascend
28	Ascend
29	Ascend
29	Ascend
30	Ascend
30	Ascend
31	Ascend
31	Ascend
31	Ascend
32	Ascend
32	Ascend
32	Ascend
33	Descend
34	Descend
35	Ascend
35	Descend
35	Descend
35	Descend
36	Descend
37	Ascend
37	Ascend
37	Ascend
38	Ascend
39	Descend
39	Ascend
40	Ascend
40	Ascend
40	Ascend
41	Descend
41	Descend
41	Descend

42	Descend
42	Ascend
43	Ascend
43	Descend
45	Descend
46	Descend
46	Descend
46	Descend
47	Descend
47	Descend
47	Descend
48	Descend
48	Descend
49	Ascend
49	Ascend
49	Ascend
50	Ascend
51	Ascend
52	Ascend
52	Ascend
53	Ascend
53	Descend
54	Ascend
55	Descend
55	Descend
56	Descend
56	Descend
57	Ascend
58	Ascend
58	Ascend
58	Descend
59	Descend
60	Descend
60	Ascend
60	Ascend
61	Ascend
61	Ascend
61	Ascend
61	Ascend
62	Ascend
62	Ascend
64	Descend

65	Ascend
65	Ascend
66	Ascend
66	Ascend
66	Ascend
67	Ascend
67	Ascend
68	Ascend
68	Ascend
69	Ascend
69	Ascend
69	Ascend
70	Ascend
70	Descend
70	Descend
70	Descend
71	Descend
71	Descend
71	Descend
72	Ascend
72	Ascend
73	Ascend
73	Ascend
73	Ascend
73	Ascend
74	Descend
75	Descend
75	Descend
75	Descend
76	Ascend
76	Ascend
76	Ascend
76	Ascend
76	Ascend
77	Ascend
77	Ascend
77	Ascend
77	Ascend
78	Ascend
78	Ascend
78	Ascend
78	Ascend

79	Ascend
79	Ascend
79	Ascend
79	Ascend
80	Ascend
80	Descend
80	Descend
81	Descend
81	Descend
81	Descend
81	Ascend
82	Ascend
82	Ascend
82	Ascend
83	Ascend
83	Ascend
84	Ascend
84	Ascend
85	Ascend
85	Ascend
86	Ascend
86	Ascend
86	Ascend
87	Ascend
87	Ascend
88	Descend
88	Descend
88	Descend
88	Descend
89	Descend
89	Descend
89	Ascend
90	Ascend
90	Ascend
90	Ascend
91	Ascend
92	Descend
92	Descend
92	Descend
92	Descend
92	Descend
93	Descend

93	Descend
93	Descend
93	Descend
94	Descend
94	Descend
94	Descend
94	Ascend
95	Ascend
95	Ascend
95	Ascend
96	Descend
97	Descend
98	Ascend
98	Ascend
99	Ascend
99	Ascend
99	Ascend
100	Ascend
101	Ascend
102	Descend
102	Descend
102	Descend
103	Descend
103	Descend
103	Descend
104	Ascend
104	Ascend
105	Ascend
105	Ascend
106	Ascend
106	Ascend
106	Ascend
107	Ascend
107	Ascend
108	Ascend
108	Ascend
109	Ascend
109	Ascend
109	Ascend
110	Ascend
110	Ascend
110	Ascend

111	Ascend
111	Ascend
111	Ascend
112	Descend
112	Descend
112	Descend
113	Ascend
113	Ascend
113	Ascend
114	Ascend
114	Ascend
115	Ascend
115	Descend
116	Descend
116	Descend
116	Descend
116	Descend
117	Descend
117	Descend
117	Descend
117	Descend
118	Descend
118	Descend
118	Descend
118	Descend
119	Descend
119	Descend
120	Ascend
120	Ascend
120	Ascend
120	Ascend
121	Ascend
121	Ascend
121	Ascend
121	Ascend
122	Ascend
122	Ascend
124	Ascend
126	Descend
127	Descend
127	Descend
128	Descend

128	Descend
128	Ascend
128	Ascend
129	Ascend
129	Ascend
130	Ascend
131	Descend
131	Descend
131	Descend
132	Ascend
132	Ascend
133	Ascend
133	Ascend
133	Ascend
133	Ascend
134	Descend
134	Descend
134	Descend
134	Descend
135	Ascend
135	Ascend
135	Ascend
135	Ascend
136	Descend
136	Descend
136	Descend
137	Descend
137	Descend
137	Descend
138	Descend
138	Descend
138	Descend
138	Descend
139	Descend
139	Ascend
139	Ascend
140	Ascend
140	Ascend
140	Ascend
140	Ascend
141	Descend
141	Descend

141	Descend
141	Descend
142	Descend
142	Ascend
142	Ascend
142	Ascend
143	Ascend
143	Ascend
143	Ascend
144	Ascend
144	Ascend
144	Descend
144	Descend
145	Descend
145	Descend
145	Descend
145	Ascend
146	Ascend
146	Ascend
146	Ascend
146	Ascend
146	Ascend
147	Ascend
147	Ascend
147	Descend
147	Descend
148	Descend
148	Descend
148	Descend
148	Descend
149	Descend
149	Descend
149	Descend
149	Descend
149	Ascend
150	Ascend
150	Ascend
150	Descend
150	Descend
150	Descend
151	Descend
151	Descend

152	Descend
152	Ascend
152	Ascend
153	Ascend
153	Ascend
153	Ascend
153	Ascend
154	Ascend
154	Ascend
154	Ascend
154	Ascend
155	Ascend
155	Ascend
155	Ascend
156	Ascend
156	Ascend
156	Ascend
157	Descend
157	Descend
157	Descend
157	Descend
158	Descend
158	Descend
158	Descend
159	Descend
159	Ascend
159	Ascend
159	Ascend
160	Ascend
160	Ascend
160	Ascend
161	Descend
161	Descend
161	Descend
161	Descend
163	Ascend
163	Ascend
163	Ascend
163	Ascend
164	Ascend
164	Ascend
164	Ascend

164	Descend
165	Descend
165	Descend
165	Descend
165	Descend
166	Descend
166	Descend
166	Ascend
166	Ascend
167	Ascend
167	Descend
167	Descend
167	Descend
167	Descend
168	Descend
168	Ascend
168	Ascend
168	Ascend
169	Ascend
169	Ascend
169	Ascend
169	Descend
170	Descend
170	Descend
170	Descend
170	Descend
171	Descend
171	Ascend
171	Ascend
172	Ascend
172	Ascend
172	Descend
173	Descend
173	Descend
173	Descend
174	Ascend
174	Ascend
174	Ascend
175	Ascend
175	Ascend
175	Ascend
176	Ascend

176	Ascend
176	Ascend
177	Ascend
177	Ascend
177	Ascend
178	Ascend
178	Ascend
178	Ascend
178	Ascend
179	Descend
179	Descend
179	Descend
180	Descend
180	Descend
180	Descend
180	Descend
181	Descend
181	Descend
181	Ascend
182	Ascend
182	Ascend
182	Descend
182	Descend
183	Descend
183	Descend
183	Descend
183	Descend
184	Descend
184	Ascend
184	Ascend
184	Ascend
186	Descend
186	Descend
186	Descend
186	Descend
187	Descend
187	Descend
187	Descend
187	Descend
188	Descend
188	Descend
188	Descend

188	Descend
189	Descend
189	Descend
189	Descend
190	Ascend
190	Ascend
191	Ascend
191	Ascend
192	Ascend
192	Ascend
192	Ascend
193	Ascend
193	Ascend
193	Ascend
194	Ascend
194	Ascend
194	Ascend
195	Ascend
195	Ascend
196	Descend
196	Descend
196	Descend
196	Descend
197	Descend
197	Descend
197	Descend
197	Ascend
197	Ascend
198	Ascend
199	Descend
199	Ascend
200	Ascend
200	Ascend
200	Ascend
201	Ascend
201	Descend
201	Descend
201	Descend
202	Descend
202	Descend
202	Ascend
203	Ascend

203	Ascend
204	Descend
205	Descend
205	Descend
205	Descend
205	Descend
206	Descend
206	Descend
206	Descend
206	Descend
207	Descend
207	Descend
207	Ascend
207	Ascend
208	Ascend
208	Ascend
208	Ascend
208	Ascend
209	Ascend
209	Ascend
210	Descend
210	Descend
210	Descend
211	Descend
211	Descend
211	Descend
211	Descend
212	Descend
212	Descend
212	Descend
212	Ascend
213	Ascend
213	Ascend
213	Ascend
213	Ascend
213	Descend
214	Descend
215	Ascend
215	Ascend
215	Ascend
215	Ascend
216	Ascend

216	Ascend
216	Ascend
216	Ascend
217	Ascend
217	Ascend
219	Ascend
220	Ascend
220	Ascend
221	Ascend
222	Descend
222	Descend
223	Descend
223	Descend
223	Ascend
225	Descend
225	Ascend
225	Ascend
225	Ascend
226	Descend
226	Descend
226	Ascend
227	Ascend
228	Ascend
228	Ascend
228	Ascend
229	Ascend
229	Ascend
230	Ascend
230	Ascend
231	Ascend
232	Ascend
232	Ascend
233	Ascend
233	Ascend
233	Ascend
234	Ascend
234	Ascend
234	Ascend
235	Ascend
235	Ascend
235	Ascend
236	Descend

236	Descend
236	Descend
236	Descend
236	Descend
236	Descend
236	Descend
237	Descend
237	Ascend
237	Ascend
238	Ascend
238	Ascend
238	Ascend
239	Ascend
239	Ascend
239	Ascend
239	Ascend
240	Ascend
240	Ascend
240	Ascend
240	Ascend
241	Ascend
241	Descend
241	Descend
241	Ascend
242	Ascend
242	Descend
242	Descend
242	Ascend
243	Ascend
243	Descend
243	Descend
243	Ascend
243	Ascend
244	Ascend
244	Ascend
244	Ascend
245	Ascend
245	Ascend
245	Descend
245	Descend
246	Descend
246	Descend

247	Descend
247	Descend
248	Descend
249	Ascend
249	Ascend
249	Ascend
249	Ascend
250	Ascend
251	Ascend
251	Descend
252	Descend
253	Ascend
253	Ascend
254	Ascend
254	Ascend
254	Ascend
254	Ascend
255	Descend
255	Descend
255	Descend
255	Descend
256	Descend
256	Descend
256	Descend
256	Descend
257	Descend
257	Descend
257	Descend
257	Descend
258	Descend
258	Descend
258	Descend
259	Ascend
259	Ascend
259	Ascend
259	Ascend
260	Ascend
260	Ascend
260	Ascend
261	Ascend
261	Ascend
261	Ascend

261 Ascend
262 Ascend
262 Descend
262 Descend
262 Descend
263 Descend
263 Descend
263 Descend
264 Descend
264 Descend
264 Ascend
264 Ascend
265 Descend
265 Descend
265 Descend
265 Descend
266 Descend
266 Descend
267 Descend
267 Descend
267 Ascend
267 Ascend
268 Ascend
268 Ascend
269 Ascend
269 Ascend
269 Ascend
270 Ascend
270 Ascend
270 Descend
270 Descend
271 Descend
271 Descend
271 Ascend
271 Ascend
272 Ascend
272 Ascend
272 Ascend
272 Descend
273 Descend
273 Descend
273 Descend

274	Descend
274	Descend
274	Ascend
274	Ascend
275	Descend
276	Descend
276	Descend
277	Descend
278	Descend
279	Descend
279	Descend
279	Descend
279	Ascend
279	Ascend
280	Ascend
280	Ascend
281	Ascend
281	Ascend
281	Ascend
282	Ascend
282	Ascend
282	Ascend
282	Ascend
283	Ascend
283	Ascend
283	Ascend
283	Ascend
284	Descend
284	Descend
284	Descend
285	Descend
285	Ascend
285	Ascend
286	Descend
286	Descend
286	Descend
287	Ascend
287	Ascend
288	Ascend
288	Ascend
288	Ascend
288	Ascend

289	Ascend
289	Descend
290	Descend
290	Descend
291	Descend
291	Descend
291	Ascend
292	Ascend
292	Ascend
292	Descend
292	Ascend
292	Ascend
293	Ascend
293	Ascend
293	Ascend
293	Ascend
294	Descend
294	Descend
294	Descend
295	Descend
296	Ascend
296	Ascend
296	Ascend
296	Ascend
296	Ascend
297	Ascend
297	Ascend
297	Descend
297	Descend
298	Descend
298	Ascend
298	Ascend
298	Ascend
298	Ascend
299	Ascend
299	Ascend
299	Descend
300	Descend
300	Descend
300	Descend
301	Ascend
301	Ascend

301	Descend
302	Descend
302	Descend
302	Descend
303	Descend
303	Ascend
304	Descend
304	Descend
305	Descend
306	Ascend
307	Ascend
307	Descend
307	Descend
308	Ascend
308	Ascend
308	Ascend
309	Ascend
309	Ascend
309	Ascend
310	Ascend
311	Descend
311	Descend
311	Ascend
312	Ascend
313	Descend
313	Descend
313	Descend
313	Ascend
314	Ascend
314	Ascend
314	Descend
315	Ascend
315	Descend
317	Descend
317	Descend
317	Ascend
317	Ascend
318	Descend
318	Descend
318	Descend
319	Descend
319	Descend

320	Ascend
320	Ascend
320	Ascend
320	Ascend
321	Ascend
321	Ascend
321	Ascend
321	Ascend
322	Ascend
322	Ascend
322	Descend
323	Descend
323	Descend
323	Descend
324	Descend
324	Descend
325	Descend
325	Descend
325	Descend
326	Descend
326	Descend
327	Ascend
328	Ascend
329	Descend
330	Descend
330	Descend
330	Descend
330	Descend
331	Descend
331	Descend
332	Descend
332	Descend
332	Descend
333	Descend
333	Descend
333	Descend
334	Descend
334	Descend
334	Descend
335	Descend
335	Descend
336	Descend

336	Descend
336	Descend
336	Descend
337	Descend
337	Descend
337	Descend
337	Descend
338	Descend
338	Descend
339	Descend
339	Descend
339	Ascend
340	Ascend
340	Ascend
340	Descend
340	Descend
340	Ascend
341	Ascend
341	Ascend
341	Ascend
341	Ascend
341	Ascend
342	Ascend
342	Ascend
342	Ascend
343	Descend
343	Descend
343	Descend
343	Descend
343	Descend
344	Ascend
344	Ascend
344	Ascend
344	Ascend
345	Ascend
345	Ascend
345	Ascend
345	Descend
345	Descend
346	Ascend
346	Ascend
346	Ascend

346	Descend
346	Descend
347	Descend
347	Ascend
347	Descend
348	Descend
348	Ascend
348	Descend
348	Descend
349	Descend
349	Descend
349	Descend
349	Descend
349	Descend
350	Descend
350	Descend
350	Ascend
351	Ascend
351	Ascend
351	Descend
351	Ascend
352	Ascend
352	Ascend
352	Ascend
352	Ascend
353	Ascend
353	Ascend
354	Ascend
355	Descend
355	Ascend
356	Descend
356	Descend
356	Descend
356	Descend
357	Descend
357	Descend
357	Descend
357	Descend
358	Descend
358	Descend
358	Descend
359	Descend

359	Descend
359	Descend
359	Descend
360	Ascend
360	Ascend
360	Ascend
361	Ascend
361	Ascend
362	Ascend
363	Descend
364	Descend
364	Descend
364	Descend
364	Descend
364	Ascend
365	Ascend
365	Ascend
365	Ascend
365	Ascend
365	Ascend
366	Ascend
366	Ascend
367	Descend
367	Ascend
367	Ascend
368	Descend
368	Descend
368	Descend
370	Ascend
370	Ascend
370	Ascend
370	Ascend
371	Ascend
371	Ascend
371	Ascend
371	Ascend
371	Ascend
372	Ascend
372	Ascend
372	Ascend
372	Ascend
373	Ascend

373	Ascend
373	Ascend
373	Descend
373	Descend
373	Descend
374	Descend
374	Descend
375	Descend
375	Ascend
375	Ascend
375	Ascend
375	Ascend
376	Ascend
376	Ascend
376	Ascend
376	Ascend
377	Ascend
377	Descend
377	Descend
377	Descend
377	Descend
378	Descend
378	Descend
378	Descend
378	Descend
378	Descend
379	Descend
379	Descend
379	Descend
379	Ascend
380	Ascend
380	Ascend
380	Ascend
380	Ascend
380	Descend
381	Descend
381	Descend
381	Descend
381	Descend
382	Ascend
382	Ascend
382	Ascend

382	Ascend
382	Ascend
383	Ascend
383	Ascend
383	Ascend
383	Ascend
384	Ascend
384	Ascend
384	Ascend
384	Ascend
385	Ascend
385	Ascend
385	Descend
385	Descend
386	Descend
386	Descend
386	Ascend
386	Ascend
386	Descend
387	Descend
387	Ascend
387	Ascend
387	Ascend
387	Descend
388	Ascend
388	Ascend
388	Ascend
388	Descend
389	Ascend
389	Ascend
389	Ascend
389	Ascend
390	Ascend
390	Ascend
390	Descend
390	Descend
390	Descend
391	Descend
391	Descend
391	Descend
391	Descend
392	Descend

392	Ascend
392	Ascend
392	Ascend
392	Ascend
393	Descend
393	Descend
393	Descend
393	Ascend
394	Descend
394	Descend
394	Descend
394	Ascend
395	Descend
395	Descend
396	Descend
396	Descend
396	Descend
397	Ascend
397	Ascend
397	Ascend
397	Ascend
398	Ascend
398	Ascend
398	Ascend
398	Ascend
398	Ascend
399	Ascend
399	Ascend
399	Ascend
399	Descend
400	Descend
400	Descend
400	Descend
400	Descend
400	Descend
400	Descend
400	Descend
401	Descend
401	Descend
402	Ascend
403	Ascend
403	Descend

403	Descend
403	Ascend
403	Descend
404	Descend
404	Descend
404	Descend
404	Ascend
404	Ascend
405	Ascend
405	Ascend
405	Ascend
405	Ascend
406	Ascend
406	Ascend
406	Ascend
406	Ascend
407	Ascend
407	Descend
408	Descend
408	Descend
408	Ascend
409	Ascend
409	Ascend
409	Ascend
409	Ascend
410	Ascend
410	Ascend
410	Ascend
411	Ascend
411	Ascend
411	Ascend
411	Ascend
411	Ascend
412	Ascend
412	Ascend
412	Ascend
412	Descend
413	Descend
413	Descend
413	Descend
413	Descend
414	Descend

414	Descend
414	Ascend
414	Ascend
414	Ascend
414	Ascend
415	Ascend
415	Ascend
415	Ascend
415	Ascend
416	Ascend
416	Ascend
416	Descend
416	Descend
417	Ascend
417	Ascend
417	Ascend
417	Ascend
418	Ascend
418	Ascend
418	Ascend
419	Ascend
419	Ascend
421	Descend
421	Descend
422	Descend
422	Descend
423	Descend
423	Descend
423	Descend
423	Descend
423	Descend
424	Descend
424	Descend
424	Descend
424	Descend
425	Descend
425	Descend
425	Descend
426	Descend
426	Descend
426	Descend
426	Descend

427	Descend
427	Descend
427	Descend
427	Descend
428	Descend
428	Descend
430	Descend
430	Descend
430	Descend
431	Descend
431	Descend
431	Descend
431	Descend
431	Descend
432	Descend
432	Descend
432	Descend
432	Descend
433	Descend
433	Descend
433	Descend
434	Descend
434	Descend
434	Descend
434	Descend
434	Descend
435	Descend
435	Descend
435	Descend
435	Descend
435	Descend
436	Descend
436	Descend
436	Descend
436	Descend
437	Descend
437	Descend
437	Descend
438	Descend
438	Descend
438	Descend
438	Descend

439	Descend
439	Descend
439	Descend
439	Descend
440	Descend
440	Descend
440	Descend
441	Descend
441	Descend
441	Descend
441	Descend
442	Descend
442	Descend
442	Descend
443	Descend
443	Descend
443	Descend
443	Descend
444	Descend
444	Descend
444	Descend
444	Descend
445	Descend
445	Descend
445	Descend
446	Descend
446	Descend
446	Descend
446	Descend
447	Descend
447	Descend
447	Descend
448	Descend
449	Descend
449	Descend
450	Descend
450	Descend
450	Descend
451	Descend
451	Descend
451	Descend
451	Descend

452	Descend
452	Descend
452	Descend
453	Descend
453	Descend
453	Descend
454	Descend
454	Descend
454	Descend
454	Descend
455	Descend
455	Descend
455	Descend
456	Descend
457	Descend
457	Descend
458	Descend
458	Descend
458	Descend
459	Ascend
459	Ascend
459	Ascend
460	Ascend
460	Descend
460	Descend
461	Descend
461	Descend
461	Descend
464	Descend
464	Descend
464	Descend
465	Descend
465	Descend
467	Descend
467	Descend
468	Descend
468	Descend
469	Descend
469	Descend
469	Descend
469	Descend
470	Descend

470	Descend
470	Descend
470	Descend
471	Descend
471	Descend
471	Descend
471	Descend
472	Descend
472	Descend
472	Descend
473	Descend
473	Descend
473	Descend
474	Descend
474	Descend
474	Descend
474	Descend
475	Ascend
475	Ascend
475	Ascend
476	Ascend
476	Ascend
476	Ascend
477	Ascend
477	Descend
477	Descend
477	Descend
478	Descend
478	Descend
478	Descend
478	Descend
479	Descend
479	Descend
479	Descend
480	Descend
480	Descend
480	Descend
480	Descend
481	Descend
481	Descend
481	Descend
481	Descend

482	Descend
482	Descend
482	Descend
483	Descend
483	Descend
483	Descend
484	Descend
484	Descend
484	Descend
485	Descend
485	Descend
485	Descend
486	Descend
487	Ascend
487	Ascend
488	Ascend
488	Ascend
488	Ascend
488	Ascend
489	Ascend
489	Ascend
489	Ascend
490	Ascend
491	Descend
491	Descend
492	Descend
492	Descend
492	Descend
493	Descend
493	Descend
493	Descend
494	Descend
495	Descend
495	Descend
496	Descend
496	Descend
496	Descend
496	Descend
497	Descend
497	Descend
497	Descend
498	Descend

498	Descend
499	Ascend
499	Ascend
499	Ascend
500	Ascend
500	Ascend
501	Ascend
501	Ascend
502	Ascend
502	Ascend
503	Descend
503	Descend
503	Descend
503	Descend
504	Descend
504	Descend
504	Descend
506	Descend
506	Descend
506	Descend
507	Descend
507	Descend
507	Descend
507	Descend
508	Descend
508	Descend
508	Descend
508	Descend
509	Descend
510	Descend
510	Descend
511	Descend
511	Descend
512	Descend
512	Descend
513	Descend
514	Descend
514	Descend
514	Descend
515	Descend
517	Descend
518	Descend

519	Descend
519	Descend
519	Descend
519	Descend
519	Descend
520	Descend
520	Descend
520	Descend
521	Descend
522	Ascend
523	Ascend
523	Ascend
524	Ascend
524	Ascend
525	Ascend
525	Ascend
527	Ascend
527	Descend
528	Descend
531	Descend
531	Descend
531	Ascend
531	Ascend
532	Ascend
532	Descend
532	Descend
533	Descend
533	Descend
533	Descend
534	Descend
534	Descend
534	Descend
535	Descend
539	Descend
539	Descend
543	Descend
543	Descend
543	Descend
543	Descend
544	Descend
544	Descend
544	Descend

545	Descend
546	Descend
546	Descend
547	Descend
547	Descend
547	Ascend
548	Ascend
548	Ascend
548	Descend
549	Descend
551	Descend
551	Descend
553	Descend
553	Descend
554	Descend
554	Descend
554	Descend
554	Descend
555	Descend
555	Descend
555	Descend
556	Descend
556	Descend
557	Descend
557	Descend
559	Descend
559	Descend
563	Descend
563	Descend
564	Descend
565	Descend
566	Descend
567	Descend
572	Descend
573	Descend
582	Descend
583	Descend
584	Descend
584	Descend
584	Descend
586	Descend
586	Descend

588	Descend
589	Descend
593	Ascend
593	Ascend
594	Ascend
595	Descend
595	Descend
596	Descend
596	Descend
598	Descend
598	Descend
601	Descend
601	Descend
604	Descend
604	Descend
607	Ascend
609	Descend
610	Descend
615	Descend
615	Descend
616	Descend
616	Descend
618	Descend
618	Descend
621	Descend
623	Ascend
623	Ascend
625	Ascend
627	Ascend
627	Ascend
628	Ascend
630	Descend
631	Descend
633	Descend
636	Descend
637	Ascend
638	Descend
641	Ascend
644	Descend
645	Descend
646	Descend
646	Descend

648	Descend
649	Ascend
650	Ascend
651	Descend
656	Descend
657	Descend
657	Descend
658	Descend
659	Descend
659	Descend
660	Descend
667	Descend
669	Descend
673	Descend
691	Descend
693	Descend
696	Descend
697	Descend
703	Descend
708	Descend
718	Descend
722	Descend
732	Descend
740	Descend
740	Descend
760	Descend
761	Descend
763	Ascend
765	Ascend
772	Descend
773	Ascend
775	Ascend
778	Ascend
780	Ascend
784	Descend
787	Descend
789	Descend
791	Descend
795	Descend
796	Descend
798	Ascend
799	Descend

800	Descend
804	Descend
805	Descend
807	Descend
809	Descend
820	Descend
820	Descend
821	Descend
822	Ascend
824	Ascend
825	Descend
826	Descend
827	Descend
831	Descend
831	Ascend
831	Ascend
832	Ascend
833	Ascend
836	Descend
837	Descend
841	Descend
851	Descend
851	Ascend
852	Descend
852	Descend
853	Descend
854	Descend
856	Ascend
858	Descend
859	Descend
860	Ascend
869	Descend
881	Ascend
885	Descend
888	Descend
889	Descend
890	Ascend
891	Ascend
892	Ascend
895	Descend
898	Descend
900	Descend

905	Descend
911	Ascend
915	Descend
916	Descend
917	Descend
918	Descend
918	Descend
919	Ascend
923	Descend
928	Descend
929	Descend
929	Descend
931	Descend
934	Descend
938	Ascend
952	Descend
953	Descend
957	Descend
974	Descend
976	Descend
979	Ascend
981	Ascend
987	Descend
993	Ascend
1000	Descend
1003	Ascend
1013	Descend
1016	Descend
1017	Descend
1019	Descend
1019	Descend
1020	Descend
1021	Descend
1022	Descend
1023	Descend
1024	Descend
1044	Ascend
1054	Descend
1058	Ascend
1070	Descend
1071	Descend
1073	Descend

1075	Descend
1075	Descend
1075	Descend
1076	Descend
1077	Descend
1077	Descend
1078	Descend
1082	Ascend
1082	Ascend
1084	Descend
1097	Descend
1101	Descend
1106	Ascend
1109	Descend
1113	Descend
1115	Descend
1119	Descend
1120	Descend
1122	Ascend
1127	Descend
1131	Descend
1134	Ascend
1135	Ascend
1139	Ascend
1142	Descend
1162	Descend
1169	Descend
1172	Descend
1173	Descend
1176	Descend
1181	Ascend
1182	Ascend
1182	Descend
1184	Ascend
1194	Ascend
1196	Descend
1197	Ascend
1198	Descend
1200	Descend
1210	Descend
1216	Descend
1220	Ascend

1224	Descend
1230	Descend
1233	Descend
1235	Descend
1239	Ascend
1242	Ascend
1246	Ascend
1248	Descend
1254	Ascend
1256	Ascend
12	AntOut
13	AntIn
14	AntIn
14	AntOut
14	AntOut
15	AntOut
16	AntIn
16	AntOut
16	AntOut
17	AntOut
18	AntOut
20	AntOut
20	AntIn
21	AntOut
22	AntIn
23	AntOut
23	AntOut
23	AntIn
24	AntIn
24	AntOut
25	AntOut
25	AntOut
25	AntIn
27	AntOut
27	AntIn
29	AntOut
29	AntIn
31	AntIn
31	AntIn
31	AntOut
31	AntOut
32	AntOut

33	AntOut
33	AntIn
34	AntOut
34	AntOut
36	AntIn
36	AntIn
37	AntIn
38	AntIn
38	AntOut
39	AntOut
40	AntIn
41	AntOut
41	AntOut
42	AntIn
43	AntOut
43	AntOut
45	AntIn
46	AntOut
47	AntIn
47	AntOut
47	AntOut
47	AntOut
48	AntOut
49	AntOut
50	AntOut
50	AntOut
50	AntOut
50	AntOut
51	AntOut
51	AntOut
52	AntOut
54	AntOut
54	AntIn
55	AntIn
55	AntIn
57	AntIn
58	AntOut
58	AntOut
59	AntOut
60	AntIn
60	AntIn
61	AntOut

61	AntOut
61	AntOut
62	AntOut
63	AntOut
63	AntIn
64	AntOut
64	AntOut
65	AntIn
66	AntIn
66	AntOut
67	AntOut
67	AntOut
68	AntIn
68	AntIn
69	AntOut
70	AntOut
70	AntOut
70	AntIn
71	AntOut
71	AntOut
72	AntIn
72	AntIn
73	AntOut
74	AntOut
75	AntOut
75	AntOut
75	AntOut
76	AntIn
76	AntIn
77	AntIn
78	AntOut
78	AntOut
79	AntIn
80	AntIn
80	AntIn
81	AntOut
81	AntIn
83	AntOut
83	AntOut
83	AntOut
84	AntIn
85	AntOut

85	AntOut
85	AntOut
86	AntIn
86	AntIn
87	AntIn
87	AntIn
88	AntIn
88	AntIn
89	AntIn
89	AntIn
90	AntIn
90	AntIn
91	AntOut
91	AntOut
91	AntOut
92	AntOut
92	AntIn
93	AntIn
93	AntOut
94	AntOut
94	AntIn
94	AntOut
95	AntOut
95	AntOut
95	AntOut
97	AntOut
97	AntOut
98	AntOut
98	AntOut
99	AntOut
100	AntOut
102	AntOut
102	AntIn
103	AntOut
103	AntOut
104	AntOut
104	AntOut
105	AntOut
105	AntIn
105	AntIn
106	AntIn
107	AntIn

107 AntIn
108 AntIn
108 AntIn
109 AntIn
110 AntIn
110 AntOut
111 AntIn
111 AntIn
112 AntOut
113 AntOut
113 AntOut
113 AntOut
113 AntOut
114 AntOut
114 AntOut
115 AntIn
115 AntIn
116 AntOut
116 AntOut
116 AntOut
116 AntOut
117 AntOut
117 AntOut
118 AntOut
118 AntIn
119 AntOut
119 AntOut
120 AntIn
121 AntOut
121 AntIn
121 AntIn
122 AntOut
124 AntOut
125 AntIn
126 AntIn
126 AntOut
127 AntOut
127 AntOut
127 AntOut
128 AntIn
128 AntIn
128 AntOut

130 AntOut
130 AntOut
131 AntOut
131 AntOut
132 AntOut
132 AntOut
133 AntIn
133 AntIn
134 AntOut
134 AntOut
137 AntOut
137 AntOut
138 AntIn
138 AntIn
140 AntOut
140 AntOut
141 AntOut
142 AntOut
144 AntIn
144 AntOut
144 AntOut
148 AntIn
148 AntIn
149 AntOut
149 AntOut
150 AntIn
151 AntOut
154 AntOut
154 AntOut
156 AntOut
156 AntOut
156 AntOut
157 AntIn
157 AntOut
158 AntOut
159 AntOut
160 AntOut
161 AntOut
161 AntOut
162 AntOut
162 AntOut
163 AntOut

163 AntOut
164 AntOut
166 AntOut
166 AntOut
167 AntOut
167 AntOut
171 AntIn
171 AntIn
171 AntIn
172 AntIn
172 AntIn
172 AntOut
172 AntIn
173 AntIn
173 AntIn
173 AntOut
173 AntOut
174 AntIn
175 AntIn
177 AntOut
177 AntOut
177 AntIn
178 AntOut
178 AntOut
179 AntOut
181 AntOut
181 AntIn
181 AntIn
182 AntIn
182 AntOut
183 AntOut
183 AntOut
184 AntOut
184 AntOut
185 AntOut
186 AntOut
187 AntIn
187 AntIn
188 AntOut
189 AntIn
190 AntOut
190 AntIn

191	AntIn
191	AntOut
193	AntIn
193	AntOut
193	AntOut
194	AntOut
194	AntIn
194	AntIn
195	AntIn
196	AntIn
196	AntIn
196	AntOut
196	AntOut
197	AntIn
199	AntIn
199	AntIn
199	AntOut
200	AntOut
200	AntOut
200	AntOut
202	AntOut
202	AntIn
202	AntIn
203	AntOut
203	AntOut
203	AntOut
204	AntOut
204	AntOut
204	AntOut
206	AntOut
206	AntOut
206	AntOut
206	AntOut
207	AntIn
207	AntIn
208	AntIn
208	AntIn
209	AntOut
209	AntOut
210	AntOut
210	AntOut
210	AntOut

211	AntOut
211	AntIn
212	AntIn
212	AntOut
213	AntOut
213	AntOut
213	AntIn
214	AntIn
214	AntIn
214	AntIn
215	AntOut
215	AntOut
215	AntOut
216	AntOut
217	AntOut
217	AntOut
217	AntIn
218	AntIn
219	AntIn
219	AntOut
219	AntOut
219	AntOut
220	AntIn
221	AntOut
222	AntOut
222	AntOut
222	AntOut
222	AntOut
223	AntOut
224	AntIn
225	AntIn
226	AntOut
226	AntOut
226	AntOut
226	AntOut
227	AntOut
227	AntOut
227	AntOut
228	AntIn
228	AntIn
229	AntOut
229	AntOut

229 AntOut
229 AntOut
230 AntOut
230 AntOut
231 AntIn
231 AntIn
231 AntIn
232 AntOut
232 AntOut
233 AntOut
233 AntIn
235 AntOut
235 AntOut
235 AntOut
235 AntOut
236 AntOut
236 AntOut
236 AntOut
237 AntOut
237 AntOut
237 AntOut
237 AntOut
237 AntIn
237 AntIn
238 AntIn
238 AntOut
238 AntOut
239 AntOut
239 AntOut
239 AntOut
239 AntOut
240 AntOut
240 AntIn
241 AntIn
241 AntIn
241 AntOut
242 AntOut
242 AntIn
243 AntOut
243 AntOut
243 AntIn
244 AntIn

244 AntOut
245 AntOut
245 AntOut
245 AntOut
245 AntOut
246 AntOut
246 AntIn
246 AntIn
247 AntIn
248 AntIn
248 AntIn
248 AntIn
249 AntIn
250 AntOut
250 AntOut
250 AntOut
251 AntIn
251 AntIn
251 AntOut
252 AntOut
252 AntIn
253 AntOut
253 AntOut
253 AntOut
254 AntOut
254 AntOut
255 AntOut
255 AntOut
256 AntOut
256 AntOut
256 AntOut
256 AntOut
257 AntOut
257 AntOut
257 AntIn
258 AntIn
258 AntIn
258 AntIn
259 AntIn
259 AntOut
260 AntOut
260 AntOut

260 AntOut
261 AntOut
261 AntIn
261 AntIn
263 AntIn
263 AntOut
263 AntOut
264 AntIn
264 AntIn
266 AntOut
266 AntOut
268 AntIn
268 AntOut
269 AntOut
269 AntOut
269 AntOut
270 AntOut
270 AntOut
270 AntOut
271 AntOut
271 AntIn
271 AntIn
272 AntIn
272 AntIn
272 AntIn
273 AntIn
273 AntIn
274 AntIn
277 AntIn
277 AntOut
278 AntOut
278 AntOut
278 AntIn
279 AntOut
279 AntOut
279 AntOut
280 AntOut
281 AntOut
281 AntOut
281 AntOut
282 AntIn
282 AntIn

283	AntOut
283	AntOut
284	AntOut
284	AntOut
284	AntOut
285	AntOut
285	AntOut
285	AntOut
285	AntOut
286	AntOut
286	AntOut
286	AntIn
287	AntOut
287	AntIn
287	AntIn
288	AntIn
288	AntIn
289	AntIn
290	AntOut
290	AntOut
290	AntOut
291	AntOut
291	AntOut
291	AntOut
292	AntIn
293	AntIn
294	AntOut
294	AntIn
295	AntIn
295	AntOut
295	AntOut
296	AntOut
296	AntOut
297	AntOut
298	AntIn
299	AntIn
300	AntIn
300	AntOut
300	AntOut
301	AntIn
302	AntOut
303	AntIn

303	AntIn
304	AntIn
304	AntIn
305	AntIn
306	AntIn
306	AntIn
307	AntOut
307	AntOut
308	AntIn
309	AntIn
309	AntIn
310	AntIn
310	AntOut
310	AntOut
311	AntOut
312	AntOut
312	AntOut
312	AntOut
313	AntOut
314	AntOut
314	AntOut
314	AntIn
314	AntIn
315	AntIn
315	AntOut
317	AntIn
319	AntOut
319	AntOut
319	AntOut
320	AntOut
320	AntOut
320	AntOut
322	AntOut
323	AntIn
323	AntIn
324	AntOut
324	AntIn
325	AntIn
325	AntOut
325	AntOut
325	AntOut
326	AntOut

326	AntIn
326	AntIn
327	AntOut
327	AntOut
327	AntOut
328	AntOut
328	AntIn
329	AntIn
329	AntIn
330	AntIn
330	AntIn
331	AntOut
331	AntOut
331	AntOut
331	AntOut
332	AntIn
332	AntOut
333	AntIn
333	AntIn
334	AntOut
334	AntOut
335	AntOut
335	AntIn
336	AntIn
336	AntOut
337	AntOut
337	AntOut
338	AntOut
339	AntOut
339	AntIn
339	AntIn
340	AntIn
340	AntIn
341	AntIn
341	AntOut
341	AntOut
342	AntIn
342	AntIn
342	AntIn
343	AntIn
343	AntOut
344	AntOut

345 AntIn
345 AntOut
346 AntOut
346 AntOut
348 AntOut
349 AntIn
353 AntOut
353 AntOut
353 AntOut
353 AntOut
353 AntIn
354 AntIn
354 AntIn
354 AntOut
355 AntOut
355 AntIn
356 AntIn
356 AntIn
356 AntOut
357 AntOut
357 AntIn
357 AntIn
358 AntIn
358 AntIn
359 AntIn
359 AntIn
360 AntIn
360 AntIn
360 AntOut
361 AntOut
361 AntIn
362 AntOut
362 AntOut
362 AntOut
362 AntIn
366 AntIn
367 AntIn
368 AntOut
368 AntOut
368 AntOut
369 AntOut
369 AntIn

369	AntIn
370	AntOut
370	AntOut
370	AntOut
370	AntOut
372	AntIn
372	AntOut
372	AntOut
373	AntOut
373	AntOut
373	AntOut
373	AntOut
374	AntOut
374	AntOut
375	AntIn
375	AntIn
375	AntIn
375	AntIn
375	AntIn
376	AntIn
376	AntIn
376	AntOut
377	AntIn
377	AntOut
378	AntOut
378	AntIn
378	AntIn
379	AntIn
379	AntIn
379	AntIn
380	AntOut
380	AntOut
380	AntOut
380	AntOut
381	AntIn
382	AntIn
382	AntOut
382	AntIn
383	AntOut
384	AntOut
384	AntOut
384	AntOut

385	AntIn
385	AntIn
386	AntOut
386	AntOut
386	AntIn
386	AntOut
387	AntIn
387	AntOut
387	AntOut
387	AntOut
387	AntOut
388	AntIn
389	AntIn
390	AntIn
390	AntIn
390	AntIn
390	AntOut
391	AntOut
391	AntOut
391	AntOut
391	AntIn
391	AntOut
391	AntIn
391	AntOut
392	AntOut
393	AntOut
393	AntOut
394	AntIn
394	AntIn
394	AntIn
395	AntIn
395	AntOut
396	AntOut
396	AntOut
396	AntOut
396	AntOut
397	AntIn
397	AntIn
398	AntOut
398	AntOut
399	AntIn
399	AntOut

399 AntOut
400 AntOut
400 AntOut
400 AntOut
400 AntOut
401 AntOut
401 AntOut
401 AntIn
402 AntIn
402 AntOut
402 AntOut
403 AntIn
404 AntIn
405 AntIn
405 AntIn
406 AntIn
406 AntIn
406 AntIn
406 AntIn
406 AntIn
407 AntIn
407 AntIn
407 AntOut
407 AntOut
408 AntOut
408 AntOut
408 AntIn
409 AntIn
409 AntOut
409 AntOut
410 AntIn
411 AntOut
411 AntOut
411 AntOut
411 AntOut
412 AntOut
412 AntOut
412 AntIn
412 AntIn
412 AntIn
413 AntIn
413 AntIn

413	AntIn
413	AntOut
414	AntOut
414	AntOut
415	AntOut
416	AntIn
416	AntIn
417	AntOut
417	AntIn
417	AntIn
417	AntOut
418	AntIn
419	AntIn
420	AntOut
421	AntIn
421	AntOut
421	AntOut
421	AntOut
423	AntIn
423	AntIn
423	AntIn
425	AntOut
426	AntOut
427	AntOut
427	AntIn
427	AntIn
428	AntIn
428	AntIn
428	AntIn
428	AntIn
429	AntIn
430	AntIn
430	AntIn
430	AntIn
430	AntIn
430	AntIn
431	AntIn
431	AntIn
431	AntIn
433	AntIn
433	AntIn
434	AntIn

436 AntIn
436 AntIn
438 AntIn
438 AntIn
438 AntOut
439 AntOut
439 AntIn
439 AntIn
439 AntIn
440 AntIn
440 AntIn
441 AntOut
441 AntIn
442 AntOut
442 AntIn
444 AntOut
444 AntOut
444 AntIn
445 AntIn
445 AntIn
445 AntIn
446 AntIn
446 AntIn
446 AntIn
446 AntIn
446 AntIn
447 AntIn
447 AntIn
447 AntIn
447 AntIn
448 AntIn
448 AntIn
448 AntIn
448 AntIn
449 AntIn
449 AntIn
449 AntIn
449 AntIn
449 AntIn
450 AntIn
450 AntIn
451 AntIn

451 AntIn
451 AntOut
451 AntIn
452 AntIn
452 AntIn
452 AntIn
452 AntIn
452 AntIn
453 AntOut
453 AntOut
453 AntIn
453 AntIn
453 AntIn
454 AntIn
454 AntIn
454 AntOut
454 AntIn
454 AntIn
455 AntIn
455 AntIn
455 AntIn
455 AntIn
456 AntIn
456 AntIn
457 AntIn
457 AntIn
457 AntIn
458 AntOut
458 AntOut
458 AntOut
458 AntOut
459 AntOut
459 AntIn
459 AntIn
460 AntIn
460 AntIn
460 AntIn
461 AntIn
461 AntOut
461 AntOut
461 AntOut
462 AntIn

462 AntIn
463 AntIn
463 AntIn
463 AntIn
463 AntIn
463 AntOut
463 AntOut
464 AntIn
464 AntIn
464 AntIn
464 AntIn
465 AntIn
465 AntIn
466 AntIn
467 AntIn
467 AntIn
467 AntIn
468 AntIn
468 AntIn
469 AntIn
470 AntOut
470 AntIn
470 AntOut
471 AntOut
472 AntIn
472 AntIn
473 AntIn
474 AntIn
475 AntIn
475 AntIn
476 AntOut
476 AntOut
477 AntIn
478 AntIn
478 AntOut
479 AntOut
480 AntOut
480 AntOut
480 AntIn
481 AntIn
481 AntIn
481 AntIn

482 AntIn
483 AntIn
484 AntIn
485 AntIn
485 AntIn
486 AntIn
487 AntOut
487 AntIn
488 AntIn
488 AntIn
489 AntIn
489 AntIn
490 AntIn
492 AntIn
493 AntIn
495 AntIn
496 AntIn
496 AntIn
497 AntIn
497 AntIn
499 AntIn
499 AntIn
500 AntIn
501 AntOut
502 AntOut
503 AntOut
504 AntOut
505 AntIn
506 AntIn
506 AntOut
507 AntIn
508 AntIn
510 AntIn
510 AntIn
511 AntIn
511 AntOut
511 AntIn
512 AntIn
513 AntIn
514 AntIn
515 AntOut
515 AntIn

516	AntIn
516	AntIn
517	AntIn
518	AntIn
518	AntIn
519	AntIn
519	AntIn
520	AntIn
521	AntIn
523	AntIn
523	AntIn
524	AntOut
525	AntIn
525	AntIn
526	AntIn
526	AntIn
526	AntIn
527	AntIn
528	AntIn
528	AntIn
529	AntIn
531	AntIn
532	AntIn
532	AntIn
532	AntIn
533	AntOut
534	AntOut
535	AntOut
536	AntIn
536	AntIn
536	AntIn
537	AntOut
538	AntOut
538	AntOut
539	AntIn
539	AntIn
539	AntIn
540	AntIn
540	AntIn
541	AntIn
542	AntIn
543	AntIn

543 AntIn
545 AntIn
547 AntIn
550 AntOut
551 AntIn
552 AntIn
553 AntIn
553 AntOut
555 AntIn
556 AntIn
557 AntIn
558 AntIn
558 AntIn
559 AntOut
560 AntIn
560 AntIn
560 AntIn
563 AntIn
564 AntIn
567 AntIn
568 AntIn
568 AntOut
568 AntIn
570 AntIn
571 AntIn
572 AntIn
572 AntIn
575 AntIn
577 AntOut
577 AntIn
578 AntIn
579 AntIn
581 AntIn
582 AntIn
583 AntIn
585 AntIn
588 AntIn
589 AntIn
589 AntIn
590 AntIn
594 AntOut
595 AntOut

601 AntIn
603 AntIn
605 AntIn
607 AntIn
609 AntIn
610 AntIn
611 AntIn
612 AntIn
619 AntIn
620 AntIn
625 AntIn
626 AntIn
627 AntIn
631 AntIn
632 AntIn
632 AntIn
634 AntOut
635 AntIn
637 AntIn
640 AntOut
640 AntIn
641 AntIn
641 AntIn
642 AntIn
642 AntIn
643 AntIn
645 AntOut
646 AntIn
647 AntOut
647 AntIn
649 AntIn
653 AntIn
653 AntIn
654 AntIn
654 AntIn
657 AntIn
658 AntIn
666 AntIn
667 AntIn
668 AntIn
670 AntIn
672 AntIn

681 AntIn
692 AntIn
694 AntIn
694 AntIn
696 AntIn
696 AntIn
698 AntIn
699 AntIn
699 AntIn
704 AntIn
713 AntIn
717 AntIn
719 AntIn
720 AntIn
728 AntIn
728 AntIn
734 AntIn
736 AntIn
736 AntIn
738 AntIn
741 AntIn
746 AntOut
747 AntIn
753 AntIn
753 AntIn
755 AntOut
756 AntIn
756 AntIn
757 AntIn
757 AntIn
762 AntIn
766 AntOut
773 AntIn
773 AntIn
775 AntIn
776 AntOut
777 AntIn
779 AntIn
780 AntOut
782 AntIn
783 AntIn
783 AntIn

783	AntIn
786	AntIn
790	AntIn
790	AntIn
792	AntIn
794	AntIn
794	AntIn
794	AntIn
795	AntIn
798	AntIn
799	AntOut
801	AntIn
802	AntIn
802	AntIn
802	AntIn
804	AntIn
812	AntIn
814	AntIn
819	AntIn
823	AntIn
823	AntIn
829	AntIn
831	AntIn
832	AntOut
832	AntIn
836	AntIn
842	AntIn
846	AntIn
846	AntIn
849	AntIn
853	AntIn
853	AntIn
858	AntIn
860	AntIn
862	AntIn
863	AntIn
864	AntIn
875	AntIn
877	AntIn
879	AntIn
881	AntIn
887	AntIn

892	AntOut
902	AntOut
903	AntIn
910	AntIn
912	AntIn
913	AntIn
913	AntIn
914	AntOut
914	AntIn
915	AntIn
918	AntIn
918	AntIn
919	AntIn
920	AntIn
920	AntIn
924	AntIn
925	AntIn
926	AntIn
926	AntIn
929	AntOut
943	AntIn
944	AntIn
947	AntIn
949	AntIn
956	AntIn
958	AntIn
959	AntOut
960	AntIn
966	AntIn
971	AntIn
972	AntOut
972	AntIn
976	AntIn
976	AntIn
978	AntOut
980	AntIn
983	AntIn
990	AntIn
993	AntIn
996	AntIn
998	AntIn
1001	AntIn

1009	AntIn
1010	AntIn
1010	AntIn
1011	AntIn
1012	AntIn
1014	AntIn
1015	AntIn
1018	AntIn
1018	AntIn
1031	AntIn
1033	AntIn
1037	AntIn
1043	AntIn
1045	AntIn
1052	AntIn
1057	AntIn
1064	AntIn
1066	AntIn
1067	AntIn
1067	AntIn
1069	AntIn
1070	AntIn
1073	AntIn
1077	AntIn
1081	AntIn
1085	AntIn
1087	AntIn
1088	AntIn
1091	AntIn
1093	AntIn
1096	AntIn
1096	AntIn
1114	AntIn
1120	AntIn
1124	AntIn
1128	AntIn
1132	AntIn
1133	AntIn
1158	AntIn
1159	AntIn
1164	AntIn
1168	AntIn

1168	AntIn
1169	AntIn
1170	AntIn
1182	AntIn
1192	AntIn
1204	AntIn
1205	AntOut
1205	AntIn
1208	AntIn
1213	AntOut
1219	AntIn
1220	AntIn
1233	AntIn
1233	AntIn
1236	AntIn
1238	AntIn
1241	AntIn
1247	AntOut
1253	AntOut
1255	AntIn
