# Supplementary material for: Interactions Increase Forager Availability and Activity in Harvester Ants
Source: PLoS One. 2015 Nov 5;10(11):e0141971. doi: 10.1371/journal.pone.0141971 (PMC4635008; doi:10.1371/journal.pone.0141971)
Supplement: S3 Dataset — We observed and filmed behavior inside the nest during and after forager removals. This dataset shows our counts made from the films of the numbers of returning and outgoing foragers at the nest entrance and the number of ascending and descending ants at all tunnel entrances. (ZIP) [file pone.0141971.s004.zip › S3 Dataset/2013 Correlation Data N5 8-17.pdf]

Researcher **Jovel Queirolo**
Colony N5
8/17/13

**Video time (seconds)**	**Event**
7	AntIn
7	AntIn
7	AntOut
8	AntIn
8	AntOut
8	AntOut
9	AntIn
9	AntIn
9	AntOut
9	AntOut
9	AntOut
9	AntOut
10	AntIn
10	AntOut
10	AntOut
10	AntOut
10	AntOut
10	AntOut
11	AntIn
11	AntOut
11	AntOut
12	AntIn
12	AntIn
12	AntOut
12	AntOut
13	AntIn
13	AntIn
13	AntOut
13	AntOut
13	AntOut
14	AntIn
14	AntIn
14	AntOut
15	AntIn
15	AntIn
15	AntIn
15	AntOut

15	AntOut
16	AntIn
16	AntIn
16	AntOut
16	AntOut
16	AntOut
16	AntOut
17	AntIn
17	AntIn
17	AntIn
17	AntOut
17	AntOut
18	AntIn
18	AntIn
18	AntOut
18	AntOut
18	AntOut
19	AntIn
19	AntOut
19	AntOut
19	AntOut
19	AntOut
20	AntIn
20	AntIn
20	AntOut
20	AntOut
20	AntOut
21	AntIn
21	AntIn
21	AntIn
21	AntIn
21	AntIn
21	AntIn
21	AntOut
21	AntOut
21	AntOut
22	AntIn
22	AntIn
22	AntIn
22	AntOut
22	AntOut
22	AntOut

23	AntOut
23	AntOut
24	AntIn
24	AntIn
24	AntOut
24	AntOut
24	AntOut
24	AntOut
24	AntOut
24	AntOut
24	AntOut
24	AntOut
25	AntIn
25	AntIn
25	AntIn
25	AntIn
26	AntIn
26	AntIn
26	AntIn
26	AntIn
27	AntIn
27	AntIn
27	AntOut
27	AntOut
27	AntOut
28	AntIn
28	AntIn
28	AntIn
28	AntIn
28	AntOut
28	AntOut
29	AntIn
29	AntIn
29	AntIn
29	AntIn
30	AntIn
30	AntIn
30	AntIn
31	AntIn
31	AntOut
31	AntOut
31	AntOut

31 AntOut
32 AntIn
32 AntIn
33 AntIn
33 AntIn
33 AntOut
33 AntOut
34 AntOut
34 AntOut
35 AntIn
35 AntIn
36 AntIn
36 AntOut
36 AntOut
36 AntOut
36 AntOut
36 AntOut
36 AntOut
37 AntIn
37 AntIn
37 AntIn
37 AntIn
37 AntOut
38 AntIn
38 AntIn
38 AntIn
38 AntOut
38 AntOut
38 AntOut
38 AntOut
38 AntOut
39 AntIn
39 AntIn
39 AntIn
39 AntIn
40 AntIn
40 AntIn
40 AntOut
40 AntOut
40 AntOut
41 AntIn
41 AntIn

41	AntIn
41	AntIn
41	AntOut
41	AntOut
41	AntOut
42	AntIn
42	AntIn
42	AntIn
42	AntOut
42	AntOut
43	AntIn
43	AntOut
43	AntOut
43	AntOut
43	AntOut
43	AntOut
44	AntOut
45	AntIn
45	AntIn
45	AntIn
45	AntIn
45	AntIn
45	AntIn
45	AntIn
45	AntOut
45	AntOut
46	AntIn
46	AntIn
46	AntOut
46	AntOut
46	AntOut
46	AntOut
47	AntOut
47	AntOut
47	AntOut
48	AntIn
49	AntIn
49	AntIn
49	AntOut
49	AntOut
49	AntOut
49	AntOut

49	AntOut
49	AntOut
50	AntIn
50	AntOut
50	AntOut
50	AntOut
50	AntOut
50	AntOut
51	AntIn
51	AntIn
51	AntIn
51	AntIn
51	AntOut
51	AntOut
52	AntIn
52	AntOut
52	AntOut
52	AntOut
52	AntOut
53	AntIn
53	AntIn
53	AntIn
53	AntOut
53	AntOut
53	AntOut
54	AntIn
54	AntIn
54	AntIn
54	AntIn
54	AntOut
55	AntIn
55	AntIn
55	AntIn
55	AntIn
55	AntOut
55	AntOut
56	AntOut
56	AntOut
57	AntIn
57	AntIn
58	AntIn
58	AntIn

58 AntOut
58 AntOut
59 AntOut
59 AntOut
59 AntOut
60 AntIn
60 AntIn
60 AntIn
60 AntOut
60 AntOut
61 AntIn
61 AntOut
62 AntIn
62 AntIn
62 AntIn
62 AntIn
62 AntIn
62 AntOut
63 AntIn
63 AntIn
63 AntIn
63 AntOut
64 AntIn
64 AntIn
64 AntIn
64 AntOut
64 AntOut
64 AntOut
65 AntIn
65 AntIn
65 AntOut
66 AntIn
66 AntIn
66 AntIn
66 AntOut
66 AntOut
67 AntIn
67 AntIn
67 AntOut
67 AntOut
68 AntIn
68 AntIn

68	AntOut
69	AntOut
69	AntOut
69	AntOut
70	AntIn
70	AntIn
70	AntOut
70	AntOut
71	AntIn
71	AntOut
72	AntIn
72	AntIn
72	AntIn
73	AntIn
73	AntIn
73	AntOut
73	AntOut
74	AntIn
74	AntOut
74	AntOut
74	AntOut
75	AntIn
75	AntIn
75	AntIn
75	AntIn
75	AntOut
76	AntOut
77	AntIn
77	AntOut
77	AntOut
77	AntOut
77	AntOut
78	AntIn
78	AntIn
78	AntOut
78	AntOut
79	AntIn
79	AntIn
79	AntIn
80	AntIn
80	AntOut
80	AntOut

80 AntOut
81 AntIn
81 AntOut
82 AntIn
82 AntIn
82 AntIn
83 AntIn
83 AntIn
83 AntIn
83 AntIn
83 AntOut
84 AntIn
84 AntOut
85 AntIn
85 AntIn
85 AntIn
85 AntIn
85 AntOut
86 AntIn
86 AntIn
86 AntOut
86 AntOut
87 AntIn
87 AntIn
88 AntIn
88 AntIn
88 AntIn
88 AntIn
88 AntIn
89 AntIn
89 AntOut
89 AntOut
90 AntIn
90 AntIn
90 AntOut
90 AntOut
91 AntIn
91 AntIn
91 AntOut
91 AntOut
91 AntOut
92 AntIn

92	AntIn
92	AntOut
92	AntOut
92	AntOut
92	AntOut
93	AntIn
93	AntIn
93	AntIn
93	AntIn
93	AntOut
93	AntOut
94	AntOut
94	AntOut
94	AntOut
94	AntOut
95	AntOut
95	AntOut
95	AntOut
95	AntOut
95	AntOut
96	AntIn
96	AntIn
96	AntIn
96	AntIn
97	AntIn
97	AntIn
97	AntOut
98	AntIn
98	AntIn
98	AntOut
99	AntIn
99	AntIn
99	AntIn
99	AntOut
100	AntIn
100	AntIn
101	AntIn
101	AntIn
101	AntIn
101	AntOut
101	AntOut
101	AntOut

101 AntOut
102 AntIn
102 AntIn
102 AntIn
102 AntIn
102 AntIn
102 AntOut
102 AntOut
103 AntIn
103 AntOut
103 AntOut
103 AntOut
103 AntOut
103 AntOut
103 AntOut
104 AntIn
104 AntIn
104 AntIn
104 AntOut
104 AntOut
105 AntIn
105 AntIn
105 AntOut
105 AntOut
105 AntOut
105 AntOut
105 AntOut
106 AntIn
106 AntIn
106 AntOut
106 AntOut
106 AntOut
107 AntIn
107 AntOut
107 AntOut
108 AntIn
108 AntIn
108 AntIn
109 AntOut
109 AntOut
109 AntOut
109 AntOut

110	AntIn
110	AntIn
110	AntOut
111	AntOut
111	AntOut
112	AntIn
112	AntOut
112	AntOut
113	AntIn
113	AntOut
113	AntOut
114	AntIn
114	AntIn
114	AntIn
114	AntOut
115	AntIn
115	AntIn
115	AntIn
115	AntIn
116	AntIn
116	AntOut
116	AntOut
116	AntOut
117	AntIn
117	AntIn
117	AntIn
117	AntOut
118	AntOut
119	AntOut
119	AntOut
119	AntOut
119	AntOut
119	AntOut
119	AntOut
120	AntIn
120	AntIn
120	AntOut
121	AntIn
121	AntIn
121	AntIn
121	AntIn
122	AntIn

122	AntOut
122	AntOut
123	AntOut
123	AntOut
123	AntOut
124	AntIn
124	AntOut
124	AntOut
124	AntOut
125	AntIn
125	AntIn
125	AntIn
125	AntIn
125	AntOut
125	AntOut
125	AntOut
126	AntIn
126	AntIn
127	AntIn
127	AntIn
127	AntIn
127	AntOut
127	AntOut
128	AntIn
128	AntIn
128	AntOut
128	AntOut
128	AntOut
128	AntOut
129	AntIn
129	AntIn
129	AntOut
130	AntIn
130	AntIn
130	AntIn
130	AntOut
130	AntOut
130	AntOut
131	AntIn
131	AntIn
131	AntIn
131	AntIn

131 AntOut
132 AntIn
132 AntIn
132 AntOut
132 AntOut
133 AntIn
133 AntOut
133 AntOut
134 AntIn
134 AntIn
134 AntOut
134 AntOut
135 AntIn
135 AntIn
135 AntIn
135 AntOut
136 AntIn
136 AntIn
136 AntOut
136 AntOut
137 AntIn
137 AntIn
137 AntIn
137 AntOut
137 AntOut
138 AntIn
138 AntIn
138 AntOut
138 AntOut
139 AntIn
139 AntIn
139 AntIn
139 AntOut
139 AntOut
139 AntOut
139 AntOut
140 AntIn
140 AntIn
140 AntIn
140 AntIn
140 AntIn
140 AntIn

141	AntIn
141	AntIn
141	AntIn
141	AntOut
141	AntOut
142	AntIn
142	AntOut
143	AntIn
143	AntIn
143	AntOut
143	AntOut
144	AntIn
144	AntIn
144	AntOut
144	AntOut
144	AntOut
144	AntOut
145	AntOut
145	AntOut
145	AntOut
145	AntOut
145	AntOut
146	AntOut
146	AntOut
146	AntOut
146	AntOut
146	AntOut
146	AntOut
146	AntOut
147	AntIn
147	AntIn
147	AntOut
148	AntIn
148	AntOut
148	AntOut
148	AntOut
149	AntIn
149	AntIn
149	AntOut
150	AntIn
150	AntIn
150	AntOut

150	AntOut
151	AntIn
151	AntIn
151	AntIn
151	AntIn
151	AntIn
151	AntIn
151	AntOut
152	AntIn
152	AntIn
152	AntIn
152	AntIn
152	AntOut
153	AntIn
153	AntIn
153	AntOut
154	AntOut
154	AntOut
154	AntOut
155	AntOut
155	AntOut
155	AntOut
155	AntOut
156	AntOut
156	AntOut
157	AntIn
157	AntOut
157	AntOut
157	AntOut
157	AntOut
158	AntOut
158	AntOut
158	AntOut
158	AntOut
158	AntOut
159	AntIn
159	AntIn
160	AntIn
160	AntIn
160	AntIn
160	AntIn
160	AntIn

160	AntOut
160	AntOut
162	AntIn
162	AntIn
162	AntOut
162	AntOut
162	AntOut
163	AntIn
163	AntIn
163	AntOut
163	AntOut
163	AntOut
164	AntIn
164	AntIn
164	AntIn
164	AntIn
164	AntOut
164	AntOut
164	AntOut
164	AntOut
165	AntIn
165	AntIn
165	AntIn
165	AntIn
165	AntIn
165	AntOut
166	AntIn
166	AntIn
166	AntOut
167	AntIn
167	AntOut
167	AntOut
167	AntOut
167	AntOut
167	AntOut
168	AntIn
168	AntOut
169	AntIn
169	AntIn
169	AntOut
169	AntOut
169	AntOut

169	AntOut
170	AntIn
171	AntIn
171	AntIn
171	AntOut
171	AntOut
171	AntOut
171	AntOut
172	AntIn
172	AntIn
172	AntOut
173	AntOut
173	AntOut
173	AntOut
174	AntIn
174	AntIn
174	AntOut
175	AntIn
175	AntIn
175	AntOut
175	AntOut
176	AntIn
176	AntIn
176	AntOut
177	AntIn
177	AntIn
177	AntOut
177	AntOut
178	AntIn
178	AntIn
178	AntOut
179	AntIn
179	AntIn
180	AntIn
180	AntIn
180	AntOut
180	AntOut
180	AntOut
181	AntIn
181	AntIn
181	AntOut
181	AntOut

181	AntOut
182	AntIn
182	AntIn
182	AntOut
182	AntOut
182	AntOut
182	AntOut
182	AntOut
182	AntOut
183	AntIn
183	AntOut
183	AntOut
184	AntIn
184	AntIn
184	AntOut
184	AntOut
184	AntOut
185	AntIn
185	AntIn
185	AntOut
185	AntOut
185	AntOut
185	AntOut
186	AntIn
186	AntIn
186	AntOut
186	AntOut
187	AntIn
187	AntOut
188	AntIn
188	AntIn
188	AntOut
189	AntIn
189	AntIn
189	AntOut
189	AntOut
189	AntOut
189	AntOut
190	AntIn
190	AntIn
190	AntOut
191	AntIn

191	AntIn
191	AntIn
191	AntIn
191	AntOut
192	AntIn
192	AntIn
192	AntOut
192	AntOut
193	AntIn
193	AntIn
193	AntIn
193	AntIn
193	AntIn
194	AntIn
194	AntIn
194	AntOut
194	AntOut
194	AntOut
195	AntIn
195	AntOut
196	AntIn
196	AntOut
196	AntOut
197	AntIn
197	AntIn
197	AntOut
198	AntIn
198	AntIn
199	AntOut
199	AntOut
200	AntOut
200	AntOut
201	AntIn
201	AntOut
202	AntOut
203	AntIn
203	AntIn
203	AntIn
204	AntIn
204	AntIn
204	AntIn
204	AntIn

204	AntOut
204	AntOut
204	AntOut
205	AntOut
205	AntOut
205	AntOut
206	AntIn
206	AntIn
206	AntIn
206	AntOut
206	AntOut
206	AntOut
207	AntOut
208	AntIn
208	AntOut
208	AntOut
209	AntOut
210	AntIn
210	AntIn
210	AntOut
211	AntOut
211	AntOut
211	AntOut
212	AntIn
212	AntOut
212	AntOut
212	AntOut
212	AntOut
213	AntIn
213	AntIn
213	AntIn
213	AntIn
213	AntIn
214	AntIn
214	AntIn
214	AntIn
215	AntOut
215	AntOut
215	AntOut
215	AntOut
216	AntIn
216	AntOut

216	AntOut
216	AntOut
216	AntOut
217	AntIn
217	AntOut
217	AntOut
218	AntIn
218	AntOut
218	AntOut
219	AntIn
219	AntOut
220	AntIn
220	AntIn
220	AntIn
220	AntOut
221	AntOut
221	AntOut
222	AntIn
222	AntIn
222	AntIn
222	AntOut
223	AntIn
224	AntIn
224	AntIn
224	AntIn
224	AntIn
225	AntIn
225	AntIn
225	AntOut
225	AntOut
225	AntOut
226	AntOut
226	AntOut
226	AntOut
227	AntIn
227	AntIn
227	AntIn
227	AntIn
228	AntIn
228	AntIn
228	AntOut
229	AntIn

229	AntIn
229	AntOut
229	AntOut
230	AntIn
231	AntIn
231	AntIn
231	AntIn
232	AntIn
232	AntIn
232	AntIn
233	AntIn
234	AntIn
234	AntIn
234	AntIn
235	AntIn
235	AntIn
235	AntIn
235	AntOut
236	AntIn
236	AntIn
236	AntOut
236	AntOut
236	AntOut
236	AntOut
236	AntOut
236	AntOut
237	AntOut
237	AntOut
238	AntIn
238	AntOut
238	AntOut
238	AntOut
238	AntOut
239	AntIn
239	AntIn
239	AntOut
239	AntOut
239	AntOut
239	AntOut
239	AntOut
240	AntIn
241	AntIn

242 AntIn
242 AntIn
242 AntOut
242 AntOut
243 AntIn
243 AntOut
243 AntOut
244 AntIn
244 AntIn
244 AntIn
245 AntIn
245 AntIn
245 AntOut
245 AntOut
245 AntOut
245 AntOut
246 AntOut
246 AntOut
247 AntIn
247 AntIn
247 AntIn
247 AntIn
247 AntOut
248 AntIn
248 AntOut
248 AntOut
248 AntOut
248 AntOut
248 AntOut
249 AntIn
249 AntIn
249 AntOut
250 AntIn
250 AntIn
250 AntOut
251 AntIn
251 AntIn
252 AntOut
252 AntOut
252 AntOut
252 AntOut
253 AntIn

253	AntIn
253	AntOut
253	AntOut
254	AntIn
254	AntOut
254	AntOut
255	AntIn
255	AntIn
255	AntIn
255	AntIn
255	AntIn
255	AntIn
256	AntIn
256	AntOut
256	AntOut
256	AntOut
256	AntOut
257	AntIn
257	AntOut
257	AntOut
258	AntIn
258	AntIn
258	AntOut
258	AntOut
258	AntOut
258	AntOut
259	AntIn
259	AntOut
259	AntOut
260	AntIn
260	AntOut
261	AntIn
261	AntIn
261	AntOut
262	AntIn
262	AntOut
262	AntOut
262	AntOut
262	AntOut
263	AntIn
263	AntOut
263	AntOut

263	AntOut
264	AntIn
264	AntOut
265	AntIn
265	AntIn
265	AntIn
265	AntOut
265	AntOut
266	AntIn
266	AntOut
266	AntOut
266	AntOut
268	AntIn
268	AntIn
268	AntIn
268	AntOut
269	AntIn
271	AntOut
271	AntOut
271	AntOut
272	AntIn
272	AntIn
272	AntOut
273	AntIn
273	AntIn
273	AntIn
273	AntOut
274	AntIn
274	AntIn
274	AntOut
274	AntOut
275	AntIn
275	AntOut
275	AntOut
275	AntOut
276	AntIn
276	AntOut
276	AntOut
276	AntOut
276	AntOut
276	AntOut
277	AntIn

277	AntOut
277	AntOut
277	AntOut
277	AntOut
277	AntOut
278	AntIn
278	AntIn
278	AntOut
278	AntOut
278	AntOut
279	AntIn
279	AntIn
279	AntIn
279	AntOut
279	AntOut
279	AntOut
280	AntIn
280	AntIn
280	AntIn
280	AntOut
280	AntOut
281	AntIn
281	AntIn
281	AntIn
281	AntIn
281	AntOut
282	AntIn
282	AntOut
282	AntOut
282	AntOut
282	AntOut
282	AntOut
283	AntOut
283	AntOut
284	AntIn
284	AntIn
284	AntOut
284	AntOut
284	AntOut
285	AntIn
285	AntIn
285	AntOut

285	AntOut
286	AntOut
286	AntOut
286	AntOut
286	AntOut
286	AntOut
287	AntOut
287	AntOut
287	AntOut
287	AntOut
287	AntOut
287	AntOut
288	AntIn
288	AntIn
288	AntIn
288	AntIn
288	AntOut
288	AntOut
288	AntOut
288	AntOut
289	AntIn
289	AntIn
289	AntOut
289	AntOut
290	AntIn
290	AntOut
290	AntOut
290	AntOut
290	AntOut
290	AntOut
291	AntIn
291	AntIn
291	AntIn
291	AntOut
292	AntIn
292	AntIn
292	AntIn
292	AntOut
292	AntOut
292	AntOut
292	AntOut
293	AntIn

293	AntOut
293	AntOut
294	AntIn
294	AntOut
294	AntOut
294	AntOut
294	AntOut
295	AntIn
295	AntIn
295	AntIn
295	AntOut
296	AntIn
296	AntOut
296	AntOut
296	AntOut
296	AntOut
297	AntIn
297	AntIn
297	AntIn
297	AntIn
297	AntOut
298	AntIn
298	AntIn
298	AntIn
598	AntIn
298	AntIn
299	AntIn
299	AntIn
299	AntIn
299	AntIn
299	AntOut
299	AntOut
299	AntOut
300	AntIn
300	AntIn
300	AntIn
301	AntOut
301	AntOut
301	AntOut
302	AntOut
302	AntOut
303	AntIn

303 AntOut
304 AntIn
304 AntOut
304 AntOut
305 AntIn
305 AntIn
305 AntOut
306 AntOut
306 AntOut
306 AntOut
307 AntOut
307 AntOut
308 AntIn
308 AntIn
308 AntIn
308 AntIn
308 AntOut
308 AntOut
309 AntIn
309 AntIn
309 AntOut
309 AntOut
309 AntOut
310 AntIn
310 AntOut
310 AntOut
310 AntOut
310 AntOut
311 AntIn
311 AntOut
311 AntOut
311 AntOut
311 AntOut
312 AntOut
312 AntOut
312 AntOut
313 AntIn
313 AntIn
313 AntOut
313 AntOut
313 AntOut
313 AntOut

313 AntOut
314 AntIn
314 AntOut
314 AntOut
314 AntOut
315 AntIn
315 AntIn
315 AntIn
315 AntOut
316 AntIn
316 AntOut
317 AntIn
317 AntOut
317 AntOut
317 AntOut
317 AntOut
317 AntOut
318 AntIn
318 AntIn
318 AntIn
319 AntIn
319 AntOut
319 AntOut
320 AntIn
320 AntOut
320 AntOut
320 AntOut
321 AntIn
321 AntOut
321 AntOut
322 AntIn
322 AntIn
322 AntOut
323 AntOut
323 AntOut
323 AntOut
323 AntOut
324 AntOut
324 AntOut
325 AntIn
325 AntIn
325 AntIn

325	AntIn
325	AntIn
325	AntOut
325	AntOut
326	AntIn
327	AntIn
327	AntIn
327	AntOut
327	AntOut
327	AntOut
327	AntOut
327	AntOut
327	AntOut
328	AntIn
328	AntIn
328	AntOut
328	AntOut
328	AntOut
329	AntIn
329	AntOut
330	AntOut
330	AntOut
330	AntOut
330	AntOut
331	AntIn
331	AntIn
331	AntOut
331	AntOut
332	AntIn
332	AntIn
332	AntOut
332	AntOut
333	AntOut
333	AntOut
333	AntOut
334	AntIn
334	AntIn
334	AntOut
335	AntOut
336	AntIn
336	AntIn
336	AntIn

336 AntOut
337 AntOut
337 AntOut
337 AntOut
337 AntOut
337 AntOut
338 AntIn
338 AntIn
338 AntOut
338 AntOut
338 AntOut
338 AntOut
339 AntOut
339 AntOut
339 AntOut
339 AntOut
339 AntOut
340 AntOut
340 AntOut
340 AntOut
340 AntOut
340 AntOut
341 AntIn
341 AntIn
341 AntIn
341 AntIn
341 AntOut
342 AntOut
342 AntOut
342 AntOut
343 AntIn
343 AntIn
344 AntIn
344 AntIn
344 AntOut
345 AntIn
345 AntIn
345 AntIn
346 AntIn
346 AntIn
346 AntIn
346 AntIn

346	AntOut
346	AntOut
346	AntOut
347	AntOut
347	AntOut
347	AntOut
347	AntOut
348	AntIn
348	AntOut
349	AntIn
349	AntOut
349	AntOut
349	AntOut
350	AntOut
350	AntOut
351	AntIn
351	AntIn
351	AntOut
351	AntOut
351	AntOut
351	AntOut
352	AntIn
352	AntIn
352	AntOut
352	AntOut
353	AntIn
353	AntIn
354	AntIn
354	AntIn
354	AntIn
354	AntOut
355	AntOut
355	AntOut
356	AntOut
356	AntOut
356	AntOut
356	AntOut
357	AntIn
357	AntOut
357	AntOut
358	AntIn
358	AntIn

359	AntIn
359	AntIn
359	AntIn
359	AntIn
359	AntOut
360	AntIn
360	AntOut
360	AntOut
360	AntOut
360	AntOut
361	AntIn
361	AntIn
361	AntIn
361	AntOut
361	AntOut
362	AntIn
362	AntOut
362	AntOut
363	AntIn
363	AntOut
364	AntIn
364	AntIn
364	AntOut
364	AntOut
364	AntOut
365	AntIn
365	AntIn
365	AntOut
365	AntOut
366	AntIn
366	AntIn
366	AntIn
367	AntIn
367	AntIn
367	AntOut
368	AntIn
368	AntIn
368	AntOut
369	AntIn
369	AntIn
369	AntOut
370	AntIn

370 AntIn
370 AntIn
371 AntOut
371 AntOut
372 AntIn
372 AntIn
372 AntIn
373 AntOut
374 AntIn
374 AntIn
375 AntIn
375 AntIn
375 AntOut
375 AntOut
376 AntIn
376 AntIn
376 AntOut
376 AntOut
377 AntIn
377 AntIn
377 AntOut
378 AntIn
378 AntOut
378 AntOut
379 AntIn
379 AntIn
379 AntOut
379 AntOut
379 AntOut
379 AntOut
380 AntIn
380 AntOut
380 AntOut
380 AntOut
380 AntOut
381 AntIn
381 AntOut
381 AntOut
382 AntIn
382 AntIn
382 AntIn
382 AntOut

383	AntIn
383	AntOut
383	AntOut
383	AntOut
383	AntOut
383	AntOut
384	AntIn
384	AntOut
385	AntOut
385	AntOut
386	AntIn
386	AntIn
386	AntIn
386	AntOut
386	AntOut
387	AntIn
387	AntOut
387	AntOut
388	AntIn
388	AntIn
389	AntIn
389	AntIn
389	AntIn
389	AntIn
389	AntOut
390	AntIn
390	AntIn
390	AntIn
391	AntIn
391	AntIn
391	AntIn
391	AntIn
391	AntIn
392	AntIn
392	AntIn
392	AntIn
392	AntOut
393	AntIn
393	AntOut
393	AntOut
394	AntIn
394	AntIn

394	AntOut
394	AntOut
395	AntIn
395	AntIn
395	AntOut
396	AntIn
396	AntIn
396	AntOut
397	AntIn
397	AntIn
397	AntOut
397	AntOut
397	AntOut
398	AntIn
398	AntIn
398	AntOut
398	AntOut
398	AntOut
398	AntOut
398	AntOut
399	AntIn
399	AntIn
399	AntOut
400	AntOut
400	AntOut
401	AntIn
401	AntIn
401	AntOut
401	AntOut
401	AntOut
402	AntIn
402	AntIn
402	AntOut
402	AntOut
403	AntIn
403	AntIn
403	AntIn
403	AntOut
404	AntIn
404	AntOut
405	AntIn
405	AntOut

405	AntOut
406	AntIn
406	AntOut
406	AntOut
407	AntIn
407	AntOut
408	AntIn
408	AntIn
408	AntIn
408	AntOut
408	AntOut
409	AntIn
409	AntIn
409	AntIn
409	AntOut
409	AntOut
410	AntIn
410	AntOut
410	AntOut
411	AntIn
411	AntIn
411	AntIn
411	AntOut
412	AntIn
412	AntOut
413	AntIn
413	AntIn
413	AntIn
413	AntOut
413	AntOut
413	AntOut
414	AntOut
414	AntOut
414	AntOut
415	AntIn
415	AntIn
416	AntIn
416	AntOut
417	AntIn
417	AntOut
417	AntOut
417	AntOut

418	AntIn
418	AntIn
418	AntIn
418	AntOut
418	AntOut
419	AntIn
419	AntOut
419	AntOut
420	AntIn
420	AntIn
420	AntOut
420	AntOut
420	AntOut
421	AntIn
421	AntIn
421	AntOut
422	AntIn
422	AntIn
422	AntOut
423	AntIn
423	AntOut
423	AntOut
423	AntOut
424	AntOut
425	AntIn
425	AntIn
425	AntIn
425	AntOut
426	AntIn
426	AntIn
426	AntIn
426	AntOut
426	AntOut
427	AntIn
427	AntIn
427	AntOut
427	AntOut
427	AntOut
428	AntIn
428	AntIn
428	AntOut
428	AntOut

429	AntIn
429	AntOut
429	AntOut
430	AntIn
430	AntIn
430	AntIn
430	AntIn
430	AntIn
431	AntIn
431	AntIn
431	AntOut
431	AntOut
431	AntOut
432	AntIn
432	AntIn
432	AntOut
433	AntIn
433	AntOut
434	AntIn
434	AntIn
434	AntIn
434	AntIn
434	AntOut
435	AntIn
435	AntIn
435	AntIn
435	AntIn
435	AntOut
435	AntOut
435	AntOut
436	AntIn
436	AntIn
436	AntOut
436	AntOut
436	AntOut
436	AntOut
437	AntIn
437	AntIn
437	AntOut
437	AntOut
437	AntOut
438	AntIn

438	AntOut
438	AntOut
438	AntOut
438	AntOut
439	AntOut
439	AntOut
439	AntOut
440	AntIn
440	AntOut
440	AntOut
441	AntIn
441	AntIn
441	AntOut
441	AntOut
441	AntOut
442	AntOut
442	AntOut
442	AntOut
443	AntIn
443	AntIn
443	AntIn
443	AntIn
444	AntIn
444	AntOut
445	AntIn
445	AntIn
445	AntIn
445	AntOut
446	AntIn
446	AntOut
446	AntOut
446	AntOut
447	AntIn
448	AntIn
448	AntIn
448	AntOut
448	AntOut
449	AntIn
449	AntOut
449	AntOut
449	AntOut
450	AntOut

450 AntOut
451 AntIn
451 AntIn
451 AntOut
451 AntOut
451 AntOut
451 AntOut
452 AntIn
452 AntIn
452 AntIn
452 AntOut
452 AntOut
452 AntOut
452 AntOut
453 AntIn
453 AntIn
453 AntIn
453 AntOut
453 AntOut
453 AntOut
454 AntIn
454 AntIn
454 AntIn
454 AntOut
455 AntIn
455 AntIn
455 AntOut
456 AntOut
456 AntOut
456 AntOut
456 AntOut
457 AntIn
457 AntOut
457 AntOut
457 AntOut
457 AntOut
457 AntOut
458 AntIn
458 AntIn
458 AntOut
458 AntOut
458 AntOut

458	AntOut
458	AntOut
459	AntIn
459	AntIn
459	AntOut
459	AntOut
459	AntOut
459	AntOut
459	AntOut
460	AntOut
460	AntOut
460	AntOut
461	AntIn
461	AntIn
461	AntOut
461	AntOut
462	AntIn
462	AntIn
462	AntIn
462	AntIn
462	AntOut
462	AntOut
462	AntOut
463	AntIn
463	AntIn
463	AntIn
463	AntOut
463	AntOut
464	AntIn
464	AntIn
464	AntIn
464	AntOut
464	AntOut
465	AntIn
465	AntIn
465	AntIn
465	AntIn
465	AntOut
465	AntOut
466	AntIn
467	AntOut
467	AntOut

467	AntOut
467	AntOut
467	AntOut
468	AntIn
468	AntIn
468	AntIn
468	AntOut
468	AntOut
468	AntOut
469	AntIn
469	AntIn
469	AntIn
469	AntOut
470	AntIn
470	AntOut
470	AntOut
470	AntOut
471	AntOut
472	AntIn
472	AntOut
472	AntOut
472	AntOut
473	AntIn
473	AntOut
473	AntOut
474	AntIn
474	AntIn
474	AntIn
474	AntIn
474	AntOut
475	AntIn
475	AntOut
475	AntOut
475	AntOut
476	AntIn
476	AntIn
476	AntIn
476	AntIn
476	AntOut
477	AntIn
477	AntOut
477	AntOut

477	AntOut
477	AntOut
478	AntIn
478	AntIn
478	AntOut
478	AntOut
478	AntOut
479	AntIn
479	AntOut
480	AntIn
480	AntIn
480	AntOut
480	AntOut
480	AntOut
481	AntIn
481	AntIn
482	AntIn
482	AntIn
482	AntIn
482	AntOut
482	AntOut
483	AntIn
483	AntOut
483	AntOut
483	AntOut
484	AntIn
484	AntIn
485	AntIn
485	AntOut
485	AntOut
485	AntOut
485	AntOut
486	AntIn
486	AntIn
486	AntOut
486	AntOut
487	AntIn
487	AntIn
488	AntIn
488	AntIn
488	AntOut
488	AntOut

488	AntOut
489	AntIn
489	AntIn
490	AntIn
490	AntIn
490	AntIn
490	AntOut
490	AntOut
491	AntIn
492	AntIn
492	AntOut
492	AntOut
492	AntOut
493	AntIn
493	AntIn
493	AntIn
494	AntIn
494	AntIn
494	AntOut
494	AntOut
495	AntOut
496	AntIn
496	AntIn
496	AntIn
496	AntOut
496	AntOut
497	AntIn
497	AntOut
497	AntOut
497	AntOut
498	AntIn
498	AntIn
498	AntOut
499	AntIn
499	AntOut
499	AntOut
499	AntOut
499	AntOut
500	AntIn
500	AntIn
500	AntOut
500	AntOut

500	AntOut
501	AntIn
501	AntIn
501	AntOut
501	AntOut
501	AntOut
502	AntIn
502	AntIn
502	AntOut
502	AntOut
502	AntOut
502	AntOut
502	AntOut
503	AntIn
503	AntIn
503	AntIn
503	AntIn
503	AntOut
504	AntIn
504	AntIn
504	AntIn
505	AntIn
505	AntIn
505	AntOut
506	AntIn
507	AntIn
507	AntIn
507	AntIn
507	AntIn
507	AntIn
507	AntOut
507	AntOut
508	AntIn
508	AntIn
508	AntOut
508	AntOut
508	AntOut
509	AntOut
509	AntOut
509	AntOut
509	AntOut
510	AntIn

510	AntIn
510	AntIn
510	AntOut
510	AntOut
510	AntOut
510	AntOut
511	AntOut
511	AntOut
511	AntOut
511	AntOut
512	AntIn
512	AntOut
512	AntOut
513	AntIn
514	AntIn
514	AntIn
514	AntOut
515	AntOut
515	AntOut
516	AntIn
516	AntIn
516	AntIn
517	AntIn
517	AntIn
517	AntOut
517	AntOut
518	AntIn
518	AntIn
519	AntIn
519	AntIn
519	AntOut
519	AntOut
520	AntIn
520	AntIn
520	AntIn
520	AntIn
521	AntIn
521	AntOut
521	AntOut
521	AntOut
522	AntIn
522	AntIn

522 AntIn
522 AntIn
522 AntIn
522 AntIn
523 AntIn
523 AntIn
523 AntIn
523 AntIn
523 AntOut
524 AntIn
524 AntOut
524 AntOut
525 AntOut
525 AntOut
525 AntOut
525 AntOut
526 AntIn
526 AntIn
526 AntIn
526 AntIn
527 AntIn
527 AntIn
527 AntIn
527 AntIn
527 AntOut
527 AntOut
528 AntIn
528 AntOut
528 AntOut
529 AntIn
529 AntIn
529 AntOut
529 AntOut
530 AntIn
530 AntIn
530 AntOut
531 AntIn
531 AntIn
531 AntIn
531 AntOut
532 AntIn
532 AntIn

532 AntOut
532 AntOut
533 AntIn
533 AntOut
533 AntOut
534 AntIn
534 AntOut
535 AntIn
535 AntOut
535 AntOut
536 AntIn
536 AntOut
536 AntOut
536 AntOut
536 AntOut
536 AntOut
537 AntIn
537 AntIn
537 AntIn
537 AntOut
537 AntOut
538 AntIn
538 AntOut
539 AntIn
539 AntOut
539 AntOut
540 AntIn
541 AntIn
542 AntIn
542 AntIn
542 AntOut
542 AntOut
543 AntIn
543 AntIn
544 AntOut
544 AntOut
545 AntIn
545 AntOut
545 AntOut
546 AntIn
546 AntIn
546 AntIn

546	AntOut
546	AntOut
547	AntIn
547	AntOut
547	AntOut
547	AntOut
548	AntIn
548	AntOut
548	AntOut
549	AntIn
549	AntOut
550	AntOut
550	AntOut
550	AntOut
550	AntOut
550	AntOut
551	AntIn
551	AntIn
551	AntIn
551	AntOut
551	AntOut
552	AntIn
552	AntIn
552	AntIn
552	AntOut
553	AntIn
553	AntIn
553	AntIn
553	AntIn
553	AntOut
553	AntOut
553	AntOut
554	AntIn
554	AntIn
554	AntOut
554	AntOut
555	AntIn
555	AntOut
556	AntIn
556	AntIn
556	AntOut
556	AntOut

556	AntOut
557	AntIn
557	AntIn
557	AntOut
557	AntOut
557	AntOut
558	AntIn
558	AntOut
559	AntIn
559	AntOut
560	AntIn
561	AntIn
561	AntIn
562	AntIn
562	AntIn
562	AntOut
562	AntOut
562	AntOut
563	AntIn
563	AntOut
564	AntIn
564	AntIn
564	AntOut
564	AntOut
564	AntOut
565	AntIn
565	AntIn
565	AntIn
566	AntIn
566	AntIn
566	AntIn
566	AntIn
566	AntIn
567	AntIn
567	AntOut
568	AntIn
568	AntIn
568	AntIn
569	AntIn
569	AntIn
569	AntIn
569	AntOut

570 AntIn
570 AntIn
570 AntIn
570 AntOut
571 AntIn
571 AntIn
571 AntIn
571 AntOut
571 AntOut
572 AntIn
572 AntIn
572 AntIn
572 AntOut
573 AntIn
273 AntOut
574 AntIn
574 AntIn
574 AntIn
574 AntIn
574 AntOut
575 AntIn
575 AntIn
575 AntIn
575 AntOut
576 AntIn
576 AntIn
576 AntIn
576 AntOut
576 AntOut
577 AntIn
577 AntOut
577 AntOut
578 AntOut
578 AntOut
579 AntIn
579 AntOut
579 AntOut
580 AntOut
581 AntIn
581 AntIn
581 AntOut
582 AntIn

582	AntIn
582	AntOut
583	AntIn
583	AntOut
583	AntOut
583	AntOut
584	AntIn
584	AntOut
585	AntIn
585	AntOut
586	AntIn
586	AntIn
586	AntOut
587	AntIn
587	AntIn
587	AntIn
587	AntOut
587	AntOut
588	AntIn
588	AntIn
588	AntIn
588	AntIn
588	AntIn
588	AntOut
589	AntIn
589	AntIn
589	AntOut
590	AntIn
590	AntIn
590	AntOut
590	AntOut
590	AntOut
590	AntOut
592	AntOut
592	AntOut
593	AntIn
593	AntIn
593	AntOut
593	AntOut
593	AntOut
593	AntOut
594	AntIn

595	AntIn
595	AntIn
595	AntOut
595	AntOut
596	AntIn
597	AntIn
597	AntIn
597	AntOut
597	AntOut
597	AntOut
597	AntOut
598	AntIn
598	AntIn
598	AntOut
599	AntIn
599	AntIn
599	AntIn
600	AntOut
600	AntOut
600	AntOut
601	AntIn
601	AntIn
601	AntOut
601	AntOut
602	AntIn
602	AntOut
602	AntOut
603	AntIn
603	AntIn
603	AntOut
603	AntOut
603	AntOut
603	AntOut
604	AntIn
604	AntIn
604	AntIn
604	AntOut
604	AntOut
606	AntIn
606	AntIn
606	AntIn
607	AntIn

607	AntOut
608	AntIn
608	AntIn
608	AntIn
608	AntIn
608	AntOut
608	AntOut
608	AntOut
609	AntIn
609	AntIn
609	AntIn
609	AntOut
609	AntOut
610	AntIn
610	AntIn
611	AntIn
611	AntIn
611	AntIn
612	AntOut
612	AntOut
613	AntIn
613	AntIn
613	AntOut
613	AntOut
614	AntIn
614	AntIn
614	AntIn
614	AntIn
614	AntIn
614	AntIn
614	AntOut
615	AntIn
615	AntOut
615	AntOut
615	AntOut
615	AntOut
616	AntIn
616	AntIn
616	AntIn
617	AntIn
618	AntIn
618	AntIn

618	AntIn
618	AntOut
618	AntOut
619	AntOut
619	AntOut
620	AntIn
620	AntIn
620	AntOut
620	AntOut
621	AntIn
621	AntIn
621	AntOut
621	AntOut
621	AntOut
622	AntIn
622	AntIn
623	AntIn
623	AntIn
623	AntIn
623	AntIn
623	AntOut
623	AntOut
624	AntIn
624	AntIn
625	AntIn
625	AntIn
625	AntOut
625	AntOut
626	AntIn
626	AntIn
626	AntOut
626	AntOut
626	AntOut
626	AntOut
627	AntIn
627	AntIn
627	AntIn
627	AntOut
627	AntOut
627	AntOut
627	AntOut
627	AntOut

628	AntIn
628	AntIn
628	AntIn
628	AntIn
628	AntOut
628	AntOut
629	AntIn
629	AntIn
629	AntIn
629	AntOut
630	AntIn
630	AntOut
631	AntIn
631	AntOut
631	AntOut
631	AntOut
632	AntIn
632	AntIn
632	AntOut
632	AntOut
633	AntOut
634	AntIn
634	AntIn
634	AntIn
634	AntOut
634	AntOut
634	AntOut
635	AntIn
635	AntIn
635	AntIn
635	AntOut
636	AntIn
636	AntIn
636	AntIn
636	AntOut
636	AntOut
637	AntIn
637	AntOut
638	AntIn
638	AntOut
639	AntIn
639	AntIn

639 AntIn
639 AntOut
639 AntOut
640 AntIn
640 AntIn
640 AntIn
640 AntIn
640 AntOut
640 AntOut
641 AntIn
641 AntIn
641 AntIn
642 AntIn
642 AntIn
642 AntIn
642 AntOut
643 AntIn
643 AntIn
643 AntOut
643 AntOut
643 AntOut
644 AntOut
644 AntOut
645 AntIn
645 AntOut
645 AntOut
645 AntOut
646 AntIn
646 AntIn
647 AntIn
647 AntOut
648 AntIn
648 AntIn
648 AntIn
648 AntOut
649 AntIn
649 AntIn
650 AntIn
650 AntIn
650 AntOut
650 AntOut
651 AntIn

651 AntIn
652 AntIn
652 AntIn
652 AntIn
652 AntIn
653 AntOut
653 AntOut
653 AntOut
653 AntOut
654 AntIn
655 AntIn
655 AntIn
655 AntOut
656 AntIn
656 AntIn
656 AntIn
657 AntIn
657 AntIn
658 AntIn
658 AntOut
658 AntOut
658 AntOut
659 AntIn
659 AntIn
659 AntOut
660 AntIn
660 AntIn
661 AntIn
661 AntOut
661 AntOut
661 AntOut
662 AntIn
662 AntIn
662 AntIn
662 AntOut
663 AntIn
663 AntOut
663 AntOut
663 AntOut
665 AntOut
665 AntOut
665 AntOut

666 AntIn
666 AntIn
666 AntOut
666 AntOut
666 AntOut
667 AntOut
668 AntIn
668 AntIn
668 AntIn
668 AntIn
668 AntOut
668 AntOut
669 AntIn
669 AntIn
669 AntIn
669 AntOut
670 AntIn
670 AntOut
670 AntOut
671 AntIn
671 AntOut
672 AntIn
672 AntIn
672 AntOut
673 AntIn
673 AntIn
673 AntOut
673 AntOut
674 AntIn
674 AntIn
674 AntOut
675 AntIn
675 AntIn
675 AntIn
675 AntIn
675 AntOut
676 AntIn
676 AntIn
676 AntOut
677 AntOut
677 AntOut
677 AntOut

677	AntOut
677	AntOut
677	AntOut
678	AntIn
678	AntIn
678	AntOut
678	AntOut
678	AntOut
679	AntIn
679	AntOut
679	AntOut
680	AntOut
684	AntIn
684	AntIn
684	AntOut
682	AntIn
682	AntOut
683	AntIn
683	AntOut
683	AntOut
683	AntOut
683	AntOut
684	AntIn
684	AntOut
684	AntOut
685	AntIn
685	AntOut
685	AntOut
686	AntIn
686	AntIn
687	AntIn
687	AntOut
687	AntOut
688	AntOut
689	AntIn
689	AntIn
689	AntOut
690	AntIn
690	AntIn
691	AntOut
692	AntIn
692	AntIn

692 AntIn
692 AntOut
692 AntOut
693 AntOut
693 AntOut
693 AntOut
693 AntOut
694 AntIn
694 AntIn
694 AntIn
695 AntIn
695 AntOut
696 AntIn
696 AntIn
696 AntIn
696 AntIn
696 AntIn
697 AntIn
697 AntOut
697 AntOut
698 AntOut
698 AntOut
699 AntIn
699 AntIn
699 AntIn
699 AntIn
699 AntOut
699 AntOut
700 AntIn
700 AntIn
700 AntIn
700 AntIn
700 AntIn
700 AntOut
700 AntOut
700 AntOut
700 AntOut
701 AntIn
701 AntIn
701 AntIn
702 AntIn
702 AntIn

702	AntIn
702	AntIn
702	AntOut
702	AntOut
703	AntOut
703	AntOut
703	AntOut
703	AntOut
703	AntOut
704	AntIn
704	AntOut
704	AntOut
704	AntOut
705	AntOut
705	AntOut
705	AntOut
706	AntIn
706	AntOut
706	AntOut
706	AntOut
706	AntOut
706	AntOut
706	AntOut
707	AntIn
707	AntIn
707	AntOut
707	AntOut
708	AntIn
708	AntIn
708	AntIn
708	AntOut
709	AntIn
710	AntOut
710	AntOut
710	AntOut
711	AntIn
711	AntOut
711	AntOut
711	AntOut
711	AntOut
711	AntOut
711	AntOut

712	AntIn
712	AntIn
712	AntOut
712	AntOut
712	AntOut
712	AntOut
713	AntIn
713	AntIn
714	AntIn
714	AntIn
714	AntOut
714	AntOut
714	AntOut
714	AntOut
714	AntOut
715	AntIn
715	AntOut
715	AntOut
716	AntIn
716	AntIn
716	AntOut
717	AntOut
717	AntOut
717	AntOut
717	AntOut
717	AntOut
717	AntOut
717	AntOut
717	AntOut
718	AntIn
718	AntIn
718	AntIn
718	AntIn
718	AntOut
718	AntOut
718	AntOut
719	AntIn
719	AntIn
719	AntOut
719	AntOut
719	AntOut
719	AntOut

720 AntIn
720 AntIn
720 AntIn
720 AntOut
720 AntOut
720 AntOut
720 AntOut
721 AntIn
721 AntIn
722 AntIn
722 AntIn
722 AntOut
722 AntOut
722 AntOut
722 AntOut
723 AntIn
723 AntOut
723 AntOut
723 AntOut
724 AntIn
724 AntIn
724 AntIn
724 AntIn
724 AntOut
725 AntIn
725 AntIn
725 AntIn
725 AntOut
725 AntOut
726 AntIn
726 AntIn
726 AntOut
727 AntIn
727 AntIn
728 AntIn
728 AntIn
728 AntIn
728 AntOut
728 AntOut
728 AntOut
728 AntOut
729 AntIn

729	AntOut
729	AntOut
730	AntIn
730	AntOut
730	AntOut
730	AntOut
730	AntOut
731	AntIn
731	AntIn
731	AntIn
731	AntOut
731	AntOut
732	AntIn
732	AntIn
733	AntIn
733	AntIn
734	AntIn
734	AntIn
734	AntIn
734	AntIn
734	AntIn
734	AntIn
734	AntOut
734	AntOut
735	AntIn
735	AntIn
735	AntIn
735	AntIn
736	AntIn
736	AntIn
736	AntOut
736	AntOut
736	AntOut
737	AntIn
737	AntOut
737	AntOut
737	AntOut
737	AntOut
738	AntIn
738	AntOut
738	AntOut
738	AntOut

738 AntOut
738 AntOut
739 AntIn
739 AntIn
739 AntIn
739 AntOut
740 AntIn
740 AntIn
740 AntIn
741 AntIn
741 AntOut
741 AntOut
742 AntIn
742 AntIn
742 AntOut
742 AntOut
743 AntIn
743 AntIn
743 AntIn
743 AntIn
743 AntIn
743 AntOut
744 AntIn
744 AntIn
744 AntIn
744 AntOut
744 AntOut
745 AntIn
546 AntIn
746 AntOut
747 AntOut
747 AntOut
747 AntOut
748 AntIn
748 AntIn
748 AntIn
748 AntOut
749 AntOut
749 AntOut
750 AntOut
750 AntOut
751 AntIn

751	AntIn
751	AntIn
751	AntOut
751	AntOut
752	AntIn
752	AntOut
753	AntIn
753	AntOut
753	AntOut
754	AntOut
755	AntIn
755	AntIn
755	AntIn
755	AntOut
755	AntOut
756	AntIn
756	AntOut
757	AntIn
757	AntIn
757	AntIn
757	AntOut
758	AntIn
758	AntOut
758	AntOut
759	AntIn
759	AntOut
760	AntIn
760	AntIn
760	AntIn
761	AntIn
761	AntIn
762	AntIn
762	AntIn
762	AntIn
762	AntOut
763	AntOut
764	AntIn
764	AntOut
765	AntIn
767	AntIn
767	AntIn
767	AntIn

767 AntOut
767 AntOut
768 AntOut
768 AntOut
768 AntOut
769 AntIn
769 AntOut
770 AntIn
770 AntIn
770 AntOut
771 AntOut
772 AntIn
772 AntOut
772 AntOut
773 AntOut
773 AntOut
774 AntIn
774 AntIn
774 AntIn
774 AntOut
775 AntIn
775 AntOut
775 AntOut
776 AntIn
777 AntIn
777 AntOut
777 AntOut
777 AntOut
777 AntOut
778 AntIn
778 AntIn
778 AntIn
778 AntOut
778 AntOut
779 AntIn
779 AntIn
779 AntIn
779 AntIn
779 AntOut
779 AntOut
780 AntIn
780 AntIn

780 AntIn
780 AntOut
780 AntOut
781 AntIn
781 AntOut
782 AntIn
782 AntIn
782 AntOut
782 AntOut
782 AntOut
783 AntIn
783 AntIn
783 AntIn
784 AntIn
784 AntIn
784 AntOut
784 AntOut
784 AntOut
784 AntOut
784 AntOut
784 AntOut
785 AntIn
785 AntIn
785 AntIn
785 AntIn
785 AntOut
786 AntIn
786 AntIn
786 AntIn
786 AntIn
786 AntIn
787 AntIn
787 AntIn
787 AntOut
787 AntOut
787 AntOut
788 AntIn
788 AntIn
788 AntIn
789 AntIn
789 AntOut
789 AntOut

789	AntOut
790	AntIn
790	AntIn
790	AntOut
791	AntOut
792	AntIn
792	AntIn
792	AntOut
792	AntOut
793	AntOut
793	AntOut
793	AntOut
794	AntIn
794	AntIn
794	AntIn
794	AntOut
795	AntIn
795	AntIn
795	AntOut
795	AntOut
795	AntOut
796	AntOut
796	AntOut
796	AntOut
796	AntOut
796	AntOut
796	AntOut
796	AntOut
797	AntIn
797	AntOut
798	AntOut
799	AntIn
799	AntIn
800	AntIn
800	AntIn
800	AntIn
801	AntIn
801	AntIn
801	AntOut
801	AntOut
801	AntOut
802	AntOut

802 AntOut
803 AntOut
803 AntOut
803 AntOut
804 AntIn
804 AntIn
804 AntOut
805 AntIn
806 AntIn
806 AntIn
806 AntIn
806 AntOut
806 AntOut
807 AntIn
807 AntIn
807 AntOut
807 AntOut
807 AntOut
807 AntOut
808 AntOut
808 AntOut
808 AntOut
808 AntOut
808 AntOut
808 AntOut
808 AntOut
809 AntIn
809 AntIn
809 AntIn
809 AntIn
809 AntIn
809 AntOut
809 AntOut
811 AntIn
811 AntOut
811 AntOut
812 AntIn
812 AntIn
812 AntIn
812 AntOut
812 AntOut
813 AntOut

813	AntOut
814	AntIn
814	AntIn
814	AntOut
814	AntOut
814	AntOut
814	AntOut
814	AntOut
815	AntIn
815	AntOut
815	AntOut
815	AntOut
815	AntOut
815	AntOut
815	AntOut
816	AntIn
817	AntIn
817	AntIn
817	AntIn
817	AntOut
818	AntIn
818	AntIn
818	AntIn
818	AntOut
818	AntOut
819	AntIn
819	AntIn
819	AntOut
819	AntOut
820	AntIn
820	AntIn
820	AntOut
820	AntOut
820	AntOut
821	AntIn
821	AntIn
821	AntOut
821	AntOut
822	AntIn
822	AntOut
822	AntOut
823	AntIn

823	AntOut
823	AntOut
824	AntIn
824	AntIn
824	AntIn
824	AntIn
824	AntIn
824	AntOut
825	AntIn
825	AntIn
825	AntIn
825	AntOut
826	AntIn
826	AntIn
826	AntIn
826	AntOut
827	AntIn
827	AntOut
828	AntOut
829	AntIn
829	AntIn
829	AntOut
829	AntOut
829	AntOut
829	AntOut
829	AntOut
830	AntIn
830	AntIn
830	AntOut
830	AntOut
830	AntOut
831	AntOut
831	AntOut
832	AntIn
832	AntOut
832	AntOut
833	AntOut
833	AntOut
833	AntOut
833	AntOut
834	AntOut
834	AntOut

834 AntOut
835 AntIn
835 AntIn
835 AntIn
835 AntOut
836 AntIn
836 AntIn
836 AntOut
836 AntOut
836 AntOut
837 AntIn
837 AntIn
838 AntIn
838 AntIn
838 AntOut
838 AntOut
838 AntOut
839 AntIn
839 AntOut
840 AntIn
840 AntIn
840 AntIn
840 AntOut
841 AntIn
841 AntIn
841 AntIn
841 AntOut
841 AntOut
841 AntOut
841 AntOut
842 AntIn
842 AntIn
842 AntIn
842 AntOut
843 AntIn
843 AntIn
843 AntOut
843 AntOut
843 AntOut
844 AntOut
845 AntIn
845 AntIn

845	AntOut
845	AntOut
846	AntOut
846	AntOut
846	AntOut
846	AntOut
846	AntOut
846	AntOut
847	AntIn
847	AntOut
848	AntIn
848	AntOut
849	AntIn
849	AntIn
850	AntIn
850	AntIn
850	AntIn
851	AntIn
851	AntIn
851	AntIn
852	AntIn
852	AntIn
852	AntIn
852	AntIn
852	AntIn
852	AntIn
852	AntIn
852	AntOut
852	AntOut
853	AntOut
853	AntOut
854	AntIn
854	AntIn
855	AntOut
856	AntIn
856	AntIn
856	AntOut
857	AntOut
857	AntOut
858	AntIn
858	AntIn
858	AntOut

858 AntOut
858 AntOut
859 AntIn
859 AntOut
859 AntOut
859 AntOut
859 AntOut
860 AntIn
860 AntOut
860 AntOut
861 AntIn
861 AntIn
861 AntOut
862 AntOut
863 AntIn
863 AntOut
863 AntOut
864 AntIn
864 AntOut
864 AntOut
864 AntOut
865 AntIn
865 AntIn
865 AntIn
865 AntOut
865 AntOut
865 AntOut
866 AntIn
866 AntIn
866 AntIn
866 AntOut
867 AntIn
867 AntIn
867 AntOut
868 AntOut
868 AntOut
868 AntOut
869 AntIn
870 AntIn
870 AntOut
870 AntOut
870 AntOut

870 AntOut
871 AntIn
871 AntIn
871 AntIn
871 AntOut
871 AntOut
871 AntOut
871 AntOut
871 AntOut
872 AntIn
872 AntIn
872 AntIn
872 AntIn
873 AntIn
873 AntIn
873 AntOut
874 AntIn
874 AntIn
874 AntOut
874 AntOut
875 AntOut
876 AntIn
876 AntIn
876 AntIn
876 AntIn
876 AntOut
877 AntIn
877 AntIn
878 AntIn
878 AntIn
878 AntIn
878 AntOut
879 AntOut
880 AntIn
880 AntIn
880 AntIn
880 AntOut
880 AntOut
880 AntOut
881 AntIn
881 AntIn
881 AntOut

881 AntOut
881 AntOut
881 AntOut
881 AntOut
882 AntIn
882 AntIn
883 AntIn
883 AntIn
883 AntIn
883 AntOut
884 AntIn
884 AntIn
884 AntIn
884 AntOut
884 AntOut
884 AntOut
885 AntIn
885 AntIn
885 AntOut
885 AntOut
885 AntOut
885 AntOut
885 AntOut
886 AntIn
886 AntIn
886 AntOut
886 AntOut
887 AntIn
887 AntOut
888 AntIn
888 AntIn
888 AntIn
888 AntOut
888 AntOut
888 AntOut
889 AntIn
889 AntOut
889 AntOut
889 AntOut
889 AntOut
890 AntIn
890 AntIn

891 AntIn
891 AntOut
891 AntOut
892 AntIn
892 AntIn
892 AntIn
892 AntOut
892 AntOut
893 AntIn
893 AntIn
893 AntIn
893 AntIn
893 AntOut
894 AntIn
894 AntOut
895 AntIn
895 AntIn
895 AntIn
896 AntIn
896 AntOut
897 AntOut
897 AntOut
897 AntOut
898 AntIn
898 AntIn
898 AntOut
899 AntIn
899 AntIn
900 AntIn
900 AntIn
900 AntOut
901 AntIn
901 AntOut
901 AntOut
902 AntOut
902 AntOut
903 AntOut
903 AntOut
903 AntOut
903 AntOut
903 AntOut
904 AntIn

904 AntOut
904 AntOut
905 AntIn
905 AntOut
905 AntOut
906 AntIn
906 AntOut
906 AntOut
907 AntIn
907 AntIn
907 AntIn
907 AntIn
907 AntOut
908 AntIn
909 AntIn
910 AntIn
910 AntOut
910 AntOut
910 AntOut
911 AntIn
911 AntOut
912 AntIn
912 AntOut
913 AntIn
913 AntIn
913 AntIn
913 AntOut
914 AntIn
914 AntOut
916 AntIn
916 AntIn
917 AntOut
918 AntOut
919 AntOut
922 AntIn
922 AntOut
923 AntOut
924 AntIn
924 AntOut
925 AntOut
927 AntOut
928 AntOut

929	AntOut
931	AntIn
934	AntIn
938	AntOut
939	AntIn
939	AntIn
941	AntIn
942	AntOut
943	AntOut
945	AntIn
945	AntOut
947	AntIn
948	AntOut
950	AntIn
950	AntIn
952	AntOut
953	AntIn
953	AntOut
956	AntIn
3	Descend
3	Descend
4	Descend
4	Descend
4	Descend
6	Descend
6	Ascend
6	Ascend
7	Ascend
8	Descend
9	Descend
9	Descend
10	Ascend
11	Ascend
11	Ascend
12	Descend
13	Descend
13	Descend
14	Ascend
14	Ascend
15	Ascend
15	Ascend
16	Descend

17	Descend
17	Descend
19	Descend
19	Descend
19	Descend
19	Descend
20	Descend
20	Descend
20	Descend
21	Descend
21	Ascend
21	Ascend
21	Ascend
22	Ascend
22	Ascend
23	Ascend
24	Descend
24	Ascend
25	Ascend
26	Descend
26	Descend
27	Descend
27	Descend
28	Descend
28	Ascend
29	Ascend
30	Ascend
30	Descend
30	Descend
32	Descend
33	Descend
33	Descend
34	Descend
34	Descend
35	Descend
35	Descend
36	Ascend
36	Ascend
37	Ascend
37	Descend
37	Descend
38	Descend

38	Descend
38	Descend
39	Descend
39	Ascend
39	Ascend
40	Ascend
40	Ascend
41	Ascend
41	Descend
41	Descend
42	Descend
42	Ascend
43	Ascend
43	Descend
43	Descend
44	Ascend
44	Ascend
45	Descend
46	Descend
46	Descend
47	Descend
48	Descend
48	Descend
49	Descend
50	Descend
51	Ascend
51	Ascend
51	Ascend
52	Descend
52	Descend
52	Ascend
53	Ascend
53	Ascend
54	Descend
54	Descend
54	Descend
55	Descend
55	Descend
56	Descend
56	Descend
56	Descend
57	Descend

57	Descend
57	Descend
58	Ascend
58	Ascend
58	Ascend
59	Ascend
59	Ascend
59	Ascend
60	Descend
60	Descend
61	Ascend
62	Ascend
62	Descend
62	Descend
63	Descend
63	Descend
63	Descend
64	Descend
65	Ascend
65	Ascend
65	Ascend
66	Descend
66	Descend
67	Descend
68	Descend
68	Descend
68	Descend
69	Descend
69	Descend
70	Descend
70	Descend
71	Descend
71	Descend
72	Ascend
72	Ascend
72	Ascend
72	Ascend
73	Ascend
73	Ascend
74	Ascend
75	Ascend
75	Ascend

76	Ascend
76	Descend
77	Descend
77	Descend
78	Ascend
78	Ascend
79	Ascend
79	Descend
79	Descend
80	Descend
80	Descend
81	Ascend
81	Ascend
81	Ascend
82	Ascend
82	Ascend
82	Ascend
83	Descend
83	Descend
83	Descend
84	Descend
84	Descend
84	Descend
85	Descend
85	Descend
85	Descend
86	Descend
86	Descend
86	Descend
87	Descend
87	Descend
88	Descend
88	Descend
88	Descend
89	Descend
89	Descend
89	Ascend
90	Ascend
90	Ascend
91	Ascend
91	Ascend
92	Ascend

92	Ascend
92	Descend
93	Descend
93	Descend
93	Descend
93	Descend
94	Descend
94	Descend
94	Descend
94	Descend
95	Descend
95	Descend
95	Descend
96	Descend
96	Ascend
96	Ascend
97	Ascend
97	Ascend
97	Ascend
97	Ascend
98	Ascend
98	Ascend
98	Ascend
98	Ascend
99	Ascend
99	Descend
99	Descend
100	Descend
100	Descend
101	Ascend
101	Ascend
101	Ascend
102	Descend
102	Descend
102	Descend
103	Ascend
103	Ascend
103	Ascend
104	Descend
104	Descend
105	Descend
105	Descend

105	Descend
106	Descend
106	Descend
106	Descend
107	Descend
107	Descend
108	Descend
108	Descend
108	Descend
108	Ascend
109	Ascend
109	Ascend
109	Ascend
110	Ascend
110	Ascend
110	Ascend
111	Ascend
111	Descend
111	Descend
112	Descend
112	Descend
112	Descend
112	Descend
112	Descend
113	Descend
113	Descend
113	Descend
113	Descend
113	Descend
114	Descend
114	Descend
114	Descend
114	Descend
115	Descend
115	Descend
115	Descend
115	Descend
116	Descend
116	Descend
116	Descend
117	Descend
117	Descend

117 Descend
117 Ascend
117 Ascend
118 Ascend
118 Ascend
118 Ascend
119 Ascend
120 Descend
120 Descend
121 Descend
121 Descend
121 Descend
121 Descend
121 Descend
122 Descend
123 Descend
124 Ascend
124 Ascend
125 Ascend
126 Ascend
126 Ascend
126 Ascend
126 Descend
126 Descend
127 Descend
127 Descend
127 Descend
127 Descend
127 Descend
127 Descend
128 Ascend
128 Ascend
128 Ascend
129 Ascend
129 Descend
129 Descend
129 Descend
129 Ascend
130 Ascend
130 Ascend
130 Ascend
131 Ascend

131	Ascend
131	Ascend
132	Descend
132	Descend
132	Descend
132	Descend
133	Descend
133	Ascend
133	Ascend
133	Ascend
133	Descend
134	Descend
134	Descend
134	Descend
134	Descend
135	Descend
135	Descend
135	Descend
135	Descend
136	Descend
136	Descend
136	Descend
136	Descend
136	Descend
137	Ascend
137	Ascend
137	Ascend
138	Ascend
138	Ascend
138	Ascend
138	Ascend
139	Descend
139	Descend
139	Descend
139	Descend
140	Descend
140	Descend
141	Descend
141	Descend
142	Descend
142	Ascend
142	Ascend

143	Ascend
143	Ascend
143	Ascend
143	Ascend
144	Ascend
144	Ascend
144	Ascend
145	Ascend
145	Ascend
145	Ascend
145	Ascend
146	Ascend
146	Ascend
146	Ascend
146	Ascend
147	Ascend
147	Ascend
147	Descend
147	Descend
148	Descend
148	Descend
148	Descend
149	Descend
149	Descend
149	Descend
150	Descend
150	Descend
150	Descend
151	Ascend
151	Ascend
151	Ascend
151	Ascend
152	Ascend
152	Ascend
152	Ascend
153	Ascend
153	Ascend
154	Ascend
154	Ascend
155	Descend
155	Descend
155	Descend

155	Descend
155	Descend
156	Descend
156	Descend
156	Descend
157	Ascend
157	Ascend
157	Ascend
158	Ascend
158	Descend
159	Descend
160	Descend
160	Descend
160	Descend
161	Descend
161	Descend
163	Descend
163	Descend
163	Ascend
164	Ascend
165	Ascend
165	Ascend
166	Ascend
166	Descend
167	Ascend
167	Ascend
169	Ascend
169	Descend
170	Descend
170	Descend
170	Descend
170	Descend
170	Descend
171	Descend
171	Descend
171	Descend
171	Descend
172	Descend
172	Descend
172	Descend
172	Descend
173	Descend

173	Descend
174	Descend
174	Descend
174	Descend
175	Descend
176	Descend
177	Descend
178	Descend
178	Descend
179	Descend
179	Ascend
180	Ascend
180	Ascend
181	Ascend
181	Descend
181	Descend
182	Descend
182	Descend
182	Descend
183	Descend
183	Descend
184	Descend
184	Descend
185	Descend
186	Descend
187	Descend
188	Ascend
188	Ascend
190	Ascend
191	Ascend
191	Ascend
192	Ascend
193	Descend
193	Descend
194	Descend
194	Descend
195	Descend
195	Descend
196	Ascend
196	Ascend
197	Ascend
197	Descend

198	Descend
199	Ascend
199	Descend
199	Descend
200	Descend
201	Descend
201	Descend
202	Descend
202	Descend
202	Ascend
203	Ascend
203	Ascend
204	Ascend
204	Ascend
205	Ascend
206	Descend
206	Descend
207	Descend
207	Descend
207	Descend
208	Descend
208	Descend
208	Descend
208	Descend
209	Descend
209	Descend
209	Descend
210	Descend
210	Descend
210	Descend
210	Descend
211	Descend
211	Descend
212	Descend
212	Descend
212	Descend
213	Descend
213	Ascend
214	Ascend
214	Ascend
215	Ascend
215	Ascend

215	Ascend
216	Ascend
216	Ascend
217	Ascend
217	Ascend
218	Ascend
218	Ascend
219	Ascend
219	Ascend
220	Descend
220	Descend
220	Descend
220	Descend
221	Descend
221	Descend
222	Descend
222	Descend
223	Descend
223	Descend
224	Descend
224	Descend
224	Ascend
225	Ascend
225	Ascend
225	Ascend
226	Descend
226	Descend
226	Descend
226	Descend
227	Descend
227	Descend
228	Descend
228	Descend
229	Descend
229	Descend
229	Descend
230	Descend
230	Ascend
231	Ascend
231	Ascend
231	Ascend
232	Ascend

232	Ascend
233	Ascend
233	Ascend
233	Ascend
234	Ascend
234	Ascend
235	Ascend
235	Ascend
235	Ascend
235	Ascend
236	Ascend
236	Ascend
236	Ascend
236	Ascend
237	Ascend
237	Ascend
238	Ascend
238	Ascend
238	Ascend
239	Ascend
239	Ascend
239	Descend
240	Descend
240	Descend
240	Descend
240	Descend
241	Descend
241	Descend
241	Descend
242	Descend
242	Descend
242	Descend
243	Descend
243	Descend
244	Descend
244	Descend
244	Ascend
244	Ascend
245	Ascend
245	Ascend
245	Ascend
246	Ascend

246	Ascend
247	Ascend
248	Ascend
249	Ascend
249	Descend
250	Descend
250	Descend
250	Descend
250	Descend
250	Descend
251	Descend
251	Descend
252	Ascend
252	Ascend
253	Ascend
253	Ascend
253	Ascend
253	Ascend
254	Ascend
254	Ascend
254	Ascend
255	Ascend
255	Ascend
255	Ascend
256	Ascend
256	Ascend
256	Ascend
257	Ascend
257	Ascend
258	Ascend
259	Descend
259	Descend
260	Descend
260	Descend
260	Descend
261	Descend
261	Descend
261	Descend
262	Descend
262	Descend
263	Descend
263	Descend

263	Descend
264	Descend
264	Descend
265	Descend
265	Descend
265	Descend
266	Descend
266	Descend
266	Descend
267	Descend
267	Descend
267	Descend
268	Descend
269	Descend
269	Descend
270	Descend
270	Descend
270	Descend
270	Descend
271	Descend
271	Descend
271	Descend
272	Descend
272	Descend
273	Descend
273	Descend
274	Descend
274	Ascend
275	Descend
275	Ascend
275	Ascend
275	Ascend
276	Ascend
276	Descend
276	Descend
276	Descend
277	Descend
278	Descend
278	Descend
279	Descend
280	Descend
280	Descend

281	Descend
281	Descend
281	Ascend
282	Ascend
282	Ascend
283	Ascend
283	Descend
284	Descend
284	Descend
284	Descend
285	Descend
285	Descend
286	Descend
286	Descend
287	Descend
287	Descend
288	Descend
288	Ascend
288	Ascend
288	Ascend
289	Ascend
289	Ascend
290	Ascend
290	Ascend
290	Descend
291	Descend
291	Descend
292	Descend
292	Descend
292	Descend
293	Descend
293	Ascend
293	Ascend
294	Ascend
294	Ascend
294	Ascend
295	Ascend
295	Ascend
296	Ascend
296	Ascend
297	Descend
297	Descend

297	Descend
297	Descend
297	Descend
298	Descend
298	Descend
298	Descend
299	Descend
299	Descend
300	Descend
301	Ascend
301	Ascend
302	Ascend
303	Ascend
303	Ascend
303	Ascend
303	Descend
304	Descend
304	Descend
305	Descend
305	Descend
305	Descend
306	Descend
307	Descend
307	Ascend
307	Ascend
308	Descend
308	Descend
308	Descend
309	Descend
309	Descend
310	Descend
310	Descend
311	Descend
311	Descend
312	Ascend
312	Ascend
312	Ascend
313	Ascend
313	Descend
313	Descend
314	Descend
314	Descend

314	Descend
315	Descend
315	Descend
316	Descend
316	Descend
317	Descend
317	Descend
318	Descend
318	Ascend
318	Ascend
319	Ascend
319	Ascend
319	Ascend
320	Ascend
320	Ascend
320	Ascend
321	Descend
321	Descend
322	Descend
322	Descend
322	Descend
323	Descend
323	Descend
324	Descend
324	Descend
325	Descend
326	Descend
327	Descend
327	Descend
328	Ascend
328	Ascend
329	Ascend
329	Ascend
330	Ascend
331	Ascend
331	Descend
331	Descend
332	Descend
332	Descend
332	Descend
333	Descend
333	Descend

333	Descend
334	Descend
334	Descend
335	Ascend
335	Ascend
335	Ascend
336	Ascend
336	Descend
337	Descend
337	Descend
337	Descend
337	Descend
338	Descend
339	Descend
339	Ascend
339	Ascend
340	Ascend
341	Ascend
342	Descend
342	Descend
343	Descend
344	Descend
346	Descend
347	Descend
347	Descend
347	Descend
348	Descend
348	Ascend
349	Ascend
349	Ascend
349	Ascend
350	Ascend
350	Ascend
351	Ascend
352	Ascend
353	Ascend
354	Descend
355	Descend
355	Descend
355	Descend
356	Descend
357	Descend

358	Descend
359	Descend
361	Descend
362	Ascend
362	Ascend
363	Ascend
363	Ascend
363	Descend
364	Descend
364	Descend
364	Descend
364	Descend
365	Descend
365	Descend
365	Descend
366	Descend
366	Descend
367	Descend
368	Descend
369	Descend
370	Descend
370	Descend
371	Descend
371	Ascend
372	Ascend
372	Ascend
372	Ascend
373	Ascend
373	Ascend
374	Ascend
374	Ascend
376	Ascend
376	Ascend
376	Ascend
377	Ascend
378	Descend
378	Descend
378	Descend
378	Descend
378	Descend
379	Descend
380	Descend

380	Descend
381	Descend
381	Descend
381	Descend
381	Descend
382	Descend
382	Descend
382	Descend
383	Ascend
383	Ascend
383	Ascend
384	Ascend
384	Ascend
384	Ascend
385	Ascend
385	Descend
385	Descend
385	Descend
386	Descend
386	Descend
387	Descend
388	Descend
389	Descend
389	Descend
390	Descend
391	Ascend
392	Ascend
392	Ascend
393	Ascend
394	Descend
394	Ascend
395	Ascend
396	Ascend
396	Ascend
396	Ascend
396	Ascend
397	Ascend
397	Ascend
398	Descend
398	Descend
399	Descend
399	Descend

399	Descend
400	Descend
400	Descend
401	Descend
401	Descend
401	Descend
401	Descend
402	Descend
402	Descend
402	Descend
404	Descend
404	Descend
405	Ascend
405	Ascend
406	Ascend
406	Ascend
407	Ascend
408	Ascend
408	Ascend
409	Ascend
409	Ascend
409	Ascend
410	Ascend
410	Descend
410	Descend
410	Descend
410	Descend
411	Descend
411	Descend
412	Descend
412	Descend
413	Descend
413	Descend
414	Ascend
414	Ascend
414	Ascend
415	Ascend
415	Descend
416	Descend
416	Descend
417	Descend
418	Descend

418	Descend
418	Ascend
419	Ascend
420	Descend
420	Descend
421	Ascend
421	Ascend
422	Descend
422	Descend
422	Descend
424	Descend
424	Descend
426	Descend
427	Descend
427	Ascend
427	Ascend
427	Ascend
428	Ascend
428	Ascend
428	Ascend
429	Descend
429	Descend
429	Descend
429	Descend
430	Descend
430	Descend
430	Descend
430	Descend
431	Descend
431	Descend
431	Descend
431	Descend
432	Descend
432	Descend
432	Descend
432	Descend
433	Ascend
433	Ascend
434	Ascend
434	Ascend
435	Descend
435	Descend

435	Descend
435	Descend
436	Descend
436	Descend
436	Descend
437	Descend
438	Descend
439	Ascend
440	Ascend
440	Ascend
441	Ascend
441	Ascend
443	Ascend
444	Ascend
444	Ascend
444	Ascend
445	Ascend
445	Descend
445	Descend
446	Descend
446	Descend
446	Descend
446	Descend
447	Descend
448	Descend
448	Descend
448	Descend
449	Descend
449	Descend
450	Descend
451	Descend
452	Descend
452	Descend
453	Descend
454	Descend
457	Ascend
459	Descend
460	Descend
460	Descend
460	Descend
461	Descend
461	Descend

461 Ascend
462 Ascend
463 Ascend
463 Descend
463 Descend
464 Descend
464 Descend
465 Descend
466 Descend
466 Descend
467 Descend
468 Ascend
468 Ascend
469 Ascend
469 Ascend
469 Ascend
470 Ascend
470 Ascend
471 Ascend
471 Ascend
472 Ascend
473 Ascend
473 Ascend
474 Ascend
475 Ascend
476 Descend
476 Descend
476 Descend
477 Descend
478 Descend
478 Descend
479 Descend
479 Ascend
479 Ascend
480 Ascend
481 Descend
481 Ascend
481 Ascend
482 Ascend
483 Ascend
484 Ascend
484 Ascend

485	Descend
485	Descend
487	Descend
487	Descend
488	Ascend
488	Descend
488	Descend
489	Descend
489	Descend
490	Descend
490	Descend
491	Descend
491	Ascend
492	Ascend
493	Ascend
494	Ascend
494	Ascend
495	Descend
495	Descend
496	Descend
497	Descend
498	Descend
499	Descend
499	Ascend
500	Ascend
501	Ascend
501	Ascend
502	Ascend
503	Ascend
504	Ascend
505	Ascend
506	Ascend
508	Descend
509	Descend
509	Descend
510	Descend
511	Descend
512	Descend
512	Descend
514	Ascend
514	Ascend
515	Ascend

516	Descend
516	Descend
517	Descend
518	Descend
519	Ascend
519	Ascend
520	Ascend
520	Ascend
521	Descend
523	Descend
523	Descend
524	Descend
525	Ascend
525	Ascend
526	Ascend
526	Ascend
526	Ascend
526	Ascend
527	Ascend
527	Ascend
527	Ascend
527	Ascend
528	Ascend
528	Ascend
528	Ascend
529	Ascend
529	Ascend
529	Ascend
530	Ascend
530	Ascend
530	Ascend
530	Ascend
530	Descend
531	Descend
531	Descend
531	Descend
531	Descend
532	Descend
532	Descend
533	Descend
533	Descend
533	Descend

533 Descend
534 Descend
534 Descend
534 Ascend
535 Ascend
535 Ascend
535 Descend
535 Descend
536 Descend
536 Descend
536 Descend
537 Descend
538 Descend
539 Ascend
539 Ascend
540 Descend
542 Ascend
543 Ascend
543 Ascend
544 Ascend
545 Ascend
547 Ascend
547 Ascend
547 Ascend
548 Ascend
548 Ascend
549 Ascend
549 Ascend
550 Ascend
551 Ascend
552 Descend
553 Descend
553 Descend
553 Descend
553 Descend
554 Descend
554 Descend
554 Descend
555 Descend
555 Descend
556 Descend
556 Descend

557	Descend
558	Descend
558	Descend
558	Descend
560	Descend
561	Descend
562	Descend
563	Descend
563	Descend
563	Descend
563	Descend
564	Ascend
564	Ascend
564	Ascend
565	Ascend
565	Ascend
566	Ascend
567	Ascend
567	Ascend
567	Ascend
568	Ascend
568	Ascend
569	Ascend
569	Ascend
570	Ascend
571	Descend
571	Ascend
572	Ascend
573	Ascend
574	Ascend
574	Ascend
575	Ascend
575	Ascend
577	Ascend
578	Ascend
578	Ascend
579	Ascend
581	Ascend
581	Ascend
582	Descend
582	Descend
582	Descend

583	Descend
584	Descend
585	Descend
586	Descend
586	Descend
586	Descend
588	Descend
589	Ascend
589	Ascend
589	Ascend
590	Ascend
590	Ascend
591	Ascend
591	Ascend
593	Ascend
594	Ascend
598	Ascend
598	Ascend
599	Ascend
600	Ascend
601	Descend
601	Descend
602	Ascend
604	Ascend
606	Ascend
607	Ascend
608	Ascend
609	Ascend
611	Descend
612	Descend
612	Descend
613	Descend
613	Descend
614	Descend
616	Ascend
616	Ascend
617	Descend
619	Ascend
619	Ascend
620	Descend
621	Descend
623	Descend

624	Descend
628	Descend
629	Descend
629	Ascend
631	Descend
633	Descend
633	Ascend
633	Ascend
634	Ascend
636	Ascend
637	Descend
637	Descend
638	Descend
639	Descend
639	Descend
640	Descend
640	Ascend
641	Ascend
642	Ascend
643	Ascend
644	Descend
644	Descend
644	Descend
644	Descend
645	Descend
645	Descend
646	Ascend
646	Ascend
647	Ascend
648	Ascend
649	Ascend
651	Ascend
652	Ascend
653	Descend
653	Descend
655	Descend
655	Ascend
656	Ascend
657	Ascend
657	Ascend
659	Ascend
659	Descend

660	Descend
661	Descend
663	Descend
663	Ascend
663	Ascend
664	Descend
664	Descend
665	Descend
666	Ascend
666	Ascend
667	Ascend
668	Ascend
668	Descend
669	Descend
671	Ascend
672	Descend
673	Descend
673	Descend
674	Ascend
674	Ascend
676	Descend
676	Descend
677	Descend
678	Ascend
679	Ascend
680	Ascend
681	Descend
682	Ascend
682	Ascend
683	Ascend
684	Descend
684	Descend
685	Descend
686	Descend
687	Descend
688	Descend
688	Descend
689	Descend
690	Descend
690	Descend
691	Descend
692	Descend

693	Ascend
693	Ascend
694	Ascend
694	Ascend
695	Ascend
695	Ascend
695	Ascend
695	Ascend
695	Ascend
696	Ascend
696	Ascend
696	Ascend
697	Ascend
697	Ascend
698	Ascend
698	Descend
698	Descend
699	Descend
699	Descend
699	Descend
700	Descend
700	Descend
701	Descend
701	Descend
701	Descend
703	Ascend
703	Ascend
703	Ascend
703	Ascend
704	Ascend
704	Descend
704	Descend
704	Descend
704	Descend
705	Descend
705	Descend
705	Descend
705	Descend
706	Ascend
706	Ascend
706	Ascend
706	Ascend

707	Ascend
707	Ascend
707	Descend
707	Descend
707	Descend
708	Descend
708	Descend
708	Descend
709	Descend
709	Descend
709	Descend
711	Ascend
711	Ascend
712	Descend
712	Descend
713	Descend
713	Descend
713	Descend
714	Descend
714	Ascend
714	Ascend
715	Ascend
715	Ascend
715	Descend
715	Descend
716	Descend
716	Descend
716	Descend
716	Descend
717	Descend
717	Descend
718	Descend
718	Descend
718	Descend
718	Descend
719	Descend
719	Descend
720	Descend
720	Descend
721	Descend
722	Descend
724	Descend

725	Ascend
725	Ascend
725	Ascend
725	Ascend
726	Ascend
726	Descend
726	Descend
726	Descend
727	Descend
727	Descend
728	Descend
729	Descend
729	Descend
730	Descend
731	Descend
732	Descend
733	Descend
733	Descend
733	Descend
734	Descend
734	Ascend
734	Ascend
735	Ascend
735	Descend
735	Descend
736	Descend
737	Descend
739	Descend
739	Descend
740	Descend
740	Descend
740	Descend
740	Descend
741	Descend
741	Descend
742	Descend
742	Descend
743	Descend
744	Descend
746	Descend
746	Descend
747	Descend

747	Descend
747	Descend
748	Descend
748	Ascend
749	Ascend
749	Ascend
750	Descend
751	Descend
751	Ascend
752	Ascend
753	Descend
753	Descend
753	Descend
754	Descend
754	Descend
754	Descend
755	Descend
755	Descend
756	Descend
756	Descend
757	Descend
757	Descend
758	Descend
758	Descend
759	Ascend
759	Ascend
759	Ascend
760	Ascend
761	Ascend
761	Ascend
762	Ascend
762	Ascend
763	Ascend
763	Descend
764	Descend
764	Descend
764	Descend
765	Descend
765	Descend
766	Descend
766	Descend
767	Descend

767	Descend
768	Descend
770	Descend
772	Descend
773	Ascend
774	Ascend
774	Ascend
774	Ascend
775	Ascend
775	Ascend
776	Ascend
777	Ascend
778	Descend
778	Descend
778	Descend
779	Descend
779	Descend
779	Descend
780	Descend
780	Descend
780	Descend
781	Ascend
781	Ascend
781	Ascend
782	Ascend
782	Ascend
783	Ascend
783	Ascend
784	Ascend
785	Ascend
785	Ascend
786	Ascend
786	Ascend
787	Ascend
787	Ascend
788	Ascend
789	Ascend
790	Ascend
791	Ascend
791	Descend
791	Descend
792	Descend

792	Descend
792	Descend
793	Descend
793	Descend
794	Descend
795	Descend
795	Descend
795	Descend
795	Descend
796	Descend
796	Descend
797	Descend
797	Descend
798	Descend
798	Ascend
799	Ascend
799	Ascend
799	Ascend
799	Ascend
800	Ascend
800	Ascend
801	Descend
802	Descend
802	Descend
803	Descend
804	Descend
805	Descend
807	Descend
809	Ascend
809	Ascend
810	Ascend
811	Ascend
811	Ascend
812	Ascend
813	Ascend
814	Descend
816	Descend
817	Ascend
820	Descend
820	Descend
821	Descend
821	Descend

821	Ascend
822	Ascend
823	Ascend
823	Ascend
823	Descend
824	Descend
824	Descend
825	Descend
827	Descend
827	Descend
827	Descend
829	Descend
830	Descend
831	Descend
832	Descend
833	Ascend
834	Ascend
835	Ascend
835	Descend
835	Descend
836	Descend
836	Descend
837	Descend
837	Descend
837	Descend
838	Descend
839	Descend
839	Descend
839	Descend
840	Descend
841	Descend
841	Descend
842	Descend
842	Descend
843	Descend
845	Descend
846	Descend
846	Ascend
847	Ascend
847	Ascend
848	Ascend
848	Ascend

848	Ascend
848	Ascend
849	Ascend
849	Ascend
850	Ascend
850	Ascend
850	Ascend
851	Ascend
851	Descend
851	Descend
852	Descend
853	Descend
853	Descend
854	Descend
854	Descend
855	Descend
856	Ascend
856	Ascend
857	Ascend
858	Ascend
859	Descend
860	Descend
861	Ascend
861	Ascend
861	Ascend
861	Ascend
862	Ascend
862	Ascend
862	Ascend
863	Ascend
863	Ascend
864	Descend
864	Descend
864	Descend
864	Descend
865	Descend
865	Descend
865	Descend
866	Descend
866	Ascend
866	Ascend
867	Ascend

867 Ascend
867 Ascend
868 Descend
868 Descend
869 Descend
869 Descend
870 Ascend
870 Ascend
870 Ascend
871 Ascend
871 Ascend
872 Descend
872 Descend
872 Descend
873 Descend
873 Descend
873 Descend
874 Descend
875 Descend
875 Ascend
876 Ascend
876 Ascend
876 Ascend
877 Ascend
879 Descend
879 Descend
880 Descend
880 Ascend
881 Ascend
881 Ascend
882 Ascend
882 Ascend
882 Ascend
883 Descend
883 Descend
884 Ascend
885 Ascend
886 Descend
887 Ascend
887 Ascend
887 Descend
887 Descend

888	Descend
888	Descend
889	Ascend
890	Ascend
890	Ascend
891	Ascend
892	Ascend
892	Ascend
893	Descend
893	Descend
894	Descend
895	Ascend
895	Descend
896	Descend
897	Descend
898	Ascend
899	Ascend
901	Ascend
901	Ascend
901	Ascend
902	Ascend
903	Descend
904	Descend
904	Descend
905	Descend
