# Supplementary material for: Interactions Increase Forager Availability and Activity in Harvester Ants
Source: PLoS One. 2015 Nov 5;10(11):e0141971. doi: 10.1371/journal.pone.0141971 (PMC4635008; doi:10.1371/journal.pone.0141971)
Supplement: S3 Dataset — We observed and filmed behavior inside the nest during and after forager removals. This dataset shows our counts made from the films of the numbers of returning and outgoing foragers at the nest entrance and the number of ascending and descending ants at all tunnel entrances. (ZIP) [file pone.0141971.s004.zip › S3 Dataset/2013 Correlation Data N5 8-18.pdf]

**Researcher**	**Jovel Queirolo**
**Colony N5**	
**8/18/13**	
**Video time (seconds)**	**Event**
3	Descend
4	Ascend
5	Descend
7	Descend
9	Descend
9	Ascend
11	Ascend
13	Ascend
14	Ascend
15	Ascend
17	Ascend
17	Descend
19	Ascend
19	Ascend
20	Ascend
21	Ascend
22	Descend
23	Descend
23	Descend
24	Descend
25	Ascend
25	Ascend
26	Ascend
27	Ascend
27	Ascend
28	Ascend
29	Ascend
30	Descend
30	Descend
31	Descend
32	Ascend
33	Descend
34	Descend
35	Descend
38	Descend
38	Descend
39	Ascend

39	Ascend
40	Ascend
41	Ascend
42	Ascend
43	Ascend
43	Ascend
44	Descend
45	Descend
46	Descend
47	Descend
47	Descend
48	Descend
49	Descend
50	Descend
50	Ascend
51	Descend
52	Ascend
52	Ascend
53	Descend
53	Descend
53	Ascend
54	Ascend
55	Ascend
55	Descend
56	Descend
56	Descend
57	Descend
58	Descend
58	Ascend
59	Ascend
59	Ascend
60	Ascend
61	Ascend
61	Ascend
62	Ascend
62	Descend
63	Descend
63	Ascend
64	Ascend
64	Descend
65	Ascend
66	Descend

67	Descend
67	Descend
68	Descend
69	Ascend
69	Ascend
70	Ascend
71	Ascend
72	Ascend
72	Ascend
73	Ascend
73	Ascend
74	Descend
75	Descend
76	Descend
76	Descend
77	Descend
77	Descend
78	Ascend
79	Ascend
79	Ascend
80	Ascend
80	Descend
81	Descend
82	Descend
82	Ascend
83	Ascend
83	Ascend
84	Ascend
84	Ascend
85	Ascend
85	Ascend
86	Ascend
86	Ascend
87	Descend
87	Descend
88	Descend
89	Descend
89	Descend
90	Descend
91	Descend
92	Ascend
92	Ascend

93	Ascend
94	Ascend
96	Ascend
97	Ascend
98	Ascend
99	Descend
102	Descend
105	Ascend
107	Ascend
109	Ascend
110	Descend
112	Descend
112	Descend
113	Ascend
113	Ascend
114	Ascend
115	Ascend
116	Ascend
116	Ascend
117	Ascend
118	Descend
118	Descend
119	Descend
119	Descend
120	Descend
121	Descend
122	Descend
123	Ascend
123	Ascend
123	Ascend
124	Ascend
125	Ascend
125	Ascend
126	Ascend
126	Descend
127	Descend
127	Ascend
127	Ascend
128	Ascend
129	Ascend
129	Ascend
130	Ascend

130	Ascend
130	Ascend
130	Ascend
131	Ascend
132	Ascend
132	Ascend
133	Ascend
133	Ascend
134	Ascend
135	Ascend
136	Ascend
137	Descend
138	Descend
138	Ascend
139	Descend
139	Descend
140	Ascend
141	Ascend
142	Ascend
144	Ascend
144	Ascend
144	Ascend
144	Ascend
145	Ascend
145	Ascend
146	Descend
146	Descend
147	Descend
147	Descend
148	Descend
149	Descend
150	Descend
150	Ascend
151	Ascend
152	Ascend
153	Ascend
154	Ascend
159	Descend
159	Descend
161	Descend
162	Ascend
162	Ascend

162	Ascend
162	Ascend
163	Ascend
163	Ascend
164	Descend
164	Descend
164	Descend
166	Descend
166	Descend
166	Ascend
167	Ascend
167	Ascend
168	Ascend
168	Ascend
169	Ascend
170	Descend
170	Descend
171	Descend
172	Descend
174	Descend
174	Descend
175	Descend
175	Descend
176	Descend
177	Ascend
177	Ascend
178	Ascend
178	Ascend
178	Ascend
179	Ascend
180	Ascend
181	Ascend
181	Ascend
182	Ascend
182	Ascend
184	Ascend
185	Ascend
186	Ascend
186	Ascend
186	Ascend
187	Ascend
188	Ascend

188	Ascend
189	Ascend
189	Ascend
190	Ascend
190	Ascend
192	Descend
192	Descend
192	Descend
193	Ascend
193	Ascend
193	Ascend
193	Ascend
194	Descend
194	Descend
195	Descend
195	Descend
195	Descend
196	Descend
196	Descend
196	Descend
196	Descend
197	Descend
197	Ascend
197	Ascend
198	Ascend
198	Ascend
198	Ascend
199	Ascend
199	Ascend
199	Ascend
200	Ascend
200	Ascend
201	Ascend
201	Descend
201	Descend
201	Descend
202	Descend
202	Descend
202	Descend
203	Descend
205	Ascend
205	Ascend

205	Ascend
205	Ascend
206	Ascend
206	Ascend
206	Ascend
207	Descend
207	Descend
207	Descend
208	Descend
209	Descend
209	Ascend
210	Ascend
210	Ascend
211	Descend
211	Descend
212	Descend
214	Descend
214	Descend
215	Descend
216	Descend
217	Ascend
219	Ascend
219	Ascend
219	Ascend
221	Ascend
222	Descend
223	Descend
223	Descend
224	Descend
224	Descend
225	Ascend
225	Ascend
225	Ascend
225	Ascend
226	Ascend
226	Ascend
227	Ascend
228	Ascend
229	Ascend
230	Ascend
231	Descend
231	Descend

231	Descend
231	Descend
232	Descend
234	Ascend
234	Ascend
235	Descend
236	Descend
236	Descend
237	Descend
237	Ascend
238	Ascend
238	Ascend
239	Descend
239	Descend
240	Descend
241	Descend
241	Ascend
241	Ascend
242	Ascend
242	Ascend
242	Descend
243	Descend
243	Descend
244	Descend
245	Descend
246	Descend
247	Ascend
248	Ascend
248	Descend
250	Ascend
250	Ascend
251	Ascend
252	Ascend
253	Ascend
253	Ascend
254	Ascend
255	Descend
255	Descend
256	Descend
256	Descend
257	Descend
258	Descend

260	Descend
260	Ascend
261	Ascend
261	Ascend
261	Ascend
262	Ascend
262	Ascend
263	Ascend
263	Ascend
264	Ascend
264	Ascend
265	Ascend
265	Ascend
265	Descend
265	Descend
266	Descend
266	Descend
266	Descend
266	Descend
266	Descend
267	Descend
267	Descend
267	Descend
268	Descend
268	Descend
270	Descend
271	Ascend
271	Ascend
271	Ascend
272	Ascend
272	Ascend
273	Ascend
274	Ascend
276	Ascend
276	Ascend
276	Descend
279	Ascend
279	Ascend
280	Ascend
281	Descend
281	Descend
284	Descend

284	Descend
284	Descend
285	Descend
285	Descend
286	Descend
286	Descend
288	Descend
288	Descend
289	Descend
289	Ascend
289	Ascend
289	Ascend
290	Ascend
290	Ascend
291	Ascend
292	Ascend
292	Ascend
293	Ascend
293	Ascend
293	Ascend
294	Descend
294	Descend
295	Descend
295	Descend
297	Descend
297	Ascend
297	Ascend
297	Ascend
298	Ascend
300	Ascend
301	Ascend
301	Descend
301	Descend
303	Descend
303	Descend
305	Ascend
305	Ascend
305	Ascend
306	Ascend
308	Descend
308	Descend
309	Ascend

310	Ascend
310	Ascend
310	Ascend
310	Ascend
312	Ascend
312	Ascend
313	Ascend
313	Ascend
313	Ascend
313	Ascend
314	Ascend
314	Descend
315	Descend
317	Descend
317	Descend
318	Descend
319	Descend
323	Ascend
324	Ascend
324	Ascend
325	Ascend
327	Ascend
327	Ascend
327	Ascend
328	Ascend
329	Descend
329	Descend
329	Descend
332	Descend
332	Ascend
333	Ascend
335	Descend
335	Descend
337	Descend
338	Descend
340	Ascend
340	Ascend
341	Descend
341	Descend
342	Descend
343	Ascend
343	Ascend

344	Ascend
345	Ascend
345	Ascend
345	Ascend
346	Ascend
346	Ascend
347	Ascend
348	Ascend
349	Ascend
349	Ascend
349	Ascend
349	Ascend
350	Ascend
350	Ascend
352	Ascend
352	Ascend
354	Ascend
354	Ascend
354	Ascend
354	Ascend
356	Ascend
357	Ascend
358	Ascend
358	Descend
358	Descend
359	Ascend
359	Ascend
360	Descend
360	Ascend
360	Ascend
361	Ascend
361	Ascend
362	Ascend
362	Ascend
363	Ascend
363	Ascend
364	Ascend
364	Ascend
365	Ascend
365	Ascend
366	Ascend
366	Ascend

367	Ascend
368	Descend
368	Descend
368	Descend
369	Descend
369	Descend
370	Descend
370	Descend
371	Descend
371	Descend
371	Descend
372	Descend
372	Descend
373	Descend
373	Descend
373	Descend
374	Descend
374	Descend
376	Descend
376	Descend
376	Descend
377	Ascend
377	Ascend
377	Ascend
377	Ascend
378	Ascend
378	Ascend
378	Ascend
378	Ascend
379	Ascend
379	Ascend
379	Ascend
379	Ascend
380	Ascend
380	Ascend
380	Ascend
381	Descend
381	Descend
382	Descend
382	Descend
382	Descend
382	Descend

383	Descend
383	Descend
384	Descend
384	Descend
385	Descend
385	Descend
385	Descend
386	Descend
386	Ascend
386	Ascend
386	Ascend
387	Ascend
388	Ascend
388	Ascend
388	Descend
388	Descend
389	Descend
389	Descend
389	Descend
389	Descend
390	Descend
390	Descend
390	Descend
391	Descend
391	Descend
391	Descend
392	Descend
393	Descend
393	Ascend
393	Ascend
394	Ascend
394	Ascend
394	Ascend
395	Ascend
396	Ascend
396	Ascend
397	Ascend
397	Ascend
398	Ascend
398	Ascend
399	Ascend
399	Descend

399	Descend
399	Descend
400	Descend
400	Descend
401	Descend
401	Descend
402	Descend
402	Descend
402	Descend
402	Descend
403	Descend
403	Descend
403	Descend
403	Descend
403	Ascend
404	Ascend
404	Ascend
404	Ascend
404	Ascend
404	Ascend
405	Ascend
405	Ascend
405	Ascend
405	Ascend
406	Ascend
407	Descend
407	Descend
408	Descend
408	Descend
409	Descend
409	Descend
409	Descend
410	Descend
410	Descend
410	Descend
411	Descend
411	Descend
411	Descend
412	Descend
412	Descend
413	Descend
414	Descend

415	Descend
415	Descend
416	Descend
417	Ascend
417	Ascend
417	Ascend
417	Ascend
418	Ascend
418	Ascend
418	Ascend
418	Ascend
419	Ascend
419	Ascend
419	Ascend
419	Ascend
420	Ascend
421	Descend
421	Descend
422	Descend
422	Descend
422	Descend
422	Descend
422	Descend
423	Descend
423	Descend
424	Descend
424	Descend
426	Descend
426	Descend
427	Ascend
427	Ascend
428	Ascend
428	Ascend
428	Ascend
430	Ascend
430	Ascend
431	Ascend
431	Ascend
431	Ascend
432	Ascend
432	Ascend
433	Ascend

433	Ascend
435	Ascend
435	Ascend
436	Ascend
436	Ascend
437	Descend
438	Descend
439	Descend
440	Descend
440	Descend
441	Descend
442	Ascend
442	Ascend
443	Ascend
443	Ascend
444	Ascend
445	Ascend
445	Ascend
447	Ascend
448	Ascend
449	Ascend
449	Ascend
450	Descend
451	Descend
451	Descend
452	Descend
454	Descend
454	Descend
455	Ascend
455	Ascend
456	Descend
460	Ascend
460	Ascend
461	Ascend
462	Ascend
462	Ascend
463	Descend
465	Ascend
465	Ascend
465	Ascend
466	Ascend
466	Ascend

467	Ascend
467	Ascend
468	Ascend
468	Descend
469	Descend
469	Ascend
470	Ascend
479	Ascend
480	Ascend
481	Ascend
482	Ascend
483	Ascend
483	Ascend
484	Ascend
484	Ascend
485	Ascend
485	Ascend
486	Ascend
487	Ascend
487	Ascend
488	Ascend
488	Ascend
489	Ascend
489	Ascend
489	Ascend
490	Ascend
490	Ascend
490	Ascend
490	Ascend
491	Ascend
491	Ascend
492	Ascend
492	Ascend
494	Ascend
494	Ascend
496	Descend
496	Descend
496	Descend
497	Descend
497	Descend
497	Descend
498	Descend

498	Descend
499	Descend
499	Descend
500	Descend
500	Descend
501	Descend
502	Descend
502	Descend
503	Descend
504	Descend
505	Ascend
505	Ascend
505	Ascend
506	Ascend
506	Ascend
506	Ascend
506	Ascend
507	Ascend
507	Ascend
507	Ascend
507	Ascend
508	Ascend
508	Ascend
510	Ascend
510	Ascend
512	Ascend
512	Ascend
513	Ascend
513	Ascend
513	Descend
514	Descend
514	Descend
514	Descend
515	Descend
516	Descend
517	Descend
517	Descend
518	Descend
518	Descend
518	Descend
519	Descend
519	Ascend

519	Ascend
520	Ascend
520	Ascend
520	Ascend
520	Ascend
521	Ascend
521	Ascend
522	Descend
522	Descend
522	Descend
523	Descend
523	Descend
524	Descend
524	Descend
524	Descend
525	Ascend
525	Ascend
526	Ascend
526	Ascend
527	Ascend
527	Ascend
528	Ascend
529	Ascend
529	Ascend
530	Descend
530	Descend
530	Descend
531	Descend
531	Descend
531	Descend
532	Descend
532	Descend
532	Descend
533	Descend
533	Descend
533	Descend
534	Descend
534	Descend
534	Descend
535	Descend
535	Descend
535	Descend

536	Ascend
536	Ascend
536	Ascend
537	Ascend
537	Descend
537	Descend
537	Descend
538	Ascend
538	Ascend
539	Ascend
539	Ascend
539	Ascend
540	Ascend
540	Ascend
540	Ascend
542	Ascend
542	Ascend
542	Ascend
542	Ascend
542	Ascend
542	Ascend
543	Ascend
543	Ascend
543	Ascend
544	Ascend
544	Ascend
544	Ascend
545	Descend
545	Descend
545	Descend
546	Descend
546	Ascend
546	Ascend
546	Ascend
547	Ascend
547	Ascend
548	Descend
548	Descend
549	Descend
549	Descend
549	Descend
549	Ascend

549 Ascend
550 Ascend
551 Ascend
551 Ascend
551 Ascend
551 Ascend
552 Ascend
552 Ascend
553 Ascend
553 Ascend
553 Ascend
554 Descend
554 Descend
554 Descend
555 Descend
555 Descend
555 Descend
556 Descend
556 Descend
558 Descend
558 Descend
559 Descend
559 Ascend
560 Ascend
560 Ascend
562 Ascend
562 Ascend
564 Ascend
564 Ascend
566 Descend
566 Descend
567 Descend
567 Descend
568 Ascend
568 Ascend
569 Ascend
572 Ascend
575 Descend
575 Descend
576 Ascend
576 Ascend
577 Ascend

578	Ascend
578	Ascend
578	Ascend
579	Ascend
579	Ascend
581	Ascend
581	Ascend
582	Descend
583	Descend
583	Descend
583	Descend
584	Descend
584	Descend
584	Descend
585	Descend
585	Descend
587	Descend
587	Descend
588	Descend
588	Descend
589	Ascend
589	Ascend
590	Ascend
591	Ascend
591	Ascend
591	Ascend
592	Ascend
592	Ascend
593	Ascend
594	Descend
594	Descend
594	Descend
595	Descend
595	Descend
596	Descend
596	Ascend
596	Ascend
597	Ascend
597	Ascend
598	Ascend
598	Ascend
599	Ascend

600 Ascend
600 Ascend
603 Descend
603 Descend
605 Ascend
606 Ascend
607 Ascend
607 Ascend
609 Ascend
609 Ascend
610 Ascend
611 Ascend
611 Ascend
611 Ascend
612 Descend
612 Descend
612 Descend
612 Descend
613 Descend
614 Descend
614 Descend
615 Ascend
615 Ascend
616 Ascend
616 Ascend
617 Descend
617 Descend
618 Descend
618 Descend
619 Descend
619 Descend
621 Descend
621 Descend
622 Descend
622 Ascend
622 Ascend
623 Ascend
623 Ascend
623 Ascend
624 Descend
624 Ascend
624 Ascend

624	Ascend
625	Descend
626	Descend
627	Descend
628	Ascend
628	Ascend
629	Descend
629	Descend
631	Descend
631	Descend
631	Ascend
632	Ascend
633	Ascend
634	Ascend
634	Descend
635	Descend
636	Ascend
637	Ascend
638	Ascend
638	Ascend
639	Ascend
640	Ascend
641	Ascend
641	Ascend
642	Ascend
643	Ascend
644	Ascend
646	Ascend
647	Ascend
648	Descend
648	Descend
648	Descend
649	Descend
649	Ascend
649	Ascend
650	Ascend
650	Ascend
650	Ascend
651	Ascend
651	Ascend
652	Ascend
653	Ascend

653 Ascend
654 Descend
654 Descend
655 Descend
655 Descend
655 Descend
656 Descend
656 Descend
656 Descend
657 Descend
661 Descend
661 Descend
667 Descend
667 Descend
667 Descend
667 Descend
668 Descend
668 Descend
669 Ascend
669 Ascend
670 Ascend
670 Ascend
671 Ascend
671 Ascend
671 Ascend
671 Ascend
674 Descend
674 Descend
675 Descend
675 Descend
681 Ascend
682 Ascend
687 Descend
688 Descend
689 Descend
689 Descend
691 Descend
692 Ascend
693 Ascend
694 Ascend
695 Ascend
695 Descend

696	Descend
696	Descend
697	Descend
698	Ascend
698	Ascend
700	Ascend
701	Descend
701	Descend
702	Descend
702	Descend
704	Ascend
704	Descend
707	Ascend
707	Ascend
708	Descend
709	Descend
710	Ascend
712	Descend
713	Ascend
714	Descend
716	Descend
717	Descend
717	Ascend
718	Ascend
719	Descend
722	Descend
723	Ascend
725	Ascend
725	Ascend
726	Ascend
726	Descend
727	Descend
730	Descend
731	Descend
731	Descend
732	Ascend
732	Ascend
733	Ascend
734	Descend
735	Ascend
737	Descend
737	Descend

739 Ascend
740 Ascend
740 Ascend
741 Ascend
743 Ascend
744 Ascend
744 Ascend
745 Ascend
746 Ascend
746 Descend
747 Descend
747 Descend
747 Descend
748 Descend
748 Descend
749 Descend
749 Descend
749 Descend
750 Descend
750 Descend
750 Descend
751 Descend
751 Descend
752 Descend
752 Ascend
752 Ascend
752 Ascend
752 Ascend
753 Ascend
753 Ascend
753 Ascend
753 Ascend
754 Ascend
754 Ascend
754 Ascend
755 Ascend
755 Ascend
755 Descend
755 Descend
756 Descend
756 Ascend
756 Ascend

757	Ascend
757	Ascend
758	Ascend
759	Ascend
759	Ascend
760	Ascend
760	Ascend
761	Ascend
762	Ascend
763	Ascend
765	Ascend
765	Descend
766	Descend
766	Descend
766	Descend
767	Descend
767	Descend
768	Ascend
770	Ascend
770	Ascend
770	Ascend
772	Ascend
772	Ascend
773	Ascend
774	Ascend
775	Ascend
776	Ascend
776	Ascend
778	Ascend
779	Ascend
780	Ascend
781	Ascend
784	Ascend
784	Descend
786	Ascend
786	Ascend
787	Ascend
789	Descend
790	Ascend
791	Descend
792	Descend
792	Descend

792	Descend
793	Descend
793	Descend
794	Descend
794	Descend
796	Descend
796	Ascend
796	Ascend
797	Ascend
797	Ascend
798	Ascend
798	Ascend
801	Ascend
801	Descend
801	Descend
802	Descend
802	Descend
802	Descend
803	Descend
803	Ascend
803	Ascend
804	Ascend
804	Ascend
805	Ascend
805	Descend
805	Descend
806	Descend
806	Descend
809	Ascend
810	Ascend
810	Ascend
810	Ascend
811	Ascend
812	Ascend
812	Ascend
813	Ascend
813	Ascend
813	Ascend
816	Ascend
818	Ascend
820	Ascend
822	Ascend

824	Ascend
827	Ascend
827	Ascend
829	Ascend
829	Ascend
831	Ascend
833	Descend
833	Descend
833	Descend
833	Descend
833	Descend
835	Descend
835	Descend
837	Ascend
837	Ascend
838	Ascend
838	Ascend
840	Descend
840	Descend
842	Ascend
842	Ascend
844	Ascend
844	Ascend
844	Ascend
844	Descend
845	Descend
846	Descend
846	Descend
847	Descend
848	Descend
848	Descend
849	Descend
850	Descend
851	Ascend
852	Ascend
852	Ascend
852	Ascend
853	Descend
853	Descend
854	Ascend
854	Ascend
857	Ascend

858	Descend
858	Descend
858	Descend
859	Descend
859	Descend
860	Ascend
860	Ascend
861	Ascend
861	Ascend
862	Descend
862	Descend
863	Ascend
864	Descend
864	Descend
865	Descend
865	Ascend
865	Ascend
865	Ascend
866	Ascend
866	Ascend
866	Ascend
866	Ascend
867	Ascend
867	Ascend
867	Ascend
868	Ascend
869	Descend
869	Descend
870	Descend
870	Ascend
870	Ascend
871	Descend
872	Descend
873	Descend
873	Descend
873	Descend
873	Descend
874	Descend
874	Descend
875	Ascend
875	Ascend
876	Ascend

876	Ascend
876	Ascend
877	Ascend
877	Ascend
878	Ascend
878	Descend
878	Descend
879	Descend
879	Descend
880	Ascend
880	Ascend
881	Ascend
882	Ascend
882	Ascend
883	Ascend
883	Ascend
884	Ascend
884	Ascend
884	Ascend
885	Ascend
885	Descend
885	Descend
886	Descend
887	Ascend
888	Ascend
888	Ascend
888	Ascend
891	Ascend
891	Ascend
892	Ascend
894	Descend
895	Descend
895	Descend
895	Descend
896	Descend
896	Descend
897	Descend
897	Ascend
897	Ascend
898	Ascend
898	Ascend
898	Ascend

898	Ascend
899	Ascend
899	Ascend
899	Ascend
899	Ascend
900	Descend
901	Descend
901	Descend
903	Ascend
903	Ascend
907	Descend
907	Descend
908	Ascend
908	Ascend
908	Ascend
909	Ascend
909	Ascend
7	AntOut
8	AntIn
8	AntOut
9	AntIn
9	AntIn
9	AntIn
10	AntIn
10	AntIn
10	AntIn
10	AntOut
11	AntIn
11	AntIn
11	AntIn
11	AntOut
11	AntOut
12	AntIn
12	AntOut
12	AntOut
13	AntOut
14	AntIn
14	AntOut
15	AntIn
15	AntOut
16	AntIn
17	AntIn

17	AntIn
18	AntIn
18	AntIn
19	AntIn
19	AntIn
19	AntIn
19	AntOut
19	AntOut
20	AntIn
21	AntIn
21	AntIn
21	AntIn
21	AntIn
22	AntIn
22	AntIn
22	AntIn
22	AntOut
23	AntIn
23	AntOut
23	AntOut
24	AntIn
24	AntIn
24	AntIn
24	AntIn
25	AntIn
25	AntOut
25	AntOut
26	AntIn
26	AntIn
27	AntIn
27	AntIn
27	AntIn
27	AntIn
28	AntIn
28	AntIn
28	AntOut
28	AntOut
29	AntIn
29	AntIn
29	AntOut
29	AntOut
30	AntIn

30 AntIn
30 AntOut
31 AntIn
31 AntIn
31 AntIn
31 AntOut
31 AntOut
32 AntIn
32 AntIn
32 AntOut
33 AntIn
33 AntIn
33 AntOut
33 AntOut
34 AntIn
34 AntOut
34 AntOut
34 AntOut
35 AntIn
36 AntIn
36 AntIn
36 AntIn
36 AntIn
36 AntOut
37 AntIn
38 AntOut
38 AntOut
39 AntOut
39 AntOut
39 AntOut
40 AntIn
40 AntOut
41 AntIn
42 AntIn
42 AntIn
42 AntIn
42 AntIn
42 AntOut
43 AntIn
43 AntIn
43 AntIn
43 AntIn

43 AntIn
44 AntIn
44 AntIn
44 AntIn
45 AntOut
45 AntOut
45 AntOut
46 AntIn
46 AntOut
47 AntIn
47 AntOut
48 AntIn
48 AntOut
49 AntIn
49 AntIn
49 AntOut
50 AntIn
50 AntIn
50 AntOut
51 AntIn
51 AntIn
53 AntIn
53 AntOut
53 AntOut
53 AntOut
54 AntIn
55 AntIn
55 AntIn
55 AntIn
56 AntIn
57 AntOut
57 AntOut
58 AntOut
58 AntOut
58 AntOut
59 AntIn
59 AntIn
59 AntIn
59 AntIn
60 AntIn
61 AntIn
61 AntIn

62	AntIn
62	AntIn
62	AntOut
62	AntOut
63	AntIn
63	AntIn
63	AntOut
64	AntIn
65	AntIn
65	AntIn
66	AntOut
67	AntIn
67	AntIn
67	AntOut
67	AntOut
68	AntIn
68	AntIn
68	AntIn
68	AntOut
69	AntIn
69	AntOut
69	AntOut
70	AntIn
70	AntOut
70	AntOut
71	AntIn
71	AntOut
71	AntOut
71	AntOut
72	AntIn
72	AntOut
73	AntIn
73	AntOut
73	AntOut
74	AntIn
74	AntIn
74	AntOut
74	AntOut
75	AntIn
75	AntIn
75	AntOut
75	AntOut

75	AntOut
76	AntIn
76	AntIn
77	AntOut
77	AntOut
77	AntOut
78	AntIn
78	AntIn
78	AntIn
78	AntOut
78	AntOut
79	AntIn
79	AntIn
80	AntIn
80	AntIn
81	AntIn
81	AntIn
82	AntIn
82	AntIn
82	AntIn
82	AntOut
83	AntIn
83	AntIn
83	AntOut
84	AntOut
84	AntOut
84	AntOut
85	AntIn
85	AntIn
85	AntOut
85	AntOut
86	AntOut
86	AntOut
86	AntOut
86	AntOut
87	AntIn
87	AntOut
88	AntOut
90	AntIn
90	AntIn
90	AntIn
90	AntOut

91	AntIn
91	AntOut
91	AntOut
92	AntIn
93	AntIn
93	AntIn
93	AntOut
93	AntOut
94	AntIn
94	AntOut
94	AntOut
94	AntOut
95	AntIn
96	AntIn
96	AntOut
97	AntIn
97	AntOut
97	AntOut
98	AntIn
98	AntOut
98	AntOut
98	AntOut
98	AntOut
101	AntIn
101	AntIn
101	AntOut
101	AntOut
103	AntIn
103	AntIn
103	AntIn
103	AntIn
103	AntOut
104	AntIn
104	AntOut
105	AntIn
105	AntOut
106	AntOut
106	AntOut
106	AntOut
107	AntIn
107	AntIn
108	AntIn

108	AntIn
108	AntOut
108	AntOut
109	AntIn
109	AntIn
109	AntOut
110	AntIn
110	AntIn
111	AntOut
112	AntOut
113	AntOut
113	AntOut
114	AntIn
114	AntIn
114	AntIn
114	AntIn
114	AntOut
115	AntIn
115	AntIn
115	AntIn
115	AntOut
116	AntIn
116	AntOut
116	AntOut
116	AntOut
116	AntOut
117	AntIn
117	AntOut
118	AntIn
118	AntIn
118	AntOut
118	AntOut
118	AntOut
119	AntIn
119	AntIn
120	AntIn
120	AntOut
120	AntOut
121	AntIn
121	AntOut
121	AntOut
122	AntIn

122 AntOut
122 AntOut
123 AntIn
123 AntIn
123 AntOut
124 AntIn
124 AntIn
125 AntIn
125 AntIn
125 AntOut
125 AntOut
125 AntOut
126 AntIn
126 AntIn
127 AntIn
127 AntOut
127 AntOut
128 AntIn
128 AntOut
128 AntOut
129 AntOut
129 AntOut
130 AntIn
130 AntIn
130 AntOut
131 AntIn
131 AntIn
131 AntOut
131 AntOut
132 AntIn
132 AntIn
132 AntOut
132 AntOut
133 AntIn
133 AntOut
133 AntOut
133 AntOut
134 AntIn
135 AntIn
135 AntIn
135 AntIn
135 AntOut

135	AntOut
136	AntOut
137	AntOut
138	AntIn
138	AntOut
138	AntOut
139	AntIn
139	AntIn
139	AntIn
139	AntOut
140	AntIn
140	AntOut
140	AntOut
141	AntIn
141	AntIn
141	AntOut
142	AntIn
142	AntIn
143	AntIn
143	AntIn
143	AntIn
143	AntIn
143	AntIn
143	AntOut
144	AntIn
144	AntOut
145	AntIn
145	AntOut
145	AntOut
145	AntOut
146	AntIn
146	AntIn
146	AntIn
146	AntOut
147	AntIn
147	AntIn
147	AntOut
147	AntOut
147	AntOut
148	AntIn
148	AntIn
148	AntIn

148	AntOut
149	AntIn
149	AntOut
149	AntOut
150	AntIn
150	AntOut
150	AntOut
152	AntOut
152	AntOut
153	AntIn
153	AntOut
153	AntOut
154	AntIn
154	AntOut
154	AntOut
155	AntIn
155	AntIn
155	AntOut
156	AntOut
156	AntOut
157	AntIn
157	AntOut
158	AntIn
158	AntIn
158	AntOut
158	AntOut
158	AntOut
158	AntOut
159	AntIn
159	AntOut
160	AntIn
160	AntIn
161	AntIn
161	AntIn
161	AntOut
161	AntOut
161	AntOut
161	AntOut
162	AntIn
162	AntOut
163	AntIn
163	AntIn

163	AntOut
164	AntIn
164	AntIn
164	AntIn
164	AntOut
164	AntOut
165	AntIn
165	AntIn
165	AntIn
165	AntIn
166	AntIn
166	AntIn
166	AntOut
168	AntIn
168	AntOut
168	AntOut
169	AntIn
169	AntIn
169	AntIn
169	AntIn
170	AntOut
170	AntOut
172	AntIn
172	AntOut
174	AntIn
174	AntIn
174	AntOut
175	AntIn
175	AntIn
175	AntOut
175	AntOut
176	AntIn
176	AntIn
176	AntIn
176	AntOut
176	AntOut
177	AntOut
177	AntOut
177	AntOut
178	AntOut
179	AntIn
179	AntIn

179	AntIn
179	AntOut
180	AntOut
181	AntIn
181	AntOut
181	AntOut
182	AntIn
182	AntIn
182	AntOut
183	AntIn
183	AntIn
183	AntIn
183	AntOut
184	AntIn
184	AntIn
184	AntIn
185	AntIn
185	AntOut
186	AntIn
186	AntIn
186	AntIn
186	AntOut
186	AntOut
187	AntOut
188	AntIn
188	AntOut
188	AntOut
188	AntOut
189	AntIn
190	AntIn
190	AntIn
190	AntIn
190	AntOut
191	AntOut
192	AntIn
192	AntOut
192	AntOut
192	AntOut
194	AntOut
194	AntOut
195	AntOut
196	AntIn

196	AntIn
197	AntIn
198	AntIn
198	AntOut
200	AntIn
200	AntOut
200	AntOut
200	AntOut
201	AntIn
201	AntIn
203	AntIn
203	AntIn
203	AntIn
203	AntOut
204	AntOut
205	AntIn
206	AntOut
206	AntOut
207	AntOut
207	AntOut
208	AntIn
209	AntIn
209	AntOut
209	AntOut
210	AntIn
210	AntIn
211	AntOut
211	AntOut
211	AntOut
211	AntOut
212	AntIn
212	AntOut
212	AntOut
213	AntIn
213	AntIn
214	AntIn
214	AntIn
214	AntIn
214	AntOut
215	AntOut
215	AntOut
216	AntIn

216	AntOut
216	AntOut
216	AntOut
217	AntOut
217	AntOut
217	AntOut
218	AntOut
218	AntOut
219	AntIn
219	AntOut
220	AntOut
221	AntIn
221	AntOut
222	AntOut
222	AntOut
222	AntOut
223	AntIn
224	AntIn
224	AntOut
225	AntIn
225	AntIn
225	AntIn
225	AntOut
226	AntIn
226	AntIn
226	AntIn
227	AntIn
227	AntOut
228	AntIn
228	AntIn
228	AntIn
228	AntIn
229	AntIn
229	AntIn
230	AntIn
230	AntOut
231	AntIn
231	AntIn
231	AntIn
231	AntOut
231	AntOut
231	AntOut

232 AntIn
232 AntOut
233 AntIn
233 AntOut
234 AntIn
234 AntIn
234 AntOut
235 AntIn
236 AntIn
236 AntIn
236 AntOut
237 AntIn
237 AntIn
237 AntOut
238 AntIn
238 AntOut
239 AntIn
240 AntIn
240 AntIn
240 AntIn
240 AntIn
240 AntIn
240 AntOut
241 AntIn
241 AntIn
241 AntIn
241 AntOut
241 AntOut
241 AntOut
242 AntIn
242 AntIn
242 AntIn
243 AntOut
243 AntOut
243 AntOut
244 AntIn
245 AntIn
245 AntOut
246 AntIn
246 AntOut
246 AntOut
247 AntOut

247	AntOut
247	AntOut
247	AntOut
247	AntOut
247	AntOut
248	AntIn
248	AntIn
248	AntOut
248	AntOut
248	AntOut
249	AntIn
249	AntOut
249	AntOut
249	AntOut
250	AntOut
250	AntOut
251	AntOut
251	AntOut
251	AntOut
251	AntOut
251	AntOut
252	AntIn
252	AntOut
253	AntOut
254	AntIn
254	AntIn
254	AntOut
254	AntOut
255	AntIn
255	AntIn
255	AntIn
256	AntIn
256	AntIn
256	AntOut
257	AntOut
258	AntOut
259	AntIn
259	AntIn
259	AntIn
259	AntOut
260	AntIn
260	AntIn

261 AntIn
261 AntIn
262 AntIn
262 AntIn
263 AntIn
263 AntOut
263 AntOut
264 AntOut
264 AntOut
264 AntOut
265 AntIn
265 AntOut
266 AntIn
266 AntIn
267 AntIn
267 AntOut
267 AntOut
267 AntOut
267 AntOut
268 AntIn
269 AntOut
269 AntOut
270 AntIn
270 AntIn
270 AntIn
270 AntOut
272 AntIn
272 AntIn
272 AntIn
273 AntIn
273 AntIn
273 AntIn
273 AntIn
273 AntIn
274 AntIn
274 AntOut
275 AntIn
275 AntOut
275 AntOut
275 AntOut
276 AntIn
276 AntIn

276	AntIn
276	AntIn
276	AntIn
276	AntOut
277	AntIn
277	AntIn
277	AntIn
277	AntIn
277	AntOut
277	AntOut
277	AntOut
278	AntIn
278	AntIn
278	AntIn
278	AntOut
278	AntOut
279	AntIn
279	AntOut
279	AntOut
280	AntIn
280	AntIn
280	AntIn
281	AntOut
281	AntOut
281	AntOut
282	AntIn
282	AntIn
282	AntIn
283	AntIn
283	AntIn
283	AntOut
283	AntOut
284	AntIn
284	AntIn
284	AntIn
284	AntIn
285	AntIn
285	AntIn
286	AntIn
286	AntOut
286	AntOut
287	AntIn

287 AntIn
288 AntIn
288 AntIn
288 AntOut
289 AntIn
289 AntIn
289 AntOut
290 AntIn
290 AntIn
290 AntOut
290 AntOut
290 AntOut
290 AntOut
291 AntIn
292 AntIn
292 AntOut
292 AntOut
293 AntIn
293 AntIn
293 AntOut
294 AntIn
294 AntIn
295 AntIn
295 AntIn
295 AntIn
296 AntIn
296 AntIn
297 AntIn
297 AntIn
297 AntOut
297 AntOut
298 AntIn
298 AntOut
299 AntIn
299 AntOut
299 AntOut
300 AntOut
300 AntOut
300 AntOut
300 AntOut
302 AntIn
303 AntIn

304 AntIn
304 AntIn
304 AntIn
304 AntIn
304 AntOut
304 AntOut
305 AntIn
305 AntOut
305 AntOut
306 AntIn
306 AntOut
307 AntIn
307 AntIn
307 AntOut
307 AntOut
309 AntIn
309 AntOut
310 AntIn
310 AntOut
310 AntOut
311 AntOut
312 AntIn
312 AntOut
312 AntOut
313 AntIn
313 AntIn
314 AntIn
314 AntIn
314 AntOut
315 AntIn
315 AntOut
315 AntOut
315 AntOut
316 AntIn
317 AntIn
318 AntIn
318 AntIn
318 AntOut
318 AntOut
318 AntOut
319 AntIn
319 AntIn

319	AntOut
319	AntOut
319	AntOut
319	AntOut
319	AntOut
320	AntIn
320	AntIn
320	AntIn
320	AntOut
320	AntOut
321	AntIn
321	AntIn
321	AntIn
321	AntIn
321	AntOut
322	AntIn
322	AntOut
323	AntIn
324	AntIn
324	AntIn
324	AntOut
325	AntIn
325	AntOut
326	AntIn
326	AntIn
326	AntIn
326	AntOut
327	AntIn
327	AntOut
328	AntOut
329	AntOut
330	AntIn
330	AntIn
330	AntIn
330	AntIn
330	AntOut
331	AntIn
332	AntIn
332	AntIn
332	AntIn
332	AntIn
333	AntIn

333 AntOut
334 AntIn
334 AntIn
335 AntIn
335 AntOut
336 AntIn
337 AntOut
338 AntIn
338 AntIn
338 AntOut
339 AntOut
339 AntOut
340 AntIn
340 AntIn
341 AntIn
341 AntIn
341 AntOut
341 AntOut
342 AntIn
342 AntIn
342 AntOut
343 AntOut
344 AntIn
344 AntOut
345 AntOut
346 AntIn
346 AntOut
346 AntOut
346 AntOut
347 AntIn
347 AntOut
348 AntOut
348 AntOut
348 AntOut
349 AntIn
349 AntOut
350 AntIn
350 AntOut
350 AntOut
350 AntOut
350 AntOut
351 AntIn

352	AntIn
352	AntIn
352	AntIn
353	AntIn
353	AntIn
353	AntIn
353	AntOut
354	AntIn
354	AntOut
355	AntIn
355	AntIn
355	AntIn
355	AntOut
355	AntOut
355	AntOut
356	AntIn
357	AntIn
357	AntOut
358	AntIn
358	AntIn
358	AntOut
359	AntIn
359	AntIn
359	AntIn
359	AntOut
359	AntOut
360	AntIn
360	AntIn
360	AntIn
360	AntOut
360	AntOut
360	AntOut
361	AntIn
362	AntIn
362	AntIn
362	AntOut
362	AntOut
363	AntIn
363	AntIn
363	AntIn
363	AntIn
363	AntIn

364	AntIn
364	AntOut
364	AntOut
365	AntIn
365	AntOut
365	AntOut
365	AntOut
365	AntOut
366	AntIn
366	AntIn
366	AntIn
366	AntOut
366	AntOut
367	AntIn
367	AntOut
367	AntOut
367	AntOut
368	AntIn
368	AntIn
368	AntIn
368	AntIn
369	AntIn
369	AntIn
369	AntIn
369	AntOut
370	AntIn
370	AntIn
370	AntIn
371	AntIn
371	AntIn
371	AntIn
372	AntIn
372	AntIn
372	AntIn
372	AntIn
373	AntIn
373	AntIn
373	AntIn
373	AntOut
374	AntIn
375	AntIn
375	AntIn

375	AntOut
376	AntIn
376	AntIn
377	AntIn
377	AntIn
378	AntIn
378	AntIn
378	AntOut
378	AntOut
379	AntIn
379	AntOut
380	AntIn
380	AntIn
380	AntOut
381	AntIn
381	AntOut
381	AntOut
381	AntOut
382	AntOut
382	AntOut
382	AntOut
383	AntOut
383	AntOut
384	AntIn
384	AntIn
384	AntIn
384	AntIn
384	AntOut
385	AntOut
385	AntOut
385	AntOut
385	AntOut
386	AntIn
386	AntIn
386	AntOut
387	AntIn
387	AntOut
387	AntOut
387	AntOut
388	AntOut
388	AntOut
388	AntOut

389	AntIn
389	AntIn
389	AntIn
389	AntOut
390	AntOut
390	AntOut
390	AntOut
390	AntOut
391	AntIn
391	AntIn
391	AntIn
392	AntIn
392	AntOut
392	AntOut
393	AntIn
393	AntIn
393	AntOut
393	AntOut
394	AntIn
394	AntIn
394	AntIn
394	AntIn
395	AntIn
395	AntOut
395	AntOut
395	AntOut
396	AntIn
396	AntOut
396	AntOut
396	AntOut
396	AntOut
397	AntOut
397	AntOut
398	AntIn
398	AntOut
398	AntOut
399	AntOut
399	AntOut
400	AntIn
400	AntIn
400	AntIn
400	AntOut

401	AntIn
401	AntIn
401	AntOut
401	AntOut
402	AntIn
402	AntIn
402	AntOut
403	AntIn
403	AntIn
404	AntIn
404	AntIn
404	AntOut
405	AntIn
405	AntIn
405	AntIn
405	AntIn
405	AntIn
405	AntOut
405	AntOut
406	AntOut
407	AntIn
408	AntIn
409	AntIn
409	AntIn
409	AntIn
409	AntOut
410	AntIn
410	AntIn
410	AntOut
411	AntIn
411	AntIn
411	AntOut
412	AntIn
413	AntOut
414	AntIn
414	AntIn
414	AntIn
414	AntOut
415	AntIn
415	AntIn
415	AntOut
415	AntOut

415	AntOut
416	AntIn
416	AntOut
416	AntOut
417	AntIn
417	AntOut
418	AntIn
418	AntOut
418	AntOut
418	AntOut
419	AntOut
420	AntIn
420	AntIn
420	AntIn
420	AntOut
421	AntIn
422	AntOut
423	AntIn
423	AntOut
423	AntOut
424	AntIn
424	AntIn
424	AntIn
424	AntIn
424	AntOut
425	AntIn
425	AntIn
426	AntIn
427	AntIn
429	AntIn
429	AntOut
429	AntOut
430	AntIn
430	AntOut
431	AntIn
431	AntIn
432	AntOut
432	AntOut
432	AntOut
433	AntIn
433	AntOut
434	AntIn

434 AntIn
434 AntOut
434 AntOut
434 AntOut
435 AntIn
435 AntOut
435 AntOut
436 AntIn
436 AntIn
437 AntOut
437 AntOut
437 AntOut
438 AntIn
438 AntIn
439 AntIn
439 AntIn
439 AntIn
439 AntIn
441 AntIn
442 AntIn
442 AntOut
443 AntIn
444 AntIn
444 AntOut
445 AntIn
446 AntIn
447 AntIn
447 AntIn
447 AntOut
447 AntOut
447 AntOut
447 AntOut
448 AntIn
448 AntIn
448 AntOut
449 AntIn
449 AntIn
449 AntOut
450 AntOut
451 AntIn
451 AntOut
451 AntOut

452 AntIn
452 AntIn
453 AntIn
453 AntIn
453 AntIn
453 AntIn
453 AntIn
453 AntOut
454 AntIn
455 AntIn
455 AntIn
455 AntOut
456 AntIn
456 AntOut
456 AntOut
457 AntIn
457 AntOut
457 AntOut
459 AntIn
460 AntIn
460 AntOut
460 AntOut
460 AntOut
460 AntOut
461 AntIn
461 AntIn
461 AntOut
461 AntOut
462 AntOut
463 AntIn
464 AntIn
464 AntOut
464 AntOut
464 AntOut
465 AntIn
465 AntIn
466 AntIn
467 AntIn
467 AntIn
467 AntOut
468 AntIn
469 AntIn

469	AntIn
469	AntOut
469	AntOut
469	AntOut
470	AntIn
470	AntOut
470	AntOut
471	AntIn
471	AntOut
471	AntOut
471	AntOut
471	AntOut
473	AntIn
473	AntIn
473	AntIn
473	AntOut
479	AntIn
481	AntIn
481	AntIn
481	AntIn
481	AntOut
482	AntIn
482	AntIn
483	AntIn
483	AntIn
483	AntIn
484	AntIn
484	AntIn
484	AntOut
484	AntOut
485	AntIn
485	AntOut
486	AntIn
486	AntIn
486	AntIn
487	AntIn
487	AntIn
487	AntOut
488	AntOut
488	AntOut
489	AntIn
490	AntIn

490 AntIn
490 AntOut
492 AntOut
492 AntOut
493 AntIn
493 AntOut
493 AntOut
494 AntIn
494 AntOut
494 AntOut
494 AntOut
495 AntIn
495 AntOut
495 AntOut
496 AntIn
496 AntOut
497 AntIn
497 AntOut
497 AntOut
498 AntIn
498 AntOut
498 AntOut
499 AntIn
500 AntIn
500 AntOut
500 AntOut
501 AntOut
501 AntOut
501 AntOut
502 AntIn
502 AntIn
502 AntOut
503 AntIn
503 AntIn
504 AntIn
504 AntIn
504 AntIn
505 AntIn
505 AntIn
505 AntIn
505 AntOut
505 AntOut

506	AntIn
506	AntOut
506	AntOut
506	AntOut
507	AntIn
507	AntIn
508	AntIn
508	AntIn
508	AntIn
508	AntOut
509	AntIn
509	AntIn
509	AntIn
509	AntOut
510	AntIn
510	AntIn
510	AntIn
511	AntIn
511	AntOut
511	AntOut
512	AntIn
512	AntOut
513	AntIn
513	AntOut
514	AntOut
514	AntOut
514	AntOut
515	AntIn
515	AntIn
515	AntIn
515	AntIn
516	AntIn
516	AntIn
516	AntOut
517	AntIn
517	AntIn
517	AntIn
517	AntIn
517	AntOut
519	AntIn
519	AntIn
519	AntIn

519	AntIn
520	AntIn
520	AntOut
521	AntIn
521	AntOut
521	AntOut
521	AntOut
522	AntIn
522	AntIn
522	AntIn
522	AntOut
523	AntOut
524	AntIn
525	AntIn
525	AntIn
526	AntIn
526	AntOut
526	AntOut
527	AntIn
527	AntIn
527	AntIn
527	AntOut
528	AntIn
528	AntIn
528	AntOut
528	AntOut
528	AntOut
528	AntOut
528	AntOut
529	AntIn
529	AntOut
530	AntIn
530	AntOut
530	AntOut
531	AntIn
531	AntOut
532	AntOut
533	AntIn
533	AntIn
533	AntOut
534	AntOut
534	AntOut

535 AntIn
535 AntOut
535 AntOut
535 AntOut
536 AntIn
536 AntOut
536 AntOut
537 AntIn
537 AntOut
538 AntIn
538 AntIn
539 AntIn
539 AntIn
539 AntOut
539 AntOut
540 AntIn
540 AntIn
540 AntOut
540 AntOut
541 AntIn
541 AntIn
541 AntOut
541 AntOut
542 AntIn
542 AntIn
543 AntIn
544 AntIn
544 AntIn
544 AntIn
544 AntOut
545 AntIn
547 AntOut
547 AntOut
547 AntOut
548 AntOut
548 AntOut
549 AntIn
549 AntIn
549 AntOut
549 AntOut
550 AntOut
551 AntOut

551 AntOut
552 AntOut
552 AntOut
553 AntIn
553 AntIn
553 AntIn
553 AntIn
553 AntIn
554 AntIn
554 AntIn
554 AntOut
554 AntOut
555 AntOut
555 AntOut
556 AntIn
556 AntIn
556 AntOut
556 AntOut
557 AntIn
557 AntOut
558 AntIn
558 AntIn
558 AntIn
559 AntIn
559 AntIn
559 AntIn
559 AntIn
560 AntIn
560 AntIn
561 AntOut
561 AntOut
562 AntIn
563 AntIn
563 AntIn
564 AntIn
564 AntIn
564 AntIn
564 AntOut
565 AntIn
565 AntIn
565 AntIn
565 AntOut

566 AntIn
567 AntIn
567 AntIn
568 AntIn
569 AntIn
569 AntIn
569 AntIn
569 AntIn
569 AntOut
570 AntIn
571 AntIn
572 AntIn
572 AntOut
572 AntOut
572 AntOut
572 AntOut
574 AntIn
574 AntIn
574 AntIn
575 AntIn
575 AntOut
576 AntOut
577 AntIn
577 AntIn
577 AntIn
577 AntOut
578 AntIn
578 AntIn
578 AntOut
578 AntOut
579 AntIn
579 AntIn
580 AntIn
580 AntIn
580 AntOut
580 AntOut
580 AntOut
582 AntIn
582 AntIn
582 AntOut
583 AntOut
584 AntIn

584 AntOut
585 AntIn
586 AntIn
587 AntIn
588 AntIn
588 AntOut
588 AntOut
589 AntOut
590 AntIn
590 AntIn
591 AntOut
592 AntIn
592 AntIn
592 AntOut
593 AntIn
593 AntOut
594 AntOut
594 AntOut
595 AntIn
595 AntIn
595 AntOut
595 AntOut
597 AntIn
598 AntIn
598 AntIn
598 AntIn
599 AntIn
599 AntIn
599 AntIn
599 AntIn
599 AntIn
599 AntIn
600 AntOut
601 AntIn
601 AntOut
601 AntOut
602 AntIn
604 AntIn
604 AntIn
604 AntOut
604 AntOut
605 AntIn

605 AntIn
606 AntIn
606 AntIn
606 AntIn
606 AntIn
607 AntIn
608 AntIn
609 AntOut
610 AntIn
610 AntIn
611 AntOut
612 AntIn
612 AntOut
612 AntOut
613 AntOut
613 AntOut
614 AntIn
614 AntOut
615 AntIn
615 AntIn
615 AntOut
615 AntOut
616 AntIn
617 AntIn
618 AntIn
618 AntIn
618 AntOut
618 AntOut
619 AntIn
619 AntOut
619 AntOut
619 AntOut
620 AntIn
620 AntIn
620 AntIn
620 AntIn
620 AntIn
621 AntIn
621 AntIn
621 AntIn
621 AntIn
622 AntIn

622 AntIn
622 AntOut
623 AntIn
623 AntIn
623 AntOut
624 AntOut
624 AntOut
626 AntIn
627 AntIn
628 AntIn
628 AntIn
628 AntIn
629 AntIn
629 AntIn
630 AntIn
630 AntOut
630 AntOut
631 AntIn
632 AntIn
632 AntIn
632 AntIn
633 AntIn
633 AntIn
634 AntIn
634 AntOut
634 AntOut
635 AntIn
635 AntIn
635 AntOut
636 AntIn
636 AntOut
636 AntOut
637 AntOut
637 AntOut
637 AntOut
638 AntIn
639 AntIn
640 AntIn
640 AntIn
640 AntIn
641 AntOut
643 AntIn

643 AntIn
644 AntIn
644 AntOut
644 AntOut
645 AntIn
645 AntOut
645 AntOut
645 AntOut
646 AntIn
646 AntOut
646 AntOut
647 AntOut
648 AntIn
648 AntOut
649 AntIn
649 AntIn
649 AntOut
649 AntOut
650 AntOut
650 AntOut
651 AntIn
651 AntIn
652 AntIn
653 AntIn
653 AntOut
654 AntIn
654 AntIn
654 AntIn
654 AntIn
654 AntOut
654 AntOut
655 AntIn
655 AntIn
656 AntIn
656 AntIn
656 AntOut
657 AntIn
657 AntIn
657 AntIn
657 AntOut
658 AntIn
658 AntIn

658 AntOut
659 AntOut
659 AntOut
660 AntIn
660 AntIn
660 AntIn
661 AntIn
661 AntIn
662 AntIn
663 AntIn
663 AntIn
663 AntOut
663 AntOut
663 AntOut
664 AntIn
664 AntOut
665 AntIn
665 AntIn
669 AntIn
670 AntIn
670 AntOut
671 AntIn
671 AntOut
672 AntOut
672 AntOut
673 AntIn
673 AntIn
674 AntIn
674 AntIn
674 AntIn
674 AntIn
675 AntOut
676 AntIn
676 AntIn
677 AntIn
677 AntIn
678 AntIn
678 AntIn
678 AntIn
678 AntIn
678 AntOut
679 AntOut

680 AntIn
681 AntIn
681 AntIn
681 AntOut
682 AntOut
683 AntOut
684 AntIn
685 AntIn
685 AntIn
685 AntOut
686 AntIn
686 AntIn
686 AntIn
686 AntOut
687 AntOut
689 AntOut
690 AntIn
690 AntOut
691 AntIn
691 AntIn
691 AntOut
691 AntOut
691 AntOut
692 AntIn
693 AntIn
694 AntIn
694 AntOut
695 AntIn
696 AntIn
697 AntIn
697 AntIn
697 AntOut
698 AntIn
698 AntIn
698 AntIn
701 AntOut
702 AntIn
702 AntIn
702 AntOut
703 AntIn
703 AntOut
704 AntIn

704	AntIn
704	AntOut
705	AntIn
705	AntIn
706	AntIn
706	AntIn
706	AntOut
707	AntOut
707	AntOut
707	AntOut
707	AntOut
708	AntIn
708	AntOut
708	AntOut
708	AntOut
708	AntOut
709	AntIn
710	AntIn
710	AntIn
710	AntIn
711	AntIn
711	AntIn
711	AntOut
711	AntOut
712	AntIn
712	AntOut
712	AntOut
713	AntIn
713	AntOut
713	AntOut
714	AntIn
714	AntOut
715	AntIn
715	AntIn
716	AntIn
716	AntOut
716	AntOut
716	AntOut
717	AntIn
718	AntIn
718	AntOut
718	AntOut

719	AntIn
719	AntOut
719	AntOut
720	AntIn
720	AntIn
720	AntIn
720	AntIn
721	AntIn
721	AntIn
721	AntOut
721	AntOut
721	AntOut
722	AntIn
723	AntOut
724	AntIn
724	AntIn
724	AntIn
725	AntIn
725	AntIn
726	AntIn
726	AntIn
726	AntIn
727	AntIn
727	AntIn
727	AntIn
727	AntOut
727	AntOut
727	AntOut
728	AntIn
728	AntIn
728	AntOut
729	AntIn
729	AntIn
729	AntIn
729	AntIn
730	AntIn
730	AntIn
730	AntIn
730	AntOut
730	AntOut
731	AntIn
731	AntOut

732	AntIn
732	AntOut
732	AntOut
733	AntIn
733	AntIn
733	AntOut
733	AntOut
733	AntOut
734	AntIn
735	AntIn
735	AntIn
735	AntIn
735	AntOut
736	AntIn
736	AntIn
736	AntIn
736	AntIn
736	AntIn
736	AntOut
737	AntIn
737	AntOut
738	AntIn
739	AntIn
739	AntIn
739	AntIn
739	AntOut
740	AntIn
740	AntIn
741	AntIn
741	AntIn
741	AntIn
741	AntIn
741	AntIn
741	AntIn
742	AntIn
742	AntIn
742	AntIn
742	AntIn
742	AntIn
743	AntIn
743	AntOut
743	AntOut

743 AntOut
744 AntIn
744 AntIn
744 AntOut
744 AntOut
745 AntIn
745 AntOut
746 AntIn
746 AntIn
746 AntOut
747 AntIn
747 AntIn
748 AntIn
748 AntIn
748 AntIn
748 AntIn
749 AntIn
749 AntIn
749 AntOut
749 AntOut
751 AntIn
751 AntIn
752 AntIn
752 AntIn
752 AntIn
753 AntIn
753 AntIn
753 AntIn
753 AntOut
753 AntOut
754 AntOut
755 AntIn
755 AntIn
755 AntIn
755 AntOut
755 AntOut
756 AntOut
756 AntOut
756 AntOut
757 AntIn
757 AntIn
758 AntIn

758	AntIn
759	AntIn
759	AntIn
759	AntIn
759	AntIn
759	AntOut
760	AntIn
760	AntIn
761	AntIn
761	AntIn
761	AntOut
762	AntIn
762	AntIn
762	AntOut
763	AntOut
764	AntIn
764	AntOut
765	AntIn
765	AntIn
766	AntIn
766	AntIn
766	AntIn
766	AntIn
767	AntIn
767	AntIn
767	AntIn
767	AntOut
768	AntIn
768	AntIn
768	AntOut
768	AntOut
769	AntIn
769	AntIn
769	AntIn
769	AntOut
769	AntOut
770	AntIn
771	AntIn
771	AntIn
771	AntOut
772	AntIn
773	AntIn

774	AntIn
774	AntIn
774	AntOut
775	AntIn
775	AntOut
776	AntIn
776	AntOut
777	AntIn
777	AntIn
777	AntOut
778	AntIn
778	AntIn
778	AntIn
778	AntOut
778	AntOut
779	AntIn
779	AntOut
780	AntOut
781	AntIn
782	AntIn
782	AntIn
783	AntIn
783	AntOut
783	AntOut
784	AntOut
785	AntOut
785	AntOut
786	AntIn
786	AntOut
788	AntIn
788	AntIn
789	AntIn
789	AntIn
790	AntIn
790	AntOut
790	AntOut
790	AntOut
791	AntIn
791	AntOut
791	AntOut
791	AntOut
792	AntIn

793	AntIn
793	AntIn
793	AntOut
794	AntIn
794	AntIn
794	AntIn
794	AntOut
795	AntIn
795	AntOut
795	AntOut
796	AntIn
796	AntOut
797	AntIn
797	AntIn
797	AntIn
797	AntOut
798	AntOut
800	AntIn
800	AntOut
800	AntOut
801	AntIn
801	AntIn
802	AntIn
803	AntIn
803	AntOut
803	AntOut
804	AntIn
805	AntIn
805	AntIn
805	AntIn
806	AntOut
807	AntIn
807	AntIn
807	AntOut
808	AntIn
808	AntIn
809	AntIn
810	AntIn
810	AntIn
811	AntIn
811	AntOut
812	AntIn

813 AntOut
813 AntOut
814 AntIn
814 AntIn
814 AntIn
815 AntIn
815 AntIn
815 AntIn
817 AntIn
817 AntIn
817 AntOut
817 AntOut
817 AntOut
818 AntOut
819 AntIn
820 AntIn
820 AntOut
821 AntIn
821 AntOut
822 AntOut
822 AntOut
822 AntOut
822 AntOut
823 AntIn
823 AntIn
824 AntIn
825 AntOut
826 AntOut
827 AntIn
827 AntIn
827 AntOut
828 AntIn
828 AntIn
828 AntOut
829 AntIn
829 AntIn
829 AntIn
830 AntIn
830 AntIn
830 AntIn
831 AntIn
831 AntIn

831 AntOut
832 AntOut
833 AntIn
833 AntIn
833 AntIn
834 AntIn
834 AntIn
834 AntIn
835 AntOut
836 AntIn
836 AntOut
837 AntIn
837 AntIn
837 AntIn
837 AntIn
837 AntOut
838 AntIn
838 AntIn
838 AntOut
839 AntOut
839 AntOut
840 AntIn
840 AntIn
841 AntIn
841 AntOut
844 AntIn
844 AntOut
844 AntOut
845 AntOut
846 AntOut
846 AntOut
846 AntOut
847 AntIn
847 AntIn
848 AntIn
848 AntIn
848 AntOut
849 AntIn
849 AntOut
850 AntIn
850 AntIn
850 AntIn

850 AntIn
851 AntIn
851 AntOut
851 AntOut
852 AntIn
852 AntIn
852 AntOut
852 AntOut
853 AntOut
854 AntOut
855 AntIn
855 AntIn
855 AntIn
856 AntIn
857 AntIn
857 AntOut
858 AntIn
858 AntIn
859 AntIn
859 AntIn
859 AntIn
859 AntIn
860 AntIn
860 AntIn
861 AntIn
861 AntIn
861 AntOut
862 AntIn
862 AntIn
863 AntOut
863 AntOut
864 AntIn
864 AntOut
865 AntIn
865 AntIn
866 AntIn
866 AntIn
866 AntIn
866 AntIn
866 AntOut
867 AntOut
868 AntIn

869 AntIn
869 AntOut
869 AntOut
869 AntOut
870 AntIn
870 AntOut
871 AntIn
871 AntIn
871 AntIn
871 AntIn
871 AntIn
872 AntIn
872 AntIn
872 AntIn
872 AntOut
873 AntIn
874 AntIn
874 AntOut
875 AntIn
875 AntIn
875 AntOut
876 AntIn
876 AntOut
876 AntOut
876 AntOut
877 AntIn
877 AntOut
877 AntOut
878 AntIn
878 AntOut
879 AntIn
879 AntOut
880 AntIn
880 AntIn
880 AntIn
880 AntOut
881 AntOut
881 AntOut
881 AntOut
881 AntOut
882 AntOut
882 AntOut

882 AntOut
883 AntOut
883 AntOut
883 AntOut
883 AntOut
884 AntIn
884 AntIn
885 AntIn
885 AntIn
885 AntOut
886 AntIn
886 AntOut
887 AntOut
888 AntOut
888 AntOut
888 AntOut
888 AntOut
889 AntIn
889 AntOut
889 AntOut
890 AntOut
890 AntOut
890 AntOut
891 AntIn
891 AntIn
891 AntIn
891 AntIn
891 AntIn
891 AntOut
892 AntIn
892 AntOut
893 AntIn
893 AntIn
894 AntIn
894 AntOut
895 AntIn
896 AntIn
896 AntIn
896 AntIn
896 AntOut
897 AntIn
897 AntOut

898	AntIn
898	AntOut
899	AntIn
899	AntIn
900	AntOut
900	AntOut
901	AntIn
901	AntIn
901	AntOut
901	AntOut
902	AntOut
903	AntIn
903	AntIn
903	AntOut
905	AntIn
905	AntIn
905	AntOut
906	AntIn
906	AntIn
906	AntOut
907	AntIn
908	AntIn
908	AntOut
908	AntOut
909	AntIn
909	AntOut
910	AntIn
911	AntIn
911	AntIn
911	AntIn
911	AntOut
911	AntOut
912	AntIn
912	AntIn
913	AntIn
913	AntIn
913	AntIn
914	AntIn
914	AntOut
914	AntOut
915	AntOut
