# Supplementary material for: Interactions Increase Forager Availability and Activity in Harvester Ants
Source: PLoS One. 2015 Nov 5;10(11):e0141971. doi: 10.1371/journal.pone.0141971 (PMC4635008; doi:10.1371/journal.pone.0141971)
Supplement: S3 Dataset — We observed and filmed behavior inside the nest during and after forager removals. This dataset shows our counts made from the films of the numbers of returning and outgoing foragers at the nest entrance and the number of ascending and descending ants at all tunnel entrances. (ZIP) [file pone.0141971.s004.zip › S3 Dataset/2013 Correlation Data N5 8-20.pdf]

**Researcher**	**Jovel Queirolo**
**Colony N5**	
**8/20/13**	
**Video time (seconds)**	**Event**
5	Ascend
7	Descend
7	Ascend
9	Ascend
10	Ascend
11	Ascend
11	Ascend
12	Ascend
12	Ascend
13	Ascend
15	Ascend
15	Ascend
16	Ascend
16	Descend
17	Descend
17	Ascend
18	Ascend
18	Descend
19	Descend
19	Descend
21	Descend
22	Ascend
22	Ascend
23	Ascend
24	Ascend
25	Ascend
26	Ascend
26	Ascend
26	Ascend
27	Ascend
27	Ascend
28	Ascend
28	Ascend
29	Ascend
30	Descend
31	Descend
31	Descend

31	Descend
32	Ascend
32	Ascend
33	Ascend
35	Ascend
35	Ascend
36	Ascend
37	Ascend
38	Ascend
38	Descend
39	Descend
39	Descend
40	Ascend
40	Ascend
40	Ascend
41	Ascend
41	Ascend
42	Ascend
43	Ascend
44	Ascend
45	Ascend
46	Descend
46	Descend
47	Descend
47	Ascend
47	Ascend
47	Ascend
48	Ascend
49	Ascend
49	Ascend
50	Ascend
50	Ascend
50	Ascend
51	Ascend
52	Ascend
52	Descend
52	Descend
53	Descend
53	Ascend
53	Ascend
54	Ascend
54	Descend

55	Descend
55	Descend
56	Ascend
56	Ascend
56	Ascend
57	Ascend
57	Ascend
58	Ascend
58	Ascend
59	Ascend
59	Ascend
60	Ascend
60	Ascend
60	Descend
60	Descend
61	Ascend
61	Ascend
62	Descend
63	Descend
64	Descend
64	Ascend
65	Ascend
65	Ascend
66	Ascend
66	Ascend
67	Ascend
68	Ascend
69	Ascend
69	Ascend
70	Ascend
70	Ascend
71	Ascend
72	Ascend
73	Ascend
73	Ascend
73	Ascend
74	Ascend
74	Ascend
75	Ascend
75	Ascend
75	Descend
76	Descend

76	Descend
77	Descend
77	Ascend
77	Ascend
78	Ascend
78	Ascend
79	Ascend
79	Ascend
79	Ascend
79	Ascend
80	Ascend
80	Ascend
80	Ascend
81	Ascend
81	Ascend
82	Ascend
82	Ascend
83	Ascend
83	Ascend
84	Ascend
84	Ascend
84	Ascend
84	Ascend
85	Ascend
85	Ascend
86	Ascend
86	Ascend
87	Ascend
87	Ascend
88	Ascend
89	Ascend
90	Descend
90	Descend
91	Descend
91	Descend
91	Descend
92	Descend
92	Descend
92	Descend
93	Descend
93	Ascend
93	Ascend

94	Ascend
94	Ascend
94	Ascend
95	Ascend
95	Ascend
95	Ascend
96	Ascend
96	Ascend
97	Descend
97	Descend
97	Descend
97	Descend
98	Ascend
98	Ascend
98	Ascend
98	Ascend
99	Ascend
99	Ascend
100	Ascend
100	Ascend
100	Descend
101	Descend
101	Descend
102	Ascend
102	Ascend
105	Descend
106	Ascend
107	Descend
107	Ascend
109	Descend
110	Descend
110	Descend
110	Descend
111	Descend
111	Descend
112	Descend
112	Descend
113	Ascend
113	Ascend
113	Ascend
114	Ascend
114	Ascend

115	Ascend
116	Descend
116	Descend
117	Descend
117	Descend
118	Descend
118	Descend
119	Descend
119	Ascend
120	Ascend
120	Ascend
121	Descend
121	Descend
121	Descend
122	Ascend
122	Ascend
122	Ascend
123	Ascend
124	Ascend
124	Descend
124	Descend
125	Descend
126	Descend
126	Ascend
127	Ascend
127	Ascend
128	Ascend
129	Ascend
129	Ascend
130	Ascend
131	Ascend
132	Ascend
132	Ascend
133	Ascend
133	Descend
134	Descend
134	Descend
134	Descend
135	Descend
135	Descend
136	Descend
136	Descend

137	Ascend
137	Ascend
137	Descend
138	Descend
138	Descend
138	Descend
138	Descend
139	Descend
139	Descend
139	Descend
140	Descend
140	Descend
141	Ascend
142	Ascend
142	Ascend
142	Descend
142	Descend
143	Descend
143	Descend
143	Descend
144	Descend
144	Descend
146	Ascend
146	Ascend
147	Ascend
147	Ascend
147	Ascend
148	Ascend
148	Ascend
148	Ascend
149	Ascend
149	Ascend
150	Ascend
150	Ascend
150	Ascend
150	Ascend
151	Ascend
152	Ascend
152	Ascend
154	Ascend
154	Ascend
154	Ascend

155	Ascend
155	Ascend
155	Ascend
156	Ascend
157	Descend
157	Descend
157	Descend
158	Descend
158	Descend
159	Descend
159	Descend
159	Ascend
160	Ascend
160	Ascend
160	Ascend
160	Ascend
161	Ascend
161	Ascend
162	Ascend
162	Ascend
163	Ascend
163	Ascend
163	Descend
164	Descend
164	Descend
165	Descend
166	Ascend
166	Ascend
167	Ascend
167	Ascend
167	Ascend
168	Ascend
168	Ascend
168	Ascend
168	Ascend
169	Ascend
169	Ascend
169	Ascend
170	Ascend
170	Ascend
171	Ascend
174	Ascend

175	Descend
176	Descend
177	Descend
178	Ascend
178	Ascend
179	Ascend
180	Ascend
182	Ascend
182	Ascend
184	Descend
185	Descend
186	Descend
186	Descend
186	Descend
187	Ascend
187	Ascend
190	Ascend
190	Ascend
191	Ascend
193	Descend
193	Descend
193	Descend
194	Descend
194	Descend
195	Descend
195	Ascend
195	Ascend
196	Descend
196	Descend
196	Descend
197	Descend
197	Descend
198	Descend
198	Descend
200	Descend
201	Ascend
202	Ascend
203	Descend
204	Descend
205	Descend
205	Ascend
206	Ascend

206	Ascend
207	Ascend
207	Ascend
207	Ascend
208	Descend
208	Descend
210	Ascend
211	Ascend
211	Ascend
211	Ascend
211	Ascend
212	Ascend
214	Ascend
215	Ascend
216	Ascend
217	Descend
218	Descend
218	Descend
219	Ascend
221	Ascend
222	Ascend
222	Ascend
223	Ascend
223	Ascend
223	Ascend
224	Ascend
224	Ascend
224	Ascend
225	Ascend
225	Ascend
226	Ascend
226	Ascend
227	Ascend
227	Ascend
228	Ascend
228	Ascend
230	Ascend
232	Ascend
232	Ascend
233	Ascend
233	Descend
233	Descend

234	Descend
234	Descend
235	Ascend
235	Ascend
237	Ascend
237	Ascend
237	Ascend
238	Ascend
238	Ascend
239	Ascend
240	Ascend
241	Ascend
241	Descend
241	Descend
242	Descend
242	Descend
243	Ascend
243	Ascend
245	Ascend
246	Ascend
246	Ascend
246	Ascend
247	Ascend
247	Descend
248	Descend
248	Descend
249	Descend
249	Descend
249	Descend
251	Descend
251	Descend
251	Ascend
251	Ascend
254	Descend
254	Descend
254	Descend
255	Descend
256	Descend
257	Descend
257	Descend
258	Descend
258	Descend

258	Descend
258	Descend
259	Descend
259	Descend
259	Descend
260	Descend
263	Descend
263	Descend
263	Descend
263	Descend
265	Descend
265	Descend
265	Ascend
266	Ascend
266	Ascend
267	Ascend
267	Ascend
267	Ascend
267	Ascend
268	Ascend
268	Ascend
268	Ascend
268	Ascend
269	Ascend
269	Ascend
270	Ascend
270	Ascend
271	Ascend
271	Ascend
272	Ascend
272	Ascend
274	Ascend
274	Ascend
275	Ascend
276	Ascend
276	Ascend
277	Ascend
278	Ascend
279	Ascend
279	Ascend
280	Descend
280	Descend

280	Descend
281	Descend
281	Descend
283	Descend
283	Descend
285	Ascend
285	Ascend
285	Ascend
285	Ascend
286	Ascend
287	Ascend
287	Ascend
288	Ascend
288	Ascend
288	Descend
288	Descend
290	Ascend
290	Ascend
291	Ascend
292	Descend
294	Descend
294	Descend
294	Descend
294	Descend
296	Descend
296	Descend
297	Ascend
297	Ascend
297	Descend
298	Descend
298	Descend
298	Descend
298	Descend
299	Descend
299	Descend
300	Descend
300	Descend
300	Ascend
301	Ascend
301	Ascend
302	Descend
302	Descend

302	Ascend
302	Ascend
303	Ascend
303	Ascend
303	Descend
304	Ascend
304	Ascend
305	Ascend
306	Ascend
306	Ascend
307	Ascend
307	Ascend
307	Descend
308	Descend
308	Descend
309	Ascend
309	Ascend
313	Descend
313	Descend
313	Descend
314	Ascend
314	Ascend
314	Ascend
314	Descend
315	Descend
315	Ascend
315	Ascend
316	Ascend
317	Descend
319	Descend
319	Ascend
320	Ascend
320	Ascend
320	Ascend
321	Ascend
322	Ascend
322	Ascend
323	Descend
323	Descend
323	Descend
324	Descend
324	Descend

324	Descend
325	Descend
325	Descend
325	Descend
326	Ascend
326	Ascend
326	Ascend
327	Ascend
327	Ascend
327	Ascend
328	Ascend
328	Ascend
328	Ascend
329	Ascend
329	Ascend
330	Ascend
330	Ascend
330	Ascend
331	Ascend
331	Ascend
332	Ascend
333	Ascend
333	Ascend
334	Ascend
334	Descend
334	Descend
335	Descend
335	Ascend
335	Ascend
335	Ascend
336	Ascend
336	Ascend
336	Ascend
337	Ascend
337	Descend
337	Descend
337	Descend
338	Descend
338	Descend
339	Descend
339	Descend
339	Descend

340	Descend
340	Descend
341	Descend
341	Descend
342	Descend
342	Descend
343	Descend
343	Ascend
343	Ascend
343	Ascend
344	Ascend
344	Descend
344	Descend
344	Descend
345	Descend
345	Descend
345	Descend
346	Descend
346	Descend
346	Descend
347	Ascend
347	Ascend
347	Ascend
348	Ascend
348	Ascend
348	Ascend
348	Ascend
349	Ascend
349	Ascend
349	Ascend
350	Ascend
350	Ascend
350	Ascend
351	Ascend
351	Ascend
352	Ascend
353	Ascend
353	Ascend
354	Descend
354	Descend
354	Ascend
356	Ascend

357	Descend
357	Descend
358	Descend
358	Descend
359	Descend
365	Ascend
365	Ascend
366	Descend
366	Descend
366	Ascend
367	Ascend
367	Descend
367	Descend
368	Ascend
368	Descend
368	Ascend
370	Descend
371	Descend
373	Ascend
374	Ascend
375	Ascend
376	Ascend
378	Ascend
378	Ascend
379	Ascend
381	Ascend
381	Ascend
381	Ascend
383	Ascend
384	Ascend
385	Ascend
385	Ascend
386	Ascend
387	Ascend
389	Ascend
389	Descend
389	Descend
390	Ascend
390	Ascend
392	Ascend
393	Ascend
394	Descend

394	Descend
395	Descend
395	Descend
396	Descend
397	Descend
397	Descend
400	Descend
401	Descend
402	Descend
402	Ascend
403	Ascend
404	Descend
404	Descend
404	Descend
405	Descend
405	Descend
406	Descend
406	Descend
406	Descend
407	Descend
407	Descend
409	Descend
411	Descend
412	Ascend
413	Ascend
413	Ascend
413	Ascend
414	Ascend
415	Ascend
419	Descend
420	Descend
421	Descend
425	Descend
425	Descend
426	Ascend
426	Ascend
427	Ascend
427	Ascend
428	Ascend
428	Ascend
428	Descend
429	Descend

429	Descend
429	Descend
429	Descend
430	Descend
430	Descend
432	Descend
432	Descend
433	Descend
433	Descend
433	Descend
434	Descend
434	Ascend
434	Ascend
435	Ascend
435	Ascend
436	Ascend
436	Ascend
437	Ascend
439	Descend
440	Descend
440	Descend
440	Descend
440	Descend
441	Descend
441	Descend
441	Descend
442	Descend
442	Descend
442	Descend
442	Descend
443	Descend
444	Descend
446	Descend
447	Descend
448	Descend
449	Descend
450	Ascend
451	Ascend
452	Ascend
453	Ascend
453	Ascend
453	Ascend

454	Ascend
455	Ascend
455	Ascend
455	Ascend
456	Descend
456	Descend
456	Descend
456	Descend
457	Descend
458	Descend
458	Descend
458	Descend
458	Ascend
459	Ascend
459	Ascend
459	Ascend
459	Ascend
460	Ascend
460	Ascend
460	Ascend
461	Ascend
461	Descend
462	Descend
462	Descend
462	Descend
463	Descend
463	Descend
463	Descend
464	Descend
465	Descend
465	Descend
466	Descend
466	Descend
467	Descend
467	Descend
468	Descend
468	Descend
469	Ascend
469	Ascend
469	Descend
470	Descend
470	Descend

470	Descend
470	Descend
471	Descend
471	Descend
471	Descend
472	Descend
473	Descend
473	Descend
473	Descend
475	Descend
476	Ascend
477	Ascend
477	Ascend
477	Ascend
477	Ascend
478	Ascend
478	Ascend
479	Ascend
480	Descend
480	Descend
480	Descend
480	Descend
481	Descend
481	Ascend
482	Ascend
482	Ascend
482	Ascend
483	Ascend
483	Ascend
483	Ascend
486	Ascend
486	Ascend
486	Ascend
486	Ascend
487	Ascend
488	Descend
488	Descend
489	Descend
489	Descend
489	Descend
490	Descend
491	Descend

491	Descend
492	Descend
492	Descend
492	Descend
492	Descend
494	Descend
494	Descend
496	Descend
497	Descend
500	Descend
500	Descend
500	Descend
501	Descend
502	Descend
502	Descend
504	Descend
504	Descend
504	Descend
507	Descend
507	Descend
507	Descend
508	Descend
508	Descend
508	Descend
508	Descend
508	Descend
510	Ascend
510	Ascend
510	Ascend
510	Ascend
510	Ascend
511	Ascend
511	Ascend
511	Ascend
511	Ascend
511	Ascend
512	Ascend
512	Ascend
512	Ascend
513	Ascend
513	Ascend
515	Ascend

515	Ascend
515	Ascend
518	Descend
522	Ascend
522	Ascend
522	Descend
522	Descend
526	Ascend
526	Ascend
527	Descend
528	Descend
528	Descend
528	Descend
529	Descend
530	Ascend
530	Ascend
532	Ascend
532	Ascend
533	Ascend
535	Ascend
536	Ascend
536	Ascend
538	Ascend
547	Descend
550	Descend
552	Descend
554	Descend
555	Ascend
556	Ascend
557	Ascend
557	Ascend
558	Ascend
558	Ascend
558	Ascend
558	Ascend
559	Ascend
559	Ascend
559	Ascend
562	Ascend
562	Ascend
564	Ascend
564	Ascend

566	Descend
566	Descend
568	Descend
569	Descend
569	Ascend
569	Ascend
572	Ascend
572	Ascend
573	Ascend
573	Ascend
575	Ascend
576	Ascend
580	Descend
583	Descend
586	Ascend
587	Ascend
587	Ascend
589	Descend
591	Ascend
591	Ascend
593	Ascend
595	Ascend
595	Ascend
595	Ascend
597	Ascend
601	Descend
601	Descend
602	Descend
604	Descend
605	Descend
610	Descend
610	Descend
610	Descend
612	Ascend
612	Ascend
615	Ascend
615	Ascend
618	Ascend
618	Ascend
620	Ascend
620	Ascend
622	Ascend

622	Ascend
626	Ascend
626	Ascend
631	Descend
632	Descend
632	Ascend
632	Ascend
632	Ascend
634	Ascend
635	Ascend
635	Ascend
635	Ascend
637	Ascend
637	Ascend
639	Descend
639	Descend
639	Descend
639	Descend
639	Descend
640	Descend
640	Descend
640	Descend
641	Descend
641	Descend
641	Descend
642	Descend
642	Descend
642	Descend
645	Ascend
645	Descend
645	Descend
646	Descend
646	Descend
647	Ascend
647	Ascend
648	Descend
648	Descend
650	Ascend
650	Ascend
652	Descend
657	Descend
657	Ascend

661	Ascend
661	Ascend
661	Ascend
662	Ascend
663	Descend
664	Descend
664	Descend
664	Descend
665	Descend
665	Descend
668	Descend
668	Descend
670	Descend
670	Descend
670	Descend
671	Descend
671	Descend
671	Descend
672	Descend
672	Ascend
672	Ascend
673	Ascend
673	Ascend
674	Ascend
674	Ascend
675	Ascend
675	Ascend
676	Ascend
676	Ascend
677	Ascend
677	Ascend
677	Ascend
678	Ascend
678	Ascend
680	Ascend
680	Descend
681	Descend
682	Descend
685	Descend
685	Descend
686	Descend
686	Descend

687 Descend
688 Ascend
688 Ascend
690 Descend
692 Descend
692 Descend
695 Descend
700 Ascend
700 Ascend
700 Ascend
702 Ascend
702 Ascend
702 Descend
703 Descend
704 Ascend
706 Ascend
706 Ascend
706 Ascend
707 Ascend
707 Ascend
707 Ascend
708 Ascend
709 Descend
709 Descend
711 Ascend
711 Ascend
713 Descend
713 Descend
714 Descend
714 Descend
715 Descend
716 Descend
716 Descend
716 Descend
717 Descend
718 Descend
719 Descend
721 Descend
721 Ascend
722 Ascend
722 Ascend
722 Ascend

723	Ascend
724	Ascend
725	Ascend
725	Ascend
725	Ascend
726	Ascend
726	Descend
726	Descend
727	Descend
727	Descend
727	Descend
728	Descend
729	Descend
730	Ascend
730	Ascend
731	Ascend
731	Ascend
732	Ascend
732	Ascend
733	Ascend
734	Ascend
734	Descend
735	Descend
735	Descend
737	Ascend
738	Ascend
738	Ascend
738	Ascend
739	Ascend
739	Ascend
740	Descend
741	Ascend
741	Descend
741	Descend
741	Descend
742	Descend
744	Descend
744	Descend
746	Ascend
746	Ascend
748	Ascend
748	Ascend

749	Descend
749	Descend
750	Ascend
750	Ascend
753	Descend
753	Descend
753	Ascend
754	Ascend
754	Ascend
754	Ascend
755	Ascend
755	Ascend
757	Descend
757	Descend
758	Descend
758	Descend
760	Descend
760	Descend
761	Ascend
761	Ascend
764	Ascend
764	Ascend
765	Ascend
765	Ascend
766	Descend
766	Descend
768	Ascend
768	Ascend
769	Descend
769	Descend
771	Descend
772	Descend
772	Descend
773	Descend
776	Descend
776	Ascend
776	Ascend
777	Ascend
777	Ascend
777	Ascend
777	Ascend
778	Ascend

778	Ascend
778	Ascend
779	Ascend
780	Descend
780	Descend
781	Ascend
781	Ascend
782	Descend
782	Descend
783	Descend
783	Descend
784	Ascend
784	Ascend
786	Ascend
786	Ascend
789	Ascend
789	Ascend
789	Ascend
790	Ascend
790	Ascend
791	Descend
791	Descend
793	Descend
793	Descend
794	Ascend
794	Ascend
794	Ascend
794	Ascend
795	Ascend
795	Ascend
795	Ascend
796	Ascend
796	Ascend
796	Ascend
797	Ascend
797	Ascend
799	Ascend
799	Ascend
800	Descend
800	Descend
801	Descend
801	Descend

802	Descend
802	Descend
804	Descend
804	Descend
805	Descend
805	Descend
807	Ascend
807	Ascend
809	Ascend
809	Ascend
811	Ascend
811	Ascend
811	Ascend
812	Ascend
812	Ascend
812	Ascend
812	Ascend
812	Ascend
813	Ascend
813	Ascend
813	Ascend
815	Ascend
816	Ascend
817	Descend
817	Descend
818	Descend
818	Descend
820	Ascend
820	Ascend
820	Ascend
821	Ascend
821	Ascend
822	Ascend
823	Descend
825	Descend
825	Descend
826	Descend
826	Descend
826	Descend
827	Ascend
828	Descend
828	Descend

829	Ascend
830	Ascend
830	Descend
831	Descend
831	Descend
832	Descend
832	Ascend
833	Ascend
833	Ascend
833	Ascend
834	Ascend
834	Ascend
834	Ascend
835	Ascend
836	Descend
836	Descend
837	Descend
837	Descend
838	Descend
838	Descend
838	Descend
839	Descend
840	Descend
842	Descend
844	Descend
845	Ascend
845	Ascend
846	Ascend
846	Ascend
846	Ascend
846	Ascend
847	Ascend
847	Ascend
847	Ascend
848	Ascend
848	Ascend
848	Ascend
850	Ascend
850	Ascend
850	Ascend
851	Ascend
851	Ascend

852	Ascend
852	Ascend
852	Ascend
852	Ascend
853	Ascend
853	Ascend
855	Descend
865	Ascend
865	Ascend
865	Descend
865	Descend
867	Ascend
867	Ascend
867	Ascend
867	Ascend
868	Ascend
868	Ascend
868	Ascend
868	Ascend
868	Ascend
869	Ascend
869	Ascend
870	Ascend
871	Ascend
871	Ascend
872	Ascend
872	Ascend
874	Descend
876	Ascend
876	Ascend
876	Ascend
876	Descend
877	Descend
877	Descend
877	Descend
878	Descend
879	Descend
879	Descend
880	Ascend
880	Ascend
881	Ascend
881	Ascend

883	Descend
883	Descend
883	Descend
886	Ascend
886	Ascend
886	Ascend
887	Ascend
887	Ascend
887	Ascend
888	Ascend
888	Ascend
888	Ascend
888	Ascend
888	Ascend
889	Ascend
889	Ascend
890	Ascend
890	Ascend
891	Ascend
891	Ascend
891	Ascend
891	Descend
892	Descend
892	Descend
892	Descend
892	Descend
893	Descend
893	Descend
894	Descend
895	Descend
895	Descend
897	Ascend
897	Ascend
898	Ascend
898	Ascend
899	Ascend
899	Ascend
899	Ascend
900	Ascend
900	Ascend
8	AntIn
8	AntIn

8	AntOut
8	AntOut
9	AntOut
10	AntOut
11	AntIn
11	AntIn
13	AntIn
13	AntOut
14	AntOut
14	AntOut
15	AntIn
15	AntOut
15	AntOut
16	AntIn
17	AntIn
17	AntIn
18	AntOut
19	AntIn
19	AntOut
20	AntOut
20	AntOut
21	AntOut
22	AntIn
22	AntOut
23	AntIn
23	AntOut
24	AntIn
24	AntIn
25	AntIn
25	AntIn
25	AntIn
26	AntOut
26	AntOut
26	AntOut
26	AntOut
26	AntOut
27	AntOut
27	AntOut
28	AntOut
28	AntOut
29	AntIn
29	AntIn

29	AntIn
30	AntOut
30	AntOut
30	AntOut
31	AntIn
31	AntOut
31	AntOut
32	AntOut
33	AntIn
33	AntOut
34	AntIn
34	AntOut
34	AntOut
35	AntIn
35	AntOut
36	AntIn
36	AntIn
36	AntIn
37	AntIn
37	AntIn
37	AntOut
37	AntOut
38	AntIn
38	AntOut
38	AntOut
39	AntOut
39	AntOut
39	AntOut
40	AntIn
40	AntIn
40	AntOut
41	AntIn
41	AntIn
41	AntOut
41	AntOut
42	AntOut
42	AntOut
42	AntOut
42	AntOut
43	AntIn
43	AntIn
44	AntIn

44 AntIn
45 AntIn
45 AntOut
46 AntIn
46 AntOut
48 AntOut
49 AntOut
50 AntOut
51 AntIn
51 AntIn
51 AntOut
51 AntOut
52 AntOut
53 AntOut
53 AntOut
53 AntOut
53 AntOut
54 AntIn
54 AntIn
54 AntOut
54 AntOut
54 AntOut
55 AntIn
55 AntIn
55 AntOut
55 AntOut
56 AntIn
56 AntIn
56 AntIn
56 AntIn
57 AntOut
57 AntOut
57 AntOut
57 AntOut
57 AntOut
57 AntOut
57 AntOut
57 AntOut
58 AntOut
58 AntOut
58 AntOut
59 AntOut

60 AntIn
60 AntIn
60 AntOut
60 AntOut
60 AntOut
61 AntIn
61 AntIn
61 AntOut
62 AntIn
62 AntIn
62 AntOut
62 AntOut
63 AntIn
64 AntIn
64 AntIn
64 AntOut
65 AntIn
65 AntIn
65 AntOut
66 AntOut
67 AntIn
67 AntIn
67 AntOut
67 AntOut
69 AntIn
69 AntOut
69 AntOut
69 AntOut
70 AntIn
70 AntIn
70 AntIn
71 AntIn
71 AntIn
71 AntIn
72 AntIn
72 AntIn
73 AntIn
73 AntIn
73 AntIn
73 AntOut
74 AntIn
74 AntIn

74	AntOut
75	AntIn
75	AntOut
76	AntIn
77	AntIn
77	AntIn
77	AntOut
77	AntOut
78	AntOut
79	AntOut
79	AntOut
80	AntIn
80	AntIn
80	AntOut
81	AntOut
81	AntOut
82	AntIn
82	AntIn
82	AntOut
82	AntOut
83	AntOut
83	AntOut
83	AntOut
84	AntOut
84	AntOut
84	AntOut
85	AntOut
85	AntOut
86	AntIn
86	AntIn
86	AntIn
86	AntIn
87	AntIn
87	AntIn
88	AntIn
89	AntOut
89	AntOut
90	AntIn
90	AntIn
91	AntIn
91	AntIn
92	AntIn

93	AntOut
94	AntIn
94	AntOut
95	AntIn
95	AntIn
95	AntIn
96	AntIn
96	AntOut
96	AntOut
97	AntIn
97	AntIn
97	AntIn
97	AntIn
97	AntIn
97	AntOut
98	AntIn
98	AntIn
98	AntOut
99	AntIn
99	AntIn
99	AntIn
100	AntIn
100	AntIn
100	AntIn
101	AntOut
101	AntOut
102	AntIn
102	AntOut
102	AntOut
102	AntOut
103	AntOut
103	AntOut
104	AntIn
104	AntOut
104	AntOut
104	AntOut
104	AntOut
105	AntOut
107	AntIn
107	AntIn
107	AntIn
107	AntIn

107	AntIn
107	AntIn
107	AntOut
108	AntOut
109	AntIn
110	AntOut
110	AntOut
110	AntOut
111	AntIn
112	AntIn
112	AntOut
113	AntIn
113	AntIn
114	AntIn
115	AntIn
115	AntOut
116	AntIn
117	AntIn
117	AntOut
118	AntOut
118	AntOut
119	AntIn
120	AntIn
120	AntIn
120	AntOut
121	AntIn
121	AntIn
121	AntIn
122	AntIn
123	AntIn
123	AntIn
123	AntOut
124	AntOut
125	AntOut
125	AntOut
126	AntIn
126	AntOut
127	AntIn
127	AntOut
127	AntOut
128	AntIn
128	AntOut

128	AntOut
129	AntIn
129	AntIn
130	AntIn
130	AntIn
130	AntOut
131	AntIn
132	AntIn
132	AntOut
133	AntIn
133	AntIn
134	AntIn
135	AntOut
136	AntOut
136	AntOut
136	AntOut
136	AntOut
136	AntOut
136	AntOut
136	AntOut
137	AntIn
137	AntOut
137	AntOut
138	AntIn
138	AntOut
138	AntOut
139	AntOut
139	AntOut
140	AntOut
141	AntOut
141	AntOut
141	AntOut
141	AntOut
142	AntIn
142	AntIn
142	AntIn
143	AntIn
143	AntOut
144	AntIn
144	AntOut
144	AntOut
145	AntIn

145	AntIn
145	AntIn
145	AntIn
146	AntIn
148	AntIn
148	AntIn
148	AntIn
148	AntIn
148	AntOut
148	AntOut
149	AntIn
150	AntIn
150	AntOut
151	AntIn
151	AntOut
151	AntOut
151	AntOut
151	AntOut
152	AntIn
152	AntOut
152	AntOut
153	AntIn
153	AntOut
153	AntOut
153	AntOut
153	AntOut
154	AntIn
154	AntOut
154	AntOut
155	AntOut
156	AntOut
157	AntIn
157	AntIn
157	AntIn
157	AntOut
157	AntOut
158	AntOut
159	AntIn
160	AntIn
160	AntOut
160	AntOut
161	AntIn

161	AntOut
161	AntOut
161	AntOut
162	AntIn
162	AntOut
163	AntIn
163	AntIn
163	AntOut
165	AntOut
165	AntOut
166	AntIn
166	AntOut
167	AntIn
168	AntIn
168	AntIn
168	AntIn
169	AntIn
169	AntIn
169	AntIn
171	AntIn
171	AntIn
171	AntIn
172	AntIn
172	AntIn
172	AntIn
172	AntIn
173	AntIn
173	AntIn
174	AntIn
174	AntOut
176	AntIn
176	AntIn
177	AntOut
177	AntOut
177	AntOut
178	AntIn
178	AntIn
178	AntOut
179	AntIn
179	AntOut
180	AntIn
181	AntIn

181	AntOut
182	AntIn
182	AntIn
182	AntIn
183	AntOut
183	AntOut
184	AntIn
184	AntOut
184	AntOut
184	AntOut
184	AntOut
185	AntIn
185	AntOut
185	AntOut
185	AntOut
186	AntIn
186	AntIn
186	AntOut
187	AntIn
188	AntIn
188	AntIn
188	AntIn
188	AntIn
189	AntIn
189	AntIn
189	AntIn
190	AntIn
192	AntIn
192	AntIn
192	AntOut
192	AntOut
193	AntOut
193	AntOut
194	AntIn
194	AntIn
194	AntIn
195	AntIn
195	AntIn
195	AntOut
196	AntIn
198	AntIn
198	AntIn

198	AntIn
198	AntIn
198	AntOut
199	AntIn
200	AntIn
201	AntIn
201	AntOut
202	AntIn
202	AntOut
203	AntIn
203	AntIn
203	AntOut
203	AntOut
204	AntIn
205	AntIn
205	AntIn
206	AntIn
206	AntIn
206	AntOut
208	AntOut
208	AntOut
208	AntOut
208	AntOut
209	AntIn
209	AntOut
209	AntOut
210	AntIn
210	AntIn
210	AntIn
211	AntIn
211	AntIn
212	AntIn
212	AntIn
213	AntIn
213	AntIn
214	AntOut
215	AntIn
215	AntOut
215	AntOut
216	AntIn
216	AntOut
216	AntOut

217	AntIn
218	AntIn
218	AntIn
218	AntIn
219	AntIn
219	AntIn
219	AntOut
220	AntIn
221	AntIn
222	AntOut
222	AntOut
223	AntIn
223	AntOut
223	AntOut
223	AntOut
225	AntOut
225	AntOut
225	AntOut
226	AntOut
226	AntOut
227	AntIn
227	AntIn
227	AntIn
228	AntIn
228	AntOut
228	AntOut
229	AntIn
229	AntOut
229	AntOut
230	AntIn
230	AntIn
230	AntOut
230	AntOut
231	AntIn
231	AntIn
232	AntIn
233	AntIn
233	AntIn
233	AntIn
234	AntOut
234	AntOut
235	AntIn

235 AntOut
236 AntIn
236 AntOut
237 AntOut
237 AntOut
238 AntOut
238 AntOut
239 AntIn
239 AntOut
240 AntIn
241 AntOut
242 AntIn
243 AntIn
243 AntOut
244 AntIn
244 AntOut
244 AntOut
246 AntIn
246 AntOut
247 AntOut
248 AntOut
248 AntOut
249 AntIn
249 AntOut
250 AntIn
250 AntOut
251 AntIn
252 AntIn
253 AntIn
253 AntIn
253 AntOut
253 AntOut
253 AntOut
255 AntIn
255 AntIn
255 AntOut
256 AntIn
256 AntOut
257 AntIn
257 AntOut
258 AntIn
258 AntOut

259 AntIn
259 AntOut
260 AntOut
260 AntOut
260 AntOut
260 AntOut
260 AntOut
261 AntOut
262 AntIn
262 AntOut
262 AntOut
263 AntIn
263 AntIn
263 AntOut
264 AntOut
264 AntOut
265 AntIn
266 AntIn
266 AntOut
268 AntIn
268 AntIn
268 AntOut
270 AntIn
270 AntOut
270 AntOut
271 AntIn
271 AntIn
271 AntOut
271 AntOut
272 AntIn
272 AntIn
273 AntIn
273 AntOut
274 AntIn
274 AntIn
274 AntOut
275 AntIn
275 AntOut
275 AntOut
276 AntOut
276 AntOut
277 AntIn

278	AntIn
278	AntOut
278	AntOut
279	AntOut
279	AntOut
280	AntIn
281	AntIn
281	AntIn
281	AntIn
282	AntIn
282	AntIn
282	AntIn
282	AntIn
283	AntIn
283	AntIn
285	AntIn
286	AntOut
286	AntOut
286	AntOut
287	AntIn
287	AntIn
287	AntOut
287	AntOut
288	AntIn
288	AntIn
289	AntIn
289	AntIn
289	AntOut
289	AntOut
290	AntIn
291	AntIn
291	AntOut
291	AntOut
292	AntOut
293	AntIn
293	AntIn
294	AntOut
294	AntOut
295	AntOut
295	AntOut
297	AntOut
297	AntOut

297 AntOut
298 AntIn
299 AntIn
299 AntOut
300 AntIn
300 AntIn
300 AntOut
301 AntIn
301 AntIn
302 AntIn
302 AntOut
303 AntOut
304 AntIn
305 AntIn
305 AntOut
306 AntIn
306 AntOut
306 AntOut
306 AntOut
307 AntIn
307 AntOut
307 AntOut
308 AntIn
308 AntIn
309 AntIn
309 AntOut
310 AntIn
310 AntIn
310 AntIn
310 AntIn
311 AntOut
312 AntIn
312 AntOut
313 AntIn
313 AntOut
314 AntIn
314 AntIn
314 AntOut
314 AntOut
314 AntOut
314 AntOut
315 AntIn

315	AntOut
316	AntOut
316	AntOut
318	AntOut
318	AntOut
318	AntOut
318	AntOut
318	AntOut
319	AntIn
319	AntIn
319	AntIn
320	AntIn
320	AntOut
321	AntIn
321	AntIn
321	AntIn
322	AntIn
322	AntIn
322	AntOut
323	AntIn
323	AntOut
323	AntOut
324	AntIn
325	AntIn
325	AntIn
325	AntOut
325	AntOut
326	AntOut
327	AntIn
327	AntIn
327	AntOut
327	AntOut
328	AntIn
328	AntOut
328	AntOut
329	AntIn
330	AntIn
330	AntIn
331	AntIn
331	AntIn
331	AntIn
331	AntIn

332 AntIn
333 AntOut
333 AntOut
333 AntOut
333 AntOut
334 AntOut
334 AntOut
335 AntIn
335 AntOut
336 AntIn
337 AntOut
338 AntIn
339 AntIn
339 AntIn
340 AntOut
340 AntOut
340 AntOut
340 AntOut
341 AntIn
341 AntOut
342 AntIn
342 AntIn
342 AntOut
342 AntOut
343 AntIn
343 AntOut
344 AntIn
344 AntIn
344 AntIn
345 AntIn
345 AntIn
345 AntIn
346 AntIn
346 AntIn
346 AntOut
347 AntIn
347 AntOut
348 AntIn
348 AntOut
348 AntOut
349 AntIn
349 AntIn

349 AntIn
350 AntIn
350 AntIn
350 AntIn
351 AntIn
351 AntOut
351 AntOut
352 AntOut
352 AntOut
353 AntIn
353 AntIn
353 AntOut
353 AntOut
353 AntOut
354 AntIn
354 AntOut
354 AntOut
354 AntOut
354 AntOut
356 AntOut
357 AntIn
357 AntOut
357 AntOut
357 AntOut
358 AntIn
359 AntIn
359 AntOut
360 AntIn
360 AntOut
361 AntIn
361 AntIn
361 AntIn
364 AntOut
364 AntOut
364 AntOut
365 AntOut
365 AntOut
365 AntOut
366 AntIn
366 AntIn
366 AntIn
366 AntOut

367 AntIn
367 AntOut
367 AntOut
368 AntIn
369 AntIn
369 AntIn
369 AntIn
369 AntOut
371 AntIn
371 AntIn
371 AntOut
371 AntOut
372 AntIn
372 AntOut
372 AntOut
372 AntOut
373 AntIn
373 AntIn
374 AntIn
374 AntOut
375 AntIn
375 AntIn
376 AntIn
376 AntIn
377 AntIn
378 AntIn
379 AntIn
379 AntIn
379 AntIn
380 AntIn
380 AntIn
380 AntIn
380 AntOut
381 AntIn
381 AntIn
382 AntIn
382 AntOut
383 AntIn
384 AntIn
384 AntOut
385 AntIn
385 AntIn

385	AntOut
386	AntIn
386	AntIn
386	AntIn
387	AntIn
387	AntIn
387	AntOut
387	AntOut
387	AntOut
388	AntIn
388	AntIn
388	AntIn
388	AntOut
389	AntOut
389	AntOut
389	AntOut
390	AntIn
390	AntOut
390	AntOut
391	AntIn
391	AntIn
391	AntOut
392	AntIn
393	AntOut
393	AntOut
394	AntIn
394	AntIn
394	AntOut
395	AntOut
395	AntOut
396	AntIn
397	AntIn
397	AntOut
398	AntOut
399	AntOut
399	AntOut
400	AntOut
400	AntOut
400	AntOut
401	AntOut
401	AntOut
402	AntOut

403	AntIn
403	AntIn
403	AntIn
403	AntOut
404	AntIn
406	AntIn
406	AntIn
406	AntIn
406	AntOut
406	AntOut
408	AntIn
408	AntOut
409	AntIn
409	AntOut
409	AntOut
410	AntIn
411	AntIn
412	AntIn
413	AntIn
414	AntOut
414	AntOut
415	AntIn
415	AntIn
416	AntIn
416	AntIn
417	AntIn
417	AntIn
418	AntIn
418	AntOut
419	AntIn
419	AntOut
420	AntIn
420	AntIn
420	AntOut
420	AntOut
421	AntIn
421	AntOut
421	AntOut
421	AntOut
422	AntIn
422	AntIn
423	AntIn

423	AntOut
423	AntOut
424	AntOut
424	AntOut
425	AntIn
425	AntIn
427	AntIn
427	AntIn
428	AntIn
428	AntIn
429	AntIn
431	AntIn
431	AntIn
432	AntIn
432	AntOut
433	AntOut
434	AntOut
435	AntIn
435	AntIn
435	AntIn
435	AntIn
436	AntIn
437	AntIn
437	AntIn
438	AntIn
439	AntIn
439	AntIn
440	AntIn
440	AntIn
441	AntOut
441	AntOut
442	AntIn
442	AntOut
442	AntOut
442	AntOut
443	AntOut
444	AntIn
444	AntIn
444	AntOut
445	AntIn
447	AntIn
447	AntOut

447 AntOut
448 AntIn
448 AntOut
448 AntOut
449 AntIn
450 AntIn
450 AntOut
451 AntOut
452 AntOut
452 AntOut
453 AntIn
453 AntOut
453 AntOut
453 AntOut
454 AntOut
454 AntOut
456 AntIn
457 AntIn
457 AntOut
458 AntOut
459 AntOut
461 AntIn
461 AntIn
461 AntIn
461 AntOut
462 AntIn
462 AntIn
462 AntOut
463 AntIn
463 AntIn
463 AntOut
464 AntIn
464 AntIn
465 AntIn
465 AntOut
466 AntIn
466 AntOut
466 AntOut
467 AntIn
467 AntOut
468 AntIn
469 AntIn

469	AntIn
469	AntOut
470	AntIn
470	AntOut
470	AntOut
471	AntIn
472	AntIn
473	AntIn
473	AntIn
474	AntIn
475	AntIn
476	AntIn
477	AntIn
477	AntOut
477	AntOut
477	AntOut
478	AntIn
478	AntIn
479	AntIn
479	AntIn
479	AntOut
479	AntOut
480	AntIn
480	AntIn
480	AntOut
481	AntIn
481	AntIn
481	AntIn
482	AntIn
484	AntIn
484	AntIn
484	AntOut
485	AntIn
486	AntIn
487	AntIn
489	AntIn
489	AntIn
489	AntOut
491	AntIn
492	AntIn
492	AntIn
492	AntIn

493	AntIn
494	AntIn
494	AntIn
495	AntIn
495	AntOut
496	AntIn
497	AntIn
498	AntOut
499	AntIn
500	AntIn
501	AntIn
502	AntIn
503	AntIn
503	AntOut
504	AntIn
504	AntOut
506	AntIn
506	AntIn
508	AntIn
509	AntOut
510	AntOut
511	AntIn
512	AntIn
513	AntIn
513	AntIn
513	AntOut
513	AntOut
513	AntOut
515	AntIn
515	AntOut
515	AntOut
515	AntOut
515	AntOut
516	AntIn
517	AntIn
517	AntOut
518	AntIn
518	AntIn
520	AntIn
520	AntIn
520	AntOut
521	AntIn

521 AntIn
522 AntIn
522 AntOut
523 AntIn
523 AntOut
523 AntOut
524 AntIn
524 AntIn
524 AntOut
524 AntOut
525 AntIn
525 AntOut
525 AntOut
526 AntIn
526 AntIn
527 AntIn
529 AntIn
529 AntOut
530 AntIn
530 AntOut
532 AntIn
532 AntIn
533 AntIn
533 AntIn
533 AntOut
533 AntOut
534 AntIn
534 AntIn
534 AntIn
534 AntOut
535 AntIn
536 AntOut
537 AntIn
537 AntOut
538 AntIn
538 AntIn
538 AntIn
539 AntIn
539 AntIn
540 AntIn
540 AntIn
541 AntIn

542	AntIn
542	AntIn
545	AntOut
546	AntOut
546	AntOut
546	AntOut
547	AntIn
547	AntOut
547	AntOut
548	AntIn
548	AntIn
548	AntIn
548	AntOut
549	AntIn
549	AntOut
550	AntIn
550	AntIn
550	AntIn
551	AntIn
551	AntIn
551	AntOut
552	AntIn
552	AntIn
552	AntIn
552	AntIn
552	AntOut
553	AntIn
553	AntOut
553	AntOut
553	AntOut
554	AntOut
555	AntIn
556	AntIn
556	AntOut
557	AntIn
557	AntIn
557	AntOut
557	AntOut
557	AntOut
558	AntIn
558	AntIn
558	AntIn

559	AntIn
559	AntOut
559	AntOut
560	AntIn
560	AntIn
560	AntIn
560	AntIn
560	AntOut
561	AntOut
562	AntIn
562	AntOut
563	AntOut
563	AntOut
564	AntIn
565	AntIn
566	AntOut
567	AntIn
567	AntIn
568	AntIn
569	AntIn
569	AntIn
569	AntIn
569	AntIn
569	AntIn
569	AntIn
570	AntIn
570	AntIn
571	AntIn
571	AntIn
572	AntOut
573	AntIn
573	AntOut
574	AntOut
574	AntOut
574	AntOut
574	AntOut
575	AntIn
575	AntIn
575	AntOut
576	AntOut
576	AntOut
576	AntOut

577	AntIn
577	AntIn
578	AntIn
579	AntIn
580	AntOut
581	AntIn
581	AntIn
581	AntIn
581	AntOut
582	AntIn
583	AntOut
583	AntOut
584	AntIn
584	AntIn
585	AntIn
585	AntIn
586	AntIn
586	AntIn
587	AntIn
588	AntIn
588	AntIn
589	AntIn
591	AntIn
591	AntIn
592	AntOut
592	AntOut
593	AntOut
593	AntOut
594	AntIn
595	AntIn
596	AntIn
597	AntIn
598	AntIn
598	AntIn
599	AntIn
599	AntIn
599	AntIn
600	AntIn
600	AntIn
601	AntIn
601	AntOut
602	AntIn

602	AntOut
603	AntIn
603	AntOut
604	AntIn
604	AntIn
605	AntIn
605	AntIn
605	AntIn
606	AntIn
607	AntIn
607	AntIn
607	AntOut
607	AntOut
608	AntOut
608	AntOut
608	AntOut
609	AntIn
609	AntIn
609	AntOut
610	AntOut
611	AntIn
611	AntIn
611	AntOut
611	AntOut
612	AntIn
612	AntIn
612	AntIn
612	AntIn
612	AntOut
613	AntIn
613	AntIn
613	AntIn
613	AntOut
614	AntIn
614	AntOut
614	AntOut
615	AntIn
615	AntIn
616	AntIn
617	AntIn
617	AntOut
618	AntIn

618 AntIn
619 AntOut
620 AntIn
621 AntIn
621 AntIn
621 AntOut
623 AntOut
623 AntOut
624 AntIn
624 AntOut
625 AntIn
627 AntIn
627 AntIn
628 AntIn
628 AntIn
629 AntIn
629 AntIn
630 AntIn
630 AntIn
631 AntOut
632 AntIn
633 AntIn
634 AntIn
634 AntOut
635 AntIn
636 AntIn
637 AntIn
638 AntIn
638 AntIn
638 AntOut
638 AntOut
638 AntOut
639 AntIn
639 AntIn
639 AntOut
640 AntIn
640 AntIn
640 AntIn
641 AntOut
641 AntOut
641 AntOut
643 AntOut

643	AntOut
644	AntIn
644	AntIn
644	AntOut
645	AntIn
646	AntIn
646	AntIn
646	AntIn
647	AntIn
647	AntIn
647	AntIn
649	AntIn
649	AntIn
649	AntIn
650	AntIn
650	AntOut
651	AntIn
652	AntOut
653	AntOut
654	AntIn
655	AntIn
656	AntIn
656	AntIn
656	AntIn
656	AntIn
656	AntIn
657	AntIn
657	AntIn
657	AntOut
658	AntIn
658	AntIn
658	AntIn
658	AntOut
659	AntIn
659	AntIn
660	AntIn
660	AntOut
660	AntOut
660	AntOut
661	AntIn
661	AntIn
662	AntIn

662 AntIn
662 AntOut
664 AntIn
665 AntIn
665 AntIn
665 AntIn
666 AntOut
667 AntIn
667 AntIn
667 AntOut
668 AntIn
669 AntIn
669 AntIn
669 AntOut
671 AntIn
671 AntIn
672 AntOut
673 AntIn
673 AntOut
674 AntIn
674 AntIn
675 AntIn
675 AntIn
675 AntIn
676 AntOut
677 AntIn
677 AntIn
677 AntOut
678 AntIn
679 AntIn
679 AntIn
679 AntOut
679 AntOut
680 AntOut
680 AntOut
681 AntOut
681 AntOut
681 AntOut
682 AntOut
683 AntIn
683 AntIn
683 AntIn

683	AntIn
684	AntIn
684	AntOut
684	AntOut
684	AntOut
685	AntIn
685	AntOut
686	AntIn
686	AntIn
686	AntIn
686	AntIn
687	AntIn
687	AntIn
687	AntOut
688	AntIn
689	AntIn
689	AntOut
690	AntIn
690	AntIn
690	AntOut
691	AntIn
692	AntIn
693	AntIn
694	AntIn
694	AntOut
696	AntIn
697	AntOut
700	AntOut
701	AntIn
702	AntIn
703	AntIn
703	AntOut
706	AntIn
707	AntIn
708	AntIn
709	AntIn
709	AntIn
710	AntIn
711	AntIn
711	AntOut
711	AntOut
712	AntIn

712	AntIn
712	AntIn
712	AntOut
713	AntOut
714	AntIn
714	AntIn
715	AntIn
716	AntOut
717	AntIn
717	AntIn
717	AntIn
718	AntOut
719	AntIn
720	AntIn
720	AntIn
721	AntIn
721	AntIn
721	AntOut
722	AntOut
722	AntOut
722	AntOut
723	AntOut
724	AntIn
725	AntIn
726	AntIn
726	AntOut
727	AntIn
727	AntOut
727	AntOut
728	AntOut
729	AntIn
729	AntOut
730	AntIn
730	AntOut
731	AntIn
731	AntIn
731	AntOut
732	AntIn
732	AntIn
733	AntIn
733	AntOut
733	AntOut

734	AntIn
734	AntIn
734	AntOut
735	AntIn
736	AntIn
737	AntOut
737	AntOut
738	AntIn
738	AntIn
738	AntOut
739	AntIn
739	AntIn
740	AntIn
740	AntIn
741	AntIn
741	AntIn
743	AntIn
743	AntIn
744	AntIn
744	AntIn
744	AntOut
744	AntOut
745	AntIn
745	AntOut
746	AntOut
747	AntIn
748	AntOut
748	AntOut
749	AntIn
749	AntOut
750	AntIn
751	AntIn
751	AntOut
752	AntIn
752	AntIn
752	AntOut
753	AntIn
753	AntOut
753	AntOut
753	AntOut
754	AntIn
755	AntIn

756 AntIn
756 AntIn
756 AntOut
757 AntIn
757 AntIn
757 AntIn
757 AntIn
757 AntIn
758 AntIn
758 AntOut
758 AntOut
759 AntIn
759 AntIn
759 AntOut
760 AntOut
761 AntOut
762 AntIn
762 AntIn
762 AntIn
764 AntIn
765 AntOut
765 AntOut
766 AntIn
766 AntIn
766 AntIn
769 AntOut
769 AntOut
770 AntIn
770 AntIn
771 AntIn
771 AntIn
772 AntIn
773 AntIn
773 AntIn
774 AntIn
775 AntIn
775 AntIn
775 AntIn
775 AntIn
776 AntOut
779 AntOut
783 AntOut

784 AntIn
784 AntIn
784 AntOut
785 AntIn
786 AntIn
786 AntIn
786 AntIn
786 AntOut
787 AntOut
788 AntIn
789 AntIn
790 AntOut
791 AntIn
791 AntOut
793 AntIn
793 AntOut
793 AntOut
794 AntIn
795 AntIn
795 AntIn
795 AntOut
796 AntIn
796 AntOut
796 AntOut
797 AntIn
797 AntIn
797 AntIn
798 AntIn
798 AntIn
799 AntIn
800 AntOut
800 AntOut
800 AntOut
801 AntOut
802 AntIn
803 AntIn
803 AntIn
803 AntIn
803 AntIn
803 AntOut
804 AntOut
806 AntIn

806	AntIn
807	AntIn
807	AntIn
807	AntIn
807	AntOut
808	AntIn
808	AntIn
808	AntOut
808	AntOut
809	AntIn
810	AntIn
811	AntIn
812	AntIn
812	AntIn
813	AntOut
813	AntOut
814	AntIn
814	AntIn
814	AntOut
815	AntIn
815	AntOut
817	AntIn
817	AntIn
817	AntIn
818	AntIn
819	AntIn
819	AntIn
819	AntOut
820	AntIn
821	AntIn
821	AntIn
822	AntIn
822	AntOut
823	AntIn
823	AntIn
825	AntOut
825	AntOut
825	AntOut
825	AntOut
826	AntOut
826	AntOut
826	AntOut

827	AntIn
828	AntIn
828	AntIn
828	AntIn
828	AntOut
829	AntIn
830	AntOut
831	AntIn
831	AntIn
831	AntOut
832	AntOut
832	AntOut
833	AntIn
833	AntIn
833	AntOut
834	AntOut
834	AntOut
834	AntOut
834	AntOut
835	AntIn
835	AntOut
836	AntIn
837	AntOut
838	AntOut
839	AntOut
839	AntOut
840	AntOut
840	AntOut
840	AntOut
841	AntIn
841	AntIn
841	AntOut
842	AntIn
843	AntIn
843	AntIn
844	AntIn
844	AntOut
844	AntOut
845	AntIn
845	AntIn
845	AntIn
846	AntIn

847	AntIn
847	AntIn
847	AntIn
847	AntIn
848	AntIn
848	AntIn
848	AntIn
848	AntOut
849	AntOut
849	AntOut
851	AntIn
851	AntIn
851	AntOut
852	AntIn
853	AntIn
853	AntIn
853	AntIn
853	AntOut
853	AntOut
854	AntIn
854	AntIn
854	AntIn
854	AntIn
855	AntIn
855	AntIn
856	AntIn
857	AntIn
857	AntIn
857	AntIn
857	AntOut
858	AntIn
858	AntOut
859	AntIn
859	AntOut
860	AntIn
860	AntOut
860	AntOut
861	AntOut
861	AntOut
862	AntIn
862	AntIn
863	AntIn

863	AntIn
863	AntOut
863	AntOut
864	AntIn
864	AntOut
865	AntIn
865	AntOut
866	AntIn
866	AntIn
866	AntOut
866	AntOut
867	AntIn
867	AntOut
868	AntIn
868	AntIn
868	AntOut
868	AntOut
869	AntIn
869	AntIn
870	AntOut
870	AntOut
871	AntOut
871	AntOut
871	AntOut
872	AntIn
872	AntIn
872	AntOut
873	AntIn
873	AntOut
874	AntIn
874	AntIn
875	AntIn
875	AntIn
876	AntOut
876	AntOut
876	AntOut
876	AntOut
876	AntOut
877	AntOut
877	AntOut
878	AntIn
878	AntOut

879 AntIn
879 AntIn
879 AntOut
880 AntIn
880 AntOut
881 AntIn
882 AntIn
882 AntIn
882 AntIn
882 AntOut
882 AntOut
883 AntIn
883 AntIn
883 AntIn
883 AntIn
883 AntOut
884 AntIn
884 AntIn
884 AntIn
885 AntIn
885 AntIn
886 AntOut
887 AntIn
887 AntIn
888 AntIn
888 AntIn
888 AntIn
888 AntOut
889 AntIn
889 AntOut
890 AntIn
890 AntIn
891 AntOut
891 AntOut
892 AntIn
892 AntOut
892 AntOut
892 AntOut
893 AntOut
894 AntIn
894 AntIn
894 AntIn

895	AntIn
895	AntIn
895	AntIn
896	AntIn
896	AntIn
896	AntOut
897	AntOut
897	AntOut
898	AntIn
898	AntIn
898	AntOut
899	AntIn
899	AntIn
899	AntIn
899	AntOut
900	AntIn
900	AntIn
901	AntIn
901	AntIn
902	AntOut
903	AntIn
