# Supplementary material for: Interactions Increase Forager Availability and Activity in Harvester Ants
Source: PLoS One. 2015 Nov 5;10(11):e0141971. doi: 10.1371/journal.pone.0141971 (PMC4635008; doi:10.1371/journal.pone.0141971)
Supplement: S4 Dataset — After splitting the 2013 videos into JPEG frames, we marked the location in the entrance chamber of each interaction of the ants we tracked. This dataset shows the locations of interactions from two instantaneous images, one at the start of the removal of returning foragers, and the other immediately after forager removals ended 3–5 minutes later. (PDF) [file pone.0141971.s005.pdf]

2013 Heat Maps Data
Researcher
Jovel Queirolo

Colony	Date	X	Y	Frame	Video time (minutes: seconds)
25	8/24/13	433	424	10807	6:00
25	8/24/13	443	464	10807	6:00
25	8/24/13	426	498	10807	6:00
25	8/24/13	399	512	10807	6:00
25	8/24/13	825	284	10807	6:00
25	8/24/13	825	305	10807	6:00
25	8/24/13	631	527	10807	6:00
25	8/24/13	693	572	10807	6:00
25	8/24/13	674	587	10807	6:00
25	8/24/13	707	609	10807	6:00
25	8/24/13	777	575	10807	6:00
25	8/24/13	811	632	10807	6:00
25	8/24/13	934	676	10807	6:00
25	8/24/13	898	719	10807	6:00
25	8/24/13	850	852	10807	6:00
25	8/24/13	1025	828	10807	6:00
25	8/24/13	1006	904	10807	6:00
25	8/24/13	955	946	10807	6:00
25	8/24/13	936	899	10807	6:00
25	8/24/13	876	974	10807	6:00
25	8/24/13	703	568	19796	11:00
25	8/24/13	727	601	19796	11:00
25	8/24/13	963	679	19796	11:00
25	8/24/13	936	859	19796	11:00
25	8/24/13	852	946	19796	11:00
25	8/24/13	963	926	19796	11:00
229	8/24/13	1408	739	10798	6:00
229	8/24/13	1374	804	10798	6:00
229	8/24/13	1383	847	10798	6:00
229	8/24/13	1393	904	10798	6:00
229	8/24/13	1342	945	10798	6:00
229	8/24/13	1280	929	10798	6:00
229	8/24/13	1283	1003	10798	6:00
229	8/24/13	1157	965	10798	6:00
229	8/24/13	1451	563	10798	6:00
229	8/24/13	830	818	10798	6:00

229	8/24/13	1047	714	10798	6:00
229	8/24/13	1051	553	10798	6:00
229	8/24/13	1237	82	10798	6:00
229	8/24/13	1470	794	19786	11:00
229	8/24/13	1374	873	19786	11:00
229	8/24/13	1319	921	19786	11:00
229	8/24/13	1323	839	19786	11:00
229	8/24/13	1275	899	19786	11:00
229	8/24/13	1268	861	19786	11:00
229	8/26/15	1230	606	10793	6:00
229	8/26/15	1141	561	10793	6:00
229	8/26/15	1051	380	10793	6:00
229	8/26/15	1003	392	10793	6:00
229	8/26/15	955	447	10793	6:00
229	8/26/15	1058	630	10793	6:00
229	8/26/15	1003	691	10793	6:00
229	8/26/15	993	625	10793	6:00
229	8/26/15	953	668	10793	6:00
229	8/26/15	888	808	10793	6:00
229	8/26/15	849	818	10793	6:00
229	8/26/15	791	880	10793	6:00
229	8/26/15	470	270	10793	6:00
229	8/26/15	845	613	10793	6:00
229	8/26/15	804	644	10793	6:00
229	8/26/15	767	674	10793	6:00
229	8/26/15	850	734	10793	6:00
229	8/26/15	900	87	10793	6:00
229	8/26/15	842	630	16188	9:00
229	8/26/15	782	678	16188	9:00
229	8/26/15	781	789	16188	9:00
242	8/25/13	981	1010	10787	6:00
242	8/25/13	999	970	10787	6:00
242	8/25/13	1230	804	10787	6:00
242	8/25/13	1273	760	10787	6:00
242	8/25/13	1272	650	10787	6:00
242	8/25/13	1302	613	10787	6:00
242	8/25/13	1347	553	10787	6:00
242	8/25/13	1277	577	10787	6:00
242	8/25/13	962	572	10787	6:00
242	8/25/13	921	395	10787	6:00
242	8/25/13	972	346	10787	6:00
242	8/25/13	1051	183	10787	6:00

242	8/25/13	984	140	10787	6:00
242	8/25/13	922	159	10787	6:00
242	8/25/13	866	185	10787	6:00
242	8/25/13	821	250	10787	6:00
242	8/25/13	847	317	10787	6:00
242	8/25/13	787	308	10787	6:00
242	8/25/13	734	325	10787	6:00
242	8/25/13	767	368	10787	6:00
242	8/25/13	684	258	10787	6:00
242	8/25/13	631	269	10787	6:00
242	8/25/13	647	313	10787	6:00
242	8/25/13	585	370	10787	6:00
242	8/25/13	638	431	10787	6:00
242	8/25/13	726	387	10787	6:00
242	8/25/13	779	337	10787	6:00
242	8/25/13	642	224	10787	6:00
242	8/25/13	616	202	10787	6:00
242	8/25/13	582	421	10787	6:00
242	8/25/13	1071	77	19792	11:00
242	8/25/13	1061	110	19792	11:00
242	8/25/13	1030	53	19792	11:00
242	8/25/13	967	67	19792	11:00
242	8/25/13	931	111	19792	11:00
242	8/25/13	825	315	19792	11:00
242	8/25/13	804	253	19792	11:00
242	8/25/13	1023	135	19792	11:00
367	8/18/13	748	445	10801	6:00
367	8/18/13	1388	587	10801	6:00
367	8/18/13	1386	618	10801	6:00
367	8/18/13	1386	659	10801	6:00
367	8/18/13	1403	592	16184	9:00
367	8/18/13	1404	593	16184	9:00
367	8/20/18	628	438	10798	6:00
367	8/20/18	710	466	10798	6:00
367	8/20/18	897	496	10798	6:00
367	8/20/18	907	325	10798	6:00
367	8/20/18	1066	476	10798	6:00
367	8/20/18	1102	519	10798	6:00
367	8/20/18	1155	462	10798	6:00
367	8/20/18	1136	484	10798	6:00
367	8/20/18	1225	577	10798	6:00
367	8/20/18	1261	721	10798	6:00

367	8/20/18	1258	681	10798	6:00
367	8/20/18	1302	668	10798	6:00
367	8/20/18	1318	640	10798	6:00
367	8/20/18	1328	604	10798	6:00
367	8/20/18	1253	693	19784	11:00
367	8/20/18	1319	611	19784	11:00
367	8/20/18	1323	652	19784	11:00
367	8/20/18	1102	546	19784	11:00
367	8/21/13	765	488	10792	6:00
367	8/21/13	830	474	10792	6:00
367	8/21/13	828	438	10792	6:00
367	8/21/13	862	394	10792	6:00
367	8/21/13	1013	426	10792	6:00
367	8/21/13	1056	353	10792	6:00
367	8/21/13	1135	145	10792	6:00
367	8/21/13	1244	354	10792	6:00
367	8/21/13	1241	407	10792	6:00
367	8/21/13	1396	863	10792	6:00
367	8/21/13	1367	765	10792	6:00
367	8/21/13	1362	685	10792	6:00
367	8/21/13	1306	618	10792	6:00
367	8/21/13	1420	657	10792	6:00
367	8/21/13	1509	673	10792	6:00
367	8/21/13	1508	638	10792	6:00
367	8/21/13	1485	590	10792	6:00
367	8/21/13	1511	567	10792	6:00
367	8/21/13	1338	796	10792	6:00
367	8/21/13	1112	784	10792	6:00
367	8/21/13	1129	265	10792	6:00
367	8/21/13	1246	284	10792	6:00
367	8/21/13	1181	267	10792	6:00
367	8/21/13	987	638	19783	11:00
367	8/21/13	1075	377	19783	11:00
367	8/21/13	1340	490	19783	11:00
367	8/21/13	1468	659	19783	11:00
367	8/21/13	1432	606	19783	11:00
868	8/19/13	1273	402	10795	6:00
868	8/19/13	1213	419	10795	6:00
868	8/19/13	1236	466	10795	6:00
868	8/19/13	1184	469	10795	6:00
868	8/19/13	1143	349	10795	6:00
868	8/19/13	1025	484	10795	6:00

868	8/19/13	1129	142	10795	6:00
868	8/19/13	1097	108	10795	6:00
868	8/19/13	1058	92	10795	6:00
868	8/19/13	640	955	10795	6:00
868	8/19/13	566	1006	10795	6:00
868	8/19/13	542	955	10795	6:00
868	8/19/13	501	904	10795	6:00
868	8/19/13	423	893	10795	6:00
868	8/19/13	344	868	10795	6:00
868	8/19/13	342	786	10795	6:00
868	8/19/13	303	743	10795	6:00
868	8/19/13	405	714	10795	6:00
868	8/19/13	327	697	10795	6:00
868	8/19/13	472	729	10795	6:00
868	8/19/13	561	659	10795	6:00
868	8/19/13	505	664	10795	6:00
868	8/19/13	583	590	10795	6:00
868	8/19/13	428	599	10795	6:00
868	8/19/13	525	536	10795	6:00
868	8/19/13	429	534	10795	6:00
868	8/19/13	323	647	10795	6:00
868	8/19/13	705	791	16185	9:00
868	8/19/13	366	816	16185	9:00
868	8/19/13	306	798	16185	9:00
868	8/19/13	316	693	16185	9:00
868	8/19/13	322	664	16185	9:00
868	8/20/13	1523	401	10798	6:00
868	8/20/13	1460	695	10798	6:00
868	8/20/13	1453	662	10798	6:00
868	8/20/13	1138	187	10798	6:00
868	8/20/13	999	147	10798	6:00
868	8/20/13	1099	342	10798	6:00
868	8/20/13	1114	401	10798	6:00
868	8/20/13	1082	604	10798	6:00
868	8/20/13	922	433	10798	6:00
868	8/20/13	969	337	10798	6:00
868	8/20/13	1302	517	10798	6:00
868	8/20/13	1040	616	10798	6:00
868	8/20/13	838	606	10798	6:00
868	8/20/13	784	568	10798	6:00
868	8/20/13	741	551	10798	6:00
868	8/20/13	737	579	10798	6:00

868	8/20/13	751	625	10798	6:00
868	8/20/13	684	676	10798	6:00
868	8/20/13	623	676	10798	6:00
868	8/20/13	671	712	10798	6:00
868	8/20/13	631	702	10798	6:00
868	8/20/13	1071	555	10798	6:00
868	8/20/13	1254	501	16184	9:00
868	8/20/13	1116	431	16184	9:00
868	8/20/13	1095	483	16184	9:00
868	8/20/13	1143	669	16184	9:00
868	8/20/13	1141	750	16184	9:00
868	8/20/13	1049	760	16184	9:00
868	8/20/13	960	806	16184	9:00
868	8/20/13	945	851	16184	9:00
868	8/20/13	924	907	16184	9:00
868	8/20/13	880	791	16184	9:00
868	8/20/13	917	714	16184	9:00
868	8/20/13	830	656	16184	9:00
868	8/20/13	815	596	16184	9:00
868	8/20/13	768	597	16184	9:00
868	8/20/13	780	700	16184	9:00
868	8/20/13	881	970	16184	9:00
868	8/20/13	623	688	16184	9:00
868	8/20/13	690	755	16184	9:00
868	8/20/13	715	721	16184	9:00
868	8/20/13	625	621	16184	9:00
868	8/20/13	702	893	16184	9:00
868	8/20/13	741	935	16184	9:00
868	8/20/13	803	856	16184	9:00
868	8/21/13	1592	517	10795	6:00
868	8/21/13	1586	426	10795	6:00
868	8/21/13	1509	337	10795	6:00
868	8/21/13	1473	356	10795	6:00
868	8/21/13	1458	560	10795	6:00
868	8/21/13	1369	702	10795	6:00
868	8/21/13	1244	606	10795	6:00
868	8/21/13	1277	313	10795	6:00
868	8/21/13	1112	390	10795	6:00
868	8/21/13	1068	308	10795	6:00
868	8/21/13	1034	301	10795	6:00
868	8/21/13	1039	252	10795	6:00
868	8/21/13	1087	430	10795	6:00

	868	8/21/13	1044	440	10795	6:00
	868	8/21/13	1107	503	10795	6:00
	868	8/21/13	993	567	10795	6:00
	868	8/21/13	953	525	10795	6:00
	868	8/21/13	919	496	10795	6:00
	868	8/21/13	927	768	10795	6:00
	868	8/21/13	871	647	10795	6:00
	868	8/21/13	873	585	10795	6:00
	868	8/21/13	815	570	10795	6:00
	868	8/21/13	856	484	10795	6:00
	868	8/21/13	727	490	10795	6:00
	868	8/21/13	763	666	10795	6:00
	868	8/21/13	743	698	10795	6:00
	868	8/21/13	731	661	10795	6:00
	868	8/21/13	777	782	10795	6:00
	868	8/21/13	737	811	10795	6:00
	868	8/21/13	681	770	10795	6:00
	868	8/21/13	695	671	10795	6:00
	868	8/21/13	672	541	10795	6:00
	868	8/21/13	601	688	10795	6:00
	868	8/21/13	559	676	10795	6:00
	868	8/21/13	845	698	19781	11:00
	868	8/21/13	643	546	19781	11:00
	868	8/21/13	575	649	19781	11:00
N5		8/17/13	994	301	11700	6:30
N5		8/17/13	1003	192	11700	6:30
N5		8/17/13	837	228	11700	6:30
N5		8/17/13	943	496	11700	6:30
N5		8/17/13	902	409	11700	6:30
N5		8/17/13	850	402	11700	6:30
N5		8/17/13	869	433	11700	6:30
N5		8/17/13	748	359	11700	6:30
N5		8/17/13	789	368	11700	6:30
N5		8/17/13	763	401	11700	6:30
N5		8/17/13	811	436	11700	6:30
N5		8/17/13	777	433	11700	6:30
N5		8/17/13	828	452	11700	6:30
N5		8/17/13	736	387	11700	6:30
N5		8/17/13	825	490	11700	6:30
N5		8/17/13	808	507	11700	6:30
N5		8/17/13	744	524	11700	6:30
N5		8/17/13	792	568	11700	6:30

N5	8/17/13	986	596	11700	6:30
N5	8/17/13	628	464	11700	6:30
N5	8/17/13	614	496	11700	6:30
N5	8/17/13	589	567	11700	6:30
N5	8/17/13	551	657	11700	6:30
N5	8/17/13	609	798	11700	6:30
N5	8/17/13	645	695	11700	6:30
N5	8/17/13	743	760	11700	6:30
N5	8/17/13	756	679	11700	6:30
N5	8/17/13	744	705	11700	6:30
N5	8/17/13	707	733	11700	6:30
N5	8/17/13	1013	209	17100	9:30
N5	8/17/13	981	262	17100	9:30
N5	8/17/13	970	156	17100	9:30
N5	8/17/13	883	193	17100	9:30
N5	8/17/13	1008	390	17100	9:30
N5	8/17/13	849	329	17100	9:30
N5	8/17/13	856	371	17100	9:30
N5	8/17/13	832	428	17100	9:30
N5	8/17/13	886	522	17100	9:30
N5	8/17/13	835	483	17100	9:30
N5	8/17/13	761	426	17100	9:30
N5	8/17/13	791	501	17100	9:30
N5	8/17/13	686	460	17100	9:30
N5	8/17/13	724	363	17100	9:30
N5	8/17/13	744	486	17100	9:30
N5	8/17/13	773	493	17100	9:30
N5	8/17/13	794	556	17100	9:30
N5	8/17/13	782	592	17100	9:30
N5	8/17/13	765	645	17100	9:30
N5	8/17/13	765	690	17100	9:30
N5	8/17/13	791	750	17100	9:30
N5	8/17/13	755	745	17100	9:30
N5	8/17/13	611	666	17100	9:30
N5	8/17/13	616	599	17100	9:30
N5	8/17/13	619	585	17100	9:30
N5	8/17/13	655	515	17100	9:30
N5	8/17/13	708	424	17100	9:30
N5	8/17/13	724	772	17100	9:30
N5	8/18/13	972	455	14393	8:00
N5	8/18/13	921	462	14393	8:00
N5	8/18/13	895	426	14393	8:00

N5	8/18/13	815	279	14393	8:00
N5	8/18/13	755	245	14393	8:00
N5	8/18/13	724	270	14393	8:00
N5	8/18/13	782	315	14393	8:00
N5	8/18/13	746	327	14393	8:00
N5	8/18/13	710	310	14393	8:00
N5	8/18/13	642	443	14393	8:00
N5	8/18/13	626	493	14393	8:00
N5	8/18/13	850	594	14393	8:00
N5	8/18/13	936	572	14393	8:00
N5	8/18/13	787	577	14393	8:00
N5	8/18/13	741	570	14393	8:00
N5	8/18/13	650	567	14393	8:00
N5	8/18/13	594	532	14393	8:00
N5	8/18/13	904	726	14393	8:00
N5	8/18/13	681	659	14393	8:00
N5	8/18/13	780	192	19787	11:00
N5	8/18/13	614	233	19787	11:00
N5	8/18/13	1054	395	19787	11:00
N5	8/18/13	878	243	19787	11:00
N5	8/18/13	840	245	19787	11:00
N5	8/18/13	756	272	19787	11:00
N5	8/18/13	722	294	19787	11:00
N5	8/18/13	909	315	19787	11:00
N5	8/18/13	902	356	19787	11:00
N5	8/18/13	838	361	19787	11:00
N5	8/18/13	986	457	19787	11:00
N5	8/18/13	919	478	19787	11:00
N5	8/18/13	847	431	19787	11:00
N5	8/18/13	857	505	19787	11:00
N5	8/18/13	842	565	19787	11:00
N5	8/18/13	818	558	19787	11:00
N5	8/18/13	878	625	19787	11:00
N5	8/18/13	645	496	19787	11:00
N5	8/18/13	664	435	19787	11:00
N5	8/18/13	698	341	19787	11:00
N5	8/18/13	799	650	19787	11:00
N5	8/20/13	1218	241	10792	6:00
N5	8/20/13	1191	212	10792	6:00
N5	8/20/13	1083	216	10792	6:00
N5	8/20/13	1027	260	10792	6:00
N5	8/20/13	1140	385	10792	6:00

N5	8/20/13	1078	443	10792	6:00
N5	8/20/13	1111	495	10792	6:00
N5	8/20/13	1111	469	10792	6:00
N5	8/20/13	1112	537	10792	6:00
N5	8/20/13	1112	589	10792	6:00
N5	8/20/13	1054	527	10792	6:00
N5	8/20/13	1037	609	10792	6:00
N5	8/20/13	981	587	10792	6:00
N5	8/20/13	919	508	10792	6:00
N5	8/20/13	849	450	10792	6:00
N5	8/20/13	838	488	10792	6:00
N5	8/20/13	828	537	10792	6:00
N5	8/20/13	818	580	10792	6:00
N5	8/20/13	909	673	10792	6:00
N5	8/20/13	873	674	10792	6:00
N5	8/20/13	791	808	10792	6:00
N5	8/20/13	717	813	10792	6:00
N5	8/20/13	676	745	10792	6:00
N5	8/20/13	638	767	10792	6:00
N5	8/20/13	604	810	10792	6:00
N5	8/20/13	575	941	10792	6:00
N5	8/20/13	683	861	10792	6:00
N5	8/20/13	1189	940	10792	6:00
N5	8/20/13	1189	912	10792	6:00
N5	8/20/13	1030	854	10792	6:00
N5	8/20/13	737	996	10792	6:00
N5	8/20/13	1239	322	16184	9:00
N5	8/20/13	1157	325	16184	9:00
N5	8/20/13	1246	433	16184	9:00
N5	8/20/13	1215	501	16184	9:00
N5	8/20/13	1094	320	16184	9:00
N5	8/20/13	1040	267	16184	9:00
N5	8/20/13	1094	416	16184	9:00
N5	8/20/13	1111	474	16184	9:00
N5	8/20/13	1121	519	16184	9:00
N5	8/20/13	1123	565	16184	9:00
N5	8/20/13	1090	584	16184	9:00
N5	8/20/13	1114	644	16184	9:00
N5	8/20/13	1095	709	16184	9:00
N5	8/20/13	763	851	16184	9:00
N5	8/20/13	726	671	16184	9:00
N5	8/20/13	820	645	16184	9:00

N5	8/20/13	948	597	16184	9:00
N5	8/20/13	974	611	16184	9:00
N5	8/20/13	1040	705	16184	9:00
N5	8/20/13	837	478	16184	9:00
N5	8/20/13	890	342	16184	9:00
N5	8/20/13	972	265	16184	9:00
N5	8/20/13	1016	635	16184	9:00
N5	8/20/13	1244	847	16184	9:00
N5	8/20/13	1258	960	16184	9:00
N5	8/20/13	1249	1008	16184	9:00
N5	8/20/13	1147	859	16184	9:00
N5	8/20/13	1032	755	16184	9:00
N5	8/20/13	931	729	16184	9:00
N5	8/20/13	842	549	16184	9:00
