# Supplementary figures and images for: Interactions Increase Forager Availability and Activity in Harvester Ants
Source: PLoS One. 2015 Nov 5;10(11):e0141971. doi: 10.1371/journal.pone.0141971 (PMC4635008; doi:10.1371/journal.pone.0141971)

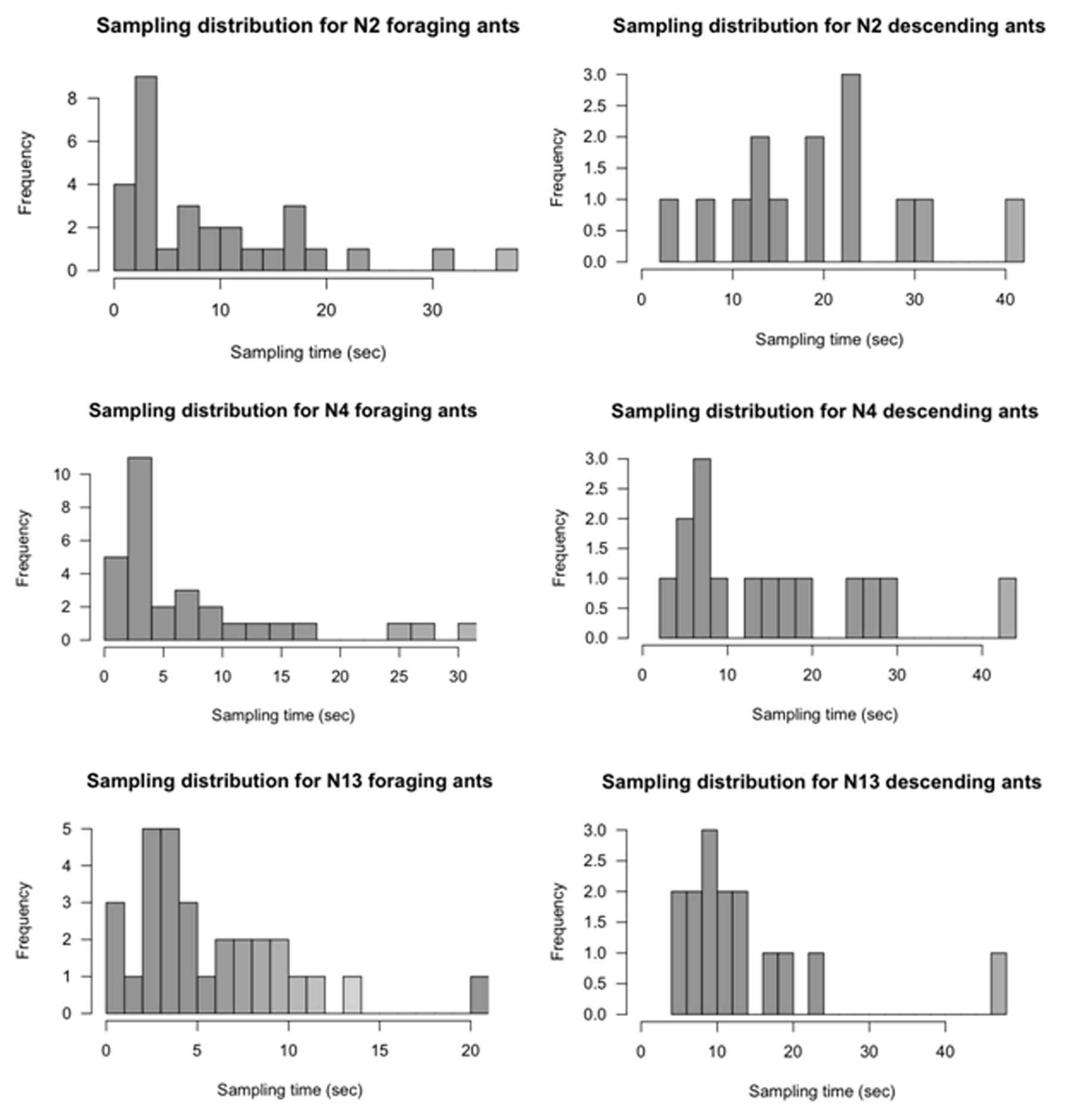

Supplement: S1 Fig — These histograms show the distribution of times that foraging and descending ants filmed in 2012 remained in the entrance chamber. (TIF) [file pone.0141971.s006.tif]
