# Supplementary material for: Interactions Increase Forager Availability and Activity in Harvester Ants
Source: PLoS One. 2015 Nov 5;10(11):e0141971. doi: 10.1371/journal.pone.0141971 (PMC4635008; doi:10.1371/journal.pone.0141971)
Supplement: S1 Table — (DOCX) [file pone.0141971.s008.docx]

| **Colony name** | **Date filmed** | **Removal experiment performed?** |
| --- | --- | --- |
| N_2 | 8-13-2012 | No |
| N_2 | 8-14-2012 | No |
| N_2 | 8-15-2012 | No |
| N_4 | 8-13-2012 | No |
| N_4 | 8-14-2012 | No |
| N_4 | 8-15-2012 | No |
| N_13 | 8-13-2012 | No |
| N_13 | 8-14-2012 | No |
| N_13 | 8-15-2012 | No |
| N_5 | 8-17-2013 | Yes |
| N_5 | 8-18-2013 | Yes |
| N_5 | 8-20-2013 | Yes |
| 367 | 8-18-2013 | Yes |
| 367 | 8-19-2013 | No |
| 367 | 8-20-2013 | Yes |
| 367 | 8-21-2013 | Yes |
| 868 | 8-19-2013 | Yes |
| 868 | 8-20-2013 | Yes |
| 868 | 8-21-2013 | Yes |
| 25 | 8-24-2013 | Yes |
| 229 | 8-24-2013 | Yes |
| 229 | 8-25-2013 | No |
| 229 | 8-26-2013 | Yes |
| 242 | 8-25-2013 | Yes |
| 242 | 8-26-2013 | No |
