# Supplementary material for: Interactions Increase Forager Availability and Activity in Harvester Ants
Source: PLoS One. 2015 Nov 5;10(11):e0141971. doi: 10.1371/journal.pone.0141971 (PMC4635008; doi:10.1371/journal.pone.0141971)
Supplement: S2 Table — (DOCX) [file pone.0141971.s009.docx]

| **Colony name** | **Date filmed** | **Length of film** |
| --- | --- | --- |
| N_5 | 8-17-2013 | 15:09:00 |
| N_5 | 8-18-2013 | 15:14:00 |
| N_5 | 8-20-2013 | 15:00:00 |
| 367 | 8-18-2013 | 15:00:00 |
| 367 | 8-19-2013 | 6:07:00 |
| 367 | 8-20-2013 | 45:00:00 |
| 868 | 8-19-2013 | 15:00:00 |
| 868 | 8-20-2013 | 15:00:00 |
| 25 | 8-24-2013 | 17:03:00 |
| 229 | 8-24-2013 | 17:02:00 |
| 229 | 8-25-2013 | 30:00:00 |
| 229 | 8-26-2013 | 15:00:00 |
| 242 | 8-25-2013 | 20:00:00 |
